# Identification and expression analysis of genes with pathogen-inducible *cis-*regulatory elements in the promoter regions in *Oryza sativa*

**DOI:** 10.1186/s12284-018-0243-0

**Published:** 2018-09-12

**Authors:** Weiwen Kong, Li Ding, Jia Cheng, Bin Wang

**Affiliations:** 1 School of Horticulture and Plant Protection, Yangzhou University, Yangzhou, 225009 Jiangsu China; 2 Joint International Research Laboratory of Agriculture and Agri-Product Safety of the Ministry of Education, Yangzhou University, Yangzhou, 225009 Jiangsu China

**Keywords:** Rice, Pathogen-inducible *cis-*regulatory elements (PICEs), Disease resistance, Disease susceptibility

## Abstract

**Background:**

Complex co-regulatory networks in plants may elicit responses during pathogen infections. A number of genes are activated when these responses take place. Identification of these genes would shed new light on understanding the mechanisms of rice response to pathogen infections and the elucidation of crosstalk among diverse signaling networks in rice disease resistance/susceptibility.

**Results:**

Here we report the identification of genes with pathogen-inducible *cis*-regulatory elements (PICEs) (AS-1, G-box, GCC-box, and H-box) in the promoter regions in rice. Our results showed that a set of 882 rice genes contained these four elements in their promoter regions. Of these genes, 190 encode disease resistance/susceptibility related proteins, and 70 encode transcription factors. Analyses of the available microarray data demonstrated that 357 transcripts were differentially expressed after pathogen infections. 48 out of 53 differentially expressed transcription factors are up-regulated or down-regulated by more than 1.1-fold in response to pathogen infections. Analyses of the public mRNA-Seq data showed that 327 transcripts were differently expressed after pathogen infections. A total of 100 up-regulated genes and 37 down-regulated genes were found in common between the microarray and mRNA-Seq data.

**Conclusions:**

We report here a set of rice genes that contain the four PICEs, i.e., AS-1, G-box, GCC-box, and H-box, in their promoter regions, of which, 53.5% were up- or down-regulated when pathogens attack. The PICEs in the gene promoters are critical for rice response to pathogen infections. They are also useful markers for identification of rice genes involved in response to pathogen infections.

**Electronic supplementary material:**

The online version of this article (10.1186/s12284-018-0243-0) contains supplementary material, which is available to authorized users.

## Background

Rice plants are exposed to a number of pathogens that may negatively affect their growth and development. To adjust to pathogen infections in their environment, rice plants have evolved complex innate immunity systems. It has been established that plants evolved pathogen-associated molecular pattern (PAMP) -triggered immunity (PTI) and effector-triggered immunity (ETI) to defend against pathogen infections (Jones and Dangl 2006; Chisholm et al. 2006). PTI is the first layer of plant defense, and it can be activated by the perception of the PAMPs through plasma membrane-localized pattern recognition receptors (PRRs) (Li et al. 2016; Macho and Zipfel 2014). Then the downstream components such as mitogen-activated protein kinase (MAPK) cascades are activated and responsible for leading to defense responses (Chisholm et al. 2006). The second layer of plant defense is ETI, which would be activated by the specific recognition of pathogen effectors through resistance (R) proteins, e.g., nucleotide-binding and leucine-rich repeat (NB-LRR) proteins, in plants. The defense response mediated by the R proteins is characteristic of hypersensitive response (HR), which is usually linked to the cellular production of reactive oxygen species (ROS) (Maekawa et al. 2011). Now, it is known that multiple signaling mechanisms, such as those mediated by salicylic acid (SA), jasmonic acid (JA), and ethylene (ET), are necessary in the transduction of the signal of pathogen perception into rapid defense responses (Vlot et al. 2009; Robert-Seilaniantz et al. 2011).

Numerous genes in rice plants are rapidly or strongly activated at the transcriptional level by various pathogen attacks. Some of these genes are vital in the perception of pathogen infection (Nasir et al. 2017). Some other genes can be induced by plant hormones that include SA, JA, ET and brassinosteroid (Shigenaga and Argueso 2016; Yang et al. 2013). These plant hormones have been demonstrated as secondary signals after pathogen attack. Some genes are implicated in different primary and secondary metabolic pathways (Cho and Lee 2015). Another group of genes, named pathogenesis-related (*PR*) genes, has often been found to be induced in defense against pathogens (van Loon et al. 2006). Some genes, including transcription factor genes and *PR* genes, in other species, were observed to be strongly activated after inoculation with pathogens (Zander et al. 2014; Sohn et al. 2006; Meng et al. 2013). Similarly, previous study has shown that in incompatible interaction between rice and blast pathogen, rice genes implicated in defense were more rapidly and strongly induced at early infection stage, and this is in contrast to the genes in compatible interaction (Wang et al. 2014). It was reported that OsWRKY45 could be strongly activated after blast pathogen infection, and it could be also induced by salicylic acid and benzothiazole, leading to strongly increased resistance to *Magnisea oryzae* or *Xanthomonas oryzae* pv. *oryzae* (Shimono et al. 2007).

Many pathogen-inducible genes were found to contain specific *cis-*acting element sequences within their promoters (Yin et al. 1997; Lebel et al. 1998; Rushton and Somssich 1998). Deletion analyses of pathogen-inducible promoters have identified such sequences that are sufficient to confer responses to pathogens. Up to date, much effort has been made to identify diverse *cis-*acting elements within pathogen-inducible promoters. AS-1 (activation sequence-1, TGACG) *cis*-element was originally found in some viral and bacterial T-DNA promoters (Lam et al. 1989; Bouchez et al. 1989). The equivalent and homologous sequences are also named *ocs* or *nos* cis-regulatory elements (Ellis et al. 1993; Kim et al. 1993). This element is characterized by two TGACG motifs. G-box (with the core sequence ACGT) was first characterized in the promoters of plant light-regulated genes (Giuliano et al. 1988). Since then, as a ubiquitous *cis-*element, this *cis*-element motif was identified in the promoters in many various plant genes (Menkens et al. 1995). An H-box *cis-*element was found to be adjacent to G-box in the promoter of one soybean chalcone synthase encoding gene. H-box [CCTACC(N)_7_CT] was first identified in the promoter of the bean *chs15* gene, which encodes chalcone synthase (Loake et al. 1992). This *cis-*element is critical for the induction of the expression of the *chs15* gene by elicitors and other stress stimuli. GCC-box (AGCCGCC) was found over-represented in the promoters of many *PRs* (Sato et al. 1996; Zhou et al. 1997; Manners et al. 1998). The *cis-*element has been shown to be responsive to ET and jasmonate, and functions after pathogen infection, followed by the induction of plant defense responses (Brown et al. 2003; Manners et al. 1998; Ohme-Takagi et al. 2000).

There are a number of *cis*-regulatory elements found in pathogen-inducible plant promoters. Some of the elements are occurring with high frequency, others with low frequency. The above four *cis*-regulatory elements have been shown present with moderate frequency in the pathogen-inducible promoters. Also, they are together involved in SA, ET, and JA signaling pathways.

Rice is one of the important food crops. Currently, the rice genome sequence is available, which provides an excellent platform to discover pathogen-inducible *cis*-regulatory elements (PICEs) within the promoters at the genome level. In this paper, we identified the AS-1, G-box, GCC-box and H-box *cis-*regulatory elements in the whole promoters in *Oryza sativa* cv. *japonica* (*O. sativa*) using in silico approaches. Analyses on the functional classification of the putative proteins encoded by the identified transcripts revealed a number of disease resistance/susceptibility proteins, transcription factors, protein kinases, and unknown/hypothetical proteins.

To test the significance of these four PICEs present in the promoter regions, we compared the transcript levels of the rice genes controlled by the promoters with these four elements after pathogen infections to those of these genes after mock inoculation. We found that the transcripts from a great number of genes controlled by the promoters with these four elements were up-regulated or down-regulated after pathogen infections, indicating that the rice genes containing these four PICEs in their promoters are responsive to pathogen infections. Our results indicated that the genes with PICEs in their promoters are important for rice response to pathogen infections.

## Results

### Identification of rice genes with PICEs in the promoter regions

The pathogen-inducible genes in host play an important role in disease resistance/susceptibility. To identify all the genes in *O. sativa* that contain AS-1, G-box, GCC-box and H-box *cis-*regulatory elements (Table 1) in their promoter regions, upstream sequences of 2 kb of 44,609 genes of *O. sativa* were downloaded from the EnsemblPlants database. The transposable elements, pseudo-genes, and noncoding RNA genes were discarded from the sequence dataset.

**Table 1:** Pathogen-inducible *cis-*elements, sequences and interacting factors

*Cis* element name	Sequence	Promoter^a^	Interacting factor	Reference
AS-1	TGACG	GSTs; PRs	bZIP	(Ulmasov et al. 1994); (Yang et al. 1997); (Strompen et al. 1998); (Chen and Singh 1999)
G-box	ACGT	CHS	bZIP/bHLH/MYC	(Droge-Laser et al. 1997); (Toledo-Ortiz et al. 2003); (Boter et al. 2004)
GCC-box	AGCCGCC	PRs	AP2/ERF	(Sato et al. 1996); (Buttner and Singh 1997); (Zhou et al. 1997); (Manners et al. 1998); (Rushton et al. 2002)
H-box	CCTACC(N)_7_CT	CHS	bZIP	(Loake et al. 1992); (Yu et al. 1993); (Droge-Laser et al. 1997)

^a^Model promoters containing the specific *cis*-elements are listed here

*GSTs* glutathione S-transferases, *PRs* pathogenesis-related proteins, *CHS* chalcone synthase

**Fig. 1:**
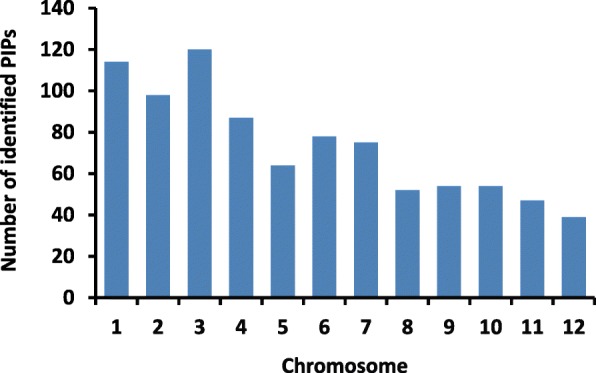
Chromosomal distribution of putative rice PIPs. PIPs: Pathogen-inducible promoters

The above four elements were identified collectively by custom Perl scripts in upstream genomic sequences. Application of this search led to the identification of total 882 sequences with the four *cis*-regulatory elements (Fig. 1). *Cis*-regulatory elements may occur on the direct strands or on the reverse strands of the promoter sequence contexts. In this study, we identified 487 sequences with the four *cis*-regulatory elements on the direct strand of the promoters, and 407 sequences with the four *cis*-regulatory elements on the reverse strand of the promoters. We found there were 12 sequences overlap between the two groups. We defined these 882 sequences as putative pathogen-inducible promoters (PIPs) in rice. Our results showed that the number of the identified PIPs present in each rice chromosome varied, ranging from 39 to 120 (Fig. 1). Then, we identified 833 genes (encoding 882 transcripts) driven by the PIPs from the *O. sativa* genomic sequences (IRGSP-v1.0.37) (Additional file 1: Table S1).

### Functional classification of identified genes driven by PIPs

**Fig. 2:**
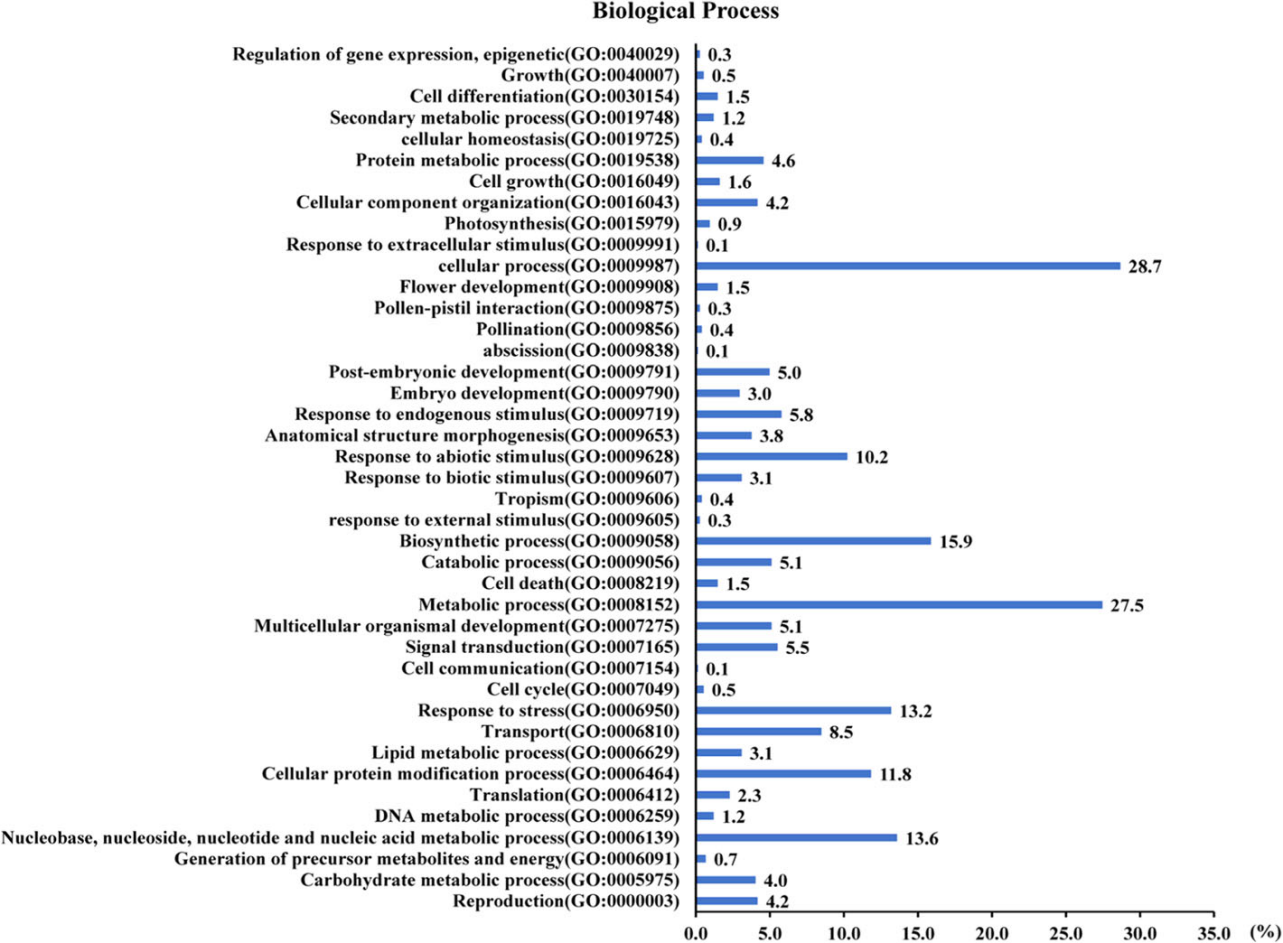
Functional classification based on GO-biological process for 743 identified candidate rice genes driven by PIPs

The sequences and annotations of these genes were obtained from the rice genome annotation project database (RGAP, http://rice.plantbiology.msu.edu/). Also, they were annotated through the InterProScan program. There were 743 transcripts whose annotations were obtained. The identified candidate genes/transcripts were classified on the basis of their putative functions. Analysis on Gene Ontology (GO) biological process of these transcripts showed that the maximum percentage of transcripts implicated in cellular process (GO:0009987) (28.7%), followed by metabolic process (GO:0008152) (27.5%), biosynthetic process (GO:0009058) (15.9%), nucleobase, nucleoside, nucleotide and nucleic acid metabolic process (GO:0006139) (13.6%), response to stress (GO:0006950) (13.2%), and cellular protein modification process (GO:0006464) (11.8%) (Fig. 2). These data suggest that the rice response to pathogen infection may activate metabolic and biosynthetic processes, as well as stimuli response. The pathogen infection may be a stress factor that promotes rice reproductive process. Classification analysis on GO molecular function presented that the top three group transcripts were protein binding (GO:0005515) (14.1%), catalytic activity (GO:0003824) (14.0%), and binding (GO:0005488) (14.0%). Also, transcripts encoded by candidate genes driven by PIPs involved in nucleotide binding (GO:0000166), hydrolase activity (GO:0016787) (9.8%), kinase activity (GO:0016301) and transcription factor activity (GO:0003700) (8.3%) (Additional file 2: Figure S1A). According to the GO cellular component annotation, the maximum percentage of transcripts localize to membrane (GO:0016020) (14.0%), plastid (GO:0009536) (12.1%) and plasma membrane (GO:0005886) (10.9%), followed by nucleus (GO:0005634) (10.2%) (Additional file 2: Figure S1B). Of the transcripts, 190 disease resistance/susceptibility-related (DRR/DSR) proteins and 70 transcription factors were identified through InterProScan annotation (Additional file 3: Table S2, Additional file 4: Table S3). Out of the DRR/DSR proteins, the maximum belongs to P-loop proteins, then followed by protein kinase domain proteins, serine-threonine/tyrosine-protein kinases, armadillo-type fold proteins, leucine-rich repeat domain proteins, cytochrome P450 and so on (Additional file 3: Table S2). Thus rice protein kinase, receptor and R protein encoding genes are involved in defense response to pathogen infections. It may be a rational explanation that the PIPs contribute to the activation of these genes during pathogen infections. Of the predicted transcription factors, zinc finger family proteins accounted for approximately 44% (31/70) and were the maximum group, followed by ethylene insensitive 3 or similar proteins (7%, 5/70) (Additional file 4: Table S3). Therefore, the zinc finger proteins are essential for rice response to pathogen infections, and ethylene signaling would be activated by pathogen infections.

**Fig. 3:**
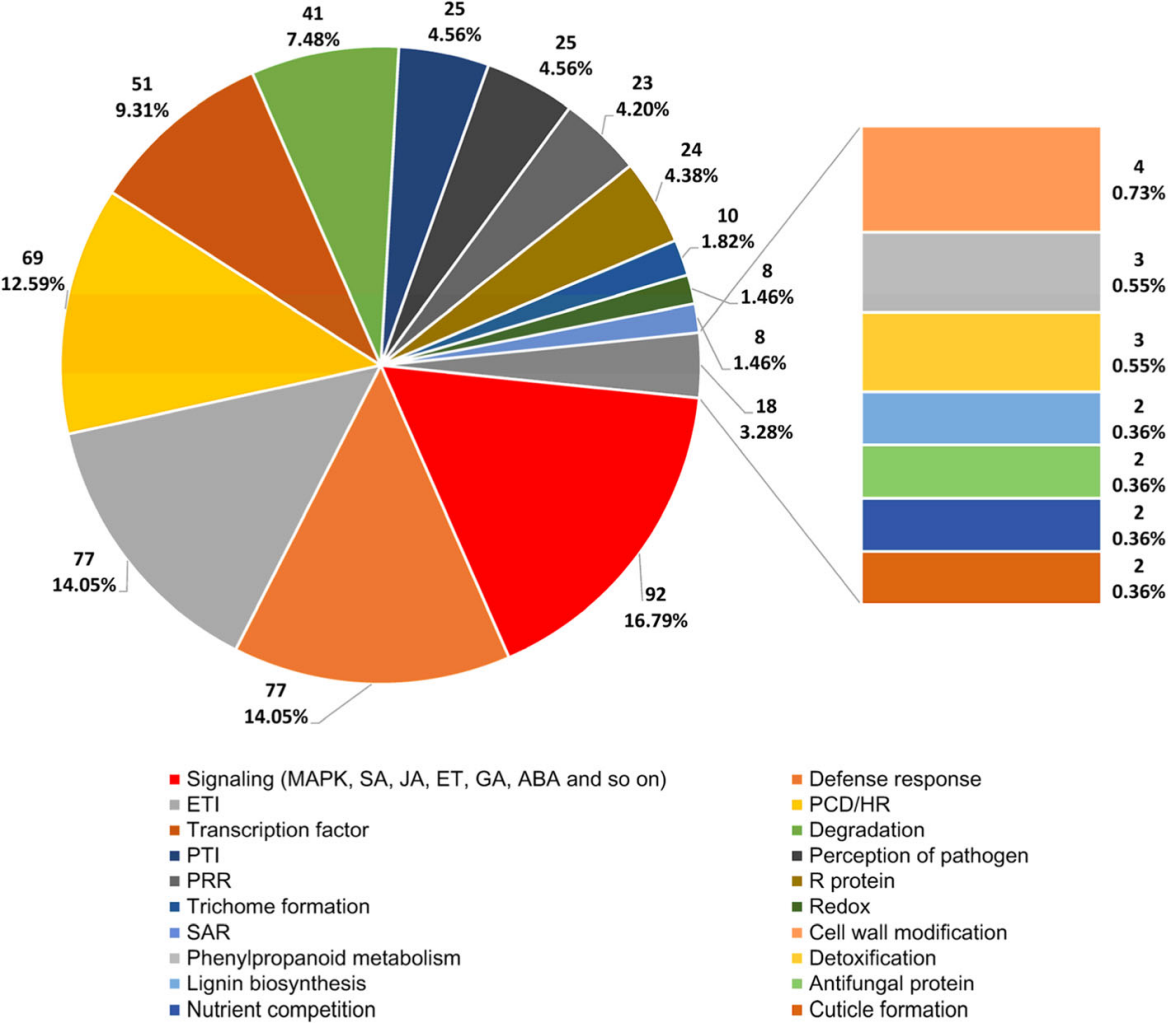
Analysis on disease resistance mechanisms of the identified DRR/DSR proteins encoded by the rice genes under the control of PIPs

A further functional analysis of the identified DRR/DSR proteins showed they were probably involved in many disease resistance mechanisms (Fig. 3). 16.79% (92) were involved in signaling pathways, including MAPK, SA, JA, ET, GA, ABA, etc. A group of 77 proteins (14.05%) was related in defense responses. Another group of 77 proteins (14.05%) was implicated in ETI. 12.59% (69) of the proteins were related to programmed cell death (PCD) or HR. 9.31% (51) were identified as transcription factors. 7.48% (41) were associated with degradation. Two groups of 25 proteins (4.56%) were implicated in PTI and perception of pathogen, respectively. Other proteins were involved in other than the above mechanisms, including R proteins, PRR, trichome formation, redox, and so forth (Fig. 3).

### Expression analysis of genes controlled by PIPs through microarray and mRNA-Seq data

We have identified 882 rice genes with the PICEs in their promoters. To answer the question whether the PICEs lead to the transcriptional activation or repression of these genes after pathogen infections, we analyzed the microarray and mRNA-Seq data available in GEO database. We searched 38 experiments (32 from microarray data and 6 from mRNA-Seq data) querying pathogen-activated or repressed gene expression changes. Some experiments were rejected for the reason that few differentially expressed genes were obtained from these experiments. In the end, 27 experiments were employed and these experiments consisted of 21 microarray as well as six mRNA-Seq data sets, including interactions between rice and *Magnaporthe oryzae*, *Rhizoctonia solani*, *Xanthomonas oryzae* pv. *oryzae*, *Meloidogyne incognita*, and *Meloidogyne graminicola* (Additional file 5: Table S4).

**Fig. 4:**
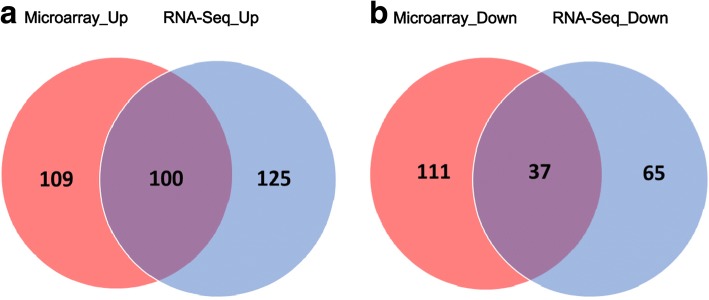
Overlap of differentially expressed genes between rice microarray and RNA-Seq data. (A) Overlap of up-regulated genes between rice microarray and RNA-Seq data. (B) Overlap of down-regulated genes between rice microarray and RNA-Seq data

In the microarray data analyses, a total of 357 differentially expressed transcripts were identified. Of these, 209 transcripts were detected to be up-regulated, and 148 were down-regulated (Additional file 6: Table S5). Analyses of mRNA-Seq data detected 327 differentially expressed transcripts. Out of these, 225 transcripts were shown to be up-regulated, and 102 were down-regulated (Additional file 7: Table S6). It is notable that 212 differentially expressed transcripts were identified in common from the microarray and mRNA-Seq data (Additional file 8: Table S7). However, the expression patterns of some of the transcripts, e.g., Os01t0229200–01 and Os01t0628000–01, are not consistent between these two experiments. This inconsistency may be caused by different conditions between the microarray and the mRNA-Seq experiments. The PICEs within the 2000 bp upstream regions of the genes encoding the 212 transcripts were highlighted (Additional file 9: Figure S2). A total of 100 up- and 37 down-regulated transcripts were discovered to be overlapped between the microarray and mRNA-Seq data (Fig. 4).

The predicted 70 transcription factors were separately checked for relative fold change in transcription during pathogen infections. The results showed that 53 transcription factors were differentially expressed, and a total of 90% (48/53) predicted candidate transcription factors were expressed more than 1.1 fold, by induction with pathogen infections (Table 2).

**Table 2:** The differentially expressed transcription factors encoded by the rice genes under the control of PIPs

InterPro annotation	Transcript-ID	Fold change^a^	adj. *P*.val/FDR^b^
Zinc finger	Os01t0123700–00	1.47	1.15E-02
	Os01t0960500–01	−1.15	2.59E-02
	Os02t0735900–01	1.4	0
	Os06t0192800–01	8.98	5.88E-03
	Os06t0687200–01	18.2	9.98E-03
	Os09t0419500–02	1.25	4.11E-02
	Os09t0468800–00	1.4	1.00E-03
	Os09t0535500–01	1.13	3.74E-02
	Os11t0572500–01	−1.8	3.96E-04
	Os03t0786400–01	1.16	1.19E-02
	Os03t0838800–00	1.26	3.36E-03
	Os10t0162300–01	−27.77	3.87E-03
	Os12t0583700–00	1.61	2.77E-02
	Os02t0727300–02	−2	0
	Os04t0686000–01	1.74	1.48E-02
	Os10t0467800–01	−1.13	3.54E-03
	Os04t0629100–01	4.28	4.67E-02
	Os04t0629100–02	4.28	4.67E-02
	Os03t0639600–01	2.5	0
	Os08t0159500–01	−6.19	2.52E-02
	Os06t0677700–00	−1.91	4.32E-04
	Os08t0491700–00	−3.36	1.40E-02
	Os09t0385700–01	15.9	0
	Os02t0724000–01	17.84	1.85E-03
	Os11t0292050–00	1.73	4.55E-02
	Os03t0619151–00	−1.77	3.46E-02
Myb/SANT	Os04t0665600–01	−1.19	2.53E-02
	Os05t0553400–01	1.99	3.24E-02
	Os03t0141100–01	−1.46	1.71E-06
	Os04t0463600–01	−1.98	2.87E-02
	Os02t0648300–01	1.57	1.97E-02
EIN3	Os03t0324300–02	1.4	0
	Os03t0324300–03	1.4	0
	Os03t0324300–04	1.4	0
	Os03t0324300–06	1.4	0
HLH	Os01t0195801–00	−1.47	4.59E-02
	Os02t0795800–00	− 1.16	3.87E-02
	Os09t0468700–00	−2.46	4.70E-02
HTH	Os03t0745000–01	−1.24	3.80E-04
	Os03t0745000–02	−1.24	3.80E-04
	Os09t0526600–03	1.5	0
AP2/ERF	Os02t0747600–01	−1.66	0
	Os05t0497200–01	1.47	1.74E-03
bZIP	Os02t0175100–01	1.15	2.50E-02
GRAS	Os07t0567700–03	−1.96	9.36E-03
NAM	Os03t0327100–01	1.68	1.17E-02
TFIID	Os03t0603300–01	1.6	1.00E-03
WRKY	Os05t0567200–00	1.17	4.24E-02

^a^Minus indicates down-regulation

^b^The values in blue are FDR (False Discovery Rate) measured in the RNA-Seq data analysis

**Fig. 5:**
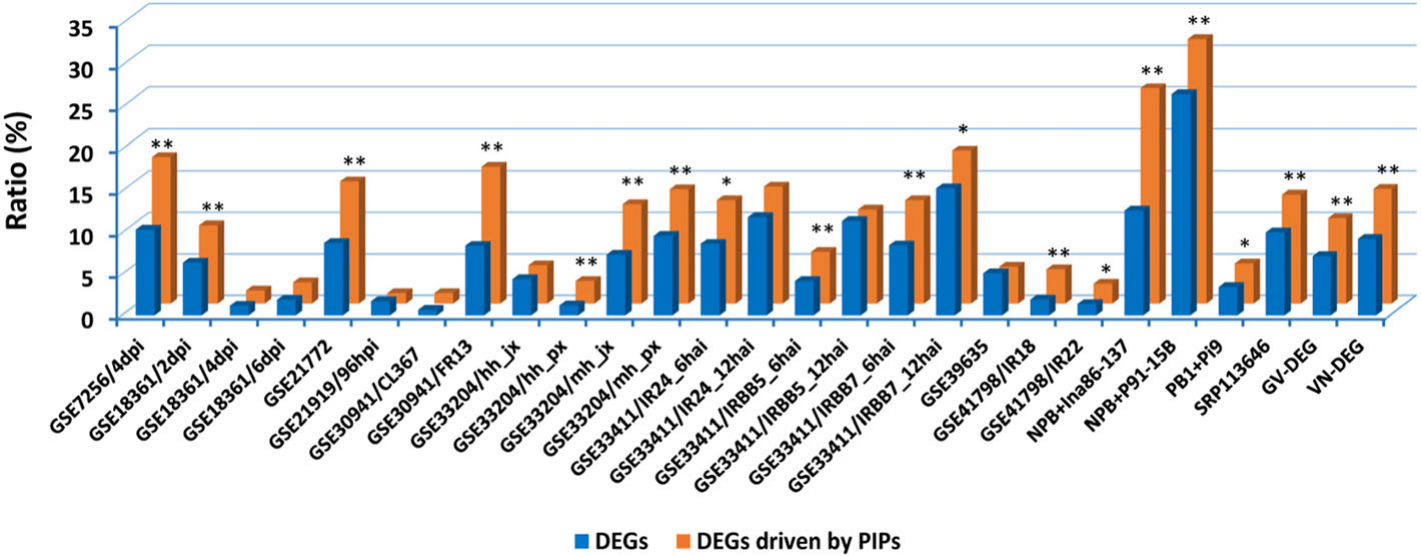
The enrichment analysis of the differentially expressed rice genes driven by PIPs. *P < 0.05, **P < 0.01 by post hoc Fisher’s exact test comparing each pair of data. The numbers of total reference genes were listed in Additional file/Table S10. *DEGs* differentially expressed genes

We randomly selected a pool of 50 genes without the four PICEs in their promoters from different chromosomes as random controls to examine their expression levels in these microarray experiments. In our analysis, no significant differentiation at the transcriptional level was found for most of the random control genes between the pathogen infected samples and mock samples (Additional file 10: Table S8, Additional file 11: Table S9). We performed an enrichment analysis, and found a higher incidence of the differentially expressed rice genes driven by PIPs with the PICEs in the total rice genes driven by PIPs than that of the differentially expressed rice genes in the total rice genes (Fig. 5; Additional file 12: Table S10, Additional file 13: Table S11). Thus there is a strong association between the PICEs and the differential expression of rice genes driven by PIPs after pathogen attack. Therefore, we infer that the PICEs in the promoters led to the differential expression of the genes under the control of PIPs after pathogen infection.

## Discussion

In this research, we performed a promoterome-wide search for AS-1, G-box, GCC-box and H-box *cis-*elements in rice and identified 833 genes, which potentially encode 882 putative transcripts under the control of pathogen-inducible promoters with these four *cis*-elements. Of the proteins encoded by these transcripts, DRR/DSR proteins account for 21.5% (190/882), and transcription factors constitute 7.9% (70/882). More than half (472/882) of the transcripts, encoded by the genes with PICEs in their promoter regions, were attested to be pathogen-inducible by the analyses of the available microarray and mRNA-Seq data. It can be expected that more of the rice genes, with the PICEs in the promoter regions, would be demonstrated to be pathogen-inducible if more analyses are carried out on the increasing microarray and mRNA-Seq data. Therefore, the PICEs are useful markers for identification of rice genes involved in response to pathogen infections.

AS-1 *cis*-element has been shown to be required for transcriptional activation of some genes. A functional plant *ocs*-element, a homologue of AS-1 element, was identified in the soybean *Gmhsp26-A* gene (Ellis et al. 1993). AS-1 *cis-*element was also found present in the promoters of some SA-inducible glutathione S-transferase genes (*GST*s), which are considered as defense genes and can be activated by SA, auxins, methyl jasmonate, hydrogen peroxide and pathogens (Ulmasov et al. 1994; Chen and Singh 1999; Yang et al. 1997; Xiang et al. 1996; Dudler et al. 1991). In addition, some pathogenesis-related protein encoding genes (*PRs*) were also found to contain AS-1 *cis-*elements in their promoters (Strompen et al. 1998). As has been demonstrated, the AS-1 *cis* element is an oxidative stress-responsive element and can be activated by SA with the help of oxidative species (Garretón et al. 2002). In plants, it can be bound by the TGA family of the basic/Leu zipper transcription factors (bZIPs) (Table 1) (Xiang et al. 1997; Johnson et al. 2001; Kim and Delaney 2002; Sarkar et al. 2017; Després et al. 2000). bZIP proteins have been demonstrated to play important roles in activating a number of defense genes (Alves et al. 2013; E et al. 2014).

G-boxes have been demonstrated to be implicated in the induced expression of some genes after pathogen attacks. Usually, these boxes play roles in concert with other *cis-*regulatory elements. G-box and H-box have been revealed to shape in combination the complex patterns of the bean *chs15* gene expression (Loake et al. 1992). These two boxes are also implicated in pathogen induction and other defense genes. Two proteins, named KAP-1 and KAP-2 respectively, were identified to bind to H-boxes in the promoter of the bean *chs15* gene (Yu et al. 1993). G-box and H-box could be bound by a bZIP protein, designated G/HBF-1, for activation of initial plant defense against pathogen attack (Droge-Laser et al. 1997). G-box also might be bound by the basic/helix-loop-helix (bHLH) proteins or the bHLH-leu zipper MYC proteins (Table 1) (Toledo-Ortiz et al. 2003; Boter et al. 2004).

ERFs (ethylene-responsive element binding proteins, i.e., ethylene response factors), belonging to APETALA2 (AP2) /ERF family, are ethylene-inducible DNA binding proteins that could recognize and bind the GCC-box element (Table 1) (Ohme-Takagi and Shinshi 1995; Brown et al. 2003; Ohme-Takagi et al. 2000). In addition, AP2/ERFs have been also revealed to modulate the expression levels of jasmonate-responsive genes through the interaction with the GCC-box (Brown et al. 2003).

Other than the above four *cis*-elements, GT-1 (GAAAAA; GGTTAA), MRE [A(A/C)C(A/T)A(A/C)C; ACC(A/T)A(A/C)(T/C)], and W-box (TTGACY), which were observed to be over-represented within the rice promoters in our analysis, are also common pathogen-inducible *cis*-regulatory elements (Park et al. 2004; Gurr and Rushton 2005; Rushton and Somssich 1998). Elicitor-responsive element (ERE) [AATTGACC; (C/T)TGAC(C/T); GTCAGAAAGTCAG] is another pathogen-inducible *cis*-regulatory element (Heise et al. 2002; Fukuda and Shinshi 1994). These four PICEs are as well occurring within the 2000 bp upstream sequences that match the 212 differentially expressed transcripts captured from the microarray and RNA-Seq data analyses (Additional file 9: Figure S2).

**Fig. 6:**
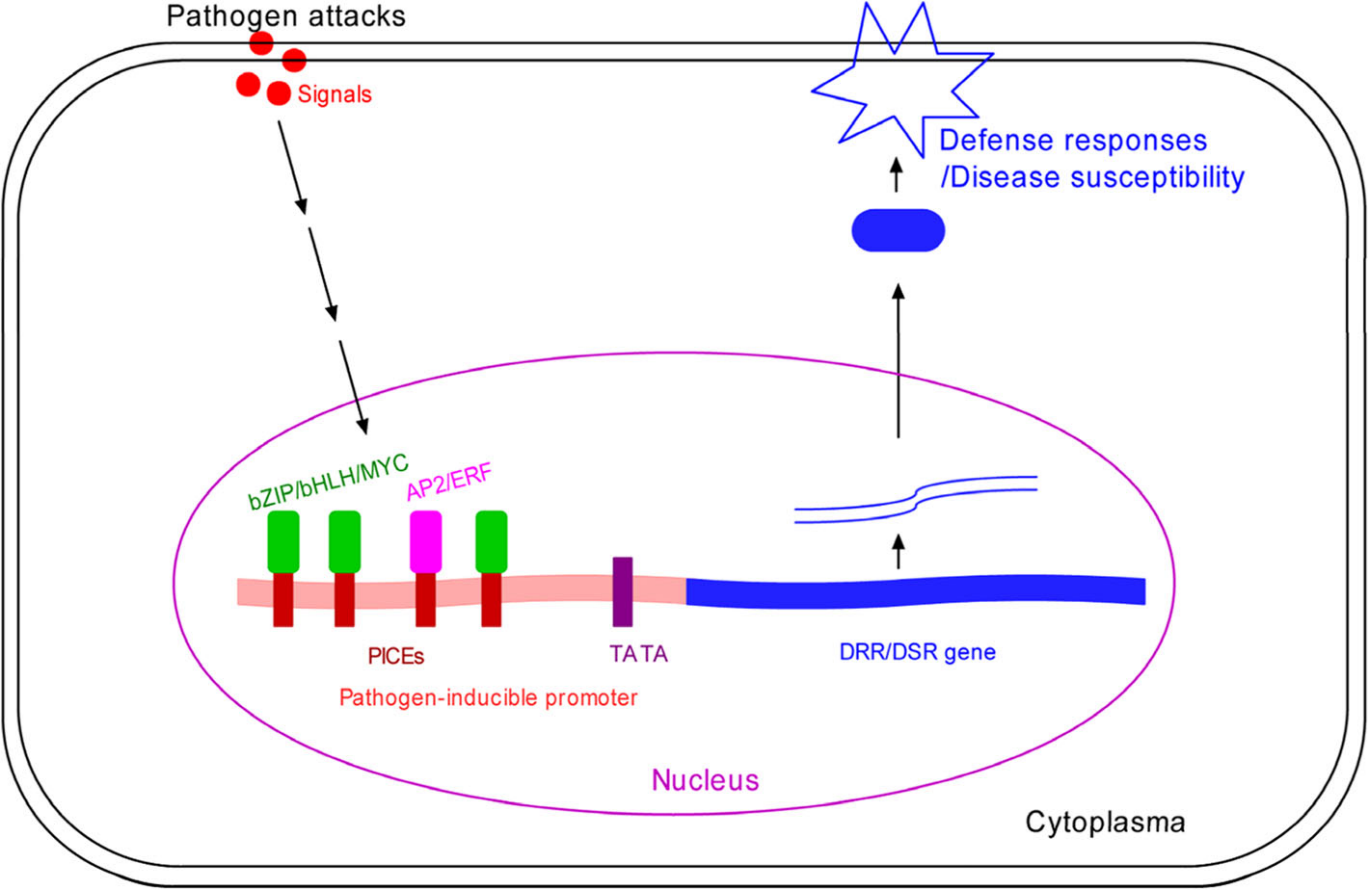
A working model for rice DRR/DSR genes under the governing of a pathogen-inducible promoter with the PICEs. DRR/DSR gene: disease resistance/susceptibility-related gene

It is well established on the basis of various experiments that promoters with particular *cis*-elements respond to specific triggers. The above PICEs are the target binding sites for specific transcription factors, such as bZIPs/bHLHs/MYC, AP2/ERFs, MYB (with recognition of MRE), and WRKY (interacting with W-box), which direct pathogen-inducible expression. We think that the combinatorial interactions of these PICEs in the rice promoters with their corresponding transcription factors are pivotal processes regulating spatio-temporal expressions of rice *DRR/DSR* genes during pathogen attacks. In other words, these genes respond to pathogen attacks through specific transcription factors which interact with the PICEs present in their promoters (Fig. 6).

Rice genes with the PICEs in their promoter regions are likely implicated in multiple defense mechanisms. A great number of the identified DRR/DSR proteins are protein kinase, protein kinase-like and phosphatase, which are potentially involved in signaling pathways, including mitogen-activated protein kinases. Many genes, e.g., encoding F-box-like domain proteins and ubiquitin-like domain proteins, are related to degradation. Some proteins, e.g., serine/threonine kinases, and NB-ARC domain proteins are likely involved in ETI. The leucine-rich repeat domain proteins are probably relevant to the perception of pathogen and PTI. There are some proteins functioning in defense response (e.g. cytochrome P450, thaumatin, chalcone/stilbene synthase). A lot of proteins belong to transcription factors, e.g., zinc finger proteins, heat shock transcription factors, bZIP proteins, and bHLH domain proteins. Several proteins, e.g., SKP1/BTB/POZ domain proteins, homeobox domain proteins, and ERF domain proteins, may be involved in SA, JA and ET signaling pathways, respectively. Other proteins, e.g., thioredoxin-like proteins, WAT1-related protein, and Barwin domain proteins, participate in redox reactions, cell wall modification, and HR, respectively. In our analysis, there are 17.1% (151/882) of the transcripts with unknown function. The genes encoding these transcripts contain PICEs in their promoter regions, indicating some unknown response mechanisms of rice to pathogen infections remain to be revealed. Our study sheds new light on rice molecular response mechanisms to pathogen infections.

The genes with the PICEs in the promoter regions in rice are likely involved in more than one regulation mechanism concerning disease resistance. All the rice genes identified here contain the PICEs in their promoter regions, they, therefore, can be potentially targeted by both bZIP and ERF transcription factors. It has been shown that bZIP and ERF transcription factors are implicated in SA, JA, and ET signaling pathways. So, the identified rice genes are likely to be activated through the hormone signaling pathways after pathogen infections. It seems that cross-coupling occurs between bZIP and ERF transcription factors and may, therefore, be of great importance in the modulation of the expression levels of the genes during the rice response to pathogen infections.

Identification and investigation of rice PIPs are valuable and useful in many aspects. Genetic engineering is an economical and convenient approach to improving rice disease resistance. Accurate regulation of exogenous gene expression is paramount to the engineering of rice plants with increased disease resistance. Many previous attempts to increase disease resistance employed constitutive over-expression of transgenes but again and again this caused poor quality or yield plants. It is now known that the poor quality or yield is attributed to the extensive cellular reprogramming connected with defence once unregulated defence reactions are triggered in uninfected cells. Therefore, compared with the constitutive expression of exogenous genes, gene expression under the control of PIPs is preferred in generating transgenic rice plants with resistance to pathogen attack, for this avoids the additional cost of resistance through restricting the expression to infected cells when needed. Therefore, it is useful to search promoters inducible by pathogens for rice disease resistance engineering. Our results would be a benefit for developing rice disease resistance engineering. The defining of a set of rice genes that are putatively regulated by PIPs will help elucidate the mechanisms by which rice plants respond to pathogen infections. It provides markers useful for subsequent study and will enable the elucidation of crosstalk among diverse signaling networks in rice disease resistance/susceptibility.

## Conclusions

A set of rice genes under the control of PIPs that contain the PICEs, AS-1, G-box, GCC-box, and H-box, were identified. More than half of the genes were activated or repressed after pathogen infection. The identified rice genes may be implicated in diverse disease resistance mechanisms. The PICEs in the promoters are important for rice response to pathogen infections. They are also valuable tags for identification of rice genes in response to pathogen infections.

## Methods

### Retrieval of upstream sequences of *O. sativa* genes

For identification of genes with PICEs in the promoter regions, upstream sequences of 2 kb of 44,609 genes of *O. sativa* were downloaded from the Rice Annotation Project database (Os-Nipponbare-Reference-IRGSP-1.0) (https://rapdb.dna.affrc.go.jp/).

### Identification of PIPs with PICEs and genes controlled by PIPs

The putative PIPs with AS-1, G-box, GCC-box and H-box *cis*-regulatory elements were screened out from the upstream sequences of 2 kb of 44,609 rice genes by custom Perl scripts. On the basis of identification of the putative PIPs from the rice upstream sequences, we further screened out the genes (or transcripts) controlled by these PIPs with PICEs from the whole rice genes.

### Functional annotation for genes controlled by PIPs

To infer the molecular functions of these transcripts, which genes were controlled by PIPs, we performed homology searches against the protein databases (Panther-12.0 (Mi et al. 2017), PfamA-31.0, PRINTS-42.0, ProDom-2006.1, SuperFamily-17.5, and TIGRFAM15.0) using the InterProScan ver. 5.25 program (Jones et al. 2014; Finn et al. 2017). To assign further functional information to these transcripts, we conducted a search using the Rice Annotation Project database, and then gene ontology terms were obtained and assigned to each identified transcript.

### Expression analysis of genes controlled by PIPs

The rice microarray data on pathogen inoculation vs mock were extracted from GEO (Gene Expression Omnibus) database of NCBI (https://www.ncbi.nlm.nih.gov/geo/). GEO2R, an interactive GEO-based web tool was used for the identification of significantly regulated genes across pathogen vs mock (GEO accession numbers: GSE7256, GSE18361, GSE21772, GSE21919, GSE30941, GSE33204, GSE33411, GSE39635, and GSE41798). We entered these GEO accession numbers and then defined groups as inoculation and mock with given number of replicates discovered in GEO. The false discovery rate method (Benjamini and Hochberg 1995) was applied to correct for multiple testing.

mRNA-Seq data (GSE81906: GSM2177582, GSM2177583, GSM2177584, GSM2177585, GSM2177586, and GSM2177587) on pathogen inoculation vs mock in SRA format were downloaded from the GEO Database and processed as follows using a combination of publicly available tools and custom Perl scripts. Quality was assessed using FastQC. Sequences were aligned onto the latest *O. sativa* genome assembly (released 37) using TopHat version 2.1.1(Langmead et al. 2009). Reads that map to a unique location in the *O. sativa* genome were adopted for differential expression analysis. The number of single mapping reads that locate each annotated gene in the *O. sativa* gene model annotations release-37 was counted by HTSeq-Count (version 0.9.1). Then, these data were provided as inputs to DESeq (R package, version 1.26.0) for statistical analysis of differential gene expression (Anders and Huber 2010).

Three studies presented complete information on differential gene expression. Therefore, some differentially expressed genes were obtained from these published data (Bagnaresi et al. 2012; Kawahara et al. 2012; Zhang et al. 2017).

## Additional files

Additional file 1: **Table S1.** Chromosomal distribution of the rice genes controlled by putative PIPs. (XLSX 21 kb)

Additional file 2 **Figure S1.** Annotation of the identified rice transcripts driven by the PIPs. (A) Annotation of Go molecular function of the identified rice transcripts driven by the PIPs. (B) Annotation of Go cellular component of the identified rice transcripts driven by the PIPs. (DOCX 1355 kb)

Additional file 3: **Table S2.** InterPro annotation for 190 putative disease resistance/susceptibility-related protein-encoding transcripts (119 groups) encoded by the rice genes under the control of PIPs. (XLSX 58 kb)

Additional file 4: **Table S3.** The putative transcription factors encoded by the rice genes under the control of PIPs. (XLSX 12 kb)

Additional file 5: **Table S4.** Pathogen-infected rice microarray and RNA-Seq data used in this study. (XLSX 13 kb)

Additional file 6: **Table S5.** Differentially expressed transcripts driven by PIPs with the PICEs identified from the rice microarray data analysis. (XLSX 20 kb)

Additional file 7: **Table S6.** Differentially expressed transcripts driven by PIPs with the PICEs identified from the rice RNA-Seq data analysis. (XLSX 20 kb)

Additional file 8: **Table S7.** Differentially expressed transcripts in common from the rice microarray and RNA-Seq data analyses. (XLSX 21 kb)

Additional file 9: **Figure S2.** The upstream sequences and putative pathogen-inducible *cis*-elements (PICEs) in rice. This document-like figure contains 212 upstream sequences (2000 bp) that correspond to the 212 differentially expressed transcripts obtained from the microarray and mRNA-Seq data analyses. The PICEs (AS-1, G-box, GCC-box and H-box) are highlighted in yellow. W-box, GT1, ERE (elicitor-responsive element) and MRE *cis*-elements are highlighted in green, cyan, magenta and red, respectively. (DOCX 165 kb)

Additional file 10: **Table S8.** Differentially expressed genes without the PICEs in the promoters identified from the rice microarray data analysis. (XLSX 16 kb)

Additional file 11: **Table S9.** Differentially expressed genes without the PICEs in the promoters identified from the rice RNA-Seq data analysis. (XLSX 13 kb)

Additional file 12: **Table S10.** The proportion of the differentially expressed genes in the total rice genes. (XLSX 12 kb)

Additional file 13: **Table S11.** The enrichment analysis of the differentially expressed rice genes driven by PIPs through Fisher’s exact test comparing each pair of the proportions of the differentially expressed genes in the total genes from Additional file 8: Table S10. (XLSX 11 kb)

## Abbreviations

AS-1: activation sequence-1
bZIPs: the basic/Leu zipper transcription factors
DRR: disease resistance-related
DSR: disease susceptibility-related
ERFs: ethylene-responsive element binding proteins
ET: ethylene
ETI: effector-triggered immunity
GEO: Gene Expression Omnibus
GSTs: glutathione S-transferases
HR: hypersensitive response
JA: jasmonic acid
MAPK: mitogen-activated protein kinase
NB-LRR: nucleotide-binding and leucine-rich repeat
PAMP: pathogen-associated molecular pattern
PICEs: pathogen-inducible *cis* regulatory elements
PR: pathogenesis-related
PRRs: pattern recognition receptors
PTI: PAMP-triggered immunity
R protein: resistance protein
ROS: reactive oxygen species
SA: salicylic acid
