# Supplementary material for: Identification and expression analysis of genes with pathogen-inducible cis-regulatory elements in the promoter regions in Oryza sativa
Source: Rice (N Y). 2018 Sep 12;11:52. doi: 10.1186/s12284-018-0243-0 (PMC6135729; doi:10.1186/s12284-018-0243-0)
Supplement: Supplementary file 2 — Figure S1. Annotation of the identified rice transcripts driven by the PIPs. (A) Annotation of Go molecular function of the identified rice transcripts driven by the PIPs. (B) Annotation of Go cellular component of the identified rice transcripts driven by the PIPs. (DOCX 1355 kb) [file 12284_2018_243_MOESM2_ESM.docx]

**Additional file**

**Figure S1. Annotation of the identified rice transcripts driven by the PIPs.** (A) Annotation of Go molecular function of the identified rice transcripts driven by the PIPs. (B) Annotation of Go cellular component of the identified rice transcripts driven by the PIPs.

(A)


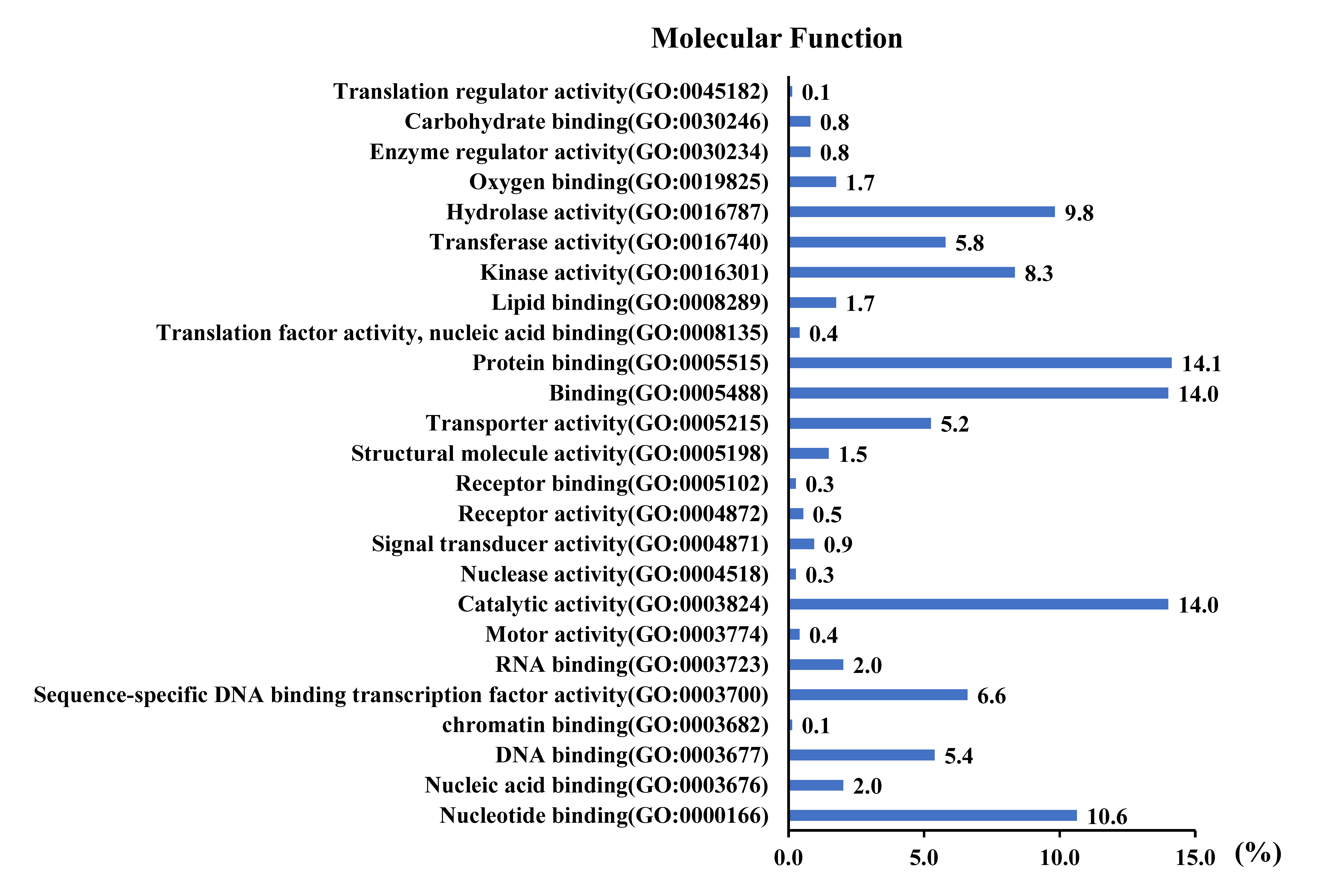


(B)





**Figure S1.**
