# Supplementary material for: Identification and expression analysis of genes with pathogen-inducible cis-regulatory elements in the promoter regions in Oryza sativa
Source: Rice (N Y). 2018 Sep 12;11:52. doi: 10.1186/s12284-018-0243-0 (PMC6135729; doi:10.1186/s12284-018-0243-0)
Supplement: Supplementary file 9 — Figure S2. The upstream sequences and putative pathogen-inducible cis-elements (PICEs) in rice. This document-like figure contains 212 upstream sequences (2000 bp) that correspond to the 212 differentially expressed transcripts obtained from the microarray and mRNA-Seq data analyses. The PICEs (AS-1, G-box, GCC-box and H-box) are highlighted in yellow. W-box, GT1, ERE (elicitor-responsive element) and MRE cis-elements are highlighted in green, cyan, magenta and red, respectively. (DOCX 165 kb) [file 12284_2018_243_MOESM9_ESM.docx]

**Figure S2. The upstream sequences and putative pathogen-inducible *cis*-elements (PICEs) in rice.** This document-like figure contains 212 upstream sequences (2000 bp) that correspond to the 212 differentially expressed transcripts obtained from the microarray and RNA-Seq data analyses. The PICEs (AS-1, G-box, GCC-box and H-box) are highlighted in yellow. W-box, GT1, ERE (elicitor-responsive element) and MRE *cis*-elements are highlighted in green, cyan, magenta and red, respectively.

>Os01t0123700-00 2000 bp upstream sequence

CGTCGTCGTAGCTGCATCTCTCGCAGATGACATCCCCGTTCGCACACTGTACATGAACCAATCAATTACGTCAACATCTAGAATGTATTTTCTACACCTAAGTGCATGCATGTAATTTAAACCATTATATAAAAATTTTATAAAATTTGATAAGATAGACTATCATAATATAAGACACTATAAACATGTAAGTCTAAATTTGATCGAGAAACAAAAATAATAAATTTTATCTCTATTCATTGTCTGATTTTATTATTTTTGTTTCTCTAGTTGTAAATCAAATTTAGTCCTGCATGTTTATATACATACTTATCACAATCGATGTTACTAAATATTTTGAATTTTTTCTATAACTATTTAAGTCAAAATCCTTGTCCGGTCAACATCGGCAACGAATCGATCGATGCAACATGAACTGATCTATACTACCTCGTAGATGGGAGAGGAGAGCATGGCGAAGCATCCGTCGCACCGGAAGATGCCGTCGTCGGCGCCGATGCCGTCGATGCGGACGGTGAACTCGTTATCCGCAGGGGGATGGTACCACGACCCATCCTGCTCGTCGTCGTCGTCTTCCCTGTCGTCGGAGCGCGGCGACGGCCTCGTGTGGCAGCACCGGCTGTCCTCGCACTCGTAGCTGCGTGTGCGGCTGCGGCTCCGGCAGCTTCTTGGCCGACGACGACGACGTCGGCGGGGGCTGTTGCTCCGGCTGCGGCCGCGGCGGCGAGAGTACCCTTCCTCGTCGTGACGACGACGGCGGCTCGGTTGGTAGGCCGGCGACAGCGACCAACGGCTGCCCTCAAGGTAGACCGGCGATTCCGGCCCGGTCGTCGGGTCTCGCCGCCGGCGCCGGGCCGTGCCGGCCGCCGGGATGGCGATCCTGTCGCCCACGCGGCCGCTGCTCATCTTGGGCGCTAGAATCCTCTGCTCCCGAATATCTCGCCGATGAAACAGATTTTATAACGATCTGCTGCATGCTTAAGAGTTAAGGAGTCTTGGAGATAGGAGATTGTGCCAAATACTTCACCAAGAGTATTTCGGAAGGTTTGTTTTAATTTAAGAAATTTTATCTTGTTTTAAGAAAAAACGGGAACTTTCGAAAATTGTGTTGTTATTAAAACTCCACCATAATTTGATAAGACCTTAAATAACCTCACTCTTCTTTACCTTGCCAAAATTGTAGCTTTACTATATATTTTCTGTTTATTGAATTAAATTTTCAAATATACTTTTTTTCTGATTGTCCGGGATTAATATTTAAGAATATTTGCAAAAATATACCTGTGCGGCCAATTCATCATGCGATGCAAGATCAACATTGCAATTTTAGTTTATTTTAACTACAATTGTGTTGTTTTATTTGGTTTTATTTTATTTCTATCTCAGTTTGAAATTTTTTTTGCATGTAATTTTGTAACTTTTACTAGTTATAAGTTTTTTTTTCATGTATTTTTCTAATTTTCTAGACACTCCTATGGAGAAAAAACTTAAAGTATAACCTGTTAGATCGTGATGTACCTAAAACAATGTAGATTAATGGCAAAATTTGCATAAACCATCACAGTTGCCACTCTAGTTTTAAGATACGACCCTAGAACCCATTTTGTGGCAAGTCCAACAACACTATCAGGCCAAACTGCTCGCTCCGTACTCATGACTATTACTCCATCCATCCCATAATATAAGAGATTTTAGGTGAATATGACATATTATAGTACAATGAGATACGACGTGTCACGTCCACCCAAATCCTTTATATTATAGGACCGAGGGAGGAGTTGATTAGGACCATTTCTTCCAATATGGATTTGCTACCGATGACTGCCTCACCTATGCGCTCAACCCTCTCGCCGTTCACAATTGATCCCTTTGGTCGATTTAATCTAGTAAGACAATAGATCTTCGGTGCCTCAAGGCTCAAGCTACTGGGTGAGTTCCATTTTAACTTATTACAGAGAAGCAAATAAGCTCAAGCTTTTCTTCTGCGCAGCATTATATAT

>Os01t0143300-01 2000 bp upstream sequence

GACAACACGTACAGGAGCACGTCGACAGTTCAAGGCACGCTGCAAAATGCCCGGGACTTCCTCGGCGGCTGCAAGTCCCACGGGGAAAAGTACAAGAAGCTGATCGAGCTGGGGCGCTCGAAAATAGTGATGGACCACGAGATGGCGCAGAAAAGCAAGGACATCATCTACAGCTACGACTCCGACGAGGAGAAGGTGAAGAAGATGATCGAGCTGGACAACTCGACGAGCAACGACACGGTGAACGTGCTTAAGATCCTGTCTCAGGGCGCGAGTGTCGCCGTGTACCTCGTCGATAGGATCGAGGACACGAGAGAGCGGTGGAAGGTGCTCGCCGCTTTCTGGGCGAACCTGATGCTGTACATCTCCCCGTCGGATAGGGCGGTGGCACACGCCACCAGGATGGCGACCGGCGGCGAGTTTATAACGATCCTATGGGCTCTGCTGACACATGCTCATGTGGTAGACCCGCTCCAGTCGCGCGGCGGTAACTCGGGATTGCATATGCAGCTTGAGGAGGAAGAGAGGAGGCGGCCGCTGATCGAAGAGCAAGAGATGGAGCTGGTGACGAGGCGCAAGCTGCGCGAAGAGCAAGAGAGGAACATGCAGATGCAGGGCCAGCCGCCGATCCAGCCCTGATCCCATCATCTATTCATCTTATTGTTCGTTGTCCCCTGTCCTTCAGTCCTTGTCCATAAGAATTGGCATGGTCACCGGCGAAGGGTGTCACTGTGCGTAGATGCTACTACTCCCTCAGTCCCATAATATAAGGAGATTTTGCCCTTTCTACTTGTCCCACCATATAACACAAATTAAGTATACTTTAATTACCTGAATGACACAAATTAAGTATACTTAGAGAAATCTACAGTATATTAACAAAATAAGTAAAAAAACAAAATGAGTATAGAAAAAATGCTCGAAATGATTTAGTTGTTCCATCACTTCTTATGTTATTATAGCAAATCACACCAACCTTGCAAGGCGTTGCATCCGTGCGCTCACTTGTTAGATTCTTGTACTAGATTGTACCCACATATCAAATTGTTGCGCTACCACGCTTACTTCTCAAGTTTTTACACTAAATTGCACTTGCATGTTAAGTTGATGTACCATTAATGCAATTTATTCATCATGTATAATACTATTTGTTATTGTATAGCTAGCTTACAAATTGGAATGATCAAATTGAGTTAAACTAAGGAGATAGTCATTGGCACCTCAAAATTATGGGAGTTAGGCTCCACTGCAATAGGCAGTAAATCCTCAGGTTCAACTTTTCAATTGAGTGCCGGATTCCAACTCCTAATGGTAATGGTATGAAGGTTTTCATCAAATTTTGGTAAAACCAAAAATCCCCATCAAATTTAATGTTTCAAATTTGAATTCTCAAAAGTGATTCAAAAATTTCACACGGAATAGCTTTTGTCCCACATCAAAACCTTGAATTTTCACAGTTTCTTGCATTTTTGTCAAAAATGTGAACATTTACTCCCTGCGTAAAAAAAAACTTCTATCTCGAACCTGAATATGCCTATGCCGAGGTTCCTATCTAGAAATGTTTTTTCGATGAAGGGAGTAAAGTGGTGTTTGTTTTTACTGAAAATGAATACAAAATTAGGATATAGAAAAAATCCATTAGCACATGATTAATTGAGTTGTAATTGTTACAAACATAAAAAATAGATTTATTTGATATTATAAAGCAACTTTTATATACAAAGTTTTCATATAAATACACCATTTAGCCGTTTAAAAAATATTGCTAAGGAAAATCGATGTAAAATTTAAATTTTAATGAAAAAGGAACGGGTCTAATAAGTTATGGCGACAGAACTATTTTTCTTCCTCTCCAAGAAGCGAAGAATATTGCTTATAGATCGAATGAACATACTGACCAAGTTTTGACAGAATGACAGGTGGGGCCCATGCCCCTACCTTTTTTTTAAAAAAAAAATTCCCTTCTTAAAACAACACAAATTCTCGCCTCCCCATCTTCC

>Os01t0183800-01 2000 bp upstream sequence

GTCGACGCCGTCGCGGCCGTCGGGGTCGCGGAAGAACACCTCCGGCACCAGCAGCGCGCACGCGATGTGCGCCCACCGCGCGTCCGTCGTGCGCTTCATCGCGCCGCCCCTCGCCGGGCAGAGGCAGCAGCGTGGCGGCGGCGGCGGCGGCTTGTTGCCCTTGCTCTTCTTGGCGGCGGCGGCGGTGCAGACGGAGCAGAACCAGTCGCCGTCGGGGATGAACGAGGCGAGCGGGTTGCCGTAGCAGGAGGCGTGCACCATGAGGTCGCACCCGTCGCAGAACACGATGGGGTCCGACGGGTCGCCGTCGGTGCTCCCGCACACCGCGCAGTGCACGCCGTCGTCCTCCTCTTCCACCTCCTCCTCCGCCGCCGCCGCCATGGCCTCTTGCGTTGGCTCTCGCGCCACTCTCTCTCCATCCCAGGAGTCTTCTTCCCGTGCTGCTTCGTTGAGATCAATCGCTTTGCAAGCTTTCTCGAGTGCTTCGACTTCAGATTGCTTCGCACTAATAGCACTGCAGGATTGATTGCATTCCTGATCTTTCGATTCTCTGCAAGTGTTTGATCTTTGTTTCTTCGACACTTGCTCCTTGATTTCTTCATGATTGATAGGCTTCTCCGGAATCTTGGGCTTCTCTTGAATCTTGGATGCTTCAGCTCTTGCATAATCATCTTCCACACCATTTGCCACGCTCGAATTGGTGGTGGCGCCACGTTTATTGGATCCTTGAGGCTTGGCTTCTTTAACAGCGGCGACGCGAGCTCCATCCGACTCCTTGGATGGAGGCGGCGATGGCGGGAGAAACGGCGGCATCACGCGCTTATTGCTCGGTAGGCAAACCGAGACCGGAGCGGCGGTGTCCTTCGGGGACGGCGGCGCGCGGGCGCTCTTCTTGGCCGGGATGCTCGGAGAAACGGCGGCGTCTCTCGGCGGCGGCAGGGTGTTGACGAGATCCTTGAACGGGACGCGGGGAGAAGCGTCAGTAGCCGCCGCCGCCGCCGTCGCGCTGACGCGCTTCTTAGCAGGGACCGGGATGCTCGGAGAAACCGCCGCCTTCTCCGGCGCCGGCGCCGGCGGCGGTTGCGGCGCGTGAACACGCTTCCTGACCGGGACCGGGATGCTCGGAGAAATAGCAGCCTTATCCGCCGCCGCCGGTGGCAGAGTGTCGACGCGCTTCGTAGTCGGGACTGAGGGAGAAGCACCACTATCCGGCGGCGGCGGCGGCGGCGCGTGGATGCGCTTCTTGGCCGGAAGACACGCCGGCGCGACGGCATCATCCACCGGTGGCGCATAGGCGCGCTTCTTAGCCGGGAGGCACAGAGCGAAACCGCCCCCCTCCACCGCCGCCGCCGCCGCATAAGCCCGCTTCTTCGCCGGGAGGCAAACGGGGACGGCCGCCGCCGCCGCCGCCGCCGCCTCCAGCATCGGCGGGCACAACCGCTTCCTCGCCGGCAGGCACGGCGCCATCCCGAGACTGGAGCCGCCGACGTACTTGAACCGCTTGGAAGGCGGCAGATCACGCACGCTCCCGCTGCCGACGGCGAACTCCATGGACGGAATCGAGACCGGAGATTTTGCTACACGGGGAAGAAGAAGAGGAGGTTCGCGGCGGGTGATTTGGGATCGAAACGAACCGGGGGTTTAAATGACTGAAAATTTCGCGGTCGAATCAGCGAGATGGCGCGCGCGCAGCCGCGCAGCGAAGAATCTGACGAAATTTTGGCTCGGTGGGAAGCGAAATGTTTGTAATTTTTGTTTGAAATTTCGAATTTGGGGCGTGCGGGCTGCGGGAAAATTTTGCGTTTCGTTTGGATTTCGCGGCGGCGGCGGTGGCGCCAGCATTTCGCTCGATTCGGGCCGTGGGCTTGGGCCTAATTAGGTTGGGCCATGAAACCCTAGGTGGGCTCACTTTTTTTTTCGCCGATTTATAATTATAAAATTATTATCTTCTTTTTCGTCTATTGAAAAAGACGAGGGCCTAGTAGCCATCGTCGATCCCC

>Os01t0191500-01 2000 bp upstream sequence

CATAAAACCGTGGCTTGCACCTGCTGCGGTCATGCTCAAAGCACAGGCAGCACAGTTCCCCTCCCATGCGCCATGAGGACTTTTAAACATCATCAAGCACAATCCGCCAGCACATGCTTCCCTTGATGCCTTGACGGTTATTTTAAGTATTTACCTAATTGACAAGAAGAGTCCAACAATCATTAGCTGATCACTCTGCAACAGCTACCTTATTGAGAGAAGAAACATTACATGAGGAACTAAATGAAAATGTCGTGCTGGTAGGAAATTGTGACAACAAAGATATTTTACAACTGCAGGCAGTTTTTCCCATCTGAGATGCTTGCACTAAAACAAGAAGGTGCAGAATAATACTATGGAATACTATCAGTTAAGTGGATGTGCTGTCTCACAGACATGTCCAGGTGCTTCAGAAATTGTGTTACTTCAAGCAATGTAAAACTTTTCCATCGAAAACAGGAAGAATATAATATATTTGTTCAAGGAATCAATAACAACAACTTAAATAGAAGGAATATGAATCTGTTTTGCTCCAAATGCACACCACCAAATGGCACAGTATTTTTAGAAAATGGGTTAAACCCGGCCTCTACATCCAATTGGATGTACACAGACAATAACACAGTATATGGCGGTCTATTCTCAGGATGTAAGCTACATCCTAACTGGGAGGCATAGCAAACTGATAATGCAAGAGAGTTCTCCTGCTCTATTAACATATACAAGGCAAAGTTTTTGCCCTCCTTTTAAGGACTGCAAGAAAATTGTGTTAACATACAACCACCAATAGTGAAAATTTTTATTGACATTGGACAGTACACTATCCAAAGACCATAGGAACAAAATGCATTGCTGTGGTTGGCCAACAACTATAGCTAGCAGCTACTCCCTCATACAAGGCACATTACAATCAAGTTAATATGTGTCCCTCTTAAAATGATCAACGGATGTTAGGTCTCACAAGCACTCTAAAAGGTCTCCTGCATGGCTGCATAGATCATTGACACGAATAAAAGTCTCCTGCGAGAGCATTGACACAATCTCCTGTACGCAAATGACTAATCTGTACAGGTCCTCCACCTAATAAGCGTGATCTGATCAAACATCAAAGCAATAACTGTTAAGACCCTACACAAACGCCCACAATAACCAACTACCTCCATACACCAACCCTACGCATACACCAAACACATGCTCGAAATCTAGAGCCGTCCTTGGCAGCTTGTCCTGAATCTGTCGAAAGCCCACAGCTCAACAAGCAGATTTGGACCATTCTGACAAGCGACAACTTTGCACACGACACATCTTTCAGACAGAACAGAAGATGTACCCGCTAAAATTTATGAACTGAAACTGGAATTCCCTCAAACAGGTGGTGGTGGTCAGATCAGTTGCATTGCATACCCACCCGGGGATGATTTCAGCACAGAAACCATCACTCTCTCTTCACTCAATGCCGTGGAAATTGGAAGTCATCTACCACGCGAGGAGCAAATCCCCAGACGTAATTGCAAATGCGACAAAATTAACTAGACACCAACAGCAATAACTAGAAAGGGGAGAAAAAAAATACGAAACGCACAAGCACGTTCAAGCGTGACATCCCTAATCGGCATTTGATCAAGCAACTCATCTGCAAACTTTGAGGGAGGATTCGACCGGCGCCGTTAGCCTCACAACCGTCGCCGTCGCACGCCTACCCCGTCCCTCCGCGTAGCCGCCTCCGCCTTCGTCTCCGCCGCCGGAGACGTCGAGCCGGGGCGGCGCATCAACGGCGGCGACTCGGCGAGCGCCGGCGCAGCGGAGGATGGCTTTGTGAGAGAGATCAGATGGCCACGACTCCCCACGAGCCCACGAGAGGCGAGAGGGGTAAGGCCCAACGCATCTAGAAAATGGGCCAAAGTGGGCCCAATCGCCACAGAATTATTCCTCTTATTCGGCCCGGCCCATTTAAGAGCTCTCGCCCACTTCTATATGCGGACCAGCGCCTCGAATTTCTT

>Os01t0229200-01 2000 bp upstream sequence

ACCTCTGCATCTTTGTCATTTTGTCATTATACTTTTCTTTCAGCTGGGTGCTAACATTCTCATCAATGAGGTTGTGCTGCGGGCATAAGGAAAGGCAAACTGCCAAGAAGGCATACATCTGTTCATTTTTCTTCAAGATCTGGTCATACTGCGGGGACTTCTGATGGTACTGCTTATATTTCAGAATGTACAGCAAGATTTTGTTGAATTCACGTATGGCTTCAGCATATCTACCAGAAGAATATAGCATTAGCTAAAACAACCATCTTGGGCAATTTCAAGAAAGTGAATTAAAAAATAAATTCATAAAATGGGGTTTTACTGAAAAGAATGCTTATATCCTAGGTGTGGCTGTACAACATGACTCAATCTAATTTTACTGGAAATAATGTTTATATCCTAGGACGCATTTCATGTGATTTAAGTACAATTTGAACATAGAAGAGCAAAAAACTCTGCGTCTAACATGGCACTGTAGATTGAAGTATCCATTGATTCCAAGGCAGTCAATGAACTTTAGTACCTGCGCATCATAAGATTTGCAAAGCCATAGTGGTAGATAGCAGAGATGTGACTCCCAATCACAATGGTGTAGACCCCCTGCTGGTTAAGGTCGATTGGTGCCAAGCACTTGAGGCCAGTGCGGTAATCCCCAAGGAGGCAGTGTATCCTGAGCAGCCCAATCATGCTGTAATAACCGAGCATCTTGAGCACATTGCTGCCACCCTGGTAGTCGTAGCCGTCGGTGGCAGTGAACTGCTCGAGCCCTTCCTTCTCCCTCTCCAGAATCTGCGCGATCATGGACTTCTCCACCAGCGCTTGCAGGTAATTGAGAACCCCGTACACATTCCACGCCTGCAGCAATTCAGCAAACCACATTAGATTGTACACCACGGATAAGAACAAATCAGAAAATCATATTAGATTGTACCCCACAGCTAAGAACAATTCAGCAAACCGGATTGCCGATTATATTATGCGCCGCAGATAAGAAAGAAACTGAATTACATATTAGATTAGGCTGCAGCTAAGAAAGAATAGTAGTAATAATGCATAATCAAGAAGCATTGGTGGTGAGCTGGGTGTGTCACCTTGTCGAACTGCTTGAGCTGGTGGAGCTCGTCGTCGGACTTGTTCTTGAGCTTGGCGCGGTACTGGCAGAAGCTCTGGAACTGGTAGACGAACTCGTCGACCATGTCCCAGAGCCACTGGTTGGGGAGCTGCATGTTGACGACGCCGTGGAGGACGACGCTGAAGAGGTCGCAGTAGTTCGCCCACGACTCGGCGCGCTGGCCGGCGGTGAGCGGCGCGGACGACGACGACGACGAGGAGGCCCGCGCGTAGGCGTGGCGGTACCAGAGCTCCCGGTAGAGGAGGAGGAAGACGTGGTCGCCGTCGCAGTAGGGCGAGACGGCCTCCGCCGCCGGCCACGGCGCGTCGCGGAAGAGGCGGTCGGAGAGCCGCTGGAACCCGCCCTCGTACATCTGGTGGATCTCGTACACGTTCTTGTCCCTGACGTGCCTGTACATGTGCGCCACGAACGTCTTCACCGAGTCCGGCACGTAGCTCGGGTCGTACACCGCCCCACCTCCGCCGCCGCCACCCTCCGCCGCCGCCGCCGCCATTGCTAGGGTTTCGCGCTTCCGGCGGCGCGTGCGGAGGGAGGAAGACGAAGACGCCTCCCCGCTCCAGGTTTTCGTTTTTAATGGGCCTCAGTGTTTCGGCCCACGTAGAGTATGGGCCAAAATGGGCCTGGTAGTGGAGGCCCACGTTGAGGGAAAAGTGACAGTTATGATGTGGGGGACAGCTCAGATCCATCCGACAAGCCGCCACCTACCAAACGAACTCGTTAACTTTCCTAAAAAAAATAAAAAAAATGATTTATTTATTCATTTTTATTTTTTTTGGGAGAGCTAATTTGGCGGGGAGAAATTTCCAAGAAAAGAAAATAGGAGGAAATCGTTAGACAAGGCGGGTGGGGGGGCATCGCATCTGGAGGT

>Os01t0544200-03 2000 bp upstream sequence

ACTGTTAATCCTGTATATCCGCTACCATCAGGTCCCAACCAAAAACCCTTATGGAAAATCGGAAGTCACTCACATTTTGGCCTGTAAAGCGGAGGAATCCCAATCCCTTTCCCTTAAAAAAAAGAAACACGTTTGCTCTTCCATATAAATATACAGGAAATGCCTTGTAGTGTCGAAAGTTATAACTCAATAAGTTGGTGGCAAATTGGCAACCACAATTTCTTCAAAGTTGGTCCACAAACCCATCCTGAAATTAATAATTAATTGCTCAAGTCGTTGATGATGATGATAGTTTTGGTAGGAGATATATGGATTGTGTTGGTTAATTTAGTGTTCCCACGGTGATATGTCGACTTCTAATAATAAAAAACATCTAGACAAATATATATATATGCTAATATTTGCAGGAACATTATAATAATCGCAAGTAGTACACTTGCATAAATTATATTGCTATATCAGCTAGTAGTTATCATTTGAGTAAATTCCTTGTGTGCCCCTGAAAAGTTATTCAATCTCTTACATGCCCCTGAAACTTACTCGATTCCTTCTATACCCTTGAATTTTTGATTGGATCCCCTACATACCCCTACCGTTAGATTTAGCAGCATTCCCTGGTTAGTTTGGTTGAAAATGTCTATAATACCCTTGAGGTTGGAGGAGCTGAAAACTAAGAACATGTTTGGCAGCTGGGTTTGAAACATTCATGACTGCACAGTATGTATTCCGTGAGACTAAAAAAATAGTGCATAAAATTAATTTCATTTTCTTTTGTTATAGCATTCAGTATTTTATAAAAAAATATAACATGACATTGGATCTTTCATGACCACTAACCGACACAGCCTGGGGGTAAAAGACAGTACCCCTACCCGTTTACTCAAACACCATTGCAATTCATCAGCATATCAAGAGCATCGGTAGCACCATTTAATAGGAAGAAAATGAAAATACATCTAAAAGCATTCATACACCATCAGCAGTACACAAATATATTACTTTTTACATCATGAGCATAATGGCACAAGCCCATAACAGTTCAAGGCATCTGCCTGCATAAAGTCAAACAAATTAATGAAGAAACACTAGCAGCAGCAGCTATTCGCATTTTGCTAATCAACGCAAGGGTGTATCTTACCGACGGCGAGGAGCAGCGGAGCAAGGAGGTAATAGGCCGGTGTCGGAGCCCCCCGAGAAGAGGGTGAGGTGACAAGAGGGAGGGCGCGGAGGAGGCAGGGAGGAGACGGCGTCGCCATGACCGCAAGCAGAGTCGAGGCCGCGGCGGGATGCGGACTCGTCGCCGCGGCAGGATGCAAAGCCGCCGCCGCCCACCAGATGCGGACCCCGCCACGCCACCGATGGGATGCGGACCCAGCCGCCGCCGCCGACCGCAAGCAGAGCCGAGACCGCGGCGGGATGCAGAGCCGCTGCCGCCGCCGGGATGTGGGAGAAGGGCGGCGATGCGCGTGCAACGATGGCTGAAAGCTGGCGATGTGCGGCACCGTGCGTGTGGGAACTATTTTGGAGGATTTGGGGATCGGATGTGGCCCTGTGTTACCTAGGGTTGACAGGGGCATTTTCGCATTTTATGAAAATACTGACAAGCTAACACACACATGAACACAAAATCTGACGGAACCCAAACGGTAGGGGTATGTAGGGGATCCAATCGAAAATTTAGGGGTATAGAAGGAATCGAGTAAGTTTCAGAGGCATGTAAGGGATTGGGTAACTTTTCAGGGGCACACAAGGAATTTACTCTTATCATTTAGATGGAGATAGAGGTTTGTCCAACCATCTTTTTTTTATTATTTTTTAACATGCATCTAAGCATATTAGCTATATACCACATCATTTTCATTATGTAAAAGAAAACCGTACCTTTGAAAAAAAAAACAAACATTTCCATTTTGTCAAGTGATATGATGGTCGTTTCATTTTGTCGTCTAACTGCTCTGCAGGCTATAGAAACCAGCAAAAGCTGGCTAGCTAGATTCGTT

>Os01t0567400-00 2000 bp upstream sequence

TCTCATCTGCCCAGGGCACATGCACTCATCCCCATTGTTGTAGGACCGACAACGCCCACAAGATTGATGGTGCAATTGGCGCCACCCATAGGGAGCTCACCATGGTGTTACAGAGAGAGTTTGAAGAGAGGAGAGGGAAGAGATGGAGGAAGAAGGCTAGAGCGGCAACTTACATATGAGTCATGTATGGGTCTCATTTATATGGGAAAGGAGGAAAAAGACATATAGGTCTACCTATTTTTTGATTTTATAATGTTTTTCTGATTGTTATGCCACGCAAGGATTGCAAGTACAGAATGGAATTTTCAAAAATTTGTTAATTTCGCCCCCACTTGCAAGCACATATCTCGCACGAAAGTTTCTCTATTGTTTTCCATTTTTTGTGAATTTGGTCAAAGTTTTTTCAAATTCATTCAAGATTTGTCAAATATTTTGGCAAAAAATGTATCGAAATTCCTAAAATTTTGGTCATTTCATGAATGTCAGAAACCTCAGCAAAAGAAAAAAAACCCATTTTGAAATTGTAACCCTGATGCCACGTAGTGCTACATAGGCACCACGTCAGAAGAATACTATGTCAAAGTGTAATATAGAACAAAACTGTTGTCCAAACCGCTCGAAGAGGCAAATGAACCGGTTTTCGTCGTTGAGAGATTCAAGTTAGACTTTTTCCGTAAAAAGAAAAAACAAGAGCAAAATAGCACTAAGCAGGGCCAAATGGGCTGTTGGCCCATTCGGGGAGAGGGGACGAAGAGATTAGATAGAGATCAATAATGTCACCTCGGTCAAGTCAATGAGGTTGCTAGCACAGTAGTACCCTCGCTATTAGTCTGCTTAACTAAACTCAACAACCTTTTTTTCCCCTACCGCTACCGCTACCGCTTCTCTGAAACAGTCAAGGTGAGACCGGTGAGAGTGATCAAGGGAACAGTGCTAATTAGCGTGACAGGATCCAATTATAATTTGCTAACTCACGCGGCAATAATGCACGGCGATGACAGGGCCCTTCTGATGCCAACGTGTACACGGTTGGCCATGAGCCGACTGATGAAGTTAAGCTAATTAAATCCAGCCCACAAACACGTAGGACAGCGCAAGTCGATACTTAATCAGCGAACAAATTACTCCCTCCATCTATTTTTTATAGTCATATTTCATCTCGGCACACAGACTAAGTAATTCTACTTATTATCCATTTAAATATGCTACTAGTCATTCCTCATAAACAAACAATTCATTAATATTTACATTTCTCGATGCCCATGTAACCAATCTTGTGTGGAAGAATGGAGAGTCATGCATTAAATCCGAGAAAGTCATTAAGATAATAGGTTGTTGAATTCAAATATGCCTATCAAAAATAAATTTTTCAGATTTAGAAATATTACTATCAAAAGTAGATGGAGGGAGTACTAATTAATTAAACGCGGTTGCTTTCTCGCCATAAATTTGCTCCTCCTTGTCGGCACCGTTAAATGATAGTACGATTTATTAAAAAAAACACTTTATGTGTAAAAAAAATACACTAAAAATTAAATAAATTTATTTTAAAAATTATAATAGCTAGTTAACATCTAACTAATCGTATGCTGATCGAGTGCCTTGTACTAGTATAAGACAAGCTGCTAATCAGCCATTAGCAGCCAAAATAGCACATCAGCTATTAGCTAGCCGAATTGGAGAGAATTCTATTCTCTTTTCTTTTTCCAAGTTTCAACCGCCACAACAATTGGACTATATGTGCTACTAATATTCGGCTATTCCATTCCAGAATTCCACCACGACAGTTGACGAAACGGGGCAACCCGATTCCCACACTAGCCCACAGCGTATAATCACATTTCCCGTTAATTTTTTCCTTCCCAACCACCAAACCCCTCCCATTTTTCTCCTCCCCAACCATCGCAAAAGCTTAAGCTACTTCCTCTCCTCCTCGTCCTCCTCTGCTCGGCGCCAAGAAGGAGAGAGCCGAGAGGGTGTGGATCGAGCAGCTAGCCGCC

>Os01t0588200-01 2000 bp upstream sequence

TCGAGCCGTCGTCTGCCACGTCAGCTTGCCGCCCTCCACTAGGGCGGCAGGGTCTATTTTTTAAATTTTTGCGACGATAGATTATTTCTGTAAATATTTAAAAAAAATTAAAAATGAAAAATTTCGAATTTTTTATATGTTTGTGTGGTGTGGCAAAGGCCGGCCTAAGTCCACCGAGTGATTCTTTCTGCATTTCTGCGCGACCGAAAAACAAGGCCCAATATAACTAAATGGGCCATAGGATATAGCATCCTTCCACACAGATACAGCCCAGGAATAAGTTCACTTAGGTCCCTTCAAAGTGACCTGACCCTCAACCGCAAAACCAAAATTTTGACCCCTAAACTGTTAAAACCGGTGCAATTTGATTTCCTAGGTGGTTTCGTTGACGTGGCGACTACGTGGCGGTGTTAACTTGATCTTCATCCGACGTGGTGTTGACATAACTCTTATATGTCATTAGAAGTGCAAAAATTTATGGGGACCCATTTGTCATTAAAAAAAAGGGCCCAACTAACATGTGGGACCCACATGTCAGCCTCTCTCTTTGACTCCTTCTTCCTCCCTCTCTCCCTCTCTCCTCGGCAGGGGCGTGCCACCGTAGCCGCGGGCGGTGAAGGCAGCCACGGACTCGGCGCTGGCGCTCATGCGAGGGGGCAATCGAGGCGGAGCTGCACCATCCTGCTACGGCGCCAGCATAATGATGATAGGAAGCGGCGCTGAGGAGGCCATGGGCGGCAAGCTTCGTGGCTGGTGAGTTGGGAGGAGGAGGAGGAAGGAGAGCCTGGAGTAGGGCGCAACATCCTCCTCGCCCGCCGTTTCGTTCCACTTTTGCATGGTCGATGGAAGAGGAGCCGGAGGCGGCAACGTTGGCGGAGGGGTCGACGCCGCCTTGGCGAGCAGTAGGTGCGCCAGCCTGCCCGCGTCAGGATGTCACCGCCGTTCCCATCACAGCCGGCCTCGACGCCTATAGAGCTCCCTCGCCAAGCTCTGTCCCGCCGCTGCCCTCCCTCTCTCTCTCTCACCACCCCTCCATTTCTCCCCACAGATCCCCAATCCTACCCCACCCCAATCGACAATGGCGTCATCGGCGGCTCACATCTAGGCCAACACCACACTGCTGTCTCTGTCGCCGGTGGCTCACAGGAGGGGATGAGCCAACCGGAGGAAGATGATAAGGCGGCAACGTACGTAGCAGGTGGGCTTGTCGGTGGATTTGGTCGGCGGAGGCGAAGGTGCCGGCTTCGCATGCGTCAGGCGGGCAAACGGGTAGGTCCCATGTCGCTGCCGCCACTCCGGCCCCCACCTGCCGCCGTCGCTGGCGGCCCCCGCTTGCCGAAGGGGGAGAGAGAGGGAGGAGGAAGAAGGGATAGATTGATATGTGGGCCCCACATGTCAGTGGTCCCATATATTTTTCTTTTGTGTGAATGACGAACGGGTCTCACGTGTATGTTTTCAATTTTAATGCCACCTGTGCGCCATGTCAACGGCATACTAAGTCAACACTGCCATATGGATGCCACGTCAGCGGAACGGCTCTCCAAAACCACCGAGAGAATCAAATTACACTGATATTAACGTTTAAGAGTCAAGATATCTGGTTTGTCGTTCAGGGCTACGGATTAGATTCGGGTCACTTTTAAGGGAGTCAAAATGAACTTATCCCTGCAGCCCGCCTAGGGATCAGATCGCTGTACGGTTCGCCAGGCCCAACCAAGCCCACGGTCGCCCGCAACCACCCTTCCCGACGCACGTGCTTAGAATAAAGACGCAGATGCCCACCACGGCGGCGTCGAATGATGCGGGCCCCACTGACAGCCTGACCCGCCGTTTGCTCCGCAGTCCGCACGAAGCACGACGCGACGACACGGATCATCCCCTCTCCCTCTCACCTCACCCGCGCCCACGCCCAGCCAAAGAAAAAAAAGAAAGAAAAAAAAGAAAAAGCTCATTTCTTTATTTTTAGAGAGAAAAGAAAAGAAAAAGCGAGTCCAGGTCCA

>Os01t0588200-02 2000 bp upstream sequence

GCGGCAGGGTCTATTTTTTAAATTTTTGCGACGATAGATTATTTCTGTAAATATTTAAAAAAAATTAAAAATGAAAAATTTCGAATTTTTTATATGTTTGTGTGGTGTGGCAAAGGCCGGCCTAAGTCCACCGAGTGATTCTTTCTGCATTTCTGCGCGACCGAAAAACAAGGCCCAATATAACTAAATGGGCCATAGGATATAGCATCCTTCCACACAGATACAGCCCAGGAATAAGTTCACTTAGGTCCCTTCAAAGTGACCTGACCCTCAACCGCAAAACCAAAATTTTGACCCCTAAACTGTTAAAACCGGTGCAATTTGATTTCCTAGGTGGTTTCGTTGACGTGGCGACTACGTGGCGGTGTTAACTTGATCTTCATCCGACGTGGTGTTGACATAACTCTTATATGTCATTAGAAGTGCAAAAATTTATGGGGACCCATTTGTCATTAAAAAAAAGGGCCCAACTAACATGTGGGACCCACATGTCAGCCTCTCTCTTTGACTCCTTCTTCCTCCCTCTCTCCCTCTCTCCTCGGCAGGGGCGTGCCACCGTAGCCGCGGGCGGTGAAGGCAGCCACGGACTCGGCGCTGGCGCTCATGCGAGGGGGCAATCGAGGCGGAGCTGCACCATCCTGCTACGGCGCCAGCATAATGATGATAGGAAGCGGCGCTGAGGAGGCCATGGGCGGCAAGCTTCGTGGCTGGTGAGTTGGGAGGAGGAGGAGGAAGGAGAGCCTGGAGTAGGGCGCAACATCCTCCTCGCCCGCCGTTTCGTTCCACTTTTGCATGGTCGATGGAAGAGGAGCCGGAGGCGGCAACGTTGGCGGAGGGGTCGACGCCGCCTTGGCGAGCAGTAGGTGCGCCAGCCTGCCCGCGTCAGGATGTCACCGCCGTTCCCATCACAGCCGGCCTCGACGCCTATAGAGCTCCCTCGCCAAGCTCTGTCCCGCCGCTGCCCTCCCTCTCTCTCTCTCACCACCCCTCCATTTCTCCCCACAGATCCCCAATCCTACCCCACCCCAATCGACAATGGCGTCATCGGCGGCTCACATCTAGGCCAACACCACACTGCTGTCTCTGTCGCCGGTGGCTCACAGGAGGGGATGAGCCAACCGGAGGAAGATGATAAGGCGGCAACGTACGTAGCAGGTGGGCTTGTCGGTGGATTTGGTCGGCGGAGGCGAAGGTGCCGGCTTCGCATGCGTCAGGCGGGCAAACGGGTAGGTCCCATGTCGCTGCCGCCACTCCGGCCCCCACCTGCCGCCGTCGCTGGCGGCCCCCGCTTGCCGAAGGGGGAGAGAGAGGGAGGAGGAAGAAGGGATAGATTGATATGTGGGCCCCACATGTCAGTGGTCCCATATATTTTTCTTTTGTGTGAATGACGAACGGGTCTCACGTGTATGTTTTCAATTTTAATGCCACCTGTGCGCCATGTCAACGGCATACTAAGTCAACACTGCCATATGGATGCCACGTCAGCGGAACGGCTCTCCAAAACCACCGAGAGAATCAAATTACACTGATATTAACGTTTAAGAGTCAAGATATCTGGTTTGTCGTTCAGGGCTACGGATTAGATTCGGGTCACTTTTAAGGGAGTCAAAATGAACTTATCCCTGCAGCCCGCCTAGGGATCAGATCGCTGTACGGTTCGCCAGGCCCAACCAAGCCCACGGTCGCCCGCAACCACCCTTCCCGACGCACGTGCTTAGAATAAAGACGCAGATGCCCACCACGGCGGCGTCGAATGATGCGGGCCCCACTGACAGCCTGACCCGCCGTTTGCTCCGCAGTCCGCACGAAGCACGACGCGACGACACGGATCATCCCCTCTCCCTCTCACCTCACCCGCGCCCACGCCCAGCCAAAGAAAAAAAAGAAAGAAAAAAAAGAAAAAGCTCATTTCTTTATTTTTAGAGAGAAAAGAAAAGAAAAAGCGAGTCCAGGTCCAGGGCGAAGAGGAGGACGCCACGAGATCTGCAGCGAGAGAGAGC

>Os01t0628000-01 2000 bp upstream sequence

TTATTTGGCACTGCACGTGGGAACCTCAATGTACGATATCTAGATAGCACAGCATACATATGGCAAGGCTCATAAATTGACCTCAAAGTTGGCAGTGGTAGACTGGTAGGCAAGCATTCCGTCAAAGACATAGCAAGCATGCATATATTAATGAACCACATTGTTAATTAGCAGGTTTACCTGTTGAAAGCAGGAGATTAAAAGTTTGGGCGTTCGTCAGCGACGTGCGAGGGCATTGGGCATCGGCGTCCCCTGCTCAGTCGTGGAGCAGAGGATGGCGTTGTCCCCTGCTCCCCGCCGCCGCCACCTCCCCTGCCGCTCATCTCGCCCCGGCCGCCGCCGGTGGCGCTTCCCTCCTACGCGACTCGCCGGCCGGGGCCGAGCGGGAGGCGCCGCTGGCGGCGGCGGCGGCGGCGGCGGGGAGGGGCGTCGGGAGGGTGGGGCGCCGGCGAGGAGGTGCGTCGGGAGTCGGCCACTCGGGAGGGTGGGTGCCGCCCGGTGGCGGTTTGGAGCGAGAGGGAAGGGCGCGGCGTGGGATTTGACGACGTGGGCGGGGATTTTGCGACGGGAAGAGGCGTGGGAGCGTTAATCTAATTCTAGTTTGAGTTCTAATTCCTGTGTTAATCCAACTCACGATGTGGATCGTTGGATGAGAGGATCTAACGATTAAGAATAGGTGGGTGTACTAAAAAGAAAACTGGTGCTTTTTATATTAGTCTAGATTATTCTGGTGTATACAAAGTTGGTAAAAGATTGTACAATCCTATTTGTATAGGATAGGCCTTTTTGTCGAGAATACATAAGAATTAGGTTATTAATTGTTTATTTTTGTCGGAAACTATACGAACCTAATACATGCTTAGGTTTTGACAACAAAGATGGCTTAGCTTGTTAAGCCTAACTAAGAAGCCAATTCGATCGGCTTATTAAGTCCAACAAAAAAAAAATCTAATTCAAGCTCAAGAACTTCTTTTATATCTTAAAAGAAGCACGTAAAATTTCATTATTGTTCTTTTATATTTTTAAATGTGTATTGGCCAAAATCGTTTTAGCTTTAAAAAACAGATTATTCTAATAGTTTTTCTTTCATAATTTTCACTTTAAAATACATAGTTTTACACATGTATTTATTTGAGTTATTTTTTTAATTTATATAAGCAGAAATACATTTTTTTTGTCCAAATCAATTTTTCTATTATATAATTAATATTTTTTAAAATGTAATTATTCATACGGAATAATAGTTACCAACTTGTATGTGAAAAAAGAGTTTTCAAAAAATATAGTATATTCTGATTCAATTATAATTTCCCCATCGATCATTTTATTTTTTTTCTTATAAATAAATATACCGTCGTAATTGGACTGAGTTCCCATTAATTGAAGTTTCCATTTCTGTTATTTTTTATTTCAAATTTCATTCTTGTGGTGCTATGGACTTTAGTTCTGATACAAATCTTCTTTTCAACTCATAAATATTTTTTTTCTGAATTTCAACATTTCTGAGGTGTGTAAATGAGTTCTTTTATTTTATTAGTAGTCACAGGCTCACAGCACCTCATGGTCGTCCCGATCGTGGCATTTCTGGGTTGGAATTTCTTCTCCACCAAGTTCATTTGGGCATAGCTGGCCCAAATGTTCGTTAGTCTTGTTTACACTCCAAGAAACCATAAACAAATTAGCACAGGAGACTAATGCAACCCAGTGACAACTCTATATAACATGGCGCGAAATCCGTACCTCATCCATCCCCACTTCTCCGGTCAAATTAAGTACTAGTCCAGTTGCATCAGCTTCTTGCTATTCAGAGTCCACAGCAATGGTTTTTGGAGAGTTGTTCAGCCGAGCCTCACTGCCACCACCATGGAGCCTCCTGGCGTACGGCCTCGTCGGGCCGGTGCTTCTGTGGCAGGCCGGCCGCCTGCTCGACCGGCTGTGGTGGCGGCCGCGGCGGCTGGAGCGCGCGCTGCGCGCGCAGGGGCTCCGCGGCACCGCGTACC

>Os01t0667600-01 2000 bp upstream sequence

TGACGTGTCGAAGGGAACGCATGTGTTCCCACATGTCAATGCCCAACTGTTCTTCCTCCTCTCCCCACCTCTCTTCCTCACTCTTCCCACCTTCTTTTGCAAGGCGGTGCACAACAGGTGGTGTGTAGAGGGCGCGCGGACAATGAGGAGTGAGGCGGCGGCTGCCGGCGGGAGGCCGGACGAAGGAGGAGCAGCAAGAGAGCGGATGAGACCAAGAGGAGCGAGGGGGGGTTGAGGATGCCGACAGAGGAGTGGAGGTCGCCAACATCTTCAAGGTTGTTTCCCTCGCCGATGAGCCACCATTTGTTCTCGGTGAAGATTTGCAACAGGATCCCCACGCACTTCTCCGTCTGCCGGAACCTCGCGCGTGCTCCTGCTTACTAGCATCACTGCCACATCGCCCTGGAAACAGCCTCTTCCTCTTCCCAACCTTGCCCCACCCCATCATTCCATTCGCCAATCACGCCAGTGTTGTCAAACTCGAGCTTGATGGTTTCGTCCACTCGTGGCCGCCATCTTATCGCTTCCCCCACGGTCACTGCTATTGAGGTCCATCAAGGTAGGAGGTTGAAGAGGATGGCGCCCTGCCCTAGCGGTCACCGCCACCACTGCCGACCTGTGCGGTTGCCTTGCTCCAACTGCTCCTCCTCCACCCGACCCCCACCGCTCCTTCTTTCCCCTCTCAGTATCCTTCGCTCCTCCCTCATTCTTCTCAGCCTCATCCGCTCTCTCGCCGCTCCTCCTAAGCCCGGCCTCCTGCTTGTCACCGCTCCTCTCCGCGCCTCCCACTGCGCTCTCCATGCTGCCCAATGCGCATCGCCCTAAAGATGGGAAGAGTGTCAGAGTGAGGAAGCGAGGTGGGAGACAGGGGCGGAGCTAGAGCGAAATGGAGGGTGGTGCCACTTGCTTAATTTACTCTACTTTATATCGAGTTTAAACTGATGCGGGTACCTAAGTTTGTATTGAGGGGGTGCATACATTGTGAAAATAGATGAAGTATAGCTAAAATTTTTTTTTGACCGGGTTCCTAGGCACCCCCCTAATATATACTAGCTCCGCCCCTGGTGGGAGAGGAGGGAGAAGATGTGGCCGATGACATGTAGGTCCCACTTACCACGTTAGTGAAAACAGTCCACTATACTACCGATGAACTAAAGTTATATTGTGAGTTGGGGGATGGGCAATACCCGGTATTTCTATCCAGGGACGAATTTCAGACTCAGTGACAACTGAAGGGATCTAAAGTGAACTTATTGCTTTTGTGATGGTTTCAGCCCATCGGTAGCGGCTTCGGTTGAGAGACACGGACGCAGTAACCGGCAAGCACCTCGCTGAGGCCCAGCTCGCTTTCCACAATTACTCGTACATCGACATCGGTACTCCTTGGGAAGAGCAGCTACATGTCGAACCCGTTGTTTTATGGACTTGGGAGTGATGCCTGATGCGGCGTTTGCTCAACCTTGTGGAGGATTTACGTGATCCTCTTTTCCTCAGAGGCTCAGACGTCAGTGTTCACGCTGTTACGTACGCCCGCGGTCGTCACGACCGCAGCGAGCGCAGCCATCTCCCCCCCTCAAAAATATTCAGAAATGTGTGCACCGATAGACACACCGGACCCTTGCTCGTCATTCGCTTGCGAACTGAAAACCAAACGACCGGTCCGGGTGACACATGCGTTTTGCCCCGGTCAGCAAGACGAAACCAAGACCCGGACAACGCCACCAAAACCGTCTCACGTCAAAATCGCCACCGGCTCACGGCTCGCCCGCTCTCGTTCTCCACCTTATCACCCAACCCGTCGTGCCAATCCACACACACACATCGTTGCAGCCTCGCACCGCCCGATAATAATACGACCAAACCAAAGACAGAGCAGCGCGACGCGGGGGGCAACAACCCGCAGCTAACCGCGGCGACCTCGTCCGCACCGCGCCACGCGACCACACTCCACACGCCTTTCTTTTCCTTTTTCCCCCAAAAATCCAAAGCAACCTCGCCCG

>Os01t0667900-01 2000 bp upstream sequence

AGTCAGTAGTGCCAAAAAATCTACTGTCACCTCCTATACGCCACCGCACTGCACGAAAGAGTTCGCAGCTAAGCTAGGCCCAAATGGCGATCTAGCAGAATTTAGACCTTTCTCTTAGTTCTTGACATTTTTAAAGCTTCTCCTGATGCGTATTGTTTTCTCCAAACCAAAATGACGGGGCCAAGAAAAGCAACACATCGTCATTCGTGATCCACATGCTGCTTACCGCCACGCAAGAGCGGTATAGCGGACAAACAGTAGTACTACCTCTGTTTTTTAATAGATGACGCCGTTGACTTTTTCTCACATGTTTAACCATTTGTCTTATTTAAAAATTTTATGCAAATGTATAAGATATAAATCACACTTAAAGCATTATGAGTGATATAACAACTCATAACAAAATAAATTATAATTACGTAATTTTTTTTGAATAAGACGAATAGTTAAACATGTGAGAAAAAGTCAACGACGTCATCCATTAAAAAACGGAGGGAGTATATATTATATACCTACTGATGGGATTGGCGGCAATACATTATGCATGCGGATATACACACATAATCTGACCATCTCTTCCTGGATGTACTCAGCCATCGATTAGACCCACAGCTCGACATTAGTTTATGAGACGGGAAGTATGAATGAAAGACCAATTAAACGTCTCTCGTGGAGTCATGGAAGAACAAACAAGAGTACAAAAGGAGTACAAATACCATATATAAGAGAGAGTAGTAACAAAGCTAAGCTAGCTAATACCCCTCTGTTTCCTGTTTCCGGTTATAAGACAATATATTATAAAAATTTATTCAATTATAGGTTTAATGAAACTATGCATGTGTTATAAATATTACTTTTTTTCTACAAAATCAGTCGAACTTAAAACAGTTTGACTTTAATTAAAGTCAGAACGTCTTACAACCTAAAACGGGGTGAGTAATAATTTAGAAAACGGGAGCTTAGTACTCCGTAATATATTATGCAGGAGCGAGTAGTCCACCAACTGTCCAAGTCCAGACGATCGAGTGACGTGTTGCATTACGTCACTGAGCGCATGTCTGCGTCGCCGATGGCGCGACGCCGTCTACGGCCGCCTGGCCGGAGCTATTATTTGGCGTGTTCTATTACGCCTCACAGTACTTGAAACGTTTTTTTTTCCCTGTATAAATCACTTCAATCAAATATTGTTATACTGCTCCCTGCAGGTCTTCCCTACCAATGTTGGATGATAACCCGAATGTACCTATCAGCCAAACCCCATCCCTAACTCCATGGCTTATCTATGCCGACACTTTGCTGGAGACGACACCACACCTGGCGGGTCCCCCTTGTCATCGTCATCCGATTGTGCCTTCCCTGATTTTCTTACAAGGGCACGAAAGTACATGATCACACCGTTGCAGTTTAAAACGTGTACTAGTATTAAGTTTGCAAGCAAGATCTGTACAGCCACGCACTTCTTATTTCTCAACAAAAAGTCTTCGATTAAATTTCCGACGGAGCGAGTACAGATAGCACATCATACGTACAGCCGCCGACGACGACGAAAAAAACCAAACACATGCACCGAGACATTCCAAGACAAACGATCTGATCAATAATCCCGCTGCTGCGAGAGTCAGCGGTCGACCGTGTCCGCTGGTCGCGGCCCCGCAGCCCATCATCACCGGACCGATCCATTCCATCCATCCACCTCCCCCCCAACGCAACGCTCTCGCGTCCGCGTCGCGGCAGCTGTCGTCACGGATCCGGTGTCCCCGGCCGCGGCTGACGCCCCTCCACGCCATCAGCCCATCACCCCCCGCCCGCGACTCGTGCGCAGTCCAAGCGAAAGCTACCCCACCACCCCCCCCCCCCCCCGGCGTCGCGCGGGCCCCACCATACTACTCCGCGGGCCCCCACGGTGACGTCACCCGCCCACCTCACCTCCGGGGCTCCGACCCCCCCTGCTTCTTCCGTGTGTGTATATATCCACCCTCCACCTCTCGTCTCCCCGAGCC

>Os01t0735300-01 2000 bp upstream sequence

CAAATATTAAGTTGTTAAGTTTTTTCTTTTAACTAAAACATGTCTATTTGCTAAGTTATTGCATTTATAGTAAAATTCTTAATTTAATAGATTACCCCTATCTACTACTCGTGTCCATATTCATCTCTATAGTTGGATTTATCTTGGAAGGAATACATTGTTGTAACGTTAGGTGCAATTTAATTAAAAAATATTTGCGGGTAATCCTGAGTTAATGATAGTTAAAGTGTAATAACATGATGTCGACACCGATGCTGCTGCCATTGGCGTATGTTTCGATTCTCCTTGTCTCTACTACCGTAAAGGACTAAGGGCGACATCCCCAGTTCTCCATGTCATCTAGTGTCCATAACATTAAATACATTGTTACAATGGCAAGAAAGTTAATGAGAAGATGGGTAAAAAAAAAACAAGAAGAAACCATATCTACACAAGAATTAAGAAAGAAAATAAAGGAATAAATAGATAAAAGAAGAGAGAAAAGAGGTAACTAGATGAAAATTAATTTGAAAACACTATCCATTGGATATATAATTTCTACACATAATATCTATGTGACATGGCAAATATGAAAACTAATTGATAGTTTCTGGTTGGAGATATCGTAAAACTGGAGTCCACCGCTATTTCGATTTCCTCCATCTCGATTTAACAACGGTGGACAAAGGACACTTGTTAATTATCACGTGTCAAAGACTGCACCACAAGTCCATAACTGCCTCATTTCATGTAGCACAGGCATGCATGTTTCTCCCCTCTACCATGTCGTCCTCCAGACAGCGATACAATCACGCGGCCGCGTCATAATAGTCATTTCCCGGCCTTAATTCACGTACCAGCACTACTAAAAAAGTATTTTTTTTCTAACGTAGGAGTCTTTTTTTCGCAGGCGGACATGAGCCTGGGAGTCAAATGACCGCCAACAAAAATATAAATCGGGGTGAAATTCGTTGTCCGTCTGCGAAAATCCTGCAAAAATCGTACCTACCGAAAAAAAACTTTGATCCTTATCTATTGCTCCAACCCCCTCCCCACCACTCATTTCTATCTTCTCAACTCTCTCTCCCTCCACCACCGGCTTTTCAGCACCCCATCCCCTCCCCTCCCTCCCTCCACCGGCGGCGGAGGCGGCCGCGGACGCGGCGGCGACCCGGTGTGGCGACGGCGGCGGCTCCCTGGCCTCCCCTCCCAGATCCGGCCGGAGGGAAGCCGGGGGAGGGCGGCTACGGCTGCGGCTACGTGGTTCCCCGGCCTCCCCTCCCAGATCCGGCCGGAGGGAGGCCGCGGGAGGGCGGCGGCGGTGGCGCATGCGGACGCGGCGGCGACCCGGTGCGGTGGCGGCGGCTCCCCGGCCTCCCCTCCCAGATCCGGCCGGAGGTAGGCCGCGGGAGGGCGGCGGCGGTGGCGCGTGCGGACGCGGCGGCGCCCCGGTGCGGCGGCAGCGGCTCCCCGGCCTCCCTCCTCCCTCCCAGATCCGGCCGAAGGTAGGCCGCGGGAGGGCGGCGGCGGTGGCGCGTGCGGACGCGGCGGCGCCCCGGTGCGGCGGCAGCGGCTCCCCGGCCTCCCTCCTCCCTCCCAGATCCGGCCGAAGGGAGGCCGGGGGAGGGCAGCGGCGGCGGCTGCAACGTGGGCGTCTCCCCTCCTGCGGCGGCCCTCTCCCGGTGCGGCGGCGGCGGCTCCCCGGCATCCCCTCCCAGATCCGGCCGGAGGGAGGCCGGGGGAGGCGATTTTTTTTATTGGATTTTCGCAAGCGGGCCATTCGCCCGCTTGCAAAAATGGATGATTTTCACTGTCGTCAGTCTGCAGGCGGTCCTCTCCTCCGCCTATAAAAATTGCTTTTGACCGTCTGAAAAAATGTTTTTTCTTTTAGTGCAGGGTAGGTCATTTCCTAGGCCGCCAGTCGGTCTTGATGGCAGATTGCCAGGTTAGGTAACGTTTGCGGTTCGGAGTGAAGCACATGAGACTACAAGAGGAAACACACTACGCTATCGTTAAGCAGC

>Os01t0747400-01 2000 bp upstream sequence

TGCGACTAATAGAGGGGCAAATGGCAATGAGAGAGAGGAGATAAGGTGGCGGCGGTGGTGGGCGGTATGGACCAGCCACCAGATGAGAAGTACAGAGGGCACTAGGCGGTAGGCCACCTTGGCTCCCTACTATGGCAGTTGGCCAGCCGCCATATCTAAGCAACGAGCGGCGGCGGCCAAGCAAAGAGGGTGGCCTAAGGAGGTAGAAGAGGCACCCCGACCGTGCGCGAGGCTACAACCTTGTGCCACGCTGCACCATACTTGACCCGTACCATGATCTCCAGCGGCCATGGCCTCAGCGAGATCCCCATCCCTCCTCCGCTACCGACACTGACACCCTCCTCCCGCGTTTGCACGACCATCTCGCTCGGACACACTTCCTACCCAACCATATCTTCCTTGGCCACCGATATCGTGTGAAAAAATGCATAGAAAAGGGTAGAGAGGGATAAAAGAGGATGGGATATTGGGATGTGTCACTGACGTGTGTGATCTACCAATTTTTAATTTTTGTTTCAGACTGAAATGCCCTGATATGTGGGTTTCACATTGACTTAGTCGCCACGTAAGCCGAAAAAAGGCATTATACTATCGCAGAACCTCGGGTACATCAGTTTTACAAGTTGAGAGACGTGTTATATCTGGTTTTGCAGTTCAAACTGTGTGGTTCAAGGCCATGATTCAAACTCAACGGTAGGATGAGGGACCTAAATTGAACTTATTGCTTTTACTTATCCCCTGTCATTTATCAGGCCCGTTGGGCCATTGGAATCCTTATACTATTTGGTCGACAGAGCAAATGGTAATGTCTTTTTCATAAAACGAACTGTTTCGTCCAATTCAATGGTCATTATCCGACCAGTCGACGGTCAGTGCCTACCCCATGTGCACGATCGAGAGCGATTGGGTTAACGTGAGAAGCTGCACGGCACGGCGCAGCGGGTGTGGCCAAAGTGAGTTATTAAGCCAATAAAACACGGTCTAATTTTTTTTGAGAGGAAACACGGTCTAATCTTAGTTGGTAGTACTATTAGAGTATTATTATAGTTTTGTCTATTACTCCGTATAAGTCATTACTTAATGTCCATGCATTTTTTTTCCTCCATCCCTCTCTTCTAATATGGCATTAAAAAAGGGGCAGTTGGTTACTTCGTCTACTATCCTGATCCACGTGTACAAATTTGAGATGACCGTTTGTTTTGTAAACGGTTTTTATATATCTCCTCATAGATTCTGCTGACGTGGACAACCTAAGCAAGCCAATATCTTGTCCCACGGATAAAACCTCCTCACTCGTCAGGCTCCTACCGAACTCGTGCCGAAATAACCTACACCAAGAAAAACTCGACGCGACGTGGCAGGACGACTCACGATGAGCCGAACCCCAAAGTGCCAAACACATGTAACCTGTACACCTTAGCTCAGAGATCTCCAAAATCTGCCTCCCAGAAGCGATCCTATAGGCCGGGTCGGGGACAAAGCTTCAGAATAGCGTGGGGAAACAGACCACGGCGGACCATGCCACGCTACGCCGACACCGTAGTCCGTAAATACCCCCTCCCTTTACACGAGATCCACATGGTCGTGGGACACAAACTATGCGAACGAATCGATCAAGCACCCTTCTTTAATCAAAAGTTTTTTTTTAAACACCCGTCTCGATCTCAAGTCTACCCCTCTTTCGTCTTTCCCGATCCAAACAACTAGTAGTTTTGTACTAATCCAAAGTCGTCTCCAATAATTATCCATTATAAAAGGAACGCAACACACGCACTCCAGGTAAACCCTACGACTCCAGCTAATAAACGCAAAGGAGGAAATAAAGCGAACGGGAGCGACAAGGACGGGGAAGATTCGTGCCCCGATTTTTTCGTGTTCGAGACGCGCAGGGAGGGGGGAAGAGATTGAGGTGGTGGGAGGATCGGAATAGGATCTGGTGCCCCCCTCTCGTCGTGGTTGCGCTCTCATCAACTCGACTCGACCGGTAATCGATCGCGACT

>Os01t0768333-00 2000 bp upstream sequence

GTCCTTGTCGGCCAGCTTCTGGCGGAGCCGCGCGGCCTCCAGCTCGAGGCGCGACGCCCGGGCCGCCACGGCCACCGCCGTGATCTTGCGCGCCACCTCCAGCTGCTCGTACGGGTCCGGCGGCAGCGCGGCCGCGATGGCGTCGGGCAGCCCCGCCTCCGACACCGCCACCGCCGGCGCCGGCGCGGCCGCAGCGGGGGTGGGAGTCTGCGTCATCGATCTCGGCCTCGGGCCGGGCTACCGCGCGCGGCGGAGGCTGGTGGCGAGATCCGAAGCGGGGAGCAGAAGGCTCTCGGGGCTCGCACGGGGAAGGAAGGAAGGAATGTGGATTGTGGAGTGGAGTGGGCTTTGCTTTGCTGAGCATGTGGCATGCAGGTAGAATTCAGTTCCAAGCGAACAAGGATTTTCTACACCTAATTACCGTGGCAACTATTATTTGCATGCGACTCAAATAGTTATAGAAAAAATTAAAAAAATTTAGTAACATCGTTTGGGATGATACAAGTCTATACACAAACATGCAGGACTAAATTTGATCTACAACTAGAGAAACAAAAATAATAAAATCAGACAGTGAATAGTACCCATACAGTGCAGATAAAATTTATTATTTTTGTTTCTTCATATATCGATCAAATTTAGACTTGCATGTTTACATAGTGTCTTGTATTATCATGTACTATCTTATCAAATTTTATGAAATATTTATATAACTATTTAAATTGTATGCATACACCTAGGTGCACCTAGGTGTAAAAAATACTTCTTCTTCTAATGCGCGAGGTGTTGGGAACGGGGCAGCTGCACGTGTGGCGAATGGATTCTGATCATCTTCTTGATTTTTTTTATGTAAATTTGATGTTCCTTTTATTATGGCAGAGTCATGAACTAGCACACAATATATCAGTCGTTTTGTGCATGTCATTTAAAAAGTTATAAAAAATTTAAATTTTTTTTGTCAAGATAGATCAATATATGACATATCTTTTTTAAACATGTGAGTTAAAATTCGACTTATACAATTCGAAACAAAAATAATAAATTTGACCGTGAATTACGCGTTCTATTCATAGTCAAATTTGTTTATTTTGTTTCTACTTGTATAAGTTGAATATGAATTTGCATGTTTGTGAAGTGACATATCACATATTGATCTATCATACTAAATTCTTTAAAAAATTTTGATGACTTTTTATGTAACATGCAAAATGAGAAGATATCTCCTTAAGAGATTATTTGAGTTTCACCAAATTGAGCGCTTTTTTTCAAGTGAAAGAACCAATGTGAGCTTTCAAGGGATCTCGAGAGGCAAGAGCATGAATCCATTCACCCTAACTGACAAAGAATACCATGTCCGCCTTTGCCAGAGAACAATGCCCACCGTTTATTATCAGTTTAAGATTTCGGAGAACTAGTCATGCACCAAACTCAATATAAAATGTTGTTTTACCAGGTCGTAGGCTCAGGTACGCAATGACAATGTGGACGCAGTTCGCTTGTTTTGCCCTTTCGTCAACTCAGCTCAGCTCGACAGTCGGCAATACCACTGCCCAATCATTTTCACTCGCAGATAATAATCTCAACCTAAGCACTGTTCGATCCCCCGAAAATACTCTCCACGTTAAACACGCTCACCGGAAGTCAAAGACCAGCCGCTCTGCCCCCCGGGTCCCACGCCGCGGCGACTGACTCCATGTCGCGACTAATCACACGTCAGGATCGAGCTCACTCACAGTCACAGGAAGTGTCAAAGTCCATGGTAGGAACGTGATTAAATTTCTCACCCTCACTTCGGTCAGTTGGTCACGGTGCGCGTAGGCGTCGTACGTGCTCGTTGCAAATCTTCAAGCTAAAGCTCTCCCCCTCGATCCCTTCCCTCCCTTTTCCTTGTCGCGGGAGCAATTCAACGAGAGAAATCGTATTACAGCTGGGTGAGCCAGTGAGACTCACTACCGGGTCGTCAGCCGCAGCGAGTTCACGCGGCTCGGGCGGCTGGTTAA

>Os01t0798500-01 2000 bp upstream sequence

AGGTTCGACGGGCGGCCGGCGATGCGGCGGCGACGGCGACGGCGGCGCCGCGCCGCCGTAGTAGGAGCCGGGGAGCGGTGGCGGCGTGCAGCGCCGGAGCCGCGGCGGGGCGTCGGGGATGCGGCTCGGCGTCGTCGTCGGACGGGAAAGGGATGTGCCCACCACCACCATCAGAGTAGCCCTCGTCGTCGTTGTCGTCCGAGGGAAAATGTGTGTGGCAGTCGCCGCCGTCGTCGTCAACCTGTTTGATGGCCGCCGCGTCGACGGGGAGAGGAACCACGGCGGCGGGGAGCGAGTGGACCCCCTTCCTTAGGTTGGGGAGGCCCAGCGGTGCAGCGGGGTCGGACGACGGCAGCGCCTAGACGTCGCGGAGGAAGTCGTGCAGCGCTGCGGCGGCATCGCGCAGCGAGGAGGAAAAAGTACAGCCTGCGCCCGCGCGCCGCTCCTTCCCCCGCCACGGGCAGGCAGAGCAGTGCAAAATTCCAATAGAAGAAACCAAACGGAGGCAGCAAAAAGACAGATCAAATGGATTCACCAACTGATCCATTCATCTGCCAGGCCAGCTGGGCTTGGCATATACATTGCAGACCACTAGAACTATAAGAACCTTATCATTGTCATTTGCTAAACAGAAAAACTACTATCTGCATATCTTCAATTCCATGTCTCCAACTCCAAGAACCAGTTTTGATCCCAACAGGAGTGCAGGAATGGAGGTAGCAGAATGCAGTGAGAAGCTGCAATTTTTGAAGGCATCAGCTTCAGCATCAATGGCTTATTCGATTGTTCAGTTCCCAGTCAAGTGGCAATCCATCAAGTACAAGTTGCAGAAACTCTGTTCCAACTTCCAACCTGAAATCCTGAATGTGCCAGGAGACGATGACAGCTGCAATGAAATGTGATCTTTGTTCAGTTTCTTCAGACGGCTATGGCAACAGTAAGTCATATTCAAGCTATTGCTTCTCAATGCAGCGATGAATCTTACAATGGTGGAAGGCTTCCCCCGCCACGCGCGCGCCGCTCCTCCCCCGGCGCCCGCACCCACTGCCCGTGCCCGGCTGCTCCCCCATCGGCCGCCGCCCGCACCCAACTGCTCCACCTTGACGCCACGGCTCGCTCGACCCCGCCGCGGTGCGGTGCGCTGCTGCCTCGTCTCCGCTGCCGCGTCGCCCGTCACCGGCCGCCGACGTTCTCCTCATGTCCCGCCTCGCACTGCCAGCCTCCTCTCCACTCGTTGAAGTCACCGGCCTCCTCCTCCTTCGCCGCTTGCTCCTACCCCTCGGCGGCTAGAGGGGCGGAGACTTCGCCGCTGTCGCCAACGGGGGCGCAGTACAGCCGCCGCCGTGCCGAGGCCGCTCCCCGTCGAGGGAGGAGGGAGACTCGGGTGGCGGAGCGGCGTTGGGCCTTGGCTTCGTGGCGCAACCTGCACAAGAGAGGAGCTGACATGGACAAGCTGACATGTGGGGGCCACGTGGATCCCGCGCTGACTCAGCAGCAACGTCGGACAAAACCGGTTTCAAAACCACCGAGGGACCATTGGTATTGGTTTTGTAAGTCTGGGGACGTGTTATATCCGGTTTTGGGACGATTTTGTAACTCGATGACAAGTTGAGGGACCTTCGGTGTACTTTTTCCAAAGTGTAAACGGCCTGGAAATGGGAGGATGGGCTGGAGCCCATGGGAGCTCAGTTCCAAATGGCGCTAGGGCTAGTAGGCTCTGATCGGTAGGCCGAGTTGGGCTAGTAGGCTGTGATCGGTCGGCCGAGTTGGGCAAAAACAGCATAGTCCGGCCTGTTTGCTGAGAACGGGCTGTCATGAACAAAAAGGGCCAAGCCCATGCGAATATATCTCGAAGTACCAAAGAGACAAGGGCTTTGTGTACTGCACGACTGTTTACACAGCTGCAACGGCGCGCTCACTGCCGCCTGGGGCCCGCAAGGGGAGCCGAGAGGTGCCGCACCGTCGTCATCCTCGCAAACCGTGCGGCCGCGGGCGAGCGG

>Os01t0798500-02 2000 bp upstream sequence

ACGGGCGGCCGGCGATGCGGCGGCGACGGCGACGGCGGCGCCGCGCCGCCGTAGTAGGAGCCGGGGAGCGGTGGCGGCGTGCAGCGCCGGAGCCGCGGCGGGGCGTCGGGGATGCGGCTCGGCGTCGTCGTCGGACGGGAAAGGGATGTGCCCACCACCACCATCAGAGTAGCCCTCGTCGTCGTTGTCGTCCGAGGGAAAATGTGTGTGGCAGTCGCCGCCGTCGTCGTCAACCTGTTTGATGGCCGCCGCGTCGACGGGGAGAGGAACCACGGCGGCGGGGAGCGAGTGGACCCCCTTCCTTAGGTTGGGGAGGCCCAGCGGTGCAGCGGGGTCGGACGACGGCAGCGCCTAGACGTCGCGGAGGAAGTCGTGCAGCGCTGCGGCGGCATCGCGCAGCGAGGAGGAAAAAGTACAGCCTGCGCCCGCGCGCCGCTCCTTCCCCCGCCACGGGCAGGCAGAGCAGTGCAAAATTCCAATAGAAGAAACCAAACGGAGGCAGCAAAAAGACAGATCAAATGGATTCACCAACTGATCCATTCATCTGCCAGGCCAGCTGGGCTTGGCATATACATTGCAGACCACTAGAACTATAAGAACCTTATCATTGTCATTTGCTAAACAGAAAAACTACTATCTGCATATCTTCAATTCCATGTCTCCAACTCCAAGAACCAGTTTTGATCCCAACAGGAGTGCAGGAATGGAGGTAGCAGAATGCAGTGAGAAGCTGCAATTTTTGAAGGCATCAGCTTCAGCATCAATGGCTTATTCGATTGTTCAGTTCCCAGTCAAGTGGCAATCCATCAAGTACAAGTTGCAGAAACTCTGTTCCAACTTCCAACCTGAAATCCTGAATGTGCCAGGAGACGATGACAGCTGCAATGAAATGTGATCTTTGTTCAGTTTCTTCAGACGGCTATGGCAACAGTAAGTCATATTCAAGCTATTGCTTCTCAATGCAGCGATGAATCTTACAATGGTGGAAGGCTTCCCCCGCCACGCGCGCGCCGCTCCTCCCCCGGCGCCCGCACCCACTGCCCGTGCCCGGCTGCTCCCCCATCGGCCGCCGCCCGCACCCAACTGCTCCACCTTGACGCCACGGCTCGCTCGACCCCGCCGCGGTGCGGTGCGCTGCTGCCTCGTCTCCGCTGCCGCGTCGCCCGTCACCGGCCGCCGACGTTCTCCTCATGTCCCGCCTCGCACTGCCAGCCTCCTCTCCACTCGTTGAAGTCACCGGCCTCCTCCTCCTTCGCCGCTTGCTCCTACCCCTCGGCGGCTAGAGGGGCGGAGACTTCGCCGCTGTCGCCAACGGGGGCGCAGTACAGCCGCCGCCGTGCCGAGGCCGCTCCCCGTCGAGGGAGGAGGGAGACTCGGGTGGCGGAGCGGCGTTGGGCCTTGGCTTCGTGGCGCAACCTGCACAAGAGAGGAGCTGACATGGACAAGCTGACATGTGGGGGCCACGTGGATCCCGCGCTGACTCAGCAGCAACGTCGGACAAAACCGGTTTCAAAACCACCGAGGGACCATTGGTATTGGTTTTGTAAGTCTGGGGACGTGTTATATCCGGTTTTGGGACGATTTTGTAACTCGATGACAAGTTGAGGGACCTTCGGTGTACTTTTTCCAAAGTGTAAACGGCCTGGAAATGGGAGGATGGGCTGGAGCCCATGGGAGCTCAGTTCCAAATGGCGCTAGGGCTAGTAGGCTCTGATCGGTAGGCCGAGTTGGGCTAGTAGGCTGTGATCGGTCGGCCGAGTTGGGCAAAAACAGCATAGTCCGGCCTGTTTGCTGAGAACGGGCTGTCATGAACAAAAAGGGCCAAGCCCATGCGAATATATCTCGAAGTACCAAAGAGACAAGGGCTTTGTGTACTGCACGACTGTTTACACAGCTGCAACGGCGCGCTCACTGCCGCCTGGGGCCCGCAAGGGGAGCCGAGAGGTGCCGCACCGTCGTCATCCTCGCAAACCGTGCGGCCGCGGGCGAGCGGAGATGCC

>Os01t0805600-01 2000 bp upstream sequence

CATTTTCAGTAGTACTACGGCATAGGAGTATGCACTAATGTACTCCGTATCCTCCCCGAGACGTCTGTCCACTGTCATAGTCACATGCAAGTCAATTCAAGTCTTGAGATACACTAATCTTCAATCCAAAGCTAGCTGTACTGGCCTTTTGTCAGTGCACAATTTGGACCACATGGACCACATATTCACATGCCTCTCTCAAGCTCTCAGCAAAATTCTCAGCAGTGTACAGTATACTCTTGTATGCGGTGTACTTCCTCGTGGGTGTAAATAAGGCTTTTGCTTAATTCCATCAGAAATTGCTGCTCCACTCTTCTCGCTCAAAGTGTTCTCTCTTTTATTTTGGAGATGCTTTTCTTGCCCAAAGTTGAGCTCAAGCTCACTTTCATCTCCTGGGAAGTTTGCACAGTGCCCTGTATGACACATGCTCGCTCAAATCAACCTTTTTCTCTTATGATTTTTCATCGTCAGTAAATTCCAAGCAGGTCTGTATATGTTAATAAAAAGAAAACTCGAATGAAAATGAAACTTCCAGCCTACTACGTACGAGTACATAGTATTGTGTTGTTAATGGCAACTCCACTAAAATGGAAGGCTTTTCCAGCTCGATGGCTCAATCCATACCCATACCTACACCCAACGGCTAGTTTCGCGTGGCAGCCGCGGCCAATCATAGCCGTCCGATGCTCCGCTTCCTGACCGTTGCGATTCGAAGCCCTCCCGACCGTTGGAGCGGCCGCTCCCCCTCCGCTCCACGTAAAAAAGGCAACGCCACCCGACACAGAGGACGCGCCCTGCCCTAGCTCGCTATATCTCGCCCCTCCTCCACGCGCCGCCTCCAAGAACTGTATACCACCACGCATCAATCACACGCCGCACGCCGACGCCGATTGGTTGATTTGATTGATTGATTGGGCAAGAGGAGGAGGAGGAAGGGGATTGTTTGCTGCTGCTGCTGCTCCAGAGCTCGCCGGAGCCGCGGTGGCTGCAGCGCTCGACGGCGGGGAAGAGGGGTCGCGGGCGGCGCGGCTGCACTCGTTCTTCGCCTCGGTGATCTCCGGGATCTTCGGCCAGGGCGAGGAGGAGGAGGAAGGGGAGATGGCGACACGCAGCCAGAACGTCGCTGCCGCTCCGCAGCCGCCGCAGAACAGAGGTGAGCGAAAACAGCGTGTCTCAGATCTTTGGGGCTTTGGCGGCTGCGCGGATTCGAGTTCTTGTCGTGTTCCTCGTTAGTTTCCTGTTCGGATCCTGTCCCAAGATTGGGTTTTTTTTTTTTACACGAGTTGCCTTATCTTTGGGTAGGCTTCTCTTCCCCCCAAACTTGGTACTCATCGGTTCATTGTCCAGCTCCAGCAGTTGATCTTTGTTCTTGTTCTTATTATTTCATTTACCTGCTTTTTGATTGGATTTCTGAAGAAAAGATTCCAATTTGATCCCTATTTGGTGGTGTTGTTCAGTCCGACTTCTCAAAATCGCGAAATTCTCATAGCGTCTGTCTCCTGCTCAATTTCAGGCAATGTTGCCGCTCTGGGAAAGCAGAAGGCCGTCGTGGCCGGCAGGCCCGACGCGAAGAACCGGCGAGCCCTTGGCGAGATCGGCAACGTCATGAACGTCCGCCTACCGGAAGGGTACGTGATACGACCAATCGCTTAGCCTCACGCTGTCATAATTTCTTCTGCTCTTTATCTGAAACCTTCACTTGTGTTCATGTAAACAGCAAGCCTCTGCAGCAGGCGCCGGCCGGCCGCACCGCCAACTTTGGTGCCCAACTGCTGAAGAACGCGCAGGCAAATGCTGCGGCAAACAAGGTACATCGGCCAACCAAATCCTTCTTTCATTCTTTGTCTTGATTCTTGAAGCATCTGAATCTGAACCTTACTGAAACTATTGGGTCTGCAGCAGAATGCAGTTGCACCTGCTGCCGTGGCGAGGCCTGCGCAGAGGCAGGCCAGGAAGGCGCCTGTCAAGCCCGCGCCTCCTCCGCCCGAGCATGTCATCGA

>Os01t0837000-01 2000 bp upstream sequence

GCCGAAGCGGACGCGCGGGTGCCTCTGCCGCGTGCCGTCGAAGAGGCAGTGGTGGATGGTCACCCGGATGGCACGGTCCTCGACGTGGCCGGCGCTGCCGCCGATCAGCACGGCCTTGTCGTGCGCCGAGAAGCGGCACCTGGACACGGTCACGTCCGTGCTGCCCCGGGTGACGTCGAGGAGGCCGTCGCCGCAGCCGCGGAGGCCGCACCGGTCGATCCACACGTGCCGCGACCCGGGCTTCACCGCCACGGCGTCGGCGTCGTGGCCGCGGCACCCTTCCACCTCCAGGTTGCAGACGATCACATGCTCGCACTCCCGCAGCTGGAGCCCCTTGCCGGACAGTGTCACACGCTGCCCGCGGCCGTCGATGGTCTTGTACGACGACACGCGCAGTCCCGTCGAGAGGTGGATGGTGCCCGACACGTCGAAGACGATCCATAGCGGCTCGCGCCTCCGGCAACCCTCCCGGAGCGACCCGTCCCCGTCGTCTGGAGATCGAGACCCATTTGCTACGTTGTTAGATGCAGGGAGATGGAATATGAATAGGCTTGTGTGTGTTAGTGTACGTGCCCTCGAGGGTCGTGACGTGGTAGACGTCGCCGTGGAGGCCGCCGATGGCGTGGCGGCCGAAGCCCTCGGCCTGACCGGCCATGACGCGCAGCGAGGAGTCCGCGGTGGCGTACGGCATGAACACGACTCCCTCCACGGATGCCGGTGGCTCGCCCCCGCACTGGCCGTCGAGTTGAGGAGGCTCCATGGCTACCGACTACGAGGAGGTTTTCGCGTCGCTATTTGTTGAGTCGCGCGCGGGGAAACGCCTGCTGTAGTGCTGTTTACTGCTGGAATTTGTTGCGACCGGGCCGTTTGGCCCGTTTCCGCTTTTGCAGCGGCGCGCGCGTTTCGGGCCGGCCCAGACAAGCCGCCAACCGGAAGTCCGGAAGGGATTAACACAGGTCAAACAGAGCGTTTTAGGAGTTTGCAGGGTCGTTGCATTGCATCCAGCACGAGCTCGTTACTATCTGCATGACGTTACGAGCATTCGATTTCAAACTATCATTCAATCTCGATCGTGATGTTTTTACCTCCCATTGCCCGGAAACGTCAGTGCATTTAAGCTTGTCCCTAACATAAGTTTTCGCTGTTAGATCGTTTAATGTTCATAACATCACATGAGACTAATCTTGAATGAAAAATCAGTTGGGAAACTCTCCCCCTTTGATCAATCTTAGCTAAAAACATCACATCGGAATATCCTCTTTGCCTGAAAACCCTCTCTAATCTTCTAGGCCAAAAAGGCAAAAAATCGGTCATGGCCCTTCCCATTTTTGTACTGTTTGATGAAAGTTTTAAGTAATATCATCGTGCGAGACATCCGACAGTGCGAAGTTTGGTGTGTCGTTGTCACGTAACCTTCCCTCGTTTCCTCATATTCCTACCGCCGGTTGCGGGTCGTCTTTTTCTCGCCCGTTTCATGGGCTCCCTTTCATGTCAGCATCGGATTGCGTCCGAACAAAGTCCACTCAAACTCTCAAAGGAAGATCCTGAATGGTCTCCGATCGAAGCTGTATTGGCTATCTGCGAAGTGTCATTGCCGGCTCAGTGGATACACACACAGGACACGAATGTACAATCACTAACCACGCAGCAGGTACGCAATAACCAGCGTTAGTTTAAGCAAAAAAAGGGTCGTCGATTACGTATAATTTCCGCTCGATAGAGCCAAAACCGAACGTGCCCATAATCCCCCCGTGCGTGGATTTACGTGACGCGCGACAGCGCAATTCTCTCCCACTTCCGATCTCTTCTTCCGCTTGGACTCATCGCCGTGACGTCGACGTTCCACGAGGAAAAGGACGGCGTCGAAGCCACCGGGCAACTTCTCACTCCCGCTCGCGTCTCTGACAAGTGGGCCCCACGCCTCGCTTTCTTCCCCCCACCTCCCGCCCGGCTGATGGCCCAAACCCCAACAAAACCTCCTCCGCGAAAGCTACAATCCA

>Os01t0872100-01 2000 bp upstream sequence

GCTGCGCCACCAATTTTAGCGCTAACCTCCGAGTTGTCATAAGTTAGCCACATTATCAAGGCCCTCTAAAGCAAAGGTCATGGGAGGTCATGGGTTTGAATCCGCGCCTGTGATTTTCAACCGTTATATGCCTAAAAAATTTGCTAGCTTAAGGCTTCGAACCCAAGACCTAGTGCTAAAAACCAATAGAGCTAACCACTGCACTAGTAGTATATTTCAGGTAGGCATCACCAATAGAGCCTTTTAGGCTTTGAAAAGGTCAGCGCCAATGAGAGTGGCGCTGACCCATGATGATGTGGCTAATTTATGACACCTCGGACGGAGGTTAGCGCTAAAATTAGTGGCGCTGAGATGTTGGATCTCAGCGCCACTTCCGATGGCACTGAGGTCGCGGTTCATTTTTAAAATAAGTTTTTAACACGATTTATTCTTGTAATAAGTTTTTACAAAGGGTTAAATTGTCAAAATTTATGGCATCCCGTGTATGGATCTGGAGATGGCGCGGTTTCCGTTTTGCCCAAGTGATGACGATGGCATAGCGAGCTCCCAATAGCAAACGATGACAGGGTGGTGAGGGCCTAGGGAAACGACGGCTGCTCGCCTCAGAGGAGCAGAAGGGCGTTGTACCTTCTCGGCGTGGATCTCCAGCGAAGAACGGAGGAGAGCACTGAGCCGACAACCCTTGGCAATCCTGTGTTCGTCGGAGGGAAGAAGATACAGTCCAAACCGGATATATTTTATTTCATTTTTTTTTATGTTTTAGCTAGTGTACTTATGATGTTTCTTTGTGTTTGGATCAAATGTTGATAGTATGCCATATTTTCATCTTTGATGCACACGGTTTTGTTTATGTTTCAATAATTCAAACCTAAAAAAAATTCAAAATTAATGTACCATATGTTGTAGTGGAATGTTTCAAGTGTGATTTGGTTATGTTTCATCATTTAAAATGTTGTGAAATATTTTCGTATAAAAAGGTGAAACAACACCAAATTTTTTTTTTGGAAAACCGGGCGTTCAATTTGTAGTCGTATCCCACTAATGATGCTTGTTTTCATTTTCAGCGCCGCTAATCCCTGTATCGCTGGACACGACACGTGCCCTAGAGCGAGCTTGAGCTTTAGCTCGTGTGAGTTTGGGCGCACGAACTGCCTACCGGTGAGCGAATCATCCTTTCACGCTTAGCTGGATTATGTGACAGCCGGAAGAAGATACCTAAAGAATAGAGAAGAGGGTTCGATACTATCGTAACTTTTGAAGCCTGCTCTATCTCTAGAAAGAACACAATGACTGAGTTTTCAACCAACGACAATGGAACAGGATCGATATTGCGAAGACGATGACAAAGGAAGCATCTCTTTAGCCTTTGACAAAGGTTGTGGTTCGGCAGCTAGCTACTTATGTAGCCACTATAGTCCTAGGTCAGCCCATCTGATTAGATAGAGTGCACTCAGTCCCTTCCCCTACTTCCTCTTTTTTTTTTTCTGTTCAGTTGTAATTGGCAACTTTACTCGTCTTATTGCAAAGATATGTAAGTCTTTTACGTATTAAAAAATTGCTCGCGAGCTTTGCGCTGGGTGGGCTTGCATTTTTGGGTCTACCGTTTAATTGGGTCAATTTATGCCTAATAAAAAGTTTAAAGAACACTGTTTCTTCTTTGATTAAATTTTACATTAGACTATTTTTTTCACATAAGTTTCAAGTAGAGATTTTATACATTTATTCAGTTCATCCGTTTGAAGGAGGCATCTATCTTTATACGCCATCACTCTCTACTCTCTATTACTCCGAATTACTATTGAGTATATTGATCTAGACATCTAGCCACAATCAGGCAAGGCCATCAGCCCATTACGACACAGAAAAATATCTTGATATCTGCCCACGTCAGCTATAAAAAAGTATCGAGAGTTCTTCCAAAAAAAAGAAGAAGAGGCGGCTAAAAGTATCCAAGCATCTGTCTGCAACTCTGCATCATAGCGGCCGGGGTGCCACTCTGC

>Os01t0875700-01 2000 bp upstream sequence

ACCCGATGCGTAACACATCCATGAGCAATTAAGTAGATAAAATTACAAACAGATCAGTGTTAGTAAGAATTTAATAAGAAAAAGTTAGTACGTATAGCCTTTTCCCACATCCAGGCCCGCGAGGCAGCCGCAGCGCGCGACGGCAAACGCGGGGATCGCCGTGCTCTCACAAGCCCACACAACCCGCCGGGCCGCGATGGATTGGGCGAATCTCGCGCGCGCAAGCGGGGGGCGCAAAACGTCTCGCCCCGCGCGGTGTCCGCAGGACGAACCCGGTGTGCGGTGCGGAGCGCGGCGTGTGCGACTGTATTACACTACGTAGTAGTACGTGGGACAAACATCTTTGGGCTCGCGCTGCGGCGCCCGCCGCACGGACGATTCGGTGGGGGGTTCACCATATGCTCGTGGCGGCTTGTTAATTCGCCGGGGATTGCATCCTGAATCCTGATCCTGATCACCCACCCCAATCGAGATTCGAGAAGCTAATAACGGACAGTTTGTCCCTCGAATCGCGAATCGATCCGGTTTTCGGTTTCCCTCTACTGTACTGCACGTGTTCGTCGATCGCAGGCATCCTCGCAATCACCCCATCAATCGAGAGATCAGTATATTTTTTACTACAAGTTTACAACCAACTCCAAAATTAATCTACCACGTGAGATCGCATCGCTCCAGATCCGAAGAAGTTACTGCAACTACCACTACACCAACAACTACAGCATGTCCAACGCCCCAGTGGCCACTGGTGGTCAGTTCGTCACGCACCTTGGCTCCAGACCCTGCGACAGAATACGTACGCACCGCTGGCAAATTTTGTTGCCACCCTTACTACACAACGTGCTCATTCAATTTCCATCGGCCCGGACGATCAAGATCGGTACGTGTATGTGTACCCGCCTACACGTCCGTATACGTGGCGTCGCGTCTGCACAGAGATGGACCAGGTGGCGATGGCCATCGACCGACCGATCGATCGCCATCTCCACCACCAGCTTCGCACATGCGCACACGGCCGTGACGAGACGACGCGCTCGGGAACAGAAGAATCCAAGGCGTTTGCATGGCCATTGACTAGAAGCGTACGTGCCCCGCTCGCAGTTTCTAAAATCCAATCGAGTACGCGTAGTAGCTCATATAATTTTATATGACAATCTGTCTGATAGTGTACAAAGAAAATTTTATATGTGTTGTGGTGACTCAGCCAGCGTGATAGGCTGACAGGCAGCACAGTGCGAGGAGAGCCACTTGATCTGAATGGGGGGATCAAGTAGAGACACACACACACACATCATATGCACGAGCAAAGCATTTTCATTTGGGCCGGTACGGACAAGGCAATAATTGCTAACCGTGCCGTTCTTATTCCCCAATCATTCACCTCCATCAGATAAAACCATCTTGTAATCATGCACTACTGCAACACACAGAACACTCCAGCTCTCGTCGGCCGTACACGCAGTAAGTCTTTGGCGCCTTCTCCCTCTGTAGTTGGCACCTTTCTTCAGGACTATATATTATAAAAAACATTACGACGGCTTTTTACGATTCCGTCTTACGGCAAAAATCCGTTAATGATGGATTTGGTTCTAAAATTATATTTGTAGTACTAGTAGTACCCCTAGATCAGTTTGTGTATTCCAATTTTTGTAACTAAACAAACTAATGAATCTGCTAATTTTTTATTTCTTGTAGCCCATAGCTTAGAACATACTCTTTTTGTAAAAAAAAACACAATCCTAATTACGAAATAGACATGCACTTATTCAAATAATGCTCAGAATTGAATTTTTTTATAGGGTTGTAACCATCAAAATTCTTTTTCTCCTCCACTGGAGAAGTAGATGGTAGGCAGTCCACTCCACTCCAGTCCTTGTCCATTGCCAATTCACCCCTCATGCCGCTATGTCCCCACATGCAATTTTTTCCCTCCCTCTCTCTAAACACCAGTTCAAATAAATGCCCCTACATAAAAGCCACTCTTCTCTCTCACTCACTCACTC

>Os01t0956200-01 2000 bp upstream sequence

ATATATGCCCCCAAAATAATAGTGATAATGATAGGAGCTCTACTATTGATGCTGCTTTACAATAATATCATTTTTGTTCTGCTTATACATCGCGACATCGGATGTATACATATTATATACACACGGAAAGTATGGAACCGTGTACTTCTGAAATTCTTTCAGTAAGAAGCCTGCACTTCAGATCTTTCTCTACTCCCGTGTCTTTAATCATTGTTAATAACATTATCCTGAACTGCGTATATGGTTGGCAGCTCGTGCATAAACTAATCTATCTATCCAAAAAGCGTGACATAGTTGAGAAATCTCGTTAGAATAGTTAAGGAGTCTCAAGTCACTTCACGGATTCGTGCTTGTCAATCTCGACAAAAAGAAGTTTTCATGGCTTTGGCTGGATTCGGTGTAATCGTTTCCCCTGTTCCATCCACCAACAAACCGGTCGCGATCGCCGGCTACTGATCACAAGCTGAAGCGATCGACGCAGCTATAGCTGAGCTAGCTGTCAAGCTGGCCGCGATAGCAGCGGGTGCAGCCGTTTTTCAGAGTTCGGGATGCGTCATGACGTCGTGAGCTGGTTGATGATCATATCTGTTCGATCGGTGGCTAGCTTTAATTTGCTTCATCCTAGGCATTCAGATTTTTGGCTTCAACGACCGTTTTCGGTGCAGCCATGTGCGACCTTGCTAATTTCACCGATGATCAATCAATCGTTCGCGTGTGGGAGTTTTGGACTGGACGAAGCAACGACTGTCAATATTAATTTGGCTTAATGACCAGGTCCAAATGGGTACAGATTTCTGTATCACAGATTAGTAGGAGTACATATAGGAAAACCTGATCATTTAGCTAGCTAGAATGCCCTGTCATTTGGTCAATGACGGCAAGACAGTTAAGAGGAGTACAAATTGATGAAAGGAACCTGCAACATGGACAGAGGATGGGTTTGATAACAGAGAGAGCGACCAAGCACCTACCATTATTCCTCTGACGAGTACTAATGCACTATATGTATTCATTTTTTTTACACTTATTCCATTCCATGCAAGGATGTCATTCGAGAAAGAATATGAATATATGATGCATATCATCATGTGTCATTTGAGGAAGAACATGCATGCGTAAGACTCTTATTAATTAATTGCCGCCTGTTTTAGCTAGCCTTTGTCATATACCAAAACCACACCATTAGATTAGTCAACACGTATCTCTGCTATCTTAACATGCAGTAGTTACGGTTACAGAAACTGACCACACTAAATTAGTATGTAGTATACACAGGCATACAGCGACAGCACATAACATATATTGCAGGAAGAAACCAGCATCGACCAAAAATGTTACGTTTCACACCTTGAAACCGGCGATCGCGCTGTGAGTGAAAATGGCGCAAACCGATCGATCCATCCATCGTCTACCTGTACGTACGTATATACCAAGCTAGTTAGGAATTAGACGACCTTTTCTACATGCATATAGTACTTGACATAGTCCCTAATTTACAACTTGATTACAAACATCTAGTTCTAGTACTGGACACTGTCCTAACTCCTGAGTTCTACAACGGACGAGGATGTAATTCATCTTGTTTGTTAGAATTCACGCTAAAAATAAAAAAGCTTTCTTGTTACTACATCCATCCTAAAATATAGTAATTTGGTACCGTCCTATAAATTTAAGCATATACTTCTATATTCAATTCGACATATTAGGATGTCCTATATCTCATGACGAGACGGAAAAAAAATGATTACACGTACAAAATGTACACGTGTCACTCTATGGGGCGATGAGTTCCAGGTAGATAGGTGCAGGTGCCGAACGATTGGACAGGAACCAGAAACGGACGCCGTCTCGGTCTTGCCAGCGAGATAGAACTAGAATTTATCCAATCAATCTGTAGTATCACCACCATTCACCAATCACCACCAGTGACCGCGCCGACCAGTGAGACGACTCAGCCGCCGAGCCTCTCTTTAAATATCCACACGATCGATCGAGACGCC

>Os01t0966000-01 2000 bp upstream sequence

TTAGGATATACTTTTATGTGTCGCCGTATAAATTTTGATCAGTCAACGTTTTTTTTTCCAAAGTACTGTAGCTAATCAAGGAGTAGTTAATAATCCGATCTGCCGGTGGCCGTGAGACGGGTGGTATAACAAAGGTTGTCCGCATGTTTATTTTGCGATTTCAAAGTACTATAATCATCTATTTTGCATGTCATGAATACGGAGCATCTTGTCTACATCTTGTGCGCGATCACGAGGGGAAATTTAAAAACCGTGCTGCTAATATTAATTGCCACATCATATGATTTGATTACGCTGGTATTTGTTGGGCGGCTGACAGAGGGATTTATTATTAAACAAGGAAAAAGCTCATTTGACTTCCTTAAATATCGGCCGAATCTAATTTGTGCCCTTTAACCAGAAAGCTGGATAAGAAAACTCCCCGAACTATTAAAACAGATACAATTTGACTCCTTAGGTGGGTTTGCAAGCGGTTTTAGCTGACGTGGCACCTACGTGGTACTGTCGATCCGGTCTTCGTCCCACGTGTCGTCGACGCGTTGCTTATGTGGAACTAGAGTAAAACAAAAGGCAAAATTTTGTAATTTTGTAGGACTGACATGTCACTCACAAACACACACAAGTGGGACCCATCGACAATGTGGGCCCCACATGTCATTCTCCCTTTCCTTCCCTTCTTCCTCCTCCTTCCCCCCTCTCTCTCTCCCTTATCTCTCTTTCTCTCCCCCGGCCTCTTTTTTCACCTTGCCCCGAGCGGCGGTGGGGAGCGATGGTGGGGACGGCGATAGGGAGCAGCGGCGGCCTGGCCACCTCTCCGTCGGCCTCTTTTTCTTTCATATTTTCCTCTCCTCTCACCTAAACACCACCGGTGTGCACACCTGCATTGGCAAGCTCTCCTAAACAACACATCTGCAAAAAATGGCACAAGCTTAATAATTTGAGCTGTTTGTAGATTCGTAGGAGACAAACATGCCGAATTGCGCGTATGTATTGATGAACATGCGCTGGTATCAATGGCGTTTTACTTGATGGCTGGAGGTGGTGTCAAAGCTGCGGGCGTGGTGGCATCGGAGGTGAAGGTGTGCGACGTCGTCGTTTGGGAGGTGAGAAGGCAAGGCTTCTTGGAGGCAGAGGCGGAAGCGGTGGCTGTGGTAGAGGCGGTTGCGGGGGAAGACGGGGATGAGCCAGTGAGCTGTTGGTGCTCACGGCGGCGAGCGGTGGGCACCTAGGTGCTCTTGACGTGGATGGAGCAACCGAAGCCGCGGCTTTTGCAACACGTGCGGCAATGTTGGTGGCCACAGTCTTTCTTTGCTTGGTTACCGCAGTCCTAGCACATCGCCGCGGCGCCGGAGCCAAACGATCCGTCAGCGATGTCCAGCATCGGGAGGGGGTTCTTGAGGTAGGGGAAGGCGCTAGCGCTACGTGATGAAGCTTCTCTCCGTTGACCCGTTGAACTACGACGACACCCCCATCGAAGGGATCAGGAAGATCCTCGAGGAGTTGTCCGGGGAGAGGATCATGCGGTCGTCCAAGATTCCCACGGCCAAATGGTCAATTGGAGCGGGAGCCAAGTGAATCGCTCCAGAAATTTATACTCCCTTCGTTTCAAAATGTTTGACACCGTTGACTTTTTAACACATGTTTGACCGTTCGTCTTATTAAAAAAAATTGTGAAATATATAAAATTATATGTGTACATGAAAGTATATTTAACAATGAATCAAATTATATGAAAAGAATAAATAATTAGTTAATTTTTTTTAATAAGACGAATGACCAAACACAAACTAAAAAGTCAACGGTGTCAAACATTTAGAAACAGAGTACTAGCGACTTAGCGAGGTGGAGTGGCGAAAACGTGGCTCCACGTCCTACTCTGAAAATAATTACAGCGCTACCCGTCCCCTTTCATCGAAATTACAGGAAATGCCATCGCATTACCTTGGATCCAGCCGAACCGTCGCGCCGCCCACTCGTCGACTCGGGCCTTATCGCGAGGA

>Os01t0966000-02 2000 bp upstream sequence

TTTTGATCAGTCAACGTTTTTTTTTCCAAAGTACTGTAGCTAATCAAGGAGTAGTTAATAATCCGATCTGCCGGTGGCCGTGAGACGGGTGGTATAACAAAGGTTGTCCGCATGTTTATTTTGCGATTTCAAAGTACTATAATCATCTATTTTGCATGTCATGAATACGGAGCATCTTGTCTACATCTTGTGCGCGATCACGAGGGGAAATTTAAAAACCGTGCTGCTAATATTAATTGCCACATCATATGATTTGATTACGCTGGTATTTGTTGGGCGGCTGACAGAGGGATTTATTATTAAACAAGGAAAAAGCTCATTTGACTTCCTTAAATATCGGCCGAATCTAATTTGTGCCCTTTAACCAGAAAGCTGGATAAGAAAACTCCCCGAACTATTAAAACAGATACAATTTGACTCCTTAGGTGGGTTTGCAAGCGGTTTTAGCTGACGTGGCACCTACGTGGTACTGTCGATCCGGTCTTCGTCCCACGTGTCGTCGACGCGTTGCTTATGTGGAACTAGAGTAAAACAAAAGGCAAAATTTTGTAATTTTGTAGGACTGACATGTCACTCACAAACACACACAAGTGGGACCCATCGACAATGTGGGCCCCACATGTCATTCTCCCTTTCCTTCCCTTCTTCCTCCTCCTTCCCCCCTCTCTCTCTCCCTTATCTCTCTTTCTCTCCCCCGGCCTCTTTTTTCACCTTGCCCCGAGCGGCGGTGGGGAGCGATGGTGGGGACGGCGATAGGGAGCAGCGGCGGCCTGGCCACCTCTCCGTCGGCCTCTTTTTCTTTCATATTTTCCTCTCCTCTCACCTAAACACCACCGGTGTGCACACCTGCATTGGCAAGCTCTCCTAAACAACACATCTGCAAAAAATGGCACAAGCTTAATAATTTGAGCTGTTTGTAGATTCGTAGGAGACAAACATGCCGAATTGCGCGTATGTATTGATGAACATGCGCTGGTATCAATGGCGTTTTACTTGATGGCTGGAGGTGGTGTCAAAGCTGCGGGCGTGGTGGCATCGGAGGTGAAGGTGTGCGACGTCGTCGTTTGGGAGGTGAGAAGGCAAGGCTTCTTGGAGGCAGAGGCGGAAGCGGTGGCTGTGGTAGAGGCGGTTGCGGGGGAAGACGGGGATGAGCCAGTGAGCTGTTGGTGCTCACGGCGGCGAGCGGTGGGCACCTAGGTGCTCTTGACGTGGATGGAGCAACCGAAGCCGCGGCTTTTGCAACACGTGCGGCAATGTTGGTGGCCACAGTCTTTCTTTGCTTGGTTACCGCAGTCCTAGCACATCGCCGCGGCGCCGGAGCCAAACGATCCGTCAGCGATGTCCAGCATCGGGAGGGGGTTCTTGAGGTAGGGGAAGGCGCTAGCGCTACGTGATGAAGCTTCTCTCCGTTGACCCGTTGAACTACGACGACACCCCCATCGAAGGGATCAGGAAGATCCTCGAGGAGTTGTCCGGGGAGAGGATCATGCGGTCGTCCAAGATTCCCACGGCCAAATGGTCAATTGGAGCGGGAGCCAAGTGAATCGCTCCAGAAATTTATACTCCCTTCGTTTCAAAATGTTTGACACCGTTGACTTTTTAACACATGTTTGACCGTTCGTCTTATTAAAAAAAATTGTGAAATATATAAAATTATATGTGTACATGAAAGTATATTTAACAATGAATCAAATTATATGAAAAGAATAAATAATTAGTTAATTTTTTTTAATAAGACGAATGACCAAACACAAACTAAAAAGTCAACGGTGTCAAACATTTAGAAACAGAGTACTAGCGACTTAGCGAGGTGGAGTGGCGAAAACGTGGCTCCACGTCCTACTCTGAAAATAATTACAGCGCTACCCGTCCCCTTTCATCGAAATTACAGGAAATGCCATCGCATTACCTTGGATCCAGCCGAACCGTCGCGCCGCCCACTCGTCGACTCGGGCCTTATCGCGAGGAGAAAAAGGGGAGACGAGCGAGGCAGCGGCGGC

>Os02t0135500-01 2000 bp upstream sequence

TTTCAGCTGATGAGTTATTTGGACGTTGCATCGGCTCATAAGGATTGCATATACATATGAGTTGCATCTAGCCGATGACAACAAAGGTTTCACTTATTAATCTAATCTTGTGGATTTCATGACATCGGACCTCCAGCCGATATGTGCTTTAGTCTTCGGATCAATGCTTATTCCATCATATCATTATAGCCGATTGGTTTATACTGGACTATATTATTGTTATATTTTATTATCAACAACCGATTGCCTTTACATCATCATCTACATTGGACATATAGCCGATTGCTTACACCTTATCGCTATCGGCTGGTATCGGCATCGGCTATTATCGGCTATCGGCTGGAACTACTCCATCGGCTTATCAGCCGATCGGCTGTTTTGATCTACTATTTGCATATATTGTCAGTTGCAGGATCAAACTGACTGGCACGCCCGCATCTCATTAATCTTTGGACCTGCACAGGAGCTAAGCAGATCTCCCAGACCGGTGTGTTCGATTTTTTCATCAACAACGACGAGACAGAGAGATTGATCGATCCAGACTCAGAGATATATCTCACCCTTTCACCTGATAGGTGGGCCCAGGGGGCAAAACAGTCCACAAAATTTGAAAAAGAATACAAATTGAGAAAAGAAATGATAATTACTTGAAAAACTGAGAAAGTGGTACCAATTAGACTAATTAGCACTTCTAATGACATATTATAAAAAAACATGCGGATTAGGTGGTAAAAAAGTTGAGGAGTACGAATTTGATGGTAAATAGTATAAATTCTCGTGGGCCCCACAACCCAGCTTCCTCCGCCTCCTCGGCCGGAGCGAGCCGGCCGGCCGGCCGGCGAGCCGCCGCTCGTCTCCGCCGTCGGTGGTAGGTAGCCAAGCCAAGCCACGCTGTCACCGAGGCGGTCAACGAGACGACGTGGCCACCAACGCGCTTCCCCCCACTGCCTCTGCTGACACATGCGGCGGCTGCTAATTACTGGGATTATTACTTGGTAAACCCTGCTACTGCCTCCGTCACACAATATAAAAGATTATGTTTTTTTCTTAATAAATTAACAAAGTAGTAAAAGAACATGTTATAGTATAAATTGTGATGGATGAGAGAATAAAATATATAAGATTTGATAGATGAAGAACAAATTAGCGCACCTTCCGTAAAAAAAGAAAAATCTAGAATTAGATATTAGGGTACGAATCTAAACATGTGTATATCTAGGCCCTGTTTAGATGGGACTAAAACTTTTAAGTCTCTATCACATCGGATGTTTGGACACTAATTATAAATATTAAACGTAGACTATTAATAAAACCCATCCATAATCTTAGACTAATTCGCGAGACGAATCTTTTGAGCCTAATTAATACATGATTAGCCTATGTGATGCTACAGTAAACATTCTCTAATTATGGATTAATTAGGCTTAAAAAAATTGTATCGCGAATTAGCTTTCATTTATGTAATTAGTTTTGTAAGTAGTCTATATTTAATACTCTAAATTAGTGTTTAAATACAGGGACTAAAGTTAAGTCCCTGGATCCAAACACCACCCCAAATTCGTAGTATTAGAATGTGTCACATTCGGTACAGATTGATTTTTTATGGGGTAAATATTATTGAATCGTACTACCAATTTCCTACCACGGTATAGATGAAGTACAAAGTTGATATTTCCTCCCACGAAAAACAAACTATATTTTCTAATACAACGAATCTAGATAGTGTACTAGAAAATATCATATCTAATATTATATTATTTTTTTTATAGTACAGAACGAGAACGAGTATGTGTGATAAATGGGAAATAAAGTATCGTCTTAGGATCAGAAATCTCTTTAGGAGTATTATGAATGGAGTACTAACCTCGCCGTTAATTGCATAATAATCTGGACAAGTGATGCCTAATTAAGCCATAGTTTAATCGGCAATTTGCTTCAGATCCAGCTCGGCATGAGACTATCAGAGGCATAAGTGTCAAAGCATCTCTCGAGTCTCGAGT

>Os02t0135500-02 2000 bp upstream sequence

CAGCTGATGAGTTATTTGGACGTTGCATCGGCTCATAAGGATTGCATATACATATGAGTTGCATCTAGCCGATGACAACAAAGGTTTCACTTATTAATCTAATCTTGTGGATTTCATGACATCGGACCTCCAGCCGATATGTGCTTTAGTCTTCGGATCAATGCTTATTCCATCATATCATTATAGCCGATTGGTTTATACTGGACTATATTATTGTTATATTTTATTATCAACAACCGATTGCCTTTACATCATCATCTACATTGGACATATAGCCGATTGCTTACACCTTATCGCTATCGGCTGGTATCGGCATCGGCTATTATCGGCTATCGGCTGGAACTACTCCATCGGCTTATCAGCCGATCGGCTGTTTTGATCTACTATTTGCATATATTGTCAGTTGCAGGATCAAACTGACTGGCACGCCCGCATCTCATTAATCTTTGGACCTGCACAGGAGCTAAGCAGATCTCCCAGACCGGTGTGTTCGATTTTTTCATCAACAACGACGAGACAGAGAGATTGATCGATCCAGACTCAGAGATATATCTCACCCTTTCACCTGATAGGTGGGCCCAGGGGGCAAAACAGTCCACAAAATTTGAAAAAGAATACAAATTGAGAAAAGAAATGATAATTACTTGAAAAACTGAGAAAGTGGTACCAATTAGACTAATTAGCACTTCTAATGACATATTATAAAAAAACATGCGGATTAGGTGGTAAAAAAGTTGAGGAGTACGAATTTGATGGTAAATAGTATAAATTCTCGTGGGCCCCACAACCCAGCTTCCTCCGCCTCCTCGGCCGGAGCGAGCCGGCCGGCCGGCCGGCGAGCCGCCGCTCGTCTCCGCCGTCGGTGGTAGGTAGCCAAGCCAAGCCACGCTGTCACCGAGGCGGTCAACGAGACGACGTGGCCACCAACGCGCTTCCCCCCACTGCCTCTGCTGACACATGCGGCGGCTGCTAATTACTGGGATTATTACTTGGTAAACCCTGCTACTGCCTCCGTCACACAATATAAAAGATTATGTTTTTTTCTTAATAAATTAACAAAGTAGTAAAAGAACATGTTATAGTATAAATTGTGATGGATGAGAGAATAAAATATATAAGATTTGATAGATGAAGAACAAATTAGCGCACCTTCCGTAAAAAAAGAAAAATCTAGAATTAGATATTAGGGTACGAATCTAAACATGTGTATATCTAGGCCCTGTTTAGATGGGACTAAAACTTTTAAGTCTCTATCACATCGGATGTTTGGACACTAATTATAAATATTAAACGTAGACTATTAATAAAACCCATCCATAATCTTAGACTAATTCGCGAGACGAATCTTTTGAGCCTAATTAATACATGATTAGCCTATGTGATGCTACAGTAAACATTCTCTAATTATGGATTAATTAGGCTTAAAAAAATTGTATCGCGAATTAGCTTTCATTTATGTAATTAGTTTTGTAAGTAGTCTATATTTAATACTCTAAATTAGTGTTTAAATACAGGGACTAAAGTTAAGTCCCTGGATCCAAACACCACCCCAAATTCGTAGTATTAGAATGTGTCACATTCGGTACAGATTGATTTTTTATGGGGTAAATATTATTGAATCGTACTACCAATTTCCTACCACGGTATAGATGAAGTACAAAGTTGATATTTCCTCCCACGAAAAACAAACTATATTTTCTAATACAACGAATCTAGATAGTGTACTAGAAAATATCATATCTAATATTATATTATTTTTTTTATAGTACAGAACGAGAACGAGTATGTGTGATAAATGGGAAATAAAGTATCGTCTTAGGATCAGAAATCTCTTTAGGAGTATTATGAATGGAGTACTAACCTCGCCGTTAATTGCATAATAATCTGGACAAGTGATGCCTAATTAAGCCATAGTTTAATCGGCAATTTGCTTCAGATCCAGCTCGGCATGAGACTATCAGAGGCATAAGTGTCAAAGCATCTCTCGAGTCTCGAGTCTT

>Os02t0161100-01 2000 bp upstream sequence

TATAAATGACTAAATGAACACTATCACCACCAAGGTCGTGTCCATAAAAGATATAATGCCCCGATCTTCAGTGGTTCAGAAACCAGGGTACAGAAAAAAATCATTATAACAGCATGCAGGCACTGCTCTTATTTGAACTCAACGGGACAGTTGGAGCAAAGCTTTCCGGTGCACTAGCACATAATTTGGCTTTTTCAAGGACAAATATCTTCTAGTGTTACCTGTTGAAAACAAAACAGAAAATGAACAAACCGAATCAGGGCAAACATATAACGCAAATGTAGATCCAATCGTCCAAAGGTAACCTAAATTGAAAACAATAATTCACTGTAAATGCGCATTCACATTTCTTTTTTTATATATGCAAGGAGCGTATCTTTATATTAAGGAGTAATAAAAGTTATTTACAGGCACATTCCCAAATTTAGGATAGCATTACGAATTTAGTAAAAAAAATTCAGGATTCCCCATTTCACTATACTTTGAATACCCACCAAGCATTCCTGGTAAGAAACATAAAATCACACCAAACAAAACATACTCCAAAATGACAAGGACTACGAAACTAAGCAGTGATTCATCCCAAGAAGGTCGATCACACGACGATTGATCTGGTGTTCTAGGAATCTAGGCTACTCCTATAAAGGAATCAACAAAGAAAAACCTGCAGAATCCATTCATTTATCAATCCCTGGTAGAACCCGTCATACTAGGGCGAACACCCAGCTCCCCAAACCCTACCTCACCGTGACACAGAGCTACAAATCCAGCCATAAACCAATCAAACAAGCAAACAATTTACCCCTATTAGGCCTTAACCGCAGAGATCATAGCCATGATTAACCTAACCAAACCAATAAACCGCATAAGCACCGCATCAGCAGCGGATCTCTTTGCGGCCACATGAAAAACAACCAAGCAATCACCAAGGCTAATACTACCAACCTGATCGAGATGAACTGGCCGCGGAGGAGAGGCGGGGATCCCGAATACTCAGGTGAAGTCTGCTCCAATCCAAGCCGGCGTAGCCACAGCGCAGCCGCCCTGGGCTTTGACCTCTTTGCAAGCACAGTTCTATTAGTATTTTAATTATTTTTTAATTACCTTTTTTGGGTTATTTTAGTAGAGGAGTGGCATCTCCGCTCCGCGTCGCAGCCACATTCTCTGATGCCTCCAGAGCACAGTCCACGTTCGCATGGCAGCTCAGCTCACAGCAGCGCAACGCGTCGTGCCGCCCGGTGGGCCCCACCGCCGCCACGGGCCTGGTTGGTGGGCCGTAGCCCACGTGAGGCCCACCGGCGGCAAGGTGGGCTTTCTCCTCGTGGCACAAAAGTCAGTGTGTGGCCGTGGCGAGGCCGGCGGAGGCAGCGGCGGCGGAGGAGGAGGTCGTCGTCGCCGGCAGCTCAGCTCAGCTCACAGCAGCGCAACGTGTCATGCCGCCCGGTGGGCCCCGCCCCCGCCACGAGCCTGGTGGGTGGGCCGTAGCCCACGTGGGCCCAATTGACAAGGTGGGCTTTCTCCTCGTGGCCCACAAGTCGGTGTGCAGCGGCGGCGAGGCTGGCGGCGGCGGAGGAGGAGGTCGTCGCCGGCGGCCGCGCGGTGACGGGTGCAGTGAAGTTCGGCGCGAAACCTCAAGAGCACCGCATTTTCTACGCTTTTTCTTTGTTTTTTTTTTTGGCTCGAGTGCGCCTTCGCCGAACAGATAAAGGCCACGTCTTTTCTTTTGCTTTCTCCTCTCCAAGCGAAGCCACTGCTTCTACTGCGTCGACTTGGAATTTTGCCGAATCTTTTTTTCCTATGCATTTGGCAAGACCAAGAAAACAAAAAGAAAAGAGATTATTAATCATGGCGACACGAATGGAAATGGTTAATAATCGCATTGAAATCTAAATCCGATCCTACTAAAGAATTAATTAAAAAATAGGAGTACTAATAATGGGCATAGATAGAGGGAGACCTCACTCTCAGCATAAAAAAATTCCATCTTTGCAGGCAGACGC

>Os02t0227200-01 2000 bp upstream sequence

TAGTTGATGGCTTGACGCCATTGCAAAACCAAAATAGTCTTCTAGTTATCATTCGCAACCATGAATGGATCGCGGGGACTTTGGGGCTGTTTGGTTCGTGTCCTAGGGGCCACGCCTGAGAAAAACTTACGCCGAATAGGCAGCGTTGGGAGATGTAACACTTGCCTCATCAGGCAGCCTGAAGCTTTGTGTGTTTGGTTCGCACACATGCCTGAAGAATAATAAAATATCAAAAAGCAACTTTTACCAGAGCAAAGTATGGAATAAATGACACATGCATCATTAAGAGTAGCTGACTGAAGCTCAGGCGTCCAAATTCGATGGCCTGGCTCAGGCTAATGAAGTTGCCTGAGGATTTAGCAAGCTAATGAAGAGTCAGGTCAGGCTAGCTCAGGATATAACAGCGCACAAACCAAACACATAGTTGTTGGAGCTCAGGCGGCGCTCCGGCCAGGCATACAAGCATCAAGACGCGAACCAAACAGCCCCTTCCTCCCTATAAAAGCAGACCAAGAGGAGTTGGTCTCATTTTCTTGGTAACAACAACCACAATACTCCATTTGTCCTATAATATAAGGTATTGTGTTATTTTTTAGAAAAATGAAGTAAGTGCTTTGGTTTAGTGCTAAATCTTGCCCTACCAAATTGTTTGTACATGTGTAATGTTTAAATTGATTCCAAAACTTGCCAAATCAATAGAAATATGTCCTAAAATATTGGTAGTTCCAAAAATTATCTAAAACTTGGCACTAGGTAAGGTGGCAATCCAAATATGCTCACAGTAAAAGGATATGTCCTCGAAAAAAGTAAAAGGATATGTGATGTTACAAAATGTGTTAGGTGAATAAATAAAATACTAGTAAAATAGTGATAGATGTGAAATAAACAGTATAAAGATGATAGGTGGGGGAGTAAAGTACTAGTATCTTAGGACCTTAGAGCAAGTAGAATAAAAGAATTGAGTAGGCTATAATATCTCCACCATGATAAAAATGATGAGCCGGAGAGAATAAGAAGAGAGAACTTGAGAGGGCGATCATATTAGCGTCGACTGTATTTAGCCTTTGAGACTAGGTGAGACCATTGTAATTGGCTAAACTTAGCACATGTCGGTGGATCTGGATCATCTAATTTACTAAAGGAACGTTACATGCGTGCAGTTATGCTATATTTGCCATCCATTTTCATCTGACAGCTCCTGCTGGCTCTCCTCCTTGTTCATATGTACAAAAGAACTTCTGACAACATTATTGGATTTAGGTTAAATCATTTAGCATCATCTACCGTGAAGTGCGAATAAAATTACATCACGATATTGTCCCAAAGAAAAGGCAGCATTTTGGTTCATGTACAAAAGAACTTAAAAAATTAATCAGTGTAATTTAAAATTAGATTAAAAAAACCTCTAAGCCAATTCTAAAATTAAGCTCTAAAATTTAAAATAGATTTCCTCTTTGTAATGTTACTGGTGATATTTATTTTGACAAGTGCTTTTATTTGTAAGTTTGATTTACAAGTGCTTTCATATGTATGTTTGTTTTAAGACAATAATCACAGGCTACAATTAAATTTTAACTATTAATATTATATTTTATAAATATTAATATTAGTTTTACCCGTTACAACGCGCATGAGCAATACTACTAGTATTAAATAAAATACAACCATTAACCCTGAAAAAGAAAGTACAACTACAAATTGGCCCTAATTTGTTAATCTCGGGGTAGGAAAACCAAACAAACTCTAATCCCCCTGTTGCAATTCCAAATCCCCGAATTTGCATCGCCATTCGCACATGCCACAACATGTGTAGCACTAGTACACCGGTAGACGTGACGTGAGCAATGAGCTTTTCGGCCGTCGCGTGCGCGAGCCGCCGGCTGCTATGCCTATGCTAGCTAACCCAAGTCGAGTAGTAGTAGCAAAGTCCGACCCGTTCACACCATCACCATCACCATCACCAAATCGATCTAAAAATACTCGCCTTCTTCTCCACCCGA

>Os02t0234500-02 2000 bp upstream sequence

GGAAGAAATGATAGAAGTGATCAAGCTAGCAGTCTGGTGCTTGCAGCGCGACTGCAGCAAACGACCAGCCATGTCACAGGTTGTGAAGGTCCTGGAAGGCGCGATCGACACGGAAACATCCGCAGGCTACGATGCAGCTAGTAAAGATGATATTAATTTTGACGCTTCATCCCCTCTGTCCCCAGCACCAGTGTCAGCACGATGAAACTCTTTTTAAGTAGGAACACCGATACTTGTTACAGTTTAGTTGCAGTACTGTGTGCAGTGTGCTAGTGTGCATCTCATGTAAATTTGTCCCTTGTAATATGTAACATCCTCATCAATGTGTGAATGAATGAAATATTATAACGATTTTATTATCGAGCTATTTCATGGTTTCCCAAGTAACAAGACGAACAGCAAAGGTAGCATGTGAAGGGCTAGGGAAAAGGTAACATCCGAACCTTGCCATGCTCGGCCACGCATTTCGTCGAACACGCGGAGGGCCGACACCATCTGTGCCCCCCCGACCGCCGACGGGGAGGCCACGCCGAGGGACACCGCCCCCGGACTGTCGGCGCCGCGCGCCGCGTGCACGCTCGTGCACCCACCCCGTAGGAATCACTCGACGCCGCAGCCCATCCTCATCCGGCGCGGCGCGGCGGGCGACGCCGGCGAGGCTGGCATGACTCACGAGCGCCAGCGCGCGGCGACGAGGGAAGCGGGAGAGAGGGAAAGAATTATAAAATAATAAAATAAAAATCTAAGCGCGACTCGAGCACTGCACGAATCTCGAGGCGCACGTGGCCCGTGCTGCGGCGGCCACGTGGGCGCCCGAGGACTCGGAAGAGTTTTGTGTGGCCGTGGTATCCTCTTCCCTCCACTCCACTCTCCAACTTCTCCCGCAGCATCGCCGCCGTCGTCGTCGCCGCCGCCGCCGCGAGGTGAGGGCTCGTAGCACCGTCCGGGAGGGCGGAGGAGGAGGAGAGATGAGCTGCTGCGCCGCCTCGTCTTCCTCTCCGGTTCTCGCCAACCCCCGGGTGAGTACGCACGCCCTACCCCGTCTTCGCCTTGCCGGCCCGCCGCCGCCGTCCTCTCCATCGACGGCGGCGTGAAATTCCTGCAGGAATCCGATGCGTTCCCCTTCCGCTAGTTCATTTCAGCCGCCATCATTGGTCTTAGCGGACTTAGGGCAAGATCAATCCCGCCGCGAACAAACCCGCGCGCTTCCGATTTTTCTCACAAATTCTTCGGTTCGATGTGCTCGTGGAATCGAATCGTCGGCGCGAATTCGCTAGTTCGTTGGGATTCCGAAAGGGAGGGAATGTTCCGAAGAGAATCGATTTTATAATATGTCTGGATTTATCTTTCACCAAGGTCCCATGGTCTAGCGGTTAGGACATTGGACTCTGAATCCAGTAACCCGAGTTCAAATCTCGGTGGGACCTATTTTTTCTCCATTTTTTTTCTGACTCGCTGATCCTTGCTAGGATGTGGCTACATCCAAAACACCATTTGCTGAGTCGAGGCGTCGAGCTGAATCCATTTGCTGAGTCGAGCTGAATTTATATATGCTCCATGAAATCTTTGCTACGAATTTATGCTTTGTTTTCATTTCAAGGATGTTGCTGCATCAAAACACCATTTGCTTAGTTGAGCTAAATTTATAAATCCCCATGAAATCTTTGCAACGAATTTATGCTTTGTTTTCATTTTTTATTTTAAACTTTCAGGGGGGATTCGCTGCTAGCTGCTCAACAAGAAGGAACCACAGGGTGATCTTCCTCGGTTCAAGGCAGTTTTCTCCGATCATATACAGCCCAGCACGCCGCGCGTCGTCACGGCTGTCTCGGAGAGAGGTGATAGCTTTTGCAGGCCAGCAATCTTGGGATATCGGCAGATTTGTCAGGACGCTGTATTTCTTCAACGGGCCACCGAATCCTCTCAAGGTTTACTCACTGTATTGGGATTTTTTTTCACCTCTTCTCGTTACAGTTATTTCACATTTGGCATTGTGCTCA

>Os02t0313700-02 2000 bp upstream sequence

AAGAGTTGGAAAACGTGACGGTTAGAACTGGTACCTCCGTCGTGCGTCACAGGCAACCACATCCGTTGACCCAGCAACCAGAGAGGAGGAATGCCCGTGGAAGGTCCAAACGGAAATTAGGTGATCTCGCATGGCCCATGTATGCTTCCATTCCACCCGTATATCTGGCGCGTCTAGTATCGTCAAATCTCTACCCATGCACATGGTCACTCCGGAAATGCTAATGGGCGGGTGATCGCTCCCCCGTCCGCCCAACGATCACCCTTTACTCCTCCCCTATACTCAGATACACTCCTCCCTCCTCTCCTCCTTCCTTCTCCTCTTATTCTCTTTCTACTATAGTACACCGCAAATTTTTTTTAAAAAAACAAAAAGTTAGAAAAATTTATGTATAGAAATACTATATATAAAAAATTTAAATTTAAATTCAAATTTGAATCGGCTATGTAAACTTTTGGCTTATAAACTTTGGATCTATAAACTTTAGGCGTATAAACTTTAGATGTATAGAAATACTATATATATAAAATATTTGAATTCAAATTCAAATTTAAATCGGATATAATTCAAATTCAAATTTGAAACGGGTATATAAACTTTTGACTTATATAAACTTTAGATGTATAGAAATACTATATAAAAAAATATTTGAATTCAAATTCAAATCAGATATAATTAAAATTCAAATTTGAAACGGGTATGTAAACTTTTGACTTATAAACTTTGGGTCTATAAACTTTAGGTGTATAAACTTTAGATGTATAGAAATACTATATATAAAAAATATTTGAATTCAAATTCAAATTTGAATCGGATATATAAACTTTTAACTTATAAACTTTGGGTCTCTAAACTTTAGATGTGTAAACTTTAGATATACAAACTTTAGGTGCATAAATTTACTAAAATAGGAAAGTATTACAGTGCTTAAAAAAAAAAGAAAATCAGGTGGAGAGATGGAGGGGGGAGGGGGATTGATCGCTACCCCATCTTCCGGCGATCGATCGCCCAGTAGGATCCTCCGGTCACTCACACGAACCGTAAGCGTCTGACCATGTATGCCGGGCCACCATCCGTGGTCCGTGGACAAGGGCAAGGCTCCGAACGGCCGAACATTCCCAAATCGTATCGAATGAGGGCCTACTGGTGGCAGTTAGTACTTAACCATCTTCTGCCGTATGGAGCGGGACCACAGACGGGTTACAGGAGTTGAGGAGTACGCAATACTGTGATTTGCTAGCCCTCTTACAATCTTTGATACAATCCTATGTGGCGGCCTCTTACTTTGCTCTCACACCAACACGAGTACCATAAAGAAACCACTCATCGCCTCTGCGAGTAGGACACCATGTTGGAGAGAAGGAGAACCACTGAACATCAACATATAAGAAAACAAGCCCAAACACATCCCTTGTCAGTCCAAAGTCCAAATTCAACCATGGTACGTCGGTGTCCATTTGGCCAATTCCAATTCCAATTCCTCGGCTGCGCGTTGAATTACAGGTCCAACTATTTCGGCCACCTCTACTGTCTCTCTCCCATCCTCTCCGCCCCCAAATGGGCAACAAAATAAAAAACCCCAACCCGCGATCCGGCGAATCGGCGTCGATGCTTAGCCGGGGAGGTAAACCCCCGACGCAACAATTCCACACCTACCGCGGCTACCGAGCCACCGGCCAACCCCTCCCCCTCCCTCCAAAACCCGCACGCGCGGATCCCGTCGCGAGAGCCGAAATCACACGTCGCCACCGCAGCAGCAGCCGCGGCGCGCCACGCGGCCGCGGCGGACCCCGCCCGCCCGCCACGTCCCCCGCGCCGCCGACCACTCCGGCACGTAGTAGGCCGTGAGACATCCACCATATCTTCCCCGCCTCGGCGCGCGCGTACTCCTACGTCTACGTGCGTGAGCCCCCCACACACCCTCGCTCGCCGCGCCGCCGTCTCTAGTTTTTTTTTTCTAAAAAAAAAAAAGCCTCGGCCGCCGCAGCCGCCCCTGCCCA

>Os02t0541300-00 2000 bp upstream sequence

AATACAAGCTATTTGGGTATTATACAATAAAAATCATAAACTTTGAGCGTTCTGAAGCAAATTTTACCTATTTTATTCACATATCAACTTCTTTGTATTATTTTATTTCCCATTTATACCCTCTTACCTATTTTCTTTTGAACTATTAAATGTTGTTACTTTCTTAATATACAGAAAATGACATAATCTTTTGTATTATACTCCATTCATTCTAAATTGTAAGGCATATAGTTTTTACACGGCCTTTACTAATATACTTTGATCACTACTTTATTTTATAGTATGTTATTAAACAACTAAAAAAATTAATATCACATAAAAGTATTTTAAAATACAAATATAATGATATAATATACGTAATACTTAACAAAACTATATTGCTAGTGTAATATTTGTTAAAATTTATCCAGTTTGATTTAGCATTTTAGAACGGATGAAGTACCGCGAACGGATGGAGCATTCCCCCATCCAAAAAATACTCAACTTATGTGGTTTGAATTTTGTCTCACAGGAAACAACTTTCACCATCTTACCTACTGTCTGCACATTAAATAAGAAGTTTTATCTCTTAAATACCCTTTACCTATCTATCACTATTACTATTTTATTTAATAATAAGGGGGTATTTTAGTCATTTTTACTCACTATTAAATTTACATTGGAATATTAGGAATTTTTTTTAAAAAACGGAGGGAGTAGGTAGGTAGAAGAGCAGTGCCCCACCATAGAGGAAGACAGGGAAAGACAGACATGAGGAGGCAGCCGTTGGCGCGCGTGAACGGGCAACGGCAAAAGCAAACCCCACACGCTTGGCGCCACGTCACGTGGACGAGGCGCGTCCGTGGCGTGACCAGAGGTGGGCCCAGAAGATTCCCACGCTGGCTGCGTGGTCGTGGTGGCGGCCTCGCCTCCCACGGGCTCGGCCCCACCACCGGATTCAGGAATCTCGATATTTTCCCCTTTTCCATGGCCCGCACACCCCGCCGGGCCCACCTGTCAGACATGTGGGCCGAAGAGATACTACTGTAGTAGTTTACTCCGCTACAGCGCGCTACTGACTTGTCGCGACAAAACAAAACAAAAAAAAGACACCACAGTATTGCAAATTGCAAGCGAAGCCGTAGAGCAGGGCGCTACTGCCTACTGGTACTGTGCACAAATCCTGCTTAATGTAACAATTAAATAAATTGCTGCTTACTAAGCGTAACTTTGCTGCTACCAGTAGTAGATGCGAGATTATCATTGATTTGTTAAAACAAGATCTAATCATGGTTACACCAGATCCTCTCACGTGTTAGAGCAAGTTTAATAGTGTAAATAACTACTAGCTCTAAATTATTTATAGTCAATTTAATAGATAATTCAAACAATAGTTACATATAAACATATAATACATGGTCCTACCTATCATACACATATTTTATCTTGGAGTCTGTGCTGTAGCTGGCTACAGATCTGTAGCTCACTGCTTCTCTCTCCCTGATATGTTTATAGTTGGTTAATAGTATGCTATTGTACCTGTACTTAGCGTGATTATCAATCGTGGTCGACCACAAATCCAACTGTCCATTTTTTTCATTAAAAAAAAATCACCAAATCCGGATCTTAGCGATAAGCCCCACACGTACGCGCCATGCATGTCAGGGCAAGCAAAACGAGAGAGCCCCTCTCTTTCTCTGTAAAAAAAAACCCAAAAAAATGAAAAAGAAAAAAAGCAGGCCACGTGGCACCTGTCGGGCTCAGACCGACTCGCACCGATGCACCGACCCTCCTCCGTGACAAACGCCCACCGACACGGCGGGCCGGCCTTCACCTGGGCCCCACACGTCAGCGTCGTTGTGTAGAAGGTGAAAGCCTCGTGCGATCCGAGCCCGAGCCGAGGCGGCTACAGTGTGACGACGCGATTTTATCGCGGAGCTCCTATAAATAGGCCCCCGCGAAACCCTCGTAACCCCTCTTCTACTATCTCTCTCTCTCCTCCGGCCGCGGCTGCTCCCCCC

>Os02t0541325-00 2000 bp upstream sequence

TACAAATATAATGATATAATATACGTAATACTTAACAAAACTATATTGCTAGTGTAATATTTGTTAAAATTTATCCAGTTTGATTTAGCATTTTAGAACGGATGAAGTACCGCGAACGGATGGAGCATTCCCCCATCCAAAAAATACTCAACTTATGTGGTTTGAATTTTGTCTCACAGGAAACAACTTTCACCATCTTACCTACTGTCTGCACATTAAATAAGAAGTTTTATCTCTTAAATACCCTTTACCTATCTATCACTATTACTATTTTATTTAATAATAAGGGGGTATTTTAGTCATTTTTACTCACTATTAAATTTACATTGGAATATTAGGAATTTTTTTTAAAAAACGGAGGGAGTAGGTAGGTAGAAGAGCAGTGCCCCACCATAGAGGAAGACAGGGAAAGACAGACATGAGGAGGCAGCCGTTGGCGCGCGTGAACGGGCAACGGCAAAAGCAAACCCCACACGCTTGGCGCCACGTCACGTGGACGAGGCGCGTCCGTGGCGTGACCAGAGGTGGGCCCAGAAGATTCCCACGCTGGCTGCGTGGTCGTGGTGGCGGCCTCGCCTCCCACGGGCTCGGCCCCACCACCGGATTCAGGAATCTCGATATTTTCCCCTTTTCCATGGCCCGCACACCCCGCCGGGCCCACCTGTCAGACATGTGGGCCGAAGAGATACTACTGTAGTAGTTTACTCCGCTACAGCGCGCTACTGACTTGTCGCGACAAAACAAAACAAAAAAAAGACACCACAGTATTGCAAATTGCAAGCGAAGCCGTAGAGCAGGGCGCTACTGCCTACTGGTACTGTGCACAAATCCTGCTTAATGTAACAATTAAATAAATTGCTGCTTACTAAGCGTAACTTTGCTGCTACCAGTAGTAGATGCGAGATTATCATTGATTTGTTAAAACAAGATCTAATCATGGTTACACCAGATCCTCTCACGTGTTAGAGCAAGTTTAATAGTGTAAATAACTACTAGCTCTAAATTATTTATAGTCAATTTAATAGATAATTCAAACAATAGTTACATATAAACATATAATACATGGTCCTACCTATCATACACATATTTTATCTTGGAGTCTGTGCTGTAGCTGGCTACAGATCTGTAGCTCACTGCTTCTCTCTCCCTGATATGTTTATAGTTGGTTAATAGTATGCTATTGTACCTGTACTTAGCGTGATTATCAATCGTGGTCGACCACAAATCCAACTGTCCATTTTTTTCATTAAAAAAAAATCACCAAATCCGGATCTTAGCGATAAGCCCCACACGTACGCGCCATGCATGTCAGGGCAAGCAAAACGAGAGAGCCCCTCTCTTTCTCTGTAAAAAAAAACCCAAAAAAATGAAAAAGAAAAAAAGCAGGCCACGTGGCACCTGTCGGGCTCAGACCGACTCGCACCGATGCACCGACCCTCCTCCGTGACAAACGCCCACCGACACGGCGGGCCGGCCTTCACCTGGGCCCCACACGTCAGCGTCGTTGTGTAGAAGGTGAAAGCCTCGTGCGATCCGAGCCCGAGCCGAGGCGGCTACAGTGTGACGACGCGATTTTATCGCGGAGCTCCTATAAATAGGCCCCCGCGAAACCCTCGTAACCCCTCTTCTACTATCTCTCTCTCTCCTCCGGCCGCGGCTGCTCCCCCCCCCCCCTTTCGTCCCCCAAAATCTCCGCGCCTCCGAGAAGATTCCACCGGCGGCGGACGACGACGACGAAGAGGATGGTGGGGAGCGTCGGGAACGGCCTGGTCGACCTGGGTGGCGCAGCCGTGGCCGTGAACGGCGTCGGGAAGGGGATGCGCCCCGAGGCGGTGGCGGTGGCGATGGAGGTGGAGTCGCCGCCGAGGCCTGCGGAGGAGGAGGGGGAGGGGTCGCCGACGAGGAGGGAGATCGTGCTGGGGAGGAACGTGCACACGGCGTCGTTCGCGGTGAAGGAGCCCGACGCGGACGACGAGGAGACCGGGGAGCGGGAGGCCGCC

>Os02t0607700-01 2000 bp upstream sequence

CACTAGTAGCAAAAGTTGGTAATTTGGCATTTATCATTTACTAGTATAGGGTAGCAAATTTTGCCAAAAAAATGCTCCGGGACCACGCGCTCTCTCTTTTTAATGCTAAATTGGTAAAACTTTAGGATGCATCTATACACCAACTCCATGCTAAATTTTTTACCACTAAAACTTTTACCATTTACCATTTATTTTTGAAACTAAACCGGCAGGCAGAGGCTGCCAATTATATTTTCAATAAGCAGGAGTAAAAACCTAGACGAGTTACAAGAAAAAGAAAACAAAGGATGCTATTGTTGTCGGCCGAACAAGTTCACAGCACGCACAACAACTCAACGCTACAACCCACTCAAAATAGACCATCGACGGAGCCCAAACACGAGTCATCATTGCCAAGCTTGCGCAAAAGCGACGTAGCAGCTCCGACCTTCACTTGAAAGCATGTTATCGTTGCCAAGCTTGCCAAAGCAAACCAGCTCCGACTTCACGGCAAACTTGTGTCGTCGTTGCCAAGCTCATCACAGAAACGACCAAACAGCTTCGACTTCCCATGGCGAACATCAACCAAATCCACCGTATCACCGCTTCCTGCTTGTTGTTGGCACCAACACACCTAGAGAGAGATTGCAACGTGTTATCGTTGCCAAGCTTCATCACACCCCATCAACAAAGCAGCTCCGACTCCACCTAGCAGAAACAAACAAGGCCGTTGGCCGCCTGCTACAAAGCAAGAAGCAGATCCAATAAGGCAAAGGCCTCGCCGAACATTCCCGACCTATTGAGCGTCAGTGTGAAGGTAGAAGCCACGGAGCTAACGCCAATTCCACGATCGGGCATGTGCATGAAAGGTTCTCCAAACGTCACTCCTAACAGAGACACGATGTGAATCACCATTGCCGCTTGCCCGGAACAGGGCAAGGTTTTCACCCGGAGCACAGTGGTAAAAGAAGAAGAGGGAACACGTCGTAACGATGCCTCAAAGGAGGGGAACGGCGCCGAAGGCGTCGCCATCGTCAAGCCGGCCATAAGACATGGCAAGGCTTTCGCCTACGGTTCCCTATCCTCTCCTACACACTGAACCAAATCGCATACGACCGTCGAAGAATGCCGCACTGCCCCGATTCACTGACCCTCCGACGAGGCGACGAGCCGACGACCGAGCACCCCGGAGCTACCTCACCGACGGGCGCAGCCAACCACCTCCACCATCAGCACTGGGCGCCGGCGCCGCAGAGCCGCCCCGGCTCCTCGCCCGACGCCGCCACCACCAAGCATGCCGGTGGCATTCGTCAGCCGCCAGCCTCACCGTCGGCCAGCCGCGTCGTCACCCGCATCTCCCGCGCCACCTGCCGCCGCCACACGCGCCAGACGTCGCCGTCACGCGCCGCACCGCCGCTCGGCCTTCCCAGCGCGCCGTGTCACCTCCTCGTGCCGGAGACAGGCTGTCGCCGGGGTTGTGGCCCCGCCGCTGCCATCCCGGCGGGCCGCGCAGTTTCCGGCGGCGCGCTCCGGCGGCGGCGAGGCGGGGGAGAGGAGGAGAGGGTGGCGGCGGCCGAGTGTTAGGGTTTCGCCCCCGGTCGCCCGCGCGTGGACGATAGGGGGCCGTCCGCTCCTCCACTTGCTGTCTATTTACCATTTATCATTCCAAATGGATATAAAGATGCCTTTAGCGCGCGTACGTACGTGTTGTGCGTGTAACAAGGAGTTCTGACGTTAAATTAATCGCACGGGCCTGCATGCCACTGGCGAGATGTCCGTTCATACGGCTAACTTAGTCACCACAGTGCACTCCGTCGTTGCTGCCTGCAGGTTGACCCGCCTCGCGGCCTCGATGGACCGCAACTAATCCCCCCATTCCCCCCTACCCCACGTTTGCCCCCGCACGAATTTCAAAGCAACCTCCCCTCGCTTCTCCGCTGCCCCCGCGTCTAAAGTCCAAGCCGCCGGGGTGGCCTTGCCGTCCCCTTATATATACCCGTGCCCTCCGGCCTCCGCTTCCC

>Os02t0623932-00 2000 bp upstream sequence

TTTAGATATTTTACAGTGCTCAATCTGAACAGAAACCTTTCCATGTCTCCATGATATAGCAGATTAAGCGCAACAAAAGCAGGCAGAATTACAACCATACCAGAACACGTACAGTAAGGACGATGCTTCTTCCGTTGATGGCCGTGCTGATCGCCTCGGCATGGCCGGCGGCAGCATCGACGACGACGGCTGCTGCACAGCCAGCCGCCGCGTGCCAGCGCCGGTGCGGCGACGTGGACATCCCCTACCCGTTCGGCATCGGCCGCGGCTGCTACCTCTACACCGGCGAGGGCGACGTCACCTTCGGGCTCACCTGCAACCGCACCGCCGACGGCAGCTACCGCCCCTTCTGCTGGGAGTACGAGGTCCTGGACGTCTCCCTCCGCCGCGGGCAGGCGCGCGTCCGCAACGACATCAACCGGTGGTGCTACAACGCCACGACCCGGTCGATGGACGCGGAGAGCACGTGGTGGTGGGACGTCTCCGACTCGTGGTTCCATGTCTCCGACGAGGGCAACCGCCTCGTCGTCGTCGGGTGCAACTCGCTCGCGTACGTGACGTCGGTGAACGAGACGGAGTACATGACCGGGTGCATGGCCACCTGCCCCAGCGTGGGGCGGCTGGAGAACGGGTCGTGCTCCGGCATGGGCTGCTGCGAGGCGGCCATCCCGAGGGGGATCAACTCCTACGTCGTGGGGTTCGAGGAGAAGTTCAACACCACCTCCGGCGCCGTCGGCCGGTGCAGCTACGCCGTGGTGGTCGAGGCCGCCTCCTTCGAGTTCAGGACGACGTACGTCACCACCGGCGACTTCGTGGAGTCCACCGGCGGCAAGGTGCCCCTGGTGCTCGACTGGGTGGTCGGCAAGAAGACGTGCCGGGAGGCGAGGCGGAACGCCACGGGCTACATGTGCGTCAGCCGCGACAGCGAGTGCGTCGATTCGAGGAACGGCCCGGGCTACCTCTGCAACTGCTCCGCCGGCTTTGAAGGAAACCCTTACCTCCTCGACGGATGCCAAGGTAAACTACTCGTATATAATTCTGTTTCAGAACTTCAGATAAAGATTGTGTATTTGTTTGGTTTTCTGAAGAAGATACTAGTAGTGAAAGAAACAGAGTAATTTGCTTTCTGCAGACATTAACGAGTGTGAGGACAGCAGATTCAAGTACCCGTGCTCTGTTCCTGGTACCTGCATTAACACTCCAGGTGGATTCAGATGTTCTTGTCCTGATAAAACAACGGGCAACGCTTATTTTGGCACATGCGAGGCCAAGAAATCTCAGCTCGGAGTTCACATCGCAATTGGTACGCCATACGCTCTCTAGTACATTTTGACTTGTAATTATCTACCGATCATGTATGTGTAGAGCTCCGTAAATTTCTTTGTAACCACCCCCCCTTTACTGCAGGTGTTAGCATTGGCATAGCTCTACTAGTAATCATCATGTCTTCTGCTTACATGATCCAGCAAAAGAGGAGGCTTGCCACTGTAAAAAGGAGGTACTTTAACCAGCATGGTGGTCTGTTGCTATTCGAAGAAATGAAGTCAAATCAGGGACTATCCTTCACAGTGTTTACCAAGGACGAGCTAGAAGAAGCAACAAACAAATTTGATGAGCGAAATGTGCTCGGGAAGGGAGGCAATGGCACTGTCTACAGGGGTACTCTGAAAGACGGTAGAGTGGTTGCGATCAAGAGGTGCAAGCTAATCAACGAGAGGCAGAAGAAGGAGTTCGGCAAGGAAATGCTCATTCTGTCCCAGATCAACCACAGGAACATCGTCAAGCTCCATGGATGCTGCCTAGAGGTGGAGGTCCCCATGCTTGTCTACGAGTTCATCCCGAATGGCACCTTGTACCAACTTATCCATGGCGGGCGACACGGGTCGCGCATTTCCTTCGCGGCGCGTCTGAAGATCGCGCACGAGGCAGCCGAAGCGCTTGCTTACCTGCACTCGTGGGCTTCACCCCCAATCATCCATGGAGACGTGAAATCGCCCAAC

>Os02t0696500-01 2000 bp upstream sequence

CGGTTTCACAGGTTCTCTTACCCCATACTCACATCTACGATGTCTAAATACTCCCACTAATACCCACACCCATCGCGGGTATAAAATTATCCCCATACCCACACCCGTGCGGATAACGGGTATCCAACGGATACCCATACACATATAATTGATTTGTAGTATTTATAGTTTTGTACTATAATTAGCCATATTAGAGTTGTAAACCATCAATATTTTCTCTAGCAACAATGCTATTAATATTAGTGTTAAAAGGCGAGTAAAAAAATTATACAACCACATTACATAATTAAGAAATCAAATAATAATTTCATATAGCTAGACTAAGCAATACTTTAAGATGTGAAAGGAGTTTTATGCTATAATTTAGTAGTCGTCGAGAGTTATATTGCTCATAGTTTTAGGCTCAATGAACGGGTTTTTTCTACCCACGGGTTGGTGGGTACGGGTGGGTAAAGAACATACCCATATCCACGTATCTAATGGTTAAGAGTTTTCGCCCGTTAATAGACCCATAGATATAAGAATTAGAACACAATTAGTTTCTAATAGAGTAAAAACTCGTCGGGTCTTGAGTTATGGGTACCCATTGCCATCTCTTGGTGCAACCGGAGCAAAGGTCGAAGCGGCCAATGGCGAGGCGAGGGCGCGAGACCCCGCGAGGCGTCGCCGCTGTAGCAGTTTGACGCTGGCCGCAGCCGCCTGCGTGCGCGTGCTTGTGTCTAGGCGTTTTTGGGGACGGCGCACATGAGGGACGGGGACCCGTGTGTCGCACTGTCTCGTCGAGTCATTGGTCGAATCATGTGTATGCATGTCCTCTTTGCGGCCGTTTTATATTGGGTTCTTCCCTTCCGGTCTTGGCGGCGGGTCGGATAATTAACTCCGTATACTTGATTTAGCTTTGGGCCATGTCGTTCAACTGTTCGAAGTGTATGCCGGTATGCCGCTGACTGAGCACCTGCATCTCCATGTGATCGATCGGTGGTTGGCAGAGCTCGGCATGGCCCGTGGGCGGCCGAGACGATCGGAGATACGTGTTGCCTTTTGCGTTGTTACTGCTATACGCGTACCCCATCCACATCCAGCCGGAGACCGGGAACAAAGGCGTTTTTACAAGGCTTCAGCTACGTCGAAATTAGAAGGGCTGATATCTACTTGAATTTGAATTTGAATTGGTTTGGTTTGTTTCGGTGACCAGCGTCCACGGCTCAAAGCGCTCTCTTGCCTGGCACGGTGGCGCATGCTGCACGGGAGCAATAGCAGGAAGCAGCAGGACAAACGGTCAGGAAAAGCAGAACGCCGCGATTGCTGTACAGCTGGTGCATGGCTGGAAATGTTGTACTACAACGCGCCATACCGGCAGGTGAAAAAGCGAATCGGACGGAGTAAATACGTGCCCGATTAATCCGGAGGTCAGGACAACGCAGCTTGCTGGCTCAACCGCTCACGCACGTCACCTTCGGAGTAACCCAAGTTATGGCGCGTTCCACGTAAAACATCCAAAAACCTCCGCCCCTTTTTCCCGGCTTCCCCCCAAACTGGCAAACGCGTCACATGAGTTTTACCTACCAATGTTTGCATTATTGGGTGTACATTGTGCAATACCGAATTTCTGACGAGTTGAAACAGGACAAGGCAACGGGAAACCCCAAGTAGCTAGGTCCATGCCTCTTCCCCCATCACCAGCAAAGCTTAGCTGTTGGTTACCCACCTTCTCCGTTCCCCATTGCCTGGTTAAGCTGATAAACCTCTCTGGCTCTCAATCTCATCCTTTCGTCTATTCCTTGCAGTCTTGCTCTGATTAGCAGTGTGGGTTTTTTCGCACAGGAGGGCCCTACGCAGCACTAGCACAAAACCAACTCAGCATGTGCAGTGCAACCTAGCGAAAGTGGCTGCACACATCAGATTGGAGTAGGATATATGGAGGAGAGTATCTATCTGTGCGGGAGGCTCCTACCAATAGTGGTACCGCGTATATAACACGACAGCGTGGGTTCTTGGGC

>Os02t0705400-01 2000 bp upstream sequence

GCTGCAGTGCTGGTGCCTCAACTTCCGAGGGCGTGTTACAGTAGCATCAGTCAAGAACTTCCAGCTCGTTGCCTCAGTTGATCCTTCCCTTGGTATCCCTGCGGCAGAGCAGGAGAAGGTCATCCTCCAATTTGGGAAAATCGGAAAGGATATATTCACAATGGATTATAGGTACCCGCTCTCGGCGTTCCAGGCATTTGCGATCTGCCTGACTAGCTTTGACACCAAACCGGCGTGCGAATAACTGAAGAAAGGTATCAAACCTGTGTCTTCTCTCTATTTAGCTCAGGCAGCGTCTGATTCCGTTGGATGCTGTAAAATTCTCGGTCTTGCTATGCCAAAATGAGCCTACTCCTGGGGGAGACAGTAGCTTTGTCAGATCAAGATGAACAATGGCATCTATATCTTGGCAACTGGCAAAACCCCAGGAAAGATTTGAGTGACAATGCTGTAACCAACAAACTGCTATTGTACTATTGTTCTTGGAATGTTTAGCGGTGAAATATCACTGCGCTATGGATAATATGGACGCTGTTCATCGTGCCCTACCTCCTTCCTTGATGCTATTTACAGTGAGGTTGGCGATTATGATGCTAATTAAGGAGTATCTCCTGTGATCCATCATTTGAGCCTGTTACAGTTACAATTACTCCTGCAGAATCATCAGTAGAAAGTTAAACTCTGCAATCATCGTCAGTTTCGGCCCTGTTTAGATTTCGGCTGTGTTTAGGCTGTGTTTAGTTCAGCGCAAAGTTTGGATTTTGGTTGAAATTGGAGATGATGTGACTGAAAAGTTGTGTGTGTATGACATGTTGATGTGATGGAAAATAACTGAAGTTTGGATCCAAACTTTGGATCTAAACACAGCTAGATCCAAACTTCCAATTTTTTTTATCACATCAACCTGTCATACACACACAACTTTTCAGTCACATCGTACCAATTTCAACCCAAACTTCCAACTTTGGAAAGAACTAAACACAGCCTTTAACTTTTATTCTTCGAACTTCTAACTTTTTCTACATACACAAACTTTCAACTTTTTTGTCACATCGTTCTAATTTCTTCTAACTTTTAATTTTAGCGTGGAACTAAACACACAGCCAGAATGCATATTGGCAGTAAACTGTTGCTTGACACTGCCTCGTCGTCTGTTCGTCGCCGGCGGGCGGTGGCGGCGGCGGCGGCGGCTAACCGAGCGGTGGCTGAATCTTCTCTTCCTGGTCAAAGGCCGGCGACTGGCTGCACGTCTGCACGCGCGGTGGGGGCCAGCCGCCAGCGCAACGGCCGCCGGCCGTCCGCGTCAGCCGGCAACATCTGCGGCCCGTGGCCGCGCTCTTCACGTGGCGTGGCGACCCTATCCATGGTCCATCATCTCCTTCCTACTCGTACACGCTAGGCTTTGCCGCTGCACCAAGCATCTCCCAGCTCCGAAGTCCATGTCGCCATGACGTCACCACATGGCACGGTAGCATACGGAACAGTGGACGACGACGACGGCGACCTCCAAGCATCTCCGATGTCCATGTCGTCGTGACGTCACCACACGGTGAGTTGGTGACATACGGAAGGGAGGAAGACGAGAGTAGACGACGACCTCTGTACAGGCCGGCCTTCTAGAAGCAGCGCTATGTACTGTATTCCTTTTCTTCCATAAAAAAAACTAACTTTTGATCCTGAGTTTGAAATCAAACTCCTTAGTCCTTAAAAAAAGTTTATGTATCTGAATATGCTGATATCCAACTAAAATCCTTTAGGCATGTAATTACGATTGGAAATAAGAAGTTGGTTTTTTATTAATGGCTGAGGGACTACTGTATTTGCGCACGTGACCTTCTAGAAGGTGAGACCTGCACAAGAGGTGAAATCCGAACGTTCAAACAGGAAGGACAAGTCCAGTCTACAGAGTCACGCGCACACGAAACCCCGGGAGTTAATGCGGCAGCGGAAACCGGAAAGTGCCCGATATATACGGGGATTCGCGCGCGGCGCCGATCTGA

>Os02t0705400-02 2000 bp upstream sequence

GCAGTGCTGGTGCCTCAACTTCCGAGGGCGTGTTACAGTAGCATCAGTCAAGAACTTCCAGCTCGTTGCCTCAGTTGATCCTTCCCTTGGTATCCCTGCGGCAGAGCAGGAGAAGGTCATCCTCCAATTTGGGAAAATCGGAAAGGATATATTCACAATGGATTATAGGTACCCGCTCTCGGCGTTCCAGGCATTTGCGATCTGCCTGACTAGCTTTGACACCAAACCGGCGTGCGAATAACTGAAGAAAGGTATCAAACCTGTGTCTTCTCTCTATTTAGCTCAGGCAGCGTCTGATTCCGTTGGATGCTGTAAAATTCTCGGTCTTGCTATGCCAAAATGAGCCTACTCCTGGGGGAGACAGTAGCTTTGTCAGATCAAGATGAACAATGGCATCTATATCTTGGCAACTGGCAAAACCCCAGGAAAGATTTGAGTGACAATGCTGTAACCAACAAACTGCTATTGTACTATTGTTCTTGGAATGTTTAGCGGTGAAATATCACTGCGCTATGGATAATATGGACGCTGTTCATCGTGCCCTACCTCCTTCCTTGATGCTATTTACAGTGAGGTTGGCGATTATGATGCTAATTAAGGAGTATCTCCTGTGATCCATCATTTGAGCCTGTTACAGTTACAATTACTCCTGCAGAATCATCAGTAGAAAGTTAAACTCTGCAATCATCGTCAGTTTCGGCCCTGTTTAGATTTCGGCTGTGTTTAGGCTGTGTTTAGTTCAGCGCAAAGTTTGGATTTTGGTTGAAATTGGAGATGATGTGACTGAAAAGTTGTGTGTGTATGACATGTTGATGTGATGGAAAATAACTGAAGTTTGGATCCAAACTTTGGATCTAAACACAGCTAGATCCAAACTTCCAATTTTTTTTATCACATCAACCTGTCATACACACACAACTTTTCAGTCACATCGTACCAATTTCAACCCAAACTTCCAACTTTGGAAAGAACTAAACACAGCCTTTAACTTTTATTCTTCGAACTTCTAACTTTTTCTACATACACAAACTTTCAACTTTTTTGTCACATCGTTCTAATTTCTTCTAACTTTTAATTTTAGCGTGGAACTAAACACACAGCCAGAATGCATATTGGCAGTAAACTGTTGCTTGACACTGCCTCGTCGTCTGTTCGTCGCCGGCGGGCGGTGGCGGCGGCGGCGGCGGCTAACCGAGCGGTGGCTGAATCTTCTCTTCCTGGTCAAAGGCCGGCGACTGGCTGCACGTCTGCACGCGCGGTGGGGGCCAGCCGCCAGCGCAACGGCCGCCGGCCGTCCGCGTCAGCCGGCAACATCTGCGGCCCGTGGCCGCGCTCTTCACGTGGCGTGGCGACCCTATCCATGGTCCATCATCTCCTTCCTACTCGTACACGCTAGGCTTTGCCGCTGCACCAAGCATCTCCCAGCTCCGAAGTCCATGTCGCCATGACGTCACCACATGGCACGGTAGCATACGGAACAGTGGACGACGACGACGGCGACCTCCAAGCATCTCCGATGTCCATGTCGTCGTGACGTCACCACACGGTGAGTTGGTGACATACGGAAGGGAGGAAGACGAGAGTAGACGACGACCTCTGTACAGGCCGGCCTTCTAGAAGCAGCGCTATGTACTGTATTCCTTTTCTTCCATAAAAAAAACTAACTTTTGATCCTGAGTTTGAAATCAAACTCCTTAGTCCTTAAAAAAAGTTTATGTATCTGAATATGCTGATATCCAACTAAAATCCTTTAGGCATGTAATTACGATTGGAAATAAGAAGTTGGTTTTTTATTAATGGCTGAGGGACTACTGTATTTGCGCACGTGACCTTCTAGAAGGTGAGACCTGCACAAGAGGTGAAATCCGAACGTTCAAACAGGAAGGACAAGTCCAGTCTACAGAGTCACGCGCACACGAAACCCCGGGAGTTAATGCGGCAGCGGAAACCGGAAAGTGCCCGATATATACGGGGATTCGCGCGCGGCGCCGATCTGACGC

>Os02t0795800-00 2000 bp upstream sequence

TGCGCGGCGGCGGCGGCGGCGGCGGCGGCGGGCAGGACGACTTCTTCGACCAGATGCTGTCCACGCTGCCGGCGGTGTGGTCCGAGCTCGGCTCCGGCAAGCCCGCCTGGGACCTCACGGCCGGCGCCGTAGGAGGAGGAGGAGGAGCCTCCGATGACCACTCCGCCGCGGCGTTCGACGACTCCGCGCTCCTCGCCTCCCGCCTCCGCCAGCACCAGATCGACGGTGGAGGCGACAAGCCCATCATGCTCCAACTCAGCGACCTCCACCGTCATCACGGCCTCGCCGCCGGCGATGACAGCGGCGGCGCTGCCGGGTTCCTTCCCCTGTCGCTGTTCGCTGACCGGTCGCAGGACGACATCGACGCCGCCTTCAAGTCCCCCAATGGCGCTGTACGATTCTATATTTCTATCGCTTGCTATATAGCTCAACGATTACAACTTGAAGGGTGAATTGAAGCTTCGGTTTACTTGAGCTATATATGGTGTTTCTTTGGGTTCTCTTCTTCGCAGAGAGGTGACCATGCGCTGTACAATGGGTTCGGGGCCGCCGGAATGCATGGCGCCGCCGCCATGCAGCCGCCGCCGTTCGGGCAGGTACGCGGCCAAGAAGCGGTTCAACTTCTCCCCCCAATTCATTCTGCTTCTTTACCAAGGTTGGAAACAACTCAGGAATCTGGATTGTTTCCTTGATTTTTGCTCAACGGAGTTTGGTTTGCTCCCTGTTCGATCAATTGCTCCACTCGTAAGAATGCACCTCTTGTTTTCCTCATGTCAAAGGGAGGATCAATGCCGGCGCAGAGCTTCGGTGGCGGAGCGGCCGCGAGCGGCGGCGGCGGCGGCGGGTCGGCGTCGGCGGCAGCGGCGGCTGGGGCATCGTCGGGCGGAGGGGCGGCGGCGCCGCCGCGGCAGCGGCAGCGGGCGAGGAGAGGGCAAGCCACTGACCCACACAGCATCGCCGAACGTGTATACCACTCTCCCACCACCTTTCCCTTCTCGCCCCCATTTTTTATTGCCTCCATGCCATGTTGTGTGAGTGTGTTCTTCTTGCTCCTGGAGCTCCACAAATTTTGGACCAACCTAACAAGTCAATTTGACCTGTTGCACACTTCAAATTTCGGCTAGAAATGGTGGTTTCGGTAGTAAGAAGAGCTTGAAATGAGATGGAACTTGTTGTGTTCTTGTGATTTCCTGAGACTTTGCATGTGTTTGATTTGCAGCTCCGGAGGGAGAGGATAGCTGAGAGGATGAAGGCGTTGCAGGAGCTGGTCCCAAATGCCAACAAGGTAGCTGAAAATTTTCCTCACTTTTGCTCCTCTTCTTTTGCTTTGTTTTACTTCTTTGCGCCATCTTTGAACAAAGTAGGAAGCTTTCCTTGAAATGCGATGAAGATGCACCCATGCTTTCTGCTTTTTTTTTTGTGTGTGTAAATATCTTATCTTAAAACTTAGTGACCAGCTAAGTGTGTTGATTTATTGCAGTATTAACACATATGTTCTGTTTCTCTTACAATTAACATGATGTGATATCTTCATTCTTCACTGTATGATTGGAAAATAGTAAGTGCATCATAGGGTAGGAAAGAGGGGACATGAGGCATATGATGCATTGCTGATTTGATGTTCTGATGCTTACACTGACACATGTTGGCCTTTCATTGGAGTATTTGGGTTGGTGATTAGCTGCTAACGGTGGTGAATGACACGTGTCGGGCTTTCGTTGGATGATTTGGGTTGGTGATTAGATGCTAATTGATGGTGAAGGCAGTAGTGGGGAAAGGGAGGTCAGACATGATGCCGAAATGAATGAGAAATGTTTCTACCCGTTAGGCCACCAACAGTAACAGATGAATAGCCACAGCTCAAATTTAGGGGAATGCAGATTAAACTATGGACTTGATCATTGCATTAGCCACCAGGTTCCTAGAGAACTGAACATCAAGAAAGGAATGGATTAGTACTAGGTGTTATATAATATATGCACATGTCTTAACTTCGAGAC

>Os02t0805100-01 2000 bp upstream sequence

TTCCCTCTCCCTTCTCCTCCAAGCCATTAGTGGTTCGTACTTAGTACTTACGAGTAGCTTAAGGAGCAGCTTAAGATATAGCAGGAGGAGCACATTGCAGTTGATTACTAGTTGGAGAAAAAAAAAGCCGGATTAATATGTGCAAATGAAAAGCGGTTGATTAGTTGTTTATGTCAAGTCATGGATGGCAACGAATATCTCGTGCTAGGAGCATGTGCTCGATTTTAATAATGGTTGTGTCTCGAGGATTATTGTAACGCGGGCTGCCGCCGAATGGGCGAAGACACTTGGCATATCTCTGAAATTTTGGCTCGGGGTGTTTCTCCCTTTTTAAAATAAAATCTTTGGTTCCGAGTTAAACTCATCGTTTCTTCTGTAGCTGGTCATAAGCTGTAACGATTTTTATGAACAAATAATTTACAGTAACATTTTTATGTTAACGATTTAAAGCAAGTCATAAAAAATAAAACTATAAAACTAACTTTTAAAATTCAAATTTTAACAACAACATACAAGTTAAAGAGCAAACGGTGAGCTGTAGTAGCTCTTTTCCATGTCTGCATGAGTTTTTGTGTTTTGAGTAGTCTATATCGGCCCTGTTTGCACCCGTCGAAGATTGAAATTAAGTACGAGCATCATGTAAAACGATAAATGAATTTCAATTATTTATTAATTTGGAAAACGTGTTATCAAAATCCATATAATTTTGTATTTTAATCAGATTCAAACGGGGGGCATCTGTATTTTTCATGCCAATGTACACGCTTGGCTTGCATGTGTCCTGCATCAGCAAGGCAAGAAACGGGTCAAAACGGAGGATTCCACAGCAGGCTGCGCTACAGCTAGGAACACTGTCAAGCAGAGCAGAGAAGCAGCTGGTTTGTTGATGGATCGCGCGCGCGCCACGTGCTTGACGGCGCGCAGGAAACAGCCACGCCCACGGCCCACGCACAGGCGCACAACCACCTCCCCGGGAGAGGCGAGGCGAGGCCACATGCCCGGCCGCGCGCCGCCTTCCGCGCCGACGGCAACATCGCGCGCGCCGGCCGGCTGGGCGGCTGACAAATCCTGGCTGCCGCATCTCATGCATGCCTAGCGCCAACGCGTCTCCCAGGCCGAACCGGTCGACCCTCGTTTCTGATTTTCAACCTGATTAGTATGAGATCTCGCTACGGTTCGTTTAAAAAAAAGTTTAACTAAGATAGTAAAAACTATGGAAATAAATGGTTTTCATGTTGTACTGATCGTTATCGTTATGGTATTCTAGTGGGAACCGTATTTCTAAATATTCTTATAATTTTTTAACATGTCTCTATTTAGCTCATAGTTTAGTGATGTATTAACTTCTAACTAAATATCTAATTTAAGGACATAAAATAGACATATATACATAGTTTGTACTAATAATTTATATAGTGTAACATTGAATTAAATATGTTATACATTAACAATTACCTGTTCCCAGTTGATACCAGCATGTTTTTTTTGTTCGTGTAGTTATATGATACGGTTTTTGTTTCCAGCTTTACATTTTCGATGACGGAGAAAAATATAGTTACGAAAATGGTATTTCCCGGCCGTTTTCATTCCAAATGCACGTCAACGCCCAACGGTGCAACGTTTCTGACTGTCAGTCTGACAGTGGGCGGCAACCTGAAGCTCTGAATTTCACATACTAGCAGCCCACAGGATCACAATCACATGACAGCAATCAAAAAAGAAAAAAAAAACAGAAACGCAAACGCTGCACAGAGGCGCAGGGAAGAACTACTGATGCGTGTGTCGGTAGGCTACAACAACAAGAGATCAAAGCGAAGCTAGACTGAATCACTGGACGGACAGGGAGACAGGTTGCCCGCCTCTGTGCGCCCCTGAACCCCGACACCCTGCCCCCCCCCCCCCCCCTCGCACCGATTCTTCCTCATCTCCTCTCGCTGTCCCACCAACACCACTGCACGTCTCTGCATATATAAACCTCATCTCGTTCCCTACTTGCAACT

>Os03t0114800-01 2000 bp upstream sequence

AAAAGCTGAATAAAATTGTTACATACTATGTCTGCTGCTCCACATCACAACCTACTTTCCATCTTTCTCATTTCTCTCTTACCCTTAAACTAAAGGAGCATCCAGTGCTGAAAATACCTAATTCATTCCTTCACCTTTCTGAACAATGATATAATAAGATCAGACGGTTGATGCTGCAGGCACCATAATCTGCTTACGTGCAGCAGCGCGTGCAAAACTAGTTCAGCCATCACATTCTCAAGGCTCTTGAGGGAAGCATGGCCCCTTAAACTACCTAAGCCAGCTCTAGATCCTGCGTGTGTACATACATGTGCAAGATCCACTGGAACTGCATTTAGTCTGTATACTTAAACAATGATCAAAACAATCTAAACCTAGAAACCAAAAACCTTGTAACAATTCACCACACATGACACAACCAGAAAAAAATTATTGCCAATTCATAATCACATTATTTATGCCAATTTAGCTTGTCTACCCATACGAAGGTTCTGTCCCAGACTAACTCTGCAAACCAAATATCTGAATCATCCACCGTAAACTGAACACACATACAATACTCAGAACTCAACATATTATGTCGCAAAAGTTAATTCGTGCAACACATCAACGATGCCATGATTAAATCTACATTTGCCCCACCACTGACGCGACTAAATCTGCATTCACCCCGGGAAAAAAAACAGTTAACCCTAAACAATATTCTACAACCGAATCGGAAGTTTCGGAACCTTACCGAGGATTGGAGGAGGCGAGCGATCCGCTTGCAGTTGCATCACTTTCGTCTCACCCCTCGGCGCACTACCAAATGAGGTACCACCTGCGGGCACCGCTGCTCCTCCAACCGTCGCCCCTTGAAGCCGCCGCATGTACATCCGCCCGCATTGCAAAGCGCGATTAGGGCATCGCCTCAAGGATCCACAAGGGCGATTCGCCGCAAGCTAGCGATGGAAATCGGATGCACCTACCTAGCAAACGCCCGCGGATGATGAGGACGCTCCGCTCCGCTCCGCCGTCCATCGCCTCCTCTCTCCCTCTCTCCGCGATTCGATCTCACCGGCGCCGTAGCGCGCGGGGCGGTGGAGAGCGTGGGATTTCTCCGCGGGTAATCCCATTGGATAAACATGGCGCGTTTGGGTGGTTCGGTCGCCGCACCACGCAACGCGACGCAGTGCGCGTGAGCGTGCCTAACCGTGTGCGACTCGGCTCGACAAAAAACCAAACCAAACCGATCAGATCGCATTAGAAAATCTCTCCACCGCTTGCAATTGCAAGCTAGCTGCAGTGGTTTAGGGCCAAACATGCTGCAAGTGTGAGTCGTTGACCAAAGAGGACATCACATTCGGAGTCAGAGATGGGAGACCGCCAGTGCAATGCTAGTCATAACCACGATATCAAATGCCAAGGAGTTAAAGAAATGATAGTTTGTGAACTTGTGAGTTGTGATGGACCGGGACGATGAGCATGGGTGAGCTAGGCATTGTCTCTGCGCAAGCACTGGTGTACTGCCGCTGCCAGGGTAGATCCACCGTCCATTGCAGTGGTCCATTGCCTCACCCTCACGCCGACACTTGTTTCACAGCGCGCAAGTAATTAACTTGATCCATTCGAGAGAGATGAGGAGAGAAAACGAACAAGGTTTGGTTGATCTGGCACTGGGGCACCGCCAATCCGATGTTCGTTCCACCGACTGTACTACAGTACTAGTATCCGGCAGTCTGGCTACGCGGTATACTACTATACGTGTCATTGTCTACTCAACCAAGAAAAATTCGCTGACGATCGAGAATTGTGTCAGTAACGCCGCCAAGCAGAAGAACACCCAAAATCCATCTATCCACCACCACTGGGACTGGATTCATCATCATCAATTGGAAGAAATCTCAACCACCGTTGGGCCCCAGACGCACAGACACACACAAACACATCCGATCCCGCGGCGGCAGCAGCAAGCGCAATTCCCATCAACCGCAAGTGCGTGCCGGCCGAAGATTTGGCGC

>Os03t0175200-01 2000 bp upstream sequence

AAAATGTTTATTAGCTAGTTAGCTAGACTTAGAGAAAAAGCATAATGTGCACAATATAAATTAATTGTGCATTATAGCTATTGGTGCCTGTGGATAAATATAGCAATCACACTTTATGCGTGACACTCTTGATACTTTGGTTTGCCACTCTTAATATTATGATAACATGTATCCTTTCACGAGTGACACAACAATATGTATGGACCGATCATTCTAACATAACTTGTAAGATTTTCACGCTCGAATTGTTTGGTTGGGTTAGCTCAAATGCCTTACTAAATAAATTTTGTCCATATCATGTTATCACGGGACACGTCCTTTACATAAATAGATAAGGAAGACCCACAGATATATCAGAAACAACTCACGAAAGCTTATCATGTTGTTCTGTACCTGACAATCTTCTTAATTATTTCTTTCACACAAAGAATAATATACGTGCTTACAAAACATTTCACTCTTTCACCACAAGTATCATCATCATCATGGATGCTTATAAAGATCAATTATACTTATATCTCATCAATCCAATATTGTAACATCCGAGAGTGTACACGTAAGAACCATCAAAGTTTCAAGTAAAAAACTGACGAATGGATTGACTCTCTGACTCTCTGTGTAAACCCAGGTAGAAATCATGATCTGGCCACGTCATGCACAGCACACAGAAAACCCATGGGCCCACACGTCGGCGACTCAGCTCGCCATTTATTGCGATTATTATCGTGGGCGCGCGCGAGGTGTCTCTGTGTGCGCAACACTCCGACTCGCGCCCATTATAAAAAGTGCGCCTCTGAAACGGTGGCCTGCTTTCCCGCGATCCAGCCAATGGGCGAGCACCCACCGTCTCACCGGCGATCCCCGGTCACCGTGCCCAACAGTAACCGATAAGACGAGCTCCCCGACAAGCACCGCCACCGTCACGTGGCAGTGGCCGTCCGGACATCTCGCTCCAGGGCGCGGGATAAGGCACAGCTCGGCCACGCCACCAGCATTTCGTATAAATATTCGCTTACCCCCTCGGGATCCCGAGAAATCGCTCGCTCTTCGCAGCTTCTCACTTTCCCCGTTTCAGGAATCCCTAGCGTTGATCTTCCTTTTTCCTTTTTGTTTTCTTGCGATCGCCGGAGTTGGTGCCGTCGGTGACCGGGGTTTTTTGGTTGTTCGCCGGAGATGGTTCATCTGGTCGTGGCTTGAGTTGTTTGGATCTGGATTTGGTAGGTGAGTAGTAGTACTAAGGTGTTTGGAAGGCGAGGGTGGAAGGATGAGCTTTGGTGTTGTGGATATGGATGAGGAGGGCGGGGCGGCGGCGGCGGCCGATGAGATCCGGCGGCTGCCGGCGGAGGTGAACTGGGAGATGCTGGACAAGTCGCGGTTCTTCGTCCTCGGCGCGGCGCTCTTCTCGGGCGTCTCCGCGGCGCTGTACCCGGCCGTCGTGGTCAAGACGCACCTGCAGGTGGCGCCGCCGCCACAGGCGGCCACCGCGACCGCCGCCGCCATCCTCCGGCGGGACGGTCTCCGGGGGTTCTACCGCGGGTTCGGCGCGTCGCTGGCCGGCACGGTGCCGGCGCGCGCTCTGTACATGGCGGCGCTCGAGGCAACCAAGAGCTCGGTCGGATCCGCCGCCGTCCGGCTCGGCGTCTCGGAGCCCGCGGCAACGGCCGCCGCCTCCGCCGCGGGGGGCGTCTCCGCCGCCATCGCCGCGCAGGTCGTGTGGACACCCGTCGACGTCATCAGCCAGCGGCTCATGGTCCAGACCTCCTCCACCTGCCGCTACCGCGGCGGCGTGGACGCCTTCAAGAAGATCCTCCTCGCCGACGGCGTCCGTGGCCTGTACCGCGGCTTCGGCCTCTCCATCGTGACCTACGCTCCATCCAACGCCGTGTGGTGGGCGTCCTACGCCATGGCACAGCGCTTCATCTGGCGCGTCGTCGGCGCCGAGCGCTCGGAGAGCTACCCGTCGCTGATGGCCGTGCAGGGCGCGAGCGCCGCCCTGGCCG

>Os03t0215000-03 2000 bp upstream sequence

TGAATAAAACTAATTTGATCCATCAGTTGACCTGTGTGTGTCTATATCCTCATCTCCCAACTTCCCCAGCCCTAATTGAGCACATGCAAGCTACTAGCACACACTATCCACGTACTCCAGTACAGAAGTACTACGCTCTAATTAAATACTACTATTAGTTAAACTCCCTGATGAGGCCAGTTAGCACGGAGAGCAGTGCTAGCTAGCCCAGAGAGAGTTAAGAAGGCCCATCTTTGGAGGGGGTTGGCATCCTACACTCTAGCTAGCCCACAGTCCGCTGCCGGCTGCCGGCCAGCGCGCGCGTGCAAGTCTGCAAGCAAAGAGTTTTCAGATGCAGATCGAAGCACCGCAAGAAAATTTAATTTTATCCCCACCAAACTACTGCCACCAAGGGTGATGAATCGCATCTCGCACCAACAAGTCCGCCGTCCGCGCGTCGTCAAATCACACCCTGCAGCGCCGCCTGCGATATGAGCTGCCGCGACGGCGCGGCGGCTGCCGGCCCGGTCGTCGTCGTCGTCCGCCCGCGCGTGCTGGCTCTGCAGCCCCCGCCGCAGTAGTGGGACCGCGCGTGCTGGGTCGGGGAAGCCCGCGCAGGCCTGTCACGCCGCCACGATCCCACGAGCCACCCACAACGTAACACGAGCCGATCGAGTCCCGCGTTTTCACTCGGCCTACATGGGCTGACGCTGCGGCCTGCTTCGGCTTTGGGCATCCCGCGGCCCATCTTCTGCGAGCCCCCCGGGAACGTGCGGCCCGCGTGATAATCCCGTGGATATGGGTCCACGGGTTTTCCTCTCCTCTTCCCATCGCATCCGCATCTCGGCATCTCGCGTCGCGACCCGCGACCCGCGGCGGCGCGTCCCGAATGGCGGCCGATCGCCCGCTCGCGAGGGTTGATGAGATAGCAAGGCGCCATTTCCAAATATTTCGATCCGCAGCGTAATCTCTCTCGCGTGCGCCGGTGCGCGATCCCCTCCCTCCCTCCTCCTCAATCCCCCAGATCGCGTGCGCCGCGGGCTCTCCCCGCGTTGACTCTCCAGCATCTAGATCCGCGCCTGCGCTCCCGACGAGGTCGGCCGGTCTACGGTCACCCTCGTGGTTTCCATTGACTTGTTTTTCTCTTCTTTTTCTTTTTTTCCCCTCTTCTCCCCAACCTCGCAACAAAATCGGCGGCTATCCTCGTGTTTCTTCTTCGCTCCGTGGCCGCAGAATCCCCCCACCGCTGTTCTTGCGGCGCCGGTGTTCGTTTGAAAGGTGAGGACTCGATCTGCTTGCCGTTTCTCCTTCTGGGCAGGGGCAGGTAGGCATGGAATGCGATCTTGGGTGGTCCATGGAGGGATTGATGGATTGATAGTTACATGGAGTTTATTGGTGTACTTAGATTGGTGGGGGATCAATTATTTCTTGAGTTCTGGTTGTGCACGAAACCTCTATTTAAATCGAAGTTTTCCTATTCATGGTGATGGGGTTGCTCCTACCTAACAAATCGATACTCCTTGCTCTGCTTATTCTCGCTTTTTATTTAATGGGCAGCATGTATTTCATGACGCATCCGTCGGCGGCGACGAACTAATTTTCAGTTCTTTATGTTATTCAGCAGTTGGCTGCGCTGTCCTGGGAGGCCTCAGCAGCAGCAGCAGCGAGCTTTGCTCTTGTGAGCGCTTCGTGTGCTTGCAGCTGCGTTTTATTATTGCGTCTTGGAGCTGTCACGACGTGGTTGTTGAGAGGAATCTATCATCCCTTCACAGTTGCGAGGAAAGCTTCCTGTGGTATGTGTTGTGCTTTGTTTTCTTGTGGGCAACTGGCAGATTGCAATGGCATTATTAGGACCTTGTCCACCTTGACTTAGACAAACTGCGAATTAGGTGGTGTGACATGTGTTGATATTTTCTTTTCCAATCCTCTGATGCTTACAACCCGGTTGTGCTTGTGACGTGCCCCTACCTTTGTTTGACTTCAATGTGTAGTATAACATGGTTGTCCTGGGCAAGCTCATT

>Os03t0221200-01 2000 bp upstream sequence

TGTTCTTCAGAACCCCAACTTATTATTTGTATTAAATTCCTATTTGCAATATCAGATTGCTTTTATGTTGTTCTTACTTGTTTCTTCGATTTGCTTGCAGGAATAGGGTTGATCTGCACCGGCAAGATCAACAACCCACGGAGAGGTGTATCGATCGCTAAGGCGCAACACAACGTCTCGTACAGTTGTAGTCGGATCGTCAACGTTTCTCCCAAATCGTAGTTATCACAACTCACCGAAAGATCGAGCCAACAACAGCCTTGAGTGTCGAGAGGAACTCAGGGTTCATCAGAAAGCAACAAAGTGATGGAGTCCTCGTACGCAAGACTACTGATAGTTCCTAGGTAGTTTATATATGTACTACGGAGACCAGGGTACTGCTATTACACTGGCTGTGGCCTGGGCTAGTGGTTTCAGGGCTGTTGGTCGAAGTTAATTTATGTAAACTTTGTTTTGGCTGACCCATTTGCTGCTGATATGGGCACTCGATGAAGGTTTCAGAGGCTGTTGCTTAGGTAATTAAGGTAAGCTTATTTTGGATGAACTGGTAGTTGCTGACATGAACAGTTGAACATTTCCGCCGGGCATGCTTTATTGAATTATTGACGGTCAAAATAGCTGTTGCTAGTCCGTTTGTCTTTCCCTTGGTCAAAATTTGATTGTCCAGATTTGCTGTATTTTTTAAATTGCTGGCAAAGATGCTTATATTGATAAGTGTCCATCTTGTGGGTGGAATTAAACATGGTGGTATTACTTAGTATAAACACGAGTTAGCAAATACTCCCTCCATCTCGATTTGTATGATGCCACAAGCCAATGGAGTGAACAATCTAAAATGAGTCAAAATGTATGATGCCACATGGAGTGAACAATGTTCCACCATCTTTCCCTTCTCCTTAAACCATGGCCTGATGGCGTGATGCGGAGCCGTTTGCATTTGCATGTCGCTGCCGTGATTCCTGAGGCCACCTCGAACCGCCTTATGCTCATGGCTCCTCGCCACCGTGGTGGCAGTGGTTGACGGCCTCATTGGTATCATGTGATGTAGCCGCCGTCCTCGTTGTACACGATAGAGTTAAGCTGGTGCTATGTTTGAGAGAGGCCATGCTACTGTTGCACTTGCATCGCTTGTGCCGCCGTCGAGAAAAAATCGTGCGCGTTGGCGGCGGCTCCCCAATGTTGTGTTGGGACTTGGGACAGTGGGCTTTTGAAGGGAAAAAAAGTACGAGGATCGTGACAATTAGGATAGTTTATACTCCCTCCGTCCCACAATATAAGGGATTTTCAGTTTTTGCTTGCAACGTTTGACCACTCGTCTTATTCAAATTTTTTTTGTAAATATAAAAAATAAAAAGTTGTGCTTAAAATACTGTAGATAATAAAATAAGTCACAAATAAAATAATTAATAATTTCAAAAATTTTTGAATAAGACGAATGGTCAAATATTACAAGCAAAAACTCAAAATCCCTTATATTATGGGACGGAGGGAGTACAATTTTGAATGTACTCCAATTACTATATTAAAAGTTGAGGAATCAAGCCAAGCAGAAGTGTCCATATTGAGTCATTTGTCCATTTGCCCAGGCAGCCCAGCCAAGGCAAACCAACCAGCTGCCGCTGCTGTAAATACTCCTGCAACAGTATTTGTTGCATCACGTACGGATAAATCAAACGTCTTTTTTTTTTCATATCTCTCTCTGCTTACCCATGCGTACTCATGCCGTTGTCTTCTCGCAGTTGCGCTGGTCCAACCGTGGAAACGAACACAGTGAATTCGAATTCACGGTTCGTTCCGTCCTCCTCCCTGGACGCCAACCCGGTTTGCGACCGCACCTACCCCAGGAACAAGTCCGCGTACGTCCGCACCCGCCCGCTGCATGCCACCTATGTCTGCGTTATTGCTTCTCCGCCAAATTTAAGTCTTCCTTTACCTACCTTCTCCTTGCTCCAATCGATCTCGCCGGTATATATACTGCTCCATCTCGTCGCTCGTCTCTC

>Os03t0241300-04 2000 bp upstream sequence

CAACCTCCCCCTCTCCCTCGCTTACGGGTGGGTCCCACACCACGGGCCAATCAGGGCCCGCCGCTTCACCACCCCCGCTGCGTTGCCTACCAAAGCCACCCGCCTTTCCTTTACGCCCACTGACGTGTGGGCCCGAATCTTCCCGGTCCCGTGTGCCAGTGATACCGTCTCGAGCATCACCTGGTAAATGGAGGAGGCCCCCAGTTGGTTCCCCGCACGACTCGGGCTCCTCCTCTCCGGCTATAAATATCGCCTCACCCCCCGCGCCCTCGAAGAGGCTCCCTTTTTTCTCTTCTTCCCCTCCCGCAACCCTAATTTTTTTTCCCCATCCGCGAAACCCTAGCCGCCACGCGAAACCAAATCCCGCCGCGCGGGATCCTTTTCCGCCGGATTCCACCCGCGAATCGGGGTTCCCCTTACGATTCGCGGGCGGATTAGCGCGAGGCGCGCCTCCCCCTACCTCTGTGTGATCCGGGGGTGAGGTTAGGCCGGACGCCGGGGCATCAGCCATGTCGAGGTGCTTCCCCTACCCGCCGCCGGGGTACGTGCGAAACCCAGTGGTGGCCGTGGCCGCGGCCGAAGCGCAGGCGACCACTAAGGTTTGTTGAACCATCGGATTTACACACGCACGTGCCGGATCATTTGCTCTTGCCTGTTGGTTTTGATCGGATCTGTTGGTTGTGCGTGTGTGATTTGGGGATCGCACGTGCGGGGAAGCTAACCTTTGCATGGATAACTTGAGATTTGTGAGGCCGCGCTTCGACCAGATCGGTCGCCAATCTTTTAGTGGCTGACCGTGGAAAGAGGATATTACTGACCTTCGGTTTGCTAATTTTGGTTGTGCCGTTGAATCTGAAATAACCAGAATAGTCATGGGGAAAAAAGTCTGATCTGGAAGGTTCGAATTACATTTCTATATATTGTTGTGCTCCCAGACGATGGTTGCAAGAAATCACTCATGCTGGATAAAATTGTGGATGTAAGAGTCTGCAGTCGTTAAAATCTGGAAACAGCACATTTTGCCGTAGTAAATTTGAATCCATGTTGCTGTCTCGTTATTGGTGTGTTACGAGTAACCTGTGTGTTGTTATCTCCGCTTGGACTAGATTCCAAGTAATCCAGTGCCTTCATGACCTGCAAATTCTATGCCTATGAAGTAACATGAACAGTTTGTATGTATGTATTCTGTTGATGCATACTTGCATTATTTGTGAGATGTACATGTTGTGGTAAAATTTTGCATTCACCATATAGAAATAGTAACTGACTATCCTTGTTTAGTTCGAAAACTACTGCAGGTTTAGTTATTCTCTGTTGCCAAGAGTGCTTGTTATGATTGTAAGGGTTACAGTTCTGTGACTAACCATGTAACAAATATATTAAGGATTATCAAATTATTCTATGTGAAGTGTCCGTGCCCTAATTGTGTTATCTTCTGTAACTGATAGCACAACATTTGTTTCCTGCTGTGTGCTTGTGTAAATTGGTACTTCATCATTACTATATATTTCAAAGAAAATTCTGCATTGCATTCCCGTCGTCCGTTCTAAATCAGAACTGACGATTGCTCTGGTGGCTGAAGCTCCAGAAAGAAAGGGAAAAGGCTGAAAAGAAGAAAGAGAAAAGGAGTGACAGGAAAGCTCTTCCACATGGTGAGATATCCAAGCATTCAAAGCGAACCCACCACAAGAAGAGAAAACATGAAGACATCAATAATGCTGATCAGAAGTCCCGGAAGGTTTCCTCCATGGAACCTGGTGAGCAATTGGAGAAGAGTGGACTCTCAGAAGAGCATGGAGCTCCTTGCTTTACTCAGACAGAGCATGGCTCTCCAGAGAGTTCACAGGACAGCAGCAAGAGAAGAAAGGTTGTGTTACCCAGTCCTAGCCAAGCTAAGAATGGTGAGGCCCTTTCTTGCATTTGTCTTCTTTTAGCTGGTGATGTTGAATTGGTTTGACTTATCCTGAATTATCATCTTGCAGGTAACATCCTTCGAATAA

>Os03t0241600-01 2000 bp upstream sequence

GGGTCAGGATCATCTCGCAACAGGAAAAAAAAAGTCAAGATGGAAACGTACCCTTTGTTGGACAAAATACGGGCAACAGATTATATGCCACGTTGGATTTGAACATTGTATTGTTATGCCTAGGCCGGTCAGCGTCAGATACATAGAACCTACAGAAGCGTGATCAATACTCATGACATACATGCTTCATTTTTTTTTAGGGGTGACAGCATTTTGCGTTGATCAAAGAAAGTGTTTGCAGTTAGAGATGCAAGCGGGATGGGCAAGCGAGTTTTTTAATCCGCTTATCTCACTTCTAATTTACTTTTTCTTCTAAATTTTGAACTATTACGTAGAAAATGAACTAACAAGCGGGTTTTTATGTGAGTAACGGGACTATCCACTTGCATCACTATTTGCAGTTTGCAGTTGCACCTATAAAAAATTTGCACTACCACCTTGATGATACCTGTGTTGTTTTCGTGTGCCTAGCACCTTTGATTATACCCACTTCTTTTTGTCTCTGCAAACGAGTAATTCGTTACGGAGTAACTAACAATTGAAATTAGAAATACTGATAAGTTTGGTCCTAAAAAGAGTCCCCAAACCCTACCGATCCGAGTTGTCCCCGTGTTTTTCGTTTTATCTCCACTTCCGTGGATGCGTCGAATGACATGAAAATGACGTTTCATGGACCGCCACTTGTACGTACTACTCACCATTATTGATCGTGCACAGATCCGAAAACTTTCAGATTTTTAGATTAGAGTTTCACCGTGACAAGCCTCCCACATCTGCAGATACCAGCAAGACGTAACAATAACGGTTGCATTTGGGACCCTCATGGTTGAAGACTTGAAGTCAGCCTAAATGTCTATTGCTAAACCAATACCAGTCCTAATCAACCAGTAGATCAATTAAAACGATCACCATTAAGGGATGAGCGAATAAATCCTCCTCTAAGTGCATCGAATTTGCATGAGATACGGCAGTAACCCGGTCTTCAGGCTTCATTGTGCTACACTAATTGTGCATAAGTATAGCACAATTCTACCCACAGAAGCATCTCCCCTTTTGCAACATTGAGACGTCCAATTTGCAAAAAGATAAAGAATACAAGGGCAATTAACGATGGTCTTTTTTAATCATGTTGTAATAAGAGTGCCTTATTGTATTTTTGTGTTTTCTTAACTATAGTAGTATAAATTGTCGAACGATAATATGTTATGTTCATGTTGTATATGTTTTCTTTTCTTCTTTTTTCTATTGGTCGTGTGCATTCTTAGTTTTTTCTTAAAATAAATAAGGTGTCTTTAGTTGATGCAGAGGCCGAATGTTTTTATTAATAAATAAAATATTTTTTTATCAAGAAAATAGGACAAAAGATCATGTGCCAATCCAATCATCATCTATCAATCCCAAGGTTATATCTATAGTTAGGTGCCTTTTTCTTTTCCGAAAGAGCACCTCGATTCCCTTTTCCTTTATTTTTGCTACTCTGCGGTGGCTATTTTGCTCCCCTTTTCCTCCCTTTCTCGAGTAGGGACTGTGTATAATGTTATGGACCGATTATTATTCACCAATGGCACTTGTCCACCTCCATCTCCAATTTATCACTTTTTCTCTTCCTTCCCCCCAATCTGACAATCTCCACCGAGAAAATCCTTTCCCCTAGTTCTCATCTCACCTTTCTTCCTCGCATCATCACATCACAATTAACAAAACCAATCGTGTGGCGCGCGCACTCGCCTGCTTCGCGCACAAAACCAATAGTTTCTTCCCGTGCTCTTCTGCGCGCCCGCTTGTCTCTGTCACCGTCTCTGTTGTCCGCTAGCCTCCGCATACCGGCAACGTCGCGCGTTCGCGCGCGCGCGCGCGCGCCAAACAGCAGCAGCCTCCCCTCGCAAGCCGCCGTGGACGGTGGACCGCGACCGGGAGGGCGACGACCGGCTCCTCCTCCTCCTCCTCCGCTGCGCGGGCGGCCTCGTGCCTTTTTAGCGCGGCATCTGCATCGGCTGCGT

>Os03t0241600-02 2000 bp upstream sequence

TGCGGCTGATCGGTTTTGGGGATGGCGGCGGAGCACGGGGACAGCGTCGGCCGGTGCATACTGGTGGGGCTGCACATGGACGGCGTCGGCAAGGAGTTGCTGCAGTGGGCGCTCAACGAGGCGGCGAGGAGCGGCGACCGCGTCGTCGCCGTGCACATTTACCGCAAATCCGGTAGGTAACCTCGCCTCTGCAAGAGCTCGCCATGAAACTGCATGCAGCTCGGTTGATTCAGTCCTAGTGAAGCAAAAGCGAGCGGCTGCTTTTCTTGACATTTTTTTTCTTCTTCTTGTCTCTTGATTTCCGGAGATAACTGCAAGACGAACACGCTGAGCCTGATCAGGACGCTGGACGACTACCTGGCAGAGTACGAGGCACTCTGCAGCAAGAAAGATGTAAGCATGGGCACATCGCCGTAGTGAATGCTAATGGAGAGAGCTCTACGCCATGATGATGAGATTAAAATTTGGGTGCTTTTATTGTTGGTCCGTGTCAGATCGTTCTTGTCGGCCGGGTGACGCCGGGGAGCTCGATCCAGAAGGTGCTAGTGAAGGAGGCCAAGCTCTGCGCCGCCATGGTGGTGGTGATTGGCGCCAACAAGAAGTACTCATTCGGGTCAGTGATGCAAGCAGCAAACTTTGTATGATCCAACTGTTTGTTTCTTCCCCGTTTCTAACTCTCTGACAATAAAAACGTTTTTTTGTGGGGGCGTGTTGGTTCAGAGGCTCGACTTGCCTGGCCAAGTACTGCGCCAAGAAGCTGCCGGCGACGACCACCATCGTCGCCATCCAGAACGGCAAGGCCATCTTCGTGAGGGAGGCGCCCAAGCCGCCACTTGGTAATTAGTGCCTCTCAATCTGAGAAGTTTTAAGATGGTAATTGTGTTTTGGAAGAGTGATTAGAGGCTCCGTTTTGGGGCGGTGGTTGATCAGGAGCAGAGCCGAAGCCGGTGCTCCGCACGGTGCTGCACCCGAGCGTCGGGTTGGAGCCCAAGGTGATCATCCCGAACCCGAACCGGAGCGCGCGGTCCATGGACTTCGACGCCATGGGCTGCGGCCACGACGGCGCCGCGCCGGTGAGCTCCTACGACGACGCCACCAAGGTCGGCGGCGGCGGCGAGAGGACGGCCGAGCAGAGGCTCGGGTGGCCGCTGCTCCGCCGCCCGCTACCCGCGGCGGACGGCGCCGTGCAGCCGCCGCCCAAGGACGACGGGCCGCGCAAGCAGTCGGTGGTGCAGTGGGTGATGAGCCTGCCGCGGCGGTCGTCCCCGTCGACTTCGCCGGAGCCACAGGCCGGGCTCGTCGCCGAGCTGAAGCGGATGCTCGACGCCGTCCCGTCGCGGTGCCGGTGGTTCCGCTACGAGGAGCTCTACGACTCCACCAACCACTTCTCCTCAGGTTCCCACATTTCATACACATATCCCCATCAAATTCTTTCACCTTAATTTCAGCCACTGATTCCTCCTCCCGTTCTTGCAGAGAATCTGATCGGGAAGGGCGCGCACAGCAGGGTGTACAGGGGCAGCCTCGCGAGCGCGCAGCCGGTGGCGATCAAGTTGTCCAAGGCGTCGGCCGTGGCGTCGAATGATTTCCTCAGGGAGGTGGACATCATCACCAAGCTGCGGCACCACCGGATCGTCCCGCTCATCGGTGTCTGCGTCGAGGGCCCCAACCTCATCTCCGTCTACTCCTACCTCCACAGAGGCAGCCTCGAGGACAACCTGCACGGTAGAATACTATACTAGTACTGCACTACATTATTTTTACAGATATCTGTACAAGATACGCATATAAAATTCTGTACGTCCATACATGTCAGTAAGTCAGCCCAGAAAAAAATCATAGTTTTCTCGGAACAGATTAGCGTAGAACAGTTCAACTCGGTAAATTTTGGAGTTCATGGAAAGGCAGTTAGGAACGGTGTCGCTGTTAGAACGGTTGCATGTTCTTCACTCCATGATGTTTGACGCTACGAACGCTGTTAATTGTTGCAGGTAAGAGGT

>Os03t0250200-02 2000 bp upstream sequence

TACCTATCTGCATGGTGGGGACCGGGAATGGAGGCAATTATGATAATAAAATAATAAAGACAATAAGTAGGGTTAGGTTGCCGTGACATAAGAACAAACCAACCAACTGAACACTGCTGAAACGTATCAGTGTCAATGCATCCATGACATTAGTGACCGATCACTAAACTGCCTATCCCCTCACCTACCAATTATGATTCCAAAGGCCCATCTATCAATAGAGAAAAATGAACGATGAGTGCCAAGGCAGAGGAACAACGGGAAAGAACAGATCTACATCAGCCGTCCTCAACTCATCAGCTAATGGCAAGATAAATTACGGTACATTAGAGTGTACTGTACAGAGTTCATGTAACTTCATGGCATCAATTGTTACAAGCGATACAGCCAGACACACAGGACATAACGTAGGACAAAACTGTACTAGGGAGAGAACATGGGCGCCATACTGCCGTATAAGTATAACCAGATAAGCTGACAGCTACGCTCCGGGCAACAGAGACATGCATCTGACATGACGCACGTATTTCAGCAATCCATGGGCATAATCACCCCCTTGGTCGCTAACAAAAGGAGTTGATGATGTTGGCGGTTGCAAGTTGCAAGATCCTTTCCACTCAAATAAACGAGTTGATCCGATCATTCGCAGATTCTCCCGTGCTTATCTGTAAAACGCGAATTGTTTATGCGATTTCGAGATTTATAAAGTTAACTAAAGCACATACATGTTGATTTCCCAAAGTATAGAAAAGGAGCTCCCCACTGCAACAAACACGCACCTAGATGCGACGCTGATCGATCGGATCCACATGTCCTAATTTTAGAGGCATCAAGCCAGCAAAGGTAATGTATTTGTCGGGAATACCACATACTACACCGAGGCAAAAAGGCAGGTAGCTAGGCAGGTAGGTATATAATCTCAGTATAAACCCAGATTTAGCCCCCCTCCTCGTCTCGTCGCCCGCCCATGCACGGCAATCATGGGATGCCATCCAAAAGCCCGAAACGTGCAGGCTCACGTGCGTGCGCCTCCCAACCGACAAAACAAACCCCTCCTCTCTCGTCCTCTCGTCCCGTCTCTACGCGAGCATTCGCTCATCAGCTCGAGAGCGAGCAGGCGGCGCGCATGTGCGGCCGCGCCGCGCAGGGGGAGCCGCCCGGGCCACGCACGGCTTGGAGGCACGCGTGAGTTCGTGACGTTTCTCGCGCACGGCGCGAGAGCGAGGTTTTTGCTCAGATCCGGCCAGATTCGCGGTGCGTGTCCTACTAGCTTTGTCACTCATGACCTACCACCGGCTACCGCTCCCATCCCACGGCTACCATCCATCACCATGATCCAACCCATGCTGCTGCCGATCGATCCGCACAAGAATAGCAGTGCTGTGTGCGGGAGGCTGAGGCGAGCCGTGTGTTCAAAGCTGCCTCTGGTTTGCTCTGCTCTCTACCGGCTTAACCACTTTGCCACTTTGGCCTTTCATCACGCGGGGTCAGGATGCAGTTGGTCATGAAGGGAAAAAGCTACTGACGAGATCAACGCCATGATTGTACGGCGGAGTGAAGCCTCCCTCATCACGGTGAACCTAATTAAAGATGATGATTCACCGGCAATCATTGCAACATAACCACACTGAAGGGGAAGAAAAACCTAAACCAGATGATGATTCAACGGTTGCATCAGGAGGAAAGGTTCGTGCGATGAAAGAATGTGTCGCATCGCATTCGAGTTAAAAAAAATTAGTGGGCGTTGAAACATTATTTTTCCCAACACGAGAGAAAGAGATAGAGAGAGAGAGAGGTAGACATGGCGCATTTATAATTGGTTAAAGATATGTTCCAGGTACATCCACTTATTATAACCCGGTGATACAAAAAGAAACAGGAAGGGGTCCGGTTGGTTGGAGCTCGAGCTGACGGCGGACGTGCTCGCCTCTTCTCCGGCTCTCTCTCTCTTCTCCTCCTCCTCATCTCGGCAAAACCACCACCGCATTGCCGGTATATTT

>Os03t0264400-02 2000 bp upstream sequence

CATTGCCCACATTTAAATAGATATTAATGAATCAGACATATATGTATGTATAGATTCATTAATATTTATATGAATATGAACAATGCTAAAAAGTTTTATAATATGAAACGGAGGAAGTAGTAACTAGTGAGAGCATGGTTGTTTCTTATCCCCTCTTTTATTTTTTGGGTTAATTGGATCCATGTCATAACAAATTTGCAACTCTTAAAAAATATCATTACCATTCACCTATTTGGTCCCATGACATTACAACTTTACATCTATTTGAGATATACCACTACTTACCACTACTTACTACTTACCCTCTTCCTCTCCCCTGTCTACTCATTCCGGCAGTGGTGCATGGCGAGGTCTGGGTCGGCGGTTCCCCTCTCTTCCTCTCCATCCCCTCTCACTTCGGGCAGAGTAAAATCGGCGACCTCGATACCGCCATTGACATCCTACGTTTGTGTTCCTCGTCACTCCACTATCGAATCCACCTCCATCATTGACAAGCACGCTGGCCATGCCTAGGTGGCTCGCCAACCTACACTTGCTTGACGACGACAGAGTCTCTAAGTGGCGCCTGAATTCAAGATGAGGTTCCGTTCGGCTTGAGGGGAACAATAAGGTGGCCAAACGAGGTTGACCTCGAATTATTCCCTTTCCCTGATACACACGCTAGAGGAGGAGATGGGTTGGTAGAGGGGAGGGAACGCGCGACGGTGGCGAGGGGATAGGGAGAGATGGCCGCCGGAGAGGTGGAGGTGGATCGGTGACCATCGTGGTTAGGCTCATTTCATTGAAAATGAGTGAGTGGAGAGGGGAAGGGAGAAGGGTAAGCTGATCAAAATTTTTAATGAATCCACCAGATAGATATATAAAATAGTAATATATCAAAAATAATTGTAAAATTCAAATAGGAATAGTAAATAACAATGATATATTTTAAAATAGGTAAGTTTATAAGGGGAAATATGTAGTGGAAACTTAAAAAATACAATACAGTTGGATCGAAAAATGGACGTCCGACATTTATCCACGTCATCACGATTTACTCATGAGTACTTATGATGATGTGAACAAATCTCGTACATTTTATTTTTTATTGTAATTTTAAGTTTCCACGTATTTTTCCCAGTTTATAATGGCACCGTCTCCCTCTCCCCATCGAGGAAGAGTAAACCCAAAAACCCATCTCGCTAGCCGCCGCCGGCCACAACCATTCCTCACGACGGGAGGAAGACGAAAAAAAAAAAAATCCCACTCCGTGTTGGTAAAATCCGACTACCACTTCGGCCTGTCTCAATCCAAGAATTCAAGAATCGAACTCCTTTGGGGTCGCCTCGCTGAATCTTCCTTATATATACGCGCTCTCCCATTCGCGTGTGCTCCAAATCGTAGTGGGGGGAAGTGGAGTTGACGAGCTGTGGAAAAAAAGCCTATCGCAATGGAGTCCATCGCCGCCGCCACGTTCACGCCCTCGCGCCTCGCCGCCCGCCCCGCCACTCCGGCGGCGGCGGCGGCCCCGGTTAGAGCGAGGGCGGCGGTAGCGGCAGGAGGGAGGAGGAGGACGAGTAGGCGCGGCGGCGTGAGGTGCTCCGCGGGGAAGCCAGAGGCAAGCGCGGTGATCAACGGGAGCGCGGCGGCGCGGGCGGCGGAGGAGGACAGGAGGCGCTTCTTCGAGGCGGCGGAGCGTGGGAGCGGGAAGGGCAACCTGGTGCCCATGTGGGAGTGCATCGTCTCCGACCACCTCACCCCCGTGCTCGCCTACCGCTGCCTCGTCCCCGAGGACAACATGGAGACGCCCAGCTTCCTCTTCGAGTCCGTCGAGCAGGGGCCCGAGGGCACCACCAACGTCGTACGGCCTTCTCCCCTCCCCTCCCCTCCATGTTCTTCCACCTTCCAATCTGCTCCCTTATCGATTCTTATCCTTTGGCAAATAACACTGCGTGCCTTGATTGGTTCGAACTTCGAAGGGTCGCTATAGCATGGTGGGAGCCCACCCAGTGATGGAGGTC

>Os03t0275500-02 2000 bp upstream sequence

TATGACAGTTTCTTTCATCGTGCATAACAGAGTTGAATAGTCTCCGTATAGTATTAACCATCAACTGCTTTAGCTATCTCCGACGACCATCGTGTTAGCCTATGCGGTCTGCCCAGAAGTAACCACAGGCCCCCCCAACGCAGCGCAGGGGTGTGTGTCACACTGTCACCTTTGGCGTGACGGGTCAGCCAGACCGACCAACCCGCCAGCTTCCGCCGCACCCGCACACGCCGATGTCAACGGCGCGCCGACCCCTCCCGTCCCGGCCTCACATCCGCGCAGCCCCCAAACCCCCCCCCCCCCCCCCCCCCAAACACCCGGGAGCTGCACGGTGCAACGCCCCCGCCCACCCGCCCGCCGCTTTGCGTGTGCGCACAACACAGCCGGCGGCGCAGCGGTCAACCGCGGCTTGGCCTTGGCTTCCCCCCGCGCGACGGCATGCCACCCCACTTTCCTTTCGCAGTGGAAAATATTTTTGGGAAAACCCGGACGGCCGAGGGGGCGCGTCGGTGCGCGCGCGCGGAAGGAAGCCACGCGAAGCTCGCTTTCCTTCCCTTCCCCTCGCGCAAAATATAAAAATCTGAAAACAGCAAGGCACCAAACGCCCCCCAACACAAAAACGCGCCACCACCACACACGCCGCCTGCCTCGCTTCTTCCTCCCTCGTCTCCCTCGCCTCCTCCGCGGACCGCAGTTTCAGACGAGCCCCAGAGCTCCCGGTCTCCCACCCCAAGAGGCCGGAGACAGAGCTGAGAGAGAGAGAGAGAGAGAGAGAGAGGGGAGGGGAGGGCGTGTGCTCGCGCGCCACCGCCCGGATCTCGAGCTCCCGGATCTACCGCAAAGGTAAATCTTTCGCTTGGATCGATCTCGCTCGCTAGATCCGTGCCTTTTTCACGATGAAGCCGGAGGGCGCCGGCTGCCGCTTTGGCTGGGAGAGAGTAGCTTCGGCTTAGCTACGGTTTGCTGCTGCTATAGATGCTGATGATAAGCTAAGAGTCGACTAGTAGTATTAATTAATATCTCTATCTAGCTATTCATTTCATCGGTGTTGGGTGGATTCCGCGGGTGAGATCTCGCCTCCCGACTCCGCGGATCTCATGCTCGGTAGGTACTCCGATGGGTTTTGAGGATAGAAGGTTGCGGAATTTCAGGGATCCGCGTTTGGTTTTGAGCAAATCCCGCGCTGGTTTTGCCCTGTTTGCGCGAGAACAGACAACAAACAGGGCGAAGTTATCTCCGATTCTCATGTGGGTGTTTGTTCTGTTCAGATCTAAACCGAGCAAGAGGCGGCGGCTGCTGCTGCTGCGGCCGCGGTTGTTGGTGGTTTGTTGGTGAGGAGGAGGAGGAGGAGGAGGAGGAGGAGGAGGCGCGGGCGACGGCGAGATGTCGTCGAGCACGATCCGGAAGGCGCTGGGGGCGGTGAAGGACCAGACCAGCATCGGTCTGGCCAAGGTCACCAGCAACATCGCGCCGGAGCTCGACGTGCTCATCGTCAAGGCTACCAGCCACGACGACGAGCCGGCCGAGGAGCGACACATCCGCGAGATCCTCCACCTCACCTCCGGCTCCCGCGCCCACGTCGCCGCCGCCGTCGCGGGATGCTCCCGGAGGCTGTCCCGCACCCGCGACTACGTCGTCGCGCTCAAGTCGCTCATGCTCGTGCACCGCCTTCTCGCCGACGGCGACCCTTCCTTCCACCGCGAGCTCCTGCACGCCACGCGCAGGGGCACTCGCCTCCTCAACCTCTCCGACTTCCGCGACGAGGCTCACTCCGGTTCATGGGACCACTCTGCCTTCGTCCGTACCTATGCGCTGTACCTCGACCAGCGCCTTGAGTTCTTCCTCCATGAGCGCAAGCAAGGCTCCGGCTCCAACGCCAGTTCCAGTGCAAATGGCCCGTCACCGCGTGACCGCTGGGGATCACCTGACCCATATGGTCGCCGGTCACCCTCATACTCCTCACCCCCTGGAAACGGAAATGGGTATGGATATGGTGGTTA

>Os03t0278300-04 2000 bp upstream sequence

TCGGATCTCCTTTTATACATGGGAGAAATTATAGTTTTGGTGTCTGCTTGAGAAAAGTATCTTCCACTGTCTTCTCCAGTTCTCCCACAAGGTGTTTCATGCAAAAGACTGAACTTGTGCTATGTACGGCCCCAACCTCCAAGGTTCTCTCTGAGATTAATTATGACCATCTAGTGGCAACTGGGGTTAAGAAAGAGCACATGTTTATTCACGGCCTTCGAAACTTCGCCGGATGTTCATTTTTCATCACGTGCTACCGGACTGAGTATTTCCCACCTGGACTTTCAAGTTTGGGCAAGTTTCACCTATTTTTATGCCATGTTGGTGGCTAAACTTGTACTGCCCACACATATCATACACTAGTATTCTTCTTCCTTCTCGCATCTATCTGTAGAAGAACTTCCGTATCTTCCTGTTTGCTTATCAGCTGTTGCTAAATTTTGAACATTGAAACTTTAGAGGTATTTTCTAGCTCCAGCTTTTAAACACTAAGGACACATACATATAAGAGTTTTATTTTGTTACTTTGTCCATTTTTCTACTCAACAGATCAATGCGTGAGACTTAACTCACTATCTTGCATGCATAATCCAAATGTTTTTTTATTAACACAATTGTCCATATTAGTTCACATTTTCTAAAAATATGGCATCACATTTTTTATTTGATTTCAATTGCTTCCCCTTATGCGCTTGCTACCTCTATCTCTGTACATAAAAGAATGTTTCCATCTCTCGTACCTTCTCTTTCTAGTAGACAATCATTTTTTACTAGCTCTTTTCTATATTGTGCAGTAGGAAATTTTATTATATATGTATATTACTTCCTCCGTTTCACAATGTAAGTCATTATAGCATTTCACACATTTATATTGATGTTAATGAATCTAGATAGATATATATGTCTAGATTCATTAACATCAATATGAATGTGAATATGAATGTGGGAAATTATAGAATGACTTACATTGTGAAACGGATGGAGTACTTTAAATGATATCATTTAAAATATTTTTTTTATCGACATGTGGTTGTCCCAAATACTCCTCCATTACGATATATAAGGATTCCTGGGTTATCTAGGAAATACTAAGTTTACGTCAAAAAGATTTCTTTTAAGTCACTTTGTTACCTTTTGAATTTTGAATTGCTTATATTATGATATAAAGTCTGAATATTAAAAACCTTCATATTTTGAGAAACAGAGAGTGAGTACTAGTCAACCGACTTTGCCGACTTGCCACGAAAAAAAAAGAACATGAGGAAGATAAGCACTCGAGGTCGAACCCAAGATACTCCAGCGAGAAATACTCGGAACAACGGAACGAGGCGACCCCGAGCCAGACCCCGACCCGGCACTGCAGGCATCCCAATATCCAATCCAATCAGTGCCTCTCCTCTCCTTCCCGTCCGCATCACGCCCTCGTGGAGTCGTGGGTCTCTCTCGCGACCGCCTCACCACGCGTTATCCCTACGCGTCCGTCTCCCCCCTTTATTTTTATCGCGCCCGCCACCGAAAAGACGAAAAAAATCTCCCAATTCCCCAACCCTAACGCCGCTCTCGCCTCCACCCCCCAAATCCCGAATCCGCCGCCTCTTCGTTCGTCGTCGAGCTCGCTCGCTCGCCCAACCCTAGCGCGCCAGATCTACCCGCCCCCGAGCAGCCGCCATGGCGGAGACCCTCGACATGACGCTCGACGACATCATCAAGAACAACAAGAAGGCCAACCCCTCGTCCGGCCGCGGCCGCCGCGGATCTGCCGCTGGCGGCGGCGGCGGCGGGGGTGGGGGTGTTGGTGGTGGCGGCGGCGGCGGCGTGGGGCCGACGAGGCGGCCCTTCAAGAGGTCGGGGAACAGGGCGGGGCCCTACCAGCCGCCCAAGGTACGAACAGCAACGGATCGAACGTGGGGCTAGGGTTTGCTGCGCGCCGTCGGCGGAGGAGCTGGGCTTCCGCTTTTCGACGGCGAGGCGGGGAAGGATGTGGATGGATGGTCGGGTGG

>Os03t0291800-02 2000 bp upstream sequence

TCAGTTTCTTCCGATTTCTCGGATCCATGTCTCCACTCTCCACACATTGTTAATGTTCTCAACTTAACAGAAGAATCATTTTTACAGCAAGTGATCAATGAAAAATAATAGTATCTGACAATGATATTCTTTTGAAAAAAGAAAATATTCGACCCTACATAGGCACATCATTTGAGATCTGGAAGTTGAAAGAAAATGATAACAGACAGTGATATCAATTCGTATGCGTACTAGCTAAGACGAGTTGGCGTGACAAGATTGGAAACATATTGAACACCCGCAGAAAAGCACTGTGTAAAAACTGAAACGTGTTATACTCTACAGAAACAGGACAACCGACTCTGAAAGATGCACCGATTCTCTGATTTTTTTGCAAGGCTATTCTGTGACCTGAAATTTCAGTCACCTATGCTGTACACGGTGCTTTCATTTTCCCCCTGTAATATCCATTTTCTGCTCGAAACATTGGTCCTATTAAATCAACCTCTGACCATGACTAACATCACTTCCTGAACAATGATTTTCGTAGACCAATCCCCCCGAACACTGTCGATCGGAACAATTCGACATTCTGCAGTTTCCTCCTGCTGCAACACTATCTGAAAATCGGAACGATTCAGGCACCGTATCGTTGTCTGATTCGTCGATTCGTTCCATCATTAGTGTGTTCACAACGCAAAGACGATGACTGTAGTAGTATCTGCGTGCTGATTACATCCAAATACAGAATGATCATCTCCGAGAATGCGAAAGGGGGGCAATTGACGTAGCCGCCCCCGCCGTTGTTTGCTTCCCCATCTCGCACTCTTCCGAGACAGCCACGGCCTACCCACTCACATCGCATAAACAAAAAACGAGTACTTACAACCATACGAGTATACACCATAATACCATATACTCCACCCCTGTATCCCGATAATGAAAACGAAATGTATGATATAGTAGGCATATAATTAAAACATGCAACGAACAAATAGAATACTAGTAGTAGAATACACACACGCACATTGCCTGTGTTGTTACATGTGTACAACAGCTTAACCTCCAAACCCCCCATGGAAGCTTTGCAGCCTTGCATGTTACTCATTTGACCCACCCCTCAGTAGTAGTACTACCCTCTATCTCTCTATATACAAAATGAAAGGGAATGAGATTCAATTAAGCAGCTAGAACAGGGGGGAGTTTACAAGTGTTGGTGGAGGGGAAGAGATCTTGCAACCATAAAAGAAGAAAGCAGGCGAACCAAACCAAACATACCAAAGGGAAGCAAAGAAAAGGGCAAACATCCAGCAAAAGTCACCAAACTTGTAAAGGGATCGACACATATATAGGAGAGCGAGGTGTCCAGTCGAGAAGGACAGCCAGGCCAAGGAGCTCCTAGCCTCCTAGCCTACACAGAGCTAAGCAAAGAGGCAAAGAGCTCAGCAAGAAGCTCCATTAATCCATTCCATATACTCATATAGAGAGGAGGAGGAGGAGGAAGAGGAAGAGAGGGGAAGCAAAGCAAGAAGCTTTTTTTACTTCTCGGGTTTGCAAGCCACCGGGAAAGGCCGGGGCTTGGTGGTGGTGGTGGTGGTAAGTTTTCTTTAAAGCGGCGGAGCGGGAGGCCTTCCTGGGCGCTCCGGAGATGCAGCAGCGGCGGAAGTCGGTGTTCGCCTCGGCGCCGTTCGCGATGAAGCAGGCGGCGCTGGGGGCCGGCGTGGCGGCGCGCAGGAACGGCGCGCCGCTGTCGCTGGCGGCGGTGGTGTTCGCGCTCTTCGTGTTCGCGACGTTCCTCTACAACGAGGACATCAAGTCCATCGCCGACTTCCCCTTCGGCGCCGGCGCGCTCCGCGCCAAGTCCCCCGACCTCCACGTCCTCCAGGAGACCGTGGGCGCCGCGCACCTCGCCGCCGGCAGCATCGCCAAGCGCGGCGAGGAGGTCATCGTCCGTGTCCTCGACGCGCCCGCCTCCACGGCGATGGCGGCCGCCGCCGGCAGCAGCAGCAACAACAGCACGA

>Os03t0302000-01 2000 bp upstream sequence

GACGACTCAAAGTGATCGCCGGGTTTCCCAGCGAAACGCCCATTCCTCTACCACGCTATAGCCAGCGTCGCACACGGAAACAACCGCACATAACACATTCGCGCGCGCGCCAGCGCCATGGTCACCGCCGCAGGCTCCCCCTCCTCCTCCTCAGCCCGCAAGCCAGCCTCCCGCCCGCGGCTGCCATGCCGCGACATCCTCGTCCACATCGAGGCCTACCTCTCCCGCCGCGACGGCGTCGACAACCTCCTCAAGGTCTCCCTCTACGCCGCCCGCCTCGCCCTCGCCCTCGCCGCCGGGCAGCCGCCGCTGCCCCACGCCGCCACCGCCCGCCTCAGGTCCTTCGAGTCCAGCGTCGGCCTCAGCCGCAAGGCCTTCCGCCTCGGCAAGTTCGTCCAGTCCATCAACGCCCTCCGCGCCGCCGCCTACCACCCCCACCCCCACGTGCACCCGCTCCTCGTCCTCCTCGCGTACGGCGGCCAGGGCGTCTACAACTTCCTCGAGCAGTTCGCGTGGCTGGCCAAGGCCGGCCTCCTGCCCGCGCGCCTCCTCCCCCGCCGCCTCCATCGCATCGGCGTCTGGGCGCAGCTGCTGGCGCACGTCGGCTCCATCGCGATCAAGCTGGAGGAGGTGGCAGAGCTGGAGTGTGGCGTCGAGGCGCGGCTCGAGGAAGGTTGCGGAGAGGAGAGCGAGGTGGTGAGAACGCTGAGCCGGAAGCTGCTGCTCAAGCTGATGTCCCTTGTGCAGGACATGGTGGATTCCGCGATGACGGTAGGGGACGTGACCGGCCGGAAGGGTTTGCTCGGCAGCTCGACGCTAATGGCGTCCGCCGGCTTGCTGTCCGCGCTGATCAGCGTTCACAAGAACTGGAATTCGTGCTGAACTGAAAGCCCACCAAAGGTACATGTTATAAAACATGATGTAATTGAACAATAATTAAATCTTAAAGTGGTGAGAGTTTGAATTAGTAAATCAAATTACTCCTGGACTCTGGATGTCGGGGTATAAATTAATCAAAAGGTGGTGCTGGTGCATTTGAGCGGATACAAAAACTGGGCTTTCTCCGCCATGGTGATCATTCAATTTTTTGCTCATGCTTCTCCTTATTAACAAGTGTATATCAACAAAGCCGATTAATATGTTTCCTCATAATTTTTTATTTCTTTCTGGAACTATAGCCTGTATAAACTTTTTTAAGATATATAAACTGTTTACACTGTAAGGCTGCTGTAATACTAACTTCAAAAGCATCTTTCGTTAGATAAGCAACCAAAAGTAAATAATCGTGGAGCTGCTTACCAAGTATATAGGAGTATGTCTCAAAAATTAAAGCAGAGGGAAAATGAAAATGCAACGGAAAGTGTTCTTAGATGTACTAGTATCCCCTGATTTTTTAGATTACTTCATTAATTGGCACGGCTTGTGATCAAACAATGTATGCTATCCCAAGCTAATGCGAAATTAAACACGATCATGCTACTAATCCAAGGAGATATTAACGCAGTAAGAATCAATGGGCGATGCCATCATGCATTCTTCAAAGAAAAGAGTGGCCTCCACATCCTCTGAAGTCTGAATTCCGAAGCCAGGCTTTCCGGTCAGTGCCAGTATAAATAAACATGATTTATCGATATGATAGGCTACTTAATTTCATGTGATATTTACTATGCATCCAAAATCACAAATGGTAGGTGCGTTGTGAGCGTCTATATTTGTACTGTGTTTCTAGAAAAAATCACAGATGATTTTGTTGTGACAGAATTCTATGTATACATAACAAGAAAATCCCAACAAAGTGGGTCAGGAAACACTGGGCTTAAAAGCAAAGGTCCAGAAAGATGATAAAGCGGCCCAAGAAGGTCCAATGTAAGAGTTTAAGGCCCATTTGCTAGGCTGACGCATGGGACCCATATTGCGGTTCGGCCCAATGACACGTCACCCTTCGTCAAGTCCAAGTTCCAAACCCGTACGAAACGCCCACTCCCCTACCACGACCAGCC

>Os03t0327100-01 2000 bp upstream sequence

ATTGCACATTTGCGCTGATAATTTTGACGCTTGAAATGTGATCCTCTGTTGATGCTTTAATTACAGTTATATATGTATTGGGGTTCCCAACTAAATCTGGATATTTTGTGTTTGAATGTATCCTCTAGATGAAATGCCCTGGAAATCAATTTCGGGTGCACTGTAGCTATCGGAAAAAGACGAAAATTGTTATGTATTTTGTAGTTGACAATGTTGCTATGGACGATGGTTAGGGGGCTGTGGTAGTGAGATTCTCTTGCATGAACTAGTCAATGCAAATATGCGAGAGCAGTGGTTGGTACAATGAGTTGAAAATCAAGTATAAATCAACTTGAAGCCTTCAAATGATTTGTTAATTTACCCATATATGATTACAAGCCGGTACTGCTTAACGCAGCGGCTATTGAACATTGAACATGCCTAGTGGTTACAAAACATTGACCCGCAAGAGAAGTACTACTTGTTGCTATTATCGCCCATTTACTTGTTTTCTCTCTGGAACTCAAAACATGCTGTCATAGCCATCCTTCTTATCTGACTTGCCAATAAACAATGATATCTACTTTTTTTAGGTGGTCTAAAGGCATTTTCAATGAACAGTTTGGAACTTTGTTAGCTTGACTCGATCCGTCCTTTATTTTTCCTCCCTTTTCTGAACTCTGCAAAGATGAGCTTTTTAAATTGTCCTTTCGACGCCAATAAGTCGTTCACGTTAGGCCAACTCCTCCTTCGATCGGCAAAGATGATATCCATTGGCGCTCGCTTAACGTGTGCAAGGACACCACCAGCAAACACAGCAATAAAAACAATTAGATTTTGCTGCGACGTCATGCTGCTGGGGCCGCAGGGACGATGCTAGGCCGGAACAGAAGCACGTCGCCGCTTGACATTTCGCCTTCCTGACGCCGGAAACTTGGGGGGGGGGGGGGGGGGGGCTGATGGCACGGTGGACACCGGCGCGTGTTCACTTCAGAAAGGGGGAAAAAACGCTGCTCGGGAGAGTCAAATTTTCCAGCACTTTTTTTGGGGCTCGGCCGCGCCCATACACGGGTGAAAGCCAGCCCGTGTCAGTCTTTGCAGAAAATGCAGAAAATTGTCGTGGTCGCGAGCTCGACGTCTCACCCGGTCCCTCCCTCCGGTCGCATGGACAAACGTCACAGGCGCCCACAGCAGCAACTGGAACAACTTCCCCAAAGAAGCGAGAGAGGAGGGAGAGATTTGGCATGGACCAATCCATCGGTATCTGCAAGGTTTCGGAGCAGCTGCGACGTGTTTTTCCGATATCTGATCGCTCGAAAGGAACTGGGACTCTGGAAGAGGTAGCTAGCGGTTGCCTGATGCACCATGCGCTGGCTGGGCTGGGGTGCAGAGCGCAGCGGCTGGCCGGCTGCTGCTGGGTTGAATTTTTCTCGTGGGGGGCATGTGGGGGGAGAGACCCAGCGAGCTAGCTCACTCGTTTTTCAAGTCAGCTAGAGAAATGTAAACATATCAAAACACATTAATGCGAACCGGTTGAGCTGTTTTATATATATATATATATATATATATATATATATATATATATATATATATATATATATATATATATATATATATATATATTAAAAAAAGAGGAAATTAAAAATCAACAAGCACGCAGGTTTTTTCTTCGCTGCAGCACCGCCGGTGCCGCGTTAGCTGTGGAGGTCCGGATGACTGAATATTCCACAGCCAAAAAGCAGCGGTGGTCGGTGCCTTGGTGGGGAAGAGCAGTGAAAAATTGAAGGGGAGAGCTAGCTACTGCCTACCAGTAGTAGTAGTAGTAGTAGTAGTAGTTAGGGCCAGGCAATCAGGATTAGCACGGTTAATTAGGTAATTAGGACAGTTTCGGCTCGGTGAAAGACGTGAAGCCCCACCCCACCGTGGGGGCGAGGCGACGGAGGCTTCGGCCGCAAGCCGCCTCGGCACGGCTCTCCTCCGCGTCGAGCCTTATAAAAGCCGCCCCTCGGTTCCTCCCCCCC

>Os03t0334200-01 2000 bp upstream sequence

ACGCACGGGTTTCTGTGGCTTTGGTGGGTTGTTGAAAAGCGAATTCATTTGTAAGACATCGATGGAATAGAATGCCCTTTTGACTGTACTGACTACAAGTAATCATGAAGTGCTCTGCTAGCATTGCTCTAGTCACTAGTCAGAATGTTCGGTTTTTACTGGTATTCAAGAAAGGGGCTTGGTTTTATCCAAAAGCGAAGAATTGGCCACTACAACAAATCTCAACAACTATAGGACAACTTACTCTATTAGAAGCAGTAATATATATATAGTCTTTTTTCCTGTGTCCTGTGCCTTCAGCTAGTGCGAGATCCTCCTACCTGTCGGCGACTTGACTCAACACGGCCACATGCTGCTTGTACGCGCGTGTAACCGACGACCTGAACCGGACCCTACTACAGTACTAGTAGTGCGTTTTTAGTTCAGCGACCAACCCAGCAGCACACATAAGCTTACGGACGTCGTCGTATCAACGACTTCTTACGAACAACTTCAAGCTGGACTGCTGGAGTAGCGATGAAGTTTGAGTGGTTGATGTGAAGCAGTAAATAGCGGATCGTTGTTCGAGTCGGTGAGCCGATGGAATGGATGACAGCGTCGGCTCATTTATTGGCAGCATACCTCCGCTGCTGCCCAGATTTATGAACCCTAACTTTTCCAGCAAAGACACACTGTTCGTGGATCGGGGCAAGCTGCCGTAGATCCTGGATTAAGGCCCCTTTGAATGAAAGGAAAAACCATAAAAATAGGATTTTATCCTATAAAAAATAATCCTATAGTGTCCTTTGAAATAAAGGATTATTTCCTATCAATTCCTTTGAAATTACTATGGAATGAACCTTACTAGAGTAACTTTGAAGGAAAATAAGCATGAGGTCTCACCTCATGTTTTCCTCTTCATGTGTCTAAGATTCCTGCGTTTTTTCTGTGTGCTAATCAAACGACAGTTTGGAGACTTTCCTGTGTTTCAAAATCCTATAGAAATCCAATAAATGTGGCATTCAATTCCTACGTTTTTTCTATTCCTCTGTTTTCTCAATCCTGCGATTTAAAGGGGCCCTAATTCCTTATGGAATACTACTTGTCGTCGTTGATGCTACCTACTCCTACCTCCTAGCCGCCATGGCTAAGGAGTATTATTATCTCTTTTCCTTCTCGTTTCAACGAGTCCTCAAGAGTGTCAATGCGAGACTGAGCTGAGCAAGCCTGAAGATGGTCGGTCTGCACTCTGCAGGCAATGGATGAATGGATACACGAATTTACCAGGGAAGAAGCCGAGTAATCTTGCTGTGGTGTGGACTGTGGAGAAAACCATCAGTCGACGAGTCGATGAGATATGTGCTCTCTCCATCGTAAAATATACTCGCAACTTATTAGGACAGAGTAGTTTTCGAAATAATGAAACCTGCAACAGAAACCGTCTACAGAACTAGTACTCCATTGCTTCAGACTTCAGCCATCAGCCATCAGCCCGTCACCGTTGAAATATTGAATGAGCTGTCAAGTACAGAACTACATACACGGGGCCACTTGCTGTGGCTCGCATCGCATCGCATACGTTCTGACTTTCTCAAGACCAAAGGAACAGGACAAGTGATTATAGAATATTGGACATTCAGAATTTCAGACTTCAGACTTTGATGAGAGTTGAGACTCTCGGGGGCTCGTTTTCTAGAAGGGTAAACGTTTTACCGCGAGACACGTTCTCATCTCATCTCACTCACCTTACTGTCCCTGTCAGCTGGGCCCCATCGCCCCGAGCGAAGAAGCACGCGTGGATAGGGCAGGCGGGCCCAACACGCGGGCGCCACGACCACCACCCATCTGGCCATCCAAACCACCGCGATGACGTGGCGTGAAGTCAACACGCCCATCCACGGTCAACCTTCAAATCCTCCCGCTCCTCCTCTCCGTTTTATTTTATTTTACTACCAACCCGCTCACAGTTTGACCACGGGTTGTTCTCCACTTCTCCTCCTCCCTCTCTCTCTCTCGCAGCA

>Os03t0334200-02 2000 bp upstream sequence

CGGCCACATGCTGCTTGTACGCGCGTGTAACCGACGACCTGAACCGGACCCTACTACAGTACTAGTAGTGCGTTTTTAGTTCAGCGACCAACCCAGCAGCACACATAAGCTTACGGACGTCGTCGTATCAACGACTTCTTACGAACAACTTCAAGCTGGACTGCTGGAGTAGCGATGAAGTTTGAGTGGTTGATGTGAAGCAGTAAATAGCGGATCGTTGTTCGAGTCGGTGAGCCGATGGAATGGATGACAGCGTCGGCTCATTTATTGGCAGCATACCTCCGCTGCTGCCCAGATTTATGAACCCTAACTTTTCCAGCAAAGACACACTGTTCGTGGATCGGGGCAAGCTGCCGTAGATCCTGGATTAAGGCCCCTTTGAATGAAAGGAAAAACCATAAAAATAGGATTTTATCCTATAAAAAATAATCCTATAGTGTCCTTTGAAATAAAGGATTATTTCCTATCAATTCCTTTGAAATTACTATGGAATGAACCTTACTAGAGTAACTTTGAAGGAAAATAAGCATGAGGTCTCACCTCATGTTTTCCTCTTCATGTGTCTAAGATTCCTGCGTTTTTTCTGTGTGCTAATCAAACGACAGTTTGGAGACTTTCCTGTGTTTCAAAATCCTATAGAAATCCAATAAATGTGGCATTCAATTCCTACGTTTTTTCTATTCCTCTGTTTTCTCAATCCTGCGATTTAAAGGGGCCCTAATTCCTTATGGAATACTACTTGTCGTCGTTGATGCTACCTACTCCTACCTCCTAGCCGCCATGGCTAAGGAGTATTATTATCTCTTTTCCTTCTCGTTTCAACGAGTCCTCAAGAGTGTCAATGCGAGACTGAGCTGAGCAAGCCTGAAGATGGTCGGTCTGCACTCTGCAGGCAATGGATGAATGGATACACGAATTTACCAGGGAAGAAGCCGAGTAATCTTGCTGTGGTGTGGACTGTGGAGAAAACCATCAGTCGACGAGTCGATGAGATATGTGCTCTCTCCATCGTAAAATATACTCGCAACTTATTAGGACAGAGTAGTTTTCGAAATAATGAAACCTGCAACAGAAACCGTCTACAGAACTAGTACTCCATTGCTTCAGACTTCAGCCATCAGCCATCAGCCCGTCACCGTTGAAATATTGAATGAGCTGTCAAGTACAGAACTACATACACGGGGCCACTTGCTGTGGCTCGCATCGCATCGCATACGTTCTGACTTTCTCAAGACCAAAGGAACAGGACAAGTGATTATAGAATATTGGACATTCAGAATTTCAGACTTCAGACTTTGATGAGAGTTGAGACTCTCGGGGGCTCGTTTTCTAGAAGGGTAAACGTTTTACCGCGAGACACGTTCTCATCTCATCTCACTCACCTTACTGTCCCTGTCAGCTGGGCCCCATCGCCCCGAGCGAAGAAGCACGCGTGGATAGGGCAGGCGGGCCCAACACGCGGGCGCCACGACCACCACCCATCTGGCCATCCAAACCACCGCGATGACGTGGCGTGAAGTCAACACGCCCATCCACGGTCAACCTTCAAATCCTCCCGCTCCTCCTCTCCGTTTTATTTTATTTTACTACCAACCCGCTCACAGTTTGACCACGGGTTGTTCTCCACTTCTCCTCCTCCCTCTCTCTCTCTCGCAGCAGTACACACAAGCGAAGCAAAAGCGCGGTGCGGAGAAGTCTCCCCGCCGGGGAGGCCCGTTCACACCGGTGATTGGGTTGGAGCAATTAAAGCTTTCCTCCTAGTCAACCCCAGCTCCTCTGCGAGGAAAGAATTGATCTCTTTTATAATCCTGGGCTCTCACTGTTCGTTTGCTTCTTCGTTTCGCCTGCCAAACTTTCCGAGAAGCGGAGCAGCAGTTGAGGAGAAACGACGGGGAGAAGCGGCCGAGGAGGTCTGGAGAAAAGGTGAGTTCTTGGCTTCTTCCTCCTCCTCCTCCTCCTTTTCGCCCTCATTTCTCCCTCTTTCCTGTCTCCGGATTTAG

>Os03t0364400-01 2000 bp upstream sequence

TGAGATCGCCATGATTATCACCGAACACTTATCAAAGCCAAAGTAAATAAATCTGTTCTAAGCAAATCAATAGAATAAGAAGAGGAGAACTTTTGAAAAATGGAAGGAGAGGTCTAGAAATATATTAGTATGCATTGAAATATGTAAATTTGGGGGCCGTGTGGTCGCTTACTTCGCACTTATCCACGGACGGGCCTAAAGAGAAGCTAAAAATGTTAAGAGAGGGGGTGAAACGGATGGTACGGTAAAGCGTTTCGGGCCCTCACGTCGCCCTCGCGCGGCGGCTTGGGGGCGAAACCCTAGCCGCCGCCGCCCCTCCTCCTCCCTCCTCCCTGCTTTGCTGTTGCCGTAGCTCGTCGCCGGCAAAGCTGGGCGACGCGCTGGGATGGCGGCGGCGGGGCCATCTCTCTCGGTTCCATCTCGCCCCGACGCGGGTGTGGCGACGATGGCGGCGGGACGGCGAGGTCGCGGCGACGGCACGGCGGTGGGGCTCTCCGCGGCTCCGGCTCCGGCGTCGCCAAATTCGGTTGCCCCGCTTCCAGATCTGGCCAAGGTGACCAGCGACGGACTCTGTCGACGCCGGTGTCGGGCTCTAGATCTGGTTCTCCCCTTCTTCTATGGTTGTTCTTCCGGTGCTCACTGGCGGTGTGGCGCCCGCTCCCCTCTTCTTAGGTGCTCCTCCCTTCTTAGGCTAGGCTGCTTCCAGTCGATGCCGGGGAGCTCCTTGGTTCGGTAGGGGTGGCGTGCGGAGTTGCTGGCGTCCTGTCTCGCTAGTCGTCGGCCGCGCCATAGGTTTGCTGCAGCTGGGTTCCTGTGTGTGAGGTGGTCTCTGGTCGAGCTGCGATGTGATTGACTGTTGACGTGGAGTCTGAGTTGCTTGTATTGCAAGCTCGGCAACGATGACCCTCAACAGATGTCCCGACAATGCGACGAGGATTGGGAGGTTGAGCATTTTGAGAGAGCCTCGGGTGAAAACCTTGCCTAGTGCTTTCCTAGGTTGATTATATCGACGCCTGTGGGCGCCGTTTCCTCCTTGGAGGTATCGTCATGCTGACTCTCTCTTCTTTCAGGTGAAAACCGGGTTCCTTTCAGAATGAGCGTTGACAGCATCCTTGATGTTGTTCCCTTGATGAAGGCTTCGCTCAGAAGGTTTCTGCTCCACTAGAGGATGTTTGGTGGTTCTGGAGACCTCGTTTATATTGTTGAAGCAGCTTGCCTTTGGTAAGCTCGGCAACGACGACTTCTGTGAGGTCTCAATAGTTGGTTTTCTTTTCCTTTTCTCTATAGGTTTTTGCTAATTTCCCGTTTAATTAACCGTGCTTGTTCGGTTTTCGGGCCCGGTTTCCCTTATTAACTGGGTCAATTATCTTCTTCTATAAATTCCGGCAGGAGAAACTCCTGCCGTCGGTTCCTCAAAAAAAAATGTTAAGAGATGGCACATGAATATTTAATTACAGTATTAGTTGATTTCTGAATTATACAACTGAGTTCTCAATCTTGAGAAAGAACATAGATTATTTAAGATAGCCGAAGGAATAATAATAATAATAATAATAATAATAATAATAATAATAATAATAATAATAACAACAATATTTTATTATTATTATTATTATTATTATTATTATTATTATTATTATTATTATTATTATTATTATTATTATTATTATTATTATTATTATTATTATTATTATTATTATTATTATTATTAGCACATCACGTGTAGCACGCCTCTGTTCCTCCCTTATCATCCAATACCTTATAAATAATGCATGCTCATATACATAGTCACATTCCATTATTCCATTGCTGATCAATCAGGTGTACACATCCTCAGTCAGATCTAGTGTAAGCTAAGCCAGTGTGGCGTGGAGCCGTGGAGACGACCATCTGACGCAAGAGACAACAAGTCCACCAAAGGCCCCCGCTCGATCGAAAAGGCGAGGCCGGTGCTTTGCCATACATACATACATAAGCCCCTCCAAAGAGAGGGAGTGGTT

>Os03t0364400-02 2000 bp upstream sequence

TATTAGTATGCATTGAAATATGTAAATTTGGGGGCCGTGTGGTCGCTTACTTCGCACTTATCCACGGACGGGCCTAAAGAGAAGCTAAAAATGTTAAGAGAGGGGGTGAAACGGATGGTACGGTAAAGCGTTTCGGGCCCTCACGTCGCCCTCGCGCGGCGGCTTGGGGGCGAAACCCTAGCCGCCGCCGCCCCTCCTCCTCCCTCCTCCCTGCTTTGCTGTTGCCGTAGCTCGTCGCCGGCAAAGCTGGGCGACGCGCTGGGATGGCGGCGGCGGGGCCATCTCTCTCGGTTCCATCTCGCCCCGACGCGGGTGTGGCGACGATGGCGGCGGGACGGCGAGGTCGCGGCGACGGCACGGCGGTGGGGCTCTCCGCGGCTCCGGCTCCGGCGTCGCCAAATTCGGTTGCCCCGCTTCCAGATCTGGCCAAGGTGACCAGCGACGGACTCTGTCGACGCCGGTGTCGGGCTCTAGATCTGGTTCTCCCCTTCTTCTATGGTTGTTCTTCCGGTGCTCACTGGCGGTGTGGCGCCCGCTCCCCTCTTCTTAGGTGCTCCTCCCTTCTTAGGCTAGGCTGCTTCCAGTCGATGCCGGGGAGCTCCTTGGTTCGGTAGGGGTGGCGTGCGGAGTTGCTGGCGTCCTGTCTCGCTAGTCGTCGGCCGCGCCATAGGTTTGCTGCAGCTGGGTTCCTGTGTGTGAGGTGGTCTCTGGTCGAGCTGCGATGTGATTGACTGTTGACGTGGAGTCTGAGTTGCTTGTATTGCAAGCTCGGCAACGATGACCCTCAACAGATGTCCCGACAATGCGACGAGGATTGGGAGGTTGAGCATTTTGAGAGAGCCTCGGGTGAAAACCTTGCCTAGTGCTTTCCTAGGTTGATTATATCGACGCCTGTGGGCGCCGTTTCCTCCTTGGAGGTATCGTCATGCTGACTCTCTCTTCTTTCAGGTGAAAACCGGGTTCCTTTCAGAATGAGCGTTGACAGCATCCTTGATGTTGTTCCCTTGATGAAGGCTTCGCTCAGAAGGTTTCTGCTCCACTAGAGGATGTTTGGTGGTTCTGGAGACCTCGTTTATATTGTTGAAGCAGCTTGCCTTTGGTAAGCTCGGCAACGACGACTTCTGTGAGGTCTCAATAGTTGGTTTTCTTTTCCTTTTCTCTATAGGTTTTTGCTAATTTCCCGTTTAATTAACCGTGCTTGTTCGGTTTTCGGGCCCGGTTTCCCTTATTAACTGGGTCAATTATCTTCTTCTATAAATTCCGGCAGGAGAAACTCCTGCCGTCGGTTCCTCAAAAAAAAATGTTAAGAGATGGCACATGAATATTTAATTACAGTATTAGTTGATTTCTGAATTATACAACTGAGTTCTCAATCTTGAGAAAGAACATAGATTATTTAAGATAGCCGAAGGAATAATAATAATAATAATAATAATAATAATAATAATAATAATAATAATAATAACAACAATATTTTATTATTATTATTATTATTATTATTATTATTATTATTATTATTATTATTATTATTATTATTATTATTATTATTATTATTATTATTATTATTATTATTATTATTATTATTATTAGCACATCACGTGTAGCACGCCTCTGTTCCTCCCTTATCATCCAATACCTTATAAATAATGCATGCTCATATACATAGTCACATTCCATTATTCCATTGCTGATCAATCAGGTGTACACATCCTCAGTCAGATCTAGTGTAAGCTAAGCCAGTGTGGCGTGGAGCCGTGGAGACGACCATCTGACGCAAGAGACAACAAGTCCACCAAAGGCCCCCGCTCGATCGAAAAGGCGAGGCCGGTGCTTTGCCATACATACATACATAAGCCCCTCCAAAGAGAGGGAGTGGTTGGCTCGGATCGGAGCAGTTGGCTGCAGCCGCAGTTTGTTGGAGCGGTTGGCCCTCGCACTCGCACTACCCCCCGCCCTTTTTCTCCCGCCATATTTCTACTTCTCCCGGCCCACCCCCCCTC

>Os03t0663400-02 2000 bp upstream sequence

TAATTGTAACTATACTGTACCTATGATATAACTTATATAAAACTTATATTCAATTATGGTTTGGTTGGTTAGCACTGGAATCTTTACGTGTGACATGTGTAAAAGTTTTTTCTAGCAATTTTTTTTCATGCACAAATCTTAAGGTGATCCAATAATCACAAATTTGAAAGTTTTTGGACTAAAAAAACTTACATAAGTTTTCTAGCAATTCCGTAAAAGTTTCCCCCGTTTTATAGAGGGCATAAACCCGTCGGCCCCATTATATGTGCCATTTGTACGTTTCTATTTTCTCAGTAATCAGACCATTAAGCCGAGACCGACCGCCAGAACATCCCTAGTTTCCGCGCCAAACTTGTGGAATTTCTGTGAGAAATTTGCGCATGGAAGCACCGGAATTCCACGTTATCCAGACTATAAATATCTGCATCGATCGATCAGTCACCACAACCACGCACTCCACTACAGGCTAACACAATGGCAGCTCCCGCCATCCTCCGCCTCCTCCCTCTTCTCCTCCTCGCCGCCGCCGCGAACGCCGCCACGTTCACCATCACCAACAAGTGCCAGATCACCGTGTGGGCGGCAGCCGTGCCGTCCGGCGGCGGGCAGCAGCTGGACCCGGGGCAGCAGTGGGTGATCGACGTGCCGGCCGGCACGACGGGCGGGCGCGTGTGGGCGCGCACGGGCTGCAGCTTCGACGGCAGCGGCAACGGGCGGTGCCAGACGGGCGACTGCGGCGGCGTGCTGCGGTGCGCGGCGTACGGGCAGCCGCCCAACACGCTGGCGGAGTTCGCGCTGAACCAGTTCAGCAACCTCGACTTCTTCGACATCTCCCTCATCGACGGATTCAACGTGCCCATGGACTTCCTCCCCGCCGGCGACGGCGCCGGGTGCGCCAAGGGCGGGCCGCGGTGCGAGGCGGACGTGGCGGGGCAGTGCCCGAGCGAGCTGAGGGCGCCCGGCGGGTGCAACAACGCGTGCACGGTGTTCAAGCAGGACCAGTACTGCTGCACCGGCTCGGCGGCGAACAACTGCGGCCCGACCAACTACTCGCAGTTCTTCAAGGGGCTCTGCCCGGACGCCTACAGCTACCCCAAGGACGACCAGACGAGCACCTTCACTTGCCCGGCCGGCACCAACTACCAGGTCGTCTTCTGCCCATGAGGGACGATGACTTGTTAGCTGGCATCTGATCGATGTGATCTTGGTTAGATGTATACACAATAAACAAGCATGCATACTTTTTATGCGAGTTTGTATCAGAATCATGAAGAGAAAAGTTACTCTGTAATAAAGTAAACCATTCAAAACTGAAGTGGTTTTTCACTTGTAGTTTGGTTAATTACACAGGCATGAACGAATCCGTTCAAATTCTATTATTTTTTCCAGGGGGGTTTGACGGGTAGTTTAGCACTTTAGCATCACTACGATGTACAAGTGAGAACTGAACAACGTTTTTTGTATGCGTGGATGACACGTTCTCTATTCGTTCCAAACCTTATGAACCGTGCTTGTGAACTGTGATCGAGGATTTTTGAACCCAGAATGTGATCGACGATTCATCGACCTAGCTCCTTTCTTATCCTGGGTTGAACGGTCTTTGTTCTTGGCAGAGCTCTGTATTTTATATCTTCAGGATAATAGCGATGGATAATATATAGAGTAAAATTTGTCCCTACCATATTTTTGGATTTTGTATCATCCAAGTCAAGATCAATTGACAATTCAGATATAAATTAAGATAGTACAACTTTATATTATCACGCAAAATCTTGGCACGATATTTGACCAAATCATTCACATCCATTTCTAGACGATTCTGCCAAATTAATACAACCAGCTGGACTCCTATCAAACCATCAGCTGGTTTCCCCACCAAACTCTCTTGGGATTTCGTAACGAAATTCGCACACGCAAAGCTGCAAAAGTTGAATTCCATTATTCCACATTATCCATTAGCCGGACCGCTATAAAAATCCACACCCCTAGCTTGCATTCTT

>Os03t0733800-00 2000 bp upstream sequence

CCAAACTAATAAACGATACGTTTTGTGTGAAAAAATATGTATAGAAATATTTTTCAAATGTGAGAATTTTTGCCTGGAGAGTAGCTTCAAACAGTTTAGCGACTTTGGAAAATAAAAAGAAGAGAAATTTGGAAGAAAAAAAAACATATATCTGTTCTATTTGTGACAGAGAGAAGGAGGATGCAGCTCATGCCCTGTGCAGAAATATTCATGCTAGCCATCTCTGGGCATCCTTATACAAGACAGACAATATCCCTAAGGAAATACTTATCTCACTTCCACGAAAAAAAGTAAACACTAGTACTCCTTTGCTCCGAGGAATCCTACCTTCTCCAAAGGTAGAATCCCTCTCACTTTAAAACACTAGAGTTTTGGGTACTAAAAAAACGAGTGGTTTCCGGGCTCGCCTCTTCGAGGGGCCTCCGCTCATGGACAACCTCACTCCGGGTCTTACCCACCCTCGAGTCCAATCTCGGGTGGGCGCTTAGGCATACCCTATTCACTTGGATATTCAGAATATTCAATTTGGCACTAACTGGCTTTTTGACTGTATTGAAGGTCTATATGCGTATGAGTGTGCCATGTTCTTGATGGTTCTTTGGAGGAACTGGTTTGTGAGGAATGAACTCATCCATGATAAAGTAGCCCCGCCAATTGAGGTCTCAAAGAGATTCATTGTAAGCTATGTAGACACACTGTTTTAAATCCGGCAAAATCCTTCTATCGACCTGGTAAAGGGCAAATATGGTTAATTCAGTCAAGGAATCGCCAAGATCCCATGCCTCGCCCAAGCCTCAGCTGCGATGGGAAAGACCACCAGTGGGGTGGATGAAACTAAACATAGATGGCTCCTTTGATAGTAGTGCAGAGAAAGGGGGTGTCGGGGCAATTCTTCGGGATAGTGCTGGGAAAACCATCTTTGCTTCCGGTAAGGCAATCGAGAGATGCAGTGAGGCACTGGAATCTGAACTCCGGGCGTTTATGGATGGTCTATCAAAGGCAGTTCAGTGGACTTTACTCCCAATAGTGGTTGAGACAGATTGCCTCATAGTTCTTCATCTTCTTGATAGCAAGTAGAAGGACCGTTTGGTGTTTGCTATCATCATTCAGGAAGCGAAGTCGCTTGTTGCTGGCGGTGATAGAGAGATTGTCATTAGGAAAGTGAGTCGAGGCCAAAATTCAGTTAGCCACTTCCTCACAAACAAAAGTAGGGTGAAGTCTTGTTCGTTTTTTGGTTGGAAGAAAATTGTAGTCCTCTTATAGTCTGTGGAATTATTGATTCTGAGTAATATATGTTTTTCTTCCCCGCACAAAAAATATTTTTCAAAAATCCAAATAAATCATTTTCTAGTTCACACTAATTAATATTTAATTAATAATATGATTAATAATTTGTCTAGTTTTTTGAGCCTTGAGCCGCCAACAGCCACCGCCGACTTGCCATATAGCCCTACCGAACTCGATTAGCTTTAGACAAAAAAAAAATGCTCCCGTGACTAGCGAATTGTTCTCATCTCTATTGTTGGTTTTGCATTAAGGTTAACTGCATTCTCAATAGGCTAATTTCTCTTAGAAAATTTGCCAATCAATTACATTTTTTTTGCGGGGATCAATTACATTTTATAATAAATAATCTAAAAGGAATATTTGATTTTGCTGGCCTCAATCATGTAACATTTTCGAGAGAAAGTTATTAGTAGACGCGTGTTGCCGTCCAGGACTCCATGGTTGTACACCTAGCAAAATTGCCTGCCAAAACAACAGGCGCGTCATTAGCGTTAAACACCTCTCGCAAACCCATGTGCCCCACGTCGCAGTGACACACAAATCCACTCTTGTAATCAAACCCCCCCCCCCCCCCCCCCCATTTGACGCCATAAATTCCAACGAACTCGCGCAACACGGGGAGACAATTTCTTCTGCGGCTCGTTTCCTTTCGTTTCGTTTCCAAGCCGCCCAGATCTCGCTTCTAGAACCTTCCACTCCAGGGCGGAGGCGGG

>Os03t0795900-01 2000 bp upstream sequence

CGAGGCAGACAAACATGCATGATAACAGGAAGAATCACAATTAGTTAAACCTCCTACTGGCCTATTAGTCTAAATAAAATTGACATAGTTATAATTCATCACATAACTTCTTTTTTGGGTTAAAGAGGCAGATCATGTCTCTATTATTTGTTCAGTTATTACAGAGGTCAGGTTCAAACATGGAATGTAAAAGGTATCATGAAACTACTCTCATCTCAGCATTCTTCTCACTACCATTAATCTACTTCTAAACCATAGGTGCGTGGGACTCCATTACAGGGCTCAGATATCAGATCAAGCAGAACTTGTAGGCTTGAAGACAACCAAAGCAGCTATCAGAAAACATTATATGTATAATAAGATGAACCTTAAGGAAAGGCAAAGCAATAAACAGATGGAACAACAGAGGGAAGAATTCAGCATGTCATTTATTAGGGAACAAGTCTACGTACGAAAGAGCCACATCGACATGCTACTGCCCTGCATGGTTTGTAGCGCATCACATACCAAAAGCATCAAGCTATTTACTTGTGGTGGAGATCATAGGACATATTCAAAATGTAAAGAGTGATTCCATTCTTCAAAATCAAGCATAATCTTGCATAATTGTTATGGATACTCAACTGAACAAATGAAAAAAAAATGAATAAAAAAAACGCCAACATATTTAGCAATGTTAGTATATGTTTGATGGAACAAGGAGCTTCAGATTCCTAATGCAGGCAGGCAGGCAGCGACCTTCTTTCATGGAAACCAAGAACCAACCCACAGAAGTGGTTCAGATACTCAATTAGACTAGAAAAACGTAATGAGTTATACCACACGCAGATGTAAACATGATTGATTCACAGAAATGAACAAGTGAAGGACGATAGGATTACCGCTGGTGTAGGCACATGTCATGCCGACGGCGAAGCTGATGGCAACCGTGAAGAGGCCGAGCAACAGCAAGTTCACCGGATGCTTCTGGTGGTAGTAGCGCAGAGGGCACAACACTATACACACAGAGTAATCAAATCATGCCAATTAGGGCACAAATTCGAGGAAATTCGACTGATACAATCCCAAAAAAAAACAGAACAAAGAGTGAATTGAACCCTACCGATGAGGGGCAGGAAGAGGAGGAAGATGTAGAGGGCGAATCCGGCGTTGGAGGAGACGAAGAACTCCGAGACGCCGCGGACCTTGACGACGAAGGCCGCCACGGCCGCCGTCATCGCGAGCTGCACGGAGAGGATGACGTAGATCTTGTGGATGAGCGCCCACCTCAGGTCGGGGCTCTCCACCATCCCCGGGTACAGCGGCTCCGACGAGCCGCCGGCCTCGAGATCATGCCCCTTCCCGAACATGGCTTCCCTCTTCCTCTAGCCTCTAGGGTTTGGCTCCGGTGACCCGACGACGACGGGGATTGGAAGCGGAGGCGGAGGCGGAGGAGGAGGAGAAGATGACGCAATCCGCGATTCCTCCGCGGCGGCGCCGCCGATTGGGGAGAAGAAGATGAAGATGAGGAGGAGGAGAGAAGACGAAGACCAACGCAACCACCAAGCACGATGCGATCGAATTTCAATTTGTTTTAATTTAATTGCTCTCTCGAAGTCCAAGTCAAACATATTTCCTTCGTGGGTAAGGTATATACGGTATCCGCTTATATTTTCATTTAGTGTGTGTTTTTTTTCTATTTTTCTTCCTGTTTCTTTTGTTTTTTTCTCTCTTTCACCTCCCTTAGTGATATTGTAAATACCTTTTATTCTCCTTAATATAACTCCAGCATAGCTTGGCTACGGTTTCAAAAAAAAAAAAAGGTATATACGGTATACGGCATCGTACAGTTATACGGGTATATGGGCCGGAGGAATCGGGTGATCCAGTCCAGGCCCATTATACGGTTAGATGGGCCGACTCCGCGTTTCTACCTCGGCCCAATCGGAGCCCGACACGTATGTCGTCGTCTCGTCTTCGTTATCTCCTCCCTGTGTCGCGGCGGAGGAGGAAAAATCCCC

>Os03t0802600-01 2000 bp upstream sequence

TCCCTTCTCCTTTTTTTTCCTCCCTTCTTCCTTTTTTTCCTCTCTGGCGGCCGCTGCACCGCGAGCTCCACAGCCACGCCTTCATCTCCTTCTCCATCAGCCTCCGCTCCACTTGCCGAGCCGTCGTGCTGGAGGCGCGCACGCGACGGGAGGGAGGACAGTGGCGCTGACGGGAGGGAGGGGGACAGCAGCGCATATATGTCCTACTCGATCTCCTCCGGGGTGAGCTCCACCAAGAACGGCGCGCGCTTCCGGCGTCGGGACAAGGACGGCATGGGCACCGCTGCCACCCATGACATGGAGGCGGCTGGGCGCCGTCGCGTCCGCTGTCGCAGGTTCCACGGCCTCACTGCACCGTCCACTTGCGGCTGTGGTAGGGCGAGATGTGGAGGCGGAGGCGGCGTCTTGAGATCAGGCTCAGAGTCCATCTCCACAGGTAGGCTCTCGGGCTTCGTCGTCGTCGTTGGCGGTGGCGGCGGTGGAGGTGGCGGGGGTGGGGGGGCTAGGCTGGGGCACGTGGAGACGGTCGACGGGTCCAAAAAACAAGGGAGAAAAAATTTCATGTCATCGTCCACGTGGCGTGCCACGTAGGCAAGACCACGGTCAAACGCGGTTTTGGACTGGGATGATACAATAAACCAAGTTTAGGAACCTCGAAGTGTACTTTGCAAGTTCGTGGACCTAAGTGAAACCTCCTGACAAGTTTAAGGACCGATGGTGCATTTTACTTTATTAAAAAAATACAATCTATGCTACAAGGAAAGACAAAACAAAGCAACAAAGTTTTTTTTTTCACAAACATGCAAAGCATGTCGATATCAAATACTCCTCTAAGAGTGATGTACCAATTTTGGTCTCGAGGACTGTAAAATTTCTCAATATTTAAAGGTACTTATGGTGTAACTTTTTTGCAAATGTGCAAAAGCTTTTAACGTCGATATCAAATATACAAGTGTCCTTAGGTGTGTTCTTTAGTACTCCCTCCGTACTCGTAAATAAAGTCGTTTAGGACAATGTTTAAGTTAAACCTTGGGAATATAAATCATGAATAACTCTCGAGTTGTTGAGTTTAAAAATGTAAAAATTATATGAATAGATTTGTCTTGAAAAATACTTTCATAAAAATATACATATATCACATTTCAATAAATATTTTTATAGAAATAAGAAGTCAAAGTTGTGTTTTGGAGACCGTGTCGCTGTCTTAAACGACTTCCTTTACGAGTACGGAGGGAGTACTAAATATCTAAGGATACTTTTGGTGTGTCAAAAAAAATAAACAAAAAGAAATTAACTAGTTATATCAATTAGTGCAATGCTATATATCTAGCTATGGCCCATGGATGACTGGATCTCCCGGCCTTTTCTTCTTTCCACCATCAATTGGTCGCTGTCAGCAAAGTGTCCCTTTCTCCTTCAGAAAGCGATCCACTCCTCTTCAGTTCAGGAGCTTGGCCGCCATGGCGCCACCACTCGATTAAATACACTCGAGCCGTCGAGAATTGCTTTAAGTTTTCTACGAAAGATGATGGCCTTACAGCTCAGATATAAAAACGACAACGGACAGCTAATCAGATGATGTTTCAGTCTCCAATTTCGTCAGTGATGACAAGAACGCTTTTGTGCGCACACATCTTTTTCCTACGAACACGACATGATCCGATCGCATCAGTGCGATCAACGGTTACGATTTTCCCTGTCTCTTACCTCTGGAAAGGTAAGATCGATGGTGCACTGCCAAAAAGCTTGACGAACATAAATGCGAAAGTTTGGCACTAACAAAAACGATGCTTTTATGAGTTTTTGGTAGAGTTTTTGCCCTCTGAAAGTCTGAAAATCTGAAATGCACGTGAACCCATTATGCACCAAAGTGCTCACAATTTGGTCTCCAATTCAGCAACTTTACGCGTGTACCTCACTACTTTGTGAGTGATCGATATCACTGATATTTTTGCACTCTATAAATATTGCTCTGTTGCTACGATGCTAGCAATGCAAGA

>Os03t0811900-01 2000 bp upstream sequence

CGTCGTCGTTGCGCTGGGCTGTAATGGCGGGAGCGGATGTAGTTGGCTGCTGCTGCTGCTGCGGCGGCGGCGGCGCCGAATGCGCGCTGGATGCGGTGGCGCCGCGGGGTCGGCCGCGGCCCCGGCGAGCGGTGGCGGCGGCGGCGGTGTTGCGGCGTCGGCGCTTGGCAGGTTCTTGCGGCGGCGCGTCTGGAACAACGGCATGGTTGCTGCCAGAGGTGGTGGCGAGCGGCGACGAGTGTACGGGAGCCGCCATGGGATGGCCGCTTCCTACCATGGCCACGCCGTCCGGCTGCTGGTGGTAGTACTGCATTTGCATGGCCGGCGGCGGGAAGAAGCGGCGGCCGGCGACGGCGGGAAGATGATCTTGTTGGTAGGCGTGCCCTGGATTGGCTTGGGAGAAGGTGGTTGGTTGCGGTTGCCATGTACGCTGCTGCTGCGGCGGCAAGGACGAGGAGGAGGAGGAGATCATCGCCGGCGAGGCCGGACTTACCGGCGACGACGTCGATTGCGTGTTTGCAGCGTAGGAGCCCGCCGCCGACGCCGAGTGGTATGGTGAGTAGTGGTTTGCCGGCAGAGGAGCTCGGACGGTGGCCATTGCCGGCGGAGGAGCTTTGGCGGTGGCCGCGGCGACCTCGCCGGCGACAGGGTTGATGGCTGCCATTGCAATGATTCACCGTTTCTCTGACGCCTACCTTTTTTTTTTGGGCTTTTTGGGATACAGTTAAAAAAGTTGTATTGTAATTACAACTCTACAACTCTGTTATTACTTATGATTAGTGTTTTGGGATACGTCTCCAACACTATATATATAACAGGAAAACATTAAATACTGTGATTGGTTCATTTGTGTGGCATTTGCTTGGACCTTTGCGGCTAATGATCTTTACTATCTTCCACATTTTTTCTCTTCTGTTTTTAGGTAATGTATAGTATGGCAGATGCTTCAAATTAAAGATGTGCCCCTCTCTCTTCTATATTAGTCCTCTGTTCCATAAAGTATTTGAGGTGAGTGAAGCCAAATGGCACACAATATATGTTCAGACTAGGATGCCTTTTTTTTTTGTGAGCACAAGATGGCATGAAATACTTATGATGAAATTGGGTTTTGAGTTGAATCATTTTTATATAATGGAATCATATTTCAATATTTTGTGATGCTTCAACTAGAATTTAGTAGTCGAAATCGACATTGGATGTAACAATATGATGGGGTGATTTGTATAAGTGTTCTGTTCGCAAATCCAAATCCTTCCAATGAATCGTGTGAAGTGGTTGGAAATACATTGTACAACACAAAATCTTTACTCCTACACCTATTCTCACCAATACATCGATTCTAATATTTTTAAAATTAAAGAACAACAGTTAGGTTCTTTACTAGATTCCCCCAAAGAAAAATACATGGTTTGATATGATCCGTAGGGTCGATCTTATCGAATGAACCATATCACAAGTTGATCTCCAGTTTTAACGTGTTGTTACCCTAAATTCCTTCGGTTTAGTCTATAGCTGATTTTCTAATTAACAAGTTTTGGTTTCCCACACAATGTCTTTTTTTTTCTCGATTCCATTCAGTCAGCAGACATAAATAGCTCAAGCTAATTTTATTTAAGTGACGTGAATTCTACGTGCACACTTGATCAGCAAAGTCCAAACAATTTCATTGCATAAAATTTCCAAAACATTTTTTCTCCTTACTAGGACCAGTGAGTGAGTGACAATGAACATACAATAGTGGTGTTTTTTTTTCAAAAAAAATAATGCTCGAATTTACATATCGTAAAAAAACACTTTATTTCAAGAAAAGAAACTTGCAATTTATGGGGAAAAAAGATGTAATTCCAAAAAGAAAAAGAAAGTTTGGTAGAGACCAAAAAAAGAAAAAAAAACTCAATAGTAGGGTGAGACGCGTTCATTTGACGCGACCCGCACAAAGGTTGGCTCTCCCTCGTTTCCCTTCCCTCGCATAAATAAAAGCTCGCACCACCGCCACAATC

>Os03t0823000-01 2000 bp upstream sequence

TATTTATCTCCTTAAAATATGTTTGTAGCTGGCTTATAGCCTGCTATTGTACCTGCTCTTAGGGCGTTTACAATGCATCTTTGGAATTTTTTTAGTTTCTAATTTTTTTTTCCAAAAATGTCACATGTAAAATCTTTGACACATGCATGGCGTATTAAATATAGATAAAAATAAAAACTAATTGCACAATTTGCATGAAAATCGCGAGACGAATCTTTTGAGCATAATTAGTCCATGATTAGCCATAAAATGCTACAGGAACTCACATGTGCTAATGGCGGCTTAATTAGACACAAAAGATTCGTCTCGCGATTTTCAAGCGAGTTATGAAATTAATTTTTTCATTCGAGTCCGAAAACCCTTTCCGATATCCGGTCAAATATCCGATGTGACACCCAAAAAATTTCTTTTTGTGAAAGACCCTTATGCTCTCTCATTTTTTGCAAGCACATTTTTAAATTGATAAATGATGTTCTCTTCTAAAGATAAGATAATTACTTTATAAAATCATATTAATATATTTATTTAGTTTTTTAAGCTAATATTTAATTAATCATACACTAATCTGTTGCTTTGTTTCACATGGTAGGAAGAAGGTTCTCAACAGTCAACCCCTTTTAAAAAAACACACCCAAGTACTCCAGCACATACACTTCACATTCTAAAAAATATATCTAATACGATATTGAAATTTATAGTACTCCTATTAAGATGTATTATACTCGCTCTATCTCATAAGGGCTTTTGCCTGGATGTGAACAATGAATCTGGACGGTTTTTCTGTCCAACTGTCTAGATTCATTGTATTATCTATTCAGATTCATTGTACTAGAATGTGTCACATTCAATCCAAAATCATTATATTTAGAGATAGAGGAAGTACTACCTCTATCCCAAAATATACTCCCTCCGTTTCAGGTTATAAGTCGTTTGACTTTGGTTAAAGTTGAACTATTTTAAGTTCGACCAAATTTATACACAAATATAGTAATATTTACATTACTAAATTAGTTTTATTAAATTGATAATTAAATATATTTTTATAATAAAATTATCTTGGGTCGAAAATATTACTATTTTTTCTATAAATTTGGTCAAACTGAAGCAGTTTGACTTTGACCAAAGTCAAAACGACTTATAACCTGAAACGAAGAGAGTAGCTATTTCTAGCACAACATCTTATCCCAAAATGAAACTATTTCTTCATCTACCTCCTCTCAACCAATCACAACCATTCTCTTTCACCTAATTTATCTTTTCAATCAATCACACAATTTCTTCAATCATTCTTACTTACTTCCTTAATACCCGTGCCAACCTTAAAATTATTTATATTTTGGATGGAGGAAATATTCTCTTCGTCCCATTTTAAGTGCAACTATAAGTTTTTGTGCCCAACTTTAATCAACTATCTTATTTAAAAATTTTCTATAATTAGTATTTTTATTGTTGTGAGATGATAAAATATGGACAGTACTTTACTACTTTATATGTGACTTATGTCTTTTAAATTTTTCAAAAAAATTTCAAATAAAACTGACGGTCAAAATTGTACACGAAAAATCATGGCTGCAATTAAAATAAGGGAGTATCTTATATTAGATTTGTCTATTTGAAAACAAAAAAGATCACGAATAGTATGCGAAGCCCCGATCGATCTCAATTGGCTGTCCTTGTTCTGTCTGCTTCTCATTTCTGAAGGGAAGGAAAAGAAAAAACAAGTGGAAAAGGGGCCCAGAGTGTGCACGCACTCCCTGCAGGCTCTAGCACAAGCTTAGCTAGCAACGTGAAATCTTTGTAGGCAGTTGAAGCTTATATGTGCTGAGCTCCTACCCTCCAATGCCGTCCATCTCCATGTCCCTCTCGCCGACGTCGCCGTGCACCTCGCATCGTCGCCGTCACCGTCGCCGACGACCAGACCAGCAACAGTGCCAGCGACGGCGGCTGCTGCCCAACTCCAACAACTAAACTGTTCAACTCCTTCAGCTACTACTTCACTT

>Os03t0838800-00 2000 bp upstream sequence

ATACATTTAAGACTAATCTCAAATAAAAGAAATAAAAGAAATGTGGTTGTGTAATTTTCAGTCACCTAAAACTACCCATAATTTTAAGTTGTGTTATGCATGGCTAGATTGCATCAATATTTGTGTGTGCTACTACAGTAACGGACATCTTCCCCTGACTCCAGATCTGATTGAGCCACATGCGTTATAATTTGTGCTTTTTCCTACCCCTTGCGTATTGTCAACAAGGCTTGCCCTACCGGCCTTCTCCCTTCCCCTCCTCTTGCTCCTCCTAAAGCCCAAGCGGCGGTAAAGTACCCCCACCCTGCTTGTTTTTTTTCCCGACGGCTTGTGCTATGAGACACCGCCACCACTGCCACCCTCATCTTTCTAACTACCTCCTCCGTTTCATATTATAAGATTTTCTAGTATTGTCCACATTCATATAAATGTTAATGAATCTAAACCCATATATATGTCTAGATTCGTTAACATATATATAAATATAGGTAATATTAAAAAGTTTTATAATATAAAACGGAGGAAGTACTCCAGATCGGAAGCACCCATTGTCGACCTCGTCCACCGTGGCTCCTCCAACCCCTCAAGCCACCTGCCCACGTCCACGGCCGATCGCATGTCCTCAGCGCTTCCCGTCATCCTTCCAAGTTCATGGAATCCCCCTGTTTCTTCTGCCTTCAACCCCCTAACCTCTAACTTAGTATCGCAGCCGCCATTGAGTCCTTTGCCATTAATTTCCCTCTCTCTTTTCTCCTATTTCCCTTGATTTAGAGGTTGGAGGTGAGCGAAATTTCTCACAATTTTAAGTTATCTAAAGCCTAGAGATCTCCAAAACAAAATAAGTACCAGTAGCGTATGTGGACTGTGGAGCATACGTATCGTGTGTAAACACTTCTGTTACAGCACAAATATGCGGTTAGCCCGCTCTTAATAAAATCGGTTGGTGTGAACACTCACAGATACTCAAGAGGAACACAATACGGTAATACCCGGTGACATCAACCCAACTTCCCAAGCATATGCATCATGCTTACCTTGTTACTTATGCTGTGGAAGGAAGGTGACCTTGCTGTTCTCTTTCAGAGGAAAGGCAAAAAAATGATCAGTGAAGGAATGGTGATCGGCAAGTGAGGTCAACACATCCATGGGGCCTTTTAACGCAACGTGTACCCAGTAAAATTATAAAATACTCCATCCCCTTTATATTATAAGTCGTTAACTTTTTTTCTAGTCAAATTTTATTAAGTTTAGCTAGATTTACATAAAAATTAGTAACATCTTAAAAATCAAATTAATTTCATTAAATCTAACATTGAATATATTTTCTAACATTAAATATACGGAATTGCTATTCATCTGTTGATAGATTTTTTTGATAATATGTTTGTTTTGTGTAAAAAATATTAAAGAAGTTTAATTAGAAAAAAGTGATTATAATATGTAAGCTTTCTGTTGTTCAAAAGGGCAAAATTTGGTGGCTTTCCTGACCAAAGCAGAGCACCAAGAGCCAGGAAGAAAAAGCACGAGATCCTGAAGAAGAAACCAGTCAACCAACTCGTGCACATTACATTTCCATGCCAATGCTTCGGCTTTAGTGACGCAAGAGCTTACCATGCCGTCGACCTAGTCGCCGCCGCCGTGGAGCCCACACACCTTATCAATTGGAGTATATATTACTGCCTTGATTAGCTCAGCTGTCAGCTCGACTCACATTTCATCACCGACAAGCAGAAGTGATCTGATCAGGAGCACAAAAAAAATCATCTGACACTCGATCGAATTTTTACCTTTCTTTGATCAGCATTGTCATTCTCATCATCTCAGGGTAAGTGTTATGCCATTGCCAATGCCACTCTGAACATGTCCATTTGGGTTCTTGATCATTGATTCAATTTTTGTTGGCATGTATTTCAGGGGAAGGATCATATTCATCGTTGTTGTCGACGATGATGATGAGTAGTAGTGCCGATCATTTCAGCAACAATAATAATAATCAGGCG

>Os04t0127200-01 2000 bp upstream sequence

GCCTCCCCTCCCGATGGATCTAGCAGAAGGGAGGGCGTCGCCGCCGCCTCCACTCTGGCCGACGAGCCGCCATGGGCTCCCCTCCAGCCGCCGGGCAGCCACAGCCGCTGGGTGATGCGCGGTGGAGAGAGAGGGAGTGAGAGAGAGAGAGCGCGCCCCTCCAGCTCCCCGCCGCCACCTCCCCTCTTGCCGTTGCTGGCTCGCTGCCTCCCCTCCCGCCGGATCTGGCGGAGGGTAGGGCGCGAGATGACGCGAGATCGAACCTTATCTCCTCCTTGCCTTGTCGTGGTGGGGCCCGCCTGTCGGCTCATCCGATTGCCGGCTCGGCTCGATTTCTGTGGGTGTCGGTTTTTAATTAACTAACACCTATACTATATTAAACGTGTCGATTCTTTTTTCAAAAAACCGACACCTATAGAATAGGTGTTGGTTCTTATTTAGAACCGACACTTATAATTGCTTAAAGGTGCCAGGTTTTTAGCGATTTTAACCCGCGAAAATAGGAAAAAGGCCTATAGGTGTTGGTTTTAGCACTTCCGGCACCTATAGTAGCAACACCTGATGTTTTGTAGTAGTGCATGATATTGGTTCTTCCAATCGACAGTATAAGAGCAGGTACAATAGCAGACTATAAGCCAGCTATAAACACATATCGAGAAGATTAAAGAAGAGAGAGATGAGCAATAGGCTACATATATGTAGCCAGCTACAGCACGAACTCCAAGACGCAATGTGGTTATGACATGTGGGACCGGATATTAGTAATGTAGTATATATTTATAGGTAACTATTGTATGAATTAGCTATTAAATCGACTATATATAATTTAGAGCTAATAGTTGGCTATACTATTAAACTAAAACGATGTCACCGGATATATAAGTCGGAAACCGAGATTTCAAAGTAGCCACAGACAATTTCATCATGGATAACCCAATAGATCTTCATAGGTTACCTTGTGAATGGGGTAGCCACCAAATTAAAGATAGAAAAGACTCTAGACAAGTCTAAGACACTATTTGGCACAGCTCTAGATTGTAGATCCACATTAGATTTGAAATTATGTGTTGAATTAAAGTGGTTCAAAATATTAAATTTCATCCAAAATGCACTACTAGGAAAAGGGTTTTTCAAGGCGGCATTCTATTTTTTTCACTGGCGGCTAGATGATAGTGCCGCTTGCATTTTCACCGGCGGTTATGTAATGTGAACCGCTAGCGAAGATATGCCATTTTCTCAAGTGGTCCTCTTAAGTGACAGCTAGCCAGCCATCGGAAATTTTCGCTGACGGCTGTTAAGACCCTGCTTGCGAAAATTAATTTTTACTAGCAGAGGGTTGCCGCTAGCAAAAAAAAATATATCTTATAAGGTCGCGTGTAGTAGATACAAACCCGAGATTATTTTTTCCTCTTCTCTCTCAGCTCTCCCTCTCCTCTCCTCTCCTCTCCTCAACCCCTCCTCTCCTCACCTAGATCTGCCCTCTCCCCAGCGTCAAGCCGTGGTGGTGGCGGACAATCGGAGAAGGGAGTTGGGAAAGGCAGGGTTGGAGAGAGAGGGGAAGGGGGATTGTTTATTATTTCTGCATTCTAGAGTGTTCAATGCAATTTTTGGACTTTTCACACTAATTGAAAAAGAAATAAGAACTAAAAAAACAGGGGATGAAATATAGAATTAAAGCCACTCCTAAAGCCACTTGCACTGTAAAGAACCTATATTTTGAATCTGTAACTTAGAATACGTGTTCTGATGCCTCTCGTATGAGTGTCATACCCTGGGATTGGGAGTGTCCTAACTTTGAGAAAAATTCTAACCATAAAAAATGATTTCATCCCTCGAGGAGATATTCCCTCGTTTCGTGTATGTAACCTAAATGGTTATAAAAAAAATTAAAACAAATCTACAAGATAGATTAATATGGAAATATCACAAAATCTTAAAAGTTTGGAATGTTTCTTCCTCCTACCCATGAGGCCATGACCCATCAATCCATCATCCCTTTC

>Os04t0127200-02 2000 bp upstream sequence

GGGTGATGCGCGGTGGAGAGAGAGGGAGTGAGAGAGAGAGAGCGCGCCCCTCCAGCTCCCCGCCGCCACCTCCCCTCTTGCCGTTGCTGGCTCGCTGCCTCCCCTCCCGCCGGATCTGGCGGAGGGTAGGGCGCGAGATGACGCGAGATCGAACCTTATCTCCTCCTTGCCTTGTCGTGGTGGGGCCCGCCTGTCGGCTCATCCGATTGCCGGCTCGGCTCGATTTCTGTGGGTGTCGGTTTTTAATTAACTAACACCTATACTATATTAAACGTGTCGATTCTTTTTTCAAAAAACCGACACCTATAGAATAGGTGTTGGTTCTTATTTAGAACCGACACTTATAATTGCTTAAAGGTGCCAGGTTTTTAGCGATTTTAACCCGCGAAAATAGGAAAAAGGCCTATAGGTGTTGGTTTTAGCACTTCCGGCACCTATAGTAGCAACACCTGATGTTTTGTAGTAGTGCATGATATTGGTTCTTCCAATCGACAGTATAAGAGCAGGTACAATAGCAGACTATAAGCCAGCTATAAACACATATCGAGAAGATTAAAGAAGAGAGAGATGAGCAATAGGCTACATATATGTAGCCAGCTACAGCACGAACTCCAAGACGCAATGTGGTTATGACATGTGGGACCGGATATTAGTAATGTAGTATATATTTATAGGTAACTATTGTATGAATTAGCTATTAAATCGACTATATATAATTTAGAGCTAATAGTTGGCTATACTATTAAACTAAAACGATGTCACCGGATATATAAGTCGGAAACCGAGATTTCAAAGTAGCCACAGACAATTTCATCATGGATAACCCAATAGATCTTCATAGGTTACCTTGTGAATGGGGTAGCCACCAAATTAAAGATAGAAAAGACTCTAGACAAGTCTAAGACACTATTTGGCACAGCTCTAGATTGTAGATCCACATTAGATTTGAAATTATGTGTTGAATTAAAGTGGTTCAAAATATTAAATTTCATCCAAAATGCACTACTAGGAAAAGGGTTTTTCAAGGCGGCATTCTATTTTTTTCACTGGCGGCTAGATGATAGTGCCGCTTGCATTTTCACCGGCGGTTATGTAATGTGAACCGCTAGCGAAGATATGCCATTTTCTCAAGTGGTCCTCTTAAGTGACAGCTAGCCAGCCATCGGAAATTTTCGCTGACGGCTGTTAAGACCCTGCTTGCGAAAATTAATTTTTACTAGCAGAGGGTTGCCGCTAGCAAAAAAAAATATATCTTATAAGGTCGCGTGTAGTAGATACAAACCCGAGATTATTTTTTCCTCTTCTCTCTCAGCTCTCCCTCTCCTCTCCTCTCCTCTCCTCAACCCCTCCTCTCCTCACCTAGATCTGCCCTCTCCCCAGCGTCAAGCCGTGGTGGTGGCGGACAATCGGAGAAGGGAGTTGGGAAAGGCAGGGTTGGAGAGAGAGGGGAAGGGGGATTGTTTATTATTTCTGCATTCTAGAGTGTTCAATGCAATTTTTGGACTTTTCACACTAATTGAAAAAGAAATAAGAACTAAAAAAACAGGGGATGAAATATAGAATTAAAGCCACTCCTAAAGCCACTTGCACTGTAAAGAACCTATATTTTGAATCTGTAACTTAGAATACGTGTTCTGATGCCTCTCGTATGAGTGTCATACCCTGGGATTGGGAGTGTCCTAACTTTGAGAAAAATTCTAACCATAAAAAATGATTTCATCCCTCGAGGAGATATTCCCTCGTTTCGTGTATGTAACCTAAATGGTTATAAAAAAAATTAAAACAAATCTACAAGATAGATTAATATGGAAATATCACAAAATCTTAAAAGTTTGGAATGTTTCTTCCTCCTACCCATGAGGCCATGACCCATCAATCCATCATCCCTTTCATCTCTCATCTACCTCGCCATGGTCGCACACAAGCCTGCGCCATGCCCTGAGCAGCAGCGCTGCTCCCCCCCCCCCCCCCCCCCCCCCCCGGCTTCACCGCGACCGCTG

>Os04t0311500-00 2000 bp upstream sequence

GCCGTGCCCCCTAGCGTGTACAACCTGCCAAACTGCCAATCCCCGTCGTCTCGTCTTCCCTCCGCACAAGACAATCCCCCAACCGAACACCCACGCCAGCCGCCGCGCGCTGCCGCCCCATCCGCAATCCACCACCCCGAACACCACCAAATTTCCACCGATCCCCCCACCCCGACCCCATTCCCCCACGCCGCCGCCGCCCGCAACGAAAACGAGACCAAATTCCCCTCACGCCTCCTCCTCCTCCTCCTCGGTTATGCGGTGATGGCGCGGTGCCCCGGCGCGGCGGGCGTGGCGGAGCGGTGGACGGCGCGGGCGCTGGCGGGCGCCGTCCTCGACGTCGCGCTCGTCTGGGCCTGCCTCTGGGTCGCGGCGCCGCCTCCGCCGCGGCCAGCGCCCTCGCCCTCCTCGGCGTACCCCTCCCCTGCACCTGCGCCCGCCCCCACCTCCCCTGCCTCCTCGCCTTCCTCTCCCGCTACCCCTCCCGCGCCCTCGCCTCCCTCGCCTCCGCCCTCCTCTCCCGCTTCCCCTTCGCCTCCTCCTGTTCCCCCTCCCCCTCCTCCGACGACGACCACGGCGTCGAACACGAGGAATCGGGCCGCGTCGATGGGGTGGAGGAGGTGGACGAGCACCCGGCCGACGACGGCGAGGTGAGGCGGGAGGCGGTGGTGGTGGAGGAGGAGCGGGACGCGCGCGCCGCGCTGCAGCGGGAGCTGGAGAAGGAGCGGAGCGCCGCGGCGTCGGCGGCCGAGGAGGCGATGGCCATGATCCTGCGCCTGCAGAAGGAGAAGTCGGCGCTCGAGATCGAGGCGCGCCAGCAGCGCCGCACCGCCGACGAGCGCTGCGCCTTCTACGAGGACGAGGTGGAGGAGCTCCGGGACATCGTGCTCGTGCGGGAGCGCGAGTCCCGCGCGCTGCAGAAGGAGGTGGACGCCTACCGCCGCCTCCTCGGCCTCGCCGCCGAGGACGGCGACGACGACGACGATGAGGATGATATGATGACGCCTCCTAGCACCATGGTGGAGGGCGAGCCAAGCACGTCCAGATCTGTGTATAAGGATAGTAGAAATGGCGCGATGCCGCAATTGGGGAATGACTATGGGTTCAGCTTGAAGACGCCGTTTTCGCCCCAAGAACTACTGTCACCTATTAAAGTGGGTCATATCAAAGGGATCAATGAGGATAAGCTGTCTGCTGGGCCATCTGAGAAAATTCCCATGGTTGGATTGAAACCAGAGGTTGATAGCTCCAAGGATGATCGCGTGGAGACGATTGAAATCCTTCCACTATCTGCTCGAAGCTTAGATCAGGTTGGTTATGGTGAAGTTGTTGCTGCAGTTGATACAAAAGCTCCAGAGCAACTGCCTCATGAGTTTCAGGAGGAGTGTGGAGGTGTAGACAAGATTGGTGGTGATTCTACGGGAAGTGAGAATGATGCGAATATCTATGATGTGCATGTGGTTGATGATATATGCTTCCCAACTGAAGGTATGAGATTGTGGATAAATAAATCTCAATTGGTTGATTCTTATATCAAGTATATGGCTTGTGGAATCGTAAACCTGTTAAATTCATTTTTGGGATGTCTTCACTCTTCATAGATGGCATGCAGTATTTACGAATTACTATTGGTACATGATAGGTTTATTTAAACCTTTCTTTGCTTAGTTTGGATAAATGTTTCCTGTTATTTGATGAGAAATTCGGTACTTTATTTACCATGCCACCTGGTCACTAGCACTGAAACCAGTCCTTTGTATACAGTTAAAGGCCTGATTTGCCGAAGTTTCTCAGATGCAACAATGCAAGCAGAGAAGTTACAAAACAGAGTTGCTGCAGATGATCTCCTTGGGAAAAGTCTGAATGCCATTAAAGGTGCACAAGACAAGATAAAGCATGCAGCAAGTGAAAGAAAGCAATCATTACAGCTGCAACTCTTAGAGGACATAGCTAACCAACTTCAGGAAATCAAGGTTGCTGCAGAAGCAGGGCGACAGCTG

>Os04t0370100-01 2000 bp upstream sequence

TTTGAAAATTTTCGAATCTGAGTTGAAAGTTTTTGAATCTGAGTTGAAAGTTTTCAAATCTGAGTCGAAAGTTTTCGGATTGGAGTCGAAAGTTTTCGAATCTGAGTCGAAAGTTTTTGAATCGGAGTCGAAAGTTTTCAAATCTCAAGTTGAAAGTTTTTAAATCTGAGTCGAAAGTTTTCATATTTTTTCAAATCCGTACTTGAAAGTTTTCAAATCTCGAGTTGAAAGTTTTCAAATTTGAGTTGAAAATTTTCAAATTGAGTTTTGAAAGTTTTTAAAATTTGACTTCAAAAGTTTTCAAATCTAACTTGAAAGTTTTTAATCAGTTAGTAGAAAAAATCTTTCGGAAAAAAATCATATCTTAATTACCGTCATTATCTACTAATCACTTAGTTAATTAAACTAAACTATCGGAAATGCTCGCCGCCAACGCGCACGCGCCACTTGTGCCCGCGACGGTGCCGGTGTTTGCTTCACCTCGTGCCACGGCCCAGCGGCGGCTGGAGCGGCATGCTCGCTGGATGGTCGCGAAGGAGGAGGGCGAGGCCGCGGGCTAGGACGCATTGTCGTTGGCCCGTCCTGACAGCATGCGAGGCGGCGGCGTGTGGTCCACTCCGCGTCATGAGGTTCTGGCCGTAGCGACGCGTGGCCATGGGCGGCGCCGTGAGGCTGTGGCTGGAGGTGGTCTGCGTGTTAGCGTGGGTCACGCTTGGTCACTCGGGCCAGCAGCACAGTGACTAGGCTTGGGTGCTGAAGGGATGGCGTGGTGGTCCAGCGACGGTCGTTGAGCACAACGGTTGTATGGGCTTTGATTGGGAATCGCTGGTGAAAACCTTGCCAGGCCATGGGCCGGCATGACGGCGACTATGCCTTTAGGCGTCATTCCCTTTCTTGGAGGCGTCGTTTTGGCGTCGATCCTTCTAGCAGCAAGAACCTTCCGTGTGCAACCTAGCCATTGGTGGACTCCTGCAACACTTCCAAAGCTCTATAGGCCTAAGGTGTTTGACGAGGCCTTCGCCGCCTTGGTCTCGTTTCTTGCTCTGCTGCGGGTGTTCATCATCCTTATTTGGTTACTGTTGGGTAGAGTCGGAGCTGCTTCACTGTACGGGTGCGATGAAGCTCAGCAACGATGATACACTGCAATCTCCTTATAGGGTTGTTGATGCTAGCTATGTGTAGGAAGTGGCTCCTTGGTAGTTTGGGCTGATGTGCTTCGTCAGTGTCAATTTTGTTTGTTCATTTTAGCTTTGAGGTAGCATCGCAGTGAAGTCGAAGCTGCTGTGTTGATTGGGTGGCAAGCTCGGCAACGATAACATGTGTAGGTTGCTGTGTCGCCTCTATGGCAATTTTGGTCGATGCCTTATGTTAGGCTCCGTCGTGGTAGCATATGTTAGGTTAGCTATGGTGTGGTCTTGTAGTTATGGTTTTCGCTCAGTTTTTCCTAATTAACTAGGCAGCGTAAGGTTTGATCCCGTTTTCTTCTTAAAATTGGACACCTTCTTAATATAAAACGTCGGCAATGGCCTGACCATTTTAAAAAAAAAATTACGTCTCCGGGCCTCTTTGCATGTCATCTAAATTACATATTTAATTATAATGGTTATTCAAAATAATATGTTTTTATGATGGTAATGCCACTTTTACCTATCAAGTCTTAATATTTATATCAGTATATATCAGTTAAGATTTTTAGACCTGGCTATGCATATATTCCTCTAAAATATATTCCTCTAAATATATATGTCTATTTCAAAACACTACGTGTACTCCGGAACAGTTCAGCTCTTAAAGAGAACAGTGTAAAACGACGTTGGAACGAACAGTCCTTAGCGAAAACTTGCCAAGTCAAAGTCGTCGGGTGTGTACATAAATTTACATATCATTGTTTATTTGTTTTAATTTTCGTGGTTAATTAGCACCCAAGTAGCTAGGTAGGACATGATAGTGCTACACCCCTCTCGTCTTTATATACGATTCCTCCGTTCCTTGTGAAAACT

>Os04t0382300-02 2000 bp upstream sequence

GAGATAAGAAGAGTTAAACAAACATCTCTTTTGTCTCTGGAAGATTGGGGGGACAAACGGATAAGCATTAGGCACTGCCGATGACGATGCAGGTGGGCCCATTTGCTCTAATCTGCCGAGATGAGGATGCAGATATGAGATATTTGTCTTTCTTAACATAAATTATCTCGTGCACATATCATTCGTCCCTTGAAAAAAGTTATACTACCAAAGTTGTTTACACACGGGCTTTTGATTATAATAACAAGTAAATCAAGCATATGTCCGGCCTACTACTGAAACAGTTAAAACAGTATTTTGTGATCAATATGAAACTCTGTAAAGTTTATTATAGAATAATAACCGGTATTCAACGAAAAAATTGTGTAACATAGATATGTTTATACGTTAATTAAGTTCCTTACGTTTAATCATATGACAACAGGATTTTGAGCAACTATTTTTCTTTTTTAAAAAAAGATCTAGTCATTTTTCTCATCCTTGGACGGAAATTAAAGGTTGAACAATGATTACTGTAACTAAAACATCCAACAAAATTATATAACTAGTAAAAAAAATCACAAACTATACAAGTACCCAGACTTGCATAGAACACCACTTTGAAACCATATAAAATAAACTAAAAATGCACATTATATAGGGGTATAATTGCTACGTGCAATTTTAGGTGACTAGAAAATTGTATGAATTTCCACATGACTAAAGTCCAGCAAATCCTTATCAAAATTGATGTTGTGATAAGAATTTTCCCAAAAAATTGTGACCTATCGAGTTGCTGTAATATTTATGGCCTATTTCAATTTGCTATAAAAACAGCAAAGAAGATTATCCCAAAATTATAACTTTATGGACACCTTAACATTACTAACCCCTGGAGCCAACATGACATAGAGGTGATATCCGGCATGACGAGACGACTACGGGATATGTTAGCATGACATATCAACATGTGCATTGGAGCACTATATCACCTTAGCAGACAACTAAGCCACCTACTTTAATCCGGCTGTAACTTCACTTGTACAGTCGGTGCAGCTGGATCAAAATGCATCAAGCTTAAAAAGGGCAAAAAGACAAGATGAGAGGGCCATTTATCCTATCCGGGGATGGGTTTTTGACCTAGGTGCACCAATCAAGTGACGTTTCAAGTAGACTTGATTGCAATAGTGTAACCGGATCGGAGTGACGGGCCGAGTAGTTTTCTAATGTGGCATGCCACTACAATTTATACATTACTAATGTATCAGGCTAACATGTTCCGCTGTCTCGCCCATGCTAGGTATCATCTCTATAACATTCCTTAATATATTATGTTAGGGTATTCATAGAGTTGTTTTTTTTTTTGATAATTTTATTTGCTACCCTTGTAACAAACTGAAATGTGTCTCTAACCTTGCAGCAAGCACTTTTCTTGTAGCTCCAAAAAAAAAACTCCAGTTTTCTTGTGCCTCTAGCATTTTGCAGCTTCTACACGCCGCCCGCCAGCTCAGTTCACCGGATCCCCGGGAATTAAAATCACACGAGATCGATCAGATCGCACGCTGAGACGTATAGACGACGTGGGTCCCGCCAAAGTACTGGGCCCACCAGTCATTGGACCACCGTACGACGACATGTGTGCACCTACCGTGGACTTCGCTGGAAAAACCACCGAAGCCGCCGCCACGTCATGCCCCGGCTGCGGTTTTCTTTAATAAAGCCCAGAACACAACTGCCACACCCACCCCACTTCTACCCACCCTCAATGACGAGTGGGCCCCACGCCAAAACCGTCCCCAGGACGTTAGTGTCCGTAGCCGACGCGCGCCGCGTCGGCTGACATGCGGGCCCCACCTCCTCGCGGTCGGATCAGCATTTCGCCTTTCGTCGTCGTCGTCATCATCCACTTCCACTTCCACCCTCCTCTGCATTTGCAACCACGCGCAGACAAGAACAAGACGCGCAGCAAAGCGCAGCAGCAGCAGCAGTATTAATCGAGGCCAAGGACAAGGAGCAGTGGC

>Os04t0454200-01 2000 bp upstream sequence

CGAGATGCACTGTCTCAACCGTGTCGTTTGAGAAGCACTCACTTAGTTGTTTTCAGAAAAGAGTTCAAATAAAATCAATTGCAAAAACAACAGCCTTTCCTTGAAGCCTGCATTAAACACTTATTTCCCATGGCTTGCTGAGTACTTCCGTACTCACCCTTGCTCTATATAAATAATCCCCCCCCAGTTGCTGAAGAAGATGAAGCGGATCCTGCTGATGAGGAGTTCTTCCAGGAGCAAGCCGGCTACGATGAGTTTTAGGGTTTCGGCCTAGTTCCCAAGTCGCGCCTGTGTTGTTTGGTCCAAGTCCTGGCTTCCGTTTCCCTTTTGTAATGCAGTTGTGAGCTCGGGATCTGTCCGTAGCCCAACATGACTGTACCTCTACTCTATAATAAAGAGACCTCTGTTGCTGTGATAATCCGTCTTCCTGTGATACCAGCACTATTTCCTGGGACTGGTATCGATTAACAGGTTAATTTGGAGCGTCACGGGCTAGTTCCGGTCGGTACTAGTCCGGGGCGTGACACTGTAGCAGCCGTCTTCGCCTCATCAGCCGCCATCCTTTCCTCCATGGCCCACAACGATGTCCGCCCAGGGAGGCCGTCGTCATCCTCGTGGTTGGAGACGGCGATTGGTGCGCACAGGCGCATGGTGTAATTGGGCACCCCAGCGAGGTGCACGGTGTCTCGCGTGCTGAGCGCCTCGATATCCGTGCACGACACCACCTCTGGGCACATCTTCTATAGCTCCGTCTTGATCCCATTGATGATGTCGTAGCCCTTCACCGATATGTTCGGCTAGGCCTCCTGCTTCGCACTCTAAATCGACGGGCGCCGTCCTCCAACAATCCGTTGAGATTGGAGAGAGAGAGAGAGAGATGAAGAGCCACGGACATGTGGAGCCTACGCTGACTCAGTGTTGTTGACTTGGGTCAAATTACCACGTAGAATAAAACCTCTTGCCATACTATCTTGGGACTTATATTGTACCGGTTTTAACATATGAGAGACCCGTTGTATCTAGTTTTGTGGTTCAAGGACGAAAATTGGATTCGTTGTTAAGTTAAGGGATCTGAAATAACCTTATTCCGGCTGCAAACCGTTGGTTGGTGCGTCTTCTTCTCTGTAGTACAGCGAAACTAGCCCATATGCACGATTTTCTGATGGCCCACTATGACATTCCTCCTACCATGCATTCTCTTTCAGCTTTGTTCCGGCGAGTTGTTTTCGTTACTCCCCTTCTTCTCGGGAGGTTTGTGCGCACTGTGCAGGCGTTCATGGTAGTTGGTGGACATCGCACACAACTCAACATGCCCACACAGGAGTACAAGACGGACCAAGTTGACGTGAAAAATCTCAGCCTTCACTCACAACAACAAACTACTCAAACTGTGGTAGCTGTCGTGGCGAGCCTGAGGGCCGTGGTGTTTCGGCTAACCGCGAGATAGTGCAACAAATTGTTTACGTGCGTCAGGCCGCCGCTGTGATGACAGGCTGAAGGGGGAGAGCAAACCAAGCCTTCTGACGAAAGCTAGATGGGAGTCTGGCTATCAGCCAACCACAAACACAATGTATGTATGTATGCCTTGGTACATATGATATCACACATTAGGCCATTAACTCAGATTCAGAACTAAACGAAGCCACAGGCCTGCAGCGGTGCAGTTGAGCCATTCGATATCCACAACAACATAGCTACCAACCCATGAAACACATCGCAATAGTAGCAGCGCGCCTGCCAGTGAGGATCTTCTCGAGTACGTTTAGTTTGTACTTTGTACGTAGGGCCTCTTCAAGAATACGACTGAATAATTCCGCCCCAACAAAAACGAAAAGAAAACGAAACGAGTTGATAAAGCAAGAGCAGCCGAACGAGTTAATTTCCAATATAGTTTGGCACGCGAATACGCGATTGCTCCGCGCGCTTACCTAACACAAGTCCTCCCGTACGTGGCCGTGGATATACAAACACCGGACACCATGATCGATCGGCCTTTGCTA

>Os04t0463500-01 2000 bp upstream sequence

TATTGTTTCTGGTACCGTGGTACCAGATTACATAGCTGTCTTGTATATCTGATACCGTAGGTACTGTGCTGACAATCTGACATTAGCACACATAAATTATTCCGTTCGAACGGTATAGCGTTTATGCCGTTCTGTGGTAGTTTTCTTTTTTCAGGCTGCTCGACTGTGACGGCGACTAGCAGAGGCAGAGCAACTTTTCCTTGCCTTCCTGGAACTTTCCCTATCTCTACAATGCCGGCGATGGCCATCCGTTTCGTTTCATCTTGGATATCCAAATCTGCCCATGATGCAAGATCATGAGACAGCAGGTGGCAGCAGCATTCGACCGCGACTTGGTAAACGGCAGTGCTTACTGGGAGTCTGGGGCATAGTACTTGTGCCGATGGCGGCATAAAATGTTTGTTTTGGCTGGCAGTCTTCAAGCGAGCTACGTACGTACCACCACATTTTCAAGCGATTCCGATATATCCTTGTCCCGTCCCGAGAATCGCCATCGATCTCCAGCATATATCGGAATCGAACGTCGTTTTCTAGTTAGGCAGAGATCGGTTCGGGGCTTCGGGCGGCACATGCAAAAAAAAATAGATCGAAAGCAATTACAAATACGCACCCTCTGAGTTCGCAGGTTAAAGTAGCTATAGTGGTCAGAACTCAGAACACAGGATGCAGCACGACCCAGCCAGATTGAAAAGTAATTTTACAAAAATATTTTTCTCTCTTTCCGGGTAAGGTAAACGTACGCAGTACGATCAAACAGTTGAAACGGTTCAGTTCCGACGAGCAGGAACAGCTGCTGCTGCTGATCAACCCACTCACCGTGCGAAGTTTGAAGCCGAAAATTTGACCCCCCACATCCCTTCTGAATTCCAACGTTTAGACCACGCAAAATCCATTCCATCCAGTCACAGCCGGCGGACCGGCCACCTACCAACCCATAAACCCAGTTGCTCTTGCCATCTTACGTACACAGCGGGGCATGACCGGCATCGCCTCGCCTTGCCCCATCCGCCCATCGATCTCCCGACTCCCGTCCTCTCGTGCAGCTCGTCAGGCTGCACGCTGCAAAATGTGCGTGGGCCATCAGCATACAGTGATTGCCTCACTCAGGGGTCTTCCGGTTAGCTTTATAAGTTAACGGACTAGCCGATCTGAGTTTGAAGTCCCATCCCTTTTAATTATTTGAGATTAAGTCCTTACCTACTATTCGCGTCTTGGCTCACGCACGTTTTGATGTTTTGCGGCGTGCGAGTGGTGCGGGAAACCGGGAGGAGAACAACGAACGGGTGAAGTGACATGTTAAAGGCAAACGATGGAAAGGGATCGACGGGTGCGAGGGAGCGCGGAGCCGCCCGGGCGGTGGTGGTGCGCGCGCGCAAAGACGAGTAAAACGCGCAAAAAGAGTAAAAGTCTCCGCTCCGCTCGGGGCGGACGCGGACGCGGGAGTGCGTGACGACGACGAGTCGATCGATCCAGCCGCGTCGGGCGCACGGCTGTGGCCTGTGGGCACGGAGCCACGGACGGGCTCCCTACTTTTCTCGAGTTTTTTTTTTTTGGTTGCGACTGGGAGATGTACTCATCAACATCTAAAACGGTCATTTCTACAGGAACATAATAATACAACAATAGCGCCACATCACTTGATAAGATAGGAATTTATTCTTGCGAGCCTAAAAGATTACAAAAGAAAAACTGACACTTTTTTTTGAGATTTAAACCAAAACTGAAAAAAATTGAGTGTTTTACTACTAGTCAGTTGGTTCCACGAGGACCACGGACAGCAAAACGTTTCCCCGAACCCATCCAAAAAAGGATTGGGATCCTTTACAGTTGACTGCACCGTGTCATTGTCACTAACATGTGGACCCGATGTGTCATAGGATCCACATGTCAGTGACAATGTCACGGTGCAGTCGACTGCAGAGGATCCCAATCCTCCAAAAAGACCCAAAACCCAGCAAACGGGGCTTATATAACCACACTCCCAACCCCTCTCCCCCCTC

>Os04t0463600-01 2000 bp upstream sequence

CTAGTAGTAAAACACTCAATTTTTTTCAGTTTTGGTTTAAATCTCAAAAAAAAGTGTCAGTTTTTCTTTTGTAATCTTTTAGGCTCGCAAGAATAAATTCCTATCTTATCAAGTGATGTGGCGCTATTGTTGTATTATTATGTTCCTGTAGAAATGACCGTTTTAGATGTTGATGAGTACATCTCCCAGTCGCAACCAAAAAAAAAAAACTCGAGAAAAGTAGGGAGCCCGTCCGTGGCTCCGTGCCCACAGGCCACAGCCGTGCGCCCGACGCGGCTGGATCGATCGACTCGTCGTCGTCACGCACTCCCGCGTCCGCGTCCGCCCCGAGCGGAGCGGAGACTTTTACTCTTTTTGCGCGTTTTACTCGTCTTTGCGCGCGCGCACCACCACCGCCCGGGCGGCTCCGCGCTCCCTCGCACCCGTCGATCCCTTTCCATCGTTTGCCTTTAACATGTCACTTCACCCGTTCGTTGTTCTCCTCCCGGTTTCCCGCACCACTCGCACGCCGCAAAACATCAAAACGTGCGTGAGCCAAGACGCGAATAGTAGGTAAGGACTTAATCTCAAATAATTAAAAGGGATGGGACTTCAAACTCAGATCGGCTAGTCCGTTAACTTATAAAGCTAACCGGAAGACCCCTGAGTGAGGCAATCACTGTATGCTGATGGCCCACGCACATTTTGCAGCGTGCAGCCTGACGAGCTGCACGAGAGGACGGGAGTCGGGAGATCGATGGGCGGATGGGGCAAGGCGAGGCGATGCCGGTCATGCCCCGCTGTGTACGTAAGATGGCAAGAGCAACTGGGTTTATGGGTTGGTAGGTGGCCGGTCCGCCGGCTGTGACTGGATGGAATGGATTTTGCGTGGTCTAAACGTTGGAATTCAGAAGGGATGTGGGGGGTCAAATTTTCGGCTTCAAACTTCGCACGGTGAGTGGGTTGATCAGCAGCAGCAGCTGTTCCTGCTCGTCGGAACTGAACCGTTTCAACTGTTTGATCGTACTGCGTACGTTTACCTTACCCGGAAAGAGAGAAAAATATTTTTGTAAAATTACTTTTCAATCTGGCTGGGTCGTGCTGCATCCTGTGTTCTGAGTTCTGACCACTATAGCTACTTTAACCTGCGAACTCAGAGGGTGCGTATTTGTAATTGCTTTCGATCTATTTTTTTTTGCATGTGCCGCCCGAAGCCCCGAACCGATCTCTGCCTAACTAGAAAACGACGTTCGATTCCGATATATGCTGGAGATCGATGGCGATTCTCGGGACGGGACAAGGATATATCGGAATCGCTTGAAAATGTGGTGGTACGTACGTAGCTCGCTTGAAGACTGCCAGCCAAAACAAACATTTTATGCCGCCATCGGCACAAGTACTATGCCCCAGACTCCCAGTAAGCACTGCCGTTTACCAAGTCGCGGTCGAATGCTGCTGCCACCTGCTGTCTCATGATCTTGCATCATGGGCAGATTTGGATATCCAAGATGAAACGAAACGGATGGCCATCGCCGGCATTGTAGAGATAGGGAAAGTTCCAGGAAGGCAAGGAAAAGTTGCTCTGCCTCTGCTAGTCGCCGTCACAGTCGAGCAGCCTGAAAAAAGAAAACTACCACAGAACGGCATAAACGCTATACCGTTCGAACGGAATAATTTATGTGTGCTAATGTCAGATTGTCAGCACAGTACCTACGGTATCAGATATACAAGACAGCTATGTAATCTGGTACCACGGTACCAGAAACAATACACATATTTATCATGCTGATAATTGGAACGCTATATTGTTTTGTAATAGTATTTCTGAAAAAGATAAACGCCGTTTCATGAGAAAAAAAAAAAGAAGTGTCGAGGCTGATGGGCTCTGCCATGGATGAGAGGCCCAAGCCCAAAGAGTTCGGGCCTGACCATGTCGTCGTGTGAGCGTGATCGATCGTGTCGCCACCCCCCCTCGCCACCACGGGAAAAAAATCCGCGGTCCCAAAATTCCCCAACCCGTC

>Os04t0474800-02 2000 bp upstream sequence

GCAGCAGGGGCAATGCCCGGTGGCCTTCTCCTCACGTTCCTCCTCCTTGCTGTCGTCGCTTCCGGCGCCTACAATGGCGCCGGCGAGCCGCCGGTCAGCCGGAGAAGCTTCCCCAAGGGGTTCATCTTCGGGACAGCCTCGTCGTCGTATCAGGTTAAGCTTCCACTTCCCTTCCACTCCTACAGCAAAGAAAGCTTCCAGTTCCATGCATATGTAACTGTGCAGAAGAAATTCAGTTCTCTCATGAACAAAAAGGAAAAGGAGCAAAACTACAAACAGATTTGTGCAACTTTTCTCCACCGAGAATACGTTGGTACAGATTCACACAGAAAGGCAATGCTACTGGGTAATGCTCAAATTGGCACCAAGGAATGAATCGGTTGCTAAAATTTTAACTGTCACACTGAGACATGTTTTTGTCTTGGTCTGAAATCTGAGGAATTGGCTGTTCTTGAGATGCAGTACGAGGGTGGCGCAGCGGAGGGCGGCAGAGGACCAAGCATCTGGGACACCTTCACACATCAGCACCCAGGCATGTTAATTGCATCTCTGCCTGCAAGCTGTGCTACGTACAGATTTGTTTTCTTTTCTTTTTTAGGATAACAAGTGCACATTCCCTAATTAAAAAAAATTGCATTCCCTAAAAGATAACAAGTGCAGACTTGTGTTAATCAACTTTAATTATGTGCATTCTATATACTCCATCCGTTTTTTAATAGATGACGCCGTTGACTTTTTCTCACATGGACCATTCGTCTTATTCAAAAATTTTATACAAATGTATAAGATATAAATCACATTTAAAGTACTATAAGTGATAAAATAACTCATAATAAAATAAATTATAATTACGTAAATTTTTTTAATAAGACAAATGGTCAAATATGTGAGAAAAAATCAACGGTGTCATCTATTAAAAAACGGAGGGAGTATATCTCAACAATAATGAAATCTGACCATATTTAGCAATCCCAGCCAGTAGAAGTTGCATGCATACACTACACATATGGTACCATAGCAATATCTGAAAAACAAATTACGCGCGATTATGTTCTGAGTATATTTTTTCCTGTTTGGTGGATTATCTATGCAACTAGACCATCCTATCTTTGCGAATGGTCCAATATTTTGCTGTGAGCAAGATTTTTGCTCTTCCTACGATAGCTCAGCGGGTATCTTCAATCTTGACCTAGTAATCCAATCACGTCGCTTTTCAAAAAATCATCATCACAATTCAGACGATTGGTCTAAACAGTCACCATCTTCTCGAATTGCTTTTCGTCCAAATATTATTTCCTGCGCGTGGTTTACAAAAGAGGCCGTTGATATTCTTGTCCTACCTGGGATGTAATGTACTGAATAGTCTAAAGGGAAATGTAGAAATCAGGGGTAACTTAAGCACATGATTGATGTGAAATGATAAGAAATCAATGGACAAGGACAACATTTTTGGATTGACGATTATCTGATTTGGCAGAGAAAATCGCCGACAGAAGCAACGGGGATGTGGCTTCGGATTCCTACCATCTCTACAAGGTATATTAGCAGTTAGTACATTTTTCAAGTAGCAAACATGCATTTCTAATTGATTTTTCGTAATGATTGTAACTAAAAGTATACTCTGAGTCTTTAGTCAAAAAAAAAAAAGTATACTCTGAGTCGTGTTAGATTGTTCTACTACAGCTAGGAGATATTGCCTATCACAATCTCTTCAAATTCTATTCTAAACTTCTTTTAAAAAAACAATTTAGTACTTATCCAGAGGTACTTGTGAAAAATAATGCAGGAAGATGTGCGCCTCATGAAGGATATGGGAATGGATGCATACAGGTTCTCCATCTCATGGACAAGAATCCTTCCAAGTGAGTGGCCATATCTTCCGACCTTCTACTTATTTCAAACCTGATACAATGCACTATCACAGAACTATCAGCAGACAGCTCTGGCTTATCTGAAGCTGATCAGCCTTTTTGTTCTGAAGTAGTACAATAATTTGCACA

>Os04t0560700-01 2000 bp upstream sequence

GGCGGGGCCCCGTGCCTCGGGAAGCTCGGCCGCCGCCACGTCCCCGGCTCATCGCCGCCACGCGCCTTGCTGCGCTCCGCGGTCGACGCCGGCCGCCGCATCGTGCCCGACGTGATGGGTATCTCGTACGTCTTGGTGAAGCAGTTGCCCATTGCAAAGCCTCCTCGTTTCCTTTCCTTCTCCTGGGGTGGCGCCGAGACGAAGCGAGGGTGGAATGGTGAGTTGGGTTTGGGAGAACGAGGAGCCGAGGTTTTGAAGGGTTTCTCTTTCTCTGCGCGGGCGGTGGGCGGCGGGAGCGGAGTTGCTTGACGTGATGCCCTACCCCTTCGTTTCGCTGTTTGCTTTTGGTCCGGGCTTTGAGCTTTATGCCTTGTTTGGTTATCTCCGGAGGGAAAGGAAGATTCAAAGAAAGCCTCGGGATCACGCTATCTCACCTTATGCAGGCAGTACAATATACTTGCGGCGGTGCGACAGCCGATTTTATTCGGCTTTGGAGGTGTAGAAACACATAGCACTACGTGCTTTTATTGATCTAGCAATAGCCATTTTACTTGAGGGATATGCTGTACTAGCTTTCGGTAACATCGTCAGAATCAGGCGCAGGCAGCACTAACAAAGTACGCCCCTTTTCAGAACGGAATCAACAGTGGGCCGGAGTAATGTGACGAGCCAAGCCGGCCCAGAAGATATTCCGGCTTACAAAACTCAGAGCGGAATCAACTATGGGCTGGAGGTAATGACCTGGTAAGTCTATCTTGACTCAAAAAGACGTTTTATTAACATGGTGGCATGTGCTTTCACGGCTTCTGGCCTCAGCAAGCGGTGACGCGCGCTCTGGCAATCGCTAACACCCGGAGCCGCCTCGCCCGCCGCCCGCCGGTGGCGCCCCGCGTCGATCGGCTAGGGGCGAGGGGCTCAGCTGATGTCTACGCTACGCGAGAGAGCGGGCGGATGCCACCTCTTCCACTACTAATCTCTTTCAGCGGCGCGTGGCCTGAGCAGGGAGAGTAGCAGACGCAGCAGCGCCAACCTGCCCCGTGACGGGCACCGCGCACCATTTCGGACCGATATGCCGTGACGGGACGCCGCATTGCGCTTCTGATAAAGCATCCGGAGAGTCAAACTCGATTCGGAAGTTCAGTGTACTTCGTCGCCCACACGGATGGGTTGGGGGCATGAATCCGAAGTGAGATCATTTCCATGGGAAAAGAGGCCAAAAAGATAAACTAAGGAAATAGTCCATTCATGTGTTTCTCTCCAATATACGTCATAAAACCTTTTTTTCTTCTCTTTACGGATATCAATATACAAAAGTTCCTTTGAAGGCTAGCATATGTGTGGTGTTTGCAGTCCAAGGTAAGAAAGTACAGTAATAAGTACCCTTGGTGACCGACCGGTACAGTTCTTCAGTTATCAATTCTTCTCAGATTAAACAGCTGTGGTTGTATATAACAAGTAAACTGGTTTTCAGAATTGCAGCACTAGAGTGGTGTGAAGGCCTGAATGGGTTCATCATGATTCTTGATGCAAACTCAGTCTAGGTCTTTCTTTGTCCTTAATTAATATATATTTGTGAAGACCTAACTTCGAGTCCAGAAAACAAAAGGAGCTTTTCGTTGTTTCTTTTGAAGCCTAGTAACAAAAACGAAGGAACACTGTGTTAAACCAAAACTAAAAAGGAAGAATATGTGAACCTTTTGTATGCCGGTAAGGCCGTATGGAATTTCAACTGCACTCGTTTTCAAATTAATAGATGTGGTTGACTAAGTAGAAAAAAGAAATACTAGTATGCTGATGCAAGCTAGTGTGATCCAGGGGTTCGTTTTGTTTCTTGACACACGCTTGAGTCTTATTCTTTATTACCCCAAAGCACCTTCCTAATCCTACCTATTACTTTGGTTAACTTTAGAAGACACTGACTCTACCAACTAACATGTAAGCAAACACTAGTTGCATAACCGTTGGTTGAGAAGAGTAGACGAGAAGGGACCCAGTCTAGT

>Os04t0564700-02 2000 bp upstream sequence

TGGATGTGTTTTTTTTCTTTTTTTTTTATCGTCTGACGGTGACTGGGGCATGTGCGTGGCCGGTTGAGCTAATGCTGGAGTGCTGGACCAGGACATCCAATGCAAAACCTTTGCGTGCTCGCGACACGTACTCAGTACTACATGATCTGAATATCTGATCATACTACTTTGTACTATGATCTCAAATATTTTCCTCATTTAATCTTGGGAAATTCAAACGCGCGTGGTATATGGGGCTTCCACTTTCCCTCGTTTGGACTTTTGAAAGTTTCGTGTAAATTTTCACCCCATTCCTGGTTGGGCTTTAGGTTGTTTACTTGTTTATGAAAAAAAAAACTTGAAAATTTCGAATGTTTTTCTTCCCGACTTATGAATGATCGTCTTAAGAAAAAAACTTAAAATTCGTAAGAGCTCCCGGCGAGGATCGAACTCGCGACCTTTCGCTTACGAAGCGAACGCACTACCACTATGCTACGGAAGCAACTGATACATTACGGTCACGTTTGCATTTAACTACCGCTACTACTACGCAGCATCGCATCGGAGGTATCAGCGGCCTCCGCGCCGAGGACGTGAGCCCGCGGCTGGCGTTCCACTACGGCGTCCCCGCCGACGCCGCGCTCCTCGCCTACGACCCCGCCCTCCACGTCCTCGCCGTCGCCATCAGGTAGCCGCCCCCCCCCCCCCCCTCCTTCCACCCGCTCTCTCGCTGACGCGCTCTTCTGCTGCATTTTCTCTGCAGGAATGGGCAGATCAAGCTGTTCGGCCGCGACAACACGCAGGCGCTGCTCCAATCCCCGTCCCCCGTCCCCTCCAAGTTCCTGCGGGTATCGTCGCTGATTCTTTTTTACTCGAAATCCCATTGATCTTTGTTTCGGTTGCCGCGTTTGCAATTCTTGGGTGTGGTTCAACTTCAAGCTCAGTTAGTTCGCACTTCCGTTTCAGTTTGCCGAGGGGCAAAGGGTTCTCCTGAACGTGAACGCCAAGAACCAGATCGAGGCAAGTTTGTTCTCGGACCTTTCAGTGCTCGTTTGGTTAAATGTGGTTTCCTTTTTGGTGTGCTGCTAATGGAAAATCATCTTATGCAGATTTGGGACATCGACACAAAGAAGTTGTGTTATGTGCATCCGTTCGAGAAGGAAATCACGGCATTTTCCGTTCTGCAGAATAGCTTCTACATGTAAGCATCGGTTGCTTTACCTACTGCTGAGTCCTGTGGAGTTAAGTGCTCTGATTAGAAGATCTTGTCTATGCAGCTATGTGGGTGATAGTTTTGGGAATGTGTCCCTGTTGAAGCTTGACTTGGGTCAGACATGTCTAGTTGACATGCCATATTGGATTCCTTTCTCTGAATCTTATGGTAGGTTTACTCCAATATGTATTGGTTGAATTATCATACTTTACATGAAAGATAAAAATAGCACCAACTTTGGAATCTAATTGATTCTTGCTGAAGGTTCTGGAGCAAATGTTGGTAATGAGGTTGAAGTTGCATTTGTATCGCCACAGCCTTTGGCTGAAAATAACAGGTTGGTGTCTCTTCTGAATATTCTGTACATCTCTTCCAAACTAGTTTGGCACAAGCAGTTAAGTATTTTATGCCAGATAGTACTCAGGTGATCTTTTCATCCATTCTATGTATAGACGTAGTGAAATGTCTAATTAAGGTACACTCTTCAGAGTGCTCATTATCTTCAGAGATGGGATTATGTCTTTGTGGGATATTAAGACGAGCAAAGTGGTATCTTTATCCGGCAAATCTATGCAACAACAGTCACATCAGGAGGCTAAAGCTGTAACATCGGCATGCTGGGCTTGTGCCAAGGGAAGCAAAATTGCTATTGGATTTGATAGTGGTGATATATATCTTTGGTCTATTCCTGATATTTTAAATGCACAAAATCTCTCATCGATGGGCAATCAAAACCTACCTCTTCAGAGGCTTAACCTTGGATACAAGCTAGACAGGGTGTCTATAGTCTCTTTGCGATGGGTCAATA

>Os04t0665600-01 2000 bp upstream sequence

TTAAAATCTGCATTGATAAAGGGGGCCTAAAATGACTGACGATGCAGATGTGTATTGCAACTTAGAAGGAGTGAGATGATATGGACCTAGAAGTCATCACTTAGATTTGAATTTACGGATGCTAGTCTTTTTTAAAGATGACAGGATCAAACGAGAAAGAAAATGGTGCCTTATACGACAATTGAATTAGATGAAACAAAAAAGCATCGATGTTATCGTGTTAACATGTGATGACTTCCTATGGCATAGTGTTGGTCCTATGAAGTGCAAGCTTTTCCTTTAGTTTGCACTTCGGTGACGATATTGGATCACCGATCAAATGTGCCGTCACGGGATGGATAGTCACTCCATGTGCCCTATGTGTGCACAGGAGCAGAAAACAACCAATCATATCCTGTTGGAGTGCGTCTTTACAAAGTAAGTTTTGCACAAGTTACTGGCCAAGGTCGGACTGCCCTTATCTTCGCCCAGTCGACAGTCCACCCCCCAAGTCCGGTACTCTGGTGGGAGTGCACTCACCGGTAGCTGCCTGATCACCTTAGGAGAGGATTCGACTCCTTCATCCTCTTTGTTACCTAGAATATCATGTTGGAAAGGAATGCTCGTGTCTTCGATGGGTTCATCTCCCAGGTGGATAGAGTGGTTGATCGCATCGTGCAAGAATAAAGGATCTGGGTGGAAGCGAAGCCGAGTCCTCTTGGAGTTCTTCTTGTCCATAGTCCTTTTTCCTGCCTTTTTGCTTGTTCGGGGCTGGTTTTGCCTAGCTTAACTGGTCATTGATCCCCTTTTCTCCCCGTTTAAACTCCCCATGACCGCTACGGTAGGTCACGGTTGTACAAACTTTTTCCTTGAGGAGGTACCATGAGGTACTATTTTTTCTATTATAAATTTGGTACCTCTTGGTACCTATGTACTACGAGGTATCATGAGGTACTAAAATTTTGGTACCTCGTGGTACCTCCTCAAGGACCGTAGAATTGCTCTTTCTAATATATTAGCGTACAAGGCTCTTGTGCGTTCACAAAAAAAAAAGATCAGTTTATTTTGCTGCTGATGAAGTTACTGCTCTATTTTGACTACTTTGTCTACCTCATGGTGACACAGAGAGTTGTTTACTTGATTATCTATGGATCTAAAATTTACCCGCTTCGTTTGGCCAATAAACATCACGTCCAAGAAGATTCAAACTAGAAACATTGTATGATTCGTCAGGTTATCTACCCTTGTTGGGACATCAAAAGACAAGCAACACACAAATTATGGAGCATAGAGATGTCCCCTCTGATCCTAACTGTAATATGTTTGAAGCTTGCAAATTATGTTCAGCTAGAGCTGGAGACTAAAAAGAACCAGATAAGCAAAGAAAATGATAGCGAGGGTGAGGGCACCAAAGTGGCTATTTGTCACCTTAGCCTTGTCCGACATGCGAGATAATTTATGCTCTGCTATATATATGTAGCAGTTGGATTTATATACATACACATATATACATCTCTTTGCATATTTATATTCTTGTCAGTTGTTTGCTGAGGCCCCCACTGTGAGAAGTTCATATTCCATTTGGGATTATCAGGACTCCTTCAGATTATTCCAATCCTTCCCTGCATACAGAAAGTGATCTAGCTGAGGTGAGATCAGAGAGAGAGAGAGATTGAGCTGGTTTTACCAGGAAGTTTCCAGTGGTGTTTGCTTTGCTGCACATGTGATGCCCTTCAAGTTGTAAGTTCATCTCAACCATGTGTGTAATAATGCTTACTTTTTGCCATGCATTGCATTGCGTCCTCTCCTTCTCCGGCGGCTGCTGCTGCTGCATGGTGTGATGGTGGTGGTGGTGGTCGTCGTCGTCGGCTGTTTTGCGATTCTGGAGATTGGTTTTGTACTTTTGTGCGTATCCGGGGACATGCACCGCCGTCGAATTATTCTCGCCGTTGTTGCGTGCCACGCCTCCCCTCCTTCCCTCCCTGCGAATATAGGCCGGTGCGGATTCCCAATTAATTCCC

>Os04t0671200-02 2000 bp upstream sequence

AAAAACGGACTCAATACTACCTCCTTTTTAAATAGATGACGTCGTTGAGTTTTGAATACACGTTTGACTAATCGTATTATTAAAAAATACGTAATTATCATTTACCATATTTTTTAGTTGTTTTATCATTAAAATTACTTAAAGCATTATTTATATGTTACGTATTTGCACAATTTTTTAACAAGACGATACTCAAACGTAACGTTATCTATTAAAATACAAAGGGAGTATATATATTATCTGTGTTCTAGTAGTTCCTCTTGTAAAAGGAAGAAAAGATAAAAACAAACTGGTGCCGACGCCAACTATTTCCACCGCATTTGGGCCCACATGTCCGGGAGCCAAACGCGGCTACGCGACCCAAACGCAAACGCAAAGGCGGAAGCTGGGATCCCGTGTCGTCGCCGACTCCGACTCCTCCTCCTCCGCTCCATATAAGACTCGCCCCAACCCCCTCATCAACTTCATACCAAACCGAACCGAAGGACCCCACCCAAAATCACACGCGCCAACCAGATCAAACCCACCTCGCAGGAGAAGAGAAGCGATCTACCTCTCCTCGAGGTGGTGGTGAGGATTTGGAGTTGAATTGTGGGGAGGGGGACCTCGCGGGTGTTGCCGGTTTGGAGCCCAAGATAGGGTTATGAGCTGCTTCGCGGACCGCCGCTGCTACGCCGCCAGCCGCCGCCAGGACGGCCTCCTCGTGTTCATGTTCTTCATCTCGATTCCCATACGTACTAGTAGCAGTAGTAGTAATAATTGTAATTCCAGTAGTAGTATTAGTAGTAATCTCCTGTAAGCGGAGTTCCTCCTCGCGGCTGCGTGTACGGGGACGGGAGGACCTTCTTCCTCTTCGTGTTCATCTTCTTCTTCTTCTTCCTCTCTTCCTTCGGCTTCGGCGCTGGCGGCTATGGATCCCAATAGCCTCAAAACTGGAGGTATGAATTATTCCCACTTTTTTGTAATTTTTTTTTCTGAAATTTTATCTGGTAGGAGTACTTGGTAGTGGTGCGTCGTGAAAGGTGGGAATATTTCCCATTGATCATAGTATTTTTAGAAATCTATCTATTGTACTTTAGAATTAAATTTTCCACATAGGGAAGGTGATCATCACACTTTAGATTTTACAAGATTATTAGAATTTATAATTCTAGTTGGGTACAAATTGGTTAGTTAGTTGGTAACTTGGGTATCTACAGTATTATGTAGATTAGTGAGCTTGATGTTCAACGTGATTTCTCTCTCTTATGATTCACATACCTGACAGTAAAAAACATTTTTTGTTCAGGCCTCTTGCTTCCGACCATTGAGAGGCAATGTGCTTCGCCTCCATCCGTCATCGTGATCGGTGGGGGAATTTCAGGGGTTGCAGCAGCCCGTGCTCTCTCCAATGCTTCGTTTGAGGTGACTGTTTTGGAGTCCAGAGATCGCGTAGGCGGTCGTGTCCATACCGATTACTCTTTCGGATGCCCAATTGATATGGGAGCCTCATGGTCAGTCTCACCCTGAATTGTTAGTGTGAGAAATTCACTTTAGATTCTGTTCATACAAGCTTACTTACTGTTCATTCCAAAAATTCAGGCTCCACGGCGTTTGTAATGAGAACTCCTTGGCACCATTGATTGGCTATCTTGGGCTGAAATTATATCGCACTAGTGGCGACAACTCTGTTCTGTATGATCATGATTTAGAAAGGTAATCTTTTAGCTCTTGATCTTACGCTTTTCTTTACCGATCCGCACAATCCTTGTTGATTCTTACATCAACTCTGTCAATTGATTCTGTTACAGTTATGCACTCTTTGATAAGGCTGGCCATCAGGTCTCAAAGGAGACAGTTGCTAAAGTTGAAGAAACATTTGAAAGAATTCTTGATGAGGTACATAATGAACTCATTTATACTTGGTACTTCTGGTGCCGTTTGAAGAGCATGTTCAGTTCACTATCTAATTTCTGTTGCATTAATGTTCCAAAAAAAATTTTCTGTTGCATTGCAGACAG

>Os04t0679400-03 2000 bp upstream sequence

TTGTCGTCGCTCGGTGTGGGCCCCACCCTGTCAGTGTACGGCTCGGCGCTCACCGGCGTTTACCTCCCCAGCCCACGGGAAGGAGGCGGCGCGCACGTGGCAGTTGCGGTGGGTGAGGCTGTGAGGGCGGTGCGCGAGCCTACGTGGCACGCCGCGTCCACGGAGGCCGTAGAGAGGTGGCCCCCGTGAGCACGCAGTTCGAGTAGGCGATGACGTGTCCGACAGGTGGTTCAGCGAGCAATAATTACGGGGTAATATAATAAACCGCTCCCGCCAAACTAACGGCGGCAGCCCACGGAAAAGGCGTCCCTCGCTGCTGTTTCCTCCGTTTTTCGCTCACCTCCGGTCATCCTACTCGGCTACCCCGGCGGCAAAAACATGAAGTTCTACAGATCTACGACATCAGTTTACTAGTTTGTCTTTTGTTCTACTTTGCTCTTTCTTTCTTGCGAATTTCTTTTTAATCACGTTTTGTGTAGACCATGTACAGCATCCTCACTTGTGCAGGGTAGGAATTTCACAAGAAAACAGTGCTCGTGCAGCGACTCGATAGAAACGGAAGAAAACCGTATCAACAACCAACACGAGGAGGTGTGTAATACTCCTTTCGCCTTGTAATATAAAAGATTTATATAGATGGATAAATTTTAATACTATAAATTTAAACAAATTTTCTATGTAGATTAGTAGTATGTGTTATATCTATTTAATTTTTTTTATATTTTAAAACGGAAGTAGTAAACAATTACTTATGTAGGCTTTGGGTGCAGGGTACCGTAGGGGGTGAGGGCACCGCACGTCACGGTTAAGGTGGCCGGTTACTACTACTGGTGTCACATCGGCAGCGGCAACTGGTATTACGTGATGATCTTATCCTCGTCGTCTCGCCGTCTTTATCCCGTTCGCTGCTTGCTTTGGCTTCTCACATCAGCACTGACTCCAATAACACGCTCATCAAACAGCGCGCGCGAGCGAGGGATGGATAGGATATTTTCGTTCGCGTTCGTGCCATCCTAAATCCCGTCTATAAAGCCGTGCACATCTCTATCCTCACCTCCATTCCATTCCACTCGCCAACCCGACACGTCTCTCATCTTCACGCAACGAACAGCAGCAGCAGCTCAAGCTAAGCTAAGCTAAAGTACTACGTGTTCTTTCGTCTTCGTCGGCGCCGGAGTTTGATTCGTCGGAGCTAGCTAGCAAGAGGAAGAAGAAAATGTTGGCCATATTCCAGAAGCAGGTGGCGCACGCGCCGGCGGAGCTGAACAGCCCGCGGAGCAGCGCGGCGAAGCCCAAGAACCCCGACGAGATCCTCAGGGACTTCCACGCCCTCCACCCCATCGAGGCCTTCTCCACCTCCTTCGGCGGCGGCGCCGCCCTCGCCTGCGTCGCCGGCCACGCCCGCAATGGCCTCTCTGGCTACGAGAGGTAAATTAAATCAATTTAATCGATGAGGCAATGAATCCGAATAATTAAGGTGATTAAAACGTAGGGGATCGATAATAATGGTAAATGGGTGTGTACACAGGATGTTCTGTGGGCTGGATGACATCTACTGCGTGTTCATGGGGAGGCTGGACAACCTGAGCAGCCTGATCCGGCAGTACGGGCTGTGCAGCCGCTCCACCAACGAGGCGCTGCTCGTCATCGAGGCCTACCGCACCCTCCGCGACCGCGGCCCCTACCCCGCCGACCAGGTCGTCAAGGATCTCTCCGGCTCCTTCGCCTTCGTCGTCTTCGACAACAAGTCCGGCGCCGTCTTCGCCGCCCTGGTAATTTAAACCACTACTAGACTGGTTTTCTGAATCGAATTGATGAATTTGTGCTTCAAATTGAACTTCCTGTGAGTGTAGCGTGGCCCTGTCCCCTTCCCGCTGTTAGGAGTTGTGTTTAGCCGCCGTAGTAGTTCCTAAGCTTCAGTTTCTGATTGTTCTGCTTTTCTTTTTCTTATATATTGACGTTCATTCGCTTTGCGCGATGCAGAGTACGGATGGGGAGGT

>Os04t0686000-01 2000 bp upstream sequence

CGGGCGGTTTGACAGAGACAGCGACCTCACGCAGCATTTCGTCTCATTCTCGATCGCAAGTCAAATTCACAGGATTAATCAACCAAAACTAGCAACTTAGCGACCATTAGTTAAGTGATTAGCCGCAGATTAATTGCAAATCCAGCACTGTACACCTCAGACCGAGGCCTACCGTTTACCAAAGTTTTGGCCTTTCTCATGTCCTCATCACTAGTCTTTACCCTGGATTTCTCTTTGGAATGCATCATGTCCTGTTCATTTGACGCTATTCTTTCTCTGAATTTTGCTCCTAACGTTTCAGCTAGCCGCATACATGGACGTAAATTAAAGTATCGCATTCAATTCAGATGAAAATGAATAACAACTTAACAAGAGTCTGATTATTCACTAAAAACAGTGTGCTGAAGGCCTAAAACATCGCCTTAATCAAGCGTTACAGATGACAAGCCGCCATCATCCGTATCGAATAATTATAATTATATTCCATAAACTGCCCCGTGATAATGAAATGATCTATAGTTTGGGTCAGATCAGACAGATCTGGAGTCGGTAGAGGAGATTATAATCTATCAGTTCAGTTCACCTAGAGCAAGAAAACAATGAGCACACTGTATCAGAAAAAAGAAGAAACAACAAACAAATGCTTAGTCTTTTACTTGCCACATGCAAACCTTCCTTAATTCCAAGTCCAACTGTACTTGTAAACAATTGGAGGGATGAGCTGCATAATTAACAAGTGTGGTCAAAGATGCCCTGCTGGTCATTAGAGGTCGCCTAGGCAAATTTCTCGTTTATATCTGCCTAGTAATTCACCTAGACAAATCTTACGTCTCCAACAAGAGGTGCAGGCCAAAACATTGAGAAATCCACTGTAAATTAAGCAAATTAAGTAATAGAGGAGCAGCGGCCTCCTAGGCGTTGGAGACCTCACGTCACTGGCCATTTTTCTCTCGATAATCCAATTGTTTTATCTCACCTCGTCAACTCTTTTCTGCTTCATCTCACTCGCTTCACCTCTAGCATCGCGCGCGCACGAACTGAAACAAACTTATCTTATAATTTGGTGCAAGCTAGTTTTTTTTTAATTTAAAAGGGTATCATGCAATGCTCGTGGCCTATTATGTGCAGTACTATAATCTTGATATTATTCACGAATGGAGATCTTCATCTCATTTACTACAAAAGCACTATCTGCTTAGGCAAATCACTTGGATCGAAGATAATGTTTGATTTTTTTTCTTGTTTTCTGAAGATTTTATTTACAAAATCAACCAACAAAATATTTTCAAGTTACTATAAATATTTTTGACAATGATATGATAACTCCAATATATGTCTATGGCAATACGATACTTCGTTATGGCATGGCTAGTCAACAACAGTGGCCTGGACAAAGGATTTATTAGTATTGAAAATGAGGCATGAGGAGTGTTTTTTTTTTACAAAAACAAGAAAAAGATTCTGAAAATAAAAAAAAATCAGTTAGTGCTATTACTCCCTCCGGTTTCATTTTAATTGACGCTTTGGACAATGACACGTTCTACAAGATATACATTTGAGCTTATTTTTTATTATAATATATACAATAAATAAATGCATGTTTATTTTTATTATAGTGTTTTGAAAGACAAATCTATATATGTTTTTCTAGTTTCTTTAAACTAAATATTTTTAAAGTTATTGATGGTTAAAGTTATAAAAGTTTAACCTCAATCTTATCCAAAACGATAATTAGTATAGAACCAGAGGGAGGGAGTAAAGAAAAAGTTTGGGGCGGTGACAAGAGAGAATCAAGTTGTAAGATTAATCAACTGAGGCGTCACCTTCATGGAAGGCTAGCAACAACCAAAGAGCTAGCTAGCTACCTTGACCGACCGAATTCTTTAATATTCGGGCAGCTCATGCTCGTGCTCTCGCTCATGCGTGTGGACTTTGAATCCATCCACCTCATCCTCCTCCCGCGTACTCCATAAAACTACTCGGATTCGATCTCTTCCTCC

>Os04t0691366-00 2000 bp upstream sequence

ATCGCATAAATTAATATTCAGACCGGCCGGGTAGTGACGAACGAAAGACCCAAATACTGTAAGAGATAACATGTTTGTATTTGTACTGATATGTATATGGAGGAGGACTACATGCACATATTTGCACTGATAACATTTTGTGCTAATATATAGTATAGGTAGCAGGTCGACGGCCAGCAGTAATTAATTAACGTACGGTGTCTATAAATACTACTATATACGATGATACAGAGTATGGATTCGAATTAATACGTGGTAGTCATTCACGCATGATGGATGCATGATGATCCGATTACTAGTAAATTTCTCATTAATATAATTAATCGACTGAATTAGCTAACAAGTGAAGTGGTGGTGGTGGAGCAAGGTGTCGACGATGATCAGCAGCAGCAGCAGCAGCAGGCGCGGTAGTAGCGTCGTTAATAATGTCGCCGCCGGTGCCTTGTGTGTGTACGGCAGTCGGACTCACCACCAGCGGAATATTATCATTGCTTAAGATCATCATCTTGGATTCATCCTCCTCGTCCTCCAACTCCATGTCCAAGGCAGGGGAGCAGAGCAAGCCGCCGCCACCAGCAGCAGCAGCAGGAGGAGGATATAAATATTGGTCGTCGTAGTAGCCGGGATGATGAAGTAGAGGAGCCGGCCTGTGCAGGTGAAGCTGATGCTGCAGGGTGGTGGTGGTGGGCTTGTGAAACGCGCGGCACACCACCCAGCAGCTGCCGTCCGGCTGCTGATGGTGGGCGACGAGGCGGTACTCGTGGATGATCCAGTCGGTCTTGCAGCCGTTGGGCGCGCGGCCGCGGTAGAAGACGAGCGTCTTCCTCATGCCGATCACCGCCGGCGACGATGATGATCGAGAGGAGGAGAGCACCGGCTTGTCACGGCCCGTGGCCTTCCAGAAGCCCGCCGCCGTGGCGCGGTTGGTCCGCGTCCCCGTCCCTCGCGCGCTGGGGTACTTGCGGTCCTTGTAGCTGAAGAAGTAGCAGTAGTACTCCTGATCATGCTGCTGAGCTTGCAGCAGGTCCCATGGCTCGATGCTGTTAAGATCCACCTCCTGGATGATCCCGTCGTCCTGCTGCCCGACCACCTTCCGTGCCAGGTAGTAACCAACCAGTTCCTCCTCCGTCGGGTGGAACCTGAATCCCGGCGCCACACAACACCACTCCTCCTCCATCCTTCACTGTTCACTGGGATATTATATATATGCCACAACCCACAATAAGAAGAAGTAGGAAGTAGAAGAAGATGATACCAGCTTGCTAGTTAGCTACCTATGCATGGTATGGGTATTTATACTGGAATTAATCTTTGTTGGGAGCTGACGACATTGACATTTATACTGAAATTAATTAATCTTTCGGAGCCTGTCCATATATTCTGCTTTCGGTAAAGGAGATGACAGAAATAGACCATTCTTTTAAAAAAAAGTTTTAAATAAGATGGACGATTAAAGTTAAACACGGAAAATCATAGTTAAAATGGGATGGATGAAGCAATTGATTCTTCGTGAAACGGAAGGAAGGTATAGAGAGTTAGTGACAGACTGTGTAGTCCGAGGTATCTAGTCTGTGGGCCTTTCCATCGTCCGATGGGATTATTATTGGGCTGAAGGCCTAAAGTTACCATATGATATGGGATGGGCGCGGGCCCAATTAATAGCAGGTCTTGTCGTCTGGTATGCCTAATTTGTGGTGGATCGTGGACTCGTTCGTTGGTCTGAAATATCTCCATCCGAATCATCACCATCAGAAGAGGATTGGAAAAGGGAAGGATCCTGGATGGTTTGATTTGGCCATGTGCAACTGGAGCAGGAGCAGGAGCAGGAGCAGAAGCAAGCCGCATTAATTAATTAATCCTCTGAGCCTTGTCAATTCAATTGGCCGATTTGTAGTTGCCACCATCATCCTACCTACGAGCAGAGCAGAGCGCACCGATTCAGCGAGGAAAGGAAAGGAAGAGAAAAGCAAAGCGATCGATCGAGGAGGAATCCGAATACG

>Os05t0108800-01 2000 bp upstream sequence

TTGGCTGTGTGAAGGAAGTGGCTCCTCGGTGGCTTCGCTGATGTCCATTGTTGGAGGTCGGAGCTGCTTTTCGCTTTGGCAACCTTCGTGCCTTCAATGTCGCTGCGGTTGTGAAGTCGGCGCTGCTGGGTCGCTTGGGCGGCTAGCTCGGCAACGATAACACGTCTTCTACGATTTGGGAGCTGTTGTGTCGCTTTTGTGTTTAGCCTAGCAGCGACGTCTTGCGTTTGGGCTCCGCTGTCGTTGTTAGATCTGTAGCGGTGGGTTTTAGCCGGTTTTCCTTTAATTAACCGTGCAATTGTACGGTTTTTGGGTCCGGTTTTTCTTATAAACTGGGTCAATTCTCTTCTTCTAATTAGAAACGTGGAACAAAGTTCCGTCCTAGGTTTCAAAAAAAAATATTAAGGGTAAAAGCCATGTGAAATGTTAGAAATATTAGCTGAAAACGTCGACCATATACTCCTAATTGACTGAGGCAGTAGAGGCTAGACATAAACAATTTGCAGAGGTTGGTTTGCCTTTGACCACAGCACTCAAATCCGGGGGCGCCACCGGGAAAATAAAATTCAGCCACAAATTTCTTCTAATTTTTATTTTGAAATTGTTTCAGTTATCCTCTCCCTGTCAAGATTGTCCAGCTAACCTTCCAAATTATGATCTACTCACTCCAGAAGCTTCTGTTGTCCTGTTAACTGATAGCATCTTCTCTTCTAATGTTCTGTGGACTGTGTTTAATTTAGCACTAGCATTTAATTTGGGGCCTACTTGAATATCATTATATTATATTGCTAAAATTTGATGGTTTGCCATTTAATTTGTTTGTAAGTTCACTGAATCATGTGTAATCTAGATAGTACTAAGTTAGTGGTAGGTGTGTTAGCTCCCTCCAACTGAAGAAGATGAGACATGCAAAGCACCCTGCGTCATGTACAACCCACAAAGTTCTAATTAACGTTCTTAACTATATATAAAATATCATAACAATAACTCCTTAATGTGAAAGGTAAATAGCCTCTTTGAGTAATCCACATGAAGATGTGGATCTGAAAGACAAATGAGGATGTAGATCTGAAAACAATTGTAAATAAATTCATAGGATAAGAAAAAGACAATGCTTGATTTGATACTGCTATGGTCACTAGTCATAAGAGAAAATGACTTCAAAGTTGGAATATGTTTTGGATTGTCAATAGGTGCTTCAATTGGACTCTCAGCTGAGCCACCAACCTTACAACTTTTGAGCGGCTAACATAATATTCCTTTCTCCAAATAAGCAAGAATAAAAACCAGAGTTCAAATACAATTAGAAAAGATTAACATTTACTTGCTCGGTTTTACGATTCCAAAAAGAAAAAAAATTCTACTATGTTTTCTCGCATGGTTATTACGACAACGTCTACCACATCATGCATGCTTTTTTTTCATTAACGGTTTGGGTGGGACAAATATACTCCTTCCGTCCTCAAATATAAGAGATTTTGATATTTTCCTTGTACTGTTTCACCATTCGTCTTATTTAAAATTTTTTAAAATTATTATTTATTTTATTTGTGACTTACTTTATTATCCAAAATACTTAAACCACAACTTTTCGTTTTATATTTGTACAATTTTTTTAGAATAAGATGAGTGGTCAAACGTTATAAGAAAATAGTGAATATTCCTTATATTAGGGGACGGAGCTAGTAGCATATATTCGATTAAGGATTTTAAGCAGTGACAGTGATTATAGACCAATCCCTTTGGAATTATGATAATAGAAATTGAATTGAAAAGGGAAAAGGTGAAGGAAAAGAGGTGTGGGTTTAGCCGTTTATTGAAAGGTGAGATGGGGGTAGGTAAGCTAATATAAGCTCTCCACTCCTACACCAACACAACACAAACTCCGATCTTGTTTCTCTCTCTCTCTCTCCCTTGTTTCAGTGGCTCAGAAATTTTCCTCTTTTCTTATTATTGCTTTCCTTTATTTAAGGAAGGAGTTGGGCTCTCTCTCTTTCTCT

>Os05t0301500-01 2000 bp upstream sequence

CTGATATTGCAAATAGGAATTTAATACAAATGATAAGTTGGGGTTCCGAAGAACAAGCAGACGGACGGTCTAGCCGACACGCGCGCTGCAAGCAAGTAGCAATGGCTAAACTTTAATCTAACAAAACCCGAGAAACCCTGAAGGGGTAACTAGCTATATATAGGGGTGGGAGGACGACCTAGGGGTGCCTAGGGTCGTGCTCCACCAGCTTGGGGCGCACCCCACATGGGCCCCACCTGGCCCGGGGTCCCAAATGAAGTTACAAGCCCATAGGCCCATTATAGGTGATGCAACACCTTGTTCCTGTGATTCTGGCCACACGAGATAGAATTCAGAGAAGAGGCTGGATCCGTTAGAAAGAGGGCTCCGAGAGCTTTCCATCAAGTACTCACGGGCCGAAAACGGAGGTCGTATGCAGATTCGGTGGCCGTTTGAAGTCAGCAGTGTAGTAGGTGGCCGAATCGGATTCCAGCACTTGGAGTACTTGATCTCGATATCTTTTTGTTCATCCATGATGTGAGCAATGGTTGCATCCGTGGTAGCCATGGTCACATCACGATGTCACCTCGCCGACGCCACCGTGGGTGGTGCTCCCTTCCTTCGCTCTGTATCTTTGTCCTCGACCTCCACGGAGGCTAGTCGACGCCGGATGACGTCCAGTAGCGTGAGGCGCCTCCCCAACACCACCGCGGTAGGAGCCAGCTTGCGGCGACCACCGCCCTCGCCCTCATCCTCGTCCTCATCGCAGAGGTTGGACAACGTGACCATCTGCGCGGCTAACATGATGCCTCGTTGCGGCGACGTCATCACCCACACCTTTATCTCCTCCTGTGCGAATTGTTCCTTGAGGTTGCCGGTGGACCCCCGTCACCATTGACCAGGGAGGAGTAGGCGGCGGCTACTTGGCAGCCGACGAAGCACACGGCTCATGTGACAAGGACAAAGTGACGTCGGCATGGGGTGTTGGTGGCTCGTGGTGAGGTCGTGGTGGTGGAACTGGTGGAACTACGGCTTGACGGTGGTGTGCCCTAACCCAACGGCCATGGACGAAGGCAGTGGCCTCGTCGCCCTTGACTGGTCAGACGGTGAGGAAGACCCACCCACCCCCCTGCATCCATATCTCTTTCTCCTCCCTCCCCTTTCTCCCCCTACATCTCCCTCGGCACAGTTCCTCTCGTCATGTGGGCAGCCCGGCACATGACAGGGATCGCGTCCACCGACACATAACTCTCTTCTCCTCCCCCAATGTCGTTGCTGCCAAGCAAGCCGAAGCACTGTGTGGCTACTCGCTCTTCCGGCATTCTACCGTGAGAGGACATCACGCTGGTGTTGCTGCTCGCCTCGTTGCTCTTCCTCGCGATAGATGTGTTCAGCCGTCAGCCCAGCGACGTTGATGCTTTTCCCTGGCCATTGCCACCACGACATGGGCTAACCTTGTGAATGGAGGTGGGCTCGGGGCTCGCAGCGGCCCGCGCGAAGGACAGGGACAGCGACATGCTACGGCACCTCGTCGTTGCTTGCGCCTCCTCTCGAGGGAAGAGGAGGAGCTCAACCACGGCGGCATGGCCCTGCTACGATGGGGAGATGTGGTGGTGGAGGAGGAGCCCGCCATATTTGACTACGCAGGATATCGTTGGTGCCCGCACCATCGCCTACCCACCGGAGAGGTGTGTGAAGGAGGAGAGAGAGAGAGAGGAGGACATGTAGGTCCCACACGCTGGCTCAGCTGATTAGACTATGTCAATGATCCATGTCATCGAAATCCACCCTCAGAACCACCGAGAGAGTCATTTTGCTCCGGTTTCGACAGTTGGGGGGTCAATATATCTGGTATTGCAGCTAATTTGAGGGACTCAATGTAGACTTACCGCGCTGCAAAACGCAGGACCGAGCCTGCCCGGCCCGATTGGGCTGCCGGGTCGGGCGGCCCACGAGCAGGTCTAGCCGCCCGGTCTCCTCTCCTCCACGATGAGACGAGGAGCCGAGGAGAGGAAGGGTTT

>Os05t0301500-02 2000 bp upstream sequence

AAATGATAAGTTGGGGTTCCGAAGAACAAGCAGACGGACGGTCTAGCCGACACGCGCGCTGCAAGCAAGTAGCAATGGCTAAACTTTAATCTAACAAAACCCGAGAAACCCTGAAGGGGTAACTAGCTATATATAGGGGTGGGAGGACGACCTAGGGGTGCCTAGGGTCGTGCTCCACCAGCTTGGGGCGCACCCCACATGGGCCCCACCTGGCCCGGGGTCCCAAATGAAGTTACAAGCCCATAGGCCCATTATAGGTGATGCAACACCTTGTTCCTGTGATTCTGGCCACACGAGATAGAATTCAGAGAAGAGGCTGGATCCGTTAGAAAGAGGGCTCCGAGAGCTTTCCATCAAGTACTCACGGGCCGAAAACGGAGGTCGTATGCAGATTCGGTGGCCGTTTGAAGTCAGCAGTGTAGTAGGTGGCCGAATCGGATTCCAGCACTTGGAGTACTTGATCTCGATATCTTTTTGTTCATCCATGATGTGAGCAATGGTTGCATCCGTGGTAGCCATGGTCACATCACGATGTCACCTCGCCGACGCCACCGTGGGTGGTGCTCCCTTCCTTCGCTCTGTATCTTTGTCCTCGACCTCCACGGAGGCTAGTCGACGCCGGATGACGTCCAGTAGCGTGAGGCGCCTCCCCAACACCACCGCGGTAGGAGCCAGCTTGCGGCGACCACCGCCCTCGCCCTCATCCTCGTCCTCATCGCAGAGGTTGGACAACGTGACCATCTGCGCGGCTAACATGATGCCTCGTTGCGGCGACGTCATCACCCACACCTTTATCTCCTCCTGTGCGAATTGTTCCTTGAGGTTGCCGGTGGACCCCCGTCACCATTGACCAGGGAGGAGTAGGCGGCGGCTACTTGGCAGCCGACGAAGCACACGGCTCATGTGACAAGGACAAAGTGACGTCGGCATGGGGTGTTGGTGGCTCGTGGTGAGGTCGTGGTGGTGGAACTGGTGGAACTACGGCTTGACGGTGGTGTGCCCTAACCCAACGGCCATGGACGAAGGCAGTGGCCTCGTCGCCCTTGACTGGTCAGACGGTGAGGAAGACCCACCCACCCCCCTGCATCCATATCTCTTTCTCCTCCCTCCCCTTTCTCCCCCTACATCTCCCTCGGCACAGTTCCTCTCGTCATGTGGGCAGCCCGGCACATGACAGGGATCGCGTCCACCGACACATAACTCTCTTCTCCTCCCCCAATGTCGTTGCTGCCAAGCAAGCCGAAGCACTGTGTGGCTACTCGCTCTTCCGGCATTCTACCGTGAGAGGACATCACGCTGGTGTTGCTGCTCGCCTCGTTGCTCTTCCTCGCGATAGATGTGTTCAGCCGTCAGCCCAGCGACGTTGATGCTTTTCCCTGGCCATTGCCACCACGACATGGGCTAACCTTGTGAATGGAGGTGGGCTCGGGGCTCGCAGCGGCCCGCGCGAAGGACAGGGACAGCGACATGCTACGGCACCTCGTCGTTGCTTGCGCCTCCTCTCGAGGGAAGAGGAGGAGCTCAACCACGGCGGCATGGCCCTGCTACGATGGGGAGATGTGGTGGTGGAGGAGGAGCCCGCCATATTTGACTACGCAGGATATCGTTGGTGCCCGCACCATCGCCTACCCACCGGAGAGGTGTGTGAAGGAGGAGAGAGAGAGAGAGGAGGACATGTAGGTCCCACACGCTGGCTCAGCTGATTAGACTATGTCAATGATCCATGTCATCGAAATCCACCCTCAGAACCACCGAGAGAGTCATTTTGCTCCGGTTTCGACAGTTGGGGGGTCAATATATCTGGTATTGCAGCTAATTTGAGGGACTCAATGTAGACTTACCGCGCTGCAAAACGCAGGACCGAGCCTGCCCGGCCCGATTGGGCTGCCGGGTCGGGCGGCCCACGAGCAGGTCTAGCCGCCCGGTCTCCTCTCCTCCACGATGAGACGAGGAGCCGAGGAGAGGAAGGGTTTAAAGCGCTTCTTCACTCGATCTCGCCG

>Os05t0310500-03 2000 bp upstream sequence

ATCCTTCGTCAGCGCATGGTCACCGCTGCATCGGCACCATCCGCCGGCTAGCCTCCGCGTGCCGCCACACGGCCACCCTCGTCGCACTGTGCCAGCCTCCACCGCCAGTCGCGTGTCCTTGCGCCGCGCTAGCCTCCGCCGGCCACCGATCCAGGAACACCCGGCACCGGCCTCCGTGCCGGTGCCACCCGCGCCAACCTTGGTAGCCGCCGGCACCTTCATCAGCTTGAGCACATTCGCCGTCATCATCGTTCACCCACACCACACCGTCGTTCAGCCGTCGCTGCCACTCGCTGTCCAGCCGTCGACCTCCTGCCGAAGCCACACTGCCGCGCCGTGGCCAGCCAGATCCAGCGCGGAGTTGCCGGATCCACCCACGGGACGGCTAGATCCGGGCGTGGCCGTTCCGGATCCGTAGCATCCACCATCGCCACCAAATGCCATCACCGTCCCCGCTACCGTCTCATCCCTACTGCTCGCAGCCACTTGACGCCACGCGCCACGGAGACGTGGCCATCGCCATCATACGTCGTCGCTAACCCCACCGCCATTGGCCACCTCCACTGCTGCCGCCAGCCGCCTCCTCCGTCCCACCACCTTCCGTCGATCGGAACTGCCGCCGCCCTCTCGCTAGATCCAGACGGTGCGGCGCGGATTTGGCCGGACCGCCGCCACCCCGAGTGGCTCCCCTAGCCGCCCAAGCACGAGGTGGGCGAAGCCCCGCCGCCGCCGTCCTTGCGGCCGCGTGGCGTTGCCGGCGGCCACTCGCGCGGTGGCGAGGCGAAGGAGGAGTGGGGGGGGGGGAGGAGGGGAGCGGCGAGGTTTTCGCCTCCGGTGCAACCCATGGGGAAGGCGACGCGAGAGTCAACAATGTGGGGAAACAAGCATTAGGTATCTATCTCTGGTATTTTTAATGAAGATGATATGACAGATAGAATGAATGGTTTGTAACCGACCGCTAAGGCACTAACAAGTGTGTGGATCACTCACGTATGCGTATGAGTGCGTCACTCGTTAAAACTAAACTGAGAACGCTCTCGGTTTAGATTTACCTTAATTATAATATACTCAATATATCTCAAGTGGTGTTCTTTTCTCCTGAAGATGAAGATTATGTTTTTTATGCAAAACAATGTGGTATTAACGTATGATTGATTAAGTTTTAATTATTATAAACTTGAAAAATAGATTAATATGATATTTAAGAGTAACTTTCATATAGAAAGTTTTCGCACGAAACATACTGTTAAGCAATTTAAAAAACATGCCACGAAAATTTTTATCTTCGTCTAATTAGTTGGAGAAATGAAGGGGGCCTCAGTTGGTCGTGGTCGCAATAAGAACAGCCAACGGCGAGCACGGCACGCAAGGAAATCAGTTGGTGGAGGAATGGGCGAGACGGCGAGTGCTCCCAAGGAAAAGGATAGGGAAAAAAAATCAGCGGTCTCTGTCTCGTGTGCCTCGCCTCACGGCCTCACCTCGTTAACATATTTTAAAAGAGATAAAGAAAGTAAGAAAAAAAGTAGCGGGCTATAAATCTTTAGCCAGCTGCAATACAGACTTCAAGATATAATATGTGTATGATAGGTAGAATCAGGTATTAATAGTATAGTAAGCAACTATTATATGAATTGACTATTATATTAGCTATAGATGATTTAGAGCTAATAGTTGGTTATACTACTATTAAACTTTTTGCTCTTAGTACCTGCGCGCCCATCTCTATTGATGCTTTTTTTTAATTATTATTTTTTCCCCTCTTGTTTATGATGGCAGCAAGAGGCACAACAGCTATAAATAAATGAGAGAGACAGACCGGGGGCACAGCCGGGGGGAGCTGCTGAGGACTGAGGTAGGGGAAAGAAGAGAAGAGAAGCAAGCAACACCAACAGCAACACGCAAGGAGTGGAGGACTCTTCAAGGTATCCCAACCAAAGATATTTTGCTCTGCCACTATCATCTCCTACCATTAATCCCACTCCCACATCCATCCTCTTGTT

>Os05t0323100-01 2000 bp upstream sequence

TTAGTCATGTAATTAAGCAGATTTATCATCTTTATTTTGGTGCACATTATCACGAGCATAGCAGTTTGGCAATATTTTGTTCTATATTAATATAGAACAAATAAAGGCACGTTAGAGGTGACCTATGACGGGCCTTTACTTTCGGGCCCGTCACAGGTAAATCACCTGTGGCAACTTCTATATACGGAGACCATCACCTCTGACGGCCTTTATTTAAAGGGCCGTCAGAGATGAGTCACTTGTGAGAGTCCTAACTGGCCCTAGGCTAGCGGAGACCAACACCTCTGATGGTCTTCTAGTAACTTGTCCGTCAGAGGTGGGTGTCACTTGTGATGGTCGGCCATCCGTCAAAGGTGACTGGACTCACCAGTGACCACTGATCACTGACCATGCTCAAAGCCGTTAAAGGTGAGGGTTTGGGACCGTCACAGGTGACCTTGGCCAGTGTAGTGATTGGTTGCAAAGAGTCTGAAAACTTGGGAACTTATCTCCCATCGTAAAACGAGATGACTTATTAGCGTATTATTAATTAAGTGTTAGTTATAAAATTTTAAAATTAATTTATATGATTTTTTAAACTACTTTTTCAATGATATTTTTAAAAATGAATTACTTATTTCCATTTTGCGCTCGTGTTTGCTTAGTATACCTATCCTATTCACTAAATAGATTTATTCCTCAGGACAGTTAAGGAGGTGTGGATTCACTTTGTGGATAGATATGATGGGCCTGTTCAGATTGTAGCCAAAATAAACTTTTGACAAGATGTCAATATTACCAAAATTTTGGCAGAATTTATTATGTATTTAGCAAATTTGGCAACAAACTAAACGTAGTTATTTTTTTTGGGCAACTTTGCCAAAAGATGGTATGGTTGAAAATGGCATCAAAGTGAGCAGGCCCGATCAATCCAACATATGATGGCAGAAAAGTAACCTGAAGCTGAAACGCAAACAGAGTATATGAAGGAGCGCGAAGTTGCAGTACGTGCATGAACGACATATAAGAGGTCCGTAAACAAGCTCAAGCCCGATTCTTCGGAAACAACAATTTCTTCGCCATCTGCCAGTGCTCTTCCTCTAACTAGCATTCAGTCTTGTAGCAACGTCAACCGGTGCTGTCTTACAAGGGCAAAGAAATTGCGGCAAAGAGATGAAACCAACAATTCCAATAAAAATAATAAATAATACCCCATTAATTCAAACATTTTAATTAAAAAAACAACTACAAAGAATAGATCACCCTAGGGGTGGGCAAGGAATATAAATCACGCTCCCCTAAGACAATTTATATAACATGGTAGGCTGGGGAGATGCCGGTCGTGCATATTTTACAGGCGGCTTCCTCCTGGCGATAAAATTGCCGGCCGCATACCAAAAGACCGCGGCGCGCCCCTCTCCTTGCCTGCAACGTCACATTCCCCACACTCCCCTATGGCGATTTATATGCATCGTCCAAGGTGAGGGTGGGAGAAGTATAGTTTTGTAAAATTTTGAAACAGGAATTTTTAAAAAATAAATATAAAAATAAAAAAATACAGTATGCATATCCGTCCACCATCCTCCATCGTCCTCCTAAGAGCATCTGTAGTGTATAAGGTCGACGCGTGAAACGCATGATCGAACGAAATTTCGATTCGATCTTTGCAGTGTAGTAATCGATATGTGGGACCCAGTGGGACCCGCATCTAAAGAAAATCTCAACCTCATTCTGGTCCGCCTCTCTCACGTAACTCAGTGAAAACTTTTTTTTATTCGGTTATGTTCGACTCCTTTGTAGTAGTCGATTTTTTCACTGACACATATGGTTCGAGTCTCACAGTGTACTCTGTTCGATTCTTCTGTCCAAGGTTCGATTTTGTCTTTACATTGGAGCTGCTCTAAGGCCGACCTAAAGCCCAATATCAGATGCGACTCCAAACCTTACTAGGCCGGCCTAAAGCCCACAGCTCGCAAGGCCCAAAATCACAGATAAAGCAAAGGCTCTCGCAAACTTGACCT

>Os05t0419600-01 2000 bp upstream sequence

AGCCGCCACGTAGGATAAAACTGGGGTCAAAACCACCGAAAGACCTATTGTGACCGGTTTTGGTTAGTTAAGAGACGCCCGATATCTGGTTTTGCGGTTGGGGGACGATTTTGTAACTCGATGACAAGTTGAGGGACCTTCGGCGTACATTTTCGATCTCGGAATAAATCCACTTTGATCCCTTCAAATATACTACCTTGTTAAAAAAATGAATTCCTAGTTATGGGTAGGATTGGTCTTTTTTTGGATGGCGGGGTAGGTCTAATCTAACCCGTATCTTTCAACCGAAATACTTATCATATATTTTGAACCTCCAACTATTAAAATAGGTGTACTTTGACTCTCACGTATTATTTTAAGGGCGGTTTTCGTAGACGTGACTCGTTGACATGGTCAAACCGACTGAGTTAGCATGTGGGACCATATGTCATTGACTCTTGCTCTACCCCTCTTTTCTTTCTCTCTCTTCTCCTCTCTCTATTGCCCGACAGTGCATGATGGCCCTGTGACCGCTACAAGTACTCTCAACTCCGTCGCCGCGAGGAGAGAGAGCTTGTGGAAAGGCCAACGACGATGGAACGTGATTGCATGGGCCGCTTCATCTCCTTCAACTTCAACAGAACGACACCCGGAGAACCCCACTGCCTTGTCACCACCAATATCCATCCCTGTAAGGCTGGATCGCATGGCACCAAGTCATCGAGGATGGAAGCCATAGCACTGTGGCTGTATCCGCTCAGTAGAGATCCCGAACACCGCGACGTCGGCCTCCCCCTCCCATCGCTGCCACGACTGTTGCTCGTCATCGCGTGGGGAAGATGGGAGAGGAGGAGGTGACGGCAAAGCAGTGGTAGGAGGCTAGGAGCAGAAACTTGGCGGCTAGGGTGCTCACCGTTGTCTGGAGCTCGAGGAGCCACTGGCCACCGGGAAGGAGGTGCAGCGATAGTCAAAGCCAGTGCTGCGTGACCCCGTCACAGAGTTCGCTGGTGGACCCATCGTCATCATCGTCCAGGGAGGAGCAAGGCAGTACCCCATGTGGCAGCCGATGAAGCCTGTGGCTCCCATGACGAGGACGGAGATCCCTCACCCGTTGGCCCGCCTGACGTGCGCAAAGGCTCGCACATTTTTCTCCTAGGCGACGCCGCCCTAGGCAGTGGTGGAATGGGAGGACGTGTGGAGGGAGCGACACGAGGATTTGGGGGACCGCCTGCGATTCTAGGGTGGTGAAGGGGAGAGGAGTAGGAAGGCGAGGAGGAGCGAGAGGAGAGGGAGCGGAAGGACAAGAAGTGACGTTGGGGAGGGGTGGTTGTGGTGCATGGCGAGTCAGCGGTTTTGAAGGTCGTAGAACTGCAGCTTGATGGCCACCGCGCCGCTCGACACCCCTGTTGTGCCAGGCACGCTGGCCACCATGCACAACTGCATTGCGAGGCAAGAAAGAGGGGAGGGAGGAGAAAAACGAGAAAGAAAAAGAAGGGTTGAATAATAGCCATTGACATGTGGGTCCCACACGCTGACTCAGCCAGTTGGACCAGGTCAACGAGCCATGTCAGCGAAATTCGCCCCTCAATACCGCCGAAGGAAGTTAAATGACACCGATTTTAATAGTTGTGGTTAAAGATAGGGTAAATTGGAACCATGCTATTATAATTTTGCAAAGTTTGAGTTATGTCATCGGTATCTCAATGACATGTAGGACCCACTTGAGTCAATGACATGTGGGTCAGGATAGCATATCTCAAATTTTGCAAAATTATAATGTCATGGTTCCAATTTTCCCTTAAAGGTACATGGCATTCCTCTTTTTTTTCTCTAATTAAGGCCCATTTAGAAATGTTGGGCCTGGAAAAAAGGCCTGTACTGAGGGTGACGTCGACCTGTCGTAGGGGGGCCCACTGCCAGCCACACGGAGCCGCCGAGAACGACCACGCAGCTACCATCCCGACCCGACGCACCACCACCTCCCGACTCCCGTAAAACGGAACGGAGCCTTCGAGGCGGGG

>Os05t0423500-01 2000 bp upstream sequence

TACGGTCATCACTCTAAACCTGGGGATCTCCCCCTTCTTCCTCCTCCCTCCAACCCCAAAGCACGTTCGGTGGCCTAAAACCTGCAAGATTTAGGGATGAATTTTTCATCAACAGAAGGTTAGCAATCGCTAAAACATAGTAGTTTGTGAGTCGCAGTTGGTTGTACCATGCACGCTTTGTATCGGGCTCTGCTGGCATGCACCATGGGACGGGGATACGCTATTATGTCTATACTTCTATAGAACCTCACACAAGGTGAAAATGGTAGGAAATTTCCCGATCATACCGCCTACCTTTCAGTTGATTTGTGCCAACCATTTAATTTCGATTGTATATTGTCCGGAAATGTTGAAGTTTTATTCGAACCGTTTTAGTAGTATACGGTTTACTGACTGCCCATCCGAATAAAAAGAAACATAATATTTATCTGTTATCGAAATATTCAAGAGTTTTATTCTAATCGTTTTAATAGTATACGGTTTACCGACAGCCGATCCGAATATAAAGAAGCATAATCTTTATCTGTTATCGATTATTCTATTTTAATGATGTATTCAAACCAGTTATAGCTTTGAATGTTCACTAATAGTTTCATGTGACGTTAATTTGTAATGTAAACATGACTATGACTTATTTACCCCTTGATATATTTTTTTGGATGCTCTATTTTGGTAATTCTATTTTAGATATTTTTTTTCGATGTTTCAATAGTACCAATATCGTTACTATTGAATCTGGATCTACCGTTCAATCACGTTTCCAAAAAGAATTAGTTTGAGCTTTTATTTGAACCGTTTTATCCCTATAAGTAGCAATGGTGCTGTAGATTTTCCACAAGAGTGATGGAAGCTAGCTGAAAAAGAATACTCCGTATCGCTTTTATGGCAAAAGTCGTGGAGAGGTAAGGATGACGACGAGAACGAGCTAGGCTGAAAAGCTACAAGGGCCAGAAATAAATAATTAAATAAAGGCATACTTGTTGTAGTGAGATGGGGATTATAAGATTATTCGGGTTATGGTGGATGAGGGAGAAGTATTTGGGAACATCCCAACTTGATGGGCTTGTTCAATTTGCTATCATTTTTAACTTTATCTTTTTTTGGTAAAGTAGAATGGCTATATTTAGTTTGTTGTCAAATTTTAGTAAATACATATGAAATCTTGCCAAAATTTAGCAACATTGACAAAATTTTGACAACCTTAGCTTACTAAAATTTGGCAATGTCAAAACTTGGTAAGGTTGAAAATGGTAGCAAAGTAAACAAGTTCGATATTACAAAAGTCCTAATTGATTTCTTTTACATTGATTATTCAAACGAAATAGCCTCCTAGGGCCTCATCGTTTGGCATATTCATATGCTTATCAGCCAAATTTGAATTTTCAACATTAAATTTGGAGCTGAATTTGAGGTTTTTTCATCGAAGTTTATTTTTCAGCCTTTGCTTTTAGATCGCTAAGAGCACATATATAAAAGTTTTATTCACAAATTAATTTTTATTTGTAAATATGCCATATTCAGAAAATATGCCAAACGATGAGGCTGCTAGTGATTTAACGTTACATATCAATCTAAGAGTAGGATGGTACAAACAGTAAACAAATGGATAGCTTAGAGCCAAAAGACAGGAACATTAGAATTTAGTACTAGAAATCAAGCACTAATCCACTCTGACACGTCATCATGTATCTTCTAATCTACGCCACGTCATTAAAAAAAACCTTGACCTCATATCAGAAACTTTCTTTTCGGCCACCACGTGGGCCCACCTCTCGTCCTCCTCATTTCGAGCGACCTTCCGGTGGACCCCGCCGGCGGCCATCGGCGTCGCCTACGTCGCCGTCAAGCCTCCCCGGCGAGGTGGGCCCCACCTGTCATCCTCCCCGGAGCGAGACGAAAAGCTCCGCGCAACGTATAAATTATTTATCCCGTACACGCCACCTTCATCTATAGCCGCCGCATTTTACTACTGCCCACCACCACCACACCACCCACCCCCT

>Os05t0443400-02 2000 bp upstream sequence

TCAAATTGTTAAGTTTTACCTATAAATTAACTTTTATTTCCTATACGATTTATTAGAAAATATTAGCAACCAATGAGACTGATACATGTGATGTGGATTTCTTCCGTCGGAACATTTATTTTTCTTCGTCGTTGCGTCGTTTTTGCTTCCAGGATTTTTCTTCAAACCCATATGTTCTATTTAGTGTGTTCTTCGACAGTTTGGACGTCACCAAGCAGGCCAAAAACAGGTTGGACCTTGTGACGGAAGGTAGCACCACTGCCCGTATACGGTGATGGATTTTTGCCGCTCAGGATATCGTCTTGTTTTTCATATTCCCCATCTCAAAATATAATAATTAGTATAGGATGGGACACATTCTGATAACACGAATATATATATGTTGTTATTTTGAGACGGTGAGAATATGCTATTTAAATACTTATAAAAGAATTAATTGTATTAGATTAATTAAACCCTACAAAGCAAACAAGTACTTAAAAAATAAGAATTTGAACTCTACCTAGTGCGTGTACTATTATGTCATATTTGTTACTAGTATTTCTTTTAGTTTTGGTACCGCTAGTATAGGTTAACTTTGACCTTGCATATTATAGAATGCTATATTGTGCATTGATTTATTTTTTTTTAAAAAAAATATATTCATAACTTTTTAAATAATAATATGAAGGAAACTACTCCATCCATCCCATAATATAAGGGATTTTGAGTTTTTGTTTGCATTGTTTGACTACTCGTCTTATTTAAAAAAATTGTGCAAATATAAAAAACGAAAAGTTATGCTTAAAATAATTTGGATAATAAAGTAAGTCAAAAAAATAAATAATAATTCTAAATTTTTTTGAATAAGACAAGTGGTCAAACAGTGCAAGAAATCTCTTATATTATGAGACGAAGGGAGTAGAAGACACGAGCGAAAGAAAAAAGAAGCTTCCCAGACTACTGTTTATCAGAGCAGGCGAGTTGCCACCGCTATAAAGAGCATGGAAAGGTCGCGCGAGCCGGCCCCCGGCATGAGAGAAAACGCACGACCTTAATAATGCATGCGTAACTGTTCTCCCGTCACGACCTCAAGAGAGCTCAGCCTAAGGCGGGCAGCAGCTAAGCCAGAGGCCGGGATGGAGCTCTGCGCCGTCTTCTTCTCCTCGGTCTTCTCCCTCCTCCTCCTCCTCCTCCTCTCAGCAGGTAGGGATACTCATGGCTCCTTCGTTTCATTCCATTCGTGCGTTCCTTGCGTGTTTTTGTCAGTGTGGATCATATGGAAGACTGAAACACAAGGATCAAGGATCGATGGTTTAGTTCCTTGTGTTCTCTGTTTCTTCTTCTCCTTCTCGTTTCATTTCGAAGAGACCATTGATGGCTTGGAGCGCTTTGAGTGCAGTTTTAAGAATTTAAGGGTGTTTACGCAAAAAAATAAAATAGAATAAAGGAGAGTAAATTTGAGCGTGGAGAAATGCGACCTTTGTACTACCGTGCAAAACAGCTGTGCTGGTGTTATCGTCTACAGGAAGTGAAAAACAGTGAATTGAATCAATTGATGATGATGCAGTTGTCTACAATGAGCCTATGAGGTCGTTTTCTTCGATTAATCAAGCATGTTTCAGGCGGCTGGTCCGACGTGTACTGGATAGTGAAAACATTGAATATTCCGCACCACGCGGTGATCCTGATCCTTTTGCTGAAAATTTTCACGGCCATTTGATTTGACATTGTTTGATATTTTTTTCTCCCTTTGATTGCAAGCTCCGTGTTTCTGCTCGTCGAAAACGACCTCCTACCCAAGATCATTTCATGTAGTTTAATTATGTGATAGGGAAACTTCCCCCCTGGATTAACTTCTTACTATCTCTTACGATATTTCGAATGACTGGTTAAAAAAAAGATATTTCAAATGCCTTGCTGATTATAGCAATTAACTGAGCTTGAAATGGTAAGCATCATGTTCAGACATGATAAGCTCTCTTTCCCTGACAAAAACAAGTACCATTTTTTTTCTTTTT

>Os05t0475300-01 2000 bp upstream sequence

AATGAGTTGTTCCGTTTTATATGCTACAGAGATTAGTTCCCACCCAGGAGATGCTGTCCTTGATCATGACTGACCTGGAAAGTGCGATCGGAAGTAGAACACGATGCATATTTTTCATATATCGTTCGTTCAGCTTTGGCGGCGAGACACGGCACAGGTGGAAAATACATGTCCGGATCGATGACGTGGACCCATGCATCTAGCTCGTGTAAATTTCGCTTTCTTACTCGCGACTCGCGAGACTCGCATTTTCAAGTCAACGTTTTTGTTCTCTTCTCCCATGCGCCTCGTTCTCCTCACCAAGCAACCAACGGGGACAAGCCGCCAATCCGATTTGATTATTTATTGGATTAGTCAAATATGCGATAGTAGTTTACTAGTAGTTATTAGTTAAATTGATAATCAGTTGGAAAGGATAAGGCGTGACGGCAGATGCTTCGCGCCATTTTTCAAAAGTAAAACTTTAGGAAGCTACGAGTTCAATCATACTACTGACTACCATTTTTTTTTAAAAAATACTAACTACCATGCATTTCATTTTGTTTTTCTTTAATATTTCTATCTAAATTTTTAAACCTACAATAACTTATTTTTATTCTTTTTTCTTGTGTTGCTGGGTCACAGTTTTGGCCTAGACACGCAACATGATAAGCAAAAATCGTTCTATACGTGTAGTTCTTAATTTAACTGTTACGACGCTTTGGTTTCCAGTTATGTGGAATTTAATCTCTTTATGTTATACTTATAGTATGATCAAAGCTAAAAAGCCTCGTTGATTAGCGGCGATGGGACATGAATACTTGGCGAATCCTTCTAGCTGGCCCTTGCTGTCGGAAAGCTCGAAAGGTCTGGTTACGTGCCGCCCAAATAGATTAAGGCCAGAATAAGTTTCCCTTTCTTTATATTACTATGTAATAAATAATAATGATGCGTATACAGTACAGGTACGTGCAAACGTAAGGAGGTTAGGGATATATATAAGGGGATTATTTCCATGGACGAAATTAATCTATTTTGCTACAAAAAATGGCTTTGTGGAAAAACTGAAAATTAATGATAAACTTCAAAACTATATTTATTTAATCTAAAACTTGAAAACCATTTGCATAAAGCTAAACTCCCTTTTAACCATAAGAATGTTAAATAAAAAAATTATAATGTCATGGTATTATATAATTCCTTTGTTCAATCATAAGCACATATTTCCTCTGTCCCATAAAAAAACAAATCTAAAATAGGATGTGACATATATCTAGCACTATAATTTGGACAGAGATATGTCCAGATTTATAATATTAGAACGTATCACATCCAGTACTAGATTGATTTTTTACGGGACGGAGTGAGTATATGGTTATCCTCTTTTACGTTGAATAATTATAATGATTTTTAAATAATTACTCCATTTGAGGGTGTAAACAAAATAATTTGATATTATAATGCTTAAAAATAAATATAAAGAAAAATCATATGTAGAAAGTTTTTATTAAAAATACATATTATTATTTAAAAGCTCGAGAAACGTGTATACTATTATTCCATGCCCTTCTTCTGTAATAAATAAAACCATAAAAAGATCAACAATAACACATTTTTTCTATTGTTCATCATCTGAAACAATCTAGACGTCTCCTCCTTCCAAAAATGACAAAATCAGCCTCTGCGAGACAAGCCGAGAAAACAGGATTTGCAACTTGCAAGTACGACTACTAAACTGTCGTCAATGGATAGATAAACCCCTTGGACCAAAACCGCTTAAACCCAAAACAATCCACACAACACCATCCCCCGATTCCTCCCCCCTACCGTCTAAACATGGCCCGCCGCCAGGTGGGCCCCGGCCCCACCCGTCATCCACCTGATTGCCAATAATCCGATGTGCATTAAACGCACCAGAACCGAACGAACCAATCCCGTCTCCTCCTCGCTTTGCTCCTCGCGATTCCGGAGTCCACCTGCCCTCCTCGTAAAAGGAAGGCGAAAAATCGAAGAATTTCTCG

>Os05t0547600-01 2000 bp upstream sequence

GCAGTGGGCTTAGGCGGGCGGGAGCGAGTCGCCACGGCGGTGGCGGTGGCAGAGACGGCAGGGCGGTGGAGGCGGAGGTCGGGATGTAGGGGAGGGGGAGCGGCGCACCCGAAGGGGAAGAGGGACGGCGGCGAGGAAAGCGGCGCCGGAGAAGGGAGAGAGAAAGGGGATCGGGGAGACGGGGGAGAGAGCCAGAGAGGGGGGGAGAGAGGAGGCGGCGTGGGGAGAGGACCGGCCACCGATTCCACGACCAGGCAAGGCCGTACGTAGGGAATGAACGGCTAGATTTCGTACACGCGTGAGAAAGAACGGCTCTGATCAGCTGAACCTAATCCGAAATACCGAAACAACGAGGGGTTTTCTGCAAAATTCAATGGCGTGGATCGGCTGAGCTCGCGGCGAATCTAGGACGTTTAAGATATCTAGTATTCCGAGGCAATATACAAAGCTCATTTTTTAAACGAATACAGTATATATACAAAACGTATACAATTAAAGTCCAAACTTGCTACATGCAAACTATATACGTAGATTTTTTTTTAGAAACATTCCGCAGAAATTTTATGCATATGAATTTGAAAGAAAATTAAAAATGTACAAACTTTATACGAGTTAAAGCACAAATTTATGCATGGAAACTATCTCTAATAGAAACTTTGAGTAAAGAAACTTTATACATATAAATTTAAAATGAATTTAACAAAGTCTACTTATTTATACTTTAAAAGACGCTGTCGTCTTCCTCTGTGTCCCCTACTGCGTGCAGCGTGAGAATAACACACCAAAGACCAAACTGTCCCACCCCTCTAACCCTACTTCACTACTTCGTATTACTCCAAATCACGTTAGTGTACGCGCATAACACACCACGTCAAGAAAAGCCGCAAGGCCTCACACAGAAAGGAATAAGTTCACTTTAGGTCCCTCCTTTGTCACTGAGTTCGAAATTCATCCGTCGAGGCACAACACATCTCTCAACTTACAAAATCAGTGCAAACGAGATCCCTCAGCAGTACTGTCCTGGATTTGGATGACATGGTGCATACTTGGCTCCTTTGAGGCTTTAACTAGGTCTTCGTCTCACGTGGCATTAACGTTGTACTTACATGGCAATTCGATCCGGAAAAAATAATAAAACACGTGAGGCCCACATATCAATTACACACAAAAAAAAATGGTGGGCCCATGGGGCCCCACATGTCATCCTCACTATCCCCTACTCTCTATCCCCCCTCTCTCTCCCCTCTTCTCTCTATCCCCCTCTCTCTCCAACCAGCGGAGCGGGGTAGGCGGCTGCCGAAGCGGGCGACGGGTGGGATGGAGTGGTCGGGCGGTGGAGGGGGCAACAACGGCGGAGGTGGCCGGAGGAGGAGCTCAAGCAGTTGCTGGTGAGCACGACAGCTGTGGCGGTGTGACAGCGGTTGACAATGACGGTCCGCTCTGCACCACTGGCATGCTCCGGTCGCCGCCGCTCCGACCAACGCCCATCCCGCTCCACCGTCACTAAGTGAAGCCATTAGAGCATCTATGAGATGAAACAACTTACTACTAGAGAGAGGATAGAGAGAAGAGGGGAGCAGTGAGGATGACATGTGGGACCATATAGCCCCACTATTTTTTATTATTTTGTGTGTGAAACTGACATGTGGGTCCATGGGTTTTATTATTTTTCCGAATCGAATTGCCTTGTAAGTCCATGTCAATGCCACGTGGGATGAAGACCTAGTCAAAGGAGCCACATAGGCGCTATGTCAGTCAAAACCGGAGATAATACTACCGAGGGATCTCGTTTGCACAGTTTTGTAAGTTGGGGAATTTAAGACTCAATGACAAATGGAGGGATCTAAAGTGAACTTATTCCACACAGGAAAAGGAAAAGGAAAAGGCGCGGCCGCCTGGGCCAGAAACGGAAGAAAGCCTGCGTTCGCGAGCATTCGCCCACTAGGGTTTTCCAGTCTCCACCACGGCACCACCACTCTCAGGCTCTCAGCTCAAGCCTC

>Os05t0553400-01 2000 bp upstream sequence

GCATTCCTTTTTTTTTTGAGAAACTACCAGAATGCCCGTATGTTGCAACACGATTTTAATTAATTAAAGAAAATATCATTAATCTATTATTATAATCTTTTAATTCAATTGATGTAGATATTTGTTCCTGACTTGTGGATTTTTTTAGTCTCACACTCTCCCTTTTTTTTCTTTCTCATGGGAGTGGTCGATATGTGCAGCGTCTAACACCACATGTCCTTTTTCTTCGAAAAAAGAGTTCTCCCTTATAACTTTTATAAGGAGTATAGATAGTTTGCATTCCTTGCTTGCTGCTTTTTTTATAGATATAATTTGCATTTCCTTGTTGCACTGATCATTAGCTCATTAGTTCATCCCATGAGGACATGATGACAGTATGACACTACCCAGAATGATTATTTTTCACTTAATATTTGGAGATTTATCTCGACGTCACTGGGTGGCATATAAGGGATGCGACAAACACTCCATATCAATCGAGGAATTTCCTCTCGTCTTGAATTATGACTGATCATGACTTGTCATTAGAGAAGTTAAAGAAAAAGATGGGACTTATCAGGAGAGACTAAAAAAAGAGTAGCCCTACGAGTAGTTTAGTGTGTTTTGCCATACTACCATGTGATGGTGATGTCAAAAACATGGGGCTCGCGTAACCGCAAAACTAAGGCTGTCCCAGACACACTAATCAAAAGCATTCGAATTTAGAACTCCTATAATTTGTTAGTTTGTTTAATCACTCTTTGTGTCTCATGATGATTCATGCTTGCATAATTGCATACACTTCTCGACTCTAAGCTTGGACCACACAACCCGTACATAGGACGAAACCGAAAGCATCACCCAAAAAAAAAAAAACTAATGTGCCCCTCTTTAGCATGTCAGAATAATATGGAAAAGGCGTCGAGCAGAAACAAATATGTCAAGAGTTTGACAAACCTGACAACTGCCAATGCAGCCAATGAGCCAAGCAGCAACTTGTTGATGGCTGGTAGGCTTTCCCATTATATTCTGGCAAAAACTTTGCTTCCATTTTTCTATTGACGACGATGAGTTCGCCGATTCCGGCCGGGCCGGCTCAGCTTTTCTCCTTCGGCTAGCGTTTGGACGCACACTTTTGAGAGAATCGAAACACGGAGAGAAGTGCTCCGATGCATGACACCTCCTTTTCCACGTACTTAACGCCAAAGGGAAACAATGATATGCAGTGAACACAAAGGGATTGACCCTGCCCAGGATCATCAATACACGTTGAGGCTTCTTCGTGTGTGAAAGCATGGCTAACTCGTCTGGTCAAAGGCAAAATTGCTGCTATCCTAAAACAAATAGTACTAGGATAAATAAATGAAAGGGCTAATATAACACTCGCACACACGCGAGACGACAAAATGGAGAGCTAGCACACACGATAGGGACGGCTCGACGGTAGGGGAGGAGGAATAATGCAGGCAGGATGTTACTATGGCCACGTTTAGTTGGTTAGTGTGACAAAAAATTTTTAAAAGTATACAGACATACATAATATATATTTAAAGTATTAAAGGTAGACTAATAACAAAATAAATTACAGATTTCACATATAAACTGTGAGGTGAATTTATTAAGTTTAATTAATCCTTCATTAGCAAATGTTTACTGTAGCATCACATTGTCAAATCATGGCGTAATTAGACTCAAAAGATTCGTTTCGCAATTTACATGCAAACTGTGCAATTGGTTTTTTTCCATATTTAATGCTTCATATATGTATTCAAATATTTGATGTGACGGAATTTTTTAAAGTTTAAATTCAAATATTTGATGTGACGGAATTTTTTAAAGTTTGAAGGGAAATAAACACGGCCTATGGCGCAAAATTGTACTACAAACTCCCTTTCGCGAGACGAGAGAATGCAACGCCGTGCCGCTCTGGCGGCTGGGGCCCAACCGCGAAAGGCAATCCATCCCTCTCTCTCCCCGTCGCGTAAGGCCGCTATAAATCAACCCGTCGCGGGTTGCATTGC

>Os05t0567200-00 2000 bp upstream sequence

GAGATTTCATTCACCATTGGAGGCAAAAAGTTTGCACTGAAGCCAGAAGAGGTGCGATGATTTCTTTATGGTTTCTCTTCTTTTTTTTCTTCCCTTGGGTGCATCTAGATTAGCTGAAAAGTTGGACACGCTTGCCGGCCCATCTAGTTTGTGTAGATATTTGACGCACTGGCTTGAGCACATAATTTACTCTCTTCTTTTGGTTGTGCAGTATATTCTGAAGGTTGGTGAAGGAGCTGCTGCCCAGTGCATCAGTGGATTCACAGCCATGGACATCCCTCCTCCCCGTGGTCCTCTCTGGTAAACTATGCATTTCCAAACCCTTGAATTAGCAGTGAAATAATTTCACTTCTTGTCATTTTTGATCTTTGACTGATGCACCCTACAATTTGCAGGATCCTGGGTGACGTTTTCATGGGTGCCTACCATACCGTGTTCGACTACGGCAAGATGAGGGTTGGCTTCGCGAAGTCGGCCTAAACGTGTTGTTGTCACGTTCCTAATGACGAGGTGGACGCATACGCCGAGAGAAGAAGACCGTAGCTTTTAACTAGCGTATTTATCCGGCGATGTGTGTATATATGCATGCTACATAACATGTTTATGTTTTCCAAGGCCTGAGATGGCGCCCGTCTATTTTAGCTTGGCAAGAATATCTCGGACCAGTCTCTGTTCGAATTCTCGTACTGATATGTGAACTTCTGATAACATGTGCACTGTGTTTTGTGACTGAATTGCTAGTCAGTCCGAAGGTTAATTATCCCATGGTTGATGTGAAGAGAGCCACCGGATTATTGCAGCTGTGATCACGTGGTTAAGCCTTAAAAGTGTGGTGGTTTCTTCAGCTAAATCTAATGTGGTGGTTTCTTCAGCTGAAGTCTTTTCACCTAAGTAACTCTTACTCTCTCCGTCCCACAATAAAAGAGATTTTTTTTCTTACAACATTCGACCACTCGTCTTATTTAATTTTTTATAATTGTGTTTAAAGTACTGTAGATAATAAAGTAATTCACAAATAAAATAAATAACAATTTCAAATTTTTTTTTAATAAGACGAGTGGTTAAACGTTACAAGAAAAACTCAAAATCCCTTTTATTATGGGACGGAGGGAGTACTAAAGCTTGTTAAGTTCCTCAAACCAGAATTACTCTGAAATTCAGACCAACTCCCTCATGAGCCGCCGGTACACCTGCAAAATTTGACTTCAAAACCCCTCAATTCCCTTATGTGCCGCCGAAACACCAGCAAAATTTGACTTCAAAACCACCAATATAAGTGATCCAACTGATATCAACCTTCAACATTCAAATTTGACTTCAAAACCACTGATATAAGTGATCCAACTGAGATCAACCTTCTTCCAACATTCTCAGGCGACTGACAAAGCTGGTCAAGTAACCACCGAAAATAGACACACCCTCTTTTTTTAGAAAGAATCCGTTGCTGGACGCTAGCAATGGCAGCAGGACTCTCCATCCAATCCAATTCATAAACAAAATAAAAAAAAATCTAGTAAAAGAAGTTGTCAACGGCAGTCTCTCCTCCATCCCCCTCTCCGATCTCTCTGCCCAGATCACGACTCGCTCGCGATGCAAGTCCGCAAACGGTTGCCGGATCAAGCATGGACTCTCTCTCTCTCTCCACGAAACCGTCAGACCCGATCGCCTCCTCTGACCTTAGACTTGCTTAGACGCCGCGCGCATGCAGCCAAGCTGCGAGCTGCGATGCTGCGAGCTTCATCCATTCCCATCCATGGCGCTCGCGTCGCTTGTGGTGTCCACGCATCCTCAGCTTAACCCCCCCTCTCTCTCTGGTCAATGGTCACCAACACTGCCTAGAATGATGGACTCAGCTCCAATCTCATCCCGCCACGTGTCCCCCGTCTCGTCGTTATATATATACACACTACGCCCTCGCCTTGGCCTGGTCGTCTACTCTCCCTCCCACCGCTCGTCTGTCACCGCCGCGCGCGACGCGACGGCGAGGTGTGTGTGTGAGA

>Os05t0581800-03 2000 bp upstream sequence

TGGGCAAACCCCAAGCTAAACCTGTGAGATCTGTTGCATGTAATACCGGGATGCCAACCATTGGTGGTTCAGTCATTTCCAATGCAACAAATCATACTTCAAAAGATTCACAAGACCTAGTTTTGCCTTCACAAGTGAACTCTGTTGCAACTGATAGAATACCAAATGGTACCAATGAGGTCTCCCCTGCATCTAATGACTCCTCTATTGATGTATTACCTCCCAGAGAGGGGTTGGAGGTGCCAGAATCTGTCGCCACAGTTAAACCTGGATCGTCAACTGCAGATGTTTACAAAGATGAAGTTGAAGAAGACATGGATTCTGATAAAAATAAGGATATGAGTGCAAGCAATGCAGACGATCGAACATCTTCAGGACCATATCCTGCATCTAGCAAAGAAGTACACTCAGAACATACTCAAATTGCCACCCATCACAATGACTTGATAGTGGAAACAGAGGACTCTCAGTCTGATGGCAATGCATTTGAGAATAACCGTGGTAGGTCATCTAAAATTGCCCCCACCTTTTTTATGACTTCATATGTATGCCTACACCAAATGCGAATATATATTCTAGAAATCTAGAACATAGATTTCGTGTTTCGGCATGGTATAGTTTTGTCTATAGTAACCAGAATACCCATCAAGCCATTGTTATTACATTGAATCGGAAGCAATCTGATTTTATTTTTGCTGGCGGCTTGATCTCTGCCTATTTGTCATTTGGCTTATGTGTTGGTGTTACTGTCTGACATGGATCTTAAGCAAGTTCCCATATCCAGCACTAAGCCCGTTTCTTTCTACTAGCTACTGCAAGTCTAAATCCGATACAATACTGTCTTAAATTTTTGCGTTTGCTTATATAACTACTAGTACTACTTAGGACTGGAACCACTGTCTCCATCAACATCTTGACTTTAGCATGAGACTATAAATCAAATCCTCATGATGAGTAATGAGTTCAATACAAATTATTTATTTTGAATCAAATTCTCCCTCCGTTCCAGAATATAACAAGTTTTAGGGTTGGACACGATTATTAAGAAAGTAGGTAGAAGTGAGTGGTGGAGGGTTGTAATTGGATGAATAGTGGAGGTAGGTGAGAAAAGTGAATGGTGGAGGGTTGTGATTGGTTGGGAAGAGAATGTTGGTAGAGAAGTTGTTATATTTTGGGACAAATCCTAAGGGCTAAAAGTTGTTATATTTTGGGACGGAGGGAGTACCTTTTAAGGTTGTTGCTCATTTACCCGAGAATGGACCAGTAGAAAGTTTGATATATTTTATTTGTTCGTATGGCTCCAGCTATTCACTATTATTACCTTTTTATCCAATCTCTACTGAAACTACAACTTTTCATATTTTACGTTCTTACCAGGACCTTTTTATTATATCTTAGTGCAGCACCACGGCCTGTGAAGGTTCTGCATGTGCCTTTTTATATTACATTTTTACACTATATATTTTCTAATTGGCTTTTCTGTTGGAAGTTACGAGCCTCCTTTCTAATTGTTTATGATAATCTATTTCCTTAGTTGCAGATTCAGAGGGAAATATGTCTGCTACTGACAAACAGTTTGAACAGTTGATTCTACATGAGGAAAAAAAATCAAAATCATCTGAAGATAACCCGGCTGTAATAATTCCTGATCATCTTCAGGTTTCAAATGCTGATTGTGCACACTTGACATTTGGTAGTTTTGTGTCTGGAACACTTGATGCGCCAGTCTCCTTGAAAACTGCCAATGGTGATGAGGAGGTTGCAGCAGTTTCTGATAACCATTCGATTGATCAGTCAGATGTCAGGTATGTATTTGTCTGTCGCATTCTTCACTTCTTTTCAAACATCATGTAACATCTATTTCCATTTTTCAGAATCCATGAGTACGAAAATAAGGATACAGTAGCACCTGCAGCTGACGAACATGTTGCCTCTTCAACAAAAAGTGACACGGAGAATGTTGATGTTGCACCTGTACAACAGCCTGAATTGAGAACAGC

>Os06t0137600-02 2000 bp upstream sequence

TAATTAAAATCATAAATACTGGTAACTGATTCATGTTATAAGCATGATATGATGATAGCATGACATCTGTATAGTATAGTGTTCTATGCTAGTGAATAACAGGAATCAGCACAACACTCATTCACTGACAAGCCTCATGAACTTCTGCAAACTCCCCCAAGCAAGCAGCTAAATCCACTTATAATCGTTATCTCATCCTTATTCCTTAACCTCGGCAAGGCACCACGTTCCAGATAGCCTTCCCTGGTCATAACTAGTAAAGAAAGAATTTGCCCCAACCTCACCTCATACCTAGCCTAACTACCAAAGAAATGTAATTCAGAAATTGCCCCAACCTCACCGCATACCTAGCCTAACTACCAAAGAAATGTAATTCTCATGTTCCACAAACCTAATGCAACATTAAGGAGACCCTGTCAGATAAATGTCCTTGATTCCAAACTACTATTAGTCAAACAGGTTCGAATGTACAATTAGAAATTAAAATTACAATTACTCCGATAATTGAGAAACTCGCATTGGTAGGATCAATCCAAGTAAGAAACACAGAAGAACGTGCACAAACCTCTTGGTCGTCGGGCCGGCACACGGTCTCGTCAGGTTCCCTGGTGTTCTCGATCGTCCGCGGCACCAGCTTCTCCGGCGGCTGCGAACACCCAAAATGAAAACCATCAGAAAAACCTAAGAAACAAAGCTTGGAGGAATTCGAGAGGGGGAGCTCGCTGCGGAGGGGAGAAATCGAGGCGCGCGCTTGCCTGCTCGCCGAGCTCGACGGCGCGCTGCGCGGCCTGGCCGCGCTCGCGGGCGAGCTTGCGCTTCTGGGCCTTCTTCTCGCGCTTGAGCTTCGCGTGCACCTCCTTGCGCTTGTCCTTGTTCTTGATCTGCGACGGCAGCACCGGGTCGGGCCTCTCCTTCCTCGGCTTCTTCTCCCTCCGGTGGTCCCTCCCCTCGTCGTCGTCCCCCGGCGGCGGCGCGTCGGCGGGCTGCCTCCGCTTCCTCTCCTTCGCCATCGCCGTCGCCGTCGCCGCCGTCGTCTCGGGGGAGGTTGAGGTGTGTGTTCGTTTGGGAGGTGAGGGTTTAAGCGTCTAGGGCAACGTGGCATTTGATGATATAGTGGGCCGGGTTTATAGGCCTGGTTACCTAGATGGGCTTTCCTTTCCAAGATGTGGGCACGGTGCCCATGGGCTGTATGTTGCTTGGTTTGACTATGGAATCAATCCTACAAAATACTCCCTCCATTCCATAATATAAGGCACAACTACTTTTCTTATACTCCCTTCGTTTCAAAATACTTGACACCGTTGACTTTTTAGTACGTGTTTGACCATTCGTCTTATTCAAAAAATTTAAGTAATTATTTATTCTTTTCATATCATTTGATTCATTGTTAAATATACTTTCATATACACATATAGTTTTACATATTTCACAATTTTTTTTGGAATAAGACAAACGGTCAAACATGTGCTAAAAAATCAACGGTGTCAAACATTTTGAAACGGAAGGAGTATTTTATAATATAAGGCATGCATGCATGTAAGCCCTTAACTATGACCATTTCTCTATTAAATTATTACTTTTTAAAATCCTTTACTCTCATGTTCTCTAATTCTATTTGATACATGCATTGTATTTATTAGGATAATAAAACTACGAGATGATAATAATAATTATTTTTTTGTCTTTGGGTTAGAGGTGGTTATGCCTTTATATTTTGGAATGGAGGGAGTATTTGACATTTTTATTAGATTTCTTATTTCTATGACTCCATCTACATCAGATAATAGTCTTCAAAATCGTATTATCCTTTTAGTGCCTTTCCAGGGCACGATGAAATTTACAGAGAGAAATTTGTAGAATTTTGGCCTGAATGGGCAAGATGATTTTGCTGGAGTTTTCTTTTACAACCAGAACTGTATAAAATGGATTGCACTAAACTCTTCTTAAAATCGTCTAAAATGCTTTCAAATAAAACAAAACTAAAAAAAGACGCGAGATAT

>Os06t0192800-01 2000 bp upstream sequence

ACCGCTGCCACAGTAGCAATGGATCCGAGCAACACCGGCCGCGAAGGCGGCGGAAAAGGGCGCCAGCGCCTGTCACAGCCTCCAACACCAACTTGGCCACCGTCAATGAAGATAGAGGCACCAGATCTAGCAGAGAGGAGCCAAGGTCCCGCCGCCGACCACCCCACGCCGTCGTCGACTCGTCCTAGCTCCCAAAGCACAAACGCCGGCCGAGGGCGGCTGGAGGGCCAGATCTGCACCACCGGCCCTCGCAGCACCACGCTTTGCCGCCACCGCCCCCGAGCCGAAGCCCGTTGAAGCCACGGCCGTTGCCATCCTAGATATGGTCACGGGCTCTGACCAGATCGAGGGATTAGGGGGAGAGGAAGGCCTAGTTGCCGCCATTCTTACAGCCATGATGTCTAGAGGCTTGCTCGGGCGGCGGCGAGGTGGAGGTAGGAAGGAGGCGGGGTAGCAGCGAGCTGGCGAGTCACCGTCCGTGTCGCTCCAGCCCTAGGATTCCTTACCATTTTCTTTTGTTTCATCCAAAATTATCACTGTCTAAAATTTAAAATAAAAGTACAAAGAAAGGTGATACATAACTGATTGACAATCACTTAGCCACGCATGCTAGTCGAGAAAAATCGAATGTACCTTACGCACGAAAGGCTAACGACTAGCATCACACAGATGTAACTACCTAGTTTTTACTACCCTCTTGTAGTATTACAATGTCGTGAATGGACTAATAAACACAGTAAATTAACCACCGGCAATCCCCAAGTATTTACAAAGGTATATATATATTTCCTCTCTCAGTGATATATAAGGCTACGTAATTACGTAAGTCACTGAATGTATATTAGAGCGATGAAATAATAGGACACACATATATTATGAAGGTGACAAATTATCAAATATCCCGATTACGGTCAGTAAGGGTCTTTTGCAGTACTGCACAGTAGAAATGCTCGCTGACATGTTGGCCCGTAGAGGATCTGGATTGCATCCTAAGAGCAAGTATAATAGTAGGCTATAAGTCGGCTAAATGCTGATGTGGAGGAGAGAAGAGAGGAGAAGCGGGCTGTAAACTTACAGCGGCTTGGACACAAGAACCAAGAAACTCTGTGAGAAAGACAAGTAGGCCATGTAATAATTATAAAGAGCTAACTATTATATAGGTAGACTAAAAGAAGACTATAAAGAACTTTATAGAATACAGGTCGACAGTATTATTAGCCTTGCTCTAATATTCGTGGACGATTAATTACACTCCGGTTGCAAATTCAGCATGCTGCCCACACACAGTGTCCTGTGTCCTCCCCCGCCCGCCAGTTGGATGCTTCCCGGATCCCGGTGCCCGCCGTCTCATCCGATCCAACGAAGAAAGAAGAGAAAGAAAGCCCGCGAGTTCGAGTCAAACCGGGCGACCCAAACGTTGTCCTCTTTCCTTGAACCCAACCACTTGTTGACAAAGTCCATCTCCCGCCACATTCCACCCCGGATCCTCTACATCTTCAACACCGCAACAACGGTGACACACGAAATGGCTCCATCGTTACAGCATATTTTTGCAAATGAAATATAATTTGTTAATAAAAGGGCGGTTCAGATTGTAGCCAAAATAAAACCTTACTAAACTTTGGTAAGTTAACATTATCCAAATTTTGGCAAGATTTTTTTTATGTATTTACTAAAGTTTGGTAAAAGCTAAATGTGGACATATCTTTGGTAACTTTGCTAGAAATATAGTATGGTTGAAAATATCATCAATATGAAACAGCCCAAAAACTTTTATATACGTGTTTTAGCAATTAAAAAAGGAAATAAATTATGTTAAAAAACCTTAAAATTAAACTCAAAATTTAAATTTAAGGTTAAAAGTTAAAATTTTACGGATGATAAGCATAAGCGAAAAGATGAGACCGGTTGGCTTCGTTGCCACATAAGATCCGGCTACTCTCCCCCACGTAGCCGCCTCGCCTCACCTTCAAATACGAGCTGCCTCCACTGCCTCGCCC

>Os06t0215900-01 2000 bp upstream sequence

AAATAAAAATCCAAAAAAAATAAAATTCACAAATTACCGAACCTTAACCCTAGATCCGCTACTCCCCGCCACCGCCACGCCGGCTGCCACCGCCGCCGCCAGATCCGAGCGGAGGGCAGCCGCCACCGTTAGATCCTGGCGTGGGGAGGGCGGCGACACTCCCGGCTGCCAGATCCCAGCCATCTCCTCCTCATCGTCATCGTCGGCGGCGGCCGCCGCGCTGCCTCCGCCAGCAGCCGCGATGGGCGCCTACTCGTTGGCGCGCAGCTGCGGCGCCGCTCCTGGCCGCCGCTTCCTCCACCTCATCGTCATCGGTGGCGGCCTCCGCGCCACCCTCCTCCCGCTAGACTCCGCGCCGCCGCCCACCGGATCCGCCCTCCCCTCTGGCCGCACCGCCGCGGACGCCCCTCCCCTCCGCCGGATCTAGGGAGGGAAGGAAGGGGAGGGGAGGGGAGCGGATCCCGCGCGGGGGAAGGCCGGCCGCATGCCGGATCCGAGCGGGGATGGCCGCCGCCCGCCGGATCCCGCGCCTCCACCGCCGCCGTGTGGAGCTGAGGCCGCCGCCGCACCGCCTCTGTCACTGCGCACCGACGCGAACCGCTGTCGCTGTCGCCGCACGCCGCCGTCGCCGTCACCCGCGCGCCGAAAGGAGACAAGAGGAGAGACGTGAAAGAGAGAGGGGGAGAGAGAAGAGAGATAAGAGGGGAGGGGTGTGAGGAGAAATTAAAAGAGATATATGAGGGGAAATTTTGAAGTGGGTGGGAGGGAAAAGAAGGAATACTATTTTTGCGTGCGGCTCTCTTAATAGATCCGCACTGGAAAATCGACTCATTTTCCCGTGCGGTCCATTTAGGAGGACCGCACGAGAAAATTGATTTTCCCGTGCGGGCGACAGCTCGGCCCCGGTTCCCCTATTTTTTTGTGCGGTTCCACTTACGAACCGCATGGAAACAAAAGAGGGTAGTGTCCACAAAAATCTATCGTGTAGTAGTGTTCTAACTCAAAGGATGTGATCATTTATTACTTAGGTTGTATTTCTATATATGGAGAACCTGTTAACCAAAACATTCAATTGTTTATCACTATGAACTCTTAAGTTTTAGTTCTTCTAATATTTATAACTCAAAAATTCAAATTGAATAAATATAAAGACGTACCTATCTTGAAATTTAATTCTAAAAATTCGTATTCATAATTAGCAACTCATAACTGGTGTGGACTCTACCTTTAGTATGAGCATTGCTATACGTACGATAAAACTTCATACGATCAGACTACGATACTTCATAGTCTCTTGTGTGTGATGCAAATGTGGGGTCCAACATGTTTCCACCCAATAAAAAAAAAAGACATGCATGGTAGGATCGTACTAAAATCGTACAAATTTTATCGTACGCAAAGCAATTTCCCTTTAGTATAACCTGCTACAATTAAAATTTTATTTATACTATCAATAATAATAGCCCGTGGGTTACTACTACTAGTAAAATTGTATTAGATGCAGTGTTGTATATATTTTATATACATGTTCTTAACGATCTAAGAGAAACAAGCAAAAGATAAAAAATAAACTGCGATGACAAAAAAAAATCTCAAAATCAACTCTACATTTAAGGTTGAAATTTTAATTTTGACTAATAAATATAAATATAAGCGAAAAGGTGAAGCCGATAATGCAAGAAACCTATACGTTAAAAAAAAAAGAACTAACAGCTCAGTGCATCTCTTACTCTTGTTGATTGACACCGGTCATCAGTAGTTCAGCACCAGACGGCTGTTGCATTAAAGAATGTTTAGAAAATTAGAACCACGCATTCCCCCACACGAGGAAAATCAGTGCGCTCTCCCAGGTAAGGAGAGACTTTCCTACCGACCAGACGATGACGGAGAGGGTGACGCAGGAAAGATCCGATGGAATAGCAGTGCGCACATTTCCGTCAAGCCGCCAGGGTCCCACTCAGACGCTATAAATGAAAGAGCATCATCAACCCCCTTGGACC

>Os06t0215900-02 2000 bp upstream sequence

AAATAAAAATCCAAAAAAAATAAAATTCACAAATTACCGAACCTTAACCCTAGATCCGCTACTCCCCGCCACCGCCACGCCGGCTGCCACCGCCGCCGCCAGATCCGAGCGGAGGGCAGCCGCCACCGTTAGATCCTGGCGTGGGGAGGGCGGCGACACTCCCGGCTGCCAGATCCCAGCCATCTCCTCCTCATCGTCATCGTCGGCGGCGGCCGCCGCGCTGCCTCCGCCAGCAGCCGCGATGGGCGCCTACTCGTTGGCGCGCAGCTGCGGCGCCGCTCCTGGCCGCCGCTTCCTCCACCTCATCGTCATCGGTGGCGGCCTCCGCGCCACCCTCCTCCCGCTAGACTCCGCGCCGCCGCCCACCGGATCCGCCCTCCCCTCTGGCCGCACCGCCGCGGACGCCCCTCCCCTCCGCCGGATCTAGGGAGGGAAGGAAGGGGAGGGGAGGGGAGCGGATCCCGCGCGGGGGAAGGCCGGCCGCATGCCGGATCCGAGCGGGGATGGCCGCCGCCCGCCGGATCCCGCGCCTCCACCGCCGCCGTGTGGAGCTGAGGCCGCCGCCGCACCGCCTCTGTCACTGCGCACCGACGCGAACCGCTGTCGCTGTCGCCGCACGCCGCCGTCGCCGTCACCCGCGCGCCGAAAGGAGACAAGAGGAGAGACGTGAAAGAGAGAGGGGGAGAGAGAAGAGAGATAAGAGGGGAGGGGTGTGAGGAGAAATTAAAAGAGATATATGAGGGGAAATTTTGAAGTGGGTGGGAGGGAAAAGAAGGAATACTATTTTTGCGTGCGGCTCTCTTAATAGATCCGCACTGGAAAATCGACTCATTTTCCCGTGCGGTCCATTTAGGAGGACCGCACGAGAAAATTGATTTTCCCGTGCGGGCGACAGCTCGGCCCCGGTTCCCCTATTTTTTTGTGCGGTTCCACTTACGAACCGCATGGAAACAAAAGAGGGTAGTGTCCACAAAAATCTATCGTGTAGTAGTGTTCTAACTCAAAGGATGTGATCATTTATTACTTAGGTTGTATTTCTATATATGGAGAACCTGTTAACCAAAACATTCAATTGTTTATCACTATGAACTCTTAAGTTTTAGTTCTTCTAATATTTATAACTCAAAAATTCAAATTGAATAAATATAAAGACGTACCTATCTTGAAATTTAATTCTAAAAATTCGTATTCATAATTAGCAACTCATAACTGGTGTGGACTCTACCTTTAGTATGAGCATTGCTATACGTACGATAAAACTTCATACGATCAGACTACGATACTTCATAGTCTCTTGTGTGTGATGCAAATGTGGGGTCCAACATGTTTCCACCCAATAAAAAAAAAAGACATGCATGGTAGGATCGTACTAAAATCGTACAAATTTTATCGTACGCAAAGCAATTTCCCTTTAGTATAACCTGCTACAATTAAAATTTTATTTATACTATCAATAATAATAGCCCGTGGGTTACTACTACTAGTAAAATTGTATTAGATGCAGTGTTGTATATATTTTATATACATGTTCTTAACGATCTAAGAGAAACAAGCAAAAGATAAAAAATAAACTGCGATGACAAAAAAAAATCTCAAAATCAACTCTACATTTAAGGTTGAAATTTTAATTTTGACTAATAAATATAAATATAAGCGAAAAGGTGAAGCCGATAATGCAAGAAACCTATACGTTAAAAAAAAAAGAACTAACAGCTCAGTGCATCTCTTACTCTTGTTGATTGACACCGGTCATCAGTAGTTCAGCACCAGACGGCTGTTGCATTAAAGAATGTTTAGAAAATTAGAACCACGCATTCCCCCACACGAGGAAAATCAGTGCGCTCTCCCAGGTAAGGAGAGACTTTCCTACCGACCAGACGATGACGGAGAGGGTGACGCAGGAAAGATCCGATGGAATAGCAGTGCGCACATTTCCGTCAAGCCGCCAGGGTCCCACTCAGACGCTATAAATGAAAGAGCATCATCAACCCCCTTGGACC

>Os06t0275500-01 2000 bp upstream sequence

TCATTTATGCGAATGAATTGCAGAACCAAACTAAAATGGGTTGATCGGAGCTATTCTCTAGAATTTCAAACTTGAGATGACTTGCTTGAAGCGGCTGAGGCTCTTCCTAATGGAAGAAAGGACATCCAATGTTAGGATGGATCTAATTCAAGAATTTCTAGCTCTATACGCGAGGAACGGATGCTGTTTTGACTTTTTTCTTCTTGCTTTAATCCAGTCGCTCGATAGGACTTCCCGTAACCCGAAAAGGTTGGTTCTTCCCCTACAACCCCTACCCCTCCAAGGCAGTACTCTTCATCGGCAAATCCGAATGTCTCGATCCGAGTTGAGACCCACCTACAGAAATTGATCCATCAAAGAATTTCCTGCTGCTATCATATAATACTCATCTCTATTTGGTGATAAATAAACCACCTGTCTCTCTTTTTTTTTCTTTGCTTTCTCAGATATTTTAGATTGAAAGCCATAGTACTAATACCTAAAGCAGTGAACCAAATCCCTACTACAGGCCAAGCAGCCAAGAAGAAGTGTAAAGAACGAGAGTTTTTAAAACTAGCATATTGGAAGATTAATCGGCCAAAATAACCACTTTTTCAAAACCTATCATTAAGGGCGTGACGACGATATACGAGAGGAATAAAAAAAAACTCGTGATTGGTGTGGAGGGGAAGATCTGCTTATGAAATTGTTCAAGAAAGAGTCGTCTCATTTCCTTATATTGAATTCCTTCTATGGTTCCACATGAGAAACCAGTACGAATATAACCTACATGTCCAATCGAATTATGAGCTAGAGGTCTTTTGACTTTCGTTAGATTATCTTGAACATAGGTGCGAGTAAGTTGTATTGTTTGGTCTTTATAGCTACAGTTTGATGGTTTGGTCGTTGCTTCCTCGTTTTCTATTTATTCATCTAATTAATCTCCATTATAACCTACGAGACAAAGTGATTAACTATATGATTAACGAGTAATATGCTGCATATGCATTGGAGCATTGTGGTGTTGTACGCATGGAGCCCGGGCAATTGCCCATGGTGGCCAGGCGCTGCATCCGCCACCAACAGTCGACGTCGCGGCTGATGAGCGTGGCGTGGAGCGCAATGCGGCCGGCTCTCCGGCCCATGAGCTTGGTGAGGCCGACGCCGTTCACTGCGCCCTCGGTCTCCACGTGCCCGGTGGCGATGGCATGCTGCGCCGCGTCCACCGCCGTGTGGAACCCGAACGAGCCGCCGCTGCTGGACCTCACGGTGGATTGTCGCCGCGCCGCGCATCATGTCGTCCCCACCGCCGTTGGGGTGGGACGGAGGAGGAGCAGAGGCGGCGCAGGGGGACGAGGATGCAGGAGGTGGGCCAGCGAGGGAGGAGCCTGGAAGGTGAGGTAGCGGTGGCGGTGAGGGCGGGGAGAGGAAGGCAGCGGCTCGAAGGTGGAGTGCGGTGGTGGCGCGCAAGGTCGCCTCCTACTGCCCACCTCGCTCACCTGCGGGAAGAGAAGATGAAGAGAGGCGGGAGGGTAGAAGAGAGAAGTGAGGATGAAGAAGCAGAGAGAGAAGGGAGAGGGAAGAAGGGAGGTGAGGTTGACATGTGGGGTCCATGTGTGCCTCATCATTTTTAATTATTTTTTAACTGACATGTGGGGCTATGATTTTTATTATTTTTCCGGGATCAAATTGTCACGTAAACGCCACGTGGGATGACGACCAACCTACCACGTGGCGCCACGTCAATCAAAATTACCTTCTAACCCGCCGAGGGAACTTGTTTGCACCGGTTTTGACAGTTCAGGGATCGGTTGTATCTTTTTGGGTTAAGGACAAAAATCGGATTCGGTGTAAAGTTAAGGACGTCAAATGAACTTATTCCAATCAATTGGTATGTCTCATATTTTGGCGCAGAAAACCCAAACTTAGGCCCATATTTTGGTACCTCGGGAATGGGCCGGGTACAGTGTGAACACGTTCCAGCCATATATAACCCCTTCGTCTCCAGTCCCCATCCCCCC

>Os06t0294600-02 2000 bp upstream sequence

ATTACAAATGTGATGCGGAAACTCAGGCACGTATGAGTTGAAATAGATGACTTTTCTTAATCTGATGTTCTATATAGTTCAGGAATTGCAAGAGGATGAGAGAAACTGCTTGGTTAATTGAGGCAAGTTTTAATTTTGTTTTCTTTCGGGACGAAGATAGGCGTCAGCTGCCCATTTTCGTTTGTGATTAAGATTATTGTTTCATGACTCGCTTAAATGTTTTAGCTCAGTTGATAAAATGTTTCAAATTCATGTAACATTTAGAAAATAATGCTTGCAACCCAATGATCCATTACATGAAACATTCAGTGTCAACGACTGATTTTTTTTTAATAAAAATGAATTCAAAATTTAAAATGTGCAATAGAGTCAAATGCTAGTTGAAAGATTTTGCTCTTCCACATAAAAAACATTGAGTTTTAATGGTTGAAATACATAATTTCTTTCATTATAATATAACCAAATGAGATCTTGTTGTAATAATATAATTACAATAAATAGAACAATATAATCGGATATAAGTATAATTTATAAAGAAACATTTGAAGTTTAAAATATTTAATTTTTAGATAATAAAAGTGTTTTACTTCTCCGTATTTTGTCGTAGCTCGCATATCCATAAATTTAGTTTTAAAAAAAGAAAGAAAAAATAAGACATAAGGTGAGAGGACAAATCATCAACTCCTCCTTATTATTGACTAGCTGTCAAGCCACATAGATTTTTCAAAAGCTAAATGGCTGGCGAAAGATTTTTTGGGTCAAAATAATGTAGATTATTGACCTTGGGATTTGCCTCAAGATTCATGCTCCGACGACCTCAGCTCTTCTCCTCTAGCTCCGGCGACAACTTCTCGAACTCTTATGACCCCGATGAAGGCAGTGGGATTAAGGCAGTGGGATTATGGTTTAGGGTCCCGGGTAGAGATGGGGATGGGAGAAAGGGTGGGGGAAGGGGGAAGAGGGGGGATCGGCAGATTTAGAGGGATAGCCGTGGGAGGCGGTAGCCTAGCGGTGGGCGGTGGACACGGGCGGGCAACAAGGCGCCGGAGGTGGCGTCCAAGCCGCTGGGATGGAGGAGCGCGGTGGGCCTACGTTAGCGACGGGGGCAAACCAGATCTTGTGGTTAGGAGGTGGCGGCTGATGGTGGCAGATCGGACAACGGGGAAGACGGTGGGGCGGGGACAGAGGTAGGGGAAGGAGGCCGACCCAGTTAGGGGGGAGGGCTCATTGGTGCTGTTGACCCCTTCCGCCAGTTGACAATGTCGGGCGGCGGCGGAGGCATCATGAGAGGTGGCGTGTGGCGGCACTGGAATGACGAGGGAAGGGAGGGGGTTTGTGTTAGAAATACCACACACAAATCTTAAAGCAATCCAACGATCTAGAATTTGCATGATTTATGAGGTGCTTTTTGTGAAATGCCATCTAGACAGTCCTAAGATTTTTTTAGCCAGCAATTTTAATGGAACTATGCGACTCATAATGGAATAAGAAATCCTGGGTTACCGATTTTCAAGAGTATATCACCATTGTACGTAGTATCCTGTCCCGTTGTGGTAGCATCGTAAACCAAAAGTAAAGCCACTAGCTAGGAAGACATAAAAATATCCTGTATTGGGTTAGACAATTATTTGTCATTATAGCACCGTCAAATTTAAAATATTAAATGAATGCTGATTGCTCCCACGTTGGAAAATTGGGGCTGCGGTCTCTCGCATGGGTCCCTCGTGCATGCACAACTACCCCCGTTCAAGTTGACTAACGACCCCATGCAAGTTGCTGCATGCTTCTGCTCAGTTTGGATAGGAGCGCCCGATTTTTTTCCTAGCAAAATCATTTTTCCTTTCTTTGCCAATGACATAGGAGTATTTGTTAGTATTTTTATGCCTATGAATGCTCATATGACTTTGTTGATTTTACCTGCGGTTGGTAGAGATAATTAGTACAGATCCTTCTCTTTTTCTTCACTATAAATGTGTCGCAGTGCCTGTGTGCTATAGCTT

>Os06t0303800-01 2000 bp upstream sequence

AACAAAATTCGATTGATGAGATGTTGAAATAGTAGATTGCTGTATTAGACTCTGATTGCTGCGATTGTTAATTAGTTTAAATGTTATTGATTTTCTTGGACTTTATTGGATTGGATTAGATAGTGGATTGTGTTTTTTTTTCAGTTTTAGGCCTCTTGTCTCCTCACGTTAAGTCTTGCTAGCATGAAATTTTATAACTAATCGCTGAATCTTCTTCCAGGCTTCAAAATGTTGATGCTGTTTGTGTTGAAGATATTGACTGGGATAACCTTCTTGATCACAGGTGAGTGCTCAAATATTTCTGATCTACCTTCAATCTTGTTTGTTCAGGGAAAAAAACTTCTTAGCCCCCTATAAAGTATTTGTTCTACGATTACTTTACCACCTCCTTCTTTAAAGTATGAAACCAGGTATTCAATCCCCTGAAGTATCAAACCCAGGTCAAATAATCTGCCCTACCCTGTATTTCAATGGAAACCTTGGGCTGCATGTCGCTACTCTTCATGCCTTTTTTTTTTCTCTACATTTGTATAATCGTATTAAGGAATTTAAGTTCACTATGGATGCACAATAGCATGTGCACTAGTTAAATGTGGGTTCTTTCATGCTGACCAGTTTGATTGCACCTGCCAGTCTTATAATGTGGAGACAGTAGTGCAGATCAGCAAGGTTTATTTTTTTGGTAATGAATGCAGATATTAGAGGCTTAGAGTTTTCCCTATAGAGGTACTTGTTACCTATAGTGATCGAAGTTGCATTCGAGGTATAATATTTGACTTGGGACTTGAAGAGAACCATACATTTTGCACTGTTGAGCAATCTATCTTTTTTTTTTCTTTTTGCTGAAAATATATGACGTTCTGTAGGCATTACCAAGAACCTATTTAAACCTTTTGAAATTGGCATGTATTTCCATGTGCGTTGAAGTTTGGTTCCATTTGGTTACAAATTTATAATAACGGAGTATTTCATTCACTCTACTAGGTCTGGAGAGGTTTGCCGTCAACGATGGAACGAGATGGTCCGCTATTTAGGTGGCCACAAGGAGAAGCCCTTCATTGAACAAGTGGAAGTGTTGTCGAAGCGCTACTGCCCAGAAATGGTTGACTATAGGGAAGGGGAAGCGTAGGAAATGTCATCCACCAAAACCTTCCCAATCCGTTGAAAGGATAGCAATAGCCGTCTTAGTTACATCTCGCCTCGTTCAAAGGATAGCATTAGCCGTCTTACATCTCGCCTGTTGTTGAAATGTGTTCTGTGGCCTGTACAAAATGTTTGGCTTTTGGAATGCATGGCACTGATGTTTAGCCGCCCAATGCACTTGGGAATTGTTTGGATAGGCCATCTATGTGCACACGGGCGACGACACTCATCCAGCAACCAGGGCACCCCCTACCCCCTCTCCCCGGGCCATGGTAAGCATGGCTGTGGCGTCCAATCATCTTCGTATGGAAAGTGCTGAACATTACGATTTAAACTATGATGGATGGAGTCATGAACCCTGTAATTTCGTCACACCACAGGTGCATTCTTTGTTGATCTGCCTGCTTTTGTGATCTTCCGTTTTCTTTTTCAGATTTTTGTGACTAAGATCCTACTCATGTTTTAGCCATTCCTTTTCATTATTGTACATGTTAAGTTGCCCAGATACTCTCTCCGTCCTGAGATAGCTATATTTTGGTACTCTCTTCATCGTTCTTAAGATAGATATATTTTGGACTGCAGTGGGAGTATCATGGATTCATGGTGCATCTGATATGAAACACTTCCCATCGTCCCATGGCACATCTGAGATGTCGCATCAATTTACAAAGGCCCTATCCTATTGCCCTTTTGCATGTCCGTTTGCAAAAGGACTCGTACAGTTCTACTAGTGTGCTAAAAAGCTAGTAATGCACTGGAAGCTACCTTCTAAAATATGGGTGCGCTACAAACCAACTTGAACATATCAAAAGCAGATGTAGACTATATAAAGCACAAACCTGCGGACACGGGCACCC

>Os06t0470000-01 2000 bp upstream sequence

GTGTGGCCCAAAACCTATAGCGAGCGGGCACGACAACCAGGAAAAGTGTGTGCAAGTGGGAGAAAAAGGTGGGGAGGGTGGTCGGGTTCTCACTGTGTATAGCAATTGGGAAGCGACACATTAAACTACAAGCGTCAAGGTCGTGGTTTGTGCCATCATGTTCCTGGTGGATGTCGGTTCGGGTTGTGGTCCTCGCTTGCCCCGGCGTTGGCCTTGCAGACCGAGGTTGCGCTTAGCAGGGCAGCTACGACGAGCATGTCGGCACGGTGGTGGTGGGGCAGGCTGAGGTGGGAGATTGCGTGGGAAGTATGGATGTGTTCGACGCGGTGGGTGAGGAGGAAAGGCAAGGCGACGACGCGGCCATCACCTTTATAGGAGGCTTCGGCGCGCGGTCAAGGCATGTAGCGGGGCTGGCGATGTAGTTGTTGGCCGTCACAGCGTCATCCTCGTTCCTCAAGGTTTCGCCGGTGAGGTATGGGACACACATCGGTTGGGCGGACTAAGAACGCACGGAGTGGTGCACGGTGAGCTGGCATGGCGGTGGCTCCCTAGCTAGGGTTAGAGCTCAGCACAACAATTATTGACGGCAATGGGCATGGTTGTTAGGGCGTATTTCGTTGGAGATCTCATCAACAGAAGGAGTAGCATTAGTCCCGGCGACTTACCGGTGGTGGTGGTCAAACTTCGTCGGGGCGTACGGGAACGAGACGAGGGGAGGATCAGCTCAGCTCGGCGGCTCGACTAGCCAGAAAATGCCACAATTGGGCAACTAAGCTGGTCCGGCCTAGCTCGGCTAGGCGAGAAGGAGACTGAAAGGAAGAGAAAAGGGTTGAGGGATTGGATTTAGAATTATAAGGGAGTTCAGGAAGATCAGTTGGGAGGTTTGGGTCTCGGTAGGCTAGGCTAGACCCACCAGGGAGCAAGAATAGACTTTTGCTGTTAATTTCATAAAATAGTTCCTTCATGATTTTGTTTTACATAAAGGTTTTGAAAATAATATTATTTGACTTTCAATGATAAGCGTGGGCAGAAAGAGAAAAATTCGGAAAGAACCAGCGTGATGCAAGTTTTCGCAAAAAACGTTAAAGCCATGATTTTTATTTTCTGAATTTTATTTATGAAAATTTCATGGTGTAACAGGTGGCGAGGGAATTGGGAGAGCGAGTGTGCCTGGACAGGGTAGTTTCTCAAGAGTTCCTCGACATGATAAGTAGTTTGCTTCGGGTTAGATTCAAATCAACCTGATAAGTAGTGTGCTATGATGAGTTTCTCAAGAATTGTTGGGGTACACTTCACTATACGTACATACGTACTTAAAGTATGATTTATCTTTTATATTTGCACTAATTTTTCAAATAAGACAACTAGTCAAACATTGTATGTAGTGACCACTAATTTGGAACGGAGGTAGTACAAAATATTCTACTTCAAATTCGATAGATTTTCACAAAAGTCTTCTTGTGAGAAGATGTACGAGTGATTGATAGACAGGATGTACCTGTTGCTAATTGACGGAATCCTCCCTAACATCGGAGACCCAGCTCAGGCATGGTGGAGTGTCAAAGCATCCGGCCGACTTGTTCCACGCTTCAATTCCATCCATCCCCACGAGCTGTCTACATTCCGATCCCCATCCCGGGTCTCCATCCCAACCAATCCATCATCGAAAATAATAAAATCAAACACAGAGATAGAGAGAGAGAAGATAAAAATTACGAACACGTATGTCCCTACTGTCCAAGATGGAATTAGGTGGGCAATCCTAATTATTGTGCACTTGATCCAGACAAGTCAAACGAAGGTGGGAGGGAGGAAGCAGCTTCGCCGTGCCGAACTACTTCGTACGTACTATTACTAGTATTTCCAATCAACGCCACGCTGTCCCCAACGAACGAAACGAAATATACAACTAGTTTCCAAGGAGAGCAATCATCGCCTACATGTTACACTCGGCTCGTCATGTGCGGATTTAAGCCTTAGAACAAAAACTTGTATATGTT

>Os06t0530600-00 2000 bp upstream sequence

ATTTAGGATTTTTAGGGTGTGACACCACCCTTGGTCAACACCAATTGCCTCTTATCACCCCCATGCCACCTCCACCCCCTGCCCCACACACTGCTATATTCCTAGTGGCCTCTGGCCGCCTCCACCTTGCTGATGGCTATTGTTAGCTCGCTACCGCTGCCTGTGTCTGCATCGTCGTTGGGCTCGCCGCGTTCACCATTTCTTGTTTCAAACCACTCTCCCCCATGTAGATCTAGGGCGTGTTTCATGGAGTTGTCGTGTTCACCATTTCTTGTCGCAAACCACTGGTCCGGTCCGAGAGCCCTCACCGGCTTGGTTCGATAAAAGGACCGCCTATGCAATCTAACCGGTAAAAACCGAACCAACGGATTTTTCCTACTAAATTGCTGATGAAAAACTGCGCTTAGGTTCCGTTCTTTTTTCCAACAAGAGTTGGATGAAGATTAAAATTTTCGTGGCATGCTTTTCAAACTACTAAGTTTGTATTGAACCAGTGGTCTAACCGGTTGAACCAATTAAACCATGAACTAACACTTCGTTGGTTAAATATCAGGTCATGGTTTTTTTAGAAAAACCCTACATATCATGGTTGACCATCAAGTCAATATATTCGAGGTTGCCTTACAATAATACAAGGCTAAGATAACTATTCATTGAAGAATCCGATCCATAAAAGTGTTCACAAGCAATCAATGGTTAAAAACATGTAAAAGTGGCCCAACTACAAATCACGTGCACAACCCCGGCCCTCAACTGGAGATCGATGATGGGGACATCGGTAAATATTTCCACGGTCAAATTCAACATTCCATCCAGAGTGACCAAAACAAACTCACCGTCTCTCTCGATCTCACTCTCACAGGTAGGATACAACCTCAGAATACCTGTAATTTTCCCTCATTTTTGTTCCGACGGAAAGATGTCTGTCTGTAAATTTTTTTTCCGCTATGAGAGAGTTCCCCTGTAATTTTGCAGTACATTTTTATTTTTATCAAAGAAGATAAAATATATTTTGATCGATAATCTGTTCAAAGCAACCTAATTAAAACTCGCACATTTTAGCGTTATGCATGCAGTGATAGAGCCAGAGAGAATTATTTCAAAACCCGATCGAGCAAACTCAACAAAACCTAATATTTGCTACATGATTTCTATCTTCTTTCATACCCTTTCAACAACCAAACTTTCAATGGAGTATTCAACGGCGAGTCTGGGGCTCCGCTCCTGTATGCAAGTTTAGATGTACTTTACGTTTGTCGTAAAATATAAAAAGCTAGTATTGAATGAGACACATTCTTTTACTATGAATTTAGACAAAATACTAGGATGTGTCCAATCTAATACTAACTAGGTTGATATATTATGAGAGGAATGAAGTATCATCCAACGTAACTAAATGATGGTTATGTTGAAAGTAAATACAAATGGACCACAAGCATACGACTTTAAATAACTAACTAACCATAGTGAATTTTTCTAGCACACTTTTGCAAGGAAAAAAAGGGAGACAAGTGGGACAAACTAAATAATGAGTAGATGCTGCTGTTGAAATTAAGCAGCTCGCGGTCAGTCCACAAGTGGAATAAGATGACACGCAGCAGGAAGGAGCAGAGCAGCGCCCCTAGCTCCCTCGCTATCCAATATCGTTGGCGTCCCAAGCGTGAAGAAAGATATCTCGATCGATAGCTATTGCGCACGAGCGCTTGACCGCACCGCACCCATCCGCTGACCGCTCCCCCGTACTCATCCCCCCCTACTACCTTTGACCGGTCAACCCCTCCGTCATACAGTCACTGACGGTGGGGCCCACCGGGCTCTGGTCCCACGTGTCGGTGGGTGGAGGGGGCTTGTGTTCGCCGGCGAGTGGTCGTGGGCGGCTAAACCTATATAAGCCACGGCCGAACTCGTGGCCGTTCCTCAGCAACAGCGAAGGTCATCTAAACCAGCACGTCGGCGACGGCGGCGGCGCAAGAACGAGTCGTACGTGTGCTTCGCCGGCA

>Os06t0681700-01 2000 bp upstream sequence

GAGATGGACCCGGGCAGCTTCCTCACCTTCGACCTGGGCTACTACCGCGGCCTCCTCAGGCGCCGCGGTCTCTTCCAGTCCGACGCCGCCCTCGTCACCGACGCCGCCGCGGAGGCCAACATCGCCAGCGTGGTGTCCAGCCCGCCGGAGGTGTTCTTCCAGGTGTTCGCGAGGTCCATGGCGAAGCTGGGCATGGTCGGCGTCAAGACCGGCTCCGAGGGCGAGATCAGGAAGCACTGCGCCCTCGTCAACGACATCCACTACTAGTATTATCACTAGCTCATCGATCTTCAGCTCCTCGGTGCTTGATAAAATTTCAGTAGAAATATATATACATGTAACTTAATTCGATCGTCATCCTGTTTATTCTCTCTCTCTATGTAATTTTGTTTCCAAATTGTAACTGTTTTATTTTGGAGCAACTGTAACTATTTCTGTATGTGCCCATGAATATATTTCAATTATAGAAAATAATTCACTTTTTAGTCATAGATATTTAATGTTTAGGATAATATTCAGATTAATTATCATTAATATTATATTAAAATAAATTTTAAAATGTTTTGATATATTTACGATTTTACTTTTCTACTAGACAAATTTACGTATATTGATTTTTTAAACTAGAATTTGAGATCCTACTACTAGTTAAAGTTTAAAAAATTAACTAAGTTTTATTATTCTAAAGTTAAATTGCTCCCCTCTTTCTCTCTCTCTCTTAATATCTGACACCATTACATTTTCAACGTACTAATCATTCATCTTACTTAAAATAATATAATAAATTGTCTTATTAAAATTATATAATTATTAATTATCTTGTTATGAATTGTACGTTTAAACATTGAAGATAATCTTAGCATAACTTATATATTCTTGTATTTATTTTACATAATTTAAATAATAAAAAGTAAAACGTATATTTAAAATTCGACGCCATACAATACTGAAAATCAAAGGAGTATTTATGACCATATAATCGTATTTGCACGTGGTCCTCCTTGTCGGCGGCTCGGTGGCTGTCGCCGTCCACGCGCTGCCTTCTCGACAGGGCGGTACTCCACCCGGTAGGTAGGCACCCGTACGGAGCTCGGCTAATCCAGCTGACCATTCAATAATTTTAGATATTTAAATATAAACTTTTGTAGTGAGGCTGTGATCAGTTGTTTTGGCAAAAAAAAAAAATGTATACGACACATATTTAAAGTATTAAACGTAAACTATAAACAAAACAAATTACAGATTCCGCCTGTAAACTGCGAGACAAATATATTAAGCCTAATTAATATGTTATTACTAAATGTTTACTGTAGCACCACATTATTAAATTATGGTGTAATTAGGCTCAAAAGATTTGTCTCGCAATTTGCATGCAAACTGTGCAATTGGTTTTTTTTTGTCCACATTTAATGTTACATGCATGTGTCCAAACATTTGATGTGACGGAAAAGTTGGAAGTTTGAAGGAACTAAACACAGCCTGATTCTCTGAAACTAATAACAGCTTTCAAATGGTCTCCTAAATTCAACTTTAACCATTACTTCTTAAACCAACTAAAAATATAATATTGTGCAATTATTTTGTAAGAAAAAATTGTGGCTTTCATGCATCCAATTTAAGCAATTTACTATATTATGGTAGCTAACGTTTAAAGTTTTTATCAGATAAACAAAACACCATATTTCACTTTGGGCCACATTTTATCACCTATATTTCGCTTTGGATCACATTTCAACTAATGTTTTATTTTGGATCAAGTAATTTTACCTCCATGATGTTTGGACCATCCTGATAACTTTTTTATCCAACTATATCCAATTTATTATATAAAAATAGATTAAAGGATGATCCAAAGCACATATCAATAGTAAAATGTGAAGCAAAGTGAAATTTACTCAAAACGAAAGTTCACTCTGTGAAGTGTCAAATTATCAAATCCCTCCTCCTATTCGTTGTGTTGTAAGACCAGTTTAAAACAATTGATGAAGTGGAGAATTACC

>Os06t0706500-00 2000 bp upstream sequence

CAGTCACTGTGTGGGATGTGGATTTTTATCCCTTGAGAGAACATTTAATTTTATATTAATATTAATTTATGTTAATTTATGTTGTCATTTAATTTTATATTTTATATTAATTTATGTTGTCCATTTTTTTTAATTTTTTTATGACTAATTAGTTGGCATGGATGAAACGAGGGATATTTCCTGGAGGGATGAAATCACTTTCCCCACTGTGTGCTGCCATGAATGCAAATCCAATCAGCTTCATGGCTGTACGTACCTAATCTGTGGTGCAGGGCATGCTCATGGCGGCTCTGTCGGCGCTTCTGCCGCTGCTGATCAAGGACACGTCGTCCATGGCTTCAGCTCAAGTGATCATCCTGTTTCTTGGCCTGTACATGATCGCATTTGGGGTGGGTGGTCTCCGGCCGTGCCTGATGTCCTTCGGCGCCGACCAGTTCGACGACGGCGACACGTCGGAGCGCATCAGCAAGGGCTCCTACTTCAACTGGTACATCTTCACCATGAACTGCGCGTCCGTGATATCCACCACCGCCATGGTGTGGGTGCAAGACCACTACGGGTGGGCATTGGGGTTGGGGATTCCGGCGATGGTCCTCGCCGTCGGGCTCTCCTGCCTTGTCGCCGCGTCTCGGGCGTACAGGTTTCAGACAACCCGCGGTAGCCCGCTCACCAGAGTCTGCCAGGTCGTCGTCGCCGCCGTCCGCAAGTTCAACGTCGCGCCGCCGGCCGACATGGCCCTTCTCTACGACCTATCGGAGGATGCCTCCTCCATGAAGGGAGTTCAGAGGATCGAGCACACCGCCGATCTCCGGTCAGTTCATGTCTCTCGTTGCATCTGGTGTTTGTGATGCTGTAGGTGTAATGTAATTGGCTAATTCTTGAGATGCAACTGCTCGATCAGATTCTTCGACAAGGCCGCCGTCGTGACGGCGTCGGACGAGGAGGCGGAGGGCGCCGCGCCGCGCAATCCATGGAGGCTTTGCGTGGTGACGCAGGTGGAGGAGCTCAAGATTCTCGTCAGGATGCTGCCCCTGTGGGCGTGCGTCGCCTTCTACTACACCGCGACGGCGCAGGCCAATTCGACGTTCGTCGAGCAGGGCATGGCGATGGACACGCGCGTCGGCTCCTTCCACGTCCCGCCGGCATCCCTGGCCACCTTCCAGATCATCACCACGATCGTGTTGATCCCGCTGTACGACCGCGCGTTCGTGCCGGCGGCGAGGAGGCTGACGGGGAGAGAGAAGGGCATCTCCGACCTTCTCAGGATCGGCGGCGGCCTCGCCATGGCCGCGCTCGCCATGGCCGCGGCGGCGCTGGTCGAGACGAGGCGCGCCCGCGCGGCGCACGCCGGGATGGAGCCGACGAGCATCCTGTGGCAGGCGCCGCAGTACGTGCTGGTGGGCGTCGGCGAGCTGCTCGCCACCGTGGGGCAGCTGGACTTCTTCTACAGCCAGGCGCCGCCGGCCATGAAGACGGTGTGCACGGCGCTCGGGTTCATCTCCGTCGCGGCGGGGGAGTACCTGAGCTCGCTCGTCGTGACGGCCGTGTCGTGGGCGACGGCGACCGGCGGCCGGCCGGGGTGGATCCCCGACGACCTCAACGAGGGGCACCTGATCGCTTCTTCTGGATGATGGCTGGGCTCGGTTGCCTCAATCTTGTGGTGTTTACGAGCTGTGCCATGAGGTACAAATCCAGGAAGGCCTGTTGATACTTCTGGGCTTGTTTGGTAGGCTCGAAATTCCGGCCCATGCACTCTGACGGGCCATTATGTATGGGAATAAGTCCATCCGACCTCCCTCATCTCTTGCACTTGGTCGAATCGCATCCCTCAGCCGCAAAAAACTGGGTAAAACGCCTCCTGAATCTCCCAAAACCGGTGCAAATAGACTCCTCGAGTGGTTTGGAAGGTGGTTTCAGCTGACGTGGCACATTGACCGTGATTGACTAGCTGAATCAGCAACTAGGACCCACATGTCATCCTAATATTATCTTCTTCCTCT

>Os07t0106200-02 2000 bp upstream sequence

GGAGGGATTATAATTTAAGGGTGAAGCGGCTATAGAGAGGTCTGGAAATAAGCCGTACCCACCGGCACCACCGGTGGTAGGAGCGAGGTAGCACTTCCCTTGGTTTGACATTTTTCCACTAGAACTTATATATGTTAAAATACTGGTTCCCCCTTCGAAAAAGTTAAAATACTAGTGAACATCAGAACATGTTTTAGACAAACTAGATAATTTTAAGGGTTTGTTTTGGTTGCAGGGCAATGACATGACAACTAAATAGAAGAGTAATTTAGGTAGAATTCGCGGGCTGGGCTACGACCGGTAAACTGTTTTACATATGTATAGGTCATAAGTGAGATGGCATGCATGATGTAGCTAGGATATTTTGTTTTCCTATCAAACAGATAGATATCATTGTCTACATATTTGTTGTTGACTCATATAATATCAAACAGGAATGATAGGTTCATACGCAGGTAAAATTGACGAAAATATGCATCGTCATCTCGAGTGGAGAAAAACAAACACATGCATATATGTACATGTCGCCATCGAACCCCTGCTATAATCTAATCTTCTTGCTTTTCTCAGTACACGCACGCAACTACTATCTAGGAATCGGCAGCTTTAATTTACTGATGCCATCATACCGATCGATAATTATAAGTACTCCCTTTTTAAGCCTTTCCTAGCTACTAGTATATGTCTATATATCGTACGTTATTTTGTCATATATATACTCTGAACTTTTTCTAAGAAACAAGCTAAAAAATCTAGCTAGCTTTTTAAATTTGTTGTGTGTGCTGAATGAAAATAATCACTTCAATGTGAATGAAGGGATAAACACGGCATGCATATGACATATATTATATATTCAGATTTGATTCGGCCGATTTTTCGGACTTTAAACCGCCGGTGGATATATATGAGTCGTAGTACATGTTTCTTTTTTCAATCGATCAATTAAATTTGTCCGTGTGAATGAATTGATCATGTATTGAGCGATGTGTTGATCAAGATCAATTAGCAATCTTCTTTTTCGGAGAAAAGTTAAGGAATCGGGCGGGTTTTGTATGTATATATAACAGTCAAGTTGGGCATGCAATTTGAACGGATGGTTCATGAGTGTGCGTGCATTATACAATTAATTATATTGCGAATATATATATATATGATACTACAAGCATACATGTGAACCAACGGGCAGAAATACGATTGTGGAGTCAAAGCTTGTTACTAGTTTGCGACGCAACTAAGCAAGCAAGCGGCGGCGGCTGGATTGGATTGGATTAGATAATTTCTCCAACTCAGCGTCACAAAAGTACAGCACATTATACCAGCTATAGCAGTATGCAAGTTAAACATGCAATTGCAAGCAGGTTAATTCGATCATATATATGGTCGTCCATGATAGTATAACGTACGGTGAGTCCAATCTGGCCAATCAATCAAGCAGCTGCTGGCTAGCTAGCTTGCACTGACCGGCCATCTCTCAAGCGTTGACTTGATTAATTGCTTCTATGTTTGGCACCCAATTAATTATTTTTTCTAATAAGTTTGAATTATCTGCGCAGATGCCATGATGCATACACACACAACTGGCGCAAATTATAAGATACTCCCACCATTTTATATTATAAGCTGTTTGACTTTTTCTTAATTAAGCTTCTTTATGTTTGATTAAATTTATAAGAAAAAATACTAATATTTTCAATATAAAACAAATATATTATCGAAATATATTTAATATCAAATTTAATGAAATTATTTAATTTGGTATTGCAGATGTTGCTATTTTTTATAAATTTAATCAAACCTAAAGAATTTTAAGTAAGAAAAAACTGAAACGATTTATAATATGAAAATGATGAGTAACTTTATGAGCTACAGAGAGAGAAACGACAGACATAACTAACAATATCATCAATATTAAGAAGATGATGAGATGGCGCGTACGTATTATATCAAGTGGAGTAGCTCAAAGAATCTATAAAAGGCGCAGCCCGTCGTCGAGCCCTCTC

>Os07t0107800-02 2000 bp upstream sequence

AATATTGCTAGTGCTATATTATGACTTCCGTTACTTTTGACCTAAACCAGCAGATATACATACGTCTTCTATCATGACATATTTAGATCGAAACAAAGTTGGTCTATATAAAAGTTTAACTAGAATTTTGGCCACCATAGAAAATAATTTGTTAATTGGTCGATACAAAAGTGCCTTTCTTCTTTTTTTTTTCTTCCTTTTTCCGACCATAGTCGTATATGGATGATAAAAGTGGTAGCTATTGAGTATGACCTGTGGGAGGCCAAATATTGGGTCCTTAAAAGCCAACTATACTCATCAGGTAGTATATAGAATTATTCATAAAAGGCAATTTTTAACTCTGTATAGTATACTGTAGTACTGTAATAAATCAACAAATAATAATAGTACTAGTAGCAGAGTTGGAATAGAAAAATCCACCAAATTAGTGGCTGTACCGAACGTTATTAGCGCTTTAAATGCAAAGGAGTCCCGTTCACGAGGAGGAGAGGAGCATGGAGATACGGAACGCAAACAAATTCATCTTCTCCTCCGTTTCTTCTTCGCTTGTCTCCAAGCTGTGTACTCTACTCCTCTGATGAGTAGAGTGGACTTTGCAGCTATCGAGCTTGGATTCTAGCGCCATCTCCATCTCCACCTCCACATCCCTACTAGTATACATCATTGCTGCTGCTCTGCCATCGTCGCGCGCACACACGCAGACAAGAAAGCTGGACCAAACGGGCCCCGCCAGCAATGGTGGTGCCGCCGCCGCCGCCTTCCCTTCTCCCCCTCTCCCTACACCCCGCCTCTGCCCGTGAGGCCAAGTCCATGGCGAGACGCGCGCCCCTACGCTGTCTCTTCCTCTCCCTCGTCGCGCTCTTCGCTTTGCTGCCATTCCCGCCCGCTGCGGCGGCGCCATGCCACCCGGAAGACCTCCTCGCGCTGCGGGCATTTGCGGGGAATCTCTCTGCGGGTGGGGGCGGCGCCGGACTCCGCGCCGCGTGGTCGGGTGACGCCTGCTGCGCCTGGGACGGCGTCGCCTGCGACGCCGCCGCCCGAGTCACGGCGCTGCGCCTCCCCGGGCGAGGTCTCGAGGGGCCCATCCCGCCCTCCCTCGCCGCCCTCGCGCGCCTCCAGGACCTCGACCTCAGCCACAACGCGCTCACCGGCGGCATCTCCGCCCTCCTCGCCGCCGTCTCCCTCCGCACCGCCAACCTCTCCTCCAACCTGCTCAACGACACGCTCCTCGACCTCGCCGCGCTGCCGCACCTGTCCGCGTTCAACGCCAGCAACAACTCGCTGTCCGGCGCGCTCGCCCCCGACCTCTGCGCCGGCGCGCCGGCGCTGCGGGTGCTCGACCTCTCCGCCAACCTCCTCGCCGGGACGCTCTCGCCGTCCCCGTCGCCGCCGCCCTGCGCCGCCACGCTCCAGGAGCTCTACCTCGCATCCAATTCCTTCCATGGCGCCCTACCGCCCACGCTCTTCGGCCTCGCCGCGCTGCAGAAGCTCTCCCTCGCCTCCAATGGCCTCACCGGCCAGGTCAGCAGCCGCCTCCGCGGCCTCACAAACCTCACCTCTCTCGATTTGTCCGTGAACCGCTTCACCGGCCACCTCCCCGACGTGTTCGCCGACCTCACGTCGCTGCAGCATCTCACCGCGCACTCCAATGGCTTCTCCGGCTTGCTGCCGCGCTCGCTCTCGTCGCTCTCATCTCTCCGTGACCTCAACCTCCGGAACAACTCCTTTTCCGGCCCAATTGCTCGTGTCAACTTCTCCTCCATGCCGTTCCTCGTGTCCATTGACCTTGCCACTAACCACTTGAATGGCTCTCTCCCGCTTAGCCTCGCCGATTGCGGCGACCTCAAGTCACTCAGCATTGCCAAGAACAGCCTCACCGGCCAATTGCCCGAGGAGTACGGCCGCCTCGGCTCGCTCTCCGTGCTCTCGCTGTCCAACAACACCATGCGCAACATCTCGGGGGCGCTCACCGTGCTCCGCGCCTGCAAGAACCTCACCAC

>Os07t0224000-00 2000 bp upstream sequence

AAAAACATATATGTGGGACCCATTTGTCATTCACACACACAAGATTGTGGGGCCCACTGACATGTGGGCCCCACCTGCCATCTCTTCCCTTTCTTCTTCCTCCCGAACTTCCCCTCCTCCTCCCTTCTCTCTTTCTGTTCTCCCTCTCCACTTCAGGCGTCTGCGGGGAGCGAGCAGCGGTGGGCGGGGTCTGAAGCGACGCGGCCGGCGGGAGCTAAAGCGGCAGCGGCGGTGGCGTCGGGAGCTAGCTGGAGCGGCGGCGGCGGCGGCGGTGGGCTGGAGTTGGCAGCGTGGGGCGTGGGCCGGCGAGCTAGAGCTACGAGAGAGAGGATGGCAGCGCTAACGTCGACAAGATCGAGCGGGGGGACGGTGGTCTACGGGCTGGTTACGGCTTCGGATCCATCCGCATCGCCGGGATCAGAGCTCCTCGTCGCCGTCCTCCACCATCTCCCCGCATCCATCCTCACATCGCGCGGGATGTCGACGGCGGCGGTGGGCGCGGGACGTCGACGGCGGAGGCATCGAGCGGGTCGAGCGGTGGCTCCGGCGGCGTGCGGGCGAGCGCGAGTGCGGGAGGAGGCACCGGCGGCGACAACATGAGCTCGGCGGCTCCGCTTCTCCCCTCTCCCTTCCGGCCCTCACGCGCCGCCGCCGCCTGTCGTGTAACCCACCACCTCCGTTCCTCCCTCACCTGCTCTCCTCTCCTCTCCTCTCTCCACTTGGCAAGGCTGATGGCAAGGCTACCGCCGCTGCCGCTCCAGCTCCAGCCGCCCGTGCTCCCCCGCGGCCAGCCAAAGGGGAGAGAAGAGAAAGAGAAAGAAAGAGAGAAGGGAGGAAGAAGAAAGGGAGAGAGAGGATGACATATGGGGCCCACATGTCAGTGGGCCCCACAATTTTTTATTTTGTGTGTGAATGACTAATGGGTCCCATGCATATTTTTTTTAATGCCACCTAAGCGCCACGTCAACGCCACGCGGAAAGAAGACCGAGTCAATACTGCCACGTCAGCAAAACTGCCATCCAAAACCACCAAGGGAGTTAAATTGCACCGGTTTCAATAGTTTGGGGGTGAAGATATCCGGTTTTGCGGTTTAGGGTCATGAATTAGATTCGAGTCACTTTTGAGGGTTATGAAGTGAACTTATTCCGAGTACAGAAGAAAAGCCGACGGCCCACTTTGACCAAGGCCCGACAAACCAGCCCGGCCCATTATCATCACACCAACTCATCCACCCCGTCCATTCCGTCCCCCCACCGCATCGGACGGCTCCGATCCATCCCGCCGCGCGCTTGCCCTACTATAAAAGGGAGCACCACCTCCCACGCGCGCCGCAGCGGAGGCGGACGCGGAGGCGCTAGGGTTTTGCTGCTCCCACAGGTGAGTCGAGCTCCTCTTCCCCCCATGGATTTGCTTCGCTGCTCGCTGCCATGGCGTTGCTGCGCCGTCTCGTGTATGTAGCGTATTCGCTGTTTCGAGTCCCGCGCTTGTTTCTTGGTAGGTTTTGCGGGCTGTTGTGTGATGGCGGCGCGGCGGCTAGGTTTGTTCGCGTTCTCGACGCGTATGATTGGATCGAGGCGTTTGATTAGATGTGGGGCTTGTTGTTTTGGTGGTTGAGGTGATGCTGTGATGGGGAATGGGGGTTTCAGCTGGATTGGAGTTGTGGTGGTGGCGTTGTCCGTGCATCCGAATGCTGCAAATGTGTTTTAGATTTCCTTTGATTAGTTTTTGGGCACTGATTTGGAATGGCAACTTAGTGGAATCGCATTTGCTTTATGCGTCATTTCTTGTACTCGTTTGATGGATAGAGAAATGCTAGAATTTTGTTAATTTAGATTGCTATATATTGGGGTCTGGTAGTGTTGGGCTGAAGAGGAGAGATCTTTGTGGTCTGTACATTGCAATGCATTTATATTCTGCTCTAGCGATGATTCCTGACGTTGTAATTTGCAGGTAAGGAGGCATTGAAGGTTTGGTGAGGCAGCGAAGATGGTTCTCAAGT

>Os07t0511000-01 2000 bp upstream sequence

TTTTAAAAACAGGGATCCGATAATGAACTGAGTATATGTTACTGTCATGTTTACATCAATGGCATTGTTTTCTTGTTCGCTTGATATCTTGGTTATGTATTTTTAAGCTACGATAAGAAGTCTGTATCTTGGGGGTGCTGATCTTAGGATAAGAATAGCCTGGCTGTCAAAAAAAAATGTAATCATCATGATTAATAGTAATTATCTAGAAAGAACTGATTAATGACCACACGTCTACACCTAACAACACATGGTTGACCAAGTGCTAGTACTGCGTGTGCAGAGAAGAGGACCAAGTAGCTGTACTGAGGAGTGAAGAAAGCGTATACTAGCACAACTGGGCTTTATAAAAACAGATTTTGCAGAAGTGAAGAAATGAATATAGCGCCTCTAGCAGCCGCCGCCGGCCGCCGGCAGCCTGTATCCTCACACACCAGGGTTTGTAGATCCTTCCAAACAAAGAGTTACCTTCTTGTTTGAACTCTATGACATTTAGCGGTGACAAGGAACTTCAGGACAAACGAAAGCATGCACACTTTCTAGGTACTGAGGATTCTATACCAATGCTATGGGATGCCATACAGATGCTAGAACATATATAAAAGTACTATTTTAATTGAAAATGCTACACACATATATAAGTAAATTCTTAAAAACTCTTTAAAAAGCTGATGTGGGACAATACAACAGCATCTTAACTTTCTATAACTACTTTGTGGATTAAATCAAACAACCGAGATATTTGTTAGTCTAAAAGTTATGCAGGAGCGTTTTAGTGCATATAGCATGGAAAGTGTTTTTTATTCTCGAGAAGATATCCCCTCTATTTTAAATGTCATCTAAATAGTTATAATATTTTTTTAAAAAAAGTGACTAGATAGATTAATGAAATATATATTACTATGCACCCACGTAAGTTCAAATTCGACTTTCACAAGTTGTAAAAAGTGAATTTACATAACTAATACTTTAATTTTAATTTGTTTATTTATAACTTGTAGAGGTTGAATTTGACCTTTTTTTAAGTGATATATTTATAAAAAGTTTATTTTTTATGATAATTTAGATATCATGCAAGAAAGTAGGGAATGTTTCTCGAGAAAAAACCACCTGCCATATGGCATTGCTCTTTTTTTATATCTTCATTTTTCGTCTTTAAGACGGATTCACACTTAATAAAAAGAAATCCTTCACTACCACTTTCTTCAATCCCATTTCTATAGTACCAATTTACCACTCATAGTTACTAGCAATAGTGTTAAAAACAGGTGTTTCTTTTCTTTAGGCTAGCCATTACTCTTATTATCTTGCACATCACAAAGTTTGGGTAATAATTTTATTTTTCATAAGTCTGTGGTGACGCCTTTTGAGGATAAGTACGTCGTGACATCTATAATATCTGGTGTTAATGTTTTCATCATGCCTAGTGACGGAAACTATATATGGAGCTAAAAATAGGTAAGTAGCAAAAGCCCCATATTATTCAGTTCATGTTTGGGTAAGAATAAAACATAGCTCAATACCATTATGACATCACCTTGGTCTCGTCCTCCTTTAGTTTAGAGATAAACCCTACCTGCAAGCAGTAACTGCTCTAGTGACGGAACAACGACAACAAAAGTTTAGTCTTGTGGCGACACTCCATCTCCTCCAACCCTGTTAGATAATGATATGCTGAAGAGAATATTGACCAATGTTAAAGCAATACAAGTAGGGAATATATAAAATAGGTAATGTATATATCGCCTACAATTTCTACTTCAAAGACGCGTATACGGGTGATTTAAGATCGACACCTAGTTTTAGTGTGAGGGAGATTCTAGCATCCCAAAACCCAGCAACGCTCCCTCCATCAACTCTCACAGGCTCTCCGACCATCCAACGGCCGAAACCGACCGAATCCAACGGCCCCTATTTTTTCCCCACCGCCACCACCACCTCTCCACTATCCAACACATCCCCTTCTTTTTTTTAAACAAAAAATTCCAAATCGCAACCTC

>Os07t0539700-01 2000 bp upstream sequence

ATCCAATTGTCACGAGGCCACTATTGCTAGATCTAGCCGCCACCGTTAGATCCGGCTGTTGCAAAGCTTCCGACCGTGGAGGAATTGAGGCGCTCGCGATAGTGGAGGAGGAAGAGCTGAGGCACGCAGTCACCGCAGTAGTCGTCGTCGCTCCCGAGGCCGACGACGGTGGATGCGCCCCTCCCGGGACAGGGGATGGTAGATTAGCCACTTTACCACCCTCGCGCGAGTCGCGATATGCCATTGTCATCATTTCCCTACCCACCACTGTCGTCGTCGTTGTCGCCCTGTCTACTGGCGTCAGATCTGACCATCACAGGGCCATTCCCGTCGCCGCTGTCGTCACGACCGTTGTCATCGCTCCCATGGATAGGCGGTGGGGGAAGGGGGACGGGTGCTAGAGGAGCGGGTGAGGAGAGGAGAATGGATGGACACACGAGTGTGGAGTGAGGAGATTTTTTTAAAAAAGATTAGAGAGAAAAAGAGATGAAGAGGGAAGTAGGGAACGGGGCAGCTGGCTTGGTGAGACTGACATGCAAAAATTGATTTTTACATACGAGTGTGTTGTCTTCCTCCCGGGCCGATAATATTTTTTTTGAACATACGGGCCGATAATATTATCACAGACGGATGATGTCATAGCCGAGTAGCAGACCGGTAAGCTAGAGAGGGGCCCCTATGCGAAAATCGTTAATGTGGTAGTGACGCTTCCTCCCACATTCATTCCTTTCCAATAGACACGTCAAATGCCGCGCGCTCCTCTGACCGTGTGTCCAAGCTTCATGCTAATCAAATCAATCGATGCTCCCTTTTTTTTTCCCTCAAAAAAAAATCGATGCTTCCTTTTTCCAGCGAGCGATCTGCATGCGCATGGCTTTCCAGATTCCATACCGGATCAAGGTTCCTGAGCAACGGATTGAGATCGGGTTACACTTGTAACTGCAAGATAAAACTGCACACAATTTTGGCAATCTATTTTTAGGATCAACGGTTCAGATCGGAGCTACAGTATTTTTTTCTATAGTACTATGCGCGGTACAGCATGCAATCTGGACCATCCAATTTGCATCCAATGACATGCACTACTGCTGCTTAAGTTGCAGTCACAGTAGTGACTGCAGCTGACCCGAATCCCTGAGCAACATCACCTATGCCTTTGCTCTTGTCACCATCGGCCGGAAAACTCGTGGAAATTAAAACCATTGAGTTAGGCTCTGATAATCAGATGTTGTGGCTGGAAAGTACGCACGCGCGGGGTCTAAAGTCTGAAAGGCGGCGACTACAAAATCATCAATCGATCACATTGCCTTTTTCATTGAAACCATATTATGCTGTCATATCCTCGAGTATTCGTGTACCAATATACTCCGGAAAAGCTAAAAATACCGCGACAATGACATCTGAGTCAATGACTCGTGATAACTAAGTACAGAAAAATTGGCACATGATTTTCCAAGGCCATGCATGCTTTGCTGCCACACGGCCGCCCAAGCAGGAAAGGCAAGCACATCCTCTATCTTTCCCTAGTGTAACAAAGCGATGCAAGTTGTTGCTTTTCCCCGGGAAAGTGATTATATTTCATCAATAGATATCCCCTCGTTTCGTACATACCACCTAAATAGTCACTAAAAATTTTTTAAAAAATTTTTAACATAGTTTAATATGAAATATATCACTCTACAAACATGCAAGTTTAAATTCAACTTCTACAAGTTGTAACAAAAATAACAAATATAGCTGCGAATGTACAATAACTATTTTTAGTTCAATTTGTTCTTTTTGTTGCAACATGTAAAAGTTGAATTTGGTATTATATGTTTGTGGAGTGATATATTTCATATTAATCTATATATGTTGTCATTTCTTTAAAATTTTTTGATAATTATTTAGATGACATGCAATCAACGAGGGATATCCCCTCGTGGAAATCCACATCCCTTTTCCCCACGTAGTGCGGCAGGCTAAATCATATAGCGGCAAATGGTTGAATGCAACTTCAA

>Os07t0567700-03 2000 bp upstream sequence

TTCCTGTGGCGACATGCATCGCACAGTGACGAGCCAGTCAGTACCCAGTACAGAAACCGCGCGGCTCACGGGGGAAAACCCACCAATCGCGACATCATCAACGACGCTGGTAAAAAACCCAGTACAAGTACACAACCTTCTCACATCGCAGGAGATCAACCAGACAACCAACCAGCCGGGCCAGCGCTCTACAGACTAGTACAGACTACAGAGATCGGCAAGGCTGCATGCTGGAACCAGAGACGCTGACGCCCCACTCCACCACCGATCGATCGCTGGTATCCGAGGGGGAGGTTTTGTCCGTCCCCTCGTCCCCCGCACTGTTCGCGCTCGCTCGCCCGCTGCTCTGCTCGTCTCTTCCACCATCAAATGATCACATGCTGTCCATCAACTCACCCCCGTCGTCTCGTGTGCGTGCAGCTTTCCCCCACGGCCTCCTGCTGCTGCCGACCCGACCCGACCTAGCGTCCCTCGCCATCGCGCGATCGTGAGAAAAATCGGGTGTTATTGTGTAGTAGAAAAAAAAACCGGTCCCGTCCTTTGATTCCCTCTGCCTGGGCGCCGCCCGCTACGGGACGCGAGAGCAGCTACCAGCAACCCGACAAACCGCACAGGCGGCCTCCCCCTCCCGCGCGCGCCAGCGTCCAACGCTAACGGCGACCCGCGCCTCTCTCACAGTGTCTCTCCCGCTAGCGTTATCATCATCAGCTGCGGGCTTGCTATGCTATGCTTGCTTGTTGCAGTGGTTTTGCCACGGAGAAGGGTCACCGGGAAATTATTGTTTAGGCTGACGTGATGCTCCAGGGAGTCCTGTCGCGAGCTCCCGGCGCCGACGCGGCAGCGATGAAGGCGAAGCGCGCGGCCGACGACGAGGAGGAAGGCGGCGAGCGGGAGCGCGCGCGTGGGAAGCGGCTGGCTGCTGAGGGGAAGCAAGGGTTAGTGGTGGTGAGTACGGGGGAGGAGGAGGAGGCGGCGGCGGAGACGCGTGGGCTGCGGCTGCTTAGTTTGTTGCTGAGGTGTGCGGAGGCGGTGGCGATGGACCAGCTGCCGGAGGCGCGGGACCTGCTGCCGGAGATCGCCGAGCTGGCGTCGCCGTTCGGGTCGTCGCCCGAGCGCGTCGCGGCCTACTTCGGGGACGCGCTGTGCGCGCGCGTGCTGAGCTCCTACCTGGGGGCCTACTCGCCGCTGGCGCTCCGCCCGCTCGCCGCCGCGCAGAGCCGCCGCATCTCCGGCGCGTTCCAGGCGTACAACGCGCTGTCGCCGCTCGTCAAGTTCTCGCACTTCACGGCCAACCAGGCCATCTTCCAGGCGCTCGACGGCGAGGACCGCGTCCACGTGATCGACCTCGACATCATGCAGGGGCTGCAGTGGCCGGGCCTCTTCCACATCCTCGCCTCCCGCCCCACCAAGCCGCGCTCGCTCCGGATCACCGGCCTCGGCGCGTCGCTCGACGTCCTCGAGGCCACCGGCCGCCGCCTCGCCGACTTCGCCGCGTCGCTCGGCTTGCCCTTCGAGTTCCGGCCCATCGAGGGGAAGATCGGGCACGTCGCCGACGCCGCCGCGCTCCTCGGCCCGCGCCACCACGGGGAGGCCACCGTTGTGCACTGGATGCACCACTGCCTCTACGACGTGACGGGCTCCGACGCCGGCACGGTGCGCCTGCTCAAGAGCCTCCGGCCGAAGCTGATCACCATCGTGGAGCAGGACCTCGGCCACAGCGGCGACTTCCTGGGCCGCTTCGTGGAGGCGCTGCACTACTACTCGGCGCTGTTCGACGCGCTGGGCGACGGCGCGGGGGCCGCCGAGGAGGAGGCGGCGGAGCGGCACGCGGTGGAGCGTCAGCTCCTCGGCGCGGAGATACGGAACATCGTCGCCGTCGGGGGCCCCAAGCGCACCGGCGAGGTGCGCGTCGAGCGGTGGGGCGACGAGCTGCGGCGAGCGGGGTTCCGGCCGGTGACCCTGGCCGGCAGCCCCGCCGCGCAGGCGAGGCTGCTTCTTGG

>Os07t0568600-02 2000 bp upstream sequence

AGTCCTATTCAATCACTTCATGAAAACTTTGTTCAAAGTCATTTTCCTCATAAACAACGCCACACCAGGACCACAAAAAAACAAAAATATCAGATGTGGGGGGGGGGGGGGGGGGGGATGGGGGGAAACCAGAAAACAAGATCATCAGACGAGATAAAGAGGAAAACGAGAGAAGCAACACAATCAACATAGTCCCTTCTTAAACAGTTATCAATTTAAGACTTTTTAGGTAAACATCAAAATCTTAACCAATTAATGCATGAAAATATATTAAAATGTAGGGATAAGCCTATATTACATCACTCAGTTATCGGGTTTGTTTAAAAACTATGCAACCGGATATTGTACACTCCTAACTAATAAAAAGGGCAAAATACCTCTCCCAAGGACAATACAACTTGATTTTGATGGTGGTTTCATGATAAGAGAAAAACAAAATACATGTTTTTATTTATATTGATTTTTTTACATATCTTACTAATTGGGTGCGATACAAATAAGAAAAAAAATTATTTGGATGTCAAAATGTTTTTAAAAAATCACCTCCAAAACCGAATTGTATTACTCTTAGAGGGTATTTTCTCATATCTTAATACTTGAGTGGCGTAAGTGTAAATTGTCTAATTTCATAGTAGAGTGACATAAAGTGTCCATTTTTATAGTTGAGTGGTGTTTTTAGATAAATCCGACAGTTATAAAAGGTATAATATAGACTCATCCCAAAGCTTTGGTACGCAAAAATGTCATTAATAATTTCTTAAAAGCTTTTCTTACATACATTTTAATAATCTTTATTAATAAAAAACAATAATCAACACACACATTACCAATGCCCTAAACAACTACTTTAACATAAAACCATTGGTACACACACCATAGCACAAGCAAGGGGAACAGAGGGGAAAATAAACACGAGAGGAAAAGGAAGAGCTTAAAAAAAGAATTAATTAAATATTTGCCACTTACAAGTGGTGTTAAATTATTTATCACTGGACCCAAATGTCATAGAGACACGACGGTTCACATGTCATATAAAACGAGTGACAAATAATTAATTGCTACAGCTTAAAAGTTGCAATAAGTTAAATGTCCCTTAAAAAAATGAGAGGAAAAGGAAAAAAAAGTAAAGAAAAAGAAAAAAACTAGGGAGAAGAGAAGAGGGCAAAGGATGCAAAAAGGAGAGCCGAGAGAGCCGAGTGAGAGAGGGAGATCTTTGACCAGCACCCCCGTCTTCTCCTACCTTCCTCCTCCCCTCCTCCGCGGCCTCGTCGTTGGCTTTACCACTGGCGGATTCCCACACGCCCCCCAGCCGCCGCCACGGCTGACCCCGTCTCCCCACCGAATCCACCCCACCTCCCAATTTAACCCCCTCCTCCTCCTCCTCCCCCCTCTCGCCTCCTTCTCCTCCCTCCCCCAACGCGTCACGCCGCCATTTCTTGGCCTCCCACTCGCCGCAGATCTTTGATTCGCGGGGGGTTCTTGCGTCGCGCGGATCTTGGCTGAAGCGAGATGGGCAACTGCTGCGTGACGCCGGAGGGCAGCGGCCGCGGGAGGAAGAAACAGCAGCAGGAGCAGAAGCAGAAGCAGAAGGAGCCGAAGCAGCAGCAGCAGCAGCAGAAGAAGGGGAAGAAGCCGAACCCGTTCTCGATCGAGTACAACCGGTCGTCGGCGCCGTCGGGGCACAGGCTGGTGGTGCTGCGCGAGCCGACGGGGCGGGACATCGCCGCGCGGTACGAGCTGGGCGGGGAGCTCGGGCGCGGCGAGTTCGGGGTCACCTACCTCTGCACGGAGCGGGAGACGGGGGACGCCTACGCGTGCAAGTCCATCTCCAAGAAGAAGCTCCGCACCGCCGTGGACATCGAGGACGTGCGCCGGGAGGTGGACATCATGCGCCACCTCCCCAAGCACCCCAACATCGTCACGCTCCGGGACACCTACGAGGACGACAATGCCGTCCACCTCGTCATGGAGCTCTGCGAGGGCGGGGAGCTCTTCGACCG

>Os07t0571600-00 2000 bp upstream sequence

GATCTCAACAGTTTTGTCAATGACCATGGCAAAATGGCATTTTCTACAAGTGTAGACACATTCAATGGCAGTATGGCACAGTATTCACAGGACAAATTGTCCAAAAGATGAACTTCCAAATGAAACTGCATTGTAGAATCGGTTGTTTCGGCTTCTTGTAGGAGAATTTACTATTTATCATCCTCCCAAAATGCATATGCACAAATACCGCTCTCTCAAATTTTCATGCACATTTGCCACTGAACTCACATGTCAGCCTCATCCTCCATTCGGACAAATGAGATATGGGATCGGGTGCACATTTTGGGAGAATGGCAAATAGTAAATTCTCCCTCTCGTTGTAATGCGATTGCCCATGCATGTTCTTGGAAAAAGAAGCAACTACAACATCTGCATTTGCAGATATGTTACTAGTATTAAGCAGTTGCAGGTGGGTACATTAAGGTTTGCAAAATGCACACGGGAAGAAATAAGCAGATCAACCAGGACTCCATTGGCCCCCTACCCAGATCAACATTATAATTGATTAAGGGACTACATATATACTCCCTCCATCCCAAAATGTTTGACACCACTGACTTTTTTAAAAATGTTTGACCGTTCGTCTTATTCAAAAGATTTAAGTAATTATTAATTCTTTTTCTATCATTTGATTTATTGTTAAATATAATTTTATACATACATATAGTTTTTACACATTTGATAAAAGTTTTTAAATAAGACGAACGGTCAAACATATTTAAAAAAGTCAACGACGTCAAATATTTAGGGAAGGAGGGAGTATATATATACGCCGTCCAAATTAGTCAACATATAGCTCTCGTTTGGTAAGGAATGGAATGACAATAGTATTCATAATACCTGATAACTTCCTGGCTTTCGACAAACATAACATAGTACTTGTCCAGATCGGGATTTCATCATCTATCCCTCATAGTCACAGTCTGACAGAACAGATCACTCGTTAGATCGAACAAGAAATGAAGCTCCAACATGATCGAGGAGCAGCATGCGGGTGTACATGATGACCATCTTCTTGGCTGTGCTCTTTGGATCCCTTGCAACGCCTGCACTATGTAAACAAACACACACATTTTACCATCTAAATTACTGTCGTATGGGAACCCAGCTTTCGATTGAAATTTAGAATATTTTTCTCACTCTAGATCTAAGTTAAAAATTTAGGGTTACATAGTGATCAATGAATGCGTCATGAATGGATTATTTATGTCACTAAAAGTATAGCAGCAAGTCATTCTCATCAGTTCAGCACAGGAAACAACTCTGCAAAATGGCCACACCAACAGTGTAGTTTTCTGATGTAGCCGCCGCCGTGGTCACCGACTCTGGCACCCGCAGCACATGACGTCGGATGAAAATTTCTCGATCTTGTGCGTGGAAGCAGGTTGTCCACCGAACAGGAGCAACCCGTCGCCACAATGCTGCTACTGCTTGATAGGACCCCATAAACCTCGGTGGAGGTTTGTGGACTGCAATACTTGCCTACGATGCAACCCCAAGGTGTGCCTGCTGATGGCTTCGTCTCCGGTGCCATGACGAGATGGGAGGACAACAACCGTGATGACTGCTACGAGCATGAATTAATGCTTCTAGTTACTCTCTCTATGGATCATGTAGGCAAATAAAAACTAAGGTTTGTTTCTTTATCTTGTTAATGGATAATGAGGCCCCCTAATATCAATATTAAGTGGAAAACCCGAGAAAATCATCATAAAAGAAAGACATTTTGGCCTTTTTTTTTTAGATATTTTATGCAATTTCTTTTAGAATCTTTCAAGCAGTTTAGATTGGATACATATCAAAATTTATCTTGCCACGTTGGTTTAATATTTAACCATTCCTTATATTCTGCTACGTCTTGGATAACATAAAACTTTAGTTTTAAAAGAAAAATCTTTTGAAAGCATCGTTCAAAAGATTTACCCCTACTATTTTTTTAAGCTGTACAAAGAGAGAATTCAGAGGAACTACTGAAAAAA

>Os07t0575600-01 2000 bp upstream sequence

TTCTAAAACTTGGGCTGCTCTGCTCGCATCCATTGCCCAATGCTAGGCCAACAATGCGGCAGGTCGCTCAGTACCTCGACGGTGATATGGCCCTACCGGATTTGTCACCGACGTACCTAAGCTTCACTTCACTAGAGCGGATGTACAAGGAGTTCAACCGCAATTCCATTTCATACATCTCATCAGCAAGCATGGGTGCCATTTCTGATATCTCGGGAGGAAGGTGACAAAGGAAGGGAGCTCGTGATTTTGAGAAGCAGTTGGTGAGGATATGAAAAATCGGGTTTCTCAGTTTATTACTTCTAGTTTATTTTCTGAATTATAATGGACTGTTTAGGGGGCTAAAAATTCAAAAAAAAAAGGCACACATCAGATACTCCCAAAGTAGGGCCAAAAATGGTGCTTGTAGCATTGCAGTTAAGAGTATGTTAACGGTGTAACTAGATGCTTTAACTATCTTGTTCTCCTTGTACTATGAAATCGAGATTCTTCTGTCTTGATTATGTTCATAGTTTGATACAGCATCGGGATGCACTAAACAGTTAAGTAGCAATCTTGAAAAGTATCAAAAGGTTTTTTTCTAAAAAAAAAGTAATACCTAGTTTTATAATAGAAAGTACATAACCAAGAGTATAAAACTACTCTTGATAGAAAGAAAAACTAGCGCTGCTATTTTTCTTTGGCGAACACGCAAAAAGACTTGCGCATCAATAACACAATATGTTATTGGAAGGAAGCAGATTGTTTACCCACCGCTACTGACTGGTAGCCATCGCCGAAGGAGGAGGAGGTAACGGCTGTCGGGCTATTACAACTAACTAGTACTGCCAATACAAATATTCATGCGGACTCTTGCACTCTACAGTAGGACTTGTCATGATTAATCACAAGTTTTACGTGCATCTACAAAAATTTCCTGTCAAGAGAAAGCAAGGTGCTAGTTTTCATGGATGTAGTCTTTAGGATCTAGCTAATCCGATTGGTGGTGTTGTGCAGCAGCTGCTTCTGTTTCTTTCATGAACTTAAATGAACGCATGTGATCCATCTTCAGTTCAGGCCATTGAACGCCCTTCAGTACCTATCATACAAAGACTAAACGTATAAATTGACAAGTATTAATTGAAGCGGCGCCGGCCAAATGAATTTATCAAACTATGGACCGCCATATGACGATAGCTAGAATCTCCTCCACAGTGCTTGTCAAAATCATTCGCAAATTGAGACGTTTGAAACCAAGACTGATCCTTGTGAAGTAGAAACATCTCACTGTCAGGAGTCAAAACCTCATCTTCTACTACTTCGTTGAAACTAGAATAGTAGCGTAGAACAAAGGCACCAGCATCCAAACGGCAGCTAGCAGAGCAGTCCACAAGTCCATTGATTCTTCAGAAAAGTTTTCTTAGAGAGAGAGAGAGAGAGAGTCCTTTGAAACAGGTAAAAACAATCTCTGAATAATAATCTCTGGTCTCTTTGACCACTATATTGCTCAAGTGCTACTACTTCTCGATCGATGTGTTCGACAGAATGCAATTTTTTTGTCCAGCAACCTAGAAGAGTCACCGTCGAAAGTACGAAAATTAAATCGATCTATGCGAAAATCAGGACGGCGAGAGAGTAGTCGTCTTACCCTGCAGGGCACGGCGCCTACCAAGGCACACGCCTCTGAAACTTGCAGCCGCCGACGTTGATGTTTGCCGATCCAGTAATCACTATTGGACAGGATGATCAACGCATGCGGAGTGCAGCCATCGCTAGCAAGATACTCCCTCTGCCCCCTAAAAAAAAGACAAACCCTTAATTTCTGTGTCCAACATTTAACTGAATAAGATGGACGGTCAAACGTTGGACACGAAAACTCAGGGTTTGTCTTTTTTTAGGACGGAGGGAGTATCTCCGTGAAGACAGTGTTGTTGCTGACTACTTGACATTGATAACAAATTCAATAATCCATCCACCAAAGTGTACTGGGTTAATGGTCGCCGGCCGGCTGATGGTTGTCT

>Os07t0575600-02 2000 bp upstream sequence

GATGTACAAGGAGTTCAACCGCAATTCCATTTCATACATCTCATCAGCAAGCATGGGTGCCATTTCTGATATCTCGGGAGGAAGGTGACAAAGGAAGGGAGCTCGTGATTTTGAGAAGCAGTTGGTGAGGATATGAAAAATCGGGTTTCTCAGTTTATTACTTCTAGTTTATTTTCTGAATTATAATGGACTGTTTAGGGGGCTAAAAATTCAAAAAAAAAAGGCACACATCAGATACTCCCAAAGTAGGGCCAAAAATGGTGCTTGTAGCATTGCAGTTAAGAGTATGTTAACGGTGTAACTAGATGCTTTAACTATCTTGTTCTCCTTGTACTATGAAATCGAGATTCTTCTGTCTTGATTATGTTCATAGTTTGATACAGCATCGGGATGCACTAAACAGTTAAGTAGCAATCTTGAAAAGTATCAAAAGGTTTTTTTCTAAAAAAAAAGTAATACCTAGTTTTATAATAGAAAGTACATAACCAAGAGTATAAAACTACTCTTGATAGAAAGAAAAACTAGCGCTGCTATTTTTCTTTGGCGAACACGCAAAAAGACTTGCGCATCAATAACACAATATGTTATTGGAAGGAAGCAGATTGTTTACCCACCGCTACTGACTGGTAGCCATCGCCGAAGGAGGAGGAGGTAACGGCTGTCGGGCTATTACAACTAACTAGTACTGCCAATACAAATATTCATGCGGACTCTTGCACTCTACAGTAGGACTTGTCATGATTAATCACAAGTTTTACGTGCATCTACAAAAATTTCCTGTCAAGAGAAAGCAAGGTGCTAGTTTTCATGGATGTAGTCTTTAGGATCTAGCTAATCCGATTGGTGGTGTTGTGCAGCAGCTGCTTCTGTTTCTTTCATGAACTTAAATGAACGCATGTGATCCATCTTCAGTTCAGGCCATTGAACGCCCTTCAGTACCTATCATACAAAGACTAAACGTATAAATTGACAAGTATTAATTGAAGCGGCGCCGGCCAAATGAATTTATCAAACTATGGACCGCCATATGACGATAGCTAGAATCTCCTCCACAGTGCTTGTCAAAATCATTCGCAAATTGAGACGTTTGAAACCAAGACTGATCCTTGTGAAGTAGAAACATCTCACTGTCAGGAGTCAAAACCTCATCTTCTACTACTTCGTTGAAACTAGAATAGTAGCGTAGAACAAAGGCACCAGCATCCAAACGGCAGCTAGCAGAGCAGTCCACAAGTCCATTGATTCTTCAGAAAAGTTTTCTTAGAGAGAGAGAGAGAGAGAGTCCTTTGAAACAGGTAAAAACAATCTCTGAATAATAATCTCTGGTCTCTTTGACCACTATATTGCTCAAGTGCTACTACTTCTCGATCGATGTGTTCGACAGAATGCAATTTTTTTGTCCAGCAACCTAGAAGAGTCACCGTCGAAAGTACGAAAATTAAATCGATCTATGCGAAAATCAGGACGGCGAGAGAGTAGTCGTCTTACCCTGCAGGGCACGGCGCCTACCAAGGCACACGCCTCTGAAACTTGCAGCCGCCGACGTTGATGTTTGCCGATCCAGTAATCACTATTGGACAGGATGATCAACGCATGCGGAGTGCAGCCATCGCTAGCAAGATACTCCCTCTGCCCCCTAAAAAAAAGACAAACCCTTAATTTCTGTGTCCAACATTTAACTGAATAAGATGGACGGTCAAACGTTGGACACGAAAACTCAGGGTTTGTCTTTTTTTAGGACGGAGGGAGTATCTCCGTGAAGACAGTGTTGTTGCTGACTACTTGACATTGATAACAAATTCAATAATCCATCCACCAAAGTGTACTGGGTTAATGGTCGCCGGCCGGCTGATGGTTGTCTGTTCGTGCCAAAACATGTCCTTGTCGATCTGAATCTTTCAGCTCTCATCGCTCGACACCCAACCACTCGATCATAAGCCAAGCAGTTTATGTTAGCCAATTGCTACAATATCCTTAAGAGGCGTCAAGGCGAAGTGTCA

>Os07t0576000-01 2000 bp upstream sequence

TCACGGCCTCGCCGGACAAGCCGCCGCGCGACGAGGTCGCGTGCGTCCGCGCCGACTTCACCGACGCGTGCGAGGCGGAGGCGACGGTGTGGGACAAGGACGGGTTCAGCGCCGTCGCCCTGAGGCCGGCGGTTCGCGGCGTGGACGCCCGCGGCGTGCACGCCGGCACGTTCGTCCTCGCCAGGAGCGACGCGACGGCGGCGAGCGCGTCGGCGCTGGCGTGCCTCAAGAACAACGGCGCGGCGTACACGTCCTGCATGCCCGACCTCGCCCAGGTGAACGCGCTCCTCGCCGCCTACGCGCCGCAGCTGTTCCTCCACCCCGACGAGCCCTACCTGCCGTCGTCGGTGACGTGGTTCTTCCAGAACGGCGCGCTGCTGTACCAGAAGGGTAGCCAGACCCCGACGCCGGTCGCCGCCGACGGGTCGAACCTCCCGCAGGGCGGCGGCAACGACGGCGGGTACTGGCTCGACCTGCCGGTGGACAACTTCCAGAGGGAGCGGGTCAAGAAGGGCGACCTCGCCGGCGCGAAGGTGTACGTGCAGGCGAAGCCGATGCTGGGCGCGACGGCGACCGACCTCGCCGTGTGGTTCTTCTACCCGTTCAACGGCCCGGCGAGGGCGAAGGTGGGGCCCCTCACCATCCCGCTCGGCAAGATCGGCGAGCACGTCGGCGACTGGGAGCACGTGACCCTCCGCGTGAGCAACTTCTCCGGCGAGCTCCTCCGCATGTACTTCTCGCAGCACAGCGCGGGCGCGTGGGTGGACGCGCCGCAGCTCGAGTACCTCGACGGCGGGAACCGGCCGTCGGCGTACTCGTCGCTGCACGGGCACGCGCTGTACCCGAGGGCAGGGCTGGTGCTGCAGGGCGACGCGAGGCTGGGCGTCGGGATACGCAACGACTGCGACAGGGGGAGCAGGCTGGACACCGGCGGCGCCGGGCGGTGCGAGGTGGTGTCGGCGGAGTACCTCGGCGGCGGCGGCGGCGGCGTGGCCGAGCCGACGTGGCTGCTGTTCGACCGGGAGTGGGGGCCGAGGGAGGAGTACGACATCGGCCGCGAGATCAACCGCGTGGCGAAGCTGCTGCCGCGGTCGACGAGGGAGCGGCTGCGGAAGCTCGTCGAGAGCGTCTTCGTCGGCGAGGGCCCGACGGGGCCGAGGATGAAGGGCAGCTGGAGAAACGACGAGAGGGAGGCCAAGTAAAAATTTTGGCGGTTCTCAAATTTCAGACGACGACACGCGGTGATGACGAGTGAAAAGTTTATTATATGGGGTTTCTCAAATTCAAATCAGATTGTTACGGAGCAATTCTGAAAAACGATGACAGAGTGCATGGTGAAATATATTCTTTTAAGATAAGATTTAATCAAACTTAAAATTCCAATCACAATTGTTTCTCAAATGTTTGGTTTTTTAAAAAAATACTGTACATAGATTCACCATAAGAACTCCTATAATAATATCATAAAATTTATTGTATTTTGTAAACTTACTCTAAAAAAATTCTAATACAAAATGTCATCTTAAAAAGTAAATATTTTTTAAACATCAAATATTTATGATTGAATAAAGTACCGTTGTACAAAATTTCAAGTCAAACTCGACCAAATAGATCTATATTTTTTTTCCTTTTTCTTTCAAATGTGTATATCGAACTTGCAGTTCACTTTCTTTACGATGGTGTATTTGTATTGTAAATATATCATATTTCTAGTAATATGTGAAAACAAATGTAATTTCTGGTAAATTTCACTTTTAAGTAAGTTTTATATTTTTCTTCTCATTTTGACGCAACTTATGCTTATACTTATTAGCCAAAATTTAATTTTTCAACATTTAATCTGAAGCTGATTTTAGGGGTTTTTCATCGAATTTTATTTTTCATCACTAAAAACACGTACATAAAAGTTTTATTTATAAATTATATTTCGTTTGTATAAATATATTGTTTGGCTTCTTCCCCGAATAAGCTAAATGATTGGGCTGTACCTTCCTCTCCGC

>Os07t0663800-01 2000 bp upstream sequence

AAGAATTATATGTAAGGATAGTGATTGAGACAATTAGACTATGTTTATTTATAAAATATGTATATATTGATGTTGCAGGTTGGCTGGCAAGGTGGCTGTCATAACCGGTGGAGCTAGCGGCATCGGCAAAGCGACAGCCAAGGAGTTCATCGAGAATGGCGCCAAGGTCATCATGGCCGATGTCCAGGATGACCTCGGCCACTCCACCGCAGCGGAGCTCGGCCCGGACGCCTCGTACACGCGCTGCGACGTCACCGACGAGGCACAGGTCGCGGCGGCCGTCGACCTCGCCGTGAAGCGGCACGGCCACCTCGACATCCTCTACAACAACGCCGGTGTCATGGGCGCCATGCCGCAGGACGACATGGCGTCCGTCGACCTCGCCAACTTCGACCGCATGATGGCGATCAACGCCCGGGCGGCGCTTGTCGGCATCAAGCACGCCGCGCGCGTCATGTCGCCCCGCCGCAGCGGCGTCATCCTCTGCACGGCCAGCGACACGGGCGTCATGCCCATGCCCAACATCGCCTTGTACGCCGTCTCCAAGGCAACCACCATCGCCATCGTGCGCGCCGCGGCGGAGCCGCTGTCGCGCCACGGCCTGCGGGTGAACGCCATCTCGCCGCACGGCACCAGGACGCCGATGGCGATGCACGTGTTATCTCAGATGTACCCCGGCGTAAGCAAAGATGATTTGGAGAAGATGGCGGACGCCGCCATGGACGCCGGAGAGGTGATGGAACCTAAGTACGTCGCTAGGGCGGCGCTGTATTTGGCTTCGGACGAGGCTAAGTATGTCAACGGGCACAACCTCGTCGTTGACGGCGGCTTCACGTCGCACAAAGGATCCGACACACGTTTGAATTAGCGATGTGTCCAACTATAGATGGACAAATGGACCGCTCGGCCCGGGCACGACCAGACACGGACCAATATGAATTAGTCACATGTGCTCCTCATTGGAATAAGTCTGTTGTACCTCCCTCATCTGTTTGGGTTGTCTTATATGGCTGGATTAAGTCAATTTTGCATCCCTCGTCTCCTTGGGCCATGCCTTATGTGACTAGAAGAAGTCTATATTGCATCCCAAATCGTTGGATCGGGTCGTGCCAGGCCGGCATGTCGTGCCAAGGAAAAGGTCCAAGCACGGCCCAACAATCGGGCTGTGCTGGCACGGGCCTAACAACAATCGGGTTGTGCCGTGTCTAGGCCGGGCCAAAACCCCGTGCTGTGGGTTGAGCCGTCGGGCCCTAAGCCTTTTGGCCATCTATACGTCCAACTAACATATATTGAACAGACACCACTACTTGTCTTGGTAGTCTCGTTAGAATAAGATAAGGTTGTCACATATGTGTTGTCGGAATTAGAAAAATATCATTGATATGATGGTAGAGTTATTGCTGCATGACTCTAGTCATATAATTTAAATTCTGGTTTTCTAAAAGAAATGTTAAATTTTGGTGTTCATAAGTATGATTGTGTGCATCAGTGGATTACAGTAGACGACTAGGCAATTTTCATAATTTACTATCTATATATGTACAATACAATAAAACAAAACTTGCATGGGCAACTCTAAGGAGGTATGATTGTGTCATCGTAGTAAATTTATGTTTTAAATTAAGGACACATATATAAATATTTATTTATAAATTATTTTTTTAAGTTGCTAATAAATCATCCGGATAAGCATAAGGATAAATGAAACGATAGAACCCTCAATCTATCGCAATCGTTGAGTCACTGGTAGCCGCGCCACAGCCATTGCAGCTGCTTCCTTTCATTGACAGCTTGGAGGATAAGACACGTGCAAATTGCCATGGAAAATTGGCAATGCCACAAGAGAAGAGAAGGTCCACCTTATCAACAACAATTAAGAGAAACCATGTGGAAGAAGCTGACACTGTACTGATTCCGGCACGCAATAATAGAAGACAAGAGACAAGGGGTAGGTAAGATGGCTGGCCTTCTTAAGGAGAGGCCATCTGTTAATTAGTACT

>Os07t0677300-01 2000 bp upstream sequence

CAGTGTCTGTCAGACCAGGGATGGTGGCCCGTGGGCGCTGAGCAGGCCGGCCTTTCATTTAGTTCGTGTCACCTGTAGGCGGCTAGGCCGGAGGGAAACTAACGTACACTTTTGTTGTTATTTATAACTTTATCGACTATAAATTTTGATAAGCAACAAATCAAACATGCCATAATCAATTAGGCAAAATTCAGTAATTTACCTTAGCAAAATTTAGTAATGGAAAAATTAGGAAAAACTAAAGTTATATTCCCTCCTCCTCAAAAAGAATACAATTTTAGATTTTAAATTTATTCCAACATATAACGTACTCTATAGTATTGCTTTTTCGTCCAACCACCACTCATTCACCCATATATTACTAACCTAGCTACTCCACCATTCCCAACTGTCCCTCATTTGATGAGTGGTCGAGTATTTTTCCTATACTATACTAGTTTGTCTTTTAAATATTACAATTGTATTTTTCTTTTTTAGAATTGAGGTAGTAAATTTGTAAACAAAGTGAACAGGCCACCAACCTACTAGGTGGACCACTGTACACGATAATATTATTTGTGCTATTGTGTTTATTTTAATCTAAGTTGCAAATCAAGAGAGTATTTTATTTCATTCAAAAAGGAATAATTTGTGAAACACTCAGCAACTGTATAATATTAGCCGTGAAACTCGGTTAACCATAATTTTTTTTCAAAGGAACTATACGATAATTTAATTATTTTACTATCCGCATTTTATGGCTTAAGAAACAAGAGTAACATACGGCACATGCAACAACCACCATTCTAGCTAGAGCTAGAAATGTCGGCCCAACTTGTGATAGCTAATTACGTTCGAATGGAAAATCATATCACCAACATGCATGGTTTAATCATGGCAACGCTGATGCGAAAGCTCTCGCAGATCTATTATTTTTTCCCTGGAGATTGATCGAAAATGATGGCACAACGGTATGTAATGCTATAACAGCATATATTGTCTTGTAACACACCGTTTGCACTTAAATTAGACAATTAGTCCGAACGTACATAAGAAGATGAATTGCGATAGACCATTCTAGCTCGTCACGAGCTGAATAGTTCTCGCACTATTATACCCGAGATCAATTAGCAGAGTATTAATAAGGTAAAGTTATTTTCATAAACTCTGTACGACGATAGTGCATACATACCAATCTTTAACAAAGGGACAAATTATCGACGGCATTTGTATGTTCATGTGCTATTCTATGGCACGGGTGCTCTTTTTTTTTGGCTTAAGCAACGCGTGATTAATGGTTAACAGATTAAGGTCAAGCGAAATGGACCGAACTGACCCTACATTGCATGAATAATGCAGAGGTGATGATGTTGGCCGTAGGCCGTACGTAGAAACTTATGTTGGACACACATGTAAGTACTAGTAGTCTAGTAGCACTAATAAAGTTAACTAATAAATTATATATGATGTCACTTTAAAAAATACAGATTCAAATTCGATATATTGAAGTTGAACAAATTAAATTCTAGCTAGTTAGTATGCACACATTCACACTCATATTTGTTTTTGTGTTGTAATTTATAGAAGTTAAAATTCAACTTGCAATTTGTTAGTAAAAACATAACTATTTAGATGACTAGAAACATATCCTTCTGTTGTAATGTGCCAGGCTCATATAAGTAGAAATATGCTATTAGAAAATTAGAGATAGTACTCTGTGCGTGGTGGGAGGAGGGATGAAAAAATGACGAGTTCGATTTTAAGGTAGTAGAGATATGTACAAAGCAATAGGATATTCCTATTGAGATTAAAATACATTCCCCTGCCCAGCTCCCCTCGTATGATTAATGTACCTGCATATTTCGTCTCATCTCGGCACCAATCGATGGCGTCAAGCCTGTAGCTAAGCTAGCTATATAAGATGCAGCGTGGCACCAGCCTCTCTTGCAAGCCACGGCCAGCGAAAAAGCTTAAAAAAAACTGCAGTTATTTAGGTAGGATTAAGCATACGCGCACGGCGC

>Os07t0687500-01 2000 bp upstream sequence

TTTTGTGAGCGTGGTGTGCAGCCGTACGTGCAATGTGCGTGCCTTTATAGAAACAGTAGCCCTGTTTCGGAACGATATCCTGCAGTAACAGACGAAACAGTGGTAATTTTCTGCAGTAACAGACGAAACAGTGGAAATTTTGGGAGAATTGGATCAGAGCATTTTCATTGGGCGGCTGGGGTGAGAGACAGGAGTAGATATTATTGTGCCAGTTAAGTTTTTTTTTATATGATGAATATTCTGGCCTTTTTTTTAAAAAAAAAAGAACTGGCATTTTCTTCAATTAAGATAGAAACCACTTATTACAAAATTTTGAACGTAATGACCAAAGATGATGAGAGAGTAAGTTCGAACAACAAAAGGAGAAATCTTATTCAATCCTATGTGTAAACTTTCATCCAAAGTTAAGCCAATATCTACATTAATGGACTTCAAATTCCAACATGCAGTATGTATAAATCTTTTATCCTTATCACACATTTGTAGTTAAGTTCAGAATCTGAGCTAGTATGCTATGCTACCTTGAAAAAATCCTGCCTATAAAATTTAGTTGGAGATTTGTACCAGCTAGCTACTGTAGTTATTAATTTTAACCATAGAAAAATTCACGCATGGAAGGAAGGAAGACAAGACAAGAGACTTGTTGGTTCGAGTTAGAGGGAGGTCACCCAGTCAAGTACTCTTACCCAAAGGATAGGTTGGAATTAACTGAGATAACCCATTCAAAGTCCAGATTCATAGGATGTGTTTCATCTATCTACAAACAAGAAGTTATCTAGGTAAAAAGATGAACGGTAAAACCAGATTGCTAGCTAGCTAGCTGTCTCACGGCTATTCGCTCTCTCCAGTCAGTCTCTAGCCAATTGGCTAGCAGGTAAACACAAGTTATTCTCTTCAGTCAAAAAATGAAGTCGTTTTCGAGTATATATATTATATTTGGATTCTTTGAAAGCAAAATCATCCAGCGCAGCATTGATCGTCAGTGATGACCGTCATCGACGATGGCATCGCAACCGCTCGACGACGAATTGAAGAAGGTGGGTGTGCTAGCCTGCTACTATTTTTTAAAGACTTGAGAAATTCAGAGCTAGAAAATCATCCATTCGTTTTGAGTTTGACCTGCCGATCGATTAATTATATTCCTTGAGATCGATCGATAAATTGTCTCATTCACACGGACCCAAGGCGCTCGTACATTTCAACATCCGTGCGCACGCGCGTGCGACTGATCAGGATATATAGTTTTTGGTGATCGATAAAATTCCCAGGTAGCTAGCATGCAGATGCAGATCATACTTATGTGCCTAATGCATGCCTAGACTAGATCAATCGATCGATTGCTGATCGAGGACAAAAGCAATCAGGAATCCAGATGCATGCATTGTCGATTCATAATTGACTAATTAAGCAAACGCATATCCAGGATCAGATACTAGTCCAATTGAGTGATTATTGACCATATAGCTTGAGTACTTTTATGGTACATTCTAGACCGGTATACGTACTAATAGCTGCTGTTTGCAGTCAAGATACATATAGATACTAGTACTACAGTCAATGTCTATCCCTTTCACGTAGCTACCGCCAGTAAAAGGCAGAACGATGAGAGAGAATCTCATTGTTCATATATGGGCTTTCAATTAATTCCTCGCAATATATATTACCAGTACTGTACGAATACTAGTATTACCAGTGATAAGAGACCGATGCACGATTAAAGGTAGGGTCCGGATCACCATATGTTCGGTGATGCTGGTTTGCTGAATTTTTTTAGAGGGAGAAGTTCGGTGATTTGTTCGAGTAACGGAAATGGTGGCCGCATTCTAAGCGGGCCTCATTCTTGGTAGGGAAGCAGGTGGAAGGAGGACAGGAAGGCCCAATAGTAAGTAAAATGGGCCGTCGGATGACATGTCAGGCCCAAGTTGAAGTGAGCCCATACAGCGACTCCGGCCTTGTTGACGAACGAACGAATGATGCGGAGGTGAGGGGATCTTTCTCTT

>Os08t0378000-03 2000 bp upstream sequence

TCGATCATTTATCCCGCATGAAAGGGGACGGGCGAACAGCCGTTCTTTATATGCTTCTGGGCTCGCGCTCCCGCGCACACACTGCGCACACACACACACAAACACAAAGATGGAGAGGGTAGCAACACTGGTCTCTTGTATAGCGGCCGCGACCGCTATGCTGCTTGCGGTGGCAGCAGCCGCAGCGGGGAGTGGCGGCGGCGGCTGCACGCGGAGCTGCGGCAACATCAGCGTGCCGTACCCGTTCGGCGTGGAGGCCGACTGCTACTACCCCGGCTTCAACCTCACCTGCAACCACTCGTACCACCCGCCCACGCTCTTCCTCGTCGACGGCGCCGGCACGTCCCACCAGGTGCTCGACATCTCCATCTCCAACTCCACGGTGCGCATCAACAGCAGCCTCATCGCGTTCACCAAGTCTGCCGACAAGCAAGCCGTCATCAACACAACGTGGGAGGTGAGAGGCCCGTACTACCTGTTGGATGATCCTTCCAATATGGTCGTCTTGGTAGGCTGCCAAGCGCGGTTCGACGTCCGGGGAGGAAGCAACAACACCCTGATCAGCTCCTGCACCGTCGTGTGCCCATCACCTGATCCTAGCACCACCTACATCGGAGCTGGCGACGGCTCCTGCTCGGGCGTCGGCTGCTGCCAGGCGAACATCGTCCTACGCTACTCTCGCTACACCGTCCAGATCCACGACCTGCAAGAGCAGGATGCGCCGTCGTCCAGTCCTACTTTTAATGATGGCTCGGCGTACATAGTAGATCAGCCATTCAATTACACCGGCCTTATGATTTTCGAAAAAAATTTCCCTCAAGCGCTCCCAGCCATGCTAGACTGGTTCATAGTAGGTGATAATTCGACATGCCCGATGTCGACGAATGAGTCTGCGCCTGCGCCTGCGTCCGCTGCCGAGTGTCGCAGCGTGCACAGCTTTTACGAAGGTTACTACGGTGACGTTTCTGACGATGTGGTGGTGGGGTATCGGTGCCGCTGCTCTCCGGGTTACCAAGGAAATCCTTACGTAAAAGATGGATGCTACGGTATGTGTTTACCCTCTCTGTTTTTTTTTACGGAATTGCCAGAAAACTTGCGCAAGTTTTTTTTCATCCCAGCTTCCCAATTTTTAGCCGTACGATGCTACGTTAAAATTTGTGTTAAACCAGTTATATATTATAATTACAGTATAGTTACAATTAGTTACAATAAGATGCAATAACTGTAACTATATTATAACCTCTATATAATTTAGGTATAAAAGGTTGCCTATATTTTTTTAATACTTATATACAAGTTATATTATAGCTATAATATAATTATGAGGAATTACATCATAACTATGTTACATTATATTTAAAAGTTTCCACTTAAAAAATTTGCAACAGATTTTTTAGATAATTATCCTCTATCTCATAATATATGACATTACTTAGTTCATTTTAAACTATCCAACGTCACAACAAAATAAATATATTTATAATATTTTTAGATGCATCTATACATGATGTGACAAATTTCCAACTACAAAGGACAACATTCAAATTTCTCTAAATCTCTACTACTTAAAAAAATTATTTTTTTAAAGAAGTTTCCGTCTGTCTACAACAAAAAAAAGAGCGAAAAAAACGTAAAAAGAAAAAAAAAGAAAGTCCGATTCAATCAATGACGCGGAAAAAAAAAGCTGTAAAAAAGGGTGATAAAAAAACGTAAGGAAGGGTGAAAAAAGGCGCGGCAGATCGCTCCCTTGTGTCCTAACCCCGAGTCTGATTAAAATAGATAAAATCAAATCTTTTGCAAAAGTCAAAGCCTCCTATTTCTATTTCTGAAGATATTCTGTCACACAATCCCCAACCCCGCCCCCAAAAAAAAATTCCCGGTCAGAATTCCATCGCACAAATAAAACTGAAGCATATACACACAAATTTTATCCAAAAATTCCCATGCAGCTAGATCTAAGGGGAGACTACCGCCGTCACCGCCGAACCGCCGCCACCGTC

>Os08t0506000-01 2000 bp upstream sequence

TGTGTTGCCACAAGCAACGAAGCAAATACCAAGCCTGGATGCTTCAGCTAGACATCTCTCGGCGTCTATAACTCTGTCGGTATGTTGTTTATTAACCACGACTAAAAATCTTGGTTTTGAAACTAAATTTTGAAGTTGGTTTTAGATTTTTATTGTTCATTGATTCTTAATCCAATAATAATATAGATTAAGGATGTGTTCTTATGCCACTTTTTTCAACTCACCTCTCTTGTTTTCCACGCGTACGTTTTTTAAACTACTAAACGGCGTGTTTTTATGCAACTATATATATATACCCACACACACGACAGTTGTTTTGAAAAAATTATATTAATCCATTTTGCAAAAATAATAATACTTAATTATTTATGTAAGAATATGTGCTTCGTTTTACGTGCCAGGAACCCCTTCCGAACGCAGCCTAAAAGTTTTACTCGTAATTTATCTTTGGCTGCTTCTTTTCTAGTATATCCTATCGGCCATAGCTCGACTATCAGATTAAGAAGGAAAATCACATAGTTACTTCGTTTTTCATGATTTTTACGGTGCTATCAATAAATTCCCATACAAGATTTTCAAATAAAGGTTGGATACGTGCTAGATTACTTTAGTTTCTGCAGGAACGAACAATATAGTTTTCATTCGTTCCAAACATGGCCACAGATATTTATCAAAAAATTCGAGTGGGCTATATGGACAGACAAGTACCATCAAAATGTTATCATGAATCCGGATCTAGGGGGAGTACTATATGTTTGATTGCTTGAGACAGTCTCTTCAGTACTATTTCCTTCCTCTCGTAGAAATTAACTTCTGGCTGTGAATCTAGACATAGAGCCTATTTAGGAAACTTATAGTTGTTGCAGTTTTTCTCAGAATTAGAAGCTTCTCAAACAGTCCTGTTTTTTTTATATTCTAAGAAGCTGTAGTTGTAGAATTCAGAAAATGAGAAGCAAGAAATTCTCAAAAGCTAGCTACCAACCAGCTGCTTATTAAAATTTTAAGCTCCTCAAACAGGCCCATAGGCTCATAGTCAGTTGGTGTTTTCTTTGGAATAAGTTCATCTGAGGTCCCTTAACTTGTCAACGAATCCGATTTTCGTCCTTCAACCAGAAAACCAGATACAACAGGTCCTTCAACTGTCAAAACCAGTGCAGATGAGGTCCCTCGGCGGTTTTGGCTGACGTGGCGCTTACGTGGCTAATTTGACTTGGTCTTTATCTGATATGGCATTGACGTGACACTTACGTGGCAATTCGATCTAGAAAAATAATAAACTTCGTAGGACCCACATGTCAGTTTCACATATAAATTAATAAAGAAATGGTGGGGCCACGTGGGCCTAAATGTCAGCTAAAAAATAAAGATGGTGGGGCCCACGTGGGCCTACATGTCATTCTTAGTCTCCTCCTTCCTCCTCTTTCCCCACCTCCCCTCACTTCATCTCTCTCTCCTCTCCTCTCTTCAGGCACCAGGGAGGTCTAGCCGCCGGCGCTACGGCGGCGGCGGCCAGCAGCAGGAGCGCAGGGCGGGCAGATGAACGAGGCGGCGACGGGCAGAGTGGAGTGCGGTGGACGGAGCGGCGGGGCGGGGCGGTCAGAGCGGTGGCACCAAGCCGAAGGGGCACATATATTTATCAAAAAATTCGCATGGGCTATATGGACAGATAAGTACCATCAAAAGGTTATCATAAATCCAGATCTAGAGATGTATATGTTTGATTGCTTGAGACAATCTCTTTAATATTAATTCCTTCATCTCATAAAAATTAATTTCTAGTTATAAATTTAAACATAGGCTCGCAGTCCGAAGTTGATTTTTTTTTTTTTTACGGAGTACTCCCCTACCAGTGACGCATTAGGTCCTCCTGGCTAGCAAAAATCAAGGCCCATTAAGGACTAAAACGGCTGTAAACAATAGGCCCATAAACCACCCAATTCAGGCGTGATGGGCCCATGGAATGGTATATAACAAAGCCCCACAATCGCCGTACTCTGTCG

>Os08t0512100-00 2000 bp upstream sequence

ACTAATATAACAATGTGATCGGGTCATAGAACAGATAAGTGGTTTAGGAGGAAAAAAATATTTAAATAAAGAAAAAAAAAGAAGAAACTAGTAGCTACTCCCTCTATGCCACAAAGGCTCCATTTTTCAGTTTTTGATACAACGTCTACTCTTCATCTTATTTAAAAATATTTTATTATTGTGTGTGGCTAAATTTTTTAAATTTGTTCATAATTTTTTAAATAAAACGGATGATCAAGCGTTTTCAAAAATAAAATATTAGTGGGACGAAGGGAATAGCTGTGTAGTCTACTGCGTCAGGGGCTCAGGACGTCTGGTTTTTATCCTGTGCCGATCGCAAGTATTCTCTTTTTCTTTTTCACTTTTGAAGATTTGGTATGATTGTACACTTCCCAGATGATTAAAACTGCAGTCCTGAGTTCAAGCCAATGATTTTTTCGTGCGAAATTCAAGTGAAAAAACTGAATTTGATTTTAAAGATCGAAGCATGAACGTGACCCTGACCCATGTCCATTGCTTGTCGACACGATAATCTGCAAGCCAAAACATGTACCAAAAGAGCTCGAGACCTGGCCGCCTTTGCCCGTCCTCTCGGGATTCGGCGCCGCCACGCCACGCACGCTGATGCCGCGGCCACTGCACACCGCGCCCGGTCTCGTCCCGTCTCACCAGCAGCGGCATCACGCGCACTCGGCGAGACGCGGGGCCGCCCACGGCGGCGACAACCGCAGCGCGAGCCGCAAGCGCAGGCCAAAGAAACGCACGCGCGCGCGCGGTAGGCAGCGGCAGCGGCAACGCGCCGTGCGCGCGCGCGTGCGTGCGGCGTTGCGGGAGGAGGCGTCAGCGGCTGCCTGCTCGGGCGGCGTCGCATCGCATGCCAGCGTCGCAGTTGGTTGATGCGATGCTCACGCACGCCCACGCCAACGCCAACGCCACCGCCACCGAGTTGGCCGCTCTTGCGTCGACGCCGCGGCTACGGCCACGCGAGCGCCGCCGACTTCCTACTTCCCGGGCCCGTTTCACTGACAGGTGGGGCATCTCATTAAAAGGATGGTTTTAAAGAATTTGTGGCCAAGATAGGAGGTTTTGGGATATCCTAATATCCTATACATGGTTTGTTTTTAAGGTGATTTATTATGGTAGATTTGAATATCTACAAGATATTGTTATTAAGGTGATTCTAAATATGTACCAGATTTGCTTTAAGACAGTTATTGTAGGAGATAATATTGGAGTTATGAAAAATTTGGATACATATGAATTCCACACGGTTGCTAACGTGGTATTTATCTTGGTGGCTTACATATCACTACTCTGTATGCCAATGTCTATTTTATTCACAAAAGGACACTAGCATAAATTTATTGAATTTATTTTTCCACACAATATTTATTGAATTATTGCTTTTATGTATAACGATGACGTTTTCATATCAATGAATGCATGCAGAAGATATATTTCATTAAACATCTAGCTCATACAGGGGTCTGTTCCACCGTGCGTCACCGTGGAACGGCTTCTCTCTGATGATGATCGAGAATCGTATATTGACTAGATACGCACCGTTTGTTTCGTAGGACGGCAACATACGACACATTGAATTGCATTGTTTGGATAAGAAAAATCTTCCTAATTCAATATAACCACACAAAGCGACTACTTCACGCGACTCTATTTTATACGCAAAACTTAACGGTTGCTACTTATGGCGGCTAGACAACGTGCATTGAATTTCTATATTCACACAAGGAAACTCTTTCCGTTTTAGCATGTCGGCGTAAAGCGTACATAGCTCATTGAATTCACATCTCAAACTCGACCACTATTATCCACTATTGCGCGGGAATTCACAATTCAAACTTACCACTTTGTATACAAATAAATAGTCGTAGCCCCACATGTCAGTGTCACCATTAGAGAAAGTTTTACACCTCCTCTTCCAATTTCCGGGTGCAGAGAGCGTTTATCACGGCCCTGAACCCTCCAAAAATATTTGCCGA

>Os08t0538000-02 2000 bp upstream sequence

TTTATGCGATTCCGCCCCCGGATTTTCTCCCCTTCGCTTTTCGGCCCCTCTAAACCCAGAACCCCAACCCTAGCCGCCTCCCCACCCGCCGCCGCCGCCGCTCGCCGCCGGATCGTCTCGATGGCCTCCTCGACGACGACGTCGGAGGACGCGCTGCGCCGGGCGCTGGCGGAGAGGCAGGCGGCCGTGGACGCGCAGGCGGAGGCGGTGCGGGCGCTCAAGGCGGCGGGGGCAGCTGCGGCCAAGGCCGAGGTGGATGCCGCGGTCGAGGCGCTCAAGGCGCTCAAGGTGGAGGCCGGCGCCGCCGCGCGGAGGCTGCAGGCGGCCGTCGGGGCTTCCGGCGGCGGAGGCGGGGCCGCGCGGGAGGAGATGCGGCAGGCGGTGGTGAACACGCTGGAGAGGAAGCTGTTCTACATCCCTTCCTTCAAGATCTACCGCGGCGTCGCCGGGCTGTACGACTACGGCCCGCCGGGATGCGCCGTCAAGGCCAACGTGCTCGCCTTCTGGAGGCAGGTCAGGCCGCGAGCGGAGCCATCTCACAAATATTACCTGTTGTTGTTCACGCTTGTTCAGTTCATTCGTTGGGGATTAAATGTATCCGAAGTTTGTTTAGATTAATTAGATAGTACTCCGTAGCACCAATCTAGTTTGAAATATTTCGTTGTGATAAAATCATAAAAATGTAACTTATGCCAGTGAATGTGTACTAACTTATTCACTTAATCTCACAAACATGACTTGTTGTTCATGCTTGTTCAGTTCATTCGTTGAGGGTTAAATGTGTCTGCTCTTACAGCTCAATTGACGTGTTCAGGTTTGTTTAGATTAACTAGAGAGTACTCCGTAGCACTGATCTAGTCCGATATATTTCGTTCTGATAAAAATGCAACTTATGCCAGTGAATGTGCACTACCAATAGGAGATTGTGACCTGAAAGTAGAATTATTTAGTTAACCATGTGACACTGTGTTCTTGATTGCTTTGTTTTGCAAGAATAGCTTTTATATGTTTGAAATTGCTTGTGTATTTGATAACTTTTTATTTTGATTTTACTTATGTTGGGTACTAAGATTTTAGTCACCAAATTCATTCTTTTCTTTTCAGCACTTCGTTTTGGAGGAGAACATGTTGGAGGTTGACTGCCCCTGTGTGACGCCTGAGGTTGTGTTGAAGGCATCGGGGCATGTCGAGAAGTTTACTGACCTCATGGTTAAGGATGAGAAGACAGGCACATGCTACCGTGCTGACCACCTGTTGAAAGATCACTGCAAGGAGAAGCTTGAGAAGGATCTCACCTTGTCCCCGGAGAAGGCAGCTGAATTGAAGCATGTTCTTGCTGTCCTGGATGACCTTTCAGCAGATGAACTTGGCGCAAAGATTAAGGAATATGGGATTGTTGCCCCTGATACTAAGAACCCGCTCTCTGATCCCTACCCGTTCAATCTTATGTTCCAGACATCCATCGGCCCTACCGGTCTTAATGTTGGGTAAGAATATCAATGTTCACTTTTTTTAACCCAAGAGATTAATGTTCACTGTTTAATTCTGTTGTTATGCTGATTGGCTGAATGTGTTCAATTTTGTGTGAACAAACAGGTATATGAGACCAGAAACAGCACAGGGTATTTTTGTCAACTTCAAGGACTTGTACTATTACAACGGGCAAAAGCTCCCTTTCGCAGCAGCTCAAATCGGGCAAGCATTCCGTAATGAGGTCTGTGTTTTGATACATTTTGTCTTTGCGTTCTGCATTTCGATTATATGCTTGAAGGAGTCTAGACTTATTGACTATAGGTCTATAGCTAACTATGCTTAGGAAACTCTCCACTATGCCATGGTTTTTTAGAGGGTTCTTTCTTTTGTGTTATACTCACTGTTTCTATACCATGCAAATCAGATTTCTCCACGACAAGGTCTTCTACGTGTGCGTGAATTCACATTAGCCGAGATTGAGCACTTTGTGGATCCTGAGGATAAATCTCACCCAAAATTTGTTGATG

>Os08t0557500-02 2000 bp upstream sequence

CCTGCGCCCCCTCTGCCGCCGCGGCGCTCGCCTCCGCAGCCATCTCCCCCTCCGCCGCCGCCGCCGCAGCCGCAAATCGAAGGCGAGGGTTTAGAAACGGATCGCGAGGACGAGCCTGGCCGAGACCCGAGCTGGAGATGGAGATTGTAGTGGGAAAAGGTGAAGCGGTGCAGTGACGACTGGCTGCCACAAGTGGAGGCCGCGGCACGCGCCTCCTAACCGGGTATCGCGTTGTCGGGTTCGGAGCCGGAGATGAGCAGGCGGCGGAGCTCGCCGCGGCTGCTCCGGCCATGGCCGCCGGCGCCACGAGCTCCTCCCTCTCGCGTGCGCGGGGGAAGCTGTGTGGATGGGGGCAATTCGAGCGACGAGTGGCTCGCGCCGCGGAAGCGGAGATGGTTCGGTGTACTGGAGCTTTTGAATCCGAAAACTCCCTAATAATCCTCGGAAAAAAAAAGGAGAGAAAAATATCGATAAGGTTGAGGAATTCGTCTAACTGGGTTGGGCCTACTGGACCGTTTTAATATGGGCCGTGGGTTACGGCCTGTGGGCTGGCTGTTGTAAACATTTGCGTAAAAAACCCCAAGAGAAGAATTTACTTTTCATTTGAATCCCCCGTGTCACTGTGTCCGTCAGATCGGCCATTGGCGGCTAGAATCGAGGCGGAGAGTAGGGACTTGTTTTTCTGGAAACCCCCTATTATTAGTTTCGTAACACAAATAATTGCTCCTCGTCGCCGGAGCAGAAGGTCTCGAGGAGTCGAGACTTCTCTCCCCCAAATCCCCAGAACCCTAGCGGAGCTCCACCTCGAGATGGCGACGGCGAGCGATGCGCCGGCGAGCTCCACGATAACGACCGCCACGGACGACGCCGAGGTGGAGCGCGACCAGGGCAACGGCAATGGCGCCGTGTCAGCCGCACCCGCCGCCGTGGGGAAGGAGGCGGCGGCTGAGGAGGAGGAGATGATCGGCCCGGCTCCCGTGCCGCCGCGGCCGAGGAAGAAGCGCCCGCTCCAGTTCGAGCAGGCCTTCCTCGACGCCCTGCCCTCCGCGGCCATGTGAGTGTTCTCCTCGCCGTTTCAGGTTCGGATTTGGTAGGGGGTGCGTCGGCTGGGAGGCTGACGGCCGTGGCTTGGTTTTGCAGGTACGAGAAGAGCTATATGCATCGGGATGTCGTCACCCACGTCGCCGTCTCTCCTGCTGACTACTTCATCACCGGGAGCGCAGATGGTAATCTCTCGCGCTCACGTCTCTGCATCGTACCGTATTCAGTGTGCGATACTAGTGGTTTGTGCCTTTGTGGTAGTTTGTTAGCTTGTGGCAGCATGTGTTAGATTTCGGATGGTATCTTGTGGAAATGTCAAGCCCTTCTACATAAGGGATAGTCCTTCACTGAACCATAATCACGGGCTTGATAGACTTGCGTAATAGTATTGGTTGAAAAACTTTTAGATCTATGGATTGATCAAATCCTGTGGTATGATTCAATAATAAGCTGATTCATTTTCATGTAATGGGAGCTTAATTACCATCCTTTTCTCCTCTCTACCCAGGACATTTGAAATTTTGGAAGAAGAAACCAGCTGGGATCGAATTTGCTAAACATTTTCGATCTCATCTCAGTCCCATTGAAGGCCTAGCTGTGAGTGTTCAGATTCTTATATTGACTTCCCTAATCCAGGGAAAACATTTGCATTCGCAATATATATTAGTCTATTTATCTTGCTAGTTGCTACTCTTAGTCACAGCTCCTGCCTATGGAACTATGAACAACCTCAAATTATAGCGTGATTCAGTAATGCAGGTGAGTGTTGATGGCCTACTTTGCTGCACGATCTCCAGCGATTGGTCTGTCAAGATATATGACGTTGTGAATTATGACATGATGTTCATGATGCGTCTCCCATTCGTTCCGGGTGCAATTGAGTGGGTTTATCGACAAGGAGATGTTAAACCAAAACTCGCTGTTAGTGATCGTAATACACCTTTTGTTCACATATATGA

>Os09t0315050-00 2000 bp upstream sequence

TGAGGCCTTAATTGGGGCTTACCTCAGTACTGCTGGGGAAAAGGCAGCATTTCTTTTGATGAAATCATTAGGGATGAATATAGAATTTCACACTGAAATTCCAGTTGAAAGGAAAATTTCAATGAAAGCTGAAGAGTTCATTGATGTGAGGAGCTTAGAGGGAATGCTTGGTTACAAGTTCAATGATTCTTTATTATTGCTCGAAGCATTGACACATGGCTCATATCAGACTTCTGGCCCTACTTCATGCTATCAGGTTGATATAAAAGCATCTATCATCTCTATTTAGTATGTGTCACGGCTTTATTATGCCTTTTGATCTTTACTCAACAAAGTGCAAATGATTAAACAGTATTGCAACAACATTTTTTTTCTATGTTAGCAATCCTCTATATGTTTCATTTCCTTTTGAGTTTAACTTGCTGTAGAATTATCTGAAACCTGAAAACTGAAGGACTAGCCTCGCTCCTTCAGCGACTCGGGCGGCGCTTCCAGCCGCCGGCCCTACCCCACCTCCTTCCTTCCCACCCCTTGCCGCCGCTGGAGCTGGCTGCCGGAAACCGTGTGGCGGCGGGCCTCCTTTCCTTGGGCTCTCGCACGGACGATGGATGGCAGCTAGATCTAAGCGTGACGTTGGTTGCTACGGCAGCGGTGGGACGGCTTTGGATGGTAGCCAAAGCCGGCAGCGCTAGGAGAGGCTGCAACAGTGGCTGGCACCGGTTAGGCAGGATGCGGGGCTTCTGGGTTGGATCCGCCGGAACGCTGTGGTGATTGCTCGAGTGCAGTCGGCAGAGCAGCTGCATGGGCGTTTGGCGGTCGTCCTAGTCGCAAAAGTGGGCCGCGTGGGCGGTGGTTGAGGCTGTGTTCCTGGCTATTCAAGTTGGCATCAACGGCGACCGCAGCCCTGCCGCGGTGAGCGACTGGATCTGGTGCGTCCTCGCTAGGGGTAGGGATCCGAGCCACGGGGCGTGCTAGCATCCGAGCAGCGAGAGGACTCCTCCCAACCGCTTGGACGTTTTGGAGCTCCTTGTGGTGCATGGATGGTGGCAACAGGGTGGAGGTGCGAGGTACGACAACGACCAAAGCTGGGCATCTGCGATGATCAAGAAGCAGCTGGGTAGTGGTGACGGCGTGTCTTCCATTTTTGCCGGCCAAGCATCTTCCTCTTCTTGGAGCTCCTCCCCTTCTATGGCTGGAGACTGTAGTTTTGGTCATGGGGAGCTTTGATTGGAGGGTGGAAGTTGTGGCTATCATCAGGAGATGTCGATGGCGTGGCCGGAAACGGGGTAGTCCTATGAGCATGCCTGAGCTGTCGGGCTATGGCATGGGCATGGTTGCTAGAGGGATGGTGTGGTGATCCGGCGACTGCCATTGTGCTGCGGATTGGTTGTGTGGGCTGGATGGAGAATCAACTGGTGAAAACCTTGTTGAGCCATTGGCTGGTTGAATGACAGCGTCGCCTTTGGGCACTGTTCCCCTCCTTGGAGGCGCCGTTATGGTGTAGTTCCACCTAGCCCATACTGATCTCCAGGTGAAAACCTTGCTTCAGCTTACGCCGAGTGGTGGTGGCGTTTACACGTCACATCCTCCATGGGTGCGCTGCTGCTTTGGAGAACCTTCCTCATACTGCGGTGACCGTTGATGGCCTCATGCATCGGCTTGAAGCTCTGAAGGCTTAGGATTTTTGATGAGGCCTTTGCCTTTTTGTCTAGCTTCTTTTTTATCTGAATCAAACTTCTCAAGTAGCTGTTGTTAGTTTGGGATCTTTTTTGAGTTGAATGACAAGTTTGTGTCCCTTGCAGAGGCTCGAGTTTCTTGGAGATGCTATTTTGGATCATCTTTTCACTGAATATTACTACAGCAAATACCCTGACTGTACTCCAGAATTGTTGACAGATCTTAGATCTGCCTCTGTGAACAACAATTGTTACGCGCATGCAGCTGTTAAATCAGGATTAAACAAGCATATCCTTCACTCATCATCAGAACTGCATAGGAAG

>Os09t0394300-01 2000 bp upstream sequence

GAACAACCACCGGTGGCTTGACGAGTTAGTGTTTGGGATTTTAGGACCGGGGTTGGGGCCAAGGTTAGGGTCAAGTGGTGGGGTATGGGTCAATCAGTGGAGTTGGATAGTATTGAGAGGCAGTGGCTCGGTAGCAGAACAGACAAGAGAGGATGCATCGGGTGGCCGCCGAAGCCATGGGGTTGGCAGCGGTGTAGTCGGCTAGTTTTGGAACAGGGGCAAAGTGGAGTGATTAGGAGATGTGGTCGATGGTGACAGATCCGACGACAGGGACCACCAGAGACGGAGAAGACGATGGGATGGGCGCTAGCTTCATAACGAGAGACGGGGTGGATTTGGCATAGCCTTGTCGGCGGTGGGAGCCTAGGGGCGACGCAACGGTAGGCAGAGGAAGAAATAAGATGGAGTATGAACCCCAAATTAAACCTGGTGCATGTATCTTAATAAAGCGATCCAACAATCAAGAATATCCATTATTTTTACTGTTGTTTTTTTATTAAATACTATGTAATACGGGAGAGAATACACATGCCACGTAGTAGCTCCGACGGTAAGATTCATCATTCGATCGCAATGACAAATGATTAGCATTACCCACGCGGGTAAACAAACACACAGCTGCGCGGCGTACGTAGTACTACGTAACACGAACGTCATGCAGGGACCGCATCCCAACCCCGGCCATGACCCGTCTGCAAAGCTTTGTTGGCTGCGCCCATCATGTCCTACAGGCCCCCGATCCACACACCCTGCCTGTCAAAACTGTCATCCTCCTGACCAACAGCCCCTCCCTCATCCCCTCCGCGCCATTCATTGAAGCCAAAGCCCACCACCGGATCGCGCCTCTCGTCTCGTCTGCTGCTGCTGGCCGTGGCCGCTCGCTCACTCACCCTCGCGAAGACCATCGCCCGCGCGCGCGTGGGCTCCGGTGAGTCCAACCCTCCAAACATGCCATGTCTCGCGGCTCCGCGGCCTGTCTCTCTCGCGCGCAGTCGCGCTTTGGCCAGTAGCCACCTGCAGCCCCAACCCCACCGCCCCCCGGCTCCCGGTCAAAACCACCACCCCCAACTCCCAACCGCTTGCCCCGCCCAGAAATCCCGCCTCGTCTCGGCCTCAGCCGTGTCAACCAACCAACCCGCCCTCCCCCGCCCGCGCACCGGGCAAAATGCGAGATTTACCCGTCCAAAACGCGTCGTCGCGCGGCCGAAGCGCGCGCGCGCGCGGTGCGGTGCGGGGGGCAGAAGTACTACGTGGCGGGGTGCGCGCGCGGTGCGGTGTATGGACGGCGCGGTGATTAGCGTCGCGTTACGTGCGCGCGGGGCCCCCCCGCCGATCATTTCTCCGCGCACCTGGAAAAACGCTTAAAACGCTTTTACACGCTCGCTTGCCTGTCCGCTCGGTTTGCTTCGCGCAATTCTCCCCGGTTTTTCTTGAGGCGCACAAGAACAATAAAGAGGAGGACGGACGCGCGAACAAAGGAGGAGTACAGGATACAGTCGGAGCCAGTTTAGTACTCCGAGAAAGGCAAAAAAAAAAAACACAGAGAGAGAGAGAGAGGATCAGGAGGACGGCGGCGCGAGCGTGTGCAGCCAGAGAGGCCGGCCGGCGCCCGATCGACAAGCGCGACGCGTACGGCGCCGCCACCGGCCGGCCGGCCATGAGCCGTGGCCGCGCCCGGCTTCAGCCACCACCACCTGGTACCAGAACCACCACCCTCGCCGCCGTGCTGGTCCTTGTCCTCCTGGCCGTCGTAGCGCTGCCACTGCGGTGCGATGCGGCGTCGGCGGGAGGAGAGGAGGAGGAGGAGCAGCAGCCGCTAGACTACAGGGAGGCGCTGGAGAAGAGCCTGCTCTACTTCGAGGCGCAGCGGTCGGGGCGGCTGCCGTACAGCCAGCGGGTGACGTGGCGCGGCCACTCGGGCCTCACCGACGGCCTCCAGCAAGGCGTGGACCTCGTCGGCGGGTACTACGACGCCGGCGACCACGTCAAGTTCGGCCTCC

>Os09t0437500-01 2000 bp upstream sequence

TAAGCTACTATGATATCATTCACAGTGAGGTGTACTACCAATCATAGTATGTTGAACAGACTCTAATAATTGACCGAAACCAATAATTAATTAAACCTTAAATATCTTAGGACTGGACGTGCACGTATTTAGGATGAAGTGTATACGCTGTTAGCCACCACAGTACAACGCATGGTGCCAAGCTCCAGAATTCTCTAAAACCGCCGTTTCTGCATGAAACTATATAGGAGATCTCGGGAGCATTAGCACGAACATTATGGGCCTGTTCGCTTTGATGCCATTTTCAACCTTACCAAATTTTGATAAAGTTGTCAAAAAAAAGCTACATTTAATTTGCTACCAAATTTTGATAACTATATAAAAAATCCTGCCAAAATTTTAGCAAGTTTACCAAAATTTTAGTAATTATACCAAAATTTGACATTAAAGTGAACAGGGCCTATATAGAGTAATAATAGATTCCTAATCCTACCCTCAACTCGTCCATCGATCCACATCCGACGGATTAAAAGCGCCCTTAGTTTATGTTAGCTGCGGCCCGGATAGCGACACGTGGGCCCCGCTACGTGGCGTGCTAGAGTTTGGCGAGCACGGGTGGATCTTATCGGCTTCGTGGAAACTGGTGGCGACGTGGGACCCACCTGGAGAACTAATCTTCGTGTTAATTTTTTTTAAAATTTTTACCGTAATAAGATGCGGCTCGGCTCACCTGTGGCTGTGGGTGGCTCGCTAGCTGGGCTCGAGCCACGCCGTAAGCTCGAGTGTCGGCTCGGTGGCCATACAGTGCCAACTGCCAACTGTGATTCACCTTAGCGAAATTGCAGACCTGCAGTGTGTGCACTCGGAGAAATACTCTCACACAAAATGCTGATTTGTGTACTAGGTGATAGGGATCACACGCTTTCAAGAGTTCTCTTTTTGACAATGTAGGAGGTGAGGTGCTTTTCGTGACCTGGCATGTGCATAGAGTTATAGATTGAGTGGCATGCAGATGCAGGGGATCGAGGGGAGAAATATAGAGTGGTGCGGTACTATGATCGTAGATTGTATTTAAAGTAAAAGTAAAACCATATTATCGTAATAATTTATTTAATTTGTAGGAAATGACGGAAGTGATTTTGAGATAAAATGGTTGACAGTTGTATGCATGTTGTCACGGACGGACTAAAGGCTTAGCGTGATCTTGGACAGCCGCCATGGATTGACTGTGAGCTCCAAAACTTTAGATGACATGTACTCCCTCCGTCCCCCCCAAAAAAACCAACCTATGAAGGATCACATCCCCTCATAGTACAACAAATCTGGACAAAGTTATTAGTTGTACTAGGAGGGGTCACATGTTGGTTTTTTTAGGACGGAGAGAGTATTTGCTAGTGTCCCATCATGTATGTATATAGACTAGGAAAATATGGATCTTCAAATTTTACTAAACTTCAGAAATTTTATGCATTTATCCTTTCATAATCCTACCAATTTATCAGCTTAAGGGTCCTATATAAAGAAAACAACATATAAATTAATCCAATAGAGCTATTTTAAACAAGTCCTTTAAAATTCCCATAAAATATGCTATTTTAGATAAACTTTGGAGGCAAATAAGTACGAGGTTTCACCTCATATTTTCTTCTGAGTTCTTGTGTTTTCATACGTGCCAATCAAATGATTATATTTAATTTGTAACTTTTATGTGTCTTAAAATTGTAGAACCCAATGAGCATAATATTTTATTACTATGTATTTTTTTCAATTAATGTATTTTTAACAATTAATGCATTTCGAAGCTGCTCTTATCATGTACATGGACGCAGACTCGAATTGTTCATGAGCCGAGCTAAGCCGTTAAGGCGCGGATCGAGGCATGGAAAAACCAAAAAGACAAAAAAAAAAAAAGGAAAGTGACAAGCCAAAATTACACATGCCTCCCAAATATCTCGCCGAAATGCTCTCTCCACCCCCAAGACTCCTCTATAAATCCACTCCCCTCTACCTCCTTCCAACAA

>Os09t0455400-01 2000 bp upstream sequence

CCCACTCGTCGTCCTTGCCCTAGGCCTCCACCCCGCTGCACTGCGCCATCGACGCCTCTCCTGTCTATGAGGTTGCATCTGCCTCTTGCTCCTTTTTCATCGATCGTTGCCCCTGCAGACCAACAGAGGGGGGAGAGAGAGAGAAGAAAAAGTGGTCATTATAACATGTGGATCCCATGTGGGTGTTACTATATTAATTAATTTGTTTAGCTGATATTAATACCGGATAGATGCCACGTTAGACGAAGACAAAATCAATTTGATTAGTCAAAAAATAACAAAATCAATTTGACGGGTAGGCACCACGTAGGACAAAATCATCCTCTAAACTACTAGAGGAGTCAAATTAAATCGGTTTGAATAGATGAGGGACTCATATACCTGATTTCACGATTGAGAAACATGGATAAGATTCGGCCAATAATTGAGGGAGTTAAAATGAGCTGTTTACAATATATGAACTAGCTGCCTCCTCTATCGGCCTATGCTAAGGTCCATAACTACCCCTTGCCTTCTTTCGGTCCATCAAGCCCAATAACGCACACATACCGCCGATCCAAATTCCAAACCGACTTCGCCTCCGAGAAGCCCTCACCTCCACACTTCTTCCCCCACGCGTGACGCGTCCAACCCGATCTCTATTCTCTAGCTTCCCCAAATCCCCAATCCGACCTAAGCCGGCGATCCGCCGCCATGGGTCGCACGGAGGGCCGCCTCGCGTCCGTGGTGCACCTCCCCGGCCGCTCCAGGGTCTCCGCCTCGCCCTCCCCTCGCCGCCGCCGCTCCCCGTCCCGGTCGCCCTCCCCGCGCCGCAACCGCCGCCGCGACCGGTCGCCTAGCCCCTACCGCAGCCGCAGGGACCGGTCCCCTAGCCCCTACCGCGACCGCCGCCGCCAGTGGTCCCCCTACCACCGCGACCGTGGCCGCGACGTGGAGAGGGAGTGGGCTCGGGACCGAGACCACCCTGCGCCCCGTCGCGGCGGCGGCGGCGCGGGGGCCGGCGCCTGGTCCGCCTCCGACGATGACGACGACGAGCAGCTCAAGGGTCTCACCTACTTCGAGTACCGCCGCGTCAAGAGAGAGAAGCTCCGCAAGAGCATGAAGCGGTGCATCTGGAACATCACGCCCAGCCCTCCTCGCCGCGAAGGTGAGGATGAGGATTATGGCTACAGCGACGAGGAGGAGGAGGAGGAGGAGAAAAAGGAATCGCCAAAGAAGGTGGCCTCCTCGGATAAGAGCGAAGAGGAGGATAGCAAGGGTTCGTCGGAATCCGACTCCGGTGAGTCTGATAGCTTGTCCGATTCCAGCAAATCAGATGATACCCGGAGGAAGAAGAAAGGGCGTAAGGGTAGTCACCGTAGCAGCAAGCGTAGCCGCCACCGCCGCCGCCACCATTCATCTGATACAGAGGGTGACGACAACAGCAAGGCTGAGGAGGATTCGGAGGGCTCTTATGATTCCGAGGACTCTATGGATAGGAGGAAGAAGAAGAGATCGCGGAGGCACAAGAAGTCTAAGAGGAGGGGAAGGAGCTCCAGGAGGAAGAAGAGGAAGAGCAATGATACAGCTTCTGAGGGAAGCTCTGAGGAGGAGGCGGTGGCAGCGGCTTCAGGATCAAGCCCTAGCCCCTTGAGGGACAGTAAGAAGAAGAGCCGGAGCTCACGGAGGAAGAGGAGCAAGCAGTCTGATTCGGAAGATCAGGCCCCATCCGATGCCGACCTTGGTGTTAAGGAAATCGATGAGACAAATGAGCCAGAGATTGACCCAGAAGCAATCAAATTCAAGGAAATGCTCGAGGCCCAAAAGAAGGCTGCTTTGGAGAATGAGATGCCGGTTGGGCCAATGCCTCTTCCTCGTGCAGAAGGTCATATTAGCTATGGTGGTGCACTGAGACCTGGAGAAGGTGATGCTATTGCACAGTACGTACAGCAAGGGAAACGTATCCCACGACGTGGTGAGGTTGGTCTATCTGCAGAGGAGATTCAGAAGTTCGAGGATTT

>Os09t0543100-01 2000 bp upstream sequence

TAGATGCTACAACTAATTAAAGTAAAGCTGATCCGGAACCTCCTCTGGTGCATGACGGTGTGACACATGGAACCTCCCCGTTCTTCACGGCCTCCACATGGTTTGTCCTGTTGTACAGAGGGGCAAGAGATCGAGATAGGACTTTATAGGAGGAGTAGTAGCGAAATTCTCCTGCTCAAGCTGTTAATCAACACAGGTACTAAGCTGATATTGTCAGTCAAAGGAAACCGAAACTCTGACGTGCGTGAGCAATCTATTATCCATTCTTGATTCACACACATATGATATGACACAGCTAAGCTAAAGTTTTTGTGGGCAGGAAACCGTTCGAGTAGCAATGAAAGTTGCATCTTAAATTCTTGATTAATCTGTCTAATGTGTAAGGGGAGATCGACATGAGCACTTGAGCAGGAAGCAGCGGCGTGAAGCGAAAGGGAGAACAAAGAACCCAATAATTATATGCTTAGTGACGGGCCACATCAGAATATGTAATCAATTAATGGAATGAAGTCGTGAGCCTAACGACGCTGGTAATCGCACGATGGCACAGCATTACAGCAATGCAATGCAAAGCTGTGTGCGAGAGATCGTCGTCCTCGCTGAATTGGTGGCCGCGAGAGCGGATCGTGTCTCGCTGCCGTCCAAGCGTTTAGTAATTTTGGCGATGATTAGTACGTTCCTACATGTTGACGGAATCGCATTAGCTGGAGGTATAGTACGGCTGCTTCAAGTAGTGGAGTACGTGCTCATGTGATCTCTGGGGCGGTTCGGTTCAGCTTCGATCCTGACAGGGTGTCCGGGCTTGTACTAGCGTGTAGGACAGGACATGCTTCATTTGCTGCGAGTACTGTGTTAGGCCTTGTTTAGATTAAAAATTTTTGGTCAAAAACGTCGTATTAAATATTTGGACACATATATGCATGGGACATTAAATATAAGAAAAAATCAATTTCACAGTTTGCATGTAAATTGTGAGACGAATCTTTTGAGCCTAATTTCGCCATGATTTGACAATGTGATGCTACAGTAAACATTTGCTAATGATAAATTAATTAGGCTTAATAAATTCGTCTCGCAGTTTACAGGTGGAATATGCAATTTATTTTATTATTAGTCTACGTTTAATACTTTAAATGTGTATCCATATATTTTAAAAACCTTACGTCCAGAGAACTAAACACACCATTAGCAGCATTCGTCCCAAGGCGAGACATTTTATGATGGTTAGGTTCAGCATCGCTTTCAGGTGACCCCAACCAGAGAGAGAGAGAGAGAAGTACCACCACCATCAGCTCGTGACTGAGCATCACTTTGGTCCGCTTGCACAAAAAGGAAGCCCACTTGCTAATTCCAGATCAAGAATCCAATATGTGTTACCTATAGCAGCAGTTTCATGTAGAAGTAGAAGTAATTTCTCCGGCCGGCCGAGCCGGGGGCAATGAAACCGTCGAAACCGCCCGGACAAATCTGGCGCCGCACGTTAAACCTACCCACCTCTCCTCTCCTCTCCAGTTGCAGCTCGCCGTTGGCGGTTGGAGGAAGACGACGTCACCCACCTTTTCCCCTTTTTTCCCCTCTCAGGCTGTGTTTAGATCACATCACAATTAAAAGTTTGATTAAAATTGGAACGATGTGATGGAAAAGTTAGAAGTTTGTGTGTATAAAAGTTTTTATGTGATGAAAAAATTAGAAATTTAAAGAATAAATTTGAAACTAAACACGGCGCCAACTGCGCTCCCGCACGCACGCAGAATCGCAGGAGAGAAGTTGGCTTTGGTGCGGAAACGGACACCTCTCCCGTCGCAAGCCACCTCACCCTCACCTCGCCGCAAGCCGCCTCCCGCCTCGTTTGACCCCCGCCGCGCGCCACGCCACGCCACCGGCCACCACGACGACCGATCTGTTCATGCGTTTTCCGATCGGATCGCGTCGCGCCCCCAGCTCAGCCGCGTGGTCGTGCTGGGCCCCGCGTGCCAGCGACCGAGTGGCCGGGAGCCATA

>Os09t0543100-02 2000 bp upstream sequence

TTCTTCACGGCCTCCACATGGTTTGTCCTGTTGTACAGAGGGGCAAGAGATCGAGATAGGACTTTATAGGAGGAGTAGTAGCGAAATTCTCCTGCTCAAGCTGTTAATCAACACAGGTACTAAGCTGATATTGTCAGTCAAAGGAAACCGAAACTCTGACGTGCGTGAGCAATCTATTATCCATTCTTGATTCACACACATATGATATGACACAGCTAAGCTAAAGTTTTTGTGGGCAGGAAACCGTTCGAGTAGCAATGAAAGTTGCATCTTAAATTCTTGATTAATCTGTCTAATGTGTAAGGGGAGATCGACATGAGCACTTGAGCAGGAAGCAGCGGCGTGAAGCGAAAGGGAGAACAAAGAACCCAATAATTATATGCTTAGTGACGGGCCACATCAGAATATGTAATCAATTAATGGAATGAAGTCGTGAGCCTAACGACGCTGGTAATCGCACGATGGCACAGCATTACAGCAATGCAATGCAAAGCTGTGTGCGAGAGATCGTCGTCCTCGCTGAATTGGTGGCCGCGAGAGCGGATCGTGTCTCGCTGCCGTCCAAGCGTTTAGTAATTTTGGCGATGATTAGTACGTTCCTACATGTTGACGGAATCGCATTAGCTGGAGGTATAGTACGGCTGCTTCAAGTAGTGGAGTACGTGCTCATGTGATCTCTGGGGCGGTTCGGTTCAGCTTCGATCCTGACAGGGTGTCCGGGCTTGTACTAGCGTGTAGGACAGGACATGCTTCATTTGCTGCGAGTACTGTGTTAGGCCTTGTTTAGATTAAAAATTTTTGGTCAAAAACGTCGTATTAAATATTTGGACACATATATGCATGGGACATTAAATATAAGAAAAAATCAATTTCACAGTTTGCATGTAAATTGTGAGACGAATCTTTTGAGCCTAATTTCGCCATGATTTGACAATGTGATGCTACAGTAAACATTTGCTAATGATAAATTAATTAGGCTTAATAAATTCGTCTCGCAGTTTACAGGTGGAATATGCAATTTATTTTATTATTAGTCTACGTTTAATACTTTAAATGTGTATCCATATATTTTAAAAACCTTACGTCCAGAGAACTAAACACACCATTAGCAGCATTCGTCCCAAGGCGAGACATTTTATGATGGTTAGGTTCAGCATCGCTTTCAGGTGACCCCAACCAGAGAGAGAGAGAGAGAAGTACCACCACCATCAGCTCGTGACTGAGCATCACTTTGGTCCGCTTGCACAAAAAGGAAGCCCACTTGCTAATTCCAGATCAAGAATCCAATATGTGTTACCTATAGCAGCAGTTTCATGTAGAAGTAGAAGTAATTTCTCCGGCCGGCCGAGCCGGGGGCAATGAAACCGTCGAAACCGCCCGGACAAATCTGGCGCCGCACGTTAAACCTACCCACCTCTCCTCTCCTCTCCAGTTGCAGCTCGCCGTTGGCGGTTGGAGGAAGACGACGTCACCCACCTTTTCCCCTTTTTTCCCCTCTCAGGCTGTGTTTAGATCACATCACAATTAAAAGTTTGATTAAAATTGGAACGATGTGATGGAAAAGTTAGAAGTTTGTGTGTATAAAAGTTTTTATGTGATGAAAAAATTAGAAATTTAAAGAATAAATTTGAAACTAAACACGGCGCCAACTGCGCTCCCGCACGCACGCAGAATCGCAGGAGAGAAGTTGGCTTTGGTGCGGAAACGGACACCTCTCCCGTCGCAAGCCACCTCACCCTCACCTCGCCGCAAGCCGCCTCCCGCCTCGTTTGACCCCCGCCGCGCGCCACGCCACGCCACCGGCCACCACGACGACCGATCTGTTCATGCGTTTTCCGATCGGATCGCGTCGCGCCCCCAGCTCAGCCGCGTGGTCGTGCTGGGCCCCGCGTGCCAGCGACCGAGTGGCCGGGAGCCATACAGGCGACCGTTACGTCCCACCACCATCACGCTCACCGCTTCGCGATCTCTTTTACGATTAACCCCTCGCAGAATTAGAT

>Os09t0572700-02 2000 bp upstream sequence

GTTTGAAAAGCGTGCGTGCGGAAAACGAGTGAAATCTCCCCTATCTCTCCTGAAAGAACGCAGCCTTAGGCTGTGTTTGAGGGAGAGGAGATTGAGAAGATTGGGAAGATACGGAAAACGAGGTAAGCCATTAGCGCATGATTAATTGAGTATTAACTATTTTAAACTTTAAAAATAGATTAATATGATTTTTTAAAGTAACTTTCATATAGAAATTTTTTGTAAAATACGCACCGTTTAGTAGTTTGGGAAACGTGCGCGCGGAAAACGAAACAAACATTCCCCCATTTGTTCTCCAAACGAACGCAGCCTTAGTGTACATCGAATTGTCGTCAGCAGAATTAATTTGAATGATGATCTATCAATGGATCGATCTGATTAGTTTAGTTGCGTAATACGCCACAGGCCAGCAGAGTAGTCAACTGGCAGCTAACATACGTGTCACATGACCAGTTAGCCGCAGTAGTCTTGAGCCGCCAATTTGGAACTCTCTTGTGGTACTAGTATAAATTAATTATATCGTGTGGAAATTATTAAGATGAGACTGCAGCAGGCCAACAACCTGCAGAATTCTTCTTCTTCCTCCAATTCTTCTTAGCCTGGGTTTATTTCACGCTTTCAATTTTAGTTGAAATTGAAACGATGTGACGAAAAAGTTAGAAGTTTATATGAGTAGAAAAGTTTGATGTGATAGAAAAAGTTAAAAGTTTGAAAAAAAAATTAGGATCTAAACACGGTGTTACGCTTTTACGCGTACAACGACCGGCCACCTGCAGTACATGCTGACCATGTAATATACTATAATTTGCAATTAAGCAGGCAAGCAGGTGTAAATAAGCTTAATTACTATTTGAAAATTATATTATTACTTAATTGAAAAGAAAACAGGTGCTAGTTTAATTACCAGCTCCTCCTCTTGACGACAGCAGCAAAGGTGTGGCCGAGGCGGCGAGGCACATAGATAGGGGCGGGCAAGCTTGGCTTGGATCGATCTTGAGTGGATAGATCAACTGGAAGGGCGTAGCGGCAACGGCGTTGGGCGGGACCTAAGATCTGACATGTGTACGTGGCGAAGGGCAAAAGTACACGGCATTGGAATGGATGACATATTATCAAATTTCTCGCAAATGATCGAGCAACCTAAGAGACAAAGTCAATCGAATTGAAATGGTCCGCGTATAGTGGAAGTGGGATTGCACTATACTCGTATTAGGTAGATGCACGGACGGACGTAATACAAACGCTAGTTGACAGTTCCAATTAATCGTTCATGCGACCCAATTGATTTAGTTCTAAACTTTCGCTCCATACTACAACCTTCTCATTTTTCTATTATCACGTTTTCTTAACCGCTAAACGGTGGATTTTATATAAAAGAAAAAGAAATACTCCATCATTTTACGTTATAAGATATTTTTGACTTTGGTCAAAGTCAAATTGTTTCAATTTTAACCAAGTTTATAGAAAAAAATAGTAATATTTTCGACCCAAAATAAATTTATTATAAAAATACATTTAATTATTAATTTAATAAAATTAATTTGGTAATATAAATATTATTATAATTGTTTATAAACTTAGCTAAATTTAAAATAGTTTGACTTTGACCAAAGTCAAAACATCTTATAACCTGAAATAGAGGGAGTATATTTATAGATTTTTTTAAAAAAATAAATCTATTTTCTAAATATATAATAATTCATACTTACTTAATCAAGCATGCGTATATATTTAGAAATGGTCTGATGGATTGAGTGAGTCGTACTTAAGGACTAGAGCTATGAGCAGTGTGAACGGATAGACGTGCCATCACAAATAATATAATGTATTTATTTTTTTATGAAGAAAAAGAAGGAGCAAAAATGAAAAATAAGAAAAAAAGACAATAATAATTAGTAGGAGTACCTACCAACCGTGGACTAGTATTACCTGCTGACCAACCGACCTTGCAAAGGAGGCTTATATATATACTCCAGTAAAAGCTGCCTGTTTCATCGATC

>Os10t0113000-01 2000 bp upstream sequence

CAATTGCTAATTTTACTTTATCTTCCATTTCTAATTTCAATTAATCTCATACTGTGTTTACATCTATTCTTCGTTCCACAATTCAAATTTCAGCTATTTATAAATTTTACTTTTACAATACTCTGTCGTTTAATATATTTACATTGTTAATAACATGTAGTACTCACTCTAGAACTACGGTTATAGATTTCACTCTATTTCGTTCTCTAGTTAATATCACAACATTTTACACGTTTTCAGATTATGGCCTTATTTAGTTGGAAAAATTTTTTGGGTTTGGTTTTTATATTGGATATATGAACACACATTTGAAGTATTAAACATAGTCTAATAACAAAACAAATTACAGATTCCGTCAGGAAACTGCGAGGTGAATTTATTAAGTCTAATTAATCCATCATTAGCAAATATTTACTGTAGCATCACATTGTTAAATCATGGCTAATTAGGCTTAAAAGATTTGTCTTGCAATTTACACACAATCTGTGTAATTGTTTTTTTTAGTAAATTGCGTTTAGGGCCACCGTTTATTACCAAAGCTTCACTTTATACCACCATTTAACACATTTTTTCACTTTGGGCTAGGTTACTTTACCTTTATTTTACTTTGGACCACCCCATAAACTTTTTAGGAATACATAAATGACTTTTCCAACTTTAAACACATCAGCAGCCCGTAACCCGGTAATATTTTCCAACTTACACAGGCAATTGTTTACAATATTATAGAGTAGATGCAGCTAAAGAAAATGACTTGGGCGGTCCAAAGTGCACCAAAGATAATCTTACCTAGTCTAAAGTAAAAATATGAGCTAGAGGATAGCCTAAAGTGAAACTTTGGCAATAAAAGGTGGACCAGAGTGCAATTAACTCTTGTTTTTTTCCTACATTTAATACTTCATATATATGTCCAAACATTCGATGTGACAGCGTGAACAATTTTGTTTTGTGACTAAGGGTGTGTTCGGACCCAAGGGTTCCCAACACCTCTCCATCGTTTTCCGCGTGCACGCTTTTCAAACTGCTAAACGGTGTGTTTTTTGTAAAAAGTTTCTATACAAAAATTGCTTTAAAAAATCATATTGATTCATTTTTGAAAAAAAAAGCTAATTTCTAATTAATCACGCGTTAATGGACCGCTCCGTTTTCCGTGCGAAGAAGATTTGTTCCCAACCTCCCCAAGCAAACACAGCCTAAACAGGGCCTAAACCTCGATGCGTTTTTATCATTTCTGAATAAGGTAACTAATAGATCGTCATAGTCTTCGTCAGACTTCTATCTCGATCGCTTTCAACGGGGCACATCAGCCTAGGCGGCTTTGGGTCATGGACATGTACAGGTAGGTGCAACTAATCATACATTTTGCAAGTTTTCATGGACTAGTAAAATAGAAAAGGTAAAAATGAATTAGTCTTGTGTTGGTACTAACTAACACAGCATTTGCAAACCTGTAAGCTACCGTAGCATATATAAATAATTGTTTAAGATTTATCCATGCCACTAAATAGTTTATTCCAAAAAAAAAAGTTACTTATGGTTAATCTTGTGGCAAAAAAAATTACTCATGATAACAAGAACAAATCTCAGATGCTCATTTGTTTATCCCATGATATTTCAGGTTTATATTGCAAATGAACTTTGTACCGCATATTGATCTCTTACTAACTTACCCATGCAGGGGCGGAGCTAGTATATATTAGGGGGTGCTCAGGAACCCGATAAAGAAAATTTTTTAAACTATACCTTATCTATTTTCACCGTATATGTACCCCCTTAATACAAACTTAGACATTTGTATTAGGTTAAACTTTAATCTAAAGTAAAGTAAATTAAGAGAGTAACACCACATTCTGTTTCGCTCTAGCTCTATCCCTGCCCATGCTTCACCTAGAAAAATGTTACCTTCATCGTCACTTGTGTTGTTTAAGTCTTGCAGCACGAAACCTCAAGGAGGAGATCCATTCTTTTCCTATATATAGCTACCCACAGAGAAAAACCTC

>Os10t0442900-01 2000 bp upstream sequence

TTTGTTACTAATATATTTGTGGTTGAAACTTACAAGTACCCAAAACAAGTTTTTTCGTAAAAAAATATGTGCTAGTATTAAACTTTTGATATGCTAGTATTAAACTTTTGAAGTATCTCAGCTGGACAAATGTGTCAACAAATGGAAATATACAATAAGTGGATGCTATTTATTTATTCCATTCATTGACAAGATGAAATTGGTCATGTACATTTGCTTCCAAAAACCGAGTTTCTATTTTTTAATAAATACGACAAGGACCTTATAATATAGAAAACACCACTAAATTCTAAGAGGTTTTTGTTTTGACAAAAGGCACTTGGGATCTGTAATACAGGGGCTGTTCAGATTGATGCCATTTTTAACCATACAATTTTTCGGCAAAGTTACCAAAAATGTGTCTACGTTTATTTTGTTGCTAAATTTTGGTAAATACATAACAAATCTTGCTAAAATTTTGGCAATAGTGTCAACTTGCCAAAATTTTGGTATTGCCAAATTTGGTAAGGTTTATTTTGGCTATAATCTGAGCAGGCCTATAGGTTTTATCTGTAATAAAAGTTGTTTCTGTAATACAGATAAGTTCATAAGTGCTTATAGTTTAGGAGGGAGAGAATACTATCTTTAATAAAAGCTGTGTTTTTCGAGCCATTTTAATATTTAAAAAATGACAAATGTTTCAAACCGTAGCGAATGCATATGCATCACAAATTCACAAAAACTGTGGAATATCAACTAAGATTGCCACGGGCCTCATGAATTCATCGGCCCATGGGCCTAAGAAACCAAAATTTGCTCCTTATCAGTAGGCCTCTCCCTGCAATCCTGCCCGTTAGGCCTAATCTATTGCTTTATTCTCTCCTTTTCTTTTTCCCCTGCGAACGATACTACTACTGTTACTTTCTTTTTCCTGTGAGTTAGTTGTATCAAGCTCACGAACAGTATATTTTGAACAAACAAACAAACACCTTTTTTTTTTAACGAACCGCTCACGAACGATATGATAATCAACGTACGTCTGCTTAGCCGCCTTGTTCTTCTTGTTAGTTGATGCTTATTATTTGAAGTCTTTTCTTAAAAAAAAAAAACTTGATATGCTGCTTGTTACTACCTCCGTTCCTTTAAAAAACATAGTTTTAACTAATAGAAAAGATTAGTAAGAGGTTTAAAGATTACTATAGCTTTCATTAATATGATGAGAAATAAAGAGGGGATAAAACAAAAGGAAAGAACGAGTACTACTGATTTGATGGTTGAAAATATAGAAAAAAAATAAAGAAGTATTAAAAAGATATAGCAAACCCTACTTTAAAAATAAACTCTCTTTTAGGAACGGGTGGAGTAGTTGGTGCTTCATGTTTGACGATTTTTAATTTTCGAAGAAAATGTTTGACTGATCGAGCTAATTAATCCTGAGCTAAACGGAAAAAAAAAAACCAACGATTAACTACGCGAGTATGACATAAATCCCGTGGGCCAGCTGCTCAGATAACGACTTAAAAATAAGTTTAACAGAAAACTATAAACCAACTATATGATTATAATTAGGGACAATTATGTAGAAGAGGGAGAAAAATATAAAGAGCTAGCACACATACTCCAAAATAAATTTATCGATTATACAAGTATAAGAAAAAGAATTAAACATAACATTATAGCTATTCTATATTTCTATTATACATATTGCTTCTAACTTTATTTTTAAATTACCTTATAGCTAGTTTTTAGTTCTGCCCTAATCACATCATCCTGCTGACAATTCCTCACAGAATTTAAGCACTTTTAGTCCTTCACTTCATCTTTTTTAAAAAAAAAAGCAAAGCAAAGAAAATGTCCACGCGCGCGTTGCCTTGCCTTGCCTAAGCTCGCGCGACACTCTGACCGTGTCTCGTCCGACGACTGCACAAAGCCACGGTCGTTCACGTAAGCTACTACCACAGCCTAAGCTAAGCTCGCCTTGGCCTCGACGGGGTCAGTGATCAGTGCGTGTCACCTACCAC

>Os10t0560000-02 2000 bp upstream sequence

GGTTGCTTGAATGCAGGAGGAATACATATACAATACAAACATACAGGTAGTGTACCTTCCCGTGAGGCTGGGACCCGTTCATCCAACAACCATCGACATGCATGCCAAACCCCCAAGGTCTCAGATAGCTCAACTGCCGGGAACAATTAGCTAGAGAACAGCAACACAAGCAGTATACTATGCTGCTAATTTGCTAGTACTAATATACCACCCCACAGGTAGTGGAAAAACTTGGAGACAGGGTAGAAAGGAAAGCCAGGGACCTGTCAGCCTTGTGTCCCCTTTGTTCTTTCTGCCTTCCTCAGCATGGCGGCTCCACACCCCTCTCCTTGTGCTTTGTGTGTGCCTCCCTCCTTCAGTATAAGTACAAATGTAGTACTCCAGTCACAGGCAACCAAAGAACCATCACTTGGTACCAGTAGTGCAACAACTTGTTCAGTCGAGAGGAATACCTTCTAGCCTTCTAGTAGCCAGGATGGCCATGGAGAGAGCTTGGAAGCCAAGGGAAATTGCTGACACTTTTGGCATTGAGATGGATGAAGAGGAGGCTGCGGCTGCGGCGATACCGCCTCCGCAGACGCCATTGGAGCCCATGGAGTACCTGTCGAGGTCATGGAGCGTGTCAGCGTCAGAGATATCCAAGATCCTTTTCAACGGGAGCAAGAAGAGCTTTGCCGCCAAGCGTCTGCCGGAGATGACCATACCGGAAAACTCTGTGGTCGCTGCGTCCATTGTTCCCAGCCATCTGCAACATGTAATCAATCAACAGCTCTCTCCATCCCTTGTTTTTCCTCCTGTTCTCCTCATCTGCAAGATAATCAGAGTCAGAAAAGAAGCAATGGAAGAACATAATTTAGATAAGTTTGAACAGCATGGTCTAATCATTTTGAATCTCGTTTATACAAACGTTTCTGCAGATAGACACAAGAAGGAATTCAATCAGCAGCCACCACCTGCCAATTGGGAGGTGGTTCCAACACAAGGAGGCAAGCCGGGTCAAGCAGAGCAGCAAAGAGAAGCTGCGAGCCGAGAAGGCCCATGTACACGCCATGGTGTCGGTGGCCCGGGTCGCAGCTGCCGTTGCTGCGGTTACCGCGGCCACCACGAGCTCAGATATCCAGACCTCCAAGATGGCCGCGGCCATGGTGTCGGCCACCGAGCTATTGGCTTCGCACTGCGTGGAGATCGCGCAGCACGCGGGGGCACGCCATGAGCAGGTGGCCTGTGCCATCCAGTCTGCAGTTGGTGTTAGAAGCTCTGGTGATCTGATGACACTCACAGCAGCAGCAGCTACTGGTATGGAATATTTGATATCTTGATGAAATCTATATATGATGTTTCATATATAAATTTAAGTTCACATGAACATTTGGTTTCACAGCTCTTCGAGGAGCCGCAACAATGAAGCAGAGAGTGCAGCGAGAGATGAGAAGCAATGCGAGTGTTCTTCCGTATGAGAAGGGTCACTCATGGAGTCCTGACATCTGGTGCAAGGAGGGGGAGTTACTGAAACGCACAAGAAAAGGTAAGAAAAAAGGGCACCATTTAGAACACCCATTTAGGAATAAAAAACCATCAACATGTGGACTTAAGCATGATCAAAATAATTAAGGTTCAGAATTTCATTCTTGATGCAGGAGATTTACACAAGACACGAGTATCTATATACATCAACAAGAGGTCACAGGTAGGAATATTTCATTGCACACTAGCAAATGCTGAAAACCTAGCAGACAGAACTGAGCAATTACTAACAACATGGCCAAATAATAACAGGTCATATTGAAGTTGAAGAGTAAACACATAGGAGGAGCACTATCAAAGAAGAACAAGAGTAAGTTTCAGTACCTGCAAAGTAGTATATCTTTCTGACTCTAACAGAGTGTCAGTCCATGAAATTTCAGAACTGAACATTCTGCCTGTAATTGGGTGCAGGTGTGGTTTTTGGTGTGTACAACGAGCTTCCCACATGGGTTGAGGCAGGGAAACATTTCACAGAG

>Os10t0572300-02 2000 bp upstream sequence

CCCTGTCCCCGTGGTGTGATGTACTATATATAAGCCCATGTTTAGCATTGCTGACGTAGCACAAGTTGATAGACAGAAAAGAACATGGGACAAACTCCAACAGAGTTTCCTCGTCCACTAATCTAACCTACGTCACTAATTTAATCCACTCTAGTAAACGAAGGCACTCTAGGAGTAATACAATCTACTCTAGCGGAATAGGAGTACTCCCCTGTAGAGCACGCAGCCTTGGGTAAACTCGTGAGGGCAGGTACAATAGCAAGCTATAAGCCAGCTGTAAATATATTTTAAGGGGTTCTTTGAATCAAAGGATTTATGTAGGAATTTCATAGGATTCAAATCCTATGGGAAATTTTCATATTTGGCCCTTTGATTCAAAGGATTGAAGCTTTCCAAATCCTATGAAATTCCTATGGAATGGCACATTGCATGTGGATTTTGGAGGAAATTTAGCAAGAGCTCTAACCTCTTGGAAAATTTCCTTTGAGTCTATCTCTCTCATCCGATTAGAGATTCCTGCGTTTTCTTACGCTCCAATCAAACGACCATTCCTATGTTTTTCCTGTGTTTTGCAATTCTTTGTTTTACACTTCAATTCCTGTCAGAATCCTGTGTTTTTCCTATTCCTTCGTTTTTTCTACTCTGCAATTCAAAGAGGCCCTAAGAGATAAATGAAGAGAGAGAAGAGCAGCGAGCTACAGATTTATAGCCAGTTGTAGCACGGACTCTAAGACACAGTGTGTGTATGACAGGTGAGACCAGGTATTAATAGCATAGTATATAACTATTATGAATGAGCTATTAGATTAGTTATAGATGAATTAGAGCTAGTAGTTGGCTATACTATTAAACTTGCTCTGATACCTCTACCCGTACTAGTAGGTCAATAATTAAACTATTCCTACATCAATTAATTACAGTAATTTGGGTTGGGACTTGGGAATTACTCCTGCAATATACTCCCTCGTCCACAAAAGTTATACATATTTCACATTTAAGTTTTTCCAAATAAGTTGTTTCTATTTATAGTCTTTATACATTTAAGATTTAAATGAAAAGATAAATTAAATGTTTTATTAGAACTCAAGGAGTCATCTAAATACTCATTGGTTGTATGCTTGCATTTACTCCTTGATTTTGTAACATTCAATGTGATTTAATTTTTTCTTGGTCTTGGTGACAAAAGTAATATGGATAATTTTGTGGATGGAGAGAGTACTGTACAGTCGGACACAGCGGTGACCACATGGTGGGTAATCTTGGGAAAAGGTGGTCGTTCCAGATCCTACCTGACACATCCCGATGCGTTGCCAGCTCATCCCGTGGGCCCCACCCAGCCGCCTAGCCCATTTTGCGCTTTACCCCCTCTACTTCCTTCCCAATCTCAACCCCACCCTCCTTATACCGAGAAGACTTTTTCTTCGCAACCGGGAGGGACCCTCTGGTTTGCTGTTTTTATAAGGCGGACTCCTCAACTCCACTCCCACTTGCAGTTGCACCGCATCTCATCTCATTTATTACCAACCCACTCCTCTCTCTCTCTCTCTCTCTCATCTCTCGTCGTTTTCTCTCTCCTCGATCGAGGGCACGCGACGCGCCATGGCGTCGGCGGCGGAGGCGGTGGAGGAGCTCACGAGGCTGTACCGGGAGCTCCCGCCGCGCCCCGCCGTCGAGGAGGTGGAGGCGGCGGAGGCCGTGCTGGCCTCGGCCGACGCGGAGGAGGCGGCGCGCCTCGATGAGGTGGCGCGGGAGGAGGCCTCGGCCTCAGCGTCCTCGTCGGCGGCGGCGCCGGGGCGGGCCGACGGGGAGCTCCTCGCCGTGCTGCGGGAGGCCAGGCGCAACGCCGTGCGGCTCCGCGCGCTGCAGCAGCGGAAGGAGGCCGCGTACGTCGTCGAGCTCGAGAGGCGCTTCAAGGTGTTCGACGATTTGATCCAGAGGGCGTCGCGGGTGGTCTCCTCCTCCTCCGACGCCGCCGAAGCGGGAGGGGGAACCACCGGCGA

>Os10t0580900-01 2000 bp upstream sequence

TCTTTGTATTTCTCTTTGCACTCTGTGCTTAGTATATGACTATGGGCCTATTCAGATTGTAGCAAAAATAAACTTTACCAAATTTTGGCGATGCCAATACTTTGGCAATTTGGCAATATTGCCAAGTTTTGGTAGGATTTCTTATGTATTTACTAGAGCTTGGTAAAAAAAACTAAGTGGATGCACATATTTTGCAGCTTTAAAAAAAAATTGGTATGGTTTGAAATGGCATCAATCTGAACAACCCCTATATGTATATGTGTTCCTATGTTGTGGTGGGTGGGTTCACTTCATATTGATAGCGCATGTTTATTTGATGTTTCGCCACATCCTTTGTGGCTAGTAATTTAGGGTGAAGTATCGTAGGTTTTGACGATACTCAACATTTTGCATGAATTTGATATTTCTTGCCATGTCATATACTGATTGCTTTTGTAGAGAGAGCTTCAACGAGAATTGGATCAAGTTGAGTTGGGGCGGTCATCAAGGCGGGAGTTTTCAGCCAATACTAACAGGTGTGTTATGCTGGTAGGCGGTTGCCTCATTGTCTCACCATAAATAAATTTTCATACTGCTTCTGATGAAACTTCACTTGACCTGTTTGTTACCCTTGCCATGTGTGTGATGTGACGGGTGTTTTTGCAGCCGGTCTAGGGAGAGATACCGTGAAAGGGATGACGGTAGGGGACAGCAGGAAGGAAGGAGCCGAGGTGGAGGGGTTGAGGCCGGTGGGTCTGCGACAAGATCATTTTCAGGCAACCTTCCCACAATACTGCAGCAACCTCGAGAGCGTACTACGAGTGACGAGCGTACTAGCACAGGAGGAAATTATTATGAGGAGAATGCTGAGGGAAGTGGTGACGCCAGCAGCGTCGGAGATCCAGAATCAGCGGCGGCTTTGGAAGCTGGCACGAGGCATGGCCCACGGGGGGGCAGCAAGTCGTCGTCCTCAAGCTCAAGGCAGGTTGTAGTAGAACGCAGGGAGCGTCGAGAAGGGAAATGGGAGAGGAAGCATTCTTGAGCAGGTTGTGGTCAAATTGGCTGCCAGTACGAACGAACTGTATCGATTATCTCCTGGTTGCCCTGTACTGTACTTTTCTCTATTATGTATGTGTACGCAGAGCGGCTAATGAAAATGGCTGTTTCGATCGGTGGCTTCGGTGTTCTTTTTCATCGCTCGTTTTCTTCACGGATATTTGTCGAACTGATAAACGGTGTCTTTTTTTATATAAAACAGAGTTTTTGCTTCATAAAATTATGTTAATCTATTTTTCAAGTTTTGTTTCTAATAATTAATCATGTATTAAATTGTTGCTTCTTTTTACATGCTAGGAGAGAAGGTTCCCACACGCTTAGACTGTAGTGGATGTCATACCTTCATCTTCTCCTTCTGCTTGCATGCATAAAGAGCTATATTGCTGGGAGGACCGTGTAAATCGTAAGTTGTCTAGTATTTCTTTCATCAAAAAATATGAGACATATTTTTCTTTTGTCATCGAGATTAAGGGGAATTAACTCAACTCCTATGAAACAAAACCATATAATTTTATACTGTATGTATATAACCAATAAGTATTAACATGATTTTGTTTTATCCCAATCAACAATTAATTTAGTATATGAAGATCACAAATATAAACTCTCTTAAAAAAAGTATATGTCTAACACTTAAGGGAATACCAATCTTGACAGAAACACTAGGCAGACCAAATCCAAATAGCCATGGCACGCTCATGCCTAGCCTAGTGAACGTGAACGAACTGGATGGTTGTCTACTCCGTGTTATTAACGGTGAAAAGGATAGGAAGAGGGGAAGTAGTCGTAGTCGTAGATGGTTCAGCGTGTGAAAATGGAGCGTCAATACGTCGCGTGGAAAGCATGAGCTGCTTGCTGCTGCAGAGTAGCTGCTGCTATTGCTTGCTGGGTCAAAGTCGTAACTCGGCAACCACTGCGCGTGCCAACAACCCCCACCACCACCACCACCATCTTCTCTTCTCTTC

>Os10t0580900-03 2000 bp upstream sequence

ATGGGCCTATTCAGATTGTAGCAAAAATAAACTTTACCAAATTTTGGCGATGCCAATACTTTGGCAATTTGGCAATATTGCCAAGTTTTGGTAGGATTTCTTATGTATTTACTAGAGCTTGGTAAAAAAAACTAAGTGGATGCACATATTTTGCAGCTTTAAAAAAAAATTGGTATGGTTTGAAATGGCATCAATCTGAACAACCCCTATATGTATATGTGTTCCTATGTTGTGGTGGGTGGGTTCACTTCATATTGATAGCGCATGTTTATTTGATGTTTCGCCACATCCTTTGTGGCTAGTAATTTAGGGTGAAGTATCGTAGGTTTTGACGATACTCAACATTTTGCATGAATTTGATATTTCTTGCCATGTCATATACTGATTGCTTTTGTAGAGAGAGCTTCAACGAGAATTGGATCAAGTTGAGTTGGGGCGGTCATCAAGGCGGGAGTTTTCAGCCAATACTAACAGGTGTGTTATGCTGGTAGGCGGTTGCCTCATTGTCTCACCATAAATAAATTTTCATACTGCTTCTGATGAAACTTCACTTGACCTGTTTGTTACCCTTGCCATGTGTGTGATGTGACGGGTGTTTTTGCAGCCGGTCTAGGGAGAGATACCGTGAAAGGGATGACGGTAGGGGACAGCAGGAAGGAAGGAGCCGAGGTGGAGGGGTTGAGGCCGGTGGGTCTGCGACAAGATCATTTTCAGGCAACCTTCCCACAATACTGCAGCAACCTCGAGAGCGTACTACGAGTGACGAGCGTACTAGCACAGGAGGAAATTATTATGAGGAGAATGCTGAGGGAAGTGGTGACGCCAGCAGCGTCGGAGATCCAGAATCAGCGGCGGCTTTGGAAGCTGGCACGAGGCATGGCCCACGGGGGGGCAGCAAGTCGTCGTCCTCAAGCTCAAGGCAGGTTGTAGTAGAACGCAGGGAGCGTCGAGAAGGGAAATGGGAGAGGAAGCATTCTTGAGCAGGTTGTGGTCAAATTGGCTGCCAGTACGAACGAACTGTATCGATTATCTCCTGGTTGCCCTGTACTGTACTTTTCTCTATTATGTATGTGTACGCAGAGCGGCTAATGAAAATGGCTGTTTCGATCGGTGGCTTCGGTGTTCTTTTTCATCGCTCGTTTTCTTCACGGATATTTGTCGAACTGATAAACGGTGTCTTTTTTTATATAAAACAGAGTTTTTGCTTCATAAAATTATGTTAATCTATTTTTCAAGTTTTGTTTCTAATAATTAATCATGTATTAAATTGTTGCTTCTTTTTACATGCTAGGAGAGAAGGTTCCCACACGCTTAGACTGTAGTGGATGTCATACCTTCATCTTCTCCTTCTGCTTGCATGCATAAAGAGCTATATTGCTGGGAGGACCGTGTAAATCGTAAGTTGTCTAGTATTTCTTTCATCAAAAAATATGAGACATATTTTTCTTTTGTCATCGAGATTAAGGGGAATTAACTCAACTCCTATGAAACAAAACCATATAATTTTATACTGTATGTATATAACCAATAAGTATTAACATGATTTTGTTTTATCCCAATCAACAATTAATTTAGTATATGAAGATCACAAATATAAACTCTCTTAAAAAAAGTATATGTCTAACACTTAAGGGAATACCAATCTTGACAGAAACACTAGGCAGACCAAATCCAAATAGCCATGGCACGCTCATGCCTAGCCTAGTGAACGTGAACGAACTGGATGGTTGTCTACTCCGTGTTATTAACGGTGAAAAGGATAGGAAGAGGGGAAGTAGTCGTAGTCGTAGATGGTTCAGCGTGTGAAAATGGAGCGTCAATACGTCGCGTGGAAAGCATGAGCTGCTTGCTGCTGCAGAGTAGCTGCTGCTATTGCTTGCTGGGTCAAAGTCGTAACTCGGCAACCACTGCGCGTGCCAACAACCCCCACCACCACCACCACCATCTTCTCTTCTCTTCTCTTCTCTTGTTTCTTCCCATCCCATCCTCTTCTCCTCCGC

>Os11t0135000-02 2000 bp upstream sequence

TTACAATAAGGTTGGAGAAGAAAAAAATGGTGACCCTAGCTATAGTAACACCAAAAAGTTTTGGCCATCAAAGTCATAGAAAAAACAACTTAAAAAAATAATCCCTCCAATCATAAATATTTGACGTTTATAACAAGATTTGGTTAAAATTTTAAAACATTAACGATCGATTTTATATTGGAATAAATTTATAAATATTTCAAATTTTATGATATTATGATAATTTTTTTCAATACGTTTTTACCGTACCAATTATTTTTTAAAAAATCTAAGCATTTGAAAAATTACTATGCCCAAAGTTTTAAAAGTTTGACTAAATCTTATCTTTAAAGGGTAAAAATTTGTTACCGGAGGAAGTACATGTTCTGCAATGCGTCGAAATGGAGATGGAATACGTATGATCCAGCTAATTCCATACAAATCAATCGTTCAGTCATATAATCGTCGCATGGATGAGAATAAGTTGCATTGACGTACCTTACTTCAAGACTTGAAGTGGCCACGCAGTGGACGAGGACGACGCTTGCACGGCAGAAGCTGGCAAATAATCAATCGTCAGCAACAGGTAGACCGTCGCGTCCACCTGCATGGCAGCCCGCTCAATCCTGTAGCAGCAACAAGAATTGACTTTGTGGCCTTTGTCCACGAACCACGATGGATAGATGCATGCATATCTTGGCCGCTGCAGCAGCAAAACACATCACACCGCATCAACAAGATCGTCTCTTGTGTTTGTGTTGTGTTCCCTCTACTGATGTGGCACGCGTACCAAGAGACAAAACCACCAGCAGCCATCCATTTATATGGCTTGACCCACTCGACTCCTCTCCCCTCTCGCCACCGTTGTTTGCCTTTGTCAATCTCCACCACATCATCTCCTACCTCGTCCTGCCTGCGGTGGCCTTCTCCTGCCTCTGCCTGCTTCACAAGTCCTTCTCTGACTCTGTCTTGGCCATCCTATTCCTAGCTCATCATCACACTCTTGCATACGTGCTTGTTGTCAACTCCATTGAGAGCGTCGCCGTTGATGGCTGAGCCGCCGGCGACCAAGGTATACCACGATGGCTGCCCCGGCTGCGCCATGGAGCAGAGGAAGGAGGAGCACAAGGGCATTCCCTACAAGGAGTTCCTCTTCGTTGCCATCACCACCCTCGCCTCCTGTATGAATATATAATATAGAATTAGCACGCATACATGCACAGTCAACTTAGTTTGATTTGATGTTTGTTTATCTCTTCTTGGTCTTATCTTATTTTACTTAGTACGTAGTAACATGCATCCTTCTCTGTCTCTCCCCTTGATTTTCAGCTCTGCCAATCTCTTCCTTGTTCCCCTTCTTGTACTTCATGGTAAGTTTAATCTCCATGCATTCCATTCAATTTCATGAAACATATATTTTGCAAAAGATACTAAATGCATTCCCTTCTTTACAACAGATAAGGGAATTGCATATTTCTCGGACAGAGGAAGATATTGGATTCTATGCTGGATTTCTTGGTATTAACATTTCTGAAAAATAATTAGTCACTGAGTAACAAAATTCACGAACACTTCTTTCACAACTACGTCAAAAATCCACTAATGTTTACATGATGAGAAATTTTATAATCCTTGAAAAAGTATCTCGATATACCTAAATTTTACACTCAAACACTGTAAAAGTTCTCTTTTTTCTTTTTGTTTTGGTACATCGAGGTACTTAGTACTAGAGGTACCAAATTTTACACTAGAAAATGTGGTATTTCCCGGTACTTTTTCAAGGATGATAAAATTACCCTTACATGATACTCCATATTTCTGCTGCAGGCGCATCATATATGATCGGTCGAGGTTTTGCATCAATCTTGTGGGGTATGGTAGCGGATCGTATTGGCCGAAAGCCTGTCATTATCTTTTCCATTTTTGCAGTGTAAGCACTATAGTACCTTTCCAAAAATATTGACAGGAATGTGTCCATGGCCTATTGGCATTTACTTTGTAGTCTGTTCCATCTGCTAAAC

>Os11t0207200-01 2000 bp upstream sequence

TATATGGTCATATGTTCTGATAAGGATATATTGACACAATATACTTTTTTAAAAAATATTGACATAATATGCACATATAGAGTGATAAGGATATATTTAGCTTCAGAGAAATCCATATCAAGTACTCCATTCGTTTCACAATGTAAGTCATTCTAACATTTATCACGTTCATATTGATACTAATGAATCTAGACATATAAATCTACATGGATTCATTAGCATCAATATGAATGTGGGAAATGTTAGAATGACTTATATGGTAAAACGGAGGAAGTACAAAACTAATAGAATCAGAGTAATGAGTGGTAGAATAAAAGATGACAAAGATGTGGAAAAAGAAATGCACATATCTTTTGATGTAATGGTAATAAAGTGAATATGTATATGTCGTACCATGTTGTTTTCGTCAACAATGCAAGCTCAAGCACTGCAAATTAAATATCATACTCCATAATAAACAATTAATCACATACATCAGCAGTAAACTTCCAGCATTGAAGCAACATATTATAAAAACAGTTTACCCACTCGATTTTTCCAACCTCTTTGGGTACATTCTTTTCTCCCCCTTGTTGACAAAGTCTCTAACCCTCATCTAGTAAACCTGAAATTGACAGACATTAATTTCTACTATCAAATAAATACGCACTCAAGCTCCAGCTCTCCTGAAGCTGCCATCCAATCAAACAATTTTAACTCTATATAAAATGAGAGTGAAACTAGGTGAAACGCTATCACAAAATAAACTAAAAAGATAGAGCTAGATTTAGACTGCTCACCTACTTCATAACTCTACTCTAAAACTCAACTTATCAAGTTAAATTTAGAAACTGGACCTCCATCAATCACCATTAGCGCACTCCCATTAATTAATACGAAAAGCCACGTGTCACCATCACCAAAAATTTAAGGCCGTTTTGGATTGAAGCCAAAACGCGCCCTACCAAAATTTTGGTAACTTCAAATAGTAAACATGCTAGTTTCGATTGAAGCCAAACAATCAGTAGTGTCTATACTTAATTTATTTGGTCACATTCTAGAAACTTCTTCACTTTACTACTCAACTTTGGCTTTATATTGGTATCAAACCAAACATGGGCTAGCCTACTTTACCCTACCAAAAAATATTGCTAGTGCCAAAATTTACCTACATTCTGATACTACCAATGAATTGGTATAGGCTCTTACACCCACCACCCGTTAACCACTACCCTACCCGACACGCCGCCGGTGCAATACCCCCACGTCAGCGCACGTCCCGGCCCCACCTGTCAGCCACCATCGGAGCATGGGCCCCACCACCCCCACCACCCCGGGTAAGGTTGCCAGCCCGGACAGCGGGATAGCTACGTGTAGTGTACCGCGTGACGTATCACACTCGCAGTATATATTTTTGCTACGTGTACCCGAGAAGGGCGGGAGGGTGGGGGCCCCACTGACAGGTGGGCCCCGCAACGGCGACCTTGGTCGTTACTACTACAAAGAAGTCGTTTCCCTCTCCTTTTCTCTCTCTCTCTCATCATCATCGTCTTCTTCGTTGTGGGCGTAGCCAATTTCGTTCGCCATTTTCTCCTTCCATTTTCTCGGCGAGGAGGAGGGGGAAGAGCTAGGATGCCGGCGTGGTGGAAGAGGGGGAAGGGGAAGAAGGGGAAAGGGGGATCGGCGGCGGCGGGGGAGGAGGAGGAGGGGAGGAGGAAGGCGGGGAGCTTCGACGAGGCGCTCCTAGCCGGGAAGCAGAGTCGGGAGGAGAAGGAGGTGGTGGTGGTCGGGCACCCGCTGCCGCGGCCGGCGTCGCTGCCGGCGCCGTCGGCGGCGTCCTCGTCCGCGGCCGGGTCCGCCTCGGCGTCCAGCGCCGGCAGCTCCTCGCTCGGCTCGTCGGCGGCGTCCGACGAGCCGCCGGATCTCGGGACTTACAGGTGAGGAGGAGGAGGAGTGGTTTGGTTTGGTTTGGTCCGGTGGTTTGCAATTCGTTCGTATTTGGTAGGTGGGTGGGTGGGTGG

>Os11t0292050-00 2000 bp upstream sequence

AGCTCTACGACCTAACCGGCGGCTGCGCGGAGCGAAGGCTGCACGGGGATGGAGAACCTCCCGGGCCACGCCGCAGCAAGGGCCTCGGTGAAAATCTGCGCGGCGAGGAGGAACGGGTGCGGGGATGTGGCGACGTCACCCGCCGTGTCGCCCGAGCGGGAGCTGTGAGGACGTAGAGCGCAGGGAGCACAGCGTCGGCGAGGAAGCCGAGCACAGGGGTTGAGAAGGCGATGTCGGTGAGCGGCACGGTGGTGGAGAAGGCGAGCACGACCGTGGCGCCGAACCAGTTGAGCTGTTTGATGGAGAAGAGGTAGTAGTGGTGGTGTGGCCACACCATTGGCGACGTCGGTGTTGGGGTGGTCGCTGGCAGTGACCGGAGGATACGGCCGGAGTGTGAGAGGGAGTTAGTTATGGGGGAAGATTATGACAAGTGGACCCAAGGGTATTTTTGATATTTTACGCTGTTTATCTTTCCTATTGAACGAGAAATCAATATTTTAATGTCTGCGGTATCCATTAGCAAATAACTATATTGAGTGTCTTTGCGCGATGTCTCCCAGCTAATACCACATAGTCGCGAAATTGCCTTTTTTTTTTCTTTCATATTTGTATCGTTTTGGTAGGGGATGGGATGTGCTATACGGGGGAATGGACTGAGTGCTTGGCGAAATTACTAAAATATCCTCGCTGTTTTTTGACGTATAGTTGTCCTCTGTCGTGAGTTATCCATCAGTTTGCCCTCCGCCGCCGCCGCCTCCGCCGCCGCCGATCCCCATCCATCCCGCGAGGCGCCGGCGAGCAGGGCGCCATCGTTCGTCATGCTGCTTCCGCCGCATCCTGGTGAGCACATCCTCCGCCACCGCCGCCTGCGCCGATCCCCATCCATCCATTGCGCGAGCGGGCGGGCGAGCAGGGTCGCCGTCGTTCGTCCTGCTGCTCCCGCTGCATCCGTTACGCACGTCCTCTTTCCCCCATCTCTGACTCTCCACCCACGCACTTTCTCTCGAATTCGCGTTAGCCCTAGCTTCTGCTGTAGAAATTTGCCTGAACCTTAATACTAATAGCAGTTTGTTTGTTCCCAAAAGGTTTAGGGGTTCAACTGAACCCCCATGTCCCATTTTAGATCCGCCACTGCCTATTCTATCTATATCGTGTGCACTTGAGGTACAGTCATGTGTATGGTCTAGTGTTGTCGTAATTAAAACCCAATGATGTGCCCTTTGGATGGGTTGAGCACAGCAGTTTACAGTAGTGTTATTTCGTATTTTGGAATTAGTGTTTCTTTATACTTTGGAGACTATCCTGTCTTATATATAAACTAACTGGAACATTTCCAATGAAACAGTTTTCACCGATAGCATCATATGTGACGCGTAACTGAAACAAGCATCGAAATACTATTTTCGTTCATTGGTATGTTCTCATTGTTAGGGTGATTTTCAACTCTGAGAAAGAAAGCACATCGGTGAGCCAGAACCTTCTTGACAAACCTGAATCAGAGACATGCGACGCCGGATGGATGATGTGAGTTCGTTATTTCTAAACCCCATTTCTTCCATCTTCTATGTTTCATCTATGGGGGCATATATTATACCAAAGTGAAGTATGCCCAGTTAATTTTCTTATTTACTTGTTATGATTGAGTTATCGTGTGCTGATCCTCTTGAAATATATTGTACTTGGGTGTAAATACAAAGTAATGATATATATGATGATACGACTGATATTCTATACCATATCGTCCAGCCATAACATATATGATATATGGACAAAATTTAAAGCCCTGTGATGGTTTCAATTTTCAGCTAGTGTCAAACGCACAAGAGAACAATGTCACCTATGTGGATAGTAGTTCTGAAGAAAGTGAAGATGTGAGTTTGGAAATTTAATTTAGTGCTGTATCACTTACTTACTGCACCTCAACAAGAGGCATTTAAATTGTATGAAGGATTTTTTTTGTTGTGGTTTTTTATTAAAATTTGATGTTGTAATGTGTGTAG

>Os11t0592200-01 2000 bp upstream sequence

TCTCTCCAGGTCGCCAGGGGCAAGGGCGGCGCCAGGCTGACACGAGAGGGGGACGTGAATCGCCACCACCTCCCACCTCCGGTCGTCGCCGGCGGCCCCACCCTCTAGCTGGCAACTTTCACAGCCGCCACGGGAGGGTTAACCAAGCAAGCTGTGACCGTCGCCGCATCCTACCCAGGCTGCGTCGAGCTCCCACCTCTAGTCGTCGCCGGCGGCCCCACCCTCTAGCTGGCAACTTTCACAGCCGCCACGGGAGGGTCAACCAAGCAAGCTGCGATCGTCGCCGCATCCTACCCAGGCTGCGCCGAGCTCCCACCTCCGATTGTCGACGTCCTCTAGCTGTCGGTTGCCTCCTTCATCCGCTTGCTTGGCCGCCTCCCACCTCCGGTCACCGCCGCCTCCTACCTCCACTGCGCTGAGCTCCCCCTTCGGCCGTTGAACTGTGACGGGTAAACGACATGCCCACGGGCACATGGCACCCGCGGGCGACGGTCATGGACAGTATTTCTTTCCTGTCATGTAGCGGGTATACCCGCGGACATGAATTACCTCGTGGGCAAGGGCATGAGTAGTGCCCGTCGTGCCCAGCTGCCATGACATATTTGGATTTTTTTTTAGATAATGGAATATAACATGCCATATTTGGATGGGCCTCAATTCCCACCCATGCTTGTTTACAATTTACAAACCAACATCTTAGCTCCAAATAAAATTGACACTAATAACACTATCTTCGTAACAGAGAGTTTAGTTTCACGGGCGGAGAATAATAATAATACTCTGTCAACTGTAAAGAATTTATATAGGAGTACTCTGGAGGTAAAAGGGGCAAAAAAAAAAGAAAAATAATCATGCTTGTCCGAGCAACAACTATGGTACACTATTTCGGAATGCATAAATATACCATGGGTGACTGAAATTCTAAAATTTTCAATTGAAGAGCTTCAAAGATTTCAACGAATTCCGCTAAGTTTGCAAAATCAAATAGAATTTGAAAGTTTCGGCTGGTAAAATATCCTACCGGGACCCTCCAATAAACCCTGAAATTTTGCGAGTTTTGGGAATTTAGTTCCTGTCAACAGCTGCCAGTGCCACACCCTTCCCTTACCTCTGAAACATAGGAAATTTTGCAGGATTTCCATGAGAATTTTTAGTGGCCACCACATATAAAGCGAAGCAAAAGTCACATGGAGCCTGATTTTTTCCCCCATAATCCGTCAAAATTCCAAGCACTTTGTACTTTTTTGCTGCTGGAATTCCAGGGCACAGCACATCGATCTGGCCCTTGGGCGATTGACAATTTTGCGCCACATAGCAGCAGCAGATAAGGTTTTGGAACGGCTGCTACACGTACGAACCAAAAGGATGACCAAGAAGGAGAATTTGAAGATTTTTTTATAGTATTAATTATGATTGTTGATCGAATTCGTCAATGGCAAAATCGGCCTGGCTCTGGCTGGCTGGCTGCTAATTCATTCCAGAAGGTAGCCAGGAATTTGGAGGTAGGACTAGCATTTACATGAATTTTCTTGAACTGAAGAACAATCATATCTTGAGGTCAATATTGAAGCACAAGTTAATTAGGACTTCAATTTACCAACTTATGTGCGTTCTAGCTCTATTTACCAACTCCTTGCCTGTAAAGGTTTGGAGATTATTTGGAATAATTAATAGGAGTATTGCTTGTGTTGATGTGTGTAATTAACAGATACTTGTAATATTAATTCTGGTGTTTGGAACATGCGCTTATTGCTAATGTGCTACTTACATTCTTTTTTTTTTCTTACTACCCAGTGCTAAAAGAGTATTACACGTTAGGTGTGTACCAGTGCTTCCCAATTACTAATATACAGGGGAAGAGAAGAAGAAATGAAGAGTATATATGCTTCTCACTTGCAACCATGGAATGGAATTAAGACACAAATTCTTCATGCCGGAAAATTCCACAATGCACGGCTCTCACACCCCTATATATATGGTCCCAAAGTTTGGTGCATACT

>Os12t0125800-00 2000 bp upstream sequence

TCTTCTGATATTGTTTTAATTTTTTATGGTAATATTGTTTTAAATTTATTTTAACTTTATAATTAATTCATTCATCCATATCCTTATATAAATATTAAAAAATCATGAATTTGCTGTTTAGTGTATATCCAAGTACTGCTTAGTACTAGTATTTGTTACTCCCTCCATCCCCTAATATAAGGAATTTTGACATTTTACCTACACTGTTTGACCGCTCGTCTTATTAAAAAATTTGTACAAATATAAAAAACAAAAAGTTGTGCTTAAAGTACTTTGGATAATAAAGTAAGTCACAAATAAAATAAATAATAATTTTAAAATTTTTAAATAAGACGAATAGTCAAACAGTGCAAGTAAAATGTTAAAATCTCTCGAGAATGGAGATGTTAAAATCTCTATATTCTCGAGAATGGAGGGAGTAGCTTCACGAGAAAAGAACGTGACCAACCTAAATAGACGGCACGTGACCAACCTACCAAAGGATATAAGTATGGTTAGCTGCAGGCGTGCTAGACTGGTGTTACTGCTAGTGTGGCTCGACGAGAAGAGTTGAATGACCGAGACGGGAGCTTATTTGGCAGACAGTGACCGACCACGACGCTCTGCTGTGTTCGAACCACACGGTTCGGAACACTTTGTCCCTATAAAAAAAACAGAGTAACCCTTATCACAAGTGATGATTAAAATGACTTTAAAAAGTAAATTAATATAATTTTTTAAACAATGTCTTTATTAAAAAAAATTAAAATACACACCGTTTTGCCAGGTTTAAAAAACCTTTGTATAAAATACAATCTCTGTCACAAGATAAACCACCCTCATCCAATACAAGTTGGTTTATTTTTCTGTTATAAAATAAACCATTCTCGTTTGGTACGTGTTGGCTTATTTTGTGAAGAAAGGAATAAATGTTTAATTTCGGCGGACATTGACCGACCACGACGCATGCATCACGCATGCAGTGGCCGATTGCAGGCCGGACAAACGTCCATTATATTTTATTGACGAATTAAGCTGACGGCGGCGGGCCAGCTACTATTCCGGCCGCCTACGTCCGCCGTGCGTGGCCTCCTGTGCTTCACGATGCAGCCTGCTGGTGGTGCCCTGTAGGTACGGGTACCTAGCATCTCTATGTGGCCTCTTCCGACGCCCCGCATTCGCAAATAAGGGCCTGTTTGTTACAGCTACAACTCCTAAATATAAATATAACTCTAGGAGTTGAGTCTGGAGTGGATTTATGGAGTTGCCTAAACCTATCTCCACAACTCTAATATATTTTGTGAGAGAGCTCCACCCAACTCCACTCTCAATTTTAGTGGAGCTGAAACTGTTTGGCTGAGCTCCAGCTCCAGAAGAGATGGAGTTGGAGCTGGAGCTGTACCAAACAGGCCCTAAGCCGTCGTACCGCGTACGCGTACGTATAACCTCGCGTCTTTACAGTCTACAACTACAGGCAGCAAAGCTATATACAGTACACGTTCTTGCCCTCATTGCCACATAGTACAATGTACACAAAACCCCAGATCGAATCTCACCCTTCCTTCTTACTCCACTTACTATTGCTAATTAATTCTCAACATGTATATGGATGATCCTGAATATTCTCAACTGGATATATACATTGATCTGATCAACTAGACTAGAGTCACTATTAATTAATAATCATCGATTAATTAAGTCCTGTTAATTCCCGATTCTGCCCTCCGCCATACCAAGTTACCAACCCACGGCTTCTCCTGTACTTCCATTCGACTCCCTTTATATATATCATCAGTAGCTCCATACTTGAGCATCCTTGATGCTTCCCTTTCCTCCAGCAGCGCCATTGCTACTGAATCTGAAGCACCACCACTCCATCTCCATTGCTGCAAACTACAAGCCGCCACTAATTAATTAGCAAAATGGAGAAGCCGGGTGATGATGAGAAGCTGACGGTTCCTCTGTTGGAGCCTAAGCCGGCTACCTACAAGCATCAGGAAGATGATGATGCAGAGGAGGAT

>Os12t0131400-01 2000 bp upstream sequence

AAAGTGGCTGAGGTTGCGCAGAGCTATGTGAGGGTCTTGACGACATGTTCCAACCTTTTCAGGCTTTCATCAGCAATGACCAGATTCTTGAGAGATCGGGATGACAGTATGAGGTCACTGGCCTTGCGCTTGCGGGCGATGTTTGAGCCGTCGACCTCGAATTCGTCGAGGACATCTTTGGCTTCCTGAGCCGCCGCATGTAGGCGCATCAGCCAGTGGAGAAGGTTGGTGTTGGTGATGAGCCGGCTGTCTGCCGCCTCGGATATGGCCTTCACCATGGTGAGCTTGGCCTTTAGTTCAGCAAGCAACTCCTTGGCATTGCTCTGGAAAATTGTAGTTGCTCTCGAGGCATCATTGCCACCGCCATCTTTCTCTCTCTACAAATCTATACCTAATTAAAATATACGAAAGATTTTCATAGCTTTTTTTCGTCTGTCATTGACTCTGAGTCAGCCCTGCTTCCTTTTTCTCTCCCTTTCAGTCTTCCGGACCTCCATTTCTCTCCCACCTATTTCACCTTTTTCTTTTAGGAGGATTTTGTATTTTCTTTAATAAGTCCGATCCACTGCAAGCTACGTGCGCCTCTAGCTGCCGGCCAAATCCTAGCACCGTTGCCACGTCGATTTGCCTCATACGTTTCCTCCTCCAATTGATTGCAAAAATCGATTCCCCACTCCCCCCTCATTATTTCCCCATGATTTTCCCTATTAATTCAGCTTTAATTCATCAAATTTAATCTGCGTTTAACGGTCACCAATGATGTAGCCGACAAGCTTCCCCGGGTCATTTTTATCCCCAATTTTGCAGATTCGCTTCAATTATTGAGAAACAGAAAGAACTCCCTCATTATTTCTCCAATGATTTTCCTTATAAATCTGGCTTTAATTCACCAGATTTAATCTTCGTTTAACGGTCACCAATAATGCGGCCGACAAGCTTCCCCAGGTCATTTTTATCCCCCAGTTTTATGGATTCGCTTCGATTACGGGGAAATAGAAACAGAAGATTGAGTGGATTAAAAATCCACAACTAAATTTGGGAGATCGAGAAAGATTCGGCCAGATTTGATGCAAAGGATAGGCTACAGCATCATAGTAGCACATGACTGTCGTCCGGAGGTTGAAGACGCTAATGAAACGTGTGGGTCTAGCTTGGTAGCAAGAGGAGACATCGGCTATGCTAGCTGGGTCTGCAGCTCCACTTATGTATAGTGATCTTAATGTTTCAATATATTGCTTAGCTCCTAATAGTTGTTGTATATTAAAAAACACTTACCTGTTGCCAGGGGCGGACTGGTGTCAGACGACACCAACGACTTTCGGCAAAACCTATAGCATAACTGCTTATCTATGTTATAACAATATGATCTAAACATAATTAGCATACTATGACATCAATAGAAACGTTATGACACCAACGAATCGATTTTATGTATCCGCCATTGCCTGTTGCAACGCAGTCTTTTTCTAGTATATGAAATATAGCACAGGCGAGTGGCCTTGACGATGGCGGCCGGGGCTTGTGTCGATCTGTTGGAGGAATTGTCTGGTTGGGGAAGAACCACCTGTATCACTGGCCTAAATTCTGTATTCTACTACTTACCTATTGTCTGATGAAACTGTTGGCTGAATGGCTCCGGACCCACGAGTCCATCCACCTGCCACGAGAGGGCTAGATGGTTGCAGCTCTTTTCCTCCTCGTCAACGCGTCGAGCACAAGCGCGTGGCAGCCGTGGCCTGATGGCGCGCCGTGGAATTTGGGTAGGAGTGGATATACAATTGTATATGTTGATGATGTAACGTACGGTTGATTAATAATAAATTAGGTGGGAAAAGATCAGTTGTTACTCATAACAACGAAATTTCCTTACTGATAAGACATGCGTTCACATACTAGATTGTACAAGTTCTATCGTGTACAGACATGCCTGATGCCTACCTATAGTCCAATATACAGTATACTAGATTGTGATATCTCCAACGACAGTTGTTGAAGCCGCT

>Os12t0273980-00 2000 bp upstream sequence

GTCAGAGATTAGTGGTCAGTGATGACTCTCTTCACCTTTGACAGGCGAAGGACCGTCAGTGGTGACCAGCCCCTATGACGGGCCACGTGTAGAACCCGTCACAGGTAACTATTATCTCAAATGGCCCATTAGTTTGTGAGCCGTCAGTGATGTCTATAATCCAGAAAAAAAAGAAAAATAGATTGCAACCCGTCACACGTGTGAAAAAACCCTAGCAAGAAAATTGGCACACGCTGCCATGGTGGTGCTTGCTGTCATTAAACTGACCACATCACAAAAACTTCAAACTGACCAATAGTTCATTGTCCTCCATCACCGCATCACCATCTTATATAGCAATCATTTACAAAAGGGGATGGCAAAAATTTATCATACAACAATATCATATCATTCACAAAAGGGGCACAATGGCCGATAGTTCGTCGTCCTCCATGACGAACCAGCTACGAGAGACACAGGTGGAGACGCCGCGCTCGCAGCCGCCGGGGAGGCGGCGGAGGATCTCGAAGAGGCACTCCACTGCTGTTGCACTACCAAACAAAAATCAAGAAACAAGAAACCACCATTAGAGCCCACATTCTCAAACCAAACACCTCAAATCATCATCATCATCAACAACAACAACAACAACAACAACAACAACAACAACAACAAGGAGAAGATTTAAGTAAAAAAAAACAACAAGAAGAAGAAACCTCACCGAGGTGGCCGGTGGGTGGGCATGGAGGAGGCGGTACCTGGCGTTCAGATCTGCGCATCCCCGGCCTCGTGGAGGTCGGATCTGGCGGCTAGCGCGGTCGGTGGCGGAGCTAAGGGAGGCGTTGGTGGCATTGGTGGCCGGGAGGCGGGGGAGGCGCTGGCGGAGGTGGAGGCGTAGGTGGCGGCCGGCTAGCGCGGGAGGAGGCGAAGGCGCGGCTGGAGGCGTCGCCGGCAGCGGCGGCGGAGGTGGAGACCGCTGCCGCCGTCAGGCCTCGGCCGCCTCCCCTCCCGCCGTCGGCCGCCACCGCGCCACGGCTCCAGATCCGTCGTCCGCCACCCCGCTTCCCCTCGCCCCTGCCGGATCGGGCGGCCGAGCCGCCGCCGCGCCTCCCCTCCCGCCCCTGTCGGATCTGGCGGTCGGGCCGGCGCCGCCGCGCCTCCCCTCCCGCCCTTGCACAAAGAAAGAGAGGGGAGATAGAGAAGAAGTGGAAGAGATAGAGGAGAAGTGGAGAAGAGGGTGCCGGCCGACCTTATCTCCTCCTATGGGCCCCGCTGGGAGAGGAGATATATATATATATATATATAGTGGCAGAAGTCATCTCAAACGGGCTAAGACTTAAAGCCGTTTGAGGTGAGTAAACCTTACCTCTGACGGGCCGTCCGGCTGCGATCCGTCACAGGTAAACCCGTCACAAGTGACTAGTTACCTGTGACGGGTCGCAGCCGGATGACTCACCGATGACGGCATCCCGTATGACCCGTCAAAGGTGACGGTCATCAGTGACCATTTCGTTTGCCTATCACAGTTATCCCGTCAGAGGTGTGAGGATCTGGTGTAGTGATAGTTTACACCGTTAACTTTTTGTTACACGTTTTACCATTCGTCTTATAAAAAAATTTACGTAATTATAATTTATTTTGTTGTGATGCCATCTATTAGGAAATGGAGGGAGTATAACGCATATCTATCGCACCATGGCCCCCATCCTAGTCGGAAACTAGGAAGAAATTAAAATTTACTCCTCCTACGACTCCTATTCGGAAACTGGTAATTTGGGCATCGAATCAAACCAGAGCGGATAGATCGCAGAGGGTTTGCTCCTCGTAACGGATTGCATTGCTCGATCCCTCCCCGGCCCCTACCAAATTCACCTCCCTGGCCGATAGCGCCGCACCGCAGTTATCCACCTCCTCCGGCCGGCCGCCGGCCAGTCGACCAGTCGAGTCGGAGGCGGAGTGCATCGATAGATCGATCGGTTTCCATCGGAAATAATCGGAGAAGGTTTCGTTTCCTTCATTAA

>Os12t0568700-00 2000 bp upstream sequence

CGAGCTAGCCTGACCTAGCTCCTCGTCGTCAAAGCACGAAGAAATTTGCAGATCACTGCCGTTTCACACTCCGATCATGTGGGCGGCTTACACGCAGGAATTAGACATGCATGCAGCATCAACACCAGATAAGTAATTAGCTGGAAGCATGGTCACTAGTCACACGCTTCACGCGCGCGGCTAGCTTATTAGCAAGTGAAACGTGTCTCCGTGGCCACCACACACACACACTTGTTCACTTTGACTTCCTAACTGGTTAAGCAGATCTGCCAATACTTCTATCAGCGAAAATTGGTCGGTTTTCCTGAAAAATGAGCTTTCCAGGTCGGTTCAGGAATACAAACCGCTCGATTTTTCAGTCAAGTCTAAATTAAACCATGTTTATTTTGTAAAGAAAGTAGCCAATAGTTCACATGTAAACCGAACAAACCATCTATCTGTTTTTGTTTTTTTAGTAGTTGGGTTTTGTGAATCCTCTTGCAACTTACAAACCTAATTTCGCCGATATTAAAATTGAACTCCATGTTACAATCATGTTAAAGATTGAAGAAAAATAGTTTTCCTATTTAGAATTTATTAATTTTGGGTGATGCATATTAAAGACCTACAAAGTCGAATGATTCCATGGCTCTCTCTCTTTACCAACCAATCACCATTATATAGTGGTAGAGCTAGAAATATCGTGGAACCCAAATATTTGGGAGATTCTATTGGTTGTATTTTTTTTAAGTTAAGCCTACGGAGCTATCCAAAACCTCACCTCTGGTTGTGGCTTCGCCGCTACCCTCGTCCTGATCCCCATCTCCTTATCCCGTCATGGTCAAGTTCATCTAGTTAGCATCAAACACTTCACCGAGCTAGAGAGATAGATGAGTTGACATCTAAGACTGTCACAAAATTTTCGGTTGCATGTACAAATCACCAAGTTCAAAGTCAAAAAAGCAAAAGGTCAAAAGCCAAGTTCATTTGAATTTCCACCTGGCACGCCGGTTGGGCTAGCTGGCCTTGGTAGCCACCAACCCAAGCATGCACAAACACTCCTTTAATTTGCTTTTTATCTGTCTCAAATCACGAGAATCCTCTCTCACACAATTTCACACGCTATCAATTAATCATACTATCAAGATGAAACTAGCTAATTAAGCTGTTGTGTCAACGTCGCCTCATATACCTAACAAATAAAGCCTTAATTGATTAACCGTGTAACATCTTCCTCACGCACCGATTGTCCTACACCACTCAGCCGTGGTACAGATGGCACTGATTTTTGGATGACATGGTTTTTGCTGCAAATATCATTTGGATTCGTCGAATGATAATCATGGCGATAGATTAAGACGCGATGAACCAACCAAACACAGACACACATACAAACAGCAATAATTTACCATCTCCTTCCTAAAAATGTATAACGCCGTTGATTTTTCACACAACGTTTAACAATTCGTTTTTATTTAAAATTTAGTATAAAATATGTAAAAATATAAGTTAAATTATATTTTATTTGATGATAAACGATAAAATAAACAAAATTTATACATATTTTTTTTAAAAAATATAAATGATCAGATATTATATGAAAAATAAACAGAGTTATATATTTCAAAATAGAGGGAATATATATAATATAGATTGATTTTGATGTAATTGTTTTCTGACTATATATCGTCACTTACATGTGGGCCCTCCTCGGAGGCAGAAAGAATCTGTTGACTGGTTACATCATCAGTAAGTGGTAGGAAACTATAGTACGAGTGTCACAAGCGCAAAATAGTCTACAGCTCCACATTAATGACACAATTAACACCCTCACATGTCCGTGCCTAACAGCCATCACACACGTGGCACCCTCTCTCTTTTTTGCCCTTCCCATTCTGAGGCTGCCATGTGGGCCCCACCTGCGTCGCCACGTCAGCGTGCTCGTGAACCCGAGGGCCGAGGCGGCGACCTATATAATTTCAGGTGGTGGAGAAGGTGTGCGAGAGTGTGGGTCGGTCGCG

>Os12t0594950-01 2000 bp upstream sequence

GGCGGCGAGGGCGCGCGCGGCGAGGAGTTGGCGGCCGCCGCGGGCGCGCCGTGCGCGAGGTGGCGGCGGCCGAGGGCGTGCTGGCGGCGACGGCTGCCGCGTCGAGGGCCAGCCGACGCCCGCTCCCCGAAGGTGTCGGCGGTCGCAGCTCTTCCACGCGGAGGCGGCTGCTCCCAGCGACGAGCTATCCGGCTGGAGGGCGAGACGAGGTAGGCGGCTGGAGGCGGGCAGAAAAGAGGAATTTGTTGGGAATGGAGGAAATCCTATCAATGTGACTTGTGCTAGTTTCGTCAAAATTCTATCGAAATGCTATTGAAATGCTGTCGAAATTTTATTCAAAATTTGGTCAGATTTTGTAAGTGTATAGTATTTGTGATTTTTTTGGTGAAAATACAAAAACATCACAACATATGCTCATATTTGAATTACTTACAAGCTGAATTTACAAAATAAGGGCAAAATCACACAAAGTTGAAAAAAATGGGGGCAAAAATACAATAACCACCTAGGGTGTAAATGTCCTTTTCTCTGCAACTAACACCGTTAAAACCTGCTCTTAAAAGTAGGGGTACACTGCTCATTCAGATTAAAAAGTAGAGGTAAAACTGCAAATCCTAGAAAGTAGTAGGAGTAAAACAGCAATTGGCTTCGAAAGTAGGGGCACTGATGCAATTGCCCCTTTAAAAAAATATTGAGAACTTGACACGTCATGACATCGATCTGTATTCATATTGCTAAATTTCCTGACAGCACACAGGTTTGTTTAGGCATTCACTTTCTCATAGTTTGTTGATTAATGGTGACAGTATTTGGCCGGTTGGCCCCTCCTGATTAAGCAGCAGCACAGAAATGAGATAACTTCACCGTTGAATGTCCTGTTTTTCTCAGAGATGTTCCTTTCATTTTTGTTCAGAGTAATCCTAAATTGAAGGTAATATGGGCAGTGTGTAAACTTTCAAAAATTTCATCATTTAGACACATATATAGAGTATTAAATATGGACGAAAAAAAACCAATTACACAGTATCAAATGTGTCCTTATACGTTAAAATTTTTTTACCAAAACAACTAAACACGGTCACAGTCAGTCGGTCGGTCGGTCATCAGAGATCACTGCTGTCAGTCAGTATATATACAGATTCATTGGAATCTAATTATCAGTATTTTGAAGGTTTATCCACTGCAGAAGTACAGAGCACAAGAGTCAAAACTCAAAAGACCAGGAGAGGCATTTACCTAGGTAACTCTGCAAACTAATTCTACTACATGTTTGGACGGTGGGTTACTGTAATACTGTTAACCACACCTAGTTTACTATTTGCACATAGTTTAATGAAACCGTACTACTGTGCATTCGACTAATTAAGTTAGAAGCATTAATGTTGCTAGGAGAAAAGTGTCCTCGATGGAAAAGTGATTCTCTGTTGCTAGCGTGTGACAGGTTTATGACAAAAGAATCGATTCTCTTCATGTGATCTTCACGTGTCCGGCCAATTCAGTGTTATTTTCTTCCTCAGTCAATCACTGGCCGGTTACTTGTACCCATTGCTTTGTTCAGTTGTCACTCACTCCTACTAATAACTTACTAATAACAGCCCTAAAAAATGACTTAGCAGCACTAGCTGTTAGCCTTCTTTAATCTGAATCTCTAGGAAACTGAAACGGAAGACGGGATTTCAGGCTGAAGACGTCAAAAAGTGATGCTGAACTTTATTCACATACCTACTCGTGGCATTTACCTAATTACCTTTGGGGTAAAGGGATCACTCGAATGTTCTGGACAATTCGTCTAATCACCATCAATGGACAAAAACAACCGAAGAAAAAACAATTCATCAGCTTAGCCCTGACACGTGAAACCATGTCCTACAGCTACCTGTTTTGATCTTCTTGGTTTAAGTACATGTCGTTTTCATTAGGTGATTAGTTGATTAATTAATCTTGAGCTCCTCCTAAAACCATGTATAAATACAGCTCACTTGCTAATCACTCTCACTCTC

>Os12t0609100-01 2000 bp upstream sequence

TGGGAACCGCTCGTCGACGCTGCCGCGGAGGGTGCTCGGGTTCGCCTGGCTGAACTGCACGGCGTGGCGCGGCGACGGCGATGGGAAATGGTGCGGCGCCGCGACGCCGTAGGAGTCCACGCCCTGCTCACGGTGGCCGTGCTGCTCGGCGGCTGCGTACTGCTGCCGGCCGTCGAATCCAAACTGCCGCTGCAGACATCTCAGACCATTAGCATCTAGCATCTCGACGGGAAGAACACGATGGCAGGGACGGGGAGGTCGGAAGCGAGGCGAACCTGCTTCATGGTGGAGGCGGCGGCGGCGCCGTCGAAGGCGTCACCGGGCGAGACAGGCGCGGCGGTGGGCGGAACCCGAAGAGGGAGAAGCGGTCGAACACCGCCGCCTCCTTGGAATCCTCCTCCCTCGCCGCCGCCAACGACGACGATGACCTGAAGGACATGAACGCTTACCTTGGACGCCGGAAACTGCCACTGCATCGCTGCCGACGGGCCTCCTCCTCCTCCGAGGTAATCTGCATCACGAGAAACTACGAATTCACTCACAAATCACGACGCACAGAAAAAAAAAACAATCTGAAATCGATATCATCCCAAATCCATAGCTGCACGCATCCATCTCTGCTCTGGCAGATAACATGCCCAAAATCCGAGCTCGCACACACCCCCGCATGAGAGAGAGAGAGATTAAAAACACCGGTAGGATCTCGCCCGAGTCGAGCATCACGGCCACGGCGGACACCGCCGCCGCCGACAGCGGCGACCCGACGGAGTCAACGAGCGCGTCCAGCGCCGTGGCGCGGGAATCCGCCGACCAAGGCAGAGTCGCGGGATCAGCACATCCGCCCCCGCCACAACCCCTTCACCCCCACCGTTCCGGGGCGATACGAGCAGCCGCGCGTCGGCGGCGAGCTAGGACAGCTCGGCAGCCGAGGACGACAACAGCGACACGGTGTGGAGATTCCCGGCGATGGACATGAGCGCGGAGGCGAGGGGGCGGAGGAGGAGCAGCGCGGAGTGGTGGGAGAGCGACTGCGAGAGCAAGGTGAGCGACGCCACGCCAGGTGAGGAAGGAGAGAAAAGATGGGGAGAAAAAAAAAAGAGAAAAAGAAAATTTAGCTATATTATCGTCCACGTAGCCAAAACCACGGTAAAACGAGACTTTGGACCGGAATGATACATTAAACCAAGTTTAAGGATCTTGATACACGTTTTACAAGTTTATGAACTTAAGTGAAACCTCGTTATAAGTTTAAAGACCGCTAGTGTAAATTACTCTATAATAAATGGCCAGCTGTGAAGGATGTTGACTAGTTAGTACTCCGGCCTGATATGGTTAGAGCAATGACTTGGACTAGTGAGTCTTCCATGTGCCGAGTACTACAACAAGTTTGGTGTTATAGAAATCGCTAAAAAATTTTCTATAAATCTAGTTTAATTTTTTTTTCTATAAATCTAGTTAAACTTAAAAGAAGTTTGACTTAAAAAATTCAAAACTGACTTATAATATGAAACACAGGGGTAGTGTATTATCAATTAATACATCAGTTTTGATAACCATTCAGATTTTCAATCGCTCGAATTATTTAATATATCATTGAAAAAAATAAATCTGTTCAATGCAAAAAGGGACAAGACTGAGACATTGCTAACGACTACGTAGTCTTTTCCACAGCCGCCTACCTTGCTCCAATATATAAGAACTTGAAATATAAAAGCAAGATTTGTATGTGAAATATGATTGTTTGTACTTTCAGATAAGAGAATTGCTCTGGTCCTTCTTTTTGTACCGCAAGGTACTAGTACCGCGAGGTACTGAAATCCCAACCGTTGATGATGACAGATCGAATGGTCAGGATTAGTTGGTACCTCGGGGTACCGACTTGGTCGTGGTGTGGCCTGCGCGATTGAGCAAGGGTGGAAGGGTAGAATCGTAGTTTCGCGTTTGGAATGAAGTTTGGATCGGTGGATGGTTTGGGGAAGTGCGGCATATCCTTCCAGACT

>Os12t0635700-01 2000 bp upstream sequence

CTCCTGCGCGTAGGCCGAACTCGTCGGCAATGACGCGCTCGACGTCTTTGGCAGTGGCAGACGCGGGGGGGGGGGGGGGGGGCATCGCCCAAGTGACCGTGGCCGTGCTCTCCCACTCGCACAGCTCTGCATCGATGGCATGCGACGTCGGGATAAAGACGACATCCTCGAGGGGGCGCGTCTCCGGGTCACCCAATTGCGCCATAACACCAGTGGGTGGCGGCGGCGGTGGCAGAAGCTCTGCTTCTCGGCAACTGTGGAGCAGGGGGAGGGGCGGCATGGGGGCGCGCTTGTCAGGCGCTCCCTGGCATTTCTTGCGTGGTGGAGGAGGATTCCGGCGGCGGTCACTCAAAGCATGTCGGCGGCGGGGGGATGGGAAACGGGATCTGCTTGTTCTTGGCGAGGCGCAAGTAGCTCTGGGCAGGCGCCGCGAGCGGTGTGTGGGACGCGGGGAGCGAGATCTGTCTGCTCTTGGTGTTCTTGGGAAAGCATGAGAAGCTTTTGCGTCCAGACGCAGCGGGCGGTAGGTGAGATGGGATGAATTGCAGTGGCGCGCTCGGTGCCCGGTGCGAAAACAGGTGAAACAGCGCGGGGGGTCGCGGCACGCCGATGCCCGGTGGTCGCGGGCTAGACAGACGAAGCAACGTCCCCTGGTTCTAGCCTTGAACTCCTCCAAGGAGAGGGCGGCTCTCCTTCCAGCGCCGTCTTCTCGCCTTGAATCGGGCCGTTGGCCATGGATTGAATGCCCTTTCTCTCTCCACCAGCGCCGAGGTTTGACCTCTGTCCATCCTGGCTCGGCCGGCGTGAATGCGGGGCGTGGCGTCGTACGCCTATGAGGGAGGATGTCCCTCCTCTCTCTCGGAGCCAGCTCGTAGGCGCGGCGGTAGGCGTCGCGTCGCGGCGAAGGCACAGTAAAGACGGACTTTAATGTGGGACGCGGCGGTGGTTGGGGAGCAAGCCGACTGGAGCTGGCTCCCGGAGACCTGCGTACCGCATCGACGAAGGAGCGTGGGGGCACTGTGGACTCGGAGACGTGGGTAGTGCTGTCTTGAGCCAGGCGGACACCGGACGCTGGTGGAGGAGGCGGTGGCGGCGAGATTTCCCAGAACATGCCGGCGCTCGGTCTTGGCGAACCGGAGGACAGTGCTGGGGCGGCCGACGGCCAGGAGGCCGCCGAGAGGGGCGTCGGGATCTGCGGGCGACGGAGGAGCATGGGCGCCACCGGCTGGGGCGCCGACGGCGGCGGAGGTGGAGTGCTCGCTAGCATTTCAAAAAAAACCCTTCTATCTTTCTTTTCATTTTAATTTGTAAGTGAATCATCCTGCCCCTTTTTCTCAATTGCAAGTACCAAATTAATTTCTTTTTCTCTTTGAAGTATATGTACTTTCAGAGTTGTAAGCAATCCGAATTACATGAAGACCTTGTTCTTTTCTTATTATGTATTCTGGATTTTTGTTTACACGCTTTGCAAGTTTTTCTAAACGGTATGTTTGTGTAAAAAAAAAATTCTAAAAATTTTATTAGAATACTATTTAAAACAACTTTGTTAACATAAGAATTCAGATTATATTACTCAATTTTAGTTATCAGATTAAAAGGAGTACTAAAAGAAATTAAGATTATTCATTAATTTTAGCTATCCTAAATGTACAACTAAGAGTGTTTGGTCCTGGCAACAACCAAACACGTAAGGAAGGAGGAGCAAAAGAGCACGCGAATCGTGCCTCTAGAAACCCAAAACAGCGAGCCTAATTTTAGCCGCTCCGGTCCCTTCTCCTGACGTTTTTTTAAAGAAAAGGATGAAAATTTAGAGAGAGTCTCCTGTCAAACTCACCCTTTTTTTATGCAAAAGGCCATCTAGAACTCTAATTATAAATCAATAAATTCGTCAAAGAAAATCAAAAATCAGAAACGAGAGCTTGAGCTGACCGACAAATTCATCCGAATCCAATAAATAAACTCGAGGCTATATATATACACACACGGACGAATCCCTTTCC

>Os01t0227700-01 2000 bp upstream sequence

ATACCTCCCATATGCCTTGGCGGAGGCGATGCGATGAAGGGCCGCATGGCATGAGGGTGATGAAGATATTCCTGGCGACGTCTGCAGTGAGGAGCAGATCACCGCTGAAGGCGTCGGCGGCCACCTGGCGACGGGAAGGGATTCGTGCGGCGGGGATCGGTGGCAGATCGGATCACCGCGGAAGGCGTCGGTGGCGACCTGGCGTCGGGAGGACTTCGTGCGGCGGGGATCGGTGGACCAGGACGGTGATCGGCGACGACGTGAATCCTGGCAGAGGACGGGTGATGGGGATCGTGTAGGGCGAGCCGGCGGCGGGGATCGGTGGCAGATCGGCGATGCAGATGACGGCGCGGCGGCTGATAGAGAGGGGAGCACTGATGGCGGAGCTCTTTCCGGCGTCAGCGCGTGCGGGAGCGGAATCCTGGCGGTGGCGCAGGTGCGAGTTGGAGAGCGAGAGAGAGAGCGGCGATTCCGCGATTGAGGGAGAGGGGGCGTGCGGTGATTGAGAGAGGTGGGAGGTGGGATCGATCTCCGCCGTTTGGATTTTACCGAGATGATCTGGACCCTTGACGCTTGAGTGATGAATGAAAGAATGAAAAATGAGTGAAACAACTTTTTTGAACTATAGGATGTATAAATAAATAAATAAGAAGATGAATATGAACATATATACATCGATATCCCTTCTTATCTTATCCATTTGAGAGAGACGGGAGGGAAGAAGAGTTCAACATACAGGCTATCATCACTATTCAATTTGCAGTATCGTCCAGAAATGAAGTACAAAACGTACATGCACCGGAAACGTACGTGTCAAGAGTTCTGCACAGTGTTAAGAGCGAATATAAACTCCTCTTGGCACACCCGGTGCACGAGGGAACCCGTACGTGCTTCGGTGGGCAACCATCATGTGCGGTAGGCATATGCAGCGACACCAAAAAAGAATATAAACTCCTCTTGGTATGCAGTAATAATAGAGCAGATAGATAAACTGCAAACTGGAGGCTCGCACGGATGCACTAGCTGCCATGTTACTTCCTTTGTAAAATAAAAACTAAATTTTCTATATGAATTTAAGCATGATCTATATGCAGATTTATAAGTCATCTAAATAGTCATATAAAAATTTTTTAAAAAAAATAGATTAATATAAAATATATCACTTTACAAACATGTAAATACCAATTCAACTTCTCCGTTCACGTTACTTAATAATAATAAATATAACTATGTATATGCATTAACTATTTTCAGTTTAATTTGTTTTTTGTTTTGTAACTTGTAGAGAAGTTCAATTTAGTTTTATATGTTCATGGAGTGATATATTTCATGCTAATATATGTTATCAAATTATTATAAGCTTTTCTATGACTATTTAAGTAGTATAAAAAAAAGAGGATATCCTTTCTAAAAATGAAAATCCAATTCCTGATATGGACGAATTGATGTCAACCATTCCAGGAGCTGTGCCCACAGATGAAAACCATCATGGATATATATATATATATATATATATATATATATATATATATATATATATATATATATATATGATTCAGATGCGTTTAATCTTTACGTTCCCGTTGAAATTGCTCCCACGGCTGCTGTTGCATGATCGCATTGCGAATGATGAGAAAATAGTATATCGTACTGGACATGTAATAAATAGGTGGACGTCTATTCGAGCTGTTAACAGTTTCCCATATCGTATATGTCAACCATGCCAGGAGCCGTGCCCACAGATTCGTCGCGAAAACCATCATGGATATATATATGATTCAGATGCGTTTAATCTTTATGTTCCCGTTGAAATTGCTCCCACTGCTGCTGTTGCATGATCGCATTGCGAATGATGAGAAAATAATATATCGTACTGGACATGTAATAAATGGGTGGACGTCTCTTCGAGTTGTTAAAATAGTTTCCCATATCGAACTCGTACGTGTCGCTCGTGCGACTTCCGCCAAATTTTCCCCAATCCGTTGTGCACGTACTTACGC

>Os01t0357100-02 2000 bp upstream sequence

AACGTTGATAAGTCACTACTACAACCATCGGCGACCTTTCTCGGGATCCAAGCATGTCGACCCCGCCAACGTGGCGTCGGTGCAGGGCACCGAGATGAACACCACGGGGCTATTTGCCTGTCCAGGGTCATCCTAGGCTTAAGGCCACGACACTCAAGGACGTGGTAGGCGGCGTCACAGAGGTGCTCCCAGCGAACAAGCTGGCCACCAAGGAGGACGCCGACAAGGTGGCGGCCACCGCTATGCAGAAACGATGGGAGGCATGCCGGTGACGACAAGGAGCTAACACGATCCATTTAGTCCCGATCCGAGTTTATCAGGAATTCAATCCTGCACCGTGCGGTTACGTTTTTCTTTTCCGCGGGAAAAGCAATCACCGATGGTAGGGACAAAGTGCGTGTGAGAACAGAGGCCAGGCCAAAGTGCGTGCGAGAACGGAGGCTAGGCCATCGCTGGATTGGATTTACGAATGAAATATCGATGTGACGAACAGAAAATTATCAGTTTGATTTAATTTTCATAATCAGAACTCTTTAATAGGAAAAAAATTACATGTACGTTCCTTCATCGTGCCCATGTCCATCTGGGAGTCCAGGTTTATTCACAAAGACCCAATCAACAGCCAGGAATCCATGTCCTTCCCCGCCGTTCCCTACTCTGCTTTTTTTTCTTTCATTTGAAACCTTCCGCTATGAATTTCTAGTCGTTCCTAGCATCCACGCACACAAAATAGATTTCCCTCGCAAGGCAAAACATACAAATATGAGTGCATGCAAGATATTACAAACCCAATCCATTAAAAATAGAAAATAATTAACTTTAGCCTACCTATCTCAATATTGGTATATGCCCAAACTCAAAAGGAGAAAAACCAAACTAAAACTTTTAATAAAGTGAACCCAAGAGATAAAAAGGTGATAGTAACAACAAAATCTCACTTGACAATGTCGTTAATCAACACTGTTTTTAAATATTACTTAAAAATCTTTATATTTACCTATTAAAACAATGAAAAACAGAAGATGTTTCTTTTTTATTTACAACAGCGTTGTATTTAGTCATGTCCTATCTAAGAGAGAAAAATGAATTTAACGAAAAGAAGCTCAGAAAAAAAAAGAGAACAGGGCCACCACACCAGTAATCCCTATGTTATCAATGAAAAAAAATTTCAATGCTAGGTTTTTTATAAGAAAAGGTGATAAAGTGTTGAAAAAATACAGCAGGAAATTATATATCTTGCTGGTTTAACATGAATTCAAGCATATAGATATAAAAATATATCAGGCTAGGAAAGGAAAAGGATAAAATTGGAGAGAAAAAGGAAAAGAACAGTAGAGGATAACCAGCAAAAAGATGAAAGGATTCGAACCCATGACCTAGCGGTACAATTGTTTCACAGGCTAACCAATTGAGAATCATCGACGTTGTGTCATCTTGTGTAGCTACATTTGAAAAAATATGTTTTGAGCTGAACGTTGGTGTGTCCGCCCCTGCATCCGATACATGTTGGAGCGTGGAGCGCGGTAAAGAAAAAATCCTATCGAACCTTATCTCCTTCTCTCTCGTCGCTTTCTGCGTCTCCCCGTCTCTCCTTCGCCAACAGCCGAGAAGAGGCAGAGAGAGCGCCGCCCCCCGTCCCTCTCTCTCCCTCTCGTCCTCGCCCCCATCCCTCTCGTCTTTCCCTTGCCGGCAGCAGAGGAGGCGGCAGCGACGGCTTCAGCTGCTCCCACGGGCCGGATCGGGCAGTGGCGGTGGCGTCGGCGGCTTCCGCTGGCGAATCCGGCGGGTGGATACAAATCAGTGTTCCGATAGGTAAAACCCTGCTCTCAGCATCTGCCCTTTTGAATTCGCCAAGAGCCAGCATCTGCCCTTTTGAATTCGCCAAGGGCCAGCATCTGCCCATTTGATTTTGAATTCGCCAAGAGCCAGCAACAGCGCCCCCGCGCCCCCTCCCTCCTCCGCAATAAACAGCCACACGCGCCGCCCCCATGTCCACCCTC

>Os01t0517900-00 2000 bp upstream sequence

CTTTAACTAATGTTTTCACTTTGGACCACCCACTATATTCTCAAGCATATGAACAATCTCGAATACTTCGGCTCCTACTCGCCAATGAACTCTCCCAAGTATATGTAAAACCATTTCTTTGGCATATGATAAGTTGGATGTAGATGTATAAAAGAGTTAGGGTGATCAAAGTGAGTTTTCAAAAAAAAAAACGAGGGTGATTAAAGTGCCACAAAGATAAAAATATCTGATCCAAAGTAAAAATATTTTTTAAATGGTGATCGAAAGCGAAACATCACTAATAAAAGTAAATTTACTCATGTAAAAACATGCATATTGAAATATTCAAATTGGACTGTGAATAGTGTACATGTGGAAGGGTTATACCTGACAACGGTATTTGTAACATATGGGGATTATTGATACTAAAGTATACGTGAGATTGTAGTAAAAAGAAGTGGAAAATAATTTTTGCACATGTAATGGCCCCCCTACTTATAGGTAATAGCCCTACATTTATTCGCCGAAACTGATGTTGCTATTATATATCTATATTTCACGAATACAATAGAGAGCACAATCTAATATAGTCTTTGTCAGCCTAGCAGATGTAAAGCTCTCACCTGTGATCGATGGTTAGTAGTAGTTGACTTTCTCAAATATGAGCTCAGGGAGATTAAAGACAGCTATAGATCCTTGGCGGCATGTTGTTACGAAACAATTGATTTATCTTTTAATTTATGTTCAATTTTTCATAGCTTTTTGGATGCCAGTTAAGAAACCCATTAACATTCCTTAAGGAGAAAAAGAATTTCTACGTTTATCATACGAGAAAGTTCTTCGTACCTAAATGGAACCAGAACCTGAAGTTTTTGGTAGGCCAGCACTGTAGCACGTCACCGCCACTCGCGAGTCCCATCCTGACCGTCGGATCAGACCACCTACGGCTCAGATACACAGTGCCACGCGTCTCACACATGTTGAACCAAACACCGACACTTGCCCCCCAGTACGTTGACTCCTCCGCGGCGGCCACGTCGCGCACAACGACGGCTGAGTATATATAGACTGGGCGCGTCGACGAGGTCTCTCCGTCAACGACGACAAAGAATCCGATTCCAATGGCGCGTATGGCGGCGGCGGCGACATTGCTGCTCGCCGTCTCCGGGTTGGCGTCGGGTCTGGTCGTCCCGGCCGACGAGCGGTGTCACTCAACGGACAACGCCGTGCTCGGCATCGACATCGGCGCGACCTACTCCTGTGTCGCCGTGTACTGCAAGGGCCGCGTCGAGATCATCCCCAACGACCAGGGCAGCCGCTTGACCCCATCCTGGGTCGCCTTCACCGACGGCGGCGGGCGGCTCGTCGTCGGCGAGGCCGCGAAGGAGCAGGCGGTGGGTAGCCCTGGGCGCGCCGTCCATGACTTCATGAGGCTCCTCGGAAAGAAGCTCGGCGACGACGATGTGCAGCGGGAGATGACGCGGCTGCCGTACGCCGTCGTGGACATGGAGGGGAAGCCGCATGTCCTGGTTGAGGCGGCCGACGGCGACGTGCGGGTGCTCAGCCCCGAGGAGATCGCCGCCGCCGTGCTCGCGAAGATGAAGAAGACGGCGGAGGCGCACCTCGGGAGAACGGTCTCGTCTGCCGTGGTCGCCGTCCCGGTCTACTTCAACGACGCGCAGCGCCGGGCTATCAGCGACGCCGGCGACATCGCCGGGCTCGACGTAATGCGCATTGTCAGTGAGCCGATCGCCGCCGCCGTGGCGTACGGTCTGGACAACGTGAGGAGCAATGGCAAGCGCGTCGTGGTGTTCGACCTCGGCGGCGAGAATTTGGACGTCACCGCGCTCGTGGCCGACGACGGCTTCTTCGACGTCCTCGCCACCAACGGCGACGGGTACCTCGGCGGCGAGGGCTTCGACCAGCGCGTCGTGAACCACTTCGTCGACCTCATCAAGCGGAAGCACGGCCGGGACATCACCGGCGACGGCCGCGCGATGCACAGGCTGCGACGGGAGTGA

>Os01t0880400-01 2000 bp upstream sequence

ATCGCCGGCGATTCCCGCGTGGCTACGTCGACTACCTGTACCTGTTCGACTGCGTGTTCGGTGTGGAGCGGCGGGTTCTTGGCTACGGCGTGATGGCGGCTTGGCTCGCCGTGCTGTTCTACCTGCTCGGTGACACGGCGGCGGTGTACTTCTGCTCCAGCCTCAAGGGCCTCTCGTGGCTGCTCCGCCTCTCCCGACGATCGCCGGCGTGACGCTCCTGTCGCTCGGGAACGGCGCGCTGGATGCGCTCTCCACCATCGCGTCGTCGCCTACGGCGGGGGAGAGGGGGAGACCACCACCGTCGTCAGGAAACTTGCAATTCGCTCACAACTCACACGCTCAACCTCACTGAATCTCTGAAACTCTGAAGATTGTAAGACTGTGAACAGTAAACTGAAAAACCGAAGAACAGAGTTCTGAACAGAGTGGTTTTTGTGCTGCTTAAGTCTCACCAGTCACCGCAGCTTCTCTGTTTTCTTCTCTATTTATTCATCTCCATCAGCAGTTCGTTACATGCATAACTGGAAAGAACAAGCCAGACTTCAGCCCCAGCCACTATATGCATGCAAAACAGAAATTAAAAAGCTCAAGATTGCACATCAGCGCACACGGCATCATCGCCGACGAACCACCAAGATCCCGACGCGAGCCAGCTTCCTCTTCTCTCGTCGCCATCGTCCCGCTCCCGTTCCCACTCCCTCCCCGCCGCCGACTGCCTCCAGTCGGGACTATCCAAGAAGAGCCGCGGCTGAGCGGGGACGAGGAGCCTCGCCCTCCTCATCGGTGCTGAACGGGCGCGAGTGGACGGAGCGGCAGCCGGACATGTCCCCCGACGCCGATGCAGTTGATGGAGATGTGGTCCAGCGACTCGCGAGCCGTCGGAGACCGCGCCGGTAACCGCAAGGGCGAGGATGACGAGCAGCATATTGGGCCGCCGCCGCCGCCGTTCCCCTCGTCCTCCCACTATCGCGAGCCTCCGCGGCCGAGCCGCCGCCGTCCTAGCCGCCGACTGCGCTGCGCCCCGATGCCGCCAGCCGCCGCCGCTCACCCCGTCCTCCCGCCGCTGCTGGCAATGAAGAAAGAGAGAACGTAGGAGAGGACTCACTGACAGGTGGGCCCCACTTTATTTTTTTAATTTATTTGATGACTAGAATGCCACGTAGGCACCACGTCAGCGAAACCGTCCTCCAAAACCACCGAATTGCACTTGTTTTAAGAGTTGAGGGGTCGAGGTATCCGGTTTTGTGGTTTAGGGTTACGGATTAGATTTCGATCACTTTTGAGGGTCACAAAGTAAACTTATTCCTTTCCTACGTGCAACAGATGTGCCCAGGCCGGACTTTGAGAACGTGCGGTCCGTAGCTGTTGGGTCGGAAACGGCCTAACGGGATCCCCACGGACGTGTGGGCTGTTGTAGTTGGGCCTATAAAGGATTGCCCGAAGGTTTCGGCCTCTTAGATCAGGCCATGGAACACTAACGCGCCGCACATGAAAGCTGGCCTAGATCCGGCGTTGTGTTCGTTTAGAAGGCCTCCCAATCCATCAGCCCATCTCCGGTTCTCCTCACATTTAACTCTGAGCTGAGAATGGCAGAAAAGTTGCAGACCTCCCCTAACCTTCGTGATGTGGACAGATCATGGGCGAGTACGGTCGTACCGATGAACACTTGACCCTCCGTATGGCCGTTGATGTTACCGGTAGGGTGATACTCCCTCGGTCCCACGATATAACAAATTTTAAATTTTTTCTTAGACCACTCGTTTCACCTCACCTCACCACCGCGCACGACCAGAGAACCACCACCCTCCTCTCGACACCGCCAAAGTCAAAAAGCAAAGCGTCACGTCACGTAGCGCAGAGCGGCTGCAACTCAATGCCAGGTGGGCCAGGTCATATACTGGCCCCACAGGTCAGTGAGCCTAGAAACCTCCTTCCTCCTGGTATCTGGAAGCTCTGGTATTTAAATCGCACGCGTTCCTCAGCCACCGCCCTCCTCCTCT

>Os01t0960500-01 2000 bp upstream sequence

ATGAGGCCGCAATAGTTAGTTGTAGATGATTTTTGTTCGCCTTCAGTCATATAAGCGCCCGTGGCCTAATGGATAAGGCGTCTGACTTCTAATCAGGCGATTGTGGGTTCGAGTCCCACCGGGCGTGTTTTTTATTTCTTTTTTTTTTAAATCGAGGGCGTGTATTTTTTGTGGGTTTGAATGTTTTTTATTTCTTTTTTTTTAAATCGAGGTGTTTTTTATTTCTTTTTTTTTCAAATCGAGGGCGTGTATTTTTTGTGGGTTTGATACTAATATTCTCTACTTTTTTTTTGTGCTCCCGAAACATCATCATTACTTAGTAGTACCATCTAGCAAAAAAAGAGAATGTAATTAAAGATGACGGTAGGGGCGGGTTGAGTGTACCACAAAAAAAGCGAATTTCTTGAACTACTTTTTTCGGGTAAGAAGATTAATACGATGACACGACGAGTGTGTCGTCTTTGCCATCATCTTGCGACGTGCGGCGTCGTCTAATATGTCATCATCGCTCTCGTGATGGTTGCTTTCACGCAATGTTCACCTAGGAGTATCTCAATCAAACTAATTTAGCAACATCTAAAATATTAAATTAATTTTATTAATCTAAAATTGAAAATATTTGTTTTGTATTAAATTACAATTACATTTTTACATGAATTTAATTAAAATTAAAGATGTTTAACTAACAAAAATATAGATTTATAAACAAGGGAGTACTCCCTCCGAAAAAAAAAACTAGGTTTTCGTGTCCAACGTTTGACTGTCCATCTTATATGAAAATTTTTTATAATTAATATTTTCATTGTTGTTAGATGATAAAACATGATTAATACTTTATACATTACTTATCTTGTTAATTTTTTTAAATATAACGGACGGTTAGAAACCTAGGGTTTATGGGACGGAGGGAGGAGTACCACATAAGCAGCCTTGTTCGTTGACCATTCGCTAATCCAGCTCCTCTCTCTCTCTCCAGTTCAGACTCCAGCCCTCGTGGGTTCCTTCCACCACTTAATTTTCAGAATATAACACCCAATACGCACTACTTCTAGGATTTATCACCCAATTCGCACTGCTTTCAGAATACACCACTGGAATGCGAATTTTCTTCTTTCCGTAACACTCCGTCCCCTAGCGTCAGTCGGCCTGTCGTCCTCCGTCCTCCCGGTGCCGTTCGTCGTCAGTTCGCCGCCGCTCGCGCCCGCGTCAGCCCCGCCATCACCGGTCCATCGCCGCCCGCGTCAGCCCCGTCGTCGCCGGTCCGTCGCCGCCCGCGTCAGCCCGCCGTCGTCGCCCGCGTCAGCCCCGCCGTCGCTCGTCCGTCGCCACCATCGGCGCACGCGGTGGAGGGCCGGCGGAGAGGGGAGCAGCGGTGGAGGGCGGAGGGCGTCGAGCTCCAGCGGCGGAGGTTGGACCGGCGCCGGCTCGGGGCGCGGTGGCGGCAGCCCGGTGCGGCGGCGGCTGAGCTCGGGGACGAGCTCGCTGGCGTGGGGAAGGACGAGCTCGCCGGCGTGGGGGAGGACGAGCTCGCCGGTGGGGGGAGTACGAGCTCGCCGGTGGGGGAGGACGAGCTCGCCGGCGTGGGGGAGGACGAGCTCGCCGGCGTGGGAGAGGATGAGCTCGCCGGCGGCCAGATTTGTGCGGGCAAACGGCGCGAGATGGATGAACTGACGACGGGCGTCGTTGGGAGGACGGAGTGTTACGGAACGAAGAAAATTCGCACTCTAGTGGTGTATTCTGAAAGTAGTGCGAATTGGGTGATAAATCCTGAAAGTAGTGCGTATTGGGTGTTATATTCTGAAAATTCTCCCACCACTTAATTTTCACACCACTTAATTTTCACACCACTTAATTCGAGTGTCCCCCATGTAGTACAGTAGTATCTTTTCCTTGCTCTTCCTTCTGCTTGGCTGTGGTGGTGGCTGTGGTGGTGGGCAAAGCAAGGGCAAAGGGCAAAGGGCAAAGCAAGCTTAATTCGAGTGTCCCCCAACGTACGTATT

>Os02t0175100-01 2000 bp upstream sequence

AGAGGTTTTCTACAAAAGAAAAAGGAACGGCATGGTACGGCTGGCGCTGTTCAGGTTTTGCCGGTTAGAGCATAGCAGTGTACTGTTGCATTGTGATTATAAATCAATGCAATGTTATAGCAATTATCAATTATGAAAAAAGAATATAAAAGTGTCATAAAAAATGTAGTTTAAAATAATTAATGAACTCCCAAAAAATTCATTTTGTACTTATACGTAAAGGTGCGTTGCTCAGACGTCCATTTCTCCAATGCTACAATCATCGGGACCATGTGTTAGGGACATCATTCACATCCTACACGCGTAGAGCGGATTAATTGTCCAAGCACAAATATGCATTTGTGATAACTATGAAGGTGCTTCGATAAATCATTGAGGTTAAATTTCATTCACCTAACCATTACCCAGTGCAATGATATCACAGTTTGTCACACAAGGGTTGAGAGAGAGTGTGTGTTTTTTTATCCATCCTGATTATCATTAGCCTAATAAATAATTTATCTATCATGAAACATTACTATATACATGTTAACTCTAGACTTTATAGCATCTAACTGTTCCTTTGCCCGTAACAAAGCATGTGTATTTTTCTAGCATAACAGTAACATGATTAAAATATCCTTCACCCGTTGCAACGCTCGGGCATTTTTAAAATACTCCCTCCATTTTAAAATGTTTGACACCGTTGACTTTTTAGTACGTGTTTGACCATTCGTCTTATTCAAAAAATTTAAGTAATTATTTATCCTTTTCATATCATTTGATTTATTGTTAAATATACTTTCATGTACACGAATGGTCAAACATGTGCTAAAAAGTTAACGGTGTCAAACATTTTGAAACGGAGGGAGTATGAGAAAATGATTTATGAAATATTGATTCATTGTTATCCCTGAGTAAATGGATAGAGTTCCAAGCACCATCGAATCTCGTACTTGTAGTCCGTCACCACCCAAAAACCTTAGAGGAGGCAGCGGCCAGGCGCGTGGAGGCCTGCGACAATCGTGAGGCTTCCTACGCCTAGTGCGGTGAGCGGCGGTAAGGCACGCCATGGTAGGTGGTGACTGTGGGGCTTGTTGCGGTGAGCGGCGGGTGAAAGGGTCATGGGATTGCTTCTATGCGATCTAGGGAAAGGGCAAAGTTGCACGGGTTGAAAAATATAGGGTCAAAATACAACAACAATCGAGAATATAAATGTTCTTTCGCGCTAGAGTTAACACCGTCAAACCTACCGTCACCCGTGAGGGCAAACAACTAAGGGGTATTTAGATACCGGGGTATAAAGTTTTGGCGTGTCACATCGAGTATTATATAGAGTCTCGCATAGGGTGTTCGGACACTAATAAAAAAACTAATTACAAAATTAATCAGTAAACCACAAGACGAATTTATTAAGTATAATTAATCCGTCATTAGCAAATATTTACTATAGCACCATATTGTCAAATCATGGAGCAATTAGACTTAAAAAATTTGTTTCGCGAATTAGTCGCAATCTATACAATTAGTTATTTTTTAAGCATATATTTAATACTTTATGCGTGTGTTAAACATTCGATGTCACATGATAAAAAAATTTGAGTGATCTAAACAGGGCCTAATTCATTTTTCAAAAAAAACAAGGACAAAATGCAAACCCTAAAACGCGAAAGAAAACAATAGTTCGGGGTTAAAAGTGACCAAAAATTTGCACCCGAGTGCTGTGTGACTGAGGACATATCACATGTGAGGTGCAAAAACGTGTGGCCACAGAAGATCACGGGCAGCTGACGTGGTGCGCCGTCACGTTGCCGCCGGTGGTCGGTGGTGCGGATCCGAGTCGGCTCCCATCACTGGGCCGGCGCCGCCATTTTCCCAAACTACCCCCGACGCCGCAACGCATTTCGCGGCGGCTTCCAAAGCAGTATCGTAAATACGTAGGGGAATGAGGGGCAAAAGGGAGCTGCTTTCCTTCCTCGCAGCGATCAACACAAAAAGCTTCTCTTTCCCTTCTCCTCCTC

>Os02t0199666-00 2000 bp upstream sequence

GTCACAACCCTGCAACGGCATGGTGTGCGCCTATGATTTAAAAGGTGCTGCTGAAGTTTTGAATCCAACAACAAGGAAGCATTTGACGCTGCCAGCTTCAGAAAGTGTATATCAGGCTCAATATTCGGAATATTTTCTTGGATATGTGCACTCTACAAAAGAGTATAAGGTAGTCGCTCTCCGCCATTGGATAAAGCACTTGACATTTGAAGTCTGCACTATTGGCACGTTGTCATGGAGGACGGTACGTGGATCTGAAGAGGAAGAACTCCTGAAGACAACAAAGCCGGTTGTTGTTAATGATGAAATGCATTGGCTACTTCTTGATGATGAATCATCTCACTTTACTCGAAAAATCCTCTCATTCAACTTGACAGATGAGAAGTTTTCATATCTTGATGTCCCAGACAGTGTAAGAGACCGTGATTTGGAATTAGTCGAGGGGGAAGGGAAACTTCATTTATGGTCTATGCCTTGTAAAGGGGCAGCATATACAGAATCAGAGATTTGGCTGGCAGACTCAACCCGGCAATTCTGGGTTCACTTGCACAATATTGCCCATCCCTCTGTTTTGGGCACGAAGCCATTTTTCATGTACAAGAGCAAGCTCTTTCTGGGGAGCCAAAAGAGATTCATCTATATTGATATTTTGGATGGGACGGTTTGCTACGTTGATATTCCTTCTGGTGAAAATATCATATCTTCTGGCATGTTTGTGGAGAGCTTTGTACCTGCTTTGACAGGCACAGGCTTGGTGAACTCAATGACATTATTAACCGGTTCTCGTTATGCTGGGTCATCATCAAGAGGCTCTGGACCATCTTCTCGTGCTGCTGGATCTTCCTCAACAAGAACTCGACGTTCACCCGCTGCCTCCAGGTGGTCCTCAGCAGTTGTGCAATCCTCCAAGCGGGCGAAGAGAACAATAAACTTAGTGTGGAAGATGTATACAGAAGGCACAAGCAAGATTCAGCAGGGGCTATGAAATAATCTCTTTGGACTGTGCAGCAGCTTGAAGCTACCTACCTACATTCCAGCAAAAGCTATTGGTCTTTGTATATGCCTATGGCAGAGAAGAAGCAGCTATTAGCAGTTTATGAGTAATGCCTATCTTGGAGAGAAAAAGGTACACCATGGAAGTGGTTTGGTAATTTGGACAGAAGATAAAGATCAGAAAAGATACAGTGATATGTTAATGTGTATTTCACATATGTTTTGCTTTGCTTAAGCCCCTGCCTATGGGGGCCTTTATACCAGAAAATGGAAAGCTATATGAAATAAACCTGTATGATTAAGGTACTCTTGTTTTGATTTGTAGTTGTAAACTGTATGCCTCCATTGCACCTGGAGCATCAGTTAATCAGTATATGTGGCAGGAAAAGATAAATACAAACATCCTTTTGTTTTTGGAAACTATATCCATACAAACTGTTGTTTAAATACATATCAGATACAAACTGTACATGATGCTTGTGGTTAATTTTGGTGTAATTTGTTTCCTTCAACTATATTAGTATATATGAGAACTGATCCTTCCATTTATGAGATATATATATATATATATATATATATATATATATATATATATATATATATATATAGACACACACACACACACAGAGAATGTTTGGGATTTTGTTGTTCAGACGGTTGGCAAGGACAGCAATGGCGGGAAGCCGAAGTATGCCCAGGCGATGTCGCCGGCGGCGCACCGCGCGCGGCAGATCATCTCCAGCTCGAAGCTCTTGACGTCGATCCTGTACAGCTCGACGTCCCTGCTCCGGTAGCTCGTCATGAACGCCGGCGTGCCATGGCTGACCTCGAGGATCAGGCTCCCGTTGGCGGCGGCGCGGATGCTGTACGGCCGGCCGTGCGCCGCCGGCCACGGCAACGGCACCGTCCGCTCCAGCCGCCACCGCGGGCCATCGCCGCCGTCGACGATGGTGTAGTAGTAGAGGCACGCCTCGCCGCCGGCGCGGTAGAAGTCGTGCGCGAACACG

>Os02t0522300-01 2000 bp upstream sequence

AAAATTGACCGCAATTAACAAGTATACTATAATTGTAGTTTTTTTTAAAAAAATATCGGTTGCCTGGTCAGATAGGCTTATCTCTCTCGCTATACATTACGATTGAAAAAGTTAATGAGAATTCTAATATTGGAATTGCTATACACTATAAATTAAAAGTTAATGAAAAAGTTTTCTTGGTTTTGGACTGAAAGCCCATTCCTTCCTTCTTATTTAGCTATAGGCTAGTCTGTTCTTTCTGTTTTCCCAATATCAGAGCGTAAAATTTAGGATTAAAAGACCAGAAAGATCACCGATCTGTCATCAACGGTGACGGGAGATTCAATCCGGCAAATTAAAGCTGAATTGAAAGGGAAAACTTGAGAAAATGATGAGGGAGGAGTGGAGAATCGATTTTTATAATCAATTGGAGGAGGAAACGCAAGATCGACATGGCGGCGGCGCTGTGCTAGGATTCGGCCCGGGCGGCGGCAGGAGTTCGGATTCGGGAAAAAAAAAGCGACAAAAGACCAGACGAAAACAAAAAAACAACGGACCAAAATATACCGGAAGCTTTGTGTTTTTGTTTTTTTATTAGATATAGATATAGATATATATTTTCTGAGTAATCGATCATAATTTTTCAACCATTTTAAATGGCTCAAAATAATGCCAAAGAAAATTATGTACATAACCAAATGAATTAAATCGGCGAAATTAAATAAAATCTTATTTTGGCCCATTACTTCCGTAACCGGCCCAAAAAATTGGCGTGGCCCGTTTAGCGCGTGCACACGAGATGATCGCGCGGCGAGCGGCCTATCTAACGGTGATGGCCCAACGCGGGGCGAACTGAGCATAGCCCAGTTGTCTTTTGTGGCCCAACAGTGGTGGCGGCCCGACCTAGGGCCGATCTGAGCCGTCCGATCTGATGGATGGTTTGGATCGGTCCCGTACCGATGAAATAACCGGCCGAAGGGGGCATACGAAAACCTTGATCTCTGACGAAATCGCCGGCTGATGAAATCCCCTTTCTCCCCTACTCTCTCCCCGATCCAATCTCCGGCGACGCAAGAGAGTGGACGGCGATGATCCAGTCTTTCTTCGTCTGTCTCCCTCCTCCACCCAAGTTGTCACCGACCAAATCGACCACCAGCTCTAAGTCGCCTTTGCTGCTTGCGTGGATCCTGTCGTCTCTCCGCCTCCCTCTCGCGTGGATGCGTCGTCGATGGCGATGGGAGGGGAGGATCTGGCGACCTCTCGGCAATGGCCCACCGGTAGCTACGATGGCCGAGACGCGCGGGCGGTGGATCTGGTGTTAGTGGAGGCTCGAGGGCAGTGGCGAGGCTAGCGGGCGACGGATCCACTGGCAGGGGAGGCTCGAGGACGCGGAGGCAGTTGAGTTCTGGTGGCCTCATCCTGAAGTTACTGCAGCCGCCTGCTTCCTCGACATCGCCACCCATGGGAGTCGCCGCCGTAGTGAATCGCCTCCCGGTTTCTTCATTGATGCTCCCTCAATACCTCAACACCCAAGAACCTTGCCGATAAGGGCCGCCGCCATGTTAGATCCGTGGAATCTTGTTGTAATTATGGGGTTGATTCCATCCCTTTTGTGTTGAACTTGTATCCCTCTTCCCTCGTTTTATTTGATTTTGTTTAAGTGGGGAAGGACGACGTACGGACTCTCGGACTTACGGCCGAAAATACGGAAAAAAAGAGAGGTGATGATCAGAAAGTTTTGAATTTTTTAAATAGTTAAGGTTAAGATTTTTTTTTTTGAAAAACCGACATCTGTTGTATATTTATAGGTCCCGTTTTTAATCTATACAAATCTTGTAGCGTATGTTCCTTAAAAAAAAACCGACTCCTATTAAAAAACCGGAGCCAACCTTATCCCCACATGCCATAGCTCACAGACGCCATCCATCCATCCTTATCCACAGACCACAGCCGCCACACTCCCACCCACCGTCAGCGCCACCGCACACCATCTCCTTGCCACCACCATCCCTACCTCTGTC

>Os02t0522300-02 2000 bp upstream sequence

AATTGACCGCAATTAACAAGTATACTATAATTGTAGTTTTTTTTAAAAAAATATCGGTTGCCTGGTCAGATAGGCTTATCTCTCTCGCTATACATTACGATTGAAAAAGTTAATGAGAATTCTAATATTGGAATTGCTATACACTATAAATTAAAAGTTAATGAAAAAGTTTTCTTGGTTTTGGACTGAAAGCCCATTCCTTCCTTCTTATTTAGCTATAGGCTAGTCTGTTCTTTCTGTTTTCCCAATATCAGAGCGTAAAATTTAGGATTAAAAGACCAGAAAGATCACCGATCTGTCATCAACGGTGACGGGAGATTCAATCCGGCAAATTAAAGCTGAATTGAAAGGGAAAACTTGAGAAAATGATGAGGGAGGAGTGGAGAATCGATTTTTATAATCAATTGGAGGAGGAAACGCAAGATCGACATGGCGGCGGCGCTGTGCTAGGATTCGGCCCGGGCGGCGGCAGGAGTTCGGATTCGGGAAAAAAAAAGCGACAAAAGACCAGACGAAAACAAAAAAACAACGGACCAAAATATACCGGAAGCTTTGTGTTTTTGTTTTTTTATTAGATATAGATATAGATATATATTTTCTGAGTAATCGATCATAATTTTTCAACCATTTTAAATGGCTCAAAATAATGCCAAAGAAAATTATGTACATAACCAAATGAATTAAATCGGCGAAATTAAATAAAATCTTATTTTGGCCCATTACTTCCGTAACCGGCCCAAAAAATTGGCGTGGCCCGTTTAGCGCGTGCACACGAGATGATCGCGCGGCGAGCGGCCTATCTAACGGTGATGGCCCAACGCGGGGCGAACTGAGCATAGCCCAGTTGTCTTTTGTGGCCCAACAGTGGTGGCGGCCCGACCTAGGGCCGATCTGAGCCGTCCGATCTGATGGATGGTTTGGATCGGTCCCGTACCGATGAAATAACCGGCCGAAGGGGGCATACGAAAACCTTGATCTCTGACGAAATCGCCGGCTGATGAAATCCCCTTTCTCCCCTACTCTCTCCCCGATCCAATCTCCGGCGACGCAAGAGAGTGGACGGCGATGATCCAGTCTTTCTTCGTCTGTCTCCCTCCTCCACCCAAGTTGTCACCGACCAAATCGACCACCAGCTCTAAGTCGCCTTTGCTGCTTGCGTGGATCCTGTCGTCTCTCCGCCTCCCTCTCGCGTGGATGCGTCGTCGATGGCGATGGGAGGGGAGGATCTGGCGACCTCTCGGCAATGGCCCACCGGTAGCTACGATGGCCGAGACGCGCGGGCGGTGGATCTGGTGTTAGTGGAGGCTCGAGGGCAGTGGCGAGGCTAGCGGGCGACGGATCCACTGGCAGGGGAGGCTCGAGGACGCGGAGGCAGTTGAGTTCTGGTGGCCTCATCCTGAAGTTACTGCAGCCGCCTGCTTCCTCGACATCGCCACCCATGGGAGTCGCCGCCGTAGTGAATCGCCTCCCGGTTTCTTCATTGATGCTCCCTCAATACCTCAACACCCAAGAACCTTGCCGATAAGGGCCGCCGCCATGTTAGATCCGTGGAATCTTGTTGTAATTATGGGGTTGATTCCATCCCTTTTGTGTTGAACTTGTATCCCTCTTCCCTCGTTTTATTTGATTTTGTTTAAGTGGGGAAGGACGACGTACGGACTCTCGGACTTACGGCCGAAAATACGGAAAAAAAGAGAGGTGATGATCAGAAAGTTTTGAATTTTTTAAATAGTTAAGGTTAAGATTTTTTTTTTTGAAAAACCGACATCTGTTGTATATTTATAGGTCCCGTTTTTAATCTATACAAATCTTGTAGCGTATGTTCCTTAAAAAAAAACCGACTCCTATTAAAAAACCGGAGCCAACCTTATCCCCACATGCCATAGCTCACAGACGCCATCCATCCATCCTTATCCACAGACCACAGCCGCCACACTCCCACCCACCGTCAGCGCCACCGCACACCATCTCCTTGCCACCACCATCCCTACCTCTGTCGC

>Os02t0522300-03 2000 bp upstream sequence

TAATTGTAGTTTTTTTTAAAAAAATATCGGTTGCCTGGTCAGATAGGCTTATCTCTCTCGCTATACATTACGATTGAAAAAGTTAATGAGAATTCTAATATTGGAATTGCTATACACTATAAATTAAAAGTTAATGAAAAAGTTTTCTTGGTTTTGGACTGAAAGCCCATTCCTTCCTTCTTATTTAGCTATAGGCTAGTCTGTTCTTTCTGTTTTCCCAATATCAGAGCGTAAAATTTAGGATTAAAAGACCAGAAAGATCACCGATCTGTCATCAACGGTGACGGGAGATTCAATCCGGCAAATTAAAGCTGAATTGAAAGGGAAAACTTGAGAAAATGATGAGGGAGGAGTGGAGAATCGATTTTTATAATCAATTGGAGGAGGAAACGCAAGATCGACATGGCGGCGGCGCTGTGCTAGGATTCGGCCCGGGCGGCGGCAGGAGTTCGGATTCGGGAAAAAAAAAGCGACAAAAGACCAGACGAAAACAAAAAAACAACGGACCAAAATATACCGGAAGCTTTGTGTTTTTGTTTTTTTATTAGATATAGATATAGATATATATTTTCTGAGTAATCGATCATAATTTTTCAACCATTTTAAATGGCTCAAAATAATGCCAAAGAAAATTATGTACATAACCAAATGAATTAAATCGGCGAAATTAAATAAAATCTTATTTTGGCCCATTACTTCCGTAACCGGCCCAAAAAATTGGCGTGGCCCGTTTAGCGCGTGCACACGAGATGATCGCGCGGCGAGCGGCCTATCTAACGGTGATGGCCCAACGCGGGGCGAACTGAGCATAGCCCAGTTGTCTTTTGTGGCCCAACAGTGGTGGCGGCCCGACCTAGGGCCGATCTGAGCCGTCCGATCTGATGGATGGTTTGGATCGGTCCCGTACCGATGAAATAACCGGCCGAAGGGGGCATACGAAAACCTTGATCTCTGACGAAATCGCCGGCTGATGAAATCCCCTTTCTCCCCTACTCTCTCCCCGATCCAATCTCCGGCGACGCAAGAGAGTGGACGGCGATGATCCAGTCTTTCTTCGTCTGTCTCCCTCCTCCACCCAAGTTGTCACCGACCAAATCGACCACCAGCTCTAAGTCGCCTTTGCTGCTTGCGTGGATCCTGTCGTCTCTCCGCCTCCCTCTCGCGTGGATGCGTCGTCGATGGCGATGGGAGGGGAGGATCTGGCGACCTCTCGGCAATGGCCCACCGGTAGCTACGATGGCCGAGACGCGCGGGCGGTGGATCTGGTGTTAGTGGAGGCTCGAGGGCAGTGGCGAGGCTAGCGGGCGACGGATCCACTGGCAGGGGAGGCTCGAGGACGCGGAGGCAGTTGAGTTCTGGTGGCCTCATCCTGAAGTTACTGCAGCCGCCTGCTTCCTCGACATCGCCACCCATGGGAGTCGCCGCCGTAGTGAATCGCCTCCCGGTTTCTTCATTGATGCTCCCTCAATACCTCAACACCCAAGAACCTTGCCGATAAGGGCCGCCGCCATGTTAGATCCGTGGAATCTTGTTGTAATTATGGGGTTGATTCCATCCCTTTTGTGTTGAACTTGTATCCCTCTTCCCTCGTTTTATTTGATTTTGTTTAAGTGGGGAAGGACGACGTACGGACTCTCGGACTTACGGCCGAAAATACGGAAAAAAAGAGAGGTGATGATCAGAAAGTTTTGAATTTTTTAAATAGTTAAGGTTAAGATTTTTTTTTTTGAAAAACCGACATCTGTTGTATATTTATAGGTCCCGTTTTTAATCTATACAAATCTTGTAGCGTATGTTCCTTAAAAAAAAACCGACTCCTATTAAAAAACCGGAGCCAACCTTATCCCCACATGCCATAGCTCACAGACGCCATCCATCCATCCTTATCCACAGACCACAGCCGCCACACTCCCACCCACCGTCAGCGCCACCGCACACCATCTCCTTGCCACCACCATCCCTACCTCTGTCGCATCCACCTACTGCCACTGGCCCCCGTC

>Os02t0555900-00 2000 bp upstream sequence

CACTGAAGTTTGAAAGTTTGGTTGAAATTGGTATGATGTGATAGCAAAGTTATGTGTGTATGAAAAATTTGATGTGATGGAAATTGAAAAGTTGGAAGTTTGAAAAAAAACTTTGGTACTAAACAGGGCCATACTATAGGAGTACGTACTTATGGACTAGATCTGTATGCGGTGTTCTAGCACACCACATAGGACGACGACGTAGAAATTTGTGTCAATGAGGATGCGATGGAATAATAATTAGGACCATTTTGCAAATTGACTTTTACTCGTACAATTTATTTGTCACTTTACAAGGTTAATGGTTAATAAAATATATTTTTACAAGTAAGAAAATATTTAAGCACGTAGTGTAGAGTTTGCCTAGACCCCTAAAAATGTAGAGAAGGTGGTGCAGTAATAAAAACAATCAAAGCATCGGCAGATTAAAACAACGGAAATATTTAGTGTTTAAATAGAAGAAAAATGATTTTAAACCATTCATATGAACTAATCAAAGTTTTCGAAAATCCAGCTAAAGCTAGTACAAATTAGTGACTTAAAAACCGTGCGTGTATAGAGTTACAACCTTGGTGTCCATTTTAACTTTCAAGTGATGCTTAATACCTCTAATTTAAAATATAGCAATTTCTAACTATGGTCCCGTTCTTTTCTCCAACAAGGTTGGATGAAAATTAAGATTTTCGTGGCACGTTTTTCAAACTGCTAAATGGTACGTTGCGTGCGGAAACTTTCTATATGAAAGTTGCTCTAAAATATCATATTAATCTATTTTTTAAGTTTGTAATAATTAAAACTCAATCAATTAAACGTTAATACCACCTCGTTTTGCGTAAAAAAATCAATCTTCATCTTCAAGAGAAAAGAACACCACCTATATACTAGTATTATTTATGCTTTGGTCCAAATACTTGCATATTCCCTCCATCCCAAAAAGCTCAACCTACCCCTTCTAGGACAACGAATCTGAACTACCTTTTGTTCAGATTCATTGTCCTGAGAGGTCTCCCATCCCTTTTTAGGTTGATTTTTTTTGACAGAGGGAATACGAGTAAGCTTGCGTTGGAAGGCATAGGGCCTGTTCACTTTGATGAAAAAAAAAAAAACCTTACCAATGCCAAAAATTTGATATAGTTATCAAAATTTGGCAGCAAACTAAATATAGCCACTTTTTTTACAACTTTACCAAAAACTTGGTAATGTTAAAAATGACATCAAAAGTGAACAGGCCCATAATCACGAGGAATGCATGTACTTGTGTGAAATTAAATATAGTATTAAAAAGAAAAAAAAGAAAACTGCAAAACATTTGTAGCACTCGTATCAGTCCAAAACATCTCCTTTTCCTTGACGAACAGATCGGAGTACTCCACTACGGAGTACGTCATCACCCCCCTCGACGTCACCGGTAGGCTGTTTTGTGGGCCCCCACTCCACCACCAAACTCCAAGCGTCCCCTCGCACACGGCTCGCCTCGCGAAGATTCCTCTCTCTCTCCCTCTCCCGCACACACATTCCCCCCCTGGGCCCCACAACCCGCCAATCCGTACGGCCCCCAACACACACACATACCCACCGTCCAGGCCACGCCCAACCCGTGTCACTGTCACGTGGGCCCCACCCGAGATCGTCCCCGCTCGCTGTCGCGTGGGCCCCACCCCCCGATCCGCGGGGCCCGCGCCGCACCTGGCCGCGGGTTGCGACGCACGCAGCACGTAGGCAAGGCAGCTAGCTGTCCCGCACCCCCGCGCTTTTATTACTAGTAGTACTTCTCCTAGCGGCAGCGCAAGCAGCAGGAGAGAACCAAAACAAAAAAAAAAAGAAAAAAAAGGAAGTAAAAAAAAAGAAAGCGGCCTTCTCCTCCAGCTCCACCTCACGCGCGCTTCGCCTCGCCGCGCCTCGTCTCGTGCGGCTGCGGCGGCGGCGGCTGCTGCTGCTGCTCCGGCGGCGGAGGAGGGGGGAAAGGCTCGTTCGGGAATAGGGAGGAACGAGCGGAGGGG

>Os02t0715400-00 2000 bp upstream sequence

AACATGCTCGCAACTGCACAGCACAAAGCTTTCAACTACACACACCTCGTCGTGGCCCCACCCACCCCTCTTGCATGAATAAAACCAGCCTGTCCAGGCCATAGCTGTGAAGACAAACATTGCGCCATCACATTCTCAAGAAGAGGAGAAGGAGAGATCTGGAGAAGAAGAGGTAGGAGGCCATTGCCACCTGACCTTCTCCTCTTCCTCCATTCATCTTCTCTTTCTTGGGACATGGCGACCATCCTGGAGAACATCCAGAAGGCGAGGTTCTTGCCGACAAGGCCGCTCAAGGACGAGCTGCCGACGTTCCAGGGCGGCGGCGGCGGCGGTGGCGGTGGGGGTGGAAAGGAGAGCCACCTGATGGGGCTGAGGAAGAGGCTGTCCTCCTTCTCCGACAAGATCCAGCCCATCTCCTCCGCGTCGGCGGAGTGGGCGTTCCGGCGGTCCAAGTCGGCGCCGTCGCTGGGCGCGTTCGCCGGCGGCCCGCTGAAGCGGTGGTGGGACTGGGGCGTCGGCTGGCTCATGTCCAAGAAGCCCGGGTTCGCCACCGACCTCGAGATGAACGAGGAGGAGGTGGCCGCCCTCGGCCGCGGCAGCAGGGGCAGCTGGGGCCACATCCTCTACAAGATGCGCTCCGGCGTCCGGCGGCTCGTCACCTCGCACTCGCTGCCCACCACGCACAGGGCGGCGGCGGCGGCGTCGGCGTCGGCCCAGTGCAAGCCGGCGGCCACGTTCAACTACACCCAGAGCTTCCACAGCGGGCAAACCGCCATGGCGTACTGAGAGCTTCTCACATGTTCCTCCAGTGAGAAACATGACCTTTTCTTGGTCGCCGTCTCCATGGGATCCAAATGAAGAAGAAGCTCAGGGAGATTGATATAATATATGGTGATGAAGATGCAACTACGATTACATGTAAAATTACACGGTAAATTTGATGAATGATGAAAAAAAAATTGTAGCTCACTACGTGATCTGTTTTTTGTGTAGATCTAGTGTCCTGTAGAGGCCGAATGTATGTTCGTTCTTTCAATTCTTTTTGGAGTTCGTTGGGCGATGTAAATGTCTCCGGTCAAATTCCTTATGCTTTTGCCTCCTGCAGTAAATAAAAAGCTAATGAAATATGGTGCTTGTTTATTTGTTCTTGGGATGATCTTGCAGAGTTTCAGCTCAGATCTGAGTTCTCATTGTAATGAGCTACCATCTTTCTCTTTACCATGATCATTTTAGCATCTTTGAGTAGTTACATGTGTTTGGTGTATGAAGATCAAGAATAGCTGGTGAATCTAACAATTAATCTCACAAAAAAAGGGAGATTACAGGTGAGGTGTCACGACTGAACAGGTAGGCAGGAGGTCGGAATCTGTCACCATGTGTGTTAGTATTACATCATGAAAGTGTCCTCTTGTGAAGCACAAGAAAATTAGTGGTTCGGTGAGAGTGGCACAGTTTTCATTTAGGCGATGCTGTACTTTTTGCTCGTGCATTCCTCCAGCCTTCGATGGAGAAAGGAACGGACTTTGGCCCATAAAAACCAGGCCCAACTGGTTACAAAATTTCCAATCTCCTGGCTTCCAAAATGGATCGTGCACGAAAGCTGGGGCCCAACCCAACCCAACCCAGCCCAAGTCGGGAAATGACACTTCCTTTTTTGTTTTTCTCTTTTCCTTTCACGTTGTTCCATATCCCCCGTTCCGTTCTTTTTTTTTTTTGGAAAAAAAGTTCTTGTCTCTCGCGTCACGTTGCTCGCTGCGTCGCTGTCCCGTTTCGACGGTTTTTTGTCTTGATCGCGGTAGCGGTGTGCCACGTTGGTCCCGTGCTAAACTTTTTTCCGTTTTGCCCCTCACGTTTCCGCGAAATTACGTGCCTTCCTCGCGTCGCTTTCTTTCTTCTTCCTCGTCTCGCCTTTCCCTTTCGTGCAAGACAAAAAAAAAAAAAAAAAACCTTCCCCAACTCACAACAAAACCCCCCCAAACCTCGAGCGCGGAGAGCATCCC

>Os02t0724000-01 2000 bp upstream sequence

ACGTTGTTGGAGCCATTATAAAAATTTCATAAGATTTGCATTATTGGCCCACTTTATTTTTTTATTTGTCATTTCAAACACCGTCAATGTATAAAGCACACACACCTTTAAGACTACTCCCTTCATATAAAAAATACCCAGCCTAGTATTGGATGATACGTTCATCCAATATTAGATTGGTTTTTTATGGGATAGACGGAGTATAAGGAAAGATATACAGCCTATGATCTCGTGGAGCCGAGGTTAAGAGAACGTATAAAAACTAAAAATGATGTAATGTGACTGTTGTTGTCATTGCTTTATTTTTTTTAATGTCATTTAGTATGTTATATATTATCGAAGCTAATATATATAGACAGTACATATCTCTCATGTTCATACTTTATAGTACGAATGATCGTTTGGTGACGGTTGAAATGATGCAACGACCTTTCCTACCTTAATTTTCCCTCAATGCGAACTGCATTATATTGTTTCACAAAAGAAAGCGTTCCATCCTTCGTGACCAGCCAATTATCTCAAATATATACTCGTTTCATATATTTTTGTCAATGTTACTAACATTCAGAATCGAATGGAGTAAAAGAAGAACCCGTGAGTCACTTGCTTACGACACCCTATATATAAATGGCATGCTCATCCACTTGCTGCAAGTACACTATAATCAGAGGCTTCCGATGATCGGTCGTTGAGAATGCACTCCTAAATCGGTAAGATCGCTATTTCACCACGCAACCACAACTTCGTTATTTTATCTCTAAATTCACAAACTTTATTCCATAATTTCATAATCACATTATATTATATGATTTATATTATCCTTTAAACGTAGCACCTTCTATTAGGATAGATTAGACTAGGTGCGAGATACCATGACAAACATTGCTAGGCTTGATTGTTCATATATTGTGACAATCATAATATCTTCGTTATTAAATTTATGGTTGAAGTATAGCATCTGTCGCCTAATCTCTGCACCTCATCACTAGAGTTCGATTATGCCGTATATATGGGATCGAGATCCTCTAACTTTTAAGTCACATGACTTTGAGTCACTGACATGTGAGCCCTCTTTGTTATGGACCCACATGTCAGTGATTTGTCAAAGGATTTTAATCCGTATATATAAACTAGTTTGGTTACTACCCTCACACAAACATTGCATGGCCTGGTTTTTTCCTTGTGTGGTTGCTTCCGCGAGAACTCCCTTTGCCTGGATCTGTTTGTTCCTCGCACCATGTGAAAAGATCGGACGGAACGGCAGCCAGCCAGCCGGCAAGCCGCCGCAACGAGCGTCTGCACTTGGCGAATTTCGCCGTATAAACCAACAGCGCGAGGCAAGGCGAAGACGAGAGAGTGAAACGAATGAGTTGTTCAAATTGTAGTCAAAATAAAACTTACCAAATTTTGACAATATCAAAATTTTAATAAGTTGGCAATATTTATTAAAATTTTGGTATGATTTTCTTATGTATCTATTGAAGTTTAGTGAAAAACTAAACACATACTTTTTCCGTCCCAAAATATAAGAATCTATTACTGGATCCGGATAAGACATTTTCAAGTACTACAAATCTAGAAAGCCAAATATAAGAATTTAGAACCGGATGAAATATTTTTTAATACTATGAATTTGGACATACTATCTGTCTAAATTCTTATATTTTAGAACGAAGAGATTACATATTTTTGATAACTCTATAATTTAAAATGACATCAACCCCGTCACGACAAAGCGGTGTGCGAGTGAGACCTCCGCGAGGGGCAGAACAGTCAATCGGCTCGGGCCGCCACTTGGCGAAAGGACCGCGCACGTGGCGCCCGCCCTGGATCCCCCTTATCTCGGACGCAAATTACGGTCCTGCCCCTCTCTCTCGAGCAAAAAGCGCAAAAAGGGCACCCCGGGTGGGGCCCACCGCCCCCTCCTCCTCATCCACCTCCTCTCCCCTCCCCCCACTCGCTGCTCGTCTTCTCGTTCTCTCGCTCGCGCATCGTCTCTT

>Os02t0782300-01 2000 bp upstream sequence

CCTAATTGCTTTATGATTTGACAATATAATGCTACAGTAAACATTTGCTAATGATATATTAATTAGACTTAATAAATCCGTATCGCATTTTACAGCAGGAATTTATAATTTTTTTATTACTAGTCTATATTTAATAATTTGAATGTGTGTCTCTAAATCCTATGTGACCCATGGAAACTTTACATATGCATCTACGCTTCGGCCTCGGCACACGGCCCACGTCGTGTCGTCACCATCGTCACTTCGCTTTATCGGGACAAAGGGAGCGGCTGCCCATGGCCCACGGTGCACCCGTTCCACGTGAGCGCGCGCGCGTACCGAACTTTGTGGGCCAAACCCGCCGCACAGCCAGAAAGATGCCGCAGCCGCCGCATGCCGTCCACGCGCGCCACGTTTCGCGGCGTCCACCGTGAGCCAGGTTCAACGCACCAGGGCGGAGGAGAGTGACGTGGCAGTGGTCGTAATTTCACCTGATCTTCGTGGCCTATCTCTCGCAGGAGGTGGCGCGTGGTTGACGCGGTCAGAAAAGCGCGCGCGGCCGCCCTCGAGGCGCCAGCTGGGCACCCGCGCCTGAAACTTCTGGATGGTCGCCCCCGGAAATATCTCCGTCCTCCCAACCACATCTCAGCCGTCAACCACCCATCGGACGGATGGTAAAAAAAAAAGAAAAATGATCCGGTTAGATGTGGGGTGGTGGGCCCCACATGTCACTGGAAAGGTCTTGCACTGTTCTAGACACATCACACATCTGCTTCGAGGCGACCGTTGCGTTTTTTTTCCTGATGCTCCAGTGGAAAAAAGAGCGGCCTTGAAGAGCAAGTTTAACAGCGTAGCCAACTACTGGCTCTAATTTATCTATAGCTAATCTAATAGTTTATTTATACAATAGTTACATACTACATTATTAATACATAGTTCCATATATTATACACACACTGTGTCTTGGAGTCTGTGCTACAGTTGGCTACAAATCTGTAGCTCGCTGCCCTTCTCTCTCTTTATTTATCTTCTTAAAATATGTTTGCAGCTGGCTTATAGCCTGCTATTATGTGTCTTCAGGCGTGTAATCATAACCAAACTAATTAACCGCTAGTGAGCGTCTAATGGAGTAGCATCACCAGCTAAACGTATTTCAGAATTGTGGATCAGATCAACTTTTTGTCTAAGAATCTTTAGTTGCTCCGGCTTTCGCAAGTGTAAAAGGCACTCGCAATTGGTAACACACGCCATAGCCACACTGAAAAGGACAAGCATATTAGCAGTTGCCCCTACCTTTCAAACGATTCTTTCGCCTGATTAACATCTTTTTCGTCCAGGTTGAAAGTTGATAATCACAATCAATTAGGCCCCTTTGCAATGCATGGTTGTCTGAAAATTCAAATACAGTAAAAGTGCAAAACAGAAGATGCAGCTAGTAATTTCCCCACATAGCAGAAACCACATAAAACCGATCAATTAGGTATCAGGTCACAATTAACCATCCGGATAAGCTCATCAGGAGTGCATCTACTACCATCTTCTCGAATGATGTGAAAAAAATGGCAAATTGTCAAGGTTAACCGTAAATCTACAGTGCCAATTTCTGACGGCACATCCTACGAGCCTCATAGATTATTCATATTCTCTAATAAACAAAAAGATTTGTGAGAGAATAAAAATAAATTTATAGATAAAATGTTTATATATTTGTTCGTAGCGATTTAAAAGTCAATGTTAAAAATAAATTACGTTAAAAATATCTTAAAATTAACTTTAAAATTTAAATTTAGATTTTAATTTTGATTTATTAGCCAACCGACGAGACTCTACGGCTCTGTGCGCAACAGTAGCAGTGTCTGGAAGCAGGGATAGCTAGCTGCAGCTGTAGCAAAAGCAACGATGGCCAGCCAAGGCTGGGCGAGACGTGGAAATAAAGCGAGGAAATTCCTAGGCAGAAATATTTAAAAGGCAAGTGGGCATCATGGCCTTGTTTCTGGAGGCTTCCTGGACTGCGTGGAGAC

>Os02t0815700-01 2000 bp upstream sequence

AAAAATCACCATGTTGCAATTAAGCAGAGAATTATAACACCCACTAACATAACAAGGATTTCCTTGTTGATTTAAAATGATTGACAAAACATATAGATATGCCGGTAATATTGAAAAATTCAGAATGTAAGAGTTACACAAGCATTTCGCCCCAGTATACAGGGGAAAAAGGACAGACAAAAAGGCCACATGTAAACTACTATGAATTTGAGTAATTACCAATCCGAACTCGCAGCTTTCTCTCTCCAGTAGAGGGCTTCTTCTTCATGTAGCAGCTGTTCCAATCACCTCCATCCTGAGAGTAGTAATCCATTGGGTACTCCCCCCCAAAGTCCTTGTCAGCACAACCACCATTGCTGCAACCACAATCTGTGGAAGTTCCTGATCCTGAACAACAACCGGTGGCATCTCCACTGCCACATTCCCTACTCCCCTTCTCCCACCATCCAGGAACATAGCCCAGTGCAACATCAAACTCGAATGTCGTCAGCATTGGCTTCTTGAATCCTTCACCCCAGTCAATCGACAAACGTGGGCATGCAATCTGCACCCACGCATCCACCGAATCCCCAAACAGCTCCATCCTTGCCGGGGACAGCTCCGACATGAGCACTACTGTGTGTTCCAGCCCTTTCTCCTCCAAATGCTCCACCACCCTGTCGAGAACTTTTACGCTTCCCTGCCGCCCAAGGGTGCCTAGTATAACTCCCCAACTCTTGGCCTTCCTTGCTGCCAGCACCGCCTCCTTCCTCGCTTGCTTCATCCCGACATGGTCATACTCCTCCAGCACAAGCACACCCAGGAATGGATCAAAGCGGTAGGCCTTGACCCCGGGATTGGCAATCATGAATGCCTCGAGGTGGAATCTCCCGTCTGCGACGAACACCACCGCTCCGACCCCCTCGGACCTTTTCAGGGCAGGTGCTGTGCACCCAAGAACCTCGCCGGCGGACAGCGGCTTTGCCTGTGGCACGACGATGCCCTGGTAACCATCGTGACTGAGGATCTCACGCGCTGCGTGAACTGCCGAAATGAACTGCACGGTGCCGGCGATGGCGAGGCGGGGCGCATCGGCGGGGTCCGGGAAGGCGGCGCGGACAGCGTCGGCGAGGCGTTGGGCGTCGACACGGATCTCGACGAAGACGTAGAGCACGGGGAGGAGGGAGGAGGTGACGGGGACGAGGCAGGAATGGCCGTAGTGGACGAGCACGTCGGCGGCGAGCGCCTTGGCGGGGCGGTCGGCGAGGCAGCACGCGCCGTAGGTGGGATCGGCGAGGATGAGGACGTCGTTGGAGGGGTCCGGCTCGAGGAAGGGCGCGAGGAGGTGGGAAAGCGGGAGGGAGAAGAGGAGCAAACCCTCCGGAAGCTGCAGCGCGGTGCGGCGGGCGCCGGAGGAGCGGATGCGGTGCGCCGTCTTGGGGAGCTCGAAGTTGTACGCCGCCGGGAGGAGGCCGGTGGCGGCAGCGGCGAGGGTTGGGTCGGAGAGGATGGAGGGCGGGATGGGCGTGTGGACGAAGCGCTTCGGGGGCGGCTTCCTCTTTGGTCCCTGCGCGGCGCCCGTGGCAAGTACCAGCGAGTCCGAGGTGGAGGTCTCGCCGGCGTCCATGGCGGCGGCGGCGGCGGGTGGGAGAGAGCGGCGGCGGCGCGAGGGTTTGCTTCTCCAGAAGGAGAGAAGAGAGGGTATCAAAGGTTCACGAAATTAATTGGGCCGGACACGTGGGCAGAAAGCCCAGGAAGGCAAAAAGCCCACGCGGTCCGCGATCGCCGTCCGCTTCGCGCTGGGGCCCAATCCAACGGCCCATCGCTTTCGATCCAGGGAAAGTTGCGTGGCTAACCACACATCCACTCAGCCGAAAAAAGTGCAGGCCACGTGGAAGGGAGGCCAACTCCGCGGCCATTATCCATTCATCTCTCAGCCCCAAAATTTCTCAAAATTTTCCAAAAAAGAATTTTAATTTGATTTTACAATTTCTAGTCGACTTGTGAGCCGGGAGAAAAAC

>Os03t0108600-01 2000 bp upstream sequence

TGTGCAAGTTGGTAGGCAAGGCAAATCGTTGTACAGTACTCCGTATACTTGCAAAAATGACACGACGTACTTAGAGTCGTGGCACGATCGAGTATACGTATACGTACAGGACCACGTACTATATATACTGGACAGTAAGGATCGATACGTACGTACGGCTCACCGGGTTTGGTTAATTCAACTCTGATAAATCTGCGTTCAATTTCGTTCCTGCTCTTGGGAACTACTTCCTCTGTTTCACAATGTAAGTAATTCTATCATTTTCTATATTCATATTGATGTTAATAAATCTAGATATATATATATGTCTAGATTCATTAACGTCACTATGAATGTGAAAAATGATAGAATGACTTATATTTTGAAACAGAGGGAGTACTGGTAATCTCTCGCTGCGTCTCTGTATACTTGTGGAGTTTTATGACGACGACGTGGCGTACGGACACGTACGGTGCCAAATTAACAATTTCACATTATATATGCGTGGGCAGCGACAAAGCTAGAGTATATTAGGGGTGCCTAGGAAGCCGGTCAAGAAAATGTTTTAGCTATACCTCATCTATTTTCACAATATATACACCCCCTTAGTATAAATTTAGACACCCGTATCAACTTAAATTTGATCTCAAGTATAATAAATTAAGCAAGTGGCACCACCCTCCATTTCGTTCTAGCTCCGCCTCAGCGTAGGTATATATACATGTTTCCTTCGTCGCATGCATGCAACAGCATTGCATGACAGTACTCTTGACTAACTTATTACTTAGCTCCTTTTTTTTAATTATTTTCATATTTCTCAAACTGTTAAACACGATTAAATCTTTGTATACAACAGCATTAATAATGCAAGTCAGTAGGCAAGGCAAATCGTTGTACGCTACTTGCAAAGTCATGGGCTCATGGCGTTGTCGCACGAGTACACACAGTACGTACACAGGGCCACTGGGCTCACTGGGTTTGATTGAAGTCTAATAGAGCTCCAAACGTAGCTGCTCAATAGCGGAGAACAGCGGTGACTCCTCTTCAGGTGAAACAAAGGTGACTCCTTCCTCTTCCTTCGGCCCTTTCCTCCGTCGATTTCGGCGGCTCCGGCCGTCGGCGACAGGAGAAGGGGTACAAGTAGTCGGGTTCTTTCTGTAGTAGGGGTTTTTCTTTGTTTTCTCTTTTCTTTTGATGTTTTTTTAGTGCAAGAGTTCGCCGGCGTCACATTTCTGTTATCCCGGTGGCTTTCTATCAATTTCGGCCAAATCCATCCATAGAATGGTCTCCAGATTTACCATGAACGCCCCCAGCTGTAACTAAGGCAGGTTTGCTTCATACAAAGTTGCACCGACTATCCTGTTCATCTCCAATAAGGCTACTACAGGAATGCAAGCCTCCATCATCGATGGTGGATTAATGTAAGAAGCTACGTTTTTATTGTTTTTCATAGAAGCCGTAACCTTCAAATGGAGGCCCCTTTTAGATACGCTTCCTGTTACTTTCATATGTAGTTACTCTTTGTTTATGTGTCACCGTTGCATTCAGCTTGTCACCTGATTGAGAAGAAAGGACTTGATTGCTTTTTCTAGTTTCTTAGAAGTTCTTCCTAGTAAATTTCAAGAGCTTATGCTAATTTGGCTATCTTCAAGAGGTCTGTTTGCAAAACTATTTGTACTTGACTCCCCGTCCCCACGGGGGATAACTCATGTGATATATATATACATAGTTATCTATATTCAACGAGTCAACCACAATCAATATGCCACATCAGCACAGACCACCTCTAAAACCGTCAAAGGAGTCGATTTGCACCGGCTTTAAAAATGTGGGAAACGTTATAACCGGTTTTGCGGTTGAGCGAAACGACCCAATGGGAATCAAGAAGGGAGAGAAAGTAGACTATTCCTTAAACCAGAGTGGTGACCTGGTCGAGGCCAGCCCGTACAGGCCCATAAAGCAGACAAGCCCAAACACCGCACTTGACAATATCTCCGGCTCTCCGCTCCGCTCTATCGAGT

>Os03t0133300-01 2000 bp upstream sequence

CGGAACAAAGATGTGCATAGGTCGTTCCACGCTGTAACGAATAAATGTAAGTTTACTTGCTGTAATTTGGCCCAATAAGGCTACCGGCCTACCAACCGGAGATCGTTAGTCTTGGCCTACTAACTCTTATGGACCTTGCCAATGGGCTAATCTGACCAAGTGCCCATGGATCATAATAAAGTCACGAGGAACCTCTCTTATCAGCCCGCAAAATTTTACCGTTTTCATCCCTTAATATCCTTTAATATAATTAATATCCCTTAATATAAAGGATTTTGATATTTTGCTTGCACTATTTAACCATTCATTGTATTTTAAAAAATTATATAAATATATAAAAAAAAGTTGTGCTTAAAAGTACTTTGATAATAAAAAGTACTTTGATAATAAAGTAAGTCACAAATAAAATAATAATAATTTTAAATTTTTTAATAAGATAAATGGTTAAACAATATAAATAAAATATCAAAATCTCTTATATTAAGATACAGATATGGTAATTTTTAAATGATTTTGAAGTTTTGTTTTCATTATGCTTTATATTTTCACCACTACCTAGCTAAAATAAATCTTACTAAAATAAACGTTTGGATGACACAAAACATTTTAACTGGATAGATGCTGCCACTTTTCCACGACAACAACGTAAGCTGCCATGGCCACGGCAGAGGCCCGCGGAACCACAGCCAAGCGTAGATAGCCTCCAAAATTCTCGACGGTAACACGCGTGTCACCGTGTCAGTGTCTCCACGGTCAATGCTTATCCAGTATATCTATCCAAAGCCGGGGAGGTAGGAGACATGTTCCACGGCGCCGCGCCATCTGCACCGGCCGCGCTGAGCCACGAGTTACCTCAGCACCTGGCCGCGCTTGTGGCGGGAGCCGGCCGTTTCACTCTCGCGTGCGCGTGACGTGGACGATCCGTGGCCGCTAGACACGCGAGTCGCTTTTCCACGTAGCCTTTTGTCGCGACGGTCTCTGGCCTGGTCCTGATCGGGTTCAGTAGAGCCGCCTGGTTGGTCGCCACTTGGGACAGGAGCGCTTTTTGTCCACATGCTGTGACTTTGCGTGGAGCCACGTAAGATAGACCAGGCCATGTGATCCGTGATCAGTGCTATACTATGCTTTAGCGTACGTGATTTATGGAATTTAATTTTATGATATAATATATATGATTTTAAAAACATTATTTTAGTATACCATCTTATTGTCAGAAGTTGAAGGTTTAACCCATCACACATTTTCTTAAGTTCTTTTCTTTTTATTTTTGAGACTTTTTGGTTGTATGCTCACAGGCAGAGATCAGAGATACAATCCATTTCTATTATTTAAAAATTTTTACTTTATTTTCATTTATAATTCTAATGAATTTTAGCATTGTAAAATTTAGTTGAAAAATAGACTAAACAAATTCTTATCATTGTTTTCTTATGAAGTTTTAGAAAATCCCAAAGCTTCCACTGTTAAAACTCTACATAAGACTTTAAACTGAAGTCTGACACTATCAAAATTTAATCCCACCAAAATATAGCAAATGGTACCGCCATTTGTAATACTAAATATTGGTAAGGTTAAAAATTACAACAAAGTAAAAAGCCCAACGTGCGTATATAGGGACCAGTGTCGCGTGATAATAATAATTTTATCTTTGCTAGTGTGCGTGAGACACAAGCAGCACAAGACATGGGACGGGGTCTTCTAGTAGGAGTGCCAAAACAATTTGAGCTCACGTCTTGGGATTCCACTACCGATTTTAAACTGCATTTTCAGATAAAGTGTGTGAATTATCACAAACAAAATAAAATGCTTTTTTTCATTAGTTTCCAGAAGAGTAGGGGAGAGAGGCTGGCCAACTTGTTGGGCCGGGCCGACCGTGCGGCTCCCTGCGTACCGATCACCACAAACTTAGCACACGGTGGCATCCTTGCGGTGGGCCCACATCGGGGGGTCCCCTCCTGATTACTGCCAATCTTGACTCCCACCGACAAGCGGGACCCACC

>Os03t0138900-01 2000 bp upstream sequence

TGCGTCGTGGGAGGTGAGAAGTGAGATCGTTGCCGACTCGACTCCGCGGGCTCGTGGCGTCCCCAATGCGGACACGAAAGACCGCGGCCGAAACGTGCCGAGACCCACGGTTTCTCGATCGAGTTGGCGGCTTGGCGCTAGCTCCGGTCCAAGCCCAACACGCGCGAACGCCGTGGTAGCAGCAGCATACGTGCACGCACGCGCGTACATGCATGTACGTCTTCCACTGGCATATGCATCTGCAGATCTGTAGCCTGCTTGGGGTTCAGTGGCATGGCATCGCTGGTACGTTTTCGCAGCATCAATCATCAAACAGTGCCAAACCGACGAGATGGGCACTGGTAATGTGCTTCTTTGCAATAATCCTCGGTACATATCCTTCTGATCGATCGAGGAGCAGAGCTATCTACTGTTCTACGGATTACGGCCGGGCGTGCGTGTTCACATGGATCGCGACCGGTTTCTAGCTCTAGATACTGGCAACGCATCTACCACGCGATCTCTACCTCTCTAGGTGAGCCGAACACAAATCCGGACCTGCAGTGTACTAGTAGCTGGAGTAGCTACTACCTTCCAATCCCCTCTTTATTCCGCTTGTTTTGCTGTCTGTACTCCATCATCAGACGATCGATCGATCGATCGATCACTGCTGCTTCCATCACCACCGGCTTTGTACATGTTTTTGGGTAGCGACGAGCAGAGACATATCTAGAAAAATATAATAGTAAGGGTTGAATAAACTACATCAGTATAAATCGAACCTCCAATTCTCACATCCTATGGATCCATAGAAAAAAAATTAGTGGAGGCTTAGGGGTCTCCACTGTCCGAGGGTGGTAGTGGGGTCTCAAGACCCCTCCATCTCCATTGTGAATCCGCCCCTGGCGACAAGCCCGTAGGGCCGGAGCAAGTAGCCGCGTCACCGCCGCTAGCTGCCGCGTCCGTGAGATGGGGGGGACGAAGGTGACAAGCGCCCGCAGGAAAGGGAGACCTCCCGCCGATGGATCTACCGTCGCGTCGCATACGTCGGCCGGGCACAAATCGATCGGCCATCGGTTTCGTCAGCGCAACGCGGAGCAGTGAGGGGAAACATCGTCTCGTTCCTTGGTATTCTCCCTCGTGGTGTAAATGTACGTATATCTGCTCTTTCGCTGTACGTCGGCTGACGGAAATGGGGGCACCGAAAGTCCGGAAGATTTGGTGAGGGCTCGTTTTGTTTCTTGAGATCAGTTGGTATTTCTGGAAGTAGTTGAACAGCTTTGCTTTTCCATGTCAAGAAGAAGCTCAGCTGCTCAGTTGGTGCGGCCGTTCATAGAAAGATTCGAGATTCAGGTAGGTTGTATAGTTCACGGGAGCAATCAGCAGTGTGGAAATTTTTATCACGAAACTACCAGACATCAAGAGTCATATTCTGTTTTCCAGGTTGTAAAGAAATGATATTAATTTTTCTGTCGACTTATCAGCATAAGCGCACCAGTGGTCTAGTGGTAGAATAGTACCCTGCCACGGTACAGACCCGGGTTCGATTCCCGGCTGGTGCAATTTTCGTTTTTGTCACCTGAACACATTGATCATTTTGCTGGTTTAAACATCATGGGCTAACTTTCGATCAAACCGATTTACCACGTTTCATTCCTTTTGTGATGTTACCACACTAGGCTTCTAGTACCTGTGAAATTTACTTTGGTTCCATTTTTTTTGTAAAATCTTATCAACAGTTCAACATACTGGCCAAATACGAAATATTCAGTCGTAGCAATCTCATTTGTCTTTAACCCTACGGATATTCAGCGAAATTCCCAAATTTTAGAACTACGGCTTCACCTCGGAATGGGTTTCATGGGCTCTTTCCTTCGGAATGCTCATCCATATGTATGGGCCACAAGTTTAAAGTGAAGCAACCATCAATTTTGGCCCATAGAAGCAAGGCAATATTAGGCTTTGGTACGGCCCATCTTGTGGGCGAGTGGATAATTTCACCCGCCGCGAATGCGAATAAC

>Os03t0146100-03 2000 bp upstream sequence

CTTAAAAGTTCTCACGACATATACTACCCCTTGGATTTCTAGATGAATGTGTTACCCCTGATTTTTTCTTGAGGATAACCATCAAACTTTTGTGGTTAAATATTGAGAAAATAATTCTGACGAAGTCGCTAGAAGCATCACCCTGCACTTATCAATCACGGCAGAGACAAAAAAAGAATAGTGGAGAACACGTTAATCCCTAAACGCTAGCCGTAGAAGCGACGGCCGTAATCAGCAATCAGTCTACCTCGCCGCCCCGGAGCAACGCAGCCAATCCCAGATCACTTCCCGCTGCCGGGACAAAAGAACCCATCAGCCAATCCCGAGGGATTTGTGGTGAGACGTCACGTCGCCCAGCCTCCGAATCCACACGGGCCACGAGGCCTGCCTCCCCCGCACACGTGGCTCGCTGACAGCTCCTCGGCCGGCGTGGCTGAGCTGGCAAGCCGCCCGCCCCCCGGTTGCTGTCGCCTCCCCCCTCACAATCCGAGCTTGATTACCAAGTGACGCTCGTGTGATCGTGTTGTTTAACGGCTTACTCCCTCCAGTTTTATTTTAATTGACGTTTTAGATAATAACACGGTCTATAAAATATGTCTTTGATCTTATTTTTCTATTATAATATATATAACTAGCATGGTGGCCCACGCAGATTGTGCGGCTAGCATCATTATATTTTCTCTCATATAATAGCATATATGTTTTCTCATTATATTATTAAAATATATTACAATGACAACATAATTTTAAATTTTGCAATAACTTTTCAAAACTACTAATGTGTAATAACTACTCTAGTCTACTCTTCTAATATTTCTTATTTTTTAATTCTGAATTTCAGCTATTTCTAAATTGTATTTTTATATAGACTCTGCTTTTTCTTTTTCTCCGATTAATGTGAGAATTTCTAGGCCATGAGAGCGAACATAGAGTCTCTTTTTTCTATTCCTTTAATTACATAATAGATAAATAAAAGCATGTTTACTTTTATTACATTGCATTGAAAGACAAATCTATATATGATGTTCTAATTCCTTTAAACTAAATATTTTTAAAGTTATTGATGGTCAAAATTATAAAAGTTTGATCTCAATTAATATGGAACCGGAGGGAGAGTAATACCAAGCGATGATCGTGTTATCGTGTTGTTTAACGGAGCTCTGCGTGAGACTGACGACAAAATTTGTTACAGATATCCATATCTTGATTGTAAAAATTCAGTAGTGTTTGCTTCAACAACACTTTCTTTTTTCAGTTTTCTATTTTTGTTTTTTCTCTCTCCCCTAGCATAAGCCGGTCTAGCTGATTGCGTCGTGTAAATTAAATTCTTTTCTTCTAATATATTGACGTGTAATCTTTTTGCGCGTTCGTGAAAAAAAAAGCTTCAACAACACTGCAACAGACCATACACGTCCTTAGCATGTTGCAAGGAAAACAAGCACAGATGATGCCGAGGACTGGACAACAAATCGTCGACTTTGATCACTGGTGCTAGCTTAATTTCGAGCTTAGCAGCCTGCCTGAATAAACATTCGGTAGTGCTACTCACGGTTCATCCCCAGTGTTTTATTACGTAGCTCCATTAGCTGTGACACAGAACAGGTCAAGTTTTAATTTTTTTTTTCCATATTTTCATCTGAGAGCTTGTATTCAATTCTATCATATCCTTATCCATCATGTCGTTCATTAATCCCCAAAACCAGCACCTACCAGCTTGCTATACGCACAAGTACCCGGCTAATTTTCCAAAATCACAAACCACACCCTACTGATCTCTAGAAACTTCACCGATAACCGTAATATGGCAAATCCGGAGGGGTGAGCACAGCCTCACACGATCACATTCGTGACTCACTCGAAAGAGAAAGCGAGAGAAACAAGAAAAGAAAAAAAAAATATCCCCATGTGTTGCCAACTCGCCACCGGCCACACCCGGTGTCTCTCGTGCATGCGCTGCGCTGGGTGTATATATACCGGCCTCTCTCCCGTCCCGTCTCCGC

>Os03t0241200-00 2000 bp upstream sequence

TCAAGGGATGGATTGTGCTGCAGTATCTGAAGGAGAGTGGGCAGATACCCTTACTGATATCAGTGTTGGATACTTACTGACAGAAGCTTCCAGAGGTGCTAATACTGATTGTCCAGGGACATCTGTTGTCAAAAACACACTGTTGCTTGAGAATCCCTGCAGCTATGATTCATTTGATGCTGCTGTTGCTCTTCATGCTTCTCGTTATAAATCAGCAGAGCAGCCAGCCCTTGCATCCCATTCAACCATCTGGGGTGCTGAAGAAACCTGTGACGCATTCAGTTTTAACCTACCAGCATCTAGGAAGCGAGAAGGTTCAAATAATTCAGCTAGCAGCTCTCCCGATAGTGACAGTGATGTTCATCCTTCAAACTCAGAAGGGTTTCAATGTTTTCTTCAGGTTTGTTCCATTTATTGATCCAACCTAGAATAATAAACATTTTTTTATACATAGTATTGTTAATTTATGACTTATTTGGTGTTGCAGGACTTAGCTGGAGCGGCGGTTGCTCATAATCCTTGTATTGACGATGCCAAAGATGTAGAGTCACTTTGTGCTGAATCGCCACCTCGAAGTGACCACGATTCTGCTCCGAAGGATCAATCTCTAGCTGACTTATACTGGGTACTATACAAATGTCCTATCCATACCTGTTTTCTTTTCATGGTGTAATGCTCATAACTAAACAATCTTCTCTCCTGACAGCCTGATTCACTTGGACCATTGGACTTGGACATACCGTCCGCGACATACCATGCTGATGACTTACTCCTAGGGGACAGCCAGAATAGTTGGAACCGCATGATGGCGAATAGCCTGGATGCATTTCGAAACCTGTCGTTCTTCACGGCAGATAAGAATGACTCGATACCATCCATCATGTAGAGCTCTGAATACTGGTGCTTCCTTCATGCGCTTGCGTGGTGAAATTGCTACGTTGGCTACGAAGGTTTACCGCCAACGATGCTGTGCATCTCGCTGAAGAGGCTTTATATTGTCCAAGTGAGCTGTTTGTGGCTAGATCCAACAGCTGCGGACTTGCTGTGCGTTTGCAGTCAAGAGGGACGCATGTTGTTGTAAAGTAGTAACATAAGAGCTGTTCGTGCTAACATGTATGTCGTGTAAATTGTGGTGTTCCTGTAAAAAAAAAAAGCTCACACAATCGCTTGGTTACAATAAAGCTTCTCCTGCCGTGTACCAGATCAATTGCCCAATGCTTCATGGCCGCAATAGTTGTGTTATCTATTGCCATTGCATAGCATATTTCATATTTGTCCAATGCCAATTTTTCATATATAAAGTTCATCATCACTTAGTGGAGTTAATGTTAATCCTCTCTTCTCTCATGAAATCATATCATCTGAATTGTTTTTAGCTTTTGGATGATTCATTAAGGGTGTAAATTGCATCTATTCTCGACTAAAAACACCATTCAACCTAATCATAACATTTGGATAATTGATAAAGATACAAGAATATATAGACACGTGAAAGAGTATATAAACACATGAAAAAAAGTTATATAGTAGGTAAGTAAGAACATTTTAAAACTCATACCTACCATATTATCACATAATTCTCCCATAGCAACAACAGGATACCATCTAGCTTTATATAATTTTCACTCCCCACGAAAATCCCGCAAATTCCTTCATTTGAGAGGAAGTGGAATTTTTTTCGAAATTTTTTTATGGAAATTAAATTGTGAAATATGCTCATGGCTTCTTTGGAATACACGAATTTTCTTTGTTTATTAACATTCTTTTTGTTAGAAACAAGTTTAGCAGAAATTAGTTAAATTTCCATGAATTTTCTCAAAAAAAAAACTTAGTAGTAATATATTCCAAAGAGGCCTTATGATTCGAGGGGCCCTCAAGTGCAAGCCCATCTCCGAGCCTAGCCGCCAGGGGGGGGGGGGGGGGGGGTGAAAACGAAATGCACAGAAAAGGGGAAAAAAGAGGAAGAATCTCGAAGGCAATCGCAGCCGTCCAATCCACCC

>Os03t0281600-01 2000 bp upstream sequence

GGCGTGTTTGAATCCTAGTCACCAGTCCTACCACCATGCCGCTAACGAATGCCAGATGTCCACTGGCCAGCAACACACTATATATCTCCATGACAGGATCAAGGTCTGTCCTACACGATGCCCCGTCAGTCGGCAACCTCTTGGATAACATGGGATTCATGAGCCTCCCTATGAGGTCATTAACCACTCCTTTTTTTTTAAAAAAAATCCACTATCCTCACATGATTTATACCTTTCTCCTTTATATCTTGTACCACTTCCTTTATTAGTAATAGATAAATAATATTTTAAAATGTGTGTAGATAATATGTTTTACTTAACCATGAATATCACTTTGAATCTTTTTCATTAAACTCATAGAAACGACAAGGATAGATTTGTCTTTGAAAATAGTTCTAAAGTATCTTAACTTTTCTATATTTTTTTTCACCGTATCATTATCTAAAAATGTCACTATTTGCCACCATTATCAAACCTTACTGAGGCAGCGTTCGTTCTGGGAGATTGGAGATAGAATGCACATCGTTTTCCGCGCGTACGCTTCCCAAACTATTAAACGGTGCGTTTTTTAAAAAATTTTCTATAGGAAAGTTGCTTTAAAAAATTATATTAGTACATTTTTAAAATTTAAAATAATTAATACTCAAGTAATCATGCACTAATAATTTACATCGTTTTGCGTATCTTCCCAATCTTATTTATCTCCCCTTTCTCAAACACAGCCACATGATCCGTCCTCCTGACCTCCTGCACGTTCAAAACCGCACTAAACTTTAAAACAGTTCAGACATGGCAAGGTACAGAAGCACGCAATAACCATCGTCACGGGGACCCGCGCGTTGACGACGTGTGCGATCCAAGGCCGTCGCATGTGCGGTTCCGCCTGTCTCCCACCAACGCAAAAAAAGCACGGCCCCCCGCAGGCGGCAGCATCTCCCCCCTTCCCTCCGTACCGTGCCAGGAAATCAGGAAACCCAAGGATCCGCGCAGTACGCTTCCGTCCCATTCTCCACTCTCCGTTTCCGCTCACCCCCCGCCTCGAGCACGACGAGCCGAATATTTCCGCGTGCCATCGCCCAGGGAAAACAACGTTGTGCTCTGCTGTGCGCGCCTGCGTGCTGAGCTCCGTACCACGCCGCAGGCTCGATCTGGTTGCTTGATTCCCCAAGCGTGCGCGACCGTGCGACCATCCGACGCGCTGCTGCACCCGACACGTTGAGTGTCCGTGTCCCCTGGAGCACTGGACGCCCGGCGGATCGGCTATTCCAGAACTTCTCTGGCAGACGGCTCTGTATTTTTCTTTTTTATAAAAATTACACGTGGCAGGATGCTATTGGCTGGAGTGGCATCTCCAGGATTTTTCCTTGTGATCGCGGCGTGGCGGCTAATGGTTGGCCAGACTGACGTCGCGCCCCGGCAGCAGATTCCTTGCGTCTCCCGATCCGCGCAAAGCGGCGGGGGAGCGACGAAAGTAGCCACTCGAGTGGCACTGCGAGTGGGGCCGGAAGCATGTGGCCCCCACGTCTCGGTCTCTCTCCCCGGTCATGGAGAGCCTTTTTTCTCGCTTTGTCGCGGGCCAAATGGGCCTCTGAAAGGCCCAGATCCCAAGGGGCACACGTGGCAGCACGGGGCGTCATCTGGTGACGTCGCAACCTCCCCCGGGTCAAGCAACGCATCTGGTTAAAAAAAACAACTGAAAAACTCCGTTGCTTACCGGTTAATTGACCCCGCCCGTTCTATCCCATCGTCGCATCGAGCCAACGTAGCCGCCACGTTGACGAGACCTACCTCTCACTGACACCTCTACCCCACCCTACTGTGTCCCCACCTGTAAGTGTACAAACTCCGTTGAGATAAAGAGATAAAACCAGATAAAACCAGCAGTATTTAAGCGGCCATTGAACTACAGTGCCAATTATATTTTCTTCTTCGTCACGATCGATGGGATTTTTATTTACCCGAGCGAGAATAATAAAGTAGTTTACTCCACGAAAGAGAGG

>Os03t0291800-01 2000 bp upstream sequence

ATCGCCTAGGTGGACATCCACCTATTTGTTGCATGTCAACTAAATAGGTATGAAAAAAAATCAAAAAAATTCCAGAAAAATAGTAGTAACTGTATGCTAGTAAGAGGATTGGTTCCCATAGGTGTAACCAAACAGCAAAGATACCACCAGACTGATCGGTGCTTGTGAGCGAGCTCAAGAAGAACTGGACATCTGAACATTCAACTGATTGCTTCCAGCTATGGAATAACCTGAAGAAGGAAAATGGACTGTGTTATACATACATTGCTGTTCTCTCTCTTTTTTTTTAACAGGGAATTGAATTTCTCTGTCTCTCAGAAAATTATCTGTTAAACCTGTAGTTGTTCTCGAAAATAAAAACTGTAGACCTCAATGTAACAAGATGTTACCCATGGAAGATGGATGAGAGCATTTCTCCCGATGAACAACAAAATAATAGAGTGGAAAGTTCCTGATCACGACTTCACGAGTTACAGCAAACTAAACAAGAGCTGGGTCCAAGATGTTTCAGTTTCTTCCGATTTCTCGGATCCATGTCTCCACTCTCCACACATTGTTAATGTTCTCAACTTAACAGAAGAATCATTTTTACAGCAAGTGATCAATGAAAAATAATAGTATCTGACAATGATATTCTTTTGAAAAAAGAAAATATTCGACCCTACATAGGCACATCATTTGAGATCTGGAAGTTGAAAGAAAATGATAACAGACAGTGATATCAATTCGTATGCGTACTAGCTAAGACGAGTTGGCGTGACAAGATTGGAAACATATTGAACACCCGCAGAAAAGCACTGTGTAAAAACTGAAACGTGTTATACTCTACAGAAACAGGACAACCGACTCTGAAAGATGCACCGATTCTCTGATTTTTTTGCAAGGCTATTCTGTGACCTGAAATTTCAGTCACCTATGCTGTACACGGTGCTTTCATTTTCCCCCTGTAATATCCATTTTCTGCTCGAAACATTGGTCCTATTAAATCAACCTCTGACCATGACTAACATCACTTCCTGAACAATGATTTTCGTAGACCAATCCCCCCGAACACTGTCGATCGGAACAATTCGACATTCTGCAGTTTCCTCCTGCTGCAACACTATCTGAAAATCGGAACGATTCAGGCACCGTATCGTTGTCTGATTCGTCGATTCGTTCCATCATTAGTGTGTTCACAACGCAAAGACGATGACTGTAGTAGTATCTGCGTGCTGATTACATCCAAATACAGAATGATCATCTCCGAGAATGCGAAAGGGGGGCAATTGACGTAGCCGCCCCCGCCGTTGTTTGCTTCCCCATCTCGCACTCTTCCGAGACAGCCACGGCCTACCCACTCACATCGCATAAACAAAAAACGAGTACTTACAACCATACGAGTATACACCATAATACCATATACTCCACCCCTGTATCCCGATAATGAAAACGAAATGTATGATATAGTAGGCATATAATTAAAACATGCAACGAACAAATAGAATACTAGTAGTAGAATACACACACGCACATTGCCTGTGTTGTTACATGTGTACAACAGCTTAACCTCCAAACCCCCCATGGAAGCTTTGCAGCCTTGCATGTTACTCATTTGACCCACCCCTCAGTAGTAGTACTACCCTCTATCTCTCTATATACAAAATGAAAGGGAATGAGATTCAATTAAGCAGCTAGAACAGGGGGGAGTTTACAAGTGTTGGTGGAGGGGAAGAGATCTTGCAACCATAAAAGAAGAAAGCAGGCGAACCAAACCAAACATACCAAAGGGAAGCAAAGAAAAGGGCAAACATCCAGCAAAAGTCACCAAACTTGTAAAGGGATCGACACATATATAGGAGAGCGAGGTGTCCAGTCGAGAAGGACAGCCAGGCCAAGGAGCTCCTAGCCTCCTAGCCTACACAGAGCTAAGCAAAGAGGCAAAGAGCTCAGCAAGAAGCTCCATTAATCCATTCCATATACTCATATAGAGAGGAGGAGGAGGAGGAAGAGGAAGAGAG

>Os03t0326500-01 2000 bp upstream sequence

AAAGGCCAGCGAGATCCGGCTTTTCTTGGGTTAATTGAATCCATATTTCTACACTCGTCATTTTAGATAAATGACACTAAATTGGTAGGCCGAATTTAATTATTTAAATCGTAAGCATATGCCAAGGAGCGCATTCTTTTTAGCAGGACAACAAAATCAGAAAATGAAAGATAATCTTACAGATAATCAACGCGTGGTTGTGCTTAAAATAGACACCTAATAATCATTCATGGCCATATTATTAGTTGCCATCGGCGGATACTTTCATTCTTTATCCGAATCAGTTACTATGAATAGGAGTTACCGTCCTGCGTTGAAACTTCACCACATAATATCATCTCTATCTAAATACTACTAGTTCACTATTAATAGTAGTGGCCTGGTTGGTAGTCCACTCTCAAGTAAATAGAGATAATTAACATGTGTGGTGCCCCAAAGGAAGATAATTAACAGTTGAAAAAGTTAAAAACAACCCATATGATTCCAACTTGTTTGCCATTTGTTGCAAAATTTCTTAACATAGTTTGATCACAGCTAGAGATAAAACAGCTTAAGAAACTAAACAAGAAAAATTATACAGTAACAATTGTTAACATTAAAAAAAAAACTTCCGATAGATGAAATCAGGGATGTAAATAGGTGTCACCAAAACAAAACCGGCCGGGGTCATGGGTCGCCGGTTTATACAGTTACCTCTCCACTTCGCTGTGCGCTGGGCCCCACCTCATCCTTGACCAAGAAACCGCGTCTCGCTACGTGCCCCCCGCGAGCTGCACCAAGCTCACCCTCCCACACGTGGCACCGCGCCTTCCTGACCCTACGGGGCATGTGAGCGGTGAGCCCCAATTTCTTTTTTTTTTCCTCTCTTATCCGGAATATATACATTCCTCGAAATAAAAACCACCGGAAAAACAGAGAAGCGCCAGAAGCTTCCGCCTTACGTGGCGTCCATCCGTCCGTTGATGGGTCGAACGGACGGCTGAGATCGAGGGATGGGAAGTGGCGGTTTTCGTTTTTATCCGTGGGCCTCCTTTTTAAAAGGAGCAGCTTTCCTTCGCCGCTTCTCCAATCCTCTTCTTCTCCTTTCCCCCTCTTCTCGTGCTCATCTTCCTCTCCTCGCGTGCTTCCTCCTCCTCCTTCCTCGGCGAGCGTGTTGGTTGATCCGTGAGAGCGGCGATGGCGGCGACGGCGGCGCAGGCGGTGGCGGTGAAGGGGAGCGTGGCGGTGCCGCCGTGCGGGAGCCGCGGCCGGCGGAGGGGCGCCGTGGCGTCGGTGCGCATGGCGGCGGCGGCGGCGACGTCGGCGTTGCGGATCGGCAGGAGGAGCCCCTTCCTCGGCCGGAGGCTGGCGGTTGGGCCGAGGAGATCCAGGCCCGTGCCCCGGAATCTCGTCGCGCCGGTGCAGGTGATTTGACTAAAAAAAAATTTTCTTCCATTTCATCCTGTTTTAGTTTTTTTTCTTTGTAATTTGTTAAGTATCTTTTATTTCACATAATAAATCATCCCTCACCATAAATTTACTTTCTACAAATTTCTTCATACTTTATTTACTTTACATAGATAGTTATTTTTACAACATTTTTTTTTCGGAGCGTGCTAATATGATTTGGTTGCTTTGTACGCGGCAGATGAATCTCGCGTTTGCGAAAGCCACGAAGTGGTGGGAGAAGGGATTGCAGCCCAACATGCGGGAGGTCGAGTCCGCGCAAGACCTCGTCGACTCCTTGACCAACGCCGGCGACAATCTCGTCATCGTCGACTTCTTCTCCCCTGGCTGCGGCGGCTGCCGTGCCCTCCACCCCAAGGTCAAAATCAGAGCCGATGAAGTAGTTCAATTGCAGCTTACCCTTCATGAGATTTCGGCAGTTCTGAAAATTTTTTACATGTATTTCTTTGCGCAGATTTGCCAGATTGCAGAGCAGAATCCGGACGTGCTGTTCTTGCAGGTGAACTATGAGGAGCACAAGTCTATGTGCTACAGCCTCCATGTTCATGTTCTTC

>Os03t0337500-00 2000 bp upstream sequence

TTCTCCATGAATAGAAATCCCCAACATTCACAAATAGCATGAAAAAAAATTAAGTGCAAGAAACCGACTTGCCTGTACCTTGATCTGGACAGCATGTGCCTCACTCCACGCATCAATCAAACCAAATGAGACAAGTCTTCAGTAGGTGGGTTGGAGTGGACTGCTACCTCATGCGTGACCTGGTTGTATCATTGACGACCGGCTGAAGAGACGGGATATCCAATGGAGATCACTGCCAACACAAACTACTTCAGTTCATTAAGTCCTTCCATAAGCATGGAAAATATGATCCAAAATGGTGGCAGAACATGCAAACTGGAATTGCGGGAGCCTCAATGGATGACCATATGTTCAAAAGTTTTGCTTAAATTGACGAAGTTCAGTAGAAATAGTGTTTTGTACCTTCAGAATTACACTACTACAAATGCAAAGGTTAAGTAGAACACATCAAGAACCATTGAATTGTTAATTTGAAAGCAGTAAACAATCAACCTGAAATTTGGTTAGTATGACATCAATACAAATAGTCAAATTGTAAACAAGAGATGATGCTAGTGCTAGTTAAACATTTCATCAAACACATTGCAAGCATGAATATGGAACATCAACCTCAACTGTTGTTGGTGTCCTTATCTGCTAGCATCGATGATGTCCAGCGGCCCTGGTCCTGTGCCCTTGTTCCAGCGCATCCCTGATAACAACAACAACGAAAAATCATATAGGATGTTGACGATGGCAGCAGTGGTGGCGCGGGTGCGGTCCTAGGGGTCCTCGGCTCTGCCTCCGGTCGGCACCCTCTCATGCGACCGCGCCAGCGGACTGCTCCTCCCTGGAACGTCTGTTGACGCTCGCCTCTTGCCCAGTCAACCTCGCCGACGCTGACGTCCTCCTGGCTTCATCTCTCCACCGCCCACGGGCCATGCGCAGCGACCTCTAGCCCTCCTCCAATGGCCCCCTCTCCTCCTCCTAGCAAGAATCTCCGCCACCTCATATCCCCTTTCCGGCGGCGACGCCTGGATGTCCGGCCTGCGCACGCGAGGAGGCGGCGGGGTAGGGCGAGCAGACCCCGCCTCTCTTTCCTCTCCATCACGGCGCGGCGCGAGCAGGGAAGCAGGCAGCGGTGGGTAGGGTGAGGCGGCTCCGGCGGGCGAGCACGTGAAGGAGGCGAGCAGCTCCGGCGGGAGGCGGCGGCAGCGGGCTGGCGCGCGCTAGGAGGAGCTGAGCGGCTCCGGCGGGAGGAGGCGGCGGCGGGCTGGCGCACAAAAGGGAGCGTTCCTTTTTTTTTTTTTTTTTTGCTTTGACCGATCCTAACTGCTGTTTAGACGTGGGAGACTAATTGTTTTCTTTTATTTTTGCGCTAACTGCTGCGTACTTGAGATGGGGATCGCTAATTGAGGGGTAAATCCAACTCTATGGACCGTTGGATGAACAAATATAAGGATAAAAAAACATTTGAGTTCGATGAACTGATGTTTTTTATATTAGTATAGGTTCGTCATGTATGAAATGGATTAGCTAAAAAAAACTATTAACGGTTTTTCTTTTAAAACTTTCAATTTTGTGGATCGTTGCATGACATTGAGATTCGTGCAAGGGAGTCATCTATTGTGACATGCACCGATGGGGTTCTAAGCCGCAACCTTGGGAACAAGAGTGCATGGGGGTACGAATTAGGCAATTACATATAAGTTATTATGATCTAATTATACTTCAGCTAGTTACGTTTGGAAGTTTTTACAAAACTGTCAAATGTCTTATAGCAACTCCCTATATGAAAGCCGGAAATGAAAAAGTACGACTAACCCCCTCCCCCCGAACACATTAGTATTCTTCACCCCCAACTATACAAACTGGACGAATTACCCCCCTCGACCCAATCTGCGGTGGTTTAGGTTTACGTGGCGTATTCATGGCAGTCCAGTCAACATTCTATTTATAAAAAATTTGTGGGACCCACTTGTCATACTCCTCTTCACTCTTCCTCTCTCTCCTCCTCTATC

>Os03t0454300-01 2000 bp upstream sequence

TACCCCTACATTGAATGGAAGTTAGTGGGACCGGGTATATGGCAGTAAAAAAAATAGAGAAATAAATGAAATATTTTTTTTCGAATTTGTGTTTTACTTTTTTACCCCTGGCACTAGTCTAACAAATTCCGTCGGCGGCGGCAAGCGGCGGCCAGGCGGCTGCGAGAGGGACAGGCGGCGGCAGCGATGACCAGGTGGCGAGGGGTGGCCAAGCGGCTGCGAGCGGCGGCCAGGCGGCGGGGAGAGGGACAGGCGGCGGCGGTGGCCAGGCGACGATGGAAGGCTAGGGCGCAGCTCCTCCCGTCGGCGGTGGCAAGCGACGGCCAGGCGGTTGCGAGCGGTGGTGGCGACCAGGCGGCGGCGGCGGCGGCGGCGGCTAGGGGGCAACTCCTCCCATCGGCAGCGGCGAGCGGCGGCCAGACGGCGGGGAGAGGGTAGGCAGCAGCGGTGGGGGCACGGGAGAAACGAAGAGGAGACATCGTCACCGTCGTCGTCGTCGCCACCACCAAGCTCCTTGCCGGCGGCCACCACCTCGTGTCCTCGCCCCTCTTGAGATTGTACCGATGGGAGAGGGGGTATAAATGTCCTATTTAACATCGTTAACTTCTCCATCCAAAACAGGGGCAAAAGCCTCTTTCGGTTCAAAAAAAGTGGGGGCAAAAACAAAAGCCCTAAAAAGTAGAGGCAAAATCAATATTGGAGATGAAAGTGGGGGCAAAAACGAAATTGTCCCTTTTTTATTTATAATCCATTAATTAATAAGGGTGAGGCTATTCTATACCCCTCCCCTTCAACAAGGCCCAACCCACCTCCCCTTCTTTCATTGGGCTCATAACCCACCTCCCCTCTTCTCCTGAAGGGTCTAACCCACCTCCCCCTTCTTTCATTGGGCTCATAACCCCCCTCCCCCTGAAGGGCCCAACCCACCTCCCCCTTCTTTCATGACTTATAATCCACCTTCCACCCAAATCAAAGCATGGTCAAAGGACCTCAAATCACTGCCACGTCAGTTAAACCCGGATGCTAACTTCCTTCCACGTGCACCTATTTGATGTCCTCGTCTCGTATAAACAGAGCAGTAATAGGATGACCACCATGCAGTAACGTTGTAAGTTACTTGCAGTAACACTGATGAAATATAGTTAAAATATATTTATGACGGATCTAGTTTTGTAGAGCTCATTTTTTAAAGCATTACGGTGTAAACGGTTCAAAAATATAATATGGTGTGAGAGATATCATTGTTTAAAGATGAGAATTCAAAAAGATATCACTTGCACCTACCTTTACTAGTTCAAGGTGGGTGGTAGGTCTTGTATCTGTGTTCGATTGTTACCACATGTTATTGTAGTAACACTATTAAAAAATTGCAGTAACAAGCAATGTTACTGTAAAAATTATAAATTATAGTTGTTATTACACACATACGGTAACGTCATGATGGAATGGTAGTAACTCACAATGAAAAGTGGTAACAATAATGGTTTAGTATTACTACTTATGGAGAGAGAAATTTTAAAAGACCAATCTTCGAGAAGAAATGTTAGTAACACATTTGGTTACTGTGAAAACACCTAAACTGTTATTACTCCACAGGACACACAAAGTTACATAGTTGTAGGAAGCTAAGTACATGTGTTATTATCTTTTCTCTTTTTTCTAGAAAACTAAAAAATAAATTGGTAAATTCAAGACATGAACATCGCTAAGTACATGTGTTATTATCTTTTCTCTCTTTTTTAGAAAACTAAAAAATAAATTGGTAAATTCAAGACATCTATATCTATACTTCTATACTAATATAAAAAGGAGAAGTTGCACCCTTCCAATGCTACAGGCCAAATCATCTGTATCAATCCGTACCGTGCATCCTCAAATCATGTAAATCTCCACCGTACTATTTCACCACGTCACCACGCGAATACACAGCCTCTCCACGTGTGCGATGGACTGCACTGGATCTCAATTCACGTGGGACGGTGCGAGGACTACGCCTCTCC

>Os03t0729300-01 2000 bp upstream sequence

GGAGTTACTGATGCCGTAGCATTGCCAGTTATAAGAGCGATTTGATGGTCAGCTTATTACAGCTTGTACAAAAGGGGAATGGTCGGTGCTAGGGAGATCTCTGCCGTTGGCTTCCAATCTGACGGCCATGCACGTACGCAACGCATGAGATGATGAGAGAGACGAATGGAGTAGGAGTATATTGTGCAGGTTTGGAACTTGCCTTTCCCTTTCACATATATAACCTGGAGGGAGCCCTAGGATCCTCAAAGGCAGATTTATAAAAATATTTTGTGTTAGTGGTAGTGTAGCACGGTTACCGTGGCTAAGCAAAATTAGCCATAGACCGATGGGGTAGGGAGACATCTTCCTAGGTAACACCATCACGCAAAACATCTCTCAATGTACCTTCGAGCCTCTCCACGCACGCAATATGGACTAGTTTACGCCATCGCCTACCTTGAGAACGCAATACTAAATTAACCCTAACTAATTAGTACTCCCTCCGTTTTAAAATATTTGACACCGTTGACTTTTTAAGTACGTGTATGATCATTCGTCTTATTCAAAAAATTTAAGTAATTATTTATTCTTTTCATATCATTTGATTCATTGTTAAATATACTTTCATGTACATATATAGTTTTACATATTTTATAAATTTTTTTAATAAGTCGAACGGTCAAACATGTGCTAAAAAGTCAACGGTGTCAAACATTTTGAAACGGAGAGAGTAGCCCCTAATAACAATGTGGTGGGCAAAAGGGCATTAACCGACAAGCTTCCAGCACTCGCAACTCGCAAGTTCCAGTGTTTTTTTTTTTTTTTTTGAAGCAACGTGCTATCGCGTCAACGAATCCGGCCGTTTCGGCGTTAGCGTGGACGTGGGCTATTGGCCCGGTTTCACGTGAGAAAAAAAAAGGCCGATTACGTGCGGTTTTCATGGGGAATAAGTTCATCTGATGTCCCTGAACTTGTCTACGAATTCGATTTTCTTTCTTCAACCGAAAAACCATATACAGCGGGTCCCTCAACAGTCAAAACCATTAAAAAATAAGTCCCAAGATGATTTGGACGACGGTTTCAGCTGACGTGACGCCTACATATCTAATTTGACTATGTCTTCATATAACGTGGCGCTTATGTGAAATTTGATTTGGAAAAAAATAAAAATTGTGGGTCCACATATCAACTTCATCGAATAATTAAGGAGCTGTTCAGATTACTGCTATTTTCAACCATTCCATTCTTTTTGTGAAGTTGTCAAAAAATGTCTACGTTTAGTTTGTTGCCAAAATTGGTAAATACAAAAAAAATTCTACCAAAATTTTAGCAATATTGCCATCTTGCCAAAACTTGTTAAGGTTTATTTTAGATATAATCTGAACATGCCCTAAATTAAAAAATGTAACTCTCACCCCCATCTTCTTCCTTCTCTTTCCCCATCTCCCCTCTTCATCTCTCTCCCCTCTCCTCTCTTTAGGAGGCTGCGACACCCTCGTCTCCCTTGTTATCCCCGGGCGAGGAAGGAGAGAGAGAAAGGAGAGAGAAGATGTGATGAGTGACGATGACATATTGGGCCACATAGGCCTACCATTTTTTATTATTTCGTGTGTGTAACTGACATGTGGGTCTCATAGTGTTTGTTATTTTTTTTGGATCGAACTACCACATAAGCGCCACGTTAATGCCTCGTAGAAAAAAATACCTAGTCAAAGGAGCCACGTAGATGTCACATTAGCTAAAACTGCCAAGGGATCTCTTTTGCACGGTTTTGTAAATTGGGGGTGCGTTGTATCCGGTGTTGCGGTGCAGGGACCTAAAGTGAACTTATTCCTTTTCACGGAGAAATCGACTGAAAACTCACCGCCAGCCCAACGAACGGCCCAGTTACTCAGTTAGACGAAAACCCACGCGTCCGCTGCCACTTGACGCCGCGCCACGCGCTCAAGGCCACACGTTGGCACCGGCGGCTGCACCTCGCCCTCGATCAGTACGCGTCTCAGAGTTTGGCCTCGTCG

>Os03t0744675-00 2000 bp upstream sequence

AGCCTAACCGGTTTCGGTTTAGTTTAAGTTTAATTTGAAAATTTAGCAGCGCCTTTGTCCCTACGCCTGCTACGTCATCGGGGCGGGGATCCGCATCCGCATCCGCTGCGCGGCGCAGAGCAGCGAACGCTCGCGCACCGACCGTCGGATAGCCGCCCGCGTGTGCATCGAGATTCGGGCTGAGGCGCTGGTTCGCGGTGGATCAGGATTTGGTGGGGCCCGGCCGGCCGAGGCGCGGGGGCGTGGGGCCCACCTGTCCGTGTCCGGAGGCGCGAGGCCCGCACGTCTGCCCGGGAGCTGCCACGTAGGAGAGAGAAAGTCTGACGTGGCTCGGGCCTCATTTGCGGGGAAGCCCTCGTCAGTCGTCAAAATTGCTCGTTGTTTGTAAGCAGACACGAAGGTTAGTACTAGGAGCCAAAGATTAATAAAGAATACATTTATTAGCATTCGAAGATGACAGCAAGATTTTTAATAATTTGGTTAATTCAAAGAATCTGCTGGAGTTGTCCAAATCCGGATAATAAATTTGACCCTATGTATTGTACATCTCCTTAGTTAATCTTCATCTAATTCAGGCTCGAATGGGACCATTGTTGCTTTAAAACTTGTTCGGTATCTCTTAGGCAGTGGTTTTTGTTTTACCTAAACTACACAATTTTATCCTTTGCTTCCTCTTACGATAAATATGATTCTTTGCTAGTATATATTCAGTATGATAGCATTTCGATAACTTTCTTTATGAATGAAATTCCAACGTATATACATTTTATAAAAGCCTTTCGATCCCTCTTATAGGGACAAAGATCTAACCATTTGATTACAAATCTAACGCCTAATGATGCTCTCCCGTCGTATTTACTCTAAAAACGTCATACCCCATCTATGTTTATTCGGTTATAGCATCGTCTTATCATTTCTTCAACCTCAACATATCCCCATCTCCTCCTACCTTGTAATGCTACTCAGATCTACGTGTTTAGATCTGCTCATTGATTTTATCCACATCTCCTTCTCTTCTCTTGGTATTTGTATCAGCCGCCACCACTAGTATAATCTTGCTTCACTCATATTCTCATGTGCCAAAAACAACTTTAGGGTTTCGCCTCGAAGCAAATAGAGTCATCAAAAGCTTACTTCATCAATATCCATCGGTGACGAATATAGATGAGTAGGAGTTGATTATGTCATCGGATTCTCTAACACTCTTGTTTTATCCCTACATCCATCGAGGGCGCTTCTCTTGATTGGCCAAATGGTATGTGTTTGGTGTTTTTGCCAAATAAAATATTATACATTTTCTCTATTAAGTTATATTCCTCACTCTAAATTTATTATTATTCCTTTAACTGAATATTCACCGAACATATATATAAAGTATCATACTATTTATATTCTCTCTCTGTAACATGTTAATTTCCTGATAAGTCTTACATTAAAAAGTACTCCCTCTGTCTTATAAAAAACCAACCTAGTAATAAATATAACACATCCTATTACAGAATCTGGATATACATCTGTCCAAATTCATTATACTAGAATATGTCATATCCAATCCTAGATTTTTTTTTGGACGGATGGAATAGGCACCATGCTCTCTAGAATTTTATTTTTTAAAATCTATTTCAAATACACACATCAAGCTTGCCACTTTCACAAAGGAAAACAAAAGATAGGTGAGTCGGAACAGGCAAAACATTTTTCTCCCAATTACGGTAGTGCGAAGACGTCAAGCAAAAGTCACAAAAGATTTTAAAGTAAAACGCACACAAAGTCAGACGAGGTTAGTAAAGCCTAGTTAACGATGTTACCCACAAGGCCATTTTCTTTTCACAATAAAAGAAAAAAACACAAAAATGGACAAGGAAGCTAAGCAACCAAATCACCCACCAAATCGCACAGTACACACACAAATTACAAACCAAACAGAGAGAGAAAGACAGAGGGGAAGGAAGAGCGCGTCGTCGAGGAAGACGAGATAGCGAGGAGGCGCGTGGGAGAAA

>Os03t0745000-01 2000 bp upstream sequence

GCGGGCAATTCACTTTATACACTCCAACACTCCCCCTCACGCGAGACCCCCTCAAGTCTCAAGCGTGGAATATTGGAGTGGGCTACAATTATTTTATTTAATTGCGCGCCAGCCAGGATTCGAAATTATTTTATTTAATTGCGCGCCAGCCAGGATTCGAACTCGAGACATTTGGCTCTGATACCATATTAAGTTGCATGCACCAGCCAGTTGCACCTAAAAGCCTAAGCTGATAAGGAAAGGCGGGCAATTCACTTTATACACTCCAACCGTCTTCCCCAATTGCGGTATGGGAGCCTCGCCCATTCTAATTATTTGATGTTAGGTCATTCCCTAATATTCATATCTTTTTTCAATTGCGGTATGAGGGGTCGCTCGACTGGTTGCTTGTTAGGGCAAGTATTACAAGCATCCAAGTAAAATCTCCTAAATGTCACATAAGGTTAAGTAGTGAGGTGGAAGAGAAAAGAGATGAGAGAGATATGAAGTCACCCCTCATACAAGAGGCAACCCCTACATAAAATTCAAGACAAAAGAAGAGAGATGAAGAGTAGATTGGGCAAATAAACATTAGAGCTAGTGTATTAGAATTAGTATATATGTGATATAATGTACATATTATCTATATTTATTAGTTGATGATATGATTAAGATTAGATGTAGCACTATCTCTTTTATAAAACTTGCCCTTAGTGATCTCGGTAGGTGCTCCTCTAGTGTCCAGGTCCATGCTCAGGGAAAGTTCTCACGCTTCGTTTCATGCGGCTTGTCGTGAACACGGCCTGTGGCAACGAGTTCATATCTTCCAACCAAGTCCACCGGCAGGCGGCAGCGGTGCAGAGGCGATGAGCTTCACACGGCACCTCATTGGCAAACTCATGCATCCCATGATGTGGAGCGGTAGCAGACTTGCCGAGGACTATAGCCGCCTCCACCATCAACTCCTTCAAATGACAATGCCGCCCGACGGTGGTCGTGAGGCCATCCACCTCCGCGAATCTTCGCTGTCACTACTGCTCAGTCTTGGCCGCTGCTTGGCTTCCTCGAACCAATTAAGAGGTTTCACTCACACGTGAGGCCACGGTATTTTTCTATCTTTTTCCTTAATATAATACCACCTCAACCTACCATGTCAGTAGAAACCACTTCCTAAACCACTAACACAATCAATTTACATCGGTTTTTAAAGTGGAGAAAAACTTTATACCGTTAAAGACCATGATAAGATGAGAGGGGGTGTAGTAGACTTATTCCATCCACCTGTCTTTTCCAAAGGAACGTGTCACGTAATCGCGTGGGTACCTTGTGCCCCCCGAGGGTGAGAAAAAAGAAAGACCGTCCCCTGGATGACGTGGCAAACGCTCCAGGCCTCGGCGGCTGCATTGCGTGGCGAGCGCTGCAGCGGCCCAACACAGCGAGCGTCGTGGAACCCTAAAAGGCCTGCAAAGTGTACACAAGAGACGGGCCGTACAACTAAAAATTTAGCCTTCAACCGCGGCAGTATTCGCCGGCCTTCTTCGCCACATTCGGCCCATCTCCCCCAGTTCTCCTCTCCGATTTCTCGCCGCCCTTCACGTGGCTCACCCCCCTCCCTCTCACGGCCCAACTGTCAGCCTCACATGCCAACCACGCGGCGGCGTGGCCAACCAGCATCCTCCACGTATACAAAAAGAAGGGGTCAATCGGGTGGGGCCATCTGGGCCCGCGCGGTTGGGCGAAAAGCGGGGGAGCCCGCAGCTGCAGCTGCAACTGGCAAAAAGAAAAAAAAGATAAGCTGGGATGGGTGTGAGTGGGGAGGGGGAGGAGCTTCCAGAACTCTCGCCCCGGGCCTTCCCGAACGTTCGCCGCCACGTCACCGCACTAGGCAGCACACCATCCCGGGGGGAAGGAATGGAGGCTCCGCTTCCAGAACTGTCTGGAAACTTCCGCCCCCACATCCCAACCCACCCGACCCGACGGGTATATATATAGGCCGGGGTGGGGGCGCGGATGCTGGTCCT

>Os03t0745000-02 2000 bp upstream sequence

GCAACGAGTTCATATCTTCCAACCAAGTCCACCGGCAGGCGGCAGCGGTGCAGAGGCGATGAGCTTCACACGGCACCTCATTGGCAAACTCATGCATCCCATGATGTGGAGCGGTAGCAGACTTGCCGAGGACTATAGCCGCCTCCACCATCAACTCCTTCAAATGACAATGCCGCCCGACGGTGGTCGTGAGGCCATCCACCTCCGCGAATCTTCGCTGTCACTACTGCTCAGTCTTGGCCGCTGCTTGGCTTCCTCGAACCAATTAAGAGGTTTCACTCACACGTGAGGCCACGGTATTTTTCTATCTTTTTCCTTAATATAATACCACCTCAACCTACCATGTCAGTAGAAACCACTTCCTAAACCACTAACACAATCAATTTACATCGGTTTTTAAAGTGGAGAAAAACTTTATACCGTTAAAGACCATGATAAGATGAGAGGGGGTGTAGTAGACTTATTCCATCCACCTGTCTTTTCCAAAGGAACGTGTCACGTAATCGCGTGGGTACCTTGTGCCCCCCGAGGGTGAGAAAAAAGAAAGACCGTCCCCTGGATGACGTGGCAAACGCTCCAGGCCTCGGCGGCTGCATTGCGTGGCGAGCGCTGCAGCGGCCCAACACAGCGAGCGTCGTGGAACCCTAAAAGGCCTGCAAAGTGTACACAAGAGACGGGCCGTACAACTAAAAATTTAGCCTTCAACCGCGGCAGTATTCGCCGGCCTTCTTCGCCACATTCGGCCCATCTCCCCCAGTTCTCCTCTCCGATTTCTCGCCGCCCTTCACGTGGCTCACCCCCCTCCCTCTCACGGCCCAACTGTCAGCCTCACATGCCAACCACGCGGCGGCGTGGCCAACCAGCATCCTCCACGTATACAAAAAGAAGGGGTCAATCGGGTGGGGCCATCTGGGCCCGCGCGGTTGGGCGAAAAGCGGGGGAGCCCGCAGCTGCAGCTGCAACTGGCAAAAAGAAAAAAAAGATAAGCTGGGATGGGTGTGAGTGGGGAGGGGGAGGAGCTTCCAGAACTCTCGCCCCGGGCCTTCCCGAACGTTCGCCGCCACGTCACCGCACTAGGCAGCACACCATCCCGGGGGGAAGGAATGGAGGCTCCGCTTCCAGAACTGTCTGGAAACTTCCGCCCCCACATCCCAACCCACCCGACCCGACGGGTATATATATAGGCCGGGGTGGGGGCGCGGATGCTGGTCCTGGTTTCGACGAGCTCCATCATCCAAGTACGAAGGGGATAAAGGGACTCATCACTCCACCCAGCTGGAGAGAAAAAAAATCTCAGCTGTACTTGCTCGTGTTCTTTCCTCTCCCTTTTCTTCTCCGCCATCATTTTCTCTGTACTTTGGGGTGTACTTCATCCTTCTGTATTTTCGGTTCTTTTTGCTCTGATTTGTCATCTTTTTTTTGCGAGCGAGCTCGTTCGCTCGCCAACGAACACTCCTGCTGTAGCTGTTCTTGGCGTTAATGAAAGCCCTGCATTCTCGCATTTGCAATCGCCATTTATTCAGCTGTGATCTTTGTAGAGTGTTGTGTTGATTAAGAATCGCATGATTTGGGTTCCATTGGTTTGTTTTTGCAAGAGTTTTTGTGTTTTTCTCTGATCCCCTTTCAGCTGATTGAATGATCATCTTCTCCCCTAATTCTCCATCTGTCTTTGGTGGTGATTGATCACTTAAACTCTACACAATTTGATATTTTTTGCAAACTTTTCAGAGGCAAGATCGTTCCAGATTGAATTCGTTCGGTGGAGGGCGATATATTTTTTTTCGGCAAGGATCGCCATTCTCACCGGTCGTTCAAATTCTTCTCCTGCGTTAAGTGGCTGTGTGTGCTCTGTTCGTCATCTTCCTCTGCTACTCAATTGCATTGGTTGGTTGGTTAGACATGAATCCATTGCGCGTCATCGTGAAAGAGGAGGAGCTCGACTTCGCCGCCGCGGCGGCGGCGGCGGCGGCGGGTGAGGGCTCGCCGTCGTCGTGGGCGGT

>Os03t0786400-01 2000 bp upstream sequence

ACTCTTGCACGTGTTGTATGTACGACTTCGGTACAGAATCACCAAGTCGTTACAGTAGACCAAATTCATTTCACTATGCACACAGCTCTCCTGTGTGTTCCTGCTAATGATTCAGATCCACTTAATTCTGTTGCAGCAGTTGGCTAGGCAAAAAAGGTAGTGGGCACCTCTCGTACCACATTTACCAAAAATCACATGCAAATGCGGGCAATGTATCAAGTACTAACATTATATGGGAGATGCCGATAGTTGCAGCTTCAAGAATCTCTAGCTTACTTAAACAATTCAGATTTTCATTTAAATTTTGAGAACTGTAGTTATTGAATTTAGAAAATGAATTAAATTTGGTTGTGTGTCTAAAAGTGGAGGCCGAAATGTAAATAATTTTCACTATCTAAAGGAGAAAATAAACTAGAAACAAAAAATTAGAAAACTTATTTTCTAGATTTTCATCAGCTAGCTACTTGGCTGTTTTACCAGATTGTTGGTTATGAGTGAGGGAACTCCAACTCGAATAATTATATGCTTGGTTTATCAAGCTTGATTTTTTTTTCTAAATTAGAACTAGCATAGATTAATCTTGTTGGCACGCTTTTTAAATTGTTAAATGCGTTTTTTTTAAGTAAGAGGTTGTAGGGTTGAGAGTGTCCTACCTCGTTTTAGGTGTGTCTGAAAAAGCTAAGCCATCGTAGTCAAACTCCATACACCATTCTTAGACCAGGTTTTAAATACTGTATTTTAGACCATAAACTCAATATATAATGATGAGCTAAAAAAGAGTTTGGGCATGTCAGTAGAGGACCCAAGCACAAGCTCTGTTTGGCGTGCAAGTTGGCACCAGTCCTACATACTGTAGTACTCCAACGTACACGCCCACATTGCTGCCATCCACTGCGGCAGTGCTGCTTCTGCTGGTGGGCCCCACCTCGCTCCGCTGTGGGGCCCACTTCCGGTGGGGGCCAGAAATTATTGCGCACAAGACCCGCAGGCCCAATGGCGAGCCGCCACGTGTACCCGCGCCTCCCCTCCCCGGCCAATCGGGAGGCCTCGTCCCTCTGGGATCCACCTGTCGGTGGCACAGAGACCTCCCCACACACGCTCGTCTCCCACTACACGCCCCCCCGCATATATTCCCGTCGCCACCACGGCGCAACTGCGCGACCGGACCGACCGAGACCGCCTTGTCTCCTCCTTTTCTTTCCCTACTTTTTTTTCTCCACTCGTGTGTAGTGAAAAAAAAATCGCCCACTTTAATCATATTCCCTCTGTCTCAAAATATGACTGACGATTAATTTTAGCAAGGTCTCAAAATTAAAGTGTAGTATTTTTATCGACAAAATCATTTTTCATATATTCAAATGTTAAAAATCTAATGGTAGGAGTACTAATTAAATTGACATTAACCTAATTGCCGGTCAAATTCTGCAACAGTAGAATCGCACCTTTTTTAAAAAAAATTTAGCACGGAAGAAACTAATATGATTAATTAAATTTTGCATAGTACCGGTACTGTGTTGGATAGTTGTACGGAAAAGGCTATACGTACTAACTTTTTCTTACTAAATTCTTACTAACACTGATGTGTCATGCTATGAGTGCATCTAGATTTTTTTTTAACCTCCATCTACTTAAATCAAATGGTTGAGATGATTGTTATAAAAGTTTAGTAAGAAAAATTTAGTACATTTAGCATTGCTCAGTACTAGTGTTGTGACTCGACTCCAATAATAAAATAATAATATTACTATTCCATCTATTCATGTCCTTGCGAAGCCAAATAGCGCTGAGGCTACTGCTTGCAGCAACAATTCTTCCTTTTCTATTTCCATATATAGAAACACCAACCTCCTCATTTCCCCCCTGACCCCAACCCCAAACCCACTCTACTCTACTGTGCCTCACCTCTTGCCACTACTATTTCTAGTAGTCGTGTATCATCATTTCAGATATCATATCGCCACCTCTCGTTTTTTTAATAATATCAGCGGCGAGCGAGCGAG

>Os04t0434600-00 2000 bp upstream sequence

AGTTTGTTGTGTTTTTTTTTTTTGTGTTTGACTATGACGAAGTAGAGATTTATGAATGCGAAAATAAGATTAGATCGTGCAACATATTAGTGTGAAAATAAAAGATGCTATGCATGCAACTATAATTATAAAGAGTGAAGATACCGACATTCACGTATGGTTCATTTTGTTCGAGGAAGCAAACCTTTACTACGAGGTGATCTATCGTAATTTTAATGGATAGTGACTACCGATTTTTTGGTTGGTATACTAAGGTTGTCATTTGCAATTTTTTTTTGCCACACCTTATTTAAAGTTCATGAGGTGTGGTGCCAAGAGAAAAGAGAGAATCTTACCACAAGTATAACTGTAAACCAAATAAGTAGTGATCAACTTGATCAAACTCGTTCAATATAAGATGTAGCAACCTTGGGCTACATGTGACATTAAACCTTACACAATGCATGTAGTGAATGTCGACGGAGGATACTTGTAGACCGGATATAGAGGGTATTGGGGTATGCTGGTACGAGGATCTACGTAATACGACATCAAGCAAACAGAAAACAGAGATTATACTGGTTCAGGCCCCTTAATAGGCAATAGCCCTAATCCAGTTGATATGGGATTATATGATGGAAGTCACAAATTACAAAGGGAATAATGGAACTCGATGATACCGACGAGATCATAGTCGAGTTGATTCGACTAGATCACCCGACGACTTGGCTCCTGTAGGCTCCGGCTTCATAGGCTATGGTGGTTATCTTGGCTATGAGATTTGATGTTTTAGGTCCTCCCAGGGGGTCATTTTTATATCGCAGGTCAGGCGGTTTCCAAGTAGAACTCGGAGACATCGGACCCTGCACGATACAGTGACGACCATCGATGGACTTCGTGATGAATTTCCTTAGCGTATACAGAGAACGTCTGTACACGCGCAGATATACCATATTGCTGTATAGCGTATATCTAAGGGTAGAGGATATGCCTTATCTGTAACCCTGACAGTGAACTCGTCACCCACCCATAAAACCTAACCAGCAGAATATAAAATAACCCCTCACTCCATTTTCTATTTTTCACTAATTCAAATAAGTGATAATTATATGTGGTGAACGAGCCGCCTATGTTTTCACTGCATTTTTATTTTCAACACATGTAGATAAATATCAGTCACTAACAAATGCTACTCGTTCTCCAAGCTCAACGATGTGGATGACTCTACCTGTGGTCCCTGAACACGTTGCTACTACGAATATCTTTTTTTTTTTAGAAAAAATGAAAACAACGAGGCTGCTCGTTAGACGTTGCGATCCCGATAAAGACGGCGGTAAGCGCACCTCCGGTGGTCAAACCCCCCCAAACCCCCACAGAAAACTTGAGCGCTTTTTAGACCAAAGCACCAAAAAGGCGGCACAAACCCAAATCGACAAAAGACGCCACGGTGACCCAGGCGCCCCTCCGAGGAAGACGACGACGGCTGCCTGCCTAGCCAAAGCGAGAAGCCCACGAGCTCGCGGCAAACCAACCTCGTGCTCGCCCAAACCCTAGCCGCCGCCCCGCACCTACCTACCCCGGGGCTCCGCCGCCGCAATGTCGTCGTCGTCGAGGCCGGGGCGGGCCAGCATCTCCCCGTTCCGATCCCGCCGCACGTCGGCGGCGGGGGGCGGGGCGGGGGTGGCGGCGGCGGCGCATCCTCCTCCGGCGAGGACGTCGTCGGGGGGGCGACCCTCGACGCCTTCCTCCTCCTCCTCCGCCGCCGGCGGTGGCCGGCCCACTACCCCGTCGTCGTCGTCCGCCGGTGGCCGGCCCACCACCCCCTCCGCGGCGTTCGCGCGGTCCACCACCCCGTCCTCCGGGAGGCCGACGACGCCCTCGTCGGCCTCCTCGAGGGCGGCCGGGAGGGCGCCGCCCGTCGCGGCGGTGGACGCGGCCAATGCCAAGGAGAACATCATGGTCACCGTCCGCTTCCGGCCCCTCAGGTGCGCTGCTGCTGCTGGTTCGATCGCTCTGTGGGT

>Os04t0481300-01 2000 bp upstream sequence

GGTTATGTTAGAGGATGACATTACTTGATTGATTCCTTCATAAGCTTGAAAGGGTTGTCCCCTGAGTTGATGAATACAGCATCAACTGCTTCCAGTGTCTGAACCTCAGGATCACCTAAAAACAGTCCACCACTGTACTCTTCAGAAATTCCAACCGAAATTCCAAAATTTGCAGTTCGTTTCAATTCGAACACCTCAGAATCCAGCAAATCCAGAATAAACCTACCGCTCTCGAAGCAAAATTGGAGCTCATTCTCAGGACTCCCCTGGAGGCTGGCTCGGAAGCCGCCGTCAAGAACCGGCAGCATGAGCGCGTACAGCGCGGCACCCGCCTCGCTCCTCGACTCCAAGAGCAGCATCTGCGTCTCCGCTGGCACGCCGGCGGCGTCCTCGCCCACCGTCGGGATCATCCACCAGATCTTGAACCTGAACAGGGACAGCCACTTCCACCCACTGCACACCAAAACCCAACAACGAATCAGTACCACCGCTGCCGGCCGCGGCGCACACCGCAATCTCTGCTCTGGGGGCGGGGGCTCTACCTGGCGAGGGTGCCGAGGGAGAACACGTGGCGGCTGGACGGGGCGGGCGCCGTGGCGCCAAGGAACGCGGCGCCCCCCGGGGAGGCGACGACCGCGGGGCGGCGGAGGGCCACGCTGGGAGGGGCCCGCTCCAGGAGCTCCCGCCCGCCGACCACCAGCGACCCGCGCTCCAGCCGCGCCATCTCCTCGCTCCGCGGGCACGCGACGGCGGCGGAACTGAGGCTGGGACGCGGCCACGGCCGGCATTGCGCCGGGACGAGGCCGCGGGGGCGCGAATTATTCCCGCGCGAGGGAGTAGGAGGAGGGGCCCGAAGCGGCGACCTCCAGGGCGCCGTCCCCGTCATCGCCATGTCAGCTTCGAGCTGGGATAGACTAAGATATCCGGTAGTACTTCCGCTGCCATGATCGGGACCCCCGCGTGTGGATGGGGGCGCTCGCCGCGACGCGAGAGAGACGGCGACGCTCGGCTCGCGTGCGCGACTACGCGGGGGGGACGAGGGCGACAGCTACGAAATCGGGGTTTTTATGGACTATTTGGCCCCCTTCCTTCTCGAAGAGCACTGCTAAATGTACTTAGTCCTTCCGTATCATAATATAGCAATATAAAATCGAACGAGACATTTCATAGTACAATGAATTTGGACATACTTCCTGTCCAGATTAGTTGTATTATAAAGGATATGTTTAGTTCTCACTAAAATTAGAAGTTTGGTTGAAATATGAACGATGTGATGGAAAAGTTGAAAGTTTATGTGTGTAGAAAAGTTTTGATGTGATGGAAAAATTGAAAGTTTGAAGAAAAACTTTAAAACTAAACACGGCGAAAATGTCACATCTTTATAGCACAACTAATATGGACAGGGAGCATGTCCAAATTCACTATACTATGAAGTGTCTCATCCGATACTAGATTGCTATAATATGGGACGGAGGGAGTAACTTCTTTCTATTTTTACAAAACTCTCATAAACAATAATATCATGCGTTTAATTTAAGAAGATAAAAATCATAGATATCTACGTATACTCACAGCATGATACATAGTCTAGTCTTAGTAAAACTTTAGGAAAAGAAAATTTAATACTAATAAACTTTTCCATTCTCAAATACCACTATCCCCTCTTGGATGACTTCGTTTTTTTACTAGAAAGAGACGATTTTTTTTCTCTCGTGTCAATTTTTAGCCAGCGAAATGCCACGTCATCATTTATGAGGGCAAGACTATCATTTTTCCCTCAGATATGGCGACATGACGCCTCGCGGGCTAAAAATAGCACACGGGCTAAAATTTGAATTTCAGTTTAAATTATTTTAAAGTTTAACCTGTTTATTGAGGAATGAGGATGATATTTGAAGCCACATAAAACGATGGCCTGAAAAGAGGGAGTTTTGATCTGCACTGGACTGTGCCACGGTCACTACACAGTGCACACTGCGACGCCAACCACTCGAACTCCC

>Os04t0560600-01 2000 bp upstream sequence

TGGGAATATGGGCTATGGCCGGCAGTTTTGGTGTGTCCGCAAAGGTGTCACCTGCTGTCTTCCTTGTTCTTTCTAAATTGTAAGTTCAGGCTCAACAGGGTCATCTGATATATATACACCAACTCTTCAAGGCTTCATGTGTAGGGTGTTGCCTTGCCCACTGAAAAAACCTAGTCACAACTCACAGTCATGGCTACAGCAACCTAATCTTCTAGAGGAATACTACTAGACTGGGTCCCTTCTCGTCTACTCTTCTCAACCAACGGTTATGCAACTAGTGTTTGCTTACATGTTAGTTGGTAGAGTCAGTGTCTTCTAAAGTTAACCAAAGTAATAGGTAGGATTAGGAAGGTGCTTTGGGGTAATAAAGAATAAGACTCAAGCGTGTGTCAAGAAACAAAACGAACCCCTGGATCACACTAGCTTGCATCAGCATACTAGTATTTCTTTTTTCTACTTAGTCAACCACATCTATTAATTTGAAAACGAGTGCAGTTGAAATTCCATACGGCCTTACCGGCATACAAAAGGTTCACATATTCTTCCTTTTTAGTTTTGGTTTAACACAGTGTTCCTTCGTTTTTGTTACTAGGCTTCAAAAGAAACAACGAAAAGCTCCTTTTGTTTTCTGGACTCGAAGTTAGGTCTTCACAAATATATATTAATTAAGGACAAAGAAAGACCTAGACTGAGTTTGCATCAAGAATCATGATGAACCCATTCAGGCCTTCACACCACTCTAGTGCTGCAATTCTGAAAACCAGTTTACTTGTTATATACAACCACAGCTGTTTAATCTGAGAAGAATTGATAACTGAAGAACTGTACCGGTCGGTCACCAAGGGTACTTATTACTGTACTTTCTTACCTTGGACTGCAAACACCACACATATGCTAGCCTTCAAAGGAACTTTTGTATATTGATATCCGTAAAGAGAAGAAAAAAAGGTTTTATGACGTATATTGGAGAGAAACACATGAATGGACTATTTCCTTAGTTTATCTTTTTGGCCTCTTTTCCCATGGAAATGATCTCACTTCGGATTCATGCCCCCAACCCATCCGTGTGGGCGACGAAGTACACTGAACTTCCGAATCGAGTTTGACTCTCCGGATGCTTTATCAGAAGCGCAATGCGGCGTCCCGTCACGGCATATCGGTCCGAAATGGTGCGCGGTGCCCGTCACGGGGCAGGTTGGCGCTGCTGCGTCTGCTACTCTCCCTGCTCAGGCCACGCGCCGCTGAAAGAGATTAGTAGTGGAAGAGGTGGCATCCGCCCGCTCTCTCGCGTAGCGTAGACATCAGCTGAGCCCCTCGCCCCTAGCCGATCGACGCGGGGCGCCACCGGCGGGCGGCGGGCGAGGCGGCTCCGGGTGTTAGCGATTGCCAGAGCGCGCGTCACCGCTTGCTGAGGCCAGAAGCCGTGAAAGCACATGCCACCATGTTAATAAAACGTCTTTTTGAGTCAAGATAGACTTACCAGGTCATTACCTCCAGCCCATAGTTGATTCCGCTCTGAGTTTTGTAAGCCGGAATATCTTCTGGGCCGGCTTGGCTCGTCACATTACTCCGGCCCACTGTTGATTCCGTTCTGAAAAGGGGCGTACTTTGTTAGTGCTGCCTGCGCCTGATTCTGACGATGTTACCGAAAGCTAGTACAGCATATCCCTCAAGTAAAATGGCTATTGCTAGATCAATAAAAGCACGTAGTGCTATGTGTTTCTACACCTCCAAAGCCGAATAAAATCGGCTGTCGCACCGCCGCAAGTATATTGTACTGCCTGCATAAGGTGAGATAGCGTGATCCCGAGGCTTTCTTTGAATCTTCCTTTCCCTCCGGAGATAACCAAACAAGGCATAAAGCTCAAAGCCCGGACCAAAAGCAAACAGCGAAACGAAGGGGTAGGGCATCACGTCAAGCAACTCCGCTCCCGCCGCCCACCGCCCGCGCAGAGAAAGAGAAACCCTTCAAAACCTCGGCTCCTCGTTCTCCCAAACCC

>Os04t0612500-02 2000 bp upstream sequence

CTTAGTAGCCCGACCGCACGGGATTAAAGATTATCCCGTGCGGTTGGACCACCCGCACATGAAAGTGTTGATTTTCGCAGACACCTGAATGCAGACGGACGGGATCTCTGCATGAAAAAATCCATTTTGACCGCACGGAAAAATCCAATCTGTAGTAGTGTACAAGTTATATATGCCCGTACACGAATCAAAACAAAGCTTTATCGTAGTCCAAACAACTGATCTAGCTAGGATCCAGTTGTGTAGATGTGTGTGCACACCCCTGGTCACACGCCACAGCTAGCTCAGCTCTGATCGATCTCCTTTTTCTGTTTTGTTGTGTTGTGTCTGGAGGAGGAAGAAGAAGAAGAAGATTGGATTGATCTCAGCCTCTAGATTTTGGTAGTGAAAATAAAATGTGATTATATTGAATAATTGAAAGGAGTTGAAGCTCACTGGCCACCTATCCTAGCTCTAGCTAGGCCTCTAAATATCTGGGGACAGTGCCAATGGAGTAAGGGCCCATAGGCATGCATATATGCCATGCAATGTATGCTGAGTAATGATGGTTTCATACTTTCATTTGCCTTCATGCATGGCAAGATCAGAGATGATTTAACAGCTAACCCGCTGTCAGTACTCGGTGACACGTTCATTAGCTAGCAAAGGTAAGACTGTTCTGGATTCCAACAATAAGGGCATGTACAATGGTTGTCAATAGTGGTCTCTTAGCGTTACTAATTAGGAATACTTATTGACATGGAAGAAAGAGAAAGAGAAGAGACAAAACGTCGTTGTTATACATGACAACGGCTTAACAACGACTCTTAATCACTATAATATGAAAATATGGTTGCACGAGGGTAAAAAAAGGGAAAGAATATTAACAAATGGAATATTTTTTGAGTTAATGATTAGAGTCTTTGTCGTCTCAACTATTGTAAAGACAGGGTCTCTAAATAATCTTGTATTGTTTAAGAGATCATACTCGTCTCTACCATTGAACGTGCCCTAATAGTGAATTATTTTGCATGCGGACCATGCTGTGTCGATCGATTAACTCACCTACCATGCAGTAATTTGGAGGTTGCATGGGCACCATGGAATATCCGAATAAATAGAATAGACATAAATTAAAGGTCCTCTTTCAAAAGAAATTTAGTGATATCAAGAATTTATCATTAATTTCTACTATATCTTTCGACGTAAAAGTTACGATTCTTTCTTGGGTGTGATATGCACGGAAAATATTTTCTACATCTAGTACTTGAATAGGGAACCTTTTTAAGATAACAGAAGAATTTTATCAATCTGAGTCAATTATATTAAAATGATACAACTGCATTAAGAATAGTCTCGACCTGTTGACTGTTTTAAGGTTAAAAAAAAAAAAGCTTTGCTCTTTTGACTGTTGACTCCCCAATCAGTCGGTTTTGTTTTAACCGCGACATGCAATATGCAGGGGCCCCGCATTTGCTCACAGGCTCACATGCCTGCGCGAGTAATCCAAAGCACGAGTCATCCAAATTAAGTCCAAACCGAGATGACTTGGTTGAATTTCAGCAGCAGCAACCACCGACTCGATGCATGCCAGCCACAATCCTCGCCACCGCGCGCGACGTGACACGCCAAGCTCTACCTAGCTAGCGTGCTCCATGTCCATCACTAGCTAGCTTCAGTGACGGCCTCTCGCCTCCACCAATGGCCTATAAATCCAGGCTCCCAGCTCGTCTTCTTCCCTCGCACACCAGCTAGCTATAGCTAAGCCCCCCAAGAGACATCGAGAGCGAGATCGCCATGGCCGCCGCCGCCGCCAAGAACGGCCGCATTGCCGTCGCCGCGCTCCTCCTGGCGGCGCTGGCCCTGTCCGCGCAGCTCGCGCCGGCCGCGGCGTGCTCCTACTGCCCGACGCCGAAGCCGCCTCCGCCGCCGCCGCCGGCGCCGTCCGGCGTCCCGTGCCCGCCGCCGCCGTACACCCCCACCCCGGCGACGCCGACGGGGAAGTGCCCGGTGAACACGCT

>Os04t0615000-01 2000 bp upstream sequence

AATTAGCTCTAAATTGAAGGTTAAAAATTTAAATTTTGGCTGATAAGTATAAGCATAAACGAAAAGACGAGACTCTCGTAACCCTAACTAACCCAGAAGGGATCAAGGGGCTCACGCAGCGCCACGTCACGTCGCCCCCCCCCCCCCCCCCGGGTAGGCGCGATCCGGGCCGTCCGTTTTGTCGTGGGGGAGAGACATGGACGGCTCCGAACCAAACACCAACACACGCACACACCCGCAAGCGCAGCGACACATCACACGGGCTCAACAGTGTAGTTGGTGTTCCTCGCATCTGCGCCCATGGCAGCCGCAGCTGCAGCAGCAGCATGATCGCCACCACCACCACCTAGCTCCTCCTCTCCCCTCCAGATTCCCAGCACTCTCTCCCTCTCTAGACCCCTCTCTCTCTCTCTCTCTCTCTCTCTCGCTCTAGGCCTCTAGGGGGTTGTCGGCGTGCGCACGCGTACACGCGCGCGCGGGGGGGGGGGGGGGGGGGGGGGAAGAGGAGAGGAGGCCTGCAAAGCTGGCGCCTGCTGCTGCTGCGCCTGCGCCCTGCCTCGCCCAAAACAGTGGAGATTCCCGTATCCATTTCCCAATTATGGGGCGGGGCGAGGAGGCTAGGAGGAGGATAGGGTGAGGCGGCTCTTCACTTCTTCAGAGGTGGCAGGCCATTCTGCTTCTCTCGCCATTGGAGTGCTCCGGGTGAGGTGAGGTGAGCGGCTCGAGATCCTGCCAAGATCAGCTCCTTGGCCTGGGGGTGGAGGACAGGTGGTATCTTGTGCCGTCTTTCTTGAGTTCTTGCGGTGGTTTTCTTTTTTTGAAGTTTGTTGGTTTCTTTTCTTCTTTTTTTTTTTGGGTTGGTTCGTGATTTTTTTGTTGTTGTGATGGCGGCTGAGTTGTTTGTTTAGTCTGTTCTTGATGTTTGCTTAGTGGTGGTACTGATGTGTGGGTAGGGTTCGTGTTCTTTTTTTTTTGCGTTTATCTTCGCTTTCCTTTTTTTTTTCTTCTTCCCCTTTTGAGATTTCGATCGTAGCATTGGTTAGCGTGTTGGTGGATGTAGTTAATCAATCCCCTTTTAGGTGCCAAATGTCATGGAAAGCGAGCTCTTTGCCCTTGTTTAACCAAGCTTTTGATCGCAGCATTCAAGAATTGGCTCCATCTCCCCGCAAAAAAAAAAAGAAAGAAAAAGGAATTGGCTCCATCGATTCAGTAAAAAAACCCGAATGCCGCGGAAGTTAGCATTGCTCATCCGTGGATTCGACGCTGGCACACTGGTTTCTTGGCTAGAGAGAAACAACGATGTGCTTGCTGTTTCAGCCACCAATTTCTTCCCGGCTTGTGATTTTTGCCTTTTGTATCTGCTTTCTTTTTCCTCTTTTGAGAAAGTACACGATTCCTTTGCCTACCTTCATTTTCAGTGCCGATTACTAAATTTGCAGTAGGTCCTACTCTCTCGCCGTGGTGTTGGACTATCATTGGGAGTAGTTGTCATGTGGATTTATGCCCATTTGCTGCGATTAAAGAGAGAATATGTGCTACGCCTTAACGTTCATGGACCTGATTTCTTGAGGTCGCTGTAACTGTTGCCTTTGGAGAACAAGAGGAAGAGGGTGTGAGTGTGTGGAGACACTAACCGTGGCTTTTGCAGATTCCTCTTATCTGTTTTCCTTGCATGGCAGTTGTGCCATACATTGATACAGATGGATCATATTCTTCGTACAACCGGATTGGAGTATGCTCGATCTGTCATCCGTGGAGAACCCGAAATTCAGATTGCTTTTGTTTTGCGTGGCTCTCCTTTGCAGTTCTTGCGGGAACCTATTCCCCCAAGAATATCATGCTACTCCACATCTTGTTGTTTATTCTACTGATTCCATCTTTTCGTTCGGGCCAGGCCAGCTAGCTAGCCGCAAGCGCTGACTGTCTTGATCATTGATTCCTCCTTCCACAATAACTCTAAAAGATTGGAAGCACATTTGCATGATTGATGGTTTTCCCGT

>Os04t0629100-01 2000 bp upstream sequence

TCAAATTTGTGAAGTGCTAGCGATGCTGTGTAAAATTTTCATTAGACAAGTTCTGATTTTGTGTAATCAAGCATAACTACTAAATTCATTCAAGAAAATATGCATACAAAAAACAAGATCATAACAAAAGGGAATCCACTGCACATTACACAAATAGGAACACCGCAACGCAAAGATCTCAAACGACGGAAATGCTGCCAATAATTCAGAGACGATCGAGTTCATGTTCAGAGCGCAAGAATGGCGCCGGCGCCGACTGCGAGGCCCACCACCAGCTTGGAGGCGAGCTCGGCGCCGCCGCCGGCCGCCGCGCCGTACGACGACGAGCCGCCCTTGGACGACGACGGCGGCGCCGTCGAGTTGGAGACCGTGATGGCCACCTTCTGGCCCATGCTGCAGTGCGACCCGGACGAACAGGCGAAGTACCTCGTCCCGGGCTTGTCGAAGACGACGGTGGTGCTGCCGCCGGCGTCCGACGAGAGCGGGGCGGCGAAGCTGCACGCCGTGTAGTCCGCCGCCGACAGCTCGTCGACGGTGTGCGGCGTCCCGTACGTGAACACTGCGCCACATTACACACGCCGATCGATCAGCTCAGACGCCATTGTTGCCAGATGCCAAGAACGGAGGAGGAATGGAGAAGCTCACTCACCGACGCTGTCGCCGACGGCGACCGTGTTCTTGGACGACCAGTCGCCGTAGTTCCCGCCGGAGGCCCACTTGATGGCCCACGTCTTGCTGCCGGCGGCGAGCTCGGCGACGGTGGCCACGGCCATGAACGCCACCAAGGCCTTCAACGACGACGACGACGACGACATTGTTGCGAGCTCTTGCACAAGCTAGCTAGGAATGGAAGAGGCTGCAGAGAAGCTAGCTAATACCAGCAATGGCGCAGCTACTGCCTGAATTTATATATCAAGCTGCTGGTTTTGCACCAGCTGGTGGTTGGATCGTGGATGACGATGAAAATTTTGTATAGTTACCTGCTCGTGCCATGTCCCATTTAATTTGCAATCCAACTAACTAAGAGGTTGACAGCATTATGCCAATGATATGGCTAATATACAAGTAATTTAGGCAATAGTTAGTTGGATTAATCGCATATCTGGGGGAGTTCATACATATATGATCACGGCACTATGACTATGGCAGGTTTGATTGATCGGATCGAAGGAGTTGACAAGGTATCAATTCAGCAATTCGATTATACTCGCTCCAAAAAAAAAATCCAATTTCTAACTATAAATCTAGAAATAGGATATATCTAAATTCATAGATATATATATACATGGGCTATACTGCTATGCATCTAATGAGAATTGTCGATCGAGCTGGCGCTCACTAGCTACCCAGCAAGTGGTAGATAATTAAGTTCCATCTCAAAAAAACAAAAAGAAAAGAAAAAGTTTGTATATTGTTTATGTATTGTGCATTGGGGACCTAGGAAGTTGTTTTCTGCTGTATTCGGTGTATCTAACACTCACAAATATTACTAGTAATTAGCAGTGTATGTTGCATTATTGAAATGATCATCACAAGCAATCTCGATGCACGTCTTGGTTACAAAGGTTGAAGGCTGGATGTTGACTTGACATGGTGATGGTGACGAACACATAAACTCGGAATGGAACTGGGCTAGTACATATACTCCCTTAACCCAATATAGAATATAATATGATACATCCTACCTACTTCTATACAGATTCATAGTACTAGAGGATGAATTACATTCTATACTACGGATTAGGTACTTGGTGGTGGTTTAATTGCTTTAGCATTCTTTTTGGGGGAATTCTTGCATAAGCATCAGGGATATGATGACAATGTTAGTTGACTGGAGTTGATAATCGCAATTTCACATATACCATATGTATGCAGTTTACAAGTAACATTCACATATTCCTTATGTGTGTGCAGTTATGCATACACCGGCTGTCAATTTGAACCAAAAATGGCAGTTTCCGTTTTCAACAAGGAGGCGTACTTCCCTTGTTTCCGCCTTC

>Os04t0629100-02 2000 bp upstream sequence

TCTGATTTTGTGTAATCAAGCATAACTACTAAATTCATTCAAGAAAATATGCATACAAAAAACAAGATCATAACAAAAGGGAATCCACTGCACATTACACAAATAGGAACACCGCAACGCAAAGATCTCAAACGACGGAAATGCTGCCAATAATTCAGAGACGATCGAGTTCATGTTCAGAGCGCAAGAATGGCGCCGGCGCCGACTGCGAGGCCCACCACCAGCTTGGAGGCGAGCTCGGCGCCGCCGCCGGCCGCCGCGCCGTACGACGACGAGCCGCCCTTGGACGACGACGGCGGCGCCGTCGAGTTGGAGACCGTGATGGCCACCTTCTGGCCCATGCTGCAGTGCGACCCGGACGAACAGGCGAAGTACCTCGTCCCGGGCTTGTCGAAGACGACGGTGGTGCTGCCGCCGGCGTCCGACGAGAGCGGGGCGGCGAAGCTGCACGCCGTGTAGTCCGCCGCCGACAGCTCGTCGACGGTGTGCGGCGTCCCGTACGTGAACACTGCGCCACATTACACACGCCGATCGATCAGCTCAGACGCCATTGTTGCCAGATGCCAAGAACGGAGGAGGAATGGAGAAGCTCACTCACCGACGCTGTCGCCGACGGCGACCGTGTTCTTGGACGACCAGTCGCCGTAGTTCCCGCCGGAGGCCCACTTGATGGCCCACGTCTTGCTGCCGGCGGCGAGCTCGGCGACGGTGGCCACGGCCATGAACGCCACCAAGGCCTTCAACGACGACGACGACGACGACATTGTTGCGAGCTCTTGCACAAGCTAGCTAGGAATGGAAGAGGCTGCAGAGAAGCTAGCTAATACCAGCAATGGCGCAGCTACTGCCTGAATTTATATATCAAGCTGCTGGTTTTGCACCAGCTGGTGGTTGGATCGTGGATGACGATGAAAATTTTGTATAGTTACCTGCTCGTGCCATGTCCCATTTAATTTGCAATCCAACTAACTAAGAGGTTGACAGCATTATGCCAATGATATGGCTAATATACAAGTAATTTAGGCAATAGTTAGTTGGATTAATCGCATATCTGGGGGAGTTCATACATATATGATCACGGCACTATGACTATGGCAGGTTTGATTGATCGGATCGAAGGAGTTGACAAGGTATCAATTCAGCAATTCGATTATACTCGCTCCAAAAAAAAAATCCAATTTCTAACTATAAATCTAGAAATAGGATATATCTAAATTCATAGATATATATATACATGGGCTATACTGCTATGCATCTAATGAGAATTGTCGATCGAGCTGGCGCTCACTAGCTACCCAGCAAGTGGTAGATAATTAAGTTCCATCTCAAAAAAACAAAAAGAAAAGAAAAAGTTTGTATATTGTTTATGTATTGTGCATTGGGGACCTAGGAAGTTGTTTTCTGCTGTATTCGGTGTATCTAACACTCACAAATATTACTAGTAATTAGCAGTGTATGTTGCATTATTGAAATGATCATCACAAGCAATCTCGATGCACGTCTTGGTTACAAAGGTTGAAGGCTGGATGTTGACTTGACATGGTGATGGTGACGAACACATAAACTCGGAATGGAACTGGGCTAGTACATATACTCCCTTAACCCAATATAGAATATAATATGATACATCCTACCTACTTCTATACAGATTCATAGTACTAGAGGATGAATTACATTCTATACTACGGATTAGGTACTTGGTGGTGGTTTAATTGCTTTAGCATTCTTTTTGGGGGAATTCTTGCATAAGCATCAGGGATATGATGACAATGTTAGTTGACTGGAGTTGATAATCGCAATTTCACATATACCATATGTATGCAGTTTACAAGTAACATTCACATATTCCTTATGTGTGTGCAGTTATGCATACACCGGCTGTCAATTTGAACCAAAAATGGCAGTTTCCGTTTTCAACAAGGAGGCGTACTTCCCTTGTTTCCGCCTTCATAGCTCGAACACGTATGTTCATATCCACACCTCTCCCCCAAGTACGAGAC

>Os04t0676100-01 2000 bp upstream sequence

GCATCAAATCATTGCAAAATATACCAATTTGTAGTCAGTTATTTAAAATTTTGTGTGCTTCTTACTTCAGCTAGTGCCATTGCAAGAAGCACATCATGAACATATGGCCTAGAATCTGGGCGTCGTAGAGCCGCCTGACCAATTTCCAAAACTAGCTTCTCTTCTCCAACCTGAAATGAATATTGTAGCATGTAATACACCATTTCATTATGATAATGGTACATTCAATTTTAATAAATTCAAGCTGCTAGGTGTCTTGTATTCATGATCACTTAGAACTCACAAACATCATAATGGTGACTAACCTGCATCCCCTGCATTTTACGCTACCCAGTCCTAAGAAAGGGCAAAGTTTAAAACCTACGCGCTCCTCACCACATTCCTAAGGATAATTTACCCTGAAGTTAGCATAAAAGTTTTCTTTATTAGTTAAACATTATTGATCCTAAATCTTCTTGATCCCTTTCAAATCATTCAGTGAATTGAGAATAGTATTCTGACAAAGAATAAGGCAGACCTATAAAGAAACATACCGGGCATGGTCATGGTGACGACTATGACTATGTCATGTAACATAAAAGGTGACAAACCTAAACCAAACTTGGTGGTTTTCCTTTATTTGTTCACAATAATAAGGATGTCTGTGTTACACATTACCTCCTGCAAGACACAAAGTGCAGCAGGCAACCAACTCCAAGGTATATGAAGAGAGGATCTGGGTGGAAGCTTTTCTTTTATGCTTCCTGCGTACTCCTGTTCAAAGAGAAGTTTGTCTCTCACATCTACCAGCAGAGCCTACCAAACATGAAAGAAATAAGCAAAGATTAACCACATGAATTCTGCATTCCCTACATTAAACAAAGCAGTATATCCAAATATATTTCCAGAATTCCTTGATTCAGCGAGCAACCTGTCTGCAGGTAGACACCTCTGCCATGTACCCGTCTTCAATTTCTGCATTTTTCAGCTCCATTGCTGCTTTGACAATCTCATCTTTCTCAGCCTTCTCTGTAACACCAAGAATCTGCATTGGCCATTTGAAACATGTCACTCCAAAAATCAGGGAGGCGTGTCACGCGTGTGAACTACCAGTGCAAATTACAATAGGCATCGTGGTGCAAACACTAAAGTTGAATTATGGTCAAGAAACGACTATCTAATCTGCAACTGTATCATTCGAACAGAGACCAGCCGAGCTAAACTAGATCCATCGACGATATAAGACCACATTTCCAAAATTCGGATCCATAAACAGTACGAGTGGCCAAAAAAAAAAAAAACCTGATAGCAAGTGACGGGGATCTCCACCATCGGCGCGGCGGGGGCCTCCTGCCTGCTCCCCTCCGCGGCCACCGGCGGCGCCTCCGCCACCCTCGCGCGCGCAACCCACGCGGCCGCCCCTCGCCGTCCGCCAGCCAGGCATCCACCCCCGGCGTGTGCGGACGCATTGCCATTTCCCACCCCAACACGCCGCGTCGAGGACCCACATGCGACCCCGGGGACAACCGCCGCCGCCGCGGCCACCGTCGGCATCGCCATGGCTGGCCTGAAGACCTCACCCCCCCAACCCTAGCCCACCCTCGCCAACGCCACCATGTGCGAGCGGATGGATGCTTTAGGGTTTTGGGAGGCGGGCGAGTCGTAGGCCCCCGCGAATAAAGGGACAAAGCGTCAGCGAGGTGGAGGAGGAGAGGCGTCGTGAAGGCGAAGCCGGAGGCCGGAGAGAGAGAGAGAGGAAGGTCGATGATGGCGATGGACTTGGTCCATGCGCTTTGTGCTAGAGCCTAGACGTTGACAGGTGGACCCTCCCAGACGCCGACGCGTGTCCTTACCCAATGGGCTTCGTTTCTGGGCCTACATGGGCCAGGCCGAAAACTTGCTTGGTGTGGTGAAGTGGGCTGAATTCCAGGCCTCACAAACGGAGGGGAAAAAATCCGCTTCGTCGCGAACAAATCTCGCGGAGGGGGGAGGGGAACTGGTGGCCGGAGTGGCGCCTTCGA

>Os04t0692200-02 2000 bp upstream sequence

GGCCCTCCCGCTCGCCCCCAACAATCCTACACTGACGACGACGAGGAAGAGGACGACCAAGACGAGGAGGAGGGGAGCTTCGGTGGGGGCACCCGGGCTGCCGCCATGCCCAAGCCGCCAGCGGGCTTCGTGCTGGACGACCAGGGAAGGTGCATCGCCGCCGCCTCCAAGCGCATCGTCACCATCGTCAGTCTATACAGCTTTTCCTCCTCTTTAGCATTACATTTTCATTTTCATGCCAACATCTTTTCCCTTCTTCTGCGGAGCAGATTGATGACACCAACAACCGGCCCTTGGAGTGCATCATCAGGAGGGTGTTCCGAAGCACGCTAGACCACGACTGCATGCTCCTTTGCCCAGTTGACATGTATTCTACTAATTGCAACTCACTTATTAGTCACTAACTAGGCATATGCATCTTCCTCAATTTTCTCTAGCTGCCACTCAAACCCCCCTCCCCCTCCCATTCTGATCCTTGTCTCTTTCAGGCCTGTGCAGGTCCTTAAGAGTGCCAATTTCAGTGGCTGGATTGCTGTATGTATCTAATCCTCTCACTGCCTTCCATTTTCATCAATCACGCCAATATCATTCATCACACACACTGCCACGCATACATACAGAGACTGATTCTCGCAATATCTCCTTTATTTCCTACCCATATAGTTCCAATTGGAATAGATAGCGTTTCCTCATCTTTATCCATCGCATCATGCATAATTTCTTTTCTTTTCTATGTATATGTAGGTTGATGATGACCAGATTAAGGAAATCATTCCATCCGTTGCATATGCCCTCGCTAGAGTGCATATGCACTTTGTAGAGAGTGGGTAAGGATCATATAAGCCCCAAATCATCTACCCTTCCTGTCGAACTGACAAATATCAGGGTATTCCATTCTTCTTGCTTATTGCTTCCATAACACTATGATCGCTGGCTGGCTTTTCCAGATTTTGTTATACAGCACGGGGTGGCTTTTGCTTTCCTGAAGACGCGATTCAAGAATTTCATGGTAACCAAAGGAACTAACATGCTACTAACTACCCTATTTGCACAAAAGGTTCTGATGATATCTTCTCACTGAAAAGGCTTTTTTTTTCCAACCGTTGTACAAAATAGAAATGTTACTATACTTGTATGATTGACATACCCATGATATAATTGTCTTTTGCACCAGATATTTGCACATGTATGCCTTTTGAATTTCATGCCTAATGCTTACAAAAAATCTAGAAACATAAAGGTAACTTTTAGTTGAAGGACTGGACAACTAGCATATGCAATTGAAACATCTCTTTGAAGCATGCAGCATGTTATGTCTCTTGTTTGATCATTTTTGCACTTCATTGTTGTGTTCATAATTCATATTAATATGCAGATTCTGGTGACGGCGGTGATAGTGTACCTTTTGAAGGTGTAGAGATTTGTTGTTTCAATTTGGTGAGAAAAATGGCTTTGTATCGACATAGTTTCAGAAATTAATGGATCGCTAAATATTCCTTGGTATTTTGTTGATTTGCACATGCTTGAGAATATGTATTTGCTGATATTCCTGGCATCGGCAGGATGGCGCACATTATATGATTTATACACCAGTTGATCCTCTTCTGTTCGTTGCAGTGAAGGTATGTTGGTTGTTCTTCAACTTGTTGAGACATTCCAAGCTAAACTTGTTGAGGCATTGTGCCTGAAAACATACGCCCTTTGTGCAGGATAAAGATGGCGTGCTACGCATTGCTGAAGATGTAAGCAAATATTTAATGCTATACTGACGTGGTTCTTTCGAAGTTAAATTATAATGACGGGTAAGGAATGCCCTCTGTGAACAGGAACTCATGGACGACCCTGCTGTTGTCGGTGCCATAGACGAAGAGACAGAATTCACAGCTTTGGTGGTAAGAGTAGACACCTACTACTTGTTAGTTTGATCATTTACATTCTTATGTGCAAGTTTTTTACATCTACATGTGGATGTGGTGCAGGAGGAGGAAGAGGCCCTTCTT

>Os05t0374500-01 2000 bp upstream sequence

AAAATATTCGTCTCATAAAGTAGTCACAATCTGTGCAATTAGTTATTTTTAGCCTATATTTAATACTTCATGCATGTGTTCAAACGTTTGATATGATATGGTGTAAAATTTTAAGGTGGGATCTAAACAGACCCTCTAATACTAATATGCGTCCTAATCCCTACATTAAAAAAATCAAATTATTATTCAACAAGGTTTAGTAAGTCATATAAAAAAATCACAAGGGCAAAATTATCCTTTCATGTGCCTTCTAACAGCAAGCAAGCCATGTACATTTTTTTGAATTGGGGACAAATAGAGGGAGAGGCAAGTTTGCGAGGGCAAATAAGAGAATCAGTTAACATCTGGGGACAAATATAAAATTCTCTCATGATTAGTATATAGAACTCTCAAGATAATTCTGTTTCTGAATTTGCATTCTTTCTCGCCAAACTTATTCTTGTGCATACTTGTTTATTATATATAAAAGAAGTGCACATCCGCATAAGAAAATCTCACCAAACCAAACATCTTATCTGAGGCACGATTCCTATACACGTAAAATAGAGCGAATCACCAGTGTATAGTTAATTAAGTATTTACTAATTTTTAAAATATAAATTAATATGAATTTTTAAAACAAATTTCGTATAGAATTTTTTTAAAACACATGTTTAATATTTTAGAAAACATGCACATCGAAAATAAGAGCGGTGGGTTGAGAACTCTATCAAAAAGAACACAGCCTTAAAAACGAATCAACAAACGGGTTCATTGTTGTTCATCCTCCAAGAAAAAAAAACAAAACAAACAAACAAAAAAAACCAGGCAACCCCACCCCACCGCACCCAAATTCAAAAAAATCGTCGTCGTCCACAGCTTTACGGACACGTCCCAAACGGTCAAAATCCCCAACACACAGAACAGAATAGGCCCAACCCAACTGCGCGACACGCACCGCCACCCCTCTCCTCTCTCCCGCACCGCATCGCCTTCGCCGCGTTCCCCACGCAACCCCATTCCTCCTCCCTCCTCCGCTTCTCCCCCCCAAATCGGACCCACCCATAACACCCTCCCCTCCCCTCCAATCCCACGCCGCCGCAGCCGCCGCCGCCGCCGCCGGCCGATGACGCCGGCGGTAGCAGCGCCAGCCCCCTACCCCACCACGGGCCAGAACCCTAGCGGCACCCCGCTCCCCGCCGCCGCCATGGGCAACCCCAACCTGGGGCTCGGCCTCGCCTCCTCCTCCGATCACGCCGCGGGACCGCCTCCTCCTTCCCGCCGCGCCCCGCGTCTGGCCAAGCGGCGCCACGCGGCGGCCTCCTCCCGCTCCCGCCAGCCGCCACCGGCGTCACCCGCTGCGGCGCCGTGGAATCCGTTCGGCGGAGGGGGCGGCGGCGGTACGGACGCATCGGGGCAGGATGGGATCGGGGGGATCGCGCCGGGTGGCGTTGGTGTTGGCGCCGGGGAGGGTCAGGACGGCGGATTCGTGTTTGGCGGTGCGGCTTCTGCCACGAGTCAGCAGCCTCCAGTGGCGTCATCCAATGGAGAAGCCCCCTTCGTGTTCGGGAGCGTCAGGGACAGCTTGCCTCGGTTCGACGAGGGATGGGCGGCTTCGGCGAAGCTGCCCGAGAAAATGGGGAGGATGAATCTTCAGACTCGTGGTGAGAGTAGCGTCAATGCCAACAAGAAGGATAGGAGCTCCATTTTTGGAGTTGATATACCTGGTTTGGTTCTCAACAGTGAAGTGAATGTGCTCCCGGAGAAATTGACGCAGTTGAATTTAGGCACCAGAGTGCCGTTGCAGGGTGATAATGACGTGCCAAAAACATTCGTGTTTGGAGGCAATGGAAGCGGGCCTTCTTCTGATGGTACTAGTTCTGATGTCCACCGTGCAGGTTCATATGCTTCTGCCAATGCAAATGGTGTTGCAGAGAAGCTGACACAATATGGTATAGGCAATCAAGCGCCTTTGGGAAGGATGGGCACTGAATCTACCAATGATGCACCACCAGTTTTTG

>Os05t0497200-01 2000 bp upstream sequence

CTGCGGGCTGTGCTGGGTACAGTGCGCACTCGCGCATGCAGGCGCGCAGAGGCATATGCATGCGGTAACATGACATCACGGCCGCGGGCCGGGGTCACGTGAGTAGCCGAACGAGGACGTTTGGGCCGGCCGCACGGCGCTCGTTTTTTTCCCCTGCGGGTGTCAGGACGGGCGGCAGGCAGGCAAAGGCAGCGAGCCGTATACGGCATGGGGAAATTATGGCACACGGCCGGCCGCTGCCTGCCGGGCGGGTCCGGGGGGGTCGCGGCGCGTGGCAGTGCACGGCGCGGGCAGCGGCAAGCGGGCAGGGGTACTGGTACTACTGGTACGTGCGTGCGTGCGTGCGTGGGGCGTCACCGGAAAAGACAAGACGGAGGCGATGCCGTACCGTACATGGTAGGCGCGCGGTAGGCACTATACGGAGTAAAGGTGCCGTGGCGGCCGTGCGAGGCGGTTTATTTGAATGCTGTGCGGCGACAGTACGGGTACAAGGCTTGAAAAAAGCGGTGCTAACATTGATTAGGCCAGGCGTCCTGTGAATCTGGGTTATTAGTTCGGTGAAGCCGTAATGTTTGCTCTGCACGTTCCATTCGTTAAATCTTTTGCTTTTTTTTTGGCATGATTAAATCTTTCAGTTGTCTGTGCAATTTATAACTGTCTACTAGCACCGCAACATTATCTTCATTTGAAAAGGCGAGGGTAAAATAAAATGCTGCACTGACAGGGAGCATCACAAAGAAATGCATGGAATAATAAAGAGGCCGGCCGGCCGGCCGGCCGGGCTGGAGCAGTACTACCGGTCTAGCCGGTCCATCCAAAACAGCATGGGCATGGCTACCCTATACAGTACACACGCACGCACTGTGCCAACGCCTTTGGCCTTTCATCAATGGTACGCCTCTTCCCAAGGCAGGGGTCGTCGCCGCTCGCTGCGCTGCACCATCAGCGCTCGACAGGTCCTCCCGCTGTCCCACCCAGCAGAAGGCAGTCAAGCCTCGCGCGTTCCCAAGGCCCCCCCCTTCTCTCCCTTCCCTTCCTCCCGCTGCTGCTGCTCCTGCCGCGGCAGCCTAGCTAGCTGCGGCCATGCCATGCCCGTCTCTTCCCCAAAGTATTTTTTCCCACATTCAATTCCCCACGAGATTTGGACGGGTCGAGCCGATCCATCGTCGGGTAGGCGCGAGATAGCTACTGCTGCCCGCGGCGCTGCGAATCTACTCCGGACGAGGAGTAATTTGGAGCGTGACAAGCGGCTGGCTACCCACCCACCGAACGGCGTGCGCGCGGACAACGCCGTGCCGGATAGGCCACCCACCCACCGGGGCGCGCCGCGGCGACTGCTACTGTTGAGCGGCGCCGCGCCGCACGCGTCGGCAAACCGCGGTGCTGCCTGCCTGCCTGCCCCCCGCGCCACGACACGGCTCCTGGACCTCCTCGCCCACGTGAACCCGACCCCGGGGGCCGAATCGTGCCACGGCACGTCGCCCCCCCTACGCCGAAACGCCAACCCCACACGTTACCTCCGCCGCGGTTGCTTTCCTTCTTCCCTTCCTTCCATCCGCCTGCTGCCGCTGGCACGTCGGGACGTAGGAGGAGTACTAGTAAACGTACGTACGCACTCGTCACGGGCCCGGTCACGTCCCCTGGGCCGTACTGTATCTCGCTTTCACCCTTGGTAGGCTAGTCCGCCGCGTCAACACGACGTAACGGACAGGGGAGAGGTATGGAGGAGGTGGAGGGCCCGTGCGGTCAGTGTGCAGGGGGGGTAGGGCGTGCAGGACGGATAGGGCGTTGGGGGTGGTGGTAGGGCTGGCGTCACGGCGATTAGACCATTCGGGGCGTCAAATCGCGCGGCGGCTGGGGCTTGATGGCCGAGGCCGAGACGGACACCGCGCCGCGCTGGTCAGCGACAGCCTCAAACCCCCCAAAACGCAGCAGGCCCCAGAGCGGCAGACACCTACTCCTACTAGTACTAGTACTACCACCATCTCCCCATTTCACGA

>Os05t0503300-02 2000 bp upstream sequence

TTATATATGTACGTACCCAAGACAAAATTTTACATCCCAAACAACAAAACATTGTAAAAAAAAAAAAAGTAGCCATACATATAAACAAGAAAAAAAAAACAGAGCAGACGCGTCAGTCCATGGCCTTAATTAGAAGAGGTATCCAAAGGCAAGGGCGGTCAAGGACGCGGCGGCGGCGGCGGCGGCGGCGAAGGCCGGCGCAGCCACCGGCGCGTCGGACGTCGGGGAAGGAGCCGGCGCGTCGGCGGCGGACGCCGTGCCGACCAGCGACGTCGCCAGGACGGCCGCGGCCGCTGCCACGGCGAGGAGCCTGAGCTTCAGTGTTGCCGCCATTTGCTTCCTCGCTTCTTGGTTGACTGATCGATCTGCACTTGGAGAGCAAGAACACTAGAGAGAAGCTGTGATGAAGCTTGTGTGTTTGGGTTGGAGAAGAAGAGATCACTTGAGGCTTATATATAGTGTCATATCAACTTGAGAACTACAAATGCAAGGTGTAGTAGGTAGGAAAGCTAGCTAGCTAGCTAGCCTTGGAAAGAAAGGATTCTGGCTGGAACCCTTCTGGCCTTGATTGGATTGGTTGATTGCTTTGCTGGTGTTTGTTTGGGCTGGCTTAAAAGCTGTGGTCTCCCCATTTGTACTCCTTGCATGTGCTGCGCAATTCGGCTTCTGGCTGGAGATTTAATTGGACAGCCATGATAAAATAGGGTGCATGTTGACAGCTCATGTTAGTACCAGCAATGCGCATTTTTCATGCTTGGCCACACATCAATCGAGCAAAATGATCTTTAGAAAAAAAATGCCCTGATAAAAATGTTTGTTTTAGGCAAATTACTGGGCTCAATTGAGTCAGGTTTAAAAAAGCCAACCCACCTCAGGTAGTGGAAGTGTGTGGCCCAGTTGATGGAAAGGACATCCCATGGATAATAGGCCGTTTGCAGCCCACTACGGAAGCCCAATGGAAGATTTGGCCTGGGCCGTTTGCAGACCACGATAGCAGTCCAATGGGATATTTGGCCCAAAGTGTGTAGCGTGTACCGTCCCTTTGTCCAAATCTTCAAAGATTTTGGTGTCCACGTCTAAAGGAAAAAAGGGAGATCGTGACGTCGACAGTTGACAGCACAAGGGCATCGAATTTTTTTTAGATAATACAAGGGCATCACATAAAGGGAAAAAGAACAAACAACAGGATACAGATACGGAGCTGTTACCCGTATAAATCAACAAGATGCGTGTGGCCATCTCAGATGCTAGTAGTTCAGCATTTTGCCCTGATAGATAATACTGTGCTGCTAATACGAAAATTATATTTTGGCTGCTGATGGGTATGACACGAGAGCAGCCAAAATCCGGCCGGTTTCCATGGCCAAGAGACACTATCAATCGCGGTAAAACAGAAGTGGCCATAAGGTTGAGGCACACAGACAATCTCTGATCTGAAATTCTGACCTCACTTCATGCGGCGGGAGGGCGGCTGCTAGGGAAGAGAAGGGAATCAAAAGCTTTTTTTGTGAGGACAAAGACGTCCAGGTTCAATGCACCCACACAAATCCCCGGGCTAATCCGGAGCCCTACCCGTCGAATCGCACGCACACCTACTCGCGCATCTCATCCTGGTCCTCCTCTGCCTCTGCCTCCAGGCCGCCTCCACACCACTTGGCGGCTATCCCCGGAGTCACTGCCACGTGGGGCCCAGCACGGGTGGCCCCACCTGTCATCCTCCCGCGGTGACGCGCGTGGGCGCCACGGTCGTCAGCGCTGATGCGATGCTCGTCGCCACGCCACTGCCGGAAGTCTTCTCTCCACCCTTGCCCACCTGAACCCGCGTCCATACGCCCGCGGCTCTATCCTGTGTCACTGACAGTGGGACCCACCACCGGTCTCGTTGAGTGGTCTACCGTGGACGGCGCGCACGGTGGGGCCAGGGGCGCACTCCCCGGCCCGTAGGAGTAGACCCGTACGCCATTAAAGCCCCTCGACCCCGACCGAGCGAAGCGCCCCGC

>Os05t0503300-03 2000 bp upstream sequence

ATTGTAAAAAAAAAAAAAGTAGCCATACATATAAACAAGAAAAAAAAAACAGAGCAGACGCGTCAGTCCATGGCCTTAATTAGAAGAGGTATCCAAAGGCAAGGGCGGTCAAGGACGCGGCGGCGGCGGCGGCGGCGGCGAAGGCCGGCGCAGCCACCGGCGCGTCGGACGTCGGGGAAGGAGCCGGCGCGTCGGCGGCGGACGCCGTGCCGACCAGCGACGTCGCCAGGACGGCCGCGGCCGCTGCCACGGCGAGGAGCCTGAGCTTCAGTGTTGCCGCCATTTGCTTCCTCGCTTCTTGGTTGACTGATCGATCTGCACTTGGAGAGCAAGAACACTAGAGAGAAGCTGTGATGAAGCTTGTGTGTTTGGGTTGGAGAAGAAGAGATCACTTGAGGCTTATATATAGTGTCATATCAACTTGAGAACTACAAATGCAAGGTGTAGTAGGTAGGAAAGCTAGCTAGCTAGCTAGCCTTGGAAAGAAAGGATTCTGGCTGGAACCCTTCTGGCCTTGATTGGATTGGTTGATTGCTTTGCTGGTGTTTGTTTGGGCTGGCTTAAAAGCTGTGGTCTCCCCATTTGTACTCCTTGCATGTGCTGCGCAATTCGGCTTCTGGCTGGAGATTTAATTGGACAGCCATGATAAAATAGGGTGCATGTTGACAGCTCATGTTAGTACCAGCAATGCGCATTTTTCATGCTTGGCCACACATCAATCGAGCAAAATGATCTTTAGAAAAAAAATGCCCTGATAAAAATGTTTGTTTTAGGCAAATTACTGGGCTCAATTGAGTCAGGTTTAAAAAAGCCAACCCACCTCAGGTAGTGGAAGTGTGTGGCCCAGTTGATGGAAAGGACATCCCATGGATAATAGGCCGTTTGCAGCCCACTACGGAAGCCCAATGGAAGATTTGGCCTGGGCCGTTTGCAGACCACGATAGCAGTCCAATGGGATATTTGGCCCAAAGTGTGTAGCGTGTACCGTCCCTTTGTCCAAATCTTCAAAGATTTTGGTGTCCACGTCTAAAGGAAAAAAGGGAGATCGTGACGTCGACAGTTGACAGCACAAGGGCATCGAATTTTTTTTAGATAATACAAGGGCATCACATAAAGGGAAAAAGAACAAACAACAGGATACAGATACGGAGCTGTTACCCGTATAAATCAACAAGATGCGTGTGGCCATCTCAGATGCTAGTAGTTCAGCATTTTGCCCTGATAGATAATACTGTGCTGCTAATACGAAAATTATATTTTGGCTGCTGATGGGTATGACACGAGAGCAGCCAAAATCCGGCCGGTTTCCATGGCCAAGAGACACTATCAATCGCGGTAAAACAGAAGTGGCCATAAGGTTGAGGCACACAGACAATCTCTGATCTGAAATTCTGACCTCACTTCATGCGGCGGGAGGGCGGCTGCTAGGGAAGAGAAGGGAATCAAAAGCTTTTTTTGTGAGGACAAAGACGTCCAGGTTCAATGCACCCACACAAATCCCCGGGCTAATCCGGAGCCCTACCCGTCGAATCGCACGCACACCTACTCGCGCATCTCATCCTGGTCCTCCTCTGCCTCTGCCTCCAGGCCGCCTCCACACCACTTGGCGGCTATCCCCGGAGTCACTGCCACGTGGGGCCCAGCACGGGTGGCCCCACCTGTCATCCTCCCGCGGTGACGCGCGTGGGCGCCACGGTCGTCAGCGCTGATGCGATGCTCGTCGCCACGCCACTGCCGGAAGTCTTCTCTCCACCCTTGCCCACCTGAACCCGCGTCCATACGCCCGCGGCTCTATCCTGTGTCACTGACAGTGGGACCCACCACCGGTCTCGTTGAGTGGTCTACCGTGGACGGCGCGCACGGTGGGGCCAGGGGCGCACTCCCCGGCCCGTAGGAGTAGACCCGTACGCCATTAAAGCCCCTCGACCCCGACCGAGCGAAGCGCCCCGCACGCACGAAATATCCCACTCGCTTCGACACTCTCTTCCCCTTCTTCTCCC

>Os06t0178650-00 2000 bp upstream sequence

ACCTACCAACTTGTTTACATAAAATCTTTCATCTTGACTAAACCCTGACCAATTTCACTCTGTCATCATCCATGGCCACAAAACAATGCCGAAATAAGATAAAAACCGGCTGGCTAGGAATCACCTGACTGAACAGAAGCACTGCGCGTTCAGACAACCATGGCTGACGGCCACCACTGGAACATTGTCAAGATACCGCCGATTGTGCAGGAGCTCGCCGCCGGCGTCCATGAGCCGCCGAGCCAGTACATGGTCGGCGAGAAAGACCGCCCTGCCATCGCCGGCTCCGACATGCCTGAGCCCATCCCCGTCGTCGACCTCAGCCGGCTGTCTGCCTCCAATGGTGAAGACAGCGCCGGCGAGCTTGCCAAGCTACGCTCCGCCTTAGAGGACTGGGGCCTCTTCCTGGTAAATAAAAGTACCACTGCATTCGTTTCATATTATAAGTTATTTTGACTTTAGTTAAAGTCAAACTGTTCCAAATTTGATTAAATTTATAGACAATTTTGGTAATATTATAACATCAAATTATTTTCATTAAATCTATAATTGAATATATTTTTATAATATAGTTATCTTGTGTTGAATATATTACTATTTTTTTCTACAAATTTAGTTTAATTTGGAGTACGTTTTTCTTTAACCAAAGTCAAAATGACTTATAATCTGAAACGGATTGAGTATTACTTTTTTTTTTTGGGTACCTATGGTACTATAAGGTGCAGGGTTATTTGGTACTATAATGGAGGGGAGCATTCTCAGTGAGATGATCAATGTGACAAGAGGATTCTACAAGCTCCCACTGGAAGAGAAGCAGAAGTACTCCAACCTGGTGAACGGCAAGGATTTCAGGATCGAAGGGTACGGCAACGACATGGTCGTGTCAGAGAAACAGATCCTGAACTGGTGCGACCGGTTCTACCACATCGTTGAGCCTGAGTCCCGGATAGCCCATAGCCTCTGGCCAACACAGCCTCCTTCTTTCAGGCACTGAATCTTCCTAACCTTGATTAATCCATTGTTCACTCTGCAATCTACACGCATGATTAAGAACTTAAAATCTGTCAAGTTCTGCCAGTACATTTGTCTGAAATTTCCTGTTTCTTTTATACAGAGATGTTCTGCATGAGTACACTATGAGGTGCAGGGAGATCACCAGCCTTGTGCTCGCCAGATTGGCCAGGCTGCTCGGCCTGCGAGAGGGCTACTTCGTCGACATGTTCGACGAGGACGCCACGACGTACGCGAGGTTCAACTACTACCCTCGCTGCCTGAGGCCGGAGGATGTGTTGGGCCTGAAGCCACACTCCGACGGCTCGGTGATCACCGTCGTCTCCGTCGACGACACCGTCAGTGGGCTTCAGGTGCTGAGGCAAGGCGTCTGGTACGATGTGCCCGTCGTCCCCAATGCTCTGCTCATCAACATGGGGGATGGAATGGAGGTATATATACATTTACTCCATCCGTTTCACAATGTAAAACTTTCTAGCATTGCGCACATATATCTAGACATATGTATGTATTTAGATTCATTAACATCTATATGTATGTGGGCAATGCTAGAAAGTCTTACATTGTGAAACGGAGGGAGTACTTGTTAGTGGTTGTTTATCCAAGGATACTGCACCCATGTAACCCAAACCAAAATATTACGGTGTTCCAAAAGTATTCTACCATGATTCCTAAAGTATATCATGATTCCTTTTATCTTCTCCTGAAGAAATCTTTGGAGGGATCAGAGATTTGGGAGATCTGAACAACAATCTGAATTGCACGTAAAGTAGACCTGTAAAGCTTCAATTTTGAGTACATCATCATTTACACGTCTGATTATATTTGATCTTTTAGGGAATTAGTCCGACTAAGTAGCAGAGGTCGGAGATGTAAATCATTTCCATTATCTAAAAAGATATGACTAACAGACACCTATTCTGGGATGCAGATAATGAGCAATGGGTTGTTAAAGAGCCCGGTGCATAGGGTGGTGACGAACGCCGAGA

>Os06t0214100-01 2000 bp upstream sequence

TTACATTCATAGAGACATATTTGAGCCCATATCACCTAGGTTTGCAAAACTTAATTTAGTTGCTTAGTTAACCTTGCATCTCACCTTGCCTAGCAACTTAGGTTAGTTTTGTTTAAGTGCCATTAAGTTTTAAAACCGCCTATTCACCCCCCTCTAGTCGGCCTCCTTGATCCTACACAAGCCAAAACGAGGGCGCGAGAAGGAGAGGAAGAAGCGAGGTGCGCGCGCTGTGGGGTGCGGTTCTTGGCCGCGCCGTCCCCTCACGCCGCATCAAGTGAAGAAAACCCCAATTTGCCCACGATTCTTCCCGCCACCAAAACACCCCAAGCAAGAACGCAGAAAAAATTACACGATCCGTCCCAAGAACACAAGAGGAATCACCAAGAAACAACACATCTCACATGCAAAAAAAACAACAATCAATAATCTCGCCGCCGCGCGCCAGCTTCCTCCTCACCGCGGCTTCCTGTCGGCGTCGCGCCGTCCGCTCCCGCCATGGCCACCACATCCGCCGCCACGACCCCCAAATCCGGTGGCGAATCCGGGGCCCCGCGTCGACGACCGCCACCACAACTCGGATCCGGACGAGCCAAGAACTGGCGGCCGGTGTCCGCAGGCCACCAAGAACCGACGTCCGCGTGCCGCCAAGAACTGGCGGCGGCGGCGGCGGCTGCCGCGCTCCCCGTGCCGCCGGTGCCGCGCGGCTCCTCCACGGGCGCTTGTGGCTCCGCCGACGCCGAGCCGCTGCAGCTGCGACGCGGTGAAGTAAGGGAGTGGGGGAGGGAGAGGGGAGGTGCGGCGGATAGATCCACCGCCGCCGCTGTCGCCACGACGACCGGCGAACCCACTCCTCCTCCTCGCGACGGCGGCGGCAGCTGCTCGAGGTCTCCCTCGGTGAGGTCGCCGCGGTGGAGGCGTCTCGCTACCGCAGCGGCCGGAGAAGGAGGCGCGACGAGGGAGGCGATGAGGACGACGGGAGGCGAGGAGGACACCGGAGCAGCAGCCTTTCCCCTCCTCCTCCGCCGCCCCCGTCGCTCCCACCGTCGGGAAAGGCGCGGCGGCGGACCGGGAGGAGGCGCGGCGGCGGACTGGGAGGAGGCGCAGCGGCGGACTTGGAGGGGAGCGGCGTGGGAGGCTAGGAGGCGAGGAACGGCGTGGGAGACGAGGAGGACGACGGGAGCAAGGGTAGGTGAAGGAATAAATTTCCTTCACTCGCCCGTGTCTTTAAAACCGGGTCACGCGCGTTGTGGGGTTTATTCCCTTGCACAGTATCTCCATCCAGTTAGGTCACGCAAAATAGGATACATGCACTGTGGGAATGAGTGTCTTTGGGGCTTTTTTTTGGAGTAAGCTACCGGGACCGGTGCTTGCACTGTGAATGGCCTAAAAGGATCAAAGACCACCACCCATGTGGCCACGTATGGTGGAAAATGAACATCCTTGTTTTGAAAGTTTCTGCAGGCTGGCTGTGTCCCTTCACAGTTTCATTTGACCATTTTCTTCTTTCTGAAGAGAGGAACAAAGTAAGAGACACACTCACGGATCCACACATATAGAATAATGGTTTCCACCAAATTACCAAATCATTAGCACAGGCCAGCTCGACAGTAAAAAAAAAACATTCCAACCAGGCAACCATACCAACCAGGCCAAATAATCCCAAAGATGGTACGGTAAACTATGACTATTTCCCATCACTTCGCGGTAAAAAACGAGGTGCTCACATTGTCGTACTCCAGTACTCCACTATCGCTGCCACGACCATTTCTAATCGCCAAGATCGTATCACAGCCGTCCACGTCACCCCATTACCCCCAACTCCATGCCTCCGTCACGCCAACCCAACGCAAACCGTGACTTTCCGTGGGCCCCACCACCCCACCACTCACCACCCAACTAAAGTACGTGGGACCCACAAGACAGGGATACTTGGGACCCACGTGTCAGGGAGCAAAAGTCCCCCTAGGTCGAGCCGCGCATCACCGCCTCAGGTGAAATTA

>Os07t0170300-01 2000 bp upstream sequence

GCCGGCGAGCGGTGCACACCGGAGAGGAGGACGATGACATCTCGGCGGTTGAGGCGAAGGTGTTGCTTTGAGCCTCGCCTCACCAGCAAGGAGGAGGTGTTCACCTAGACCAAGAGCAACAATCAACGACTGCTCCAAGTTGGCGACATTGACAGAACAAGCAAGTAAGGAAATGACACTCCAATTTGTATCTGCATACTCTCCTTCAACTTCACGCGTGAAGCCCCAATTTCTTTTTGAAAATCAAATTAACATGCAATCTACAATATTACCTGGTCTGAATTGCTAGGCTGTACAGGTCCTACATTTGCACATCGTGCTCTATGTGGCTGGCCACAGAGAATAGGGTGGAGTCAGCCGATGATGGAGGTTGTTTGCTATTTTTCTTTTGCTCGTCGTCTCTATGACATGAACGAGAATGGTTTTGCTTTTTTTCTTCTTGCCAGATAATGGATGGCTACTACTATGCAACGTCGAGCTCATCTCTATACCACACCGCTACATATCCTTCCGTGATTCTATCACCGACTGCCGCTCGCTACCCGGTTGCCCTCTGCACCCCCTGTCATAAGACACGGACCAACATCTCCATCCTTATCGCAAGATCCAGCAAAGCCACCAGGATCTCAGCTTCATGATGGTTTTTAGGCTGGACCATTTTTTCTTTTCTAGTTTGATTCACCTGTGAGTAATTTTATCACTAATGTGTGATTGAGCCTATAAATATTTTATTCGTATATTCATTTTGTGATTCAGACTAACAATACTTTTATTATCAGTTTTTACTCCTATACTATGTATTGAACACATCAGTAATCATATTACTGTATGGTGGATACGAGAACATGGGCTATATAAAGAGGAATGTGGTAATTGTCTTAATTCCCCGTGGAGGTTCTGGCTCTATTACTCGTCAAGGCCGGCCTGACTTCACTAGCATCTCCTCTGCCATGAGCTCGCCGGCGCCACATCCATCTGCACTATCGTCGCCGCCGAATCTCTCTCTCTCTCATCATCGACACGGATTGCTCCACGCAGAGGCATAGCTTCTTGCATGCTGAGCCGCCATCATCCTCGTCGCTGGAGCAGCAGCACGACGAGTCCATCTGGAACATGACGATGTTCGCGCTGGGGGTAGCGTTGCTCGTCGACCACCGGCCCTATCCATTCGCCATAGAGAAAGGGGAAAGGGGAGAGAGAAACAGAGGGATGAGTAGAGAAACCGGGTCACCAATGATGCCACGCATCGCTGCCGCGCATCTGACCTACCCACCCATGCTCCTGCTCTTCCATGGCGGCGGCGACACGCACCCTCGCGCCACTCTCATCCGCCTCCGCATATGTCGGCCTCGTGCGCGCCTTCGCCCGTCGCCCGTGTTGCCTCTCGATCGCTGTTTGGGGGAACCCTCACCCTCCATGGCACCTCCTCCTACGCCAGCACCGCTGCCGTGATGGGGAAGGCCAGCACTGTGGAAGCCGACACTGGGATGGACACCGTTCAGCGGCGACGCCGTTAGTGAGGAGGAAGGAGAAAGGAGAAGAAAGGAAGGAGAGATGAGGATGACATGTGGGCCCCGCATGGGGCCCATCATTTTATTATTATTTATTAGGTTTACCTGACATGTGGGTCTTATGGTTTTTTTTTTTCCGGATCAAATTGCCACGTCAACACGATATGGGACGAAGACCTAGTCAAAGGAGCCACGTAGATCAAAACTGGAGACAATACCGTTGAGAGATCTCAGTTTTACTGGTTTTGTAAGTTGGAGGATGCGTTGTATCCGGTTTTGTGGTTTAAGGACGATTTTGTAATTCAGCTGACAAGACGAGGGATCTCTGGTGTACTTTTTCCTTTGCTTTCTGAACAAACATCCGGTTAGGGGCAGCCCACTTCTTAGTCTCGACGCAGCCCGGCCCATCAAACCATGGGCCCCACACGGCAGCAGCCCATCTCCCACCACACATATAACCCAGCGACGTGGCCTCCGCTCGCTCGCCTC

>Os07t0181500-01 2000 bp upstream sequence

GTTTTTCATATTCCTCTGTTTTTTCATTCCTGTTATTCAAAGGGGCCCTAAGGGCTTGCTTGTTTGTGTTGTGGCTTATAATTTTAAATTTTAGAACTTTATTTTTAAAATTAAATTAAAGGTTTTGTTTTATAAGTTTATTTTCATTTGGGTTTGACTTTTTTTAATGAAACACATTACAAACGAAAACGCTCATAATATGTGCACACTCACATCTATAAATGCACACACACATCCTACTCCTATGAGCACCTTGAGAAGATTGAGTCCAACATGATGAAGTCACCACGGATGTCTCGCTGTTGATGGATACGTCACTTACCACTGACAGAATAATTAGCCGCAAATGTGAGCACCCATGTCAATTTTAGAACTTGAACCCGACTAGGTTGGTTTCAGCAAAATAAACTAACCAGTTAAATTATACTCACTTCACCTTTTGGGTTTGACTTTTGACCGCATATAGCAGAAAGGCAAAAGCCTTAATTTAAAATTACAATTTTCGTTATTATAGAAAAATATATTGAAAAGTACAACCACAGAGTTTATAACTTATTTCTATATATATAGAGAGTAATAATAACATAGTTAAAATGTATACTTGCATGTTTAGGCATGGCGGATAATAAAAATAAAAAATAAAGCCCAAAACCCTATCGAAAAGATCAAGGCAAAATTTACTACAGAATACTTAAAAAACGTGTAATTAGTTGTGGGACACCGCAAATATGTGAATTGGCTATAGGACATCCCAAAAGAGTGATAATTAGCTACTAAACACCGGAGACATTATTTTATCATTTCTGGTGGAAACGGAGAGAGGAACAATGGAAAAAGGACCAGATTTCCCTATGTGAGCCAAGGATGTCAGCATCTCCACTATTTTCATGTCCTCTTACTCCTGATGAGGCACACCACACGCAATACGGCGCCGCTGACACCGTCCTTCTCCATCACGCCGGCAGGCTCGTACGCAAACGTGGGTAGGGCATCGATGGCGTGATAATTAGCTACTAGACACCGGAGACATTATTTTATCATTTCCGGTGGAAACGGAGAGAGGAACAATGGAAAAAGGACCAGATTTCCCTATGTGAGCCAAGGATGTCAGCATCTCCACTATTTTCATGTCCTCTTACTCCTGATGAGGCACACCACACGCAATGCGGCGCCGCTGACACCGTCCTTCTCCATCACGCTGGCAGGCTCGTACGCAAACGTGGGTAGGGCATCAATGGCGTCGCGGACACTCACATCGTGCCCACTATGCCACGTCACGCTCTCCGCGCGCACCCTCGCCGCCCTCGCCGCCGCCTGCGCACAGCGATGCAGCCGCAACCGGCGAGGACGGCGACGCCAATGCAAGGCGGTAGGTGCTGCATCGACGAGAAGTGGTGCTATTCCATCGGCTCCTTGCCGTGGCCGCCAGAGCCAGTGCCCGAGGCAGCGGCTGTTGCTCGGCTTGCTCCTCTCGCCTTGCTTGCGCCTGGGCTAGCATCTGATGCACCTGCACCGCCACAGCCCCAGACGGCGGCTTCTTGATGGCATGCGACTCTAACACAAGCAAACGACACGGGGGTATGTTGGTACTTCCAGCGCTCTTTCTCTCTCTGTTTCCACTAGAATAATAAAATAATGCATCAAGTGTCTAGTAGCTAATTACCACTATTTTATAATATCCTACAGCCAATTCACATCTTTATTGAGTCCCACAGCTAATTACACGTTTTCTGAGGGTCCCGTAGCAAATATTGCCAAAGATGAAAACCACATTTCCATCAGCGCGCGAAATCCCAATCCTACCCCTGCACAATGACATCCACCGAGGGGCAAGCCCGTCATTTCAGCTGCGCCCACGGAGTCCACTTCCCCCTCCTCCCCCACAACGGCGACAATACCGTGAGCCACCCGCCGGGCCCACCTGTCAACGTCCCCTCCACCCACTACGCCACGTCACCCCCCTCCCGCCTTTATAAAACCTCGCGAACAAACTCCTCGTC

>Os07t0294800-01 2000 bp upstream sequence

AAGAAGAATGTGCACGGGGGCGGCTGGGGTTCTCACCGCTGAAACGGGTGGTGATCAAGATCGGCGCAGATGAAAGGGGGCATCCCTTTAGGGTCTAACGAACGGAATATCTACACTAGAAGGTAGGTGCATTGGGCGGGGGGGGGGGGAGGATTTGTGCTTGGGCAACATGTCCTTGGTGTTGCCTATCATCTCTTAAAACTAGGTCAGAGAATTGGGAAATAAATAGTTTTATTGACGAGCACACATGTCCACAAAGGAAGAAAAACAAGCTTGTCACTTCAAGGAGAATTGCTGAGAAGCATGAGAACGTGATCAAAGCAAATTCACGGTGGAGTTTGAATTACATTCAGACTACTATAAGTGAGGAGATGTTTGCCAATGTTAATATATCCAAGATCAAAAGGGCCAAAACTTTAGTGACGAAGAAAATGCTTGATGACAAGAAGGGTGAATACGGTTTGGTGTTTAATTACCGAGAAGAATTACTGAGAAGTAATCTACGAAGCACGATGGTGGTAAAACTATGGCAAAAAATTATGCGCTGATGTGGCGAAGGGCTGACGTAGCATCCGACATGTCATTTAGTTGTGCAAAAACCATGGCAAAACAGCTCATGCGGGGTGTTAAAGTTTCCAGTATTGATAGTTGGGAGGTTTATTATACCCGCTCACGTAGTTCAGGGATTTTAGACAAGCATAAGACTTCGAGGGTCTAGAATAGACTTTTCCATTTCCTATTTTCCCTTTTTTTTATTCATTTCCTTTTCCCCTCTTTGTTTTGCTCACATTTTAAAATTGGTGGGCCGGATGTGGTAGTGTGTAGACCTCTAGCTTCATGCCAGATGGTCTAGTGGTTTGCAATGGTTTAACATGGTTTCTGCACACCTAGATGACACTGATGCCACGTCAGCCCTTTGTCATGTCAGAGCATAAGATTATACAGTAGTTTTATTATTACTGTGCCTCCCAGATTACTTCTTAGTAATTCTTCTTGTAATTAAACACCAAACTATATTCACCCTTCTTGTCATAAAACATTTTCTTCGTCACTAAAGTTTTGGTCATATTGATATTGGATATCTAACATTGGCAAACATCTCCTTACTTATAGTAGTCTTAATGTAATTCGAACTCCACTGTGGATTGGCTTTGATCATGTTCTCATACTTCTCAGCGTCCAATCATACTTATAGTACCAAGGACATGTTGCCCAATCACACTTGGCTCTAATTCTGCGACTCATCCTTTGGAAAGTTTATAACCCTCCTTTCAGCCAAGGTATACTGAATTATTACATTCTTAAATTCCATTGTAGTACTGAATTTCATTGCCAAAGAAAATGGAGAGTTTGAAGCATTGCTATTCATGACGTACCAACTCAGCACTCAGCACTGCTTCCTTTTTCATCGAAAGAGGCATCATCATCACTTGAGTCATAATATGCACAATTTTAAGGATTAAAGTTAATGCCACGTCAGGGAGGGTCTATATAGCATACCACGTCAGCGGCGTCGCGTAAGATCTCGGAGATGTCCTAATAGTTTAGCACCGGAATAACATGATACAATAAGTTTCATAATAATATAACAGTAGGAGGGAGGGTATGGCCAAACCGACCCCACCCACACCCGGGGTCAGATCAGATTCCCTATCACCAGGGCAGGCTCGATGGGGAGGCGAGGTGGCTACTTGGCTACTAGACGCCAACGCGCGCACCTACCTACCGTGAAATTTTCCCTTTTCTCCGCCGCGGCGCGAGGGACAGCGCAATCCAAACCGGCCACTTCCACACGAGGCCTCCCTCTCGCACCGCAACCAACCAAACACGACGGCGACCTCCTCTCCCCCAATAATTCCCATTCCTTCGCTCTCGCCGCCACCCCGGCCCGCGCGCGCAAGCAACCTTACCTTCCCATGCTGCTGTGATGTGTCATACGCCGCCGCCACAGAGCAACCGGCCGTCTTCTTCCTTCCTTCTTGGTCTTAGCTGAGCTGCCC

>Os07t0418200-01 2000 bp upstream sequence
[truncated: 34,737 more chars]
